# Evaluation of the Global White Lupin Collection Reveals Significant Associations Between Homologous *FLOWERING LOCUS T* Indels and Flowering Time, Providing Validated Markers for Tracking Spring Ecotypes Within a Large Gene Pool

**DOI:** 10.3390/ijms26146858

**Published:** 2025-07-17

**Authors:** Wojciech Bielski, Anna Surma, Michał Książkiewicz, Sandra Rychel-Bielska

**Affiliations:** 1 Department of Gene Structure and Function, Institute of Plant Genetics, Polish Academy of Sciences, Strzeszyńska 34, 60-479 Poznań, Poland; wbie@igr.poznan.pl (W.B.); asur@igr.poznan.pl (A.S.); 2 Department of Genetics, Plant Breeding and Seed Production, Wroclaw University of Environmental and Life Sciences, Plac Grunwaldzki 24A, 50-363 Wrocław, Poland; sandra.rychel-bielska@upwr.edu.pl

**Keywords:** flowering, white lupin, indel, promoter, FT, vernalization, AGL15

## Abstract

*FLOWERING LOCUS T* (*FT*) is a key integrator of flowering pathways. White lupin, a grain legume, encodes four *FT* homologs: *LalbFTa1*, *LalbFTa2*, *LalbFTc1*, and *LalbFTc2*. Widespread distribution of white lupin implies diverse phenological adaptations to contrasting ecosystems. Recent studies highlighted associations between *FT* indels and flowering regulation. Therefore, we surveyed the global white lupin collection for the presence of such indels and potential links to phenology. A panel of 626 white lupin genotypes, representing several European and African agro-climates, was phenotyped under a long-day photoperiod in a two-year study, showing up to 80 days of flowering time difference between early landraces from Eastern Mediterranean and late accessions from France, Madeira, the Canaries, Greece, Italy, and the Azores. As many as seventeen indel variants were identified for *LalbFTc1*, twelve for *LalbFTa2*, nine for *LalbFTa1,* and four for *LalbFTc2*, yielding roughly three hundred allelic combinations. Significant correlations with phenology were confirmed for one *LalbFTa1* indel and twelve *LalbFTc1* indels. A large, highly correlated *LalbFTc1* indel was revealed to be conserved among all domesticated Old World lupins, carrying all *FTc1*-promoter candidate binding sites of the same major floral repressor, AGAMOUS-LIKE 15. A small *LalbFTa1* indel, providing additional contribution to earliness, showed homology between white and yellow lupins. *LalbFTc1* indel-based PCR markers revealed high discriminatory power towards early (PR_42a and PR_71b) or late (PR_58c, PR_36b, PR_80, and PR_60b) flowering.

## 1. Introduction

White lupin (*Lupinus albus* L.) is a grain legume with a relatively long history of cultivation, dating back to ancient Greece and Egypt [1,2,3,4]. This species, currently growing in a wide range of environments from East Africa and Western Asia to Madeira and the Azores, originates putatively from the Eastern Mediterranean region, where wild *greacus*-type accessions can still be found [5,6]. Since ancient times, white lupin has been partially domesticated and distributed across numerous countries, including Italy, France, Spain, Ethiopia, Germany, Switzerland, Poland, and Australia [7,8,9,10,11,12]. As a low-alkaloid crop (min. 0.02%) with high grain protein (up to 38%) and moderate seed oil content (10–13%), characterized by a favorable ratio of omega-6 to omega-3 acids, white lupin has been recognized as valuable for human consumption [13,14,15,16,17]. White lupin evolved in the temperate climate of the Mediterranean Basin, and as a result, it developed natural adaptations to this environment. One such adaptation is responsiveness to vernalization during the vegetative growth phase, which allows synchronization of flowering time with seasonal weather patterns to enhance the chances of successful reproduction. This mechanism has been observed in numerous white lupin landraces across many countries. For instance, a typical vernalization-responsive white lupin “winter” ecotype achieves flowering competence after prolonged cold exposure of autumn-germinated plants [18,19]. Moreover, as a natural adaptation to summer seed dispersal and subsequent germination before the onset of winter, vernalization-responsive white lupin accessions have developed a degree of frost tolerance and cold hardening ability [20,21,22]. Aside from the winter ecotype, the global germplasm pool of white lupin also includes representatives of a vernalization-independent “spring” ecotype, which is characterized by rapid flowering and low frost tolerance, along with a variety of intermediate forms that show moderate responsiveness to vernalization [18,22,23,24,25,26,27,28]. Breeders have exploited both ecotypes to develop cultivars aligned with the local agro-climatic conditions. The winter ecotype is sown in autumn in regions with mild winters, such as the Mediterranean Basin and Western Europe. In contrast, the spring ecotype is sown after the frost period ends in colder regions, which include central and eastern parts of Europe, the western part of Asia, and the northern territories of North America [7,29,30,31]. In France, the winter ecotype is also effectively intercropped with triticale, significantly suppressing weed growth and increasing total grain yield [32]. In Australia, autumn sowing of white lupin is carried out using vernalization-independent spring accessions to guarantee early flowering [8,33]. In Greece, efforts to reselect early-flowering white lupin germplasm have recently been undertaken [34].

Current studies revealed quantitative control of flowering time in white lupin, evidenced both by linkage mapping of quantitative trait loci (QTLs) in a recombinant inbred line (RIL) population and by a genome-wide association study (GWAS) in a germplasm diversity panel carrying landraces from various eco-geographical locations [10,27,28,35]. In addition to several single-nucleotide polymorphisms (SNPs) and presence/absence variants (PAVs) from different regions of the genome, significant associations with white lupin phenology were also revealed for the newly identified insertion–deletion polymorphism in the promoter region of the *FLOWERING LOCUS T* homolog, the *LalbFTc1* gene (*Lalb_Chr14g0364281*) [27,35,36]. Similar observations were made for two other Old World domesticated lupin species, narrow-leafed lupin (*L. angustifolius*) and yellow lupin (*L. luteus*), up to ~6 kbp upstream of the start codon [37,38,39,40,41,42,43,44]. Moreover, in yellow lupin, besides the *LlutFTc1* promoter deletion associated with early phenology and vernalization independence, indels in two other homologs, the *LlutFTa1a* and *LlutFTc2* genes, revealed remarkable associations with flowering time [37]. A question has arisen regarding the level of evolutionary and functional conservation among *FT* indels in the white lupin genome compared to other cultivated Old World lupin species.

To answer this question, we developed a PCR-based marker array spanning the promoter regions and selected introns of all four *FT* homologs in the white lupin genome. We implemented this array to assess the newly established large germplasm diversity panel, which comprises 626 genotypes from 32 countries and territories, representing all regions where white lupin currently grows. In parallel, this germplasm panel was subjected to a two-year controlled-environment phenotyping for plant phenology phases. Then, the identified indel polymorphisms in all *FT* homologs were correlated with the observed white lupin phenology. The shared microsynteny of three Old World lupin species was analyzed in genomic regions where significantly correlated *FT* indels occur.

## 2. Results

### 2.1. Over 80 Days of Flowering Time Difference Was Observed Between Early and Late White Lupin Lines

Data on phenological phases were obtained for all studied genotypes, with a total of 626 lines (Supplementary Tables S1 and S2) in 2020 and 2021. In the first year of observations, the mean number of days from sowing to floral bud emergence ranged from 36.0 to 111.7, from sowing to the start of flowering between 41.3 and 127.0, and from sowing to the end of flowering from 62.0 to 129.0. In the second year, these values were similar, ranging from 33.0 to 114.7 days to floral bud emergence, 40.7 to 121.0 days to the start of flowering, and 54.0 to 128.0 days to the end of flowering. The earliest genotypes included several landraces from Turkey, Syria, and Jordan that developed floral buds 34 to 37 days after sowing and outperformed all domesticated materials in earliness. The earliest cultivars were Butan from Poland (with a mean value of 37.5 days from sowing to floral bud emergence), Lotos from Russia (38.6 days), and Terre from South Africa (38.7 days). In general, early-flowering domesticated materials originated from Russia, Israel, Chile, Hungary, Poland, Greece, Belarus, Germany, Ukraine, and Italy. On the other hand, the latest lines were winter-type cultivars from France—Adam, Aster, and Luxe (104.1–112.8 days)—that developed floral buds about 4–8 weeks later than late-flowering landraces from Madeira (58.7 days), the Canaries (59.6 days), Greece (63.7 days), Italy (64.6 days), and the Azores (76.5 days). High correlation coefficients between years were revealed, reaching 0.97 for days from sowing to floral bud emergence, 0.96 for days from sowing to the start of flowering, and 0.95 for days from sowing to the end of flowering. Slightly decreasing correlation coefficients for subsequent developmental phases may indicate the increasing influence of environmental conditions on the phenology of plants during their growth in the greenhouse. The automated DNA isolation protocol yielded an average concentration of 810.7 ± 329.9 ng/µL DNA (Supplementary Table S3).

### 2.2. Nine Indels Were Identified in the LalbFTa1 Gene Region, Including One Significantly Correlated with White Lupin Phenology

The white lupin pangenome [45] was screened for the coverage of short-read sequences provided for 39 white lupin varieties (https://www.whitelupin.fr/, accessed on 16 July 2025). Eight candidate structural variants in the *LalbFTa1* gene promoter were identified, along with one in the *LalbFTa1* third intron. Based on short-read mapping, three lines were selected for sequence alignment: Amiga (Lalb_Chr02 reference), LD37, and P27174. The 13,417 bp long alignment (spanning promoter, all introns and 3′UTR) further confirmed all variants except LD37 deletion of 46 bp (DOI: 10.5281/zenodo.15224868) (Supplementary Figure S1). The alignment included the Lalb_Chr02 sequence and genome sequence contigs: LD37_contig_002595, containing four deletions, three insertions, and one inversion; P27174-4_contig_014309, carrying one deletion; and P27174-4_contig_000651, similar to the reference Amiga sequence. Besides these indels, 160 SNP loci were identified in the white lupin pangenome (Supplementary Table S4).

Markers from the recently published PCR array [27] (Supplementary Table S5) recognized all indel variants (Supplementary Table S6) except three. These include the following: an 8 bp insertion amplified by a PR_10 marker, with a product size difference too small to provide reliable determination on standard agarose gels; a 59 bp insertion not amplified by the PR_05a/PR_05b marker due to primer binding in a 635 bp deletion; and the previously mentioned 46 bp deletion in LD37. To clarify, a PR_13 marker targeting the 46 bp deletion was scored as polymorphic due to an additional shorter polymorphic product visible on a gel; however, it may represent a repetitive element because the main product was identical in all studied lines. The sequence annotated in the pangenome as the 46 bp deletion is a repetitive element present in eighteen LD37 contigs.

To supplement the PR_05a/b PCR marker, which targeted four structural variants and produced complex patterns of PAVs and length polymorphisms, a pair of new PCR markers (PR_74 and PR_75) was designed to fine-tune the scoring accuracy. Similarly, a 59 bp insertion localized at −5649 bp was supplied with a newly developed PR_76 PCR marker. Agarose gel electrophoregrams showing polymorphism of PCR-based markers targeting white lupin *LalbFTa1* indels are provided in Supplementary Figure S2.

The screening of a white lupin germplasm testing panel, which included 190 genotypes, along with twenty PCR markers that span the entire promoter and third intron of the *LalbFTa1* gene, revealed that ten of the markers were monomorphic and were discarded from further analysis (Table 1, Supplementary Table S7). The remaining ten markers revealed minor allele frequency (MAF) values between 1.1% to 48.9%, with only four below 3% (markers PR_03, PR_04, PR_05b, and QTL11). All polymorphic markers were analyzed across 626 genotypes, yielding similar MAF values to those observed in the germplasm testing panel (Table 1, Supplementary Table S8).

**Figure 1:**
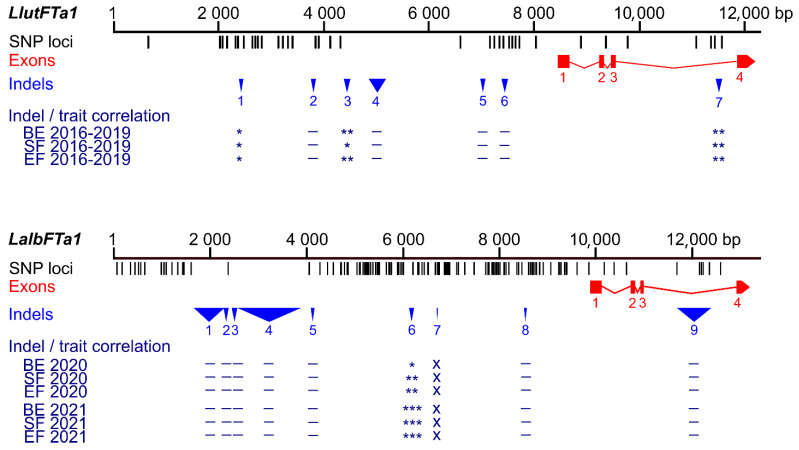
Comparison of sequence polymorphisms identified in the white lupin *LalbFTa1* gene and homologous yellow lupin *LluFTa1* gene and correlations of main indels with plant phenology. Black tags visualize SNP and short (≤5 bp) indel loci, whereas red rectangles and blue triangles show exons and longer (≥6 bp) indels, respectively. The Bonferroni-corrected *p*-value of Spearman’s rank correlation coefficient, calculated for three phenology traits (the number of days to floral bud emergence (BE), start of flowering (SF), and end of flowering (EF)), is shown in the following scheme: ***, *p* < 0.0001; **, 0.0001 ≤ *p* < 0.001; *, 0.001 ≤ *p* ≤ 0.05; –, *p* > 0.05 (not significant); x, not analyzed. White lupin phenotyping was conducted without pre-sowing vernalization during the 2020 and 2021 growing seasons in a greenhouse located at the Institute of Plant Genetics, Polish Academy of Sciences in Poznań. Phenological and sequence data for yellow lupin were retrieved from [37].

Correlation analysis highlighted statistically significant associations with white lupin phenology solely for the PR_09 marker (Supplementary Figure S3, Supplementary Table S9), recognizing a 90 bp deletion located 3572 bp upstream of the transcription start site (TSS) of the *LalbFTa1* gene (r_*ρ*_-value ranging from 0.16 to 0.20, Bonferroni-corrected *p*-value between 2 × 10^−5^ and 0.0013). A shorter indel sequence was associated with delayed flowering. Interestingly, the position of this indel relative to the TSS in white lupin corresponds to the position of the homologous indel in the yellow lupin *LlutFTa1* gene (3848 bp before TSS), which is also significantly associated with plant phenology (Figure 1, Supplementary Table S10) [37]. However, it has the opposite allelic effect, with a shorter indel sequence linked to accelerated flowering.

### 2.3. Twelve Indels Were Identified in the LalbFTa2 Gene Region, but Without Significant Correlation with Plant Phenology

Screening of the white lupin pangenome [45] revealed the presence of twelve potential indels in the *LalbFTa2* gene region, nine localized in the promoter, one in the first intron, and two in the third intron. All indels were confirmed by the 11,669 bp long alignment (DOI: 10.5281/zenodo.15224868) (Supplementary Figure S1) of Lalb_Chr21 and genome sequence contigs from lines Clovis (Clovis_contig_006417 carrying one deletion and one insertion) and GR38 (GR38_contig_001073 containing seven insertions and three deletions). Apart from indels, 213 SNP loci were identified in the white lupin pangenome (Supplementary Table S4).

Published PCR markers [27] (Supplementary Table S5) were able to recognize the first five structural variants at the 5′ sequence of the promoter (Supplementary Table S6). Detection of short indels localized at −2681 bp, −1762 bp, −1603 bp, and +700 bp from the TSS was not feasible with simple agarose-based methodology because of a slight length difference of the PCR products between alleles. For the remaining three unsolved indels, located at positions −1194 bp, +1184 bp, and +1900 bp from the TSS, new PCR markers were designed: PR_77, PR_78, and PR_79 (Supplementary Table S5). Among the twenty markers covering the full promoter and the third intron of the *LalbFTa2* gene, ten were monomorphic in the white lupin germplasm testing panel of 190 genotypes (Supplementary Table S7). Agarose gel electrophoregrams showing the polymorphism of PCR-based markers targeting white lupin *LalbFTa2* gene indels are provided in Supplementary Figure S4.

**Figure 2:**
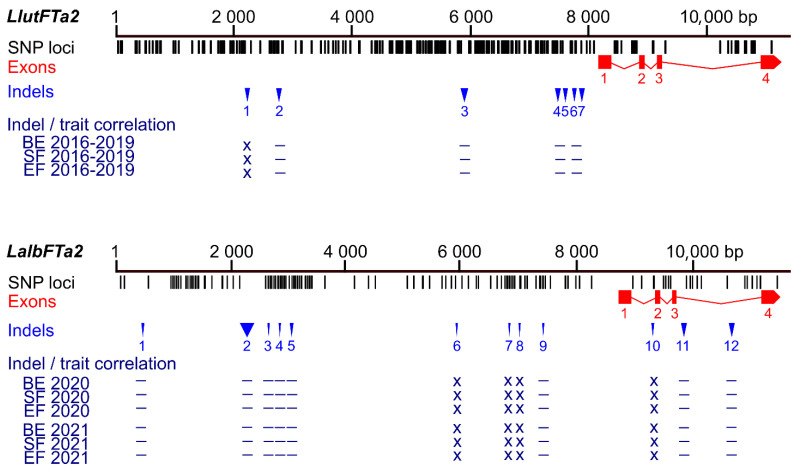
Comparison of sequence polymorphisms identified in the white lupin *LalbFTa2* gene and homologous *L. lutes LluFTa2* gene and correlations of main indels with plant phenology. Black tags visualize SNP and short (≤3 bp) indel loci, whereas red rectangles and blue triangles show exons and large (≥4 bp) indels, respectively. The Bonferroni-corrected *p*-value of Spearman’s rank correlation coefficient, calculated for three phenology traits (the number of days to floral bud emergence (BE), start of flowering (SF), and end of flowering (EF), is shown in the following scheme: ***, *p* < 0.0001; **, 0.0001 ≤ *p* < 0.001; *, 0.001 ≤ *p* ≤ 0.05; –, *p* > 0.05 (not significant); x, not analyzed (correlations for all analyzed *LluFTa2* indel markers were not significant). White lupin phenotyping was conducted without pre-sowing vernalization during the 2020 and 2021 growing seasons in a greenhouse located at the Institute of Plant Genetics, Polish Academy of Sciences in Poznań. Phenological and marker data for yellow lupin were retrieved from [37].

Polymorphic markers revealed MAF values between 1.3% (PR_79) and 14.5% (PR_16) and were tested on the complete array of 626 genotypes (Table 2, Supplementary Table S8). Then, nine markers revealed MAF values in the 0.5–4.8% range, whereas one (PR_16) was significantly more polymorphic (MAF value 13.8%). All analyzed *LalbFTa2* indel markers revealed a lack of significant correlation with the examined traits, with r*_ρ_*-values between −0.04 and 0.13, as well as *p*-values between 0.06 and 1 (Supplementary Figure S5, Supplementary Table S9). Visualization of sequence polymorphisms identified in the white lupin *LalbFTa2* and the homologous yellow lupin *LluFTa2* gene is presented in Figure 2.

### 2.4. Seventeen Indel Variants, Including at Least Twelve That Are Significantly Correlated with Plant Phenology, Were Identified in the LalbFTc1 Gene Region

The presence of seventeen indels (including overlapping variants) in the *LalbFTc1* gene region was revealed by white lupin pangenome alignment [45], sixteen in the promoter, including nine previously reported [27], and one in the third intron. All indels were confirmed by the 14,708 bp long alignment (DOI: 10.5281/zenodo.15224868) (Supplementary Figure S1) of Lalb_Chr14 and genome sequence contigs from lines P27174 (P27174-4_contig_001786 carrying four deletions and one insertion), GR38 (GR38_contig_001107 with one insertion and three deletions), LD37 (LD37_contig 001630 with four deletions and two insertions), and Sanger-sequenced PCR products obtained for P36a/b and PCR42a/b markers (targeting one insertion and three deletions). Besides indels, 251 SNP loci were identified in the white lupin pangenome (Supplementary Table S4). The PCR markers, including those recently published [27] and those developed in this study (Supplementary Table S5), successfully recognized twelve indel variants and three sets of SNPs located in the binding sites of three primers (Supplementary Table S6).

Large indels, such as the P27174 deletion of 2126 bp, the LD37 deletion of 2388 bp, the GR38 insertion of 3800 bp, the P27174 insertion of 3818 bp, and the LD37 insertion of 3877 bp, were supplemented with several markers, designed to have primer bindings sites flanking indels or anchored in particular deletions. A pair of closely located LAP029B deletions of 7 bp and 28 bp was also supplied with specific primers to facilitate agarose gel-based discrimination of particular variants (markers PR_71a-d). A similar approach was used for an LD37 insertion of 24 bp and an adjacent deletion of 75 bp (marker PR_80).

All analyzed primer pair combinations were polymorphic in the white lupin germplasm testing panel of 190 genotypes (Table 3, Supplementary Table S7). Agarose gel electrophoregrams showing the polymorphism of PCR-based markers targeting white lupin *LalbFTc1* gene indels are provided in Supplementary Figure S6. MAF values ranged from 1.1% to 45.3%. A total of 13 primer pairs generated markers with MAF ≥ 10.0. Markers with an MAF value < 1.5 were discarded from complete panel genotyping. Moreover, several polymorphic markers revealed similar segregation patterns, including PR_30 and PR_31 or PR_39, PR_62, PR_67, and PR_70. In these cases, single representative markers (PR_30 and PR_70) were chosen for screening (Supplementary Table S7).

**Figure 3:**
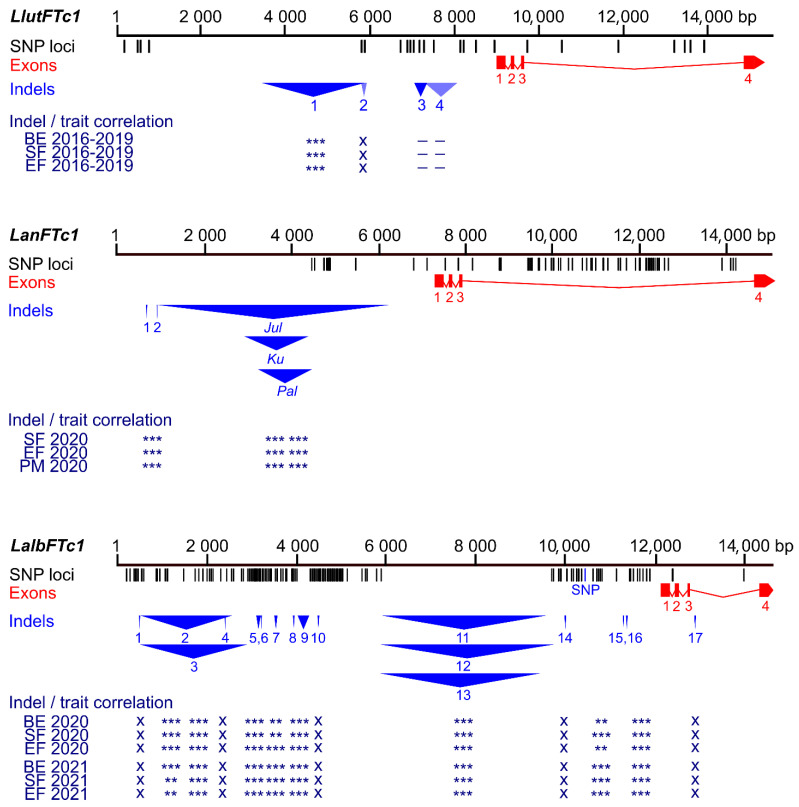
Comparison of sequence polymorphisms identified in the white lupin *LalbFTc1* gene and homologous yellow lupin *LluFTc1* and narrow-leafed lupin *LanFTc1* genes with indicated correlations of main indels with plant phenology. Black tags visualize SNP and short (≤3 bp) indel loci whereas red rectangles and blue triangles show exons and large (≥4 bp) indels, respectively. The Bonferroni-corrected *p*-value of Spearman’s rank correlation coefficient, calculated for phenology traits (the number of days to floral bud emergence (BE), start of flowering (SF), end of flowering (EF), and pod maturity (PM)), is shown in the following scheme: ***, *p* < 0.0001; **, 0.0001 ≤ *p* < 0.001; *, 0.001 ≤ *p* ≤ 0.05; –, *p* > 0.05 (not significant); x, not analyzed. White lupin phenotyping was conducted without pre-sowing vernalization during the 2020 and 2021 growing seasons in a greenhouse located at the Institute of Plant Genetics, Polish Academy of Sciences in Poznań. Phenological and marker data for yellow lupin were retrieved from [37], whereas those for narrow-leafed lupin were retrieved from [39,41], including genotyping performed in this study.

As many as 18 markers revealed a significant correlation with white lupin phenological traits (Supplementary Figure S7, Supplementary Table S9). The highest correlation coefficients were identified for markers PR_71d (from −0.43 to −0.40), PR_60c (from 0.31 to 0.35), PR_58c (from −0.34 to −0.31), PR_71a (−0.32), PR_36b (from −0.33 to −0.31), PR_60b (from 0.30 to 0.33), PR_42a (from −0.34 to −0.28), PR_80 (from 0.30 to 0.32), and PR_70 (from −0.31 to −0.28). These markers target LAP029B deletions of 7 bp and 28 bp, LD37 deletion of 2388 bp, LAP022E insertion of 25 bp, LAP022E deletion of 264 bp, LD37 deletion of 70 bp, and LD37 insertion of 18 bp. It is noteworthy that the LD37 deletion of 2388 bp is located at a corresponding position relative to the transcription start site, analogous to the *Ku*, *Jul*, and *Pal* indels in the *LanFTc1* promoter that facilitate vernalization-independent early flowering in narrow-leafed lupin, as well as a major indel in the *LlutFTc1* promoter, which is associated with vernalization independence in yellow lupin (Figure 3, Supplementary Tables S10 and S11) [27,37,38,41].

The presence of LAP022E and LAP029B indels was evidenced by sequencing of PCR products obtained for markers PR_36a/b and PR42a/b, whereas the presence of two closely located LD37 indels was shown by sequencing of PR_80 PCR products (DOI 10.5281/zenodo.15223455). Taking into consideration the geographic distribution of analyzed white lupin accessions, two highly associated alleles represented by markers PR_58c and PR_36b were identified predominantly in the Azores, the Canaries, and Greece (Supplementary Tables S1 and S8).

### 2.5. No Indel Was Found in the LalbFTc2 Gene Promoter, While Four Indels Were Identified in the Introns, Albeit Without Significant Correlation with Phenology

White lupin pangenome alignment [45] revealed no indel in the *LalbFTc2* gene promoter. Nevertheless, it highlighted the presence of four indels located between particular exons: one in the second intron and three in the third intron (Supplementary Table S6). The first one, identified in the white lupin line GRC5262B, was visualized by short sequence reads and a GRC5262B_contig_007970 misassembly at approximate positions spanning a length of 209 bp in the reference genome. The region was also marked by the gap between the ends of Dieta_contig_007291 and Dieta_contig_008195, as well as between P27174_contig_011400 and P27174_contig_003576 in the 13,133 bp long alignment (Supplementary Figure S1), including the Lalb_Chr09 sequence (DOI 10.5281/zenodo.15224868). Apart from these indels, 389 SNP loci were identified in the pangenome (Supplementary Table S4).

The screening of the white lupin germplasm testing panel of 190 genotypes using the PR_64 marker targeting the GRC5262B missassembly resulted in a presence/absence variant, suggesting that a large insertion influences PCR amplification. Three other indels (13 bp, 1463 bp, and 8 bp) were localized in the third intron and were identified in alignment with Dieta_contig_007291. The marker PR_73 flanking these indels confirmed the expected PCR product length polymorphism. Apart from the indels, PCR screening with *LalbFTc2* gene promoter primers (Supplementary Table S5) revealed one presence/absence variant (marker PR_57), indicating a novel sequence polymorphism absent in the pangenome alignment (Supplementary Tables S6 and S7). Nevertheless, it was rare, identified only in a few white lupin accessions (Table 4). Agarose gel electrophoregrams showing the polymorphism of PCR-based markers targeting white lupin *LalbFTc*2 gene indels are provided in Supplementary Figure S8.

All polymorphic PCR markers (PR_57, PR_64, PR_73) were analyzed in the complete white lupin germplasm panel, revealing MAF values of 0.9%, 2.2%, and 17.4%, respectively (Supplementary Table S8). Although the location of a third *LalbFTc2* indel corresponds to the *LlutFTc2* indel, which is associated with photoperiodic responsiveness in yellow lupin [37], the correlation of the corresponding PR_73 marker with white lupin phenology was negligible, with r*_ρ_*-values of 0.09–0.12 and non-significant *p*-values (Supplementary Figure S9, Supplementary Table S8).

**Figure 4:**
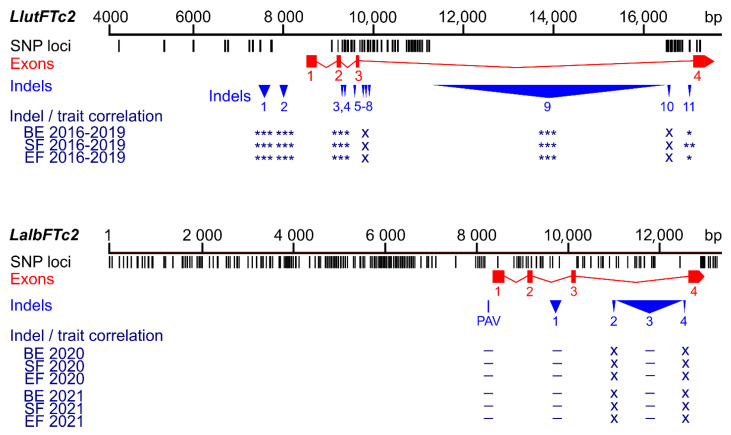
Comparison of sequence polymorphisms identified in the white lupin *LalbFTc2* gene and homologous yellow lupin *LluFTc2* gene and correlations of main indels and a presence/absence variant (PAV) with plant phenology. Black tags visualize SNP and short (≤5 bp) indel loci, whereas red rectangles and blue triangles show exons and large (≥6 bp) indels, respectively. *p*-value of Spearman’s rank correlation coefficient with Bonferroni correction, calculated for three phenology traits (the number of days to floral bud emergence (BE), start of flowering (SF), and end of flowering (EF)), is shown in the following scheme: ***, *p* < 0.0001; **, 0.0001 ≤ *p* < 0.001; *, 0.001 ≤ *p* ≤ 0.05; –, *p* > 0.05 (not significant); x, not analyzed. White lupin phenotyping was conducted without pre-sowing vernalization during the 2020 and 2021 growing seasons in a greenhouse located at the Institute of Plant Genetics, Polish Academy of Sciences in Poznań. Phenological and marker data for yellow lupin were retrieved from [37].

Non-significant correlations were also found in PR_64 and PR_57 markers, representing the first indel and the presence/absence variant, respectively. Interestingly, the PR_57 marker turned out to be highly selective towards winter-type cultivar Aster (represented by lines LAP119c and LAP119d), with only three false-positive homozygotes and one heterozygote in the complete array of 626 genotypes (99.4% accuracy). A comparison of sequence polymorphisms identified in the white lupin *LalbFTc2* gene and the homologous yellow lupin *LluFTc2* gene and the correlations of main indels with plant phenology is presented in Figure 4.

### 2.6. LalbFTc1 Promoter Indels Carry Potential Binding Sites for Transcription Factors Involved in Flowering Control

**Figure 5:**
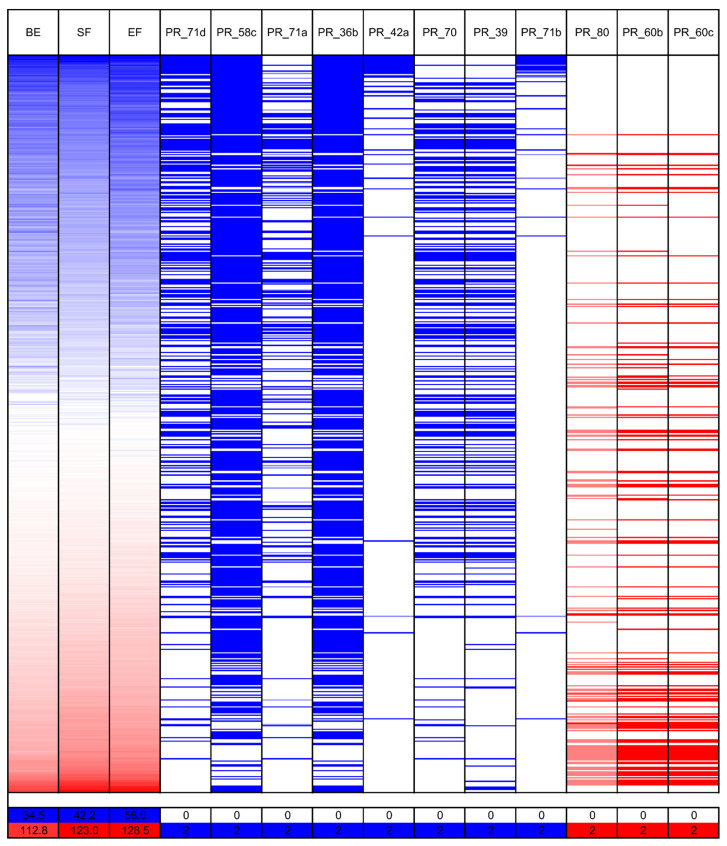
Allelic composition heatmap of PCR markers that revealed the highest correlations with white lupin phenology: (the number of days to floral bud emergence (BE), start of flowering (SF), and end of flowering (EF)). The bar below the heatmap indicates the color legend of phenological observations and PCR marker alleles recorded for 626 white lupin genotypes. White lupin phenotyping was conducted without pre-sowing vernalization during the 2020 and 2021 growing seasons in a greenhouse located at the Institute of Plant Genetics, Polish Academy of Sciences in Poznań.

As *LalbFTc1* indels revealed the most significant correlations with white lupin phenology (Figure 5), in silico analysis of transcription factor binding sites was performed. An alignment of 14,708 bp carrying all indels recognized in the *LalbFTc1* gene region (DOI 10.5281/zenodo.15224868) was submitted to the PlantPAN for annotation of transcription factor binding sites targeting the region upstream of the *LalbFTc1* TSS (located at 12,009 bp in the alignment). This analysis revealed that many transcription factors (115) have all their *LalbFTc1* promoter binding sites localized exclusively in polymorphic regions (indels or SNPs) (Supplementary Table S12). This set included 18 elements from flowering regulatory pathways, which had potential binding sites, sometimes even in two or more indels. Thus, the overlapping section of 2126 and 2388 indels revealed binding sites for PHYA-INDUCED MOTIF (SORLIP2AT), ABSCISIC ACID RESPONSIVE ELEMENT-BINDING FACTOR 1 or 4 (ABF1 or ABF4), FLOWERING BHLH 4 (FBH4), GATA TRANSCRIPTION FACTOR 25 (GATA25), AGAMOUS-LIKE 15 (AGL15), and INDETERMINATE(ID)-DOMAIN 7 (IDD7), while the remaining part of the 2388 indel contained another AGL15 site. A 264 bp deletion contained an additional IDD7 site, whereas the largest 3800–3877 bp indels included additional binding sites for SORLIP2AT, GATA25, and AGL15, as well as unique loci for DWARF AND DELAYED FLOWERING 2 (DDF2), NAC DOMAIN CONTAINING PROTEIN 50 (NAC050), ELONGATED HYPOCOTYL 5 (HY5), AGAMOUS-LIKE 71 (AGL71), and TGA4/OCTOPINE SYNTHASE (OCS)-ELEMENT-BINDING FACTOR 4 (OBF4). Moreover, LAP022E carried binding sites of MYB30 and MYB73 at SNP loci. The list of transcription factors from flowering regulatory pathways that revealed *LalbFTc1* binding sites exclusively in the polymorphic regions of the promoter is provided in Table 5, whereas their putative roles are presented in the Discussion Section.

## 3. Discussion

### 3.1. Conserved Function of FTc1 Promoter Indels in Domesticated Old World Lupin Species

In the present study, significant correlations were identified between the number of days from sowing to reaching a specific growth phase (bud emergence, start of flowering, and end of flowering) and indel polymorphism in the regulatory regions of the *LalbFTc1* gene and, to a lesser extent, the *LalbFTa1* gene. Interestingly, the positions of partially overlapping *LalbFTc1* indels of 2126 bp and 2388 bp associated with early flowering of white lupin correspond to the positions of partially overlapping *LanFTc1* indels of 1423 bp, 5162 bp, and 1208 bp underlying *Ku*, *Jul*, and *Pal*, conferring early flowering of narrow-leafed lupin, as well as the *LlutFTc1* indel of 2227 bp associated with early flowering of yellow lupin [27,37,38,41,44]. Additionally, the position of a *LalbFTa1* indel of 90 bp significantly correlated with flowering time in white lupin and converged with the localization of the *LlutFTa1* indel of 58 bp, which is significantly associated with plant phenology in yellow lupin [37]. However, the other two white lupin *LalbFTa1* indels, which are localized at similar positions as the two significantly associated *LlutFTa1* indels, did not show such associations in the analyzed panel of 626 white lupin genotypes. One of these indels, represented by a QTL11 marker, is located in the third intron of the *LalbFTa1* gene. This indel is perfectly co-segregated with one of just a few major QTLs for flowering time in a mapping population derived from a cross between domesticated early-flowering Kiev Mutant and late-flowering Ethiopian landrace P27174 [10,35]. In the current study, the Ethiopian *LalbFTa1* allele was found in nine accessions at the homozygous phase and six as heterozygotes. Homozygous lines included two Ethiopian landraces, one Italian landrace (LAP098a), and one Polish breeding line (95441, Wat × *L. graecus* Boiss.). Three of these lines were early-flowering, whereas the remaining ones revealed late flowering time, like the P27174 landrace. The breakdown of the genotype–phenotype association for this locus in two lines (95133 and 95441) carrying the late QTL11 allele in a homozygous state may result from the presence, in the genomes of these lines, of several *LalbFTc1* indels related to earliness, evidenced by the corresponding PR_36b, PR_39, PR_41, PR_42b, PR_58c, PR_60a, PR_60b, PR_60c, PR_70, PR_71a, PR_71b, PR_71c, and PR_71d marker scores. Haplotype analysis of *LalbFTc1* promoter indels could improve the correlation study; however, the white lupin genome was recently revealed to have very rapid LD decay [11,27,46,47], and we observed this phenomenon also around *FT* promoters in our germplasm collection. Therefore, we decided to focus on particular indels rather than on co-segregating markers. Some level of recombination occurred even between two closely located markers, separated by just a few hundred base pairs. Our recent white lupin GWAS highlighted significant association of *LalbFTc1* indels with plant phenology and vernalization responsiveness; however, that study was focused on non-domesticated germplasm [27]. In the present study, we supplemented a large panel of landraces with a substantial number of materials representing more than 40 years of white lupin breeding programs in Europe. As this analysis does not control for kinship or relatedness at other genetic loci, it cannot be ruled out that the true genetic basis for the observed phenological variation is conferred by other correlated genetic markers. This issue may be resolved by genome complexity reduction-based sequencing of developed genetic diversity panel and association analysis including both PCR-based and genome-wide markers. Such an approach is currently being implemented.

Interestingly, the flowering time in the narrow-leafed lupin is controlled by a single major gene (*LanFTc1*) with very few minor QTLs. In contrast, in white and yellow lupins, this trait showed significant quantitative variation, suggesting an involvement of several genes from flowering regulatory pathways [27,28,41,43,48,49,50].

### 3.2. AGL15 Is the Most Likely the Main Transcription Factor in Maintaining the Late-Flowering Phenotype of White Lupin

The common indel-specific transcription factor identified for *FTc1* homologs in three domesticated Old World lupin species is AGL15 [37,41]. AGL15 is a known floral repressor that binds directly to the *FT* gene promoter at sites partially overlapping with those bound by the floral repressors SHORT VEGETATIVE PHASE (SVP) and FLOWERING LOCUS C (FLC) [51,52,53,54,55]. Moreover, AGL15 is also involved in epigenetic silencing of *FT* chromatin to prevent precocious flowering in response to long days in *Arabidopsis* [56]. The white lupin genome, like many other legumes, lacks the FLC gene [28,35,57]; however, it putatively contains AGL15 homologs, as BLAST 2.16.0 alignment of the *Glycine max AGL15* coding sequence (AY370659.1) to the white lupin CDS database [58] matched three loci (Lalb_Chr19g0140231, Lalb_Chr01g0000501, and Lalb_Chr04g0263791) with high sequence identity (about 75%) and significant e-values (between 5.2 × 10^−128^ and 1.9 × 10^−133^). Therefore, it is possible that AGL15 is a major *FT* repressor in white lupin, and the elimination of all AGL15 binding sites from the *LalbFTc1* promoter releases this gene from negative control and triggers flowering.

Apart from AGL15, a few other candidate transcription factors were highlighted, including ABF1, DDF2, FBH4, NAC050, GATA25, HY5, AGL71, OBF4, IDD7, MYB30, and MYB73. ABF1 is a drought-inducible transcription factor that delays flowering in rice [59]. DDF2 is closely related to DDF1, which confers a dwarf and late-flowering phenotype in *Arabidopsis* [60]. FBH4 accelerates *Arabidopsis* flowering in low-nitrogen conditions by transcriptional activation of the direct target *CONSTANS* (*CO*) and downstream florigen (*FT*) genes [61,62]. NAC050 is involved in the repression of floral integrator genes, including *FT*, providing a late-flowering phenotype of *Arabidopsis* [63,64]. GATA25 acts as an expression activator and accelerates the flowering time of *Arabidopsis,* directly binding to the promoters of several flowering pathway genes, including *FT* [65]. HY5 acts as an epigenetic repressor of flowering time in *Arabidopsis* [66]. Moreover, overexpression of poplar HY5 in *Arabidopsis* also results in prolonged vegetative growth and delayed flowering [67]. AGL71 promotes flowering through a gibberellin-dependent pathway [68] and OBF4 interacts with CO and binds to the *FT* promoter [69], whereas IDD7 is responsible for the inhibition of multiple traits, including plant growth and development, including the transition from the vegetative to generative phase [70]. MYB30 is a CO-independent *FT* activator accelerating flowering irrespective of the length of photoperiod [71], whereas MYB73 is an *FT* activator negatively targeted by HETEROCHROMATIN PROTEIN 1 (LHP1) [72]. Taking into consideration the direction of phenotypic effects of indel allelic phases and transcription factors, the best repressors for 2126 bp and 2388 bp deletions, which accelerate flowering, are AGL15 and IDD7. In contrast, for 3800–3877 bp insertions that delay flowering, AGL15, DDF2, and HY5 are the best repressors.

### 3.3. Novel Perspectives for White Lupin Breeding Towards Spring Sowing in a Changing Climate

The present study highlighted the vast diversity of white lupin germplasm in plant phenology, revealing the presence of numerous very early, vernalization-independent landraces, predominantly from Turkey and Syria, which flowered earlier than current cultivars and breeding lines developed for spring-sown cultivation in Central and Eastern Europe. This finding is coherent with previous reports on the earliness of Turkish white lupin germplasm [28,73]. Indeed, population structure analysis based on DArT-seq markers revealed that very early Turkish, Jordanian, and Syrian landraces are genetically distinct from other potential donors of earliness from Egypt, Kenya, Israel, and Sudan, as well as from French spring cultivars [27]. It would be highly advantageous for white lupin breeders to have several different genetic sources of essential traits, as these will help to avoid the critical domestication bottleneck effect. Such a bottleneck unintentionally occurred in another Old World lupin species, narrow-leafed lupin, when only single donors of major traits were available during major domestication efforts targeting earliness, sweetness, seed coat permeability, and anthracnose resistance [74,75,76,77]. Furthermore, white lupin breeders have already reported substantial challenges in incorporating a key agronomic trait, specifically anthracnose resistance, into standard (Ukrainian) sources of early flowering [8,33,78]. The presence of diverse genetic donors of earliness offers greater flexibility in selecting components for crossbreeding. There is a range of heritable traits that could be transferred between particular genotypes, including, besides those mentioned, anthracnose resistance [11,12,50,79,80,81,82], drought tolerance [46,83], calcareous soil adaptation [47,84,85,86], low alkaloid content [15,87,88,89,90], and resistance to *Diaporthe toxica* and *Pleiochaeta setosa* causing Phomopsis blight and Pleiochaeta root rot diseases [91,92], as well as yield-related traits, such as pod length, number of seeds in a pod, thousand grain weight, and number of seeds per plant [93,94,95]. Numerous white lupin genotypes in the present study exhibited intermediate phenology, representing a wide range of flowering dates. They constitute valuable resources for addressing ongoing climate change by producing adapted cultivars exploiting growing seasons extended in both directions, with earlier sowing and later maturity [96]. Current climate change (global warming) has already resulted in significant advancement of spring phenology and the onset of flowering of plants, reaching a few days per 1 °C of spring warming [97,98,99]. Nevertheless, the patterns are not uniform across species and regions (including even delay of flowering in some cases) and exhibit significant latitudinal gradients [97,100]. Earlier spring sowing phenology seems to be beneficial in the context of yield under the future climate. In contrast, maturity time requires adaptation to the water regime, contrasting regions with water limitations (earlier maturity to avoid terminal water stress), and regions without significant water shortage during grain filling in summer (later maturity to exploit the full range of available growing season) [96]. European farmers already responded to ongoing climate changes by advancing spring activities as a form of agronomic adaptation to observed trends [101]. White lupin breeders should follow them by releasing thermoneutral, anthracnose-resistant spring cultivars with increased drought tolerance and phenology adapted to the lengths of current and future growing seasons at target cultivation sites.

## 4. Materials and Methods

### 4.1. White Lupin Germplasm

The germplasm panel analyzed in this study consisted of 626 genotypes: 313 accessions provided by Poznań Plant Breeding Ltd. (Wiatrowo, Poland) and 313 genotypes selected from 120 accessions based on their phenological diversity within accessions [102] that were provided by the Council for Agricultural Research and Economics (Lodi, Italy). The list of genotypes, including countries/regions of origin, domestication status, and germplasm donors, is provided in Supplementary Table S1. Genotypes originate from 31 countries or territories (i.e., Canaries, Madeira, and Azores) and differ by domestication status: 386 are landraces, 135 wild or primitive accessions, 74 cultivars, 28 breeding lines, and 3 mutants. Domesticated material was selected from major white lupin cultivation countries such as Poland (27 lines), France (22), Germany (13), Ukraine (6), Belarus (6), Russia (5), South Africa (5), Chile (4), Spain (4), Portugal (3), and others, located primarily in the temperate subcontinental climate zone. Landrace collection sites represent a wide range of climates, including tropical and subtropical (Ethiopia), cold semi-arid (Anatolia and Maghreb), dry-summer Mediterranean (sites around the Mediterranean Basin), warm-summer Mediterranean (the Azores and Madeira), and humid temperate (i.e., oceanic, France and Portugal). These regions also diverged in the length of the photoperiod during the white lupin growing season, ranging from approximately 9–10 h in winter sowing in the northern areas of the Mediterranean Basin to 11–12 h in Ethiopia and 12–17 h in spring sowing in other regions of Europe.

### 4.2. Phenotyping of White Lupin Growth Transition into the Generative Phase

Experiments were conducted without pre-sowing vernalization in a greenhouse located at the Institute of Plant Genetics, Polish Academy of Sciences, Poznań, Poland (52°26′ N 16°54′ E), with an air temperature above 18 °C. Seeds were sown by the end of winter (19 March 2020 and 11 March 2021), and plants were grown under an ambient long-day photoperiod, increasing during plant growth from about 12 h in March to greater than 16 h in June and July. Phenology traits were phenotyped as the number of days from sowing to reaching a specific developmental stage. Floral bud emergence was inspected every second day. The start of flowering was recorded when the first fully colored petals on the main stem appeared, and the end of flowering was registered when half of the petals on the primary inflorescence had faded. All observations were made with a minimum of three and a maximum of ten biological replicates. The experimental design (positions of pots in the greenhouse) is provided in Supplementary Table S2.

### 4.3. DNA Isolation from the White Lupin Germplasm Panel

Two medium-sized young leaves (about 100–150 mg tissue in total) were collected from 5-week-old plants into 2 mL microcentrifuge tubes (Eppendorf, Hamburg, Germany), immediately frozen in liquid nitrogen and stored in an ultra-low temperature freezer at −70 °C. Homogenization of frozen tissue was performed with two stainless steel beads (ø 5 mm, Qiagen, Hilden, Germany) using TissueLyser II (Qiagen) at 30 rpm for 30 s. The automated isolation system Maxwell^®^ RSC 48 Instrument (Promega, Mannheim, Germany) and the Maxwell^®^ RSC PureFood GMO and Authentication Kit (Promega) [103] were used for DNA isolation without any changes to the standard protocol. DNA concentration and quality (Supplementary Table S3) were measured using a NanoDrop 2000 (ThermoFisher Scientific, Warsaw, Poland). DNA was isolated from 3 plants per genotype separately and subsequently mixed in equal amounts of DNA to provide a final 100 ng/µL concentration (this approach enabled us to detect possible heterogeneity within genotypes).

### 4.4. PCR-Based Genotyping of Indel Polymorphism in the FT Gene Promoters

PCR-based markers were anchored in the regulatory regions of four *FT* homologs: *LalbFTa1* (*Lalb_Chr02g0156991*), *LalbFTa2* (*Lalb_Chr21g0317021*), *LalbFTc1* (*Lalb_Chr14g0364281*), and *LalbFTc2* (*Lalb_Chr09g0331851*), spanning by overlapping products the region from 8 kbp upstream of the transcription start site to the first exon of the analyzed genes [27]. Moreover, based on the sequence alignment of the white lupin pangenome sequence [45] and results of preliminary genotyping [27], three additional PCR-based markers (PR_74, PR_75, and PR_76) were designed for *LalbFTa1* indels, three (PR_77, PR_78, and PR_79) for *LalbFTa2* indels, five (PR_60, PR_62, PR_66, PR_67, and PR_80) for *LalbFTc1* indels, and two (PR_64 and PR_73) for *LalbFTc2* indels. Sequence alignment was performed using the progressive Mauve algorithm [104], assuming genome collinearity, whereas PCR primers were designed using Primer 3 Plus [105]. Both programs were run in Geneious Prime 2025.0.3 [106]. The list of primer sequences and corresponding white lupin genome coordinates is provided in Supplementary Table S5.

GoTaq G2 Flexi DNA Polymerase (Promega) was used to amplify PCR products. The PCR protocol included initial denaturation (94 °C, 3 min) and 35 cycles composed of three phases—denaturation (94 °C, 30 s), annealing (56–62 °C, 30 s), and elongation (72 °C, 30 s up to 1 kbp, 60 s for longer products)—followed by the final elongation (72 °C, 5 min). The visualization of length differences between alleles was conducted using agarose gel electrophoresis, with the agarose concentration (1–3%) inversely proportional to the size of the expected products. For small indels (shorter than about 50 bp), high-resolution 3:1 agarose (Serva, Heidelberg, Germany) was used, whereas for larger indels, wide-range agarose (Serva) was preferred. Moreover, for comparison of *FTc1* allelic effects between lupin species, narrow-leafed lupin germplasm [39] was genotyped with *LanFTc1* indel markers [43]. Sanger sequencing of selected PCR products was performed by Genomed (Warsaw, Poland).

### 4.5. Correlation Analysis

A Kolmogorov–Smirnov test was performed to check the normality of phenotypic data [107]. This test revealed that the datasets for all analyzed phenological traits in both years significantly diverged from a normal distribution. Therefore, a calculation of the Spearman rank correlation was performed. The ranks for phenotypic observations were determined by using the formula =RANK.AVG in Microsoft Excel. Correlations were calculated between rank values and marker scores (0, 1, 2).

To determine if the obtained correlation coefficient was statistically significant, a two-tailed *t*-test was performed with Bonferroni correction applied by multiplying each calculated *p*-value by the number of tested markers. To compare specific allelic effects between species, correlation calculations were also conducted for narrow-leafed lupin *LanFTc1* indels using phenotypic observations for 126 accessions reported in field studies [39]. This was performed alongside analysis of yellow lupin *LlutFTa1*, *LlutFTa2*, *LlutFTc1*, and *LlutFTc2* indels using controlled-environment phenotypic observations and genotypic data published for 111 accessions [37].

### 4.6. Annotation of Transcription Factor Binding Sites in LalbFTc1 Promoter

To investigate whether the observed sequence polymorphism in the *LalbFTc1* promoter may be associated with the presence or absence of binding sites for specific transcription factors from flowering regulatory pathways, we constructed sequence alignments using the reference Amiga sequence and contigs LD37_contig_001630, P27174-4_contig_001786, and GR38_contig_001107 between the PRFTC1F1 primer and the *LalbFTc1* transcription start site. Additionally, we sequenced PR_42 PCR products for lines LAP029B and LAP030A between PRFTC1F5 and PRFTc1_R5b primers and sequenced PR_36 PCR products for lines LAP022B and LAP022E between PRFTC1F3 and PRFTc1_R3b primers. Sequences were extracted from the alignments, with gaps representing indel polymorphism and submitted to the Plant Promoter Analysis Navigator (PlantPAN) [108,109] using the *Arabidopsis thaliana* transcription factors database and by applying a similarity threshold of 0.85. The identified transcription factors were annotated using the genes that control flowering time in *Arabidopsis* [110]. Additionally, allele-specific transcription factors with potential binding sites identified solely in the polymorphic loci were screened in the literature database for their potential involvement in flowering time regulation.

## 5. Conclusions

The present study evidenced a high structural variation in promoter sequences of three *FT* homologs and introns of all four *FT* genes encoded by the white lupin genome. More than three hundred allelic combinations of *FT* indels were found in the white lupin germplasm panel by PCR screening, of which one-third could be attributed to the *LalbFTc1* gene. The *LalbFTc1* promoter was evidenced to carry not only the highest number of indels among all white lupin *FT* homologs but also the largest ones, up to 3877 bp in length. Moreover, at least twelve *LalbFTc1* promoter indels demonstrated significant correlations with plant phenology, and some also showed high discriminatory power towards earliness. A narrow list of indel-specific transcription factors was annotated for the *LalbFTc1* promoter, highlighting AGL15 as the most plausible candidate. *LalbFTc1* indel-based elimination of all binding sites for important repressive transcription factor(s) from flowering regulatory pathways seems to be a conserved mechanism in Old World lupins, as it was shown to be present in three distinct lineages (white lupin, narrow-leafed lupin, and yellow lupin). Besides contributing to the general knowledge of indel polymorphisms around *FT* genes and their relations with plant phenology, our study provided support for the ongoing domestication of white lupin as a crop, highlighting key structural mutations that breeders can target to align white lupin growing season requirements with the characteristics of the growing periods experienced in a given location.

## Figures and Tables

**Table 1:** PCR validation of indel polymorphism for the *LalbFTa1* (*Lalb_Chr02g0156991*) gene and minor allele frequency (MAF) values in the white lupin germplasm testing panel (190 genotypes) and full germplasm array (626 genotypes).

Marker Name	Max Distance to TSS (bp) ^1^	Min Distance to TSS (bp) ^1^	Score “0”	Score “1”	Score “2”	MAF 190	MAF 626
PR_01	−8280	−7770	511 bp	-	-	0.0	-
PR_02	−7874	−7160	715 bp	-	-	0.0	-
PR_03	−7327	−6626	702 bp	-	no product	1.1	0.5
PR_04	−6980	−6109	872 bp	-	no product	1.1	0.8
PR_05a	−6203	−5430	774 bp or no product	-	~850 bp or 2207 bp	5.3	5.4
PR_05b	−6203	−5430	774 or ~850 bp or 2207 bp	-	no product	1.6	0.5
PR_06	−5519	−4763	757 bp	-	-	0.0	-
PR_07	−4848	−4401	448 bp	-	-	0.0	-
PR_08	−4848	−4079	770 bp	-	-	0.0	-
PR_09	−4161	−3336	826 bp	heterozygote	736 bp	28.2	22.5
PR_10	−3526	−2733	794 bp	-	-	0.0	-
PR_11	−2800	−2128	673 bp	-	-	0.0	-
PR_12	−2258	−1509	750 bp	-	-	0.0	-
PR_13	−1725	−1096	630 bp	-	~401 bp	48.9	43.0
PR_14	−1241	−442	800 bp	-	-	0.0	-
PR_15	−511	229	741 bp	-	-	0.0	-
QTL11	3390	1169	2218 bp	heterozygote	1535 bp ^2^	2.1	1.9
PR_74	−6980	−6029	951 bp	heterozygote	380 bp	2.4	1.7
PR_75	−6057	−5739	319 bp	heterozygote	1523 bp	2.9	1.4
PR_76	−5732	−5430	303 bp	heterozygote	362 bp	7.9	6.9

^1^ position relative to the transcription start site of the *LalbFTa1* gene on chromosome Lalb_Chr02 at locus 14,996,788 bp, direction “forward”. ^2^ deletion in the P27174 line verified by Sanger sequencing [35] to have 683 bp instead of 687 bp present in the pangenome sequence alignment.

**Table 2:** PCR validation of indel polymorphism in the *LalbFTa2* (*Lalb_Chr21g0317021*) gene promoter and minor allele frequency (MAF) values in the white lupin germplasm testing panel (190 genotypes) and full germplasm array (626 genotypes).

Marker Name	Max Distance to TSS (bp) ^1^	Min Distance to TSS (bp) ^1^	Score “0”	Score “1”	Score “2”	MAF 190	MAF 626
PR_16	−7866	−7163	704 bp	heteorzygote	676 bp	14.5	13.8
PR_17	−7287	−6615	673 bp	-	-	0.0	-
PR_18a	−6826	−6120	707 bp or heterozygote	-	950 bp	4.2	2.4
PR_18b	−6826	−6120	950 bp or heterozygote	-	707 bp	6.8	3.2
PR_19a	−6214	−5515	700 bp or heterozygote	-	927 bp or no product	4.2	2.4
PR_19b	−6214	−5515	927 bp or heterozygote or no product	-	700 bp	6.3	3.2
PR_20	−5626	−4914	713 bp	heterozygote	661 bp	5.8	3.2
PR_21	−5014	−4345	700 bp	-	-	0.0	-
PR_22	−4626	−3893	734 bp	-	-	0.0	-
PR_23	−3965	−3266	700 bp	-	-	0.0	-
PR_24	−3383	−2783	601 bp	-	-	0.0	-
PR_25	−2880	−2101	780 bp	-	no product	0.0	-
PR_26	−2182	−1409	774 bp	-	-	0.0	-
PR_27	−1691	−1137	555 bp	-	-	0.0	-
PR_28	−1279	−428	852 bp	-	-	0.0	-
PR_29	−564	275	840 bp	-	-	0.0	-
PR_77a	−1279	−1137	157 bp and/or “longer”	-	143 bp	6.8	3.4
PR_77b	−1279	−1137	157 bp	-	143 bp and/or “longer”	7.4	4.8
PR_78	1335	1050	286 bp	-	360 bp	3.7	2.2
PR_79	2074	1854	249 bp	-	271 bp	1.3	0.5

^1^ position relative to the transcription start site of the *LalbFTa2* gene localized on chromosome Lalb_Chr21 at locus 12,758,313 bp, reverse-complement.

**Table 3:** PCR validation of indel polymorphism in the *LalbFTc1* (*Lalb_Chr14g0364281*) gene promoter [27] and minor allele frequency (MAF) values in the white lupin germplasm testing panel (190 genotypes) and full germplasm array (626 genotypes).

Marker Name ^1^	Min Distance to TSS (bp) ^2^	Max Distance to TSS (bp)	Score “0”	Score “1”	Score “2”	MAF 190	MAF 626
PR_30	−8065	−7254	812 bp	-	no product	6.8	9.1
PR_58a	−8065	−5562	2504 bp	heterozygote	378 bp	7.1	12.5
PR_58b	−8065	−5562	2504 or 378 bp	-	no product or 116 bp	7.9	5.6
PR_58c	−8065	−5562	2504 or 378 bp or no product	-	116 bp	22.6	20.0
PR_60a	−8065	−4899	no product	-	1041 bp, 779 bp, or heterozygote	33.2	36.4
PR_60b	−8065	−4899	no product or 1041 bp	-	779 bp or heterozygote	24.7	20.6
PR_60c	−8065	−4899	no product or 1041 bp or heterozygote	-	779 bp	24.7	19.3
PR_31	−7312	−6553	760 bp	-	no product	6.8	-
PR_66	−7312	−4320	2993 bp	-	no product	11.1	-
PR_33	−6242	−5562	681 bp	-	no product	10.0	-
PR_34	−5694	−4899	796 bp	-	no product	4.2	-
PR_80	−5202	−4944	259 bp	-	204 bp	12.4	10.9
PR_35a	−4968	−4320	649 bp or no product	heterozygote	625 bp	3.7	2.6
PR_35b	−4968	−4320	649 bp	-	625 bp, heterozygote or no product	6.3	5.6
PR_61	−4968	−3254	1715 bp	-	no product	4.2	-
PR_36a	−4585	−3860	726 bp or heterozygote	-	482 bp or no product	1.6	2.2
PR_36b	−4585	−3860	482 bp or heterozygote	-	726 bp or no product	22.1	20.1
PR_37	−3965	−3254	712 bp	-	no product	1.6	-
PR_38	−3403	−2695	709 bp	-	no product	1.6	-
PR_67	−3403	−451	2953 bp	-	no product	31.1	-
PR_39	−2908	−2262	no product	-	647 bp	32.1	35.3
PR_62	−2908	−1179	1730 bp or no product	-	647 bp	30.5	-
PR_40	−2365	−1591	775 bp	-	no product	1.1	-
PR_41	−1944	−1179	no product	-	766 bp	37.4	40.3
PR_42a	−1259	−451	802 or 809 bp	-	774 or 781 bp	12.6	6.0
PR_42b	−1259	−451	other variants	-	~850 bp	10.5	19.6
PR_71a	−970	−714	222 or 229 or 250 bp	heterozygote	257 bp	27.1	25.2
PR_71b	−970	−714	250 or 257 bp	heterozygote	222 or 229 bp	11.3	4.6
PR_71c	−970	−714	other variants	-	~280 bp	17.9	22.7
PR_71d	−970	−714	250 bp	-	other variants	45.3	41.2
PR_70	−875	−600	no product	-	276 bp	32.6	35.5
PR_43	−560	140	701 bp	-	-	0.0	-

^1^ marker PR_32 provided unspecific product, marker PR_40 recognized rare (MAF 1%) unknown polymorphism (presence/absence variant not found in the pangenome alignment), and marker PR_43 did not target any mutation in the alignment and was monomorphic. ^2^ position relative to the transcription start site of the *LalbFTc1* gene localized on chromosome Lalb_Chr14 at locus 5,850,617 bp, direction “forward”.

**Table 4:** PCR validation of indel polymorphism in the *LalbFTc2* (*Lalb_Chr09g0331851*) gene promoter and minor allele frequency (MAF) values in the white lupin germplasm testing panel (190 genotypes) and full germplasm array (626 genotypes).

Marker Name	Min Distance to TSS (bp) ^1^	Max Distance to TSS (bp) ^1^	Score “0”	Score “1”	Score “2”	MAF 190	MAF 626
PR_44	−7711	−7066	646 bp	-	-	0.0	-
PR_45	−7161	−6557	605 bp	-	-	0.0	-
PR_46	−6635	−6022	614 bp	-	-	0.0	-
PR_47	−6109	−5353	757 bp	-	-	0.0	-
PR_48	−5413	−4692	722 bp	-	-	0.0	-
PR_49	−4787	−4340	448 bp	-	-	0.0	-
PR_50	−4454	−3722	733 bp	-	-	0.0	-
PR_51	−3822	−3088	735 bp	-	-	0.0	-
PR_52	−3180	−2641	540 bp	-	-	0.0	-
PR_53	−2745	−1984	762 bp	-	-	0.0	-
PR_54	−2087	−1331	757 bp	-	-	0.0	-
PR_55	−1418	−951	468 bp	-	-	0.0	-
PR_56	−1095	−252	844 bp	-	-	0.0	-
PR_57	−263	355	619 bp	-	no product	1.1	0.9
PR_64	1899	355	1588 bp	-	no product	6.8	2.2
PR_73	2567	4624	2058 bp or no product	-	600 bp	10.5	17.4

^1^ position relative to the transcription start site of the *LalbFTc2* gene localized on chromosome Lalb_Chr09 at locus 7,814,590 bp, direction “reverse-complement”.

**Table 5:** The list of flowering-related transcription factors that have binding sites only in the polymorphic regions of the *LalbFTc1* promoter alignment.

Transcription Factor or Motif Name	Sequence	Position	Indel	Strand ^1^	Score
AT1G49720; AT3G19290 (ABF1; ABF4)	Chr14, GR38	557	2126 bp, 2388 bp	−	0.96
	P27174, GR38	7369	3800–3877 bp	−	0.92
AT1G55110 (IDD7)	Chr14, GR38	1942	2126 bp, 2388 bp	+	0.90
	Chr14, GR38, LD37, P27174, LAP022B	3936	264 bp	+	0.86
AT1G63030 (DDF2)	GR38, LD37, P27174	7430	3800–3877 bp	−	0.99
	GR38, LD37, P27174	8053	3800–3877 bp	+	0.89
AT2G42280 (FBH4)	Chr14, GR38	892	2126 bp, 2388 bp	−	0.98
	Chr14, GR38	908	2126 bp, 2388 bp	+	0.98
	Chr14, GR38	1085	2126 bp, 2388 bp	+	0.98
	Chr14, GR38	1401	2126 bp, 2388 bp	+	0.96
	Chr14, GR38	1408	2126 bp, 2388 bp	−	1.00
AT3G10480 (NAC050)	GR38, LD37, P27174	6631	3800–3877 bp	+	0.95
	GR38, LD37, P27174	7067	3800–3877 bp	−	0.88
AT3G28910 (MYB30)	GR38, LAP022E	3560	SNP	+	0.92
AT4G24470 (GATA25)	Chr14, GR38	460	2126 bp, 2388 bp	−	0.86
	GR38, LD37, P27174	7432	3800–3877 bp	+	0.99
	GR38, LD37, P27174	7434	3800–3877 bp	−	0.97
	GR38, LD37, P27174	8237	3800–3877 bp	+	0.86
AT4G37260 (MYB73)	LAP022E	3592	SNP	−	0.96
AT5G10030 (OBF4)	GR38, LD37, P27174	9392	3800–3877 bp	+	0.89
AT5G11260 (HY5)	GR38, LD37, P27174	9390	3800–3877 bp	−	0.89
AT5G13790 (AGL15), CARGNCAT	Chr14, GR38	161	2126 bp, 2388 bp	+/−	1
	Chr14, GR38, P27174	2302	2388 bp	+/−	1
	LAP022E	4200	SNP	−	0.96
	GR38, LD37, P27174	6325	3800–3877 bp	+/−	1
	GR38, LD37, P27174	6929	3800–3877 bp	−	0.96
	GR38, LD37, P27174	7821	3800–3877 bp	+/−	1
AT5G51870 (AGL71)	GR38, LD37, P27174	6928	3800–3877 bp	+	0.88
SORLIP2AT (phyA-induced motif)	Chr14, GR38	1735	2126 bp, 2388 bp	−	1
	LD37	7572	3800–3877 bp	+	1

^1^ +, coding strand; −, complementary strand.

## Data Availability

Data generated during this study are included in this published article and its supplementary information files. Moreover, sequence alignments constructed for the *LalbFTa1*, *LalbFTa2*, *LalbFTc1*, and *LalbFTc2* genes as well as Sanger sequencing chromatogram alignments for PCR products are available in the Zenodo repository under accession numbers 15223456 (DOI 10.5281/zenodo.15223455) https://zenodo.org/records/15223456 and 15224869 (DOI 10.5281/zenodo.15224868) https://zenodo.org/records/15224869, both records accessed on 3 June 2025.

## Supplementary Materials

The following supporting information can be downloaded at https://www.mdpi.com/article/10.3390/ijms26146858/s1.
