# Supplementary material for: Evaluation of the Global White Lupin Collection Reveals Significant Associations Between Homologous FLOWERING LOCUS T Indels and Flowering Time, Providing Validated Markers for Tracking Spring Ecotypes Within a Large Gene Pool
Source: Int J Mol Sci. 2025 Jul 17;26(14):6858. doi: 10.3390/ijms26146858 (PMC12295241; doi:10.3390/ijms26146858)
Supplement: Supplementary file 1 [file ijms-26-06858-s001.zip › ijms-3711938-supplementary captions.pdf]

Table captions

Supplementary Table S1. List of white lupin genotypes used in this study.

Supplementary Table S2. Experimental design and results of white lupin phenotypic observations in controlled environment.

Supplementary Table S3. Results of DNA isolation from white lupin genotypes.

Supplementary Table S4. Positions on transcription start sites, exons, polyA ends, primers, indels, and other polymorphic loci in *LalbFTa1*, *LalbFTa2*, *LalbFTc1,* and *LalbFTc2* alignments.

Supplementary Table S5. Primer sequences of PCR-based markers developed for white lupin *LalbFTa1*, *LalbFTa2*, *LalbFTc1,* and *LalbFTc2* indel screening.

Supplementary Table S6. Structural variants identified in the *LalbFTa1*, *LalbFTa2*, *LalbFTc1,* and *LalbFTc2* promoters and introns by the white lupin pangenome sequence alignment and PCR marker screening.

[1] position relative to the transcription start site of the *LalbFTa1* gene localized on chromosome Lalb_Chr02 at locus 14 996 788 bp, direction "forward". LD37 deletion of 46 bp was mentioned in the pangenome VCF file but was not confirmed by alignment of genome sequence contigs.
[2] markers PR_01, PR_02, PR_06, PR_07, PR_08, PR_11, PR_12, PR_14, and PR_15 did not target any large indel in the alignment and were monomorphic when resolved on agarose gels.
[3] product size difference for the PR_10 marker was too small to provide reliable scoring on agarose gels.
[4] marker PR_13 provided a monomorphic main product and an additional shorter polymorphic product.
[5] position relative to the transcription start site of the *LalbFTa2* gene localized on chromosome Lalb_Chr21 at locus 12 758 313 bp, direction "reverse-complement".
[6] product size differences for the PR_25 and PR_26 markers were too small to provide reliable scoring on agarose gels.
[7] markers PR_17, PR_21, Pr_22, PR_23, PR_24, PR_27, PR_28, and PR_29 did not target any large indel in the alignment and were monomorphic when resolved on agarose gels.
[8] position relative to the transcription start site of the *LalbFTc1* gene localized on chromosome Lalb_Chr14 at locus 5 850 617 bp, direction "forward".
[9] markers with more than 2 alleles were encoded in several variants (indicated with letters a, b, c, and d), assuming dominance or codominance of particular alleles.
[10] position relative to the transcription start site of the *LalbFTc2* gene localized on chromosome Lalb_Chr09 at locus 7 814 590 bp, reverse-complement.
[11] a rare presence/absence variant that was found by PCR screening of the white lupin germplasm testing panel of 190 genotypes. No candidate sequence polymorphism was identified in the pangenome alignment for it.
[12] GRC5262B indel coordinates indicate the positions of short sequence reads and contig misassembly; however, the exact length of the indel is unknown.

Supplementary Table S7. Results of white lupin germplasm testing panel screening with PCR-based markers developed for *LalbFTa1*, *LalbFTa2*, *LalbFTc1*, and *LalbFTc2* indels.

Supplementary Table S8. Results of white lupin full germplasm panel screening with PCR-based markers developed for *LalbFTa1*, *LalbFTa2*, *LalbFTc1*, and *LalbFTc2* indels.

Supplementary Table S9. Spearman's rank correlation coefficient and P-values calculated for comparisons of white lupin phenology traits with genotypes of PCR-based markers developed for *LalbFTa1*, *LalbFTa2*, *LalbFTc1*, and *LalbFTc2* indels.

Supplementary Table S10. P-values calculated for Spearman's rank correlations of yellow lupin phenology traits with genotypes of PCR-based markers developed for *LlutFTa1*, *LlutFTa2*, *LlutFTc1*, and *LlutFTc2* indels.

Supplementary Table S11. Results of narrow-leafed lupin germplasm panel screening with PCR-based markers developed for *LanFTc1 Jul* and *Ku* indels and correlation with phenotypic observations.

Supplementary Table S12. The list of transcription factors with candidate binding sites localized in the *LalbFTc1* gene promoter by in silico analysis.

Figure captions

Supplementary Figure S1. *LalbFTa1*, *LalbFTa2*, *LalbFTc1*, and *LalbFTc2* sequence alignments.

Supplementary Figure S2. Agarose gel electrophoregrams showing polymorphism of PCR-based markers targeting *LalbFTa1* indels.

Supplementary Figure S3. Correlation of PCR markers tagging *LalbFTa1* gene indels with white lupin phenology. Phenotyping was conducted without pre-sowing vernalization during the 2020 and 2021 growing seasons in a greenhouse located at the Institute of Plant Genetics, Polish Academy of Sciences in Poznań. Spearman's rank correlation coefficient calculated for three phenology traits (the number of days to floral bud emergence (BE), start of flowering (SF), and end of flowering (EF)) is presented in color scale from -0.5 to 0.5, whereas the Bonferroni-corrected p-value is shown in the following scheme:
***, $p < 0.0001$; **, $0.0001 \leq p < 0.001$; *, $0.001 \leq p \leq 0.05$; no symbol, $p > 0.05$ (not significant).

Supplementary Figure S4. Agarose gel electrophoregrams showing polymorphism of PCR-based markers targeting *LalbFTa2* indels.

Supplementary Figure S5. Correlation of PCR markers tagging *LalbFTa2* gene indels with white lupin phenology. Phenotyping was conducted without pre-sowing vernalization during the 2020 and 2021 growing seasons in a greenhouse located at the Institute of Plant Genetics, Polish Academy of Sciences in Poznań. Spearman's rank correlation coefficient calculated for three phenology traits (the number of days to floral bud emergence (BE), start of flowering (SF), and end of flowering (EF)) is presented in color scale from -0.5 to 0.5, whereas the Bonferroni-corrected p-value is shown in the following scheme:
***, $p < 0.0001$; **, $0.0001 \leq p < 0.001$; *, $0.001 \leq p \leq 0.05$; no symbol, $p > 0.05$ (not significant).

Supplementary Figure S6. Agarose gel electrophoregrams showing polymorphism of PCR-based markers targeting *LalbFTc1* indels.

Supplementary Figure S7. Correlation of PCR markers tagging *LalbFTc1* gene indels with white lupin phenology. Phenotyping was conducted without pre-sowing vernalization during the 2020 and 2021 growing seasons in a greenhouse located at the Institute of Plant Genetics, Polish Academy of Sciences in Poznań. Spearman's rank correlation coefficient calculated for three phenology traits (the number of days to floral bud emergence (BE), start of flowering (SF), and end

of flowering (EF)) is presented in color scale from -0.5 to 0.5, whereas the Bonferroni-corrected p-value is shown in the following scheme:
***, $p < 0.0001$; **, $0.0001 \leq p < 0.001$; *, $0.001 \leq p \leq 0.05$; no symbol, $p > 0.05$ (not significant).

Supplementary Figure S8. Agarose gel electrophoregrams showing polymorphism of PCR-based markers targeting *LalbFTc2* indels.

Supplementary Figure S9. Correlation of PCR markers tagging *LalbFTc2* gene indels with white lupin phenology. Phenotyping was conducted without pre-sowing vernalization during the 2020 and 2021 growing seasons in a greenhouse located at the Institute of Plant Genetics, Polish Academy of Sciences in Poznań. Spearman's rank correlation coefficient calculated for three phenology traits (the number of days to floral bud emergence (BE), start of flowering (SF), and end of flowering (EF)) is presented in color scale from -0.5 to 0.5, whereas the Bonferroni-corrected p-value is shown in the following scheme:
***, $p < 0.0001$; **, $0.0001 \leq p < 0.001$; *, $0.001 \leq p \leq 0.05$; no symbol, $p > 0.05$ (not significant).
