# Supplementary material for: Evaluation of the Global White Lupin Collection Reveals Significant Associations Between Homologous FLOWERING LOCUS T Indels and Flowering Time, Providing Validated Markers for Tracking Spring Ecotypes Within a Large Gene Pool
Source: Int J Mol Sci. 2025 Jul 17;26(14):6858. doi: 10.3390/ijms26146858 (PMC12295241; doi:10.3390/ijms26146858)
Supplement: Supplementary file 1 [file ijms-26-06858-s001.zip › Supplementary Figure S1_LalbFTa1, LalbFTa2, LalbFTc1, and LalbFTc2 sequence alignments.pdf]

Wojciech Bielski, Anna Surma, Michał Książkiewicz, Sandra Rychel-Bielska

# Evaluation of the global white lupin collection reveals significant associations between homologous *FLOWERING LOCUS T* indels and flowering time, providing validated markers for tracking spring ecotypes within a large gene pool

International Journal of Molecular Sciences

**Supplementary Figure S1.** *LalbFTa1*, *LalbFTa2*, *LalbFTc1*, and *LalbFTc2* sequence alignments.

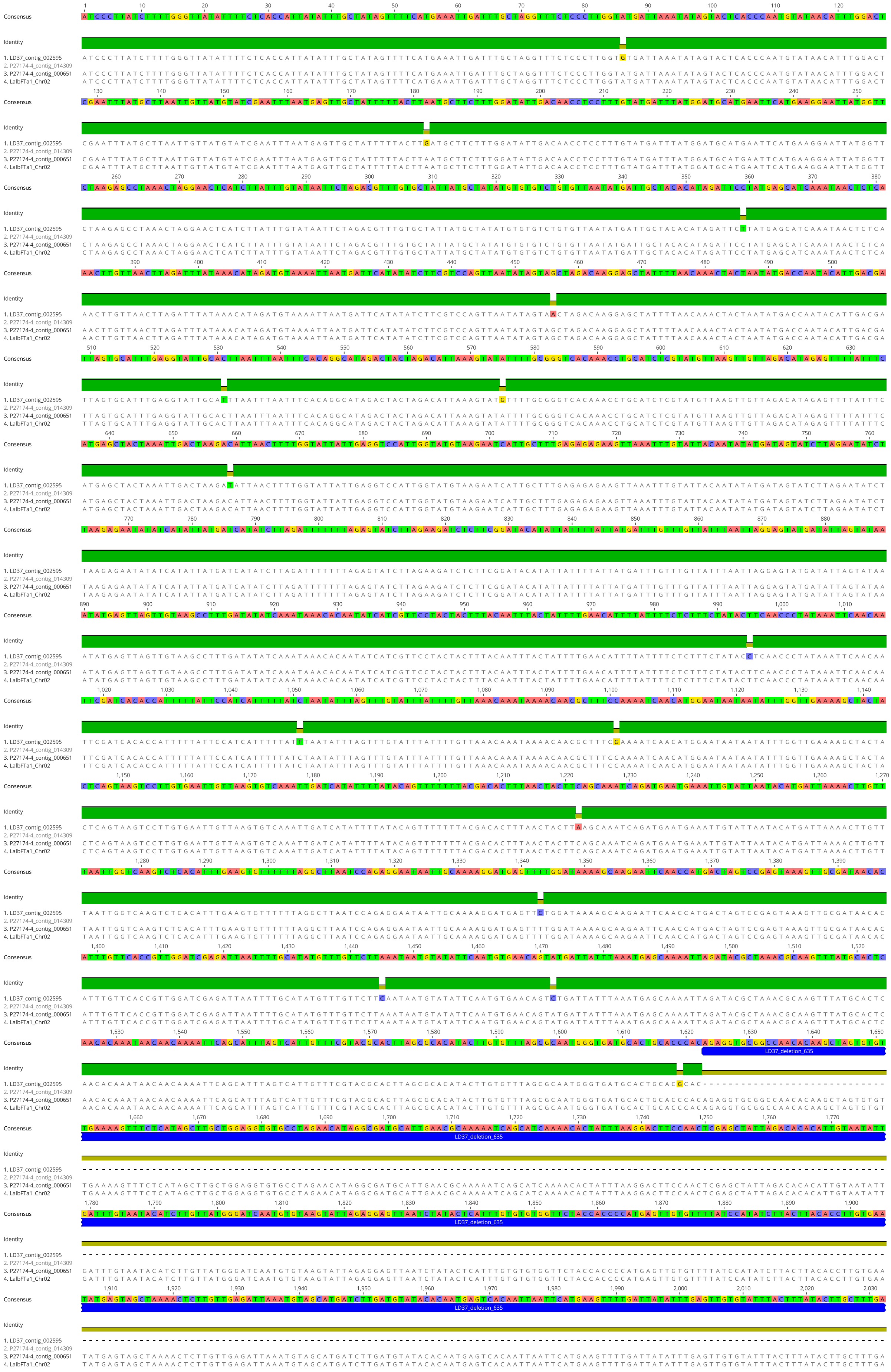
Consensus
Identity
1. LD37_contig_002595
2. P27174-4_contig_014309
3. P27174-4_contig_000651
4. LalbFTa1_Chr02
LD37_deletion_635

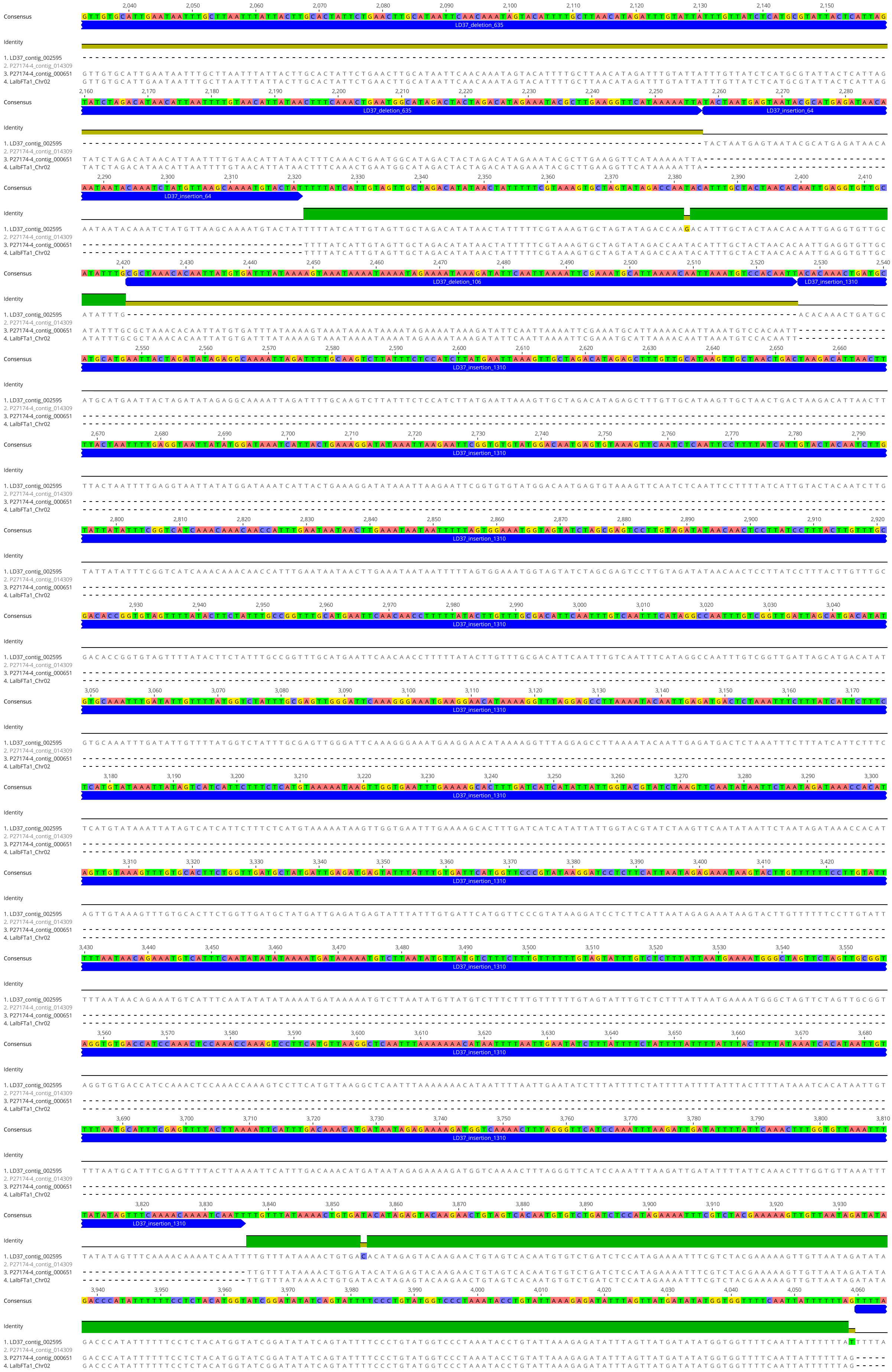
Consensus
Identity
1. LD37_contig_002595
2. P27174-4_contig_014309
3. P27174-4_contig_000651
4. LalbFTa1_Chr02
LD37_deletion_635
LD37_insertion_64
LD37_deletion_106
LD37_insertion_1310

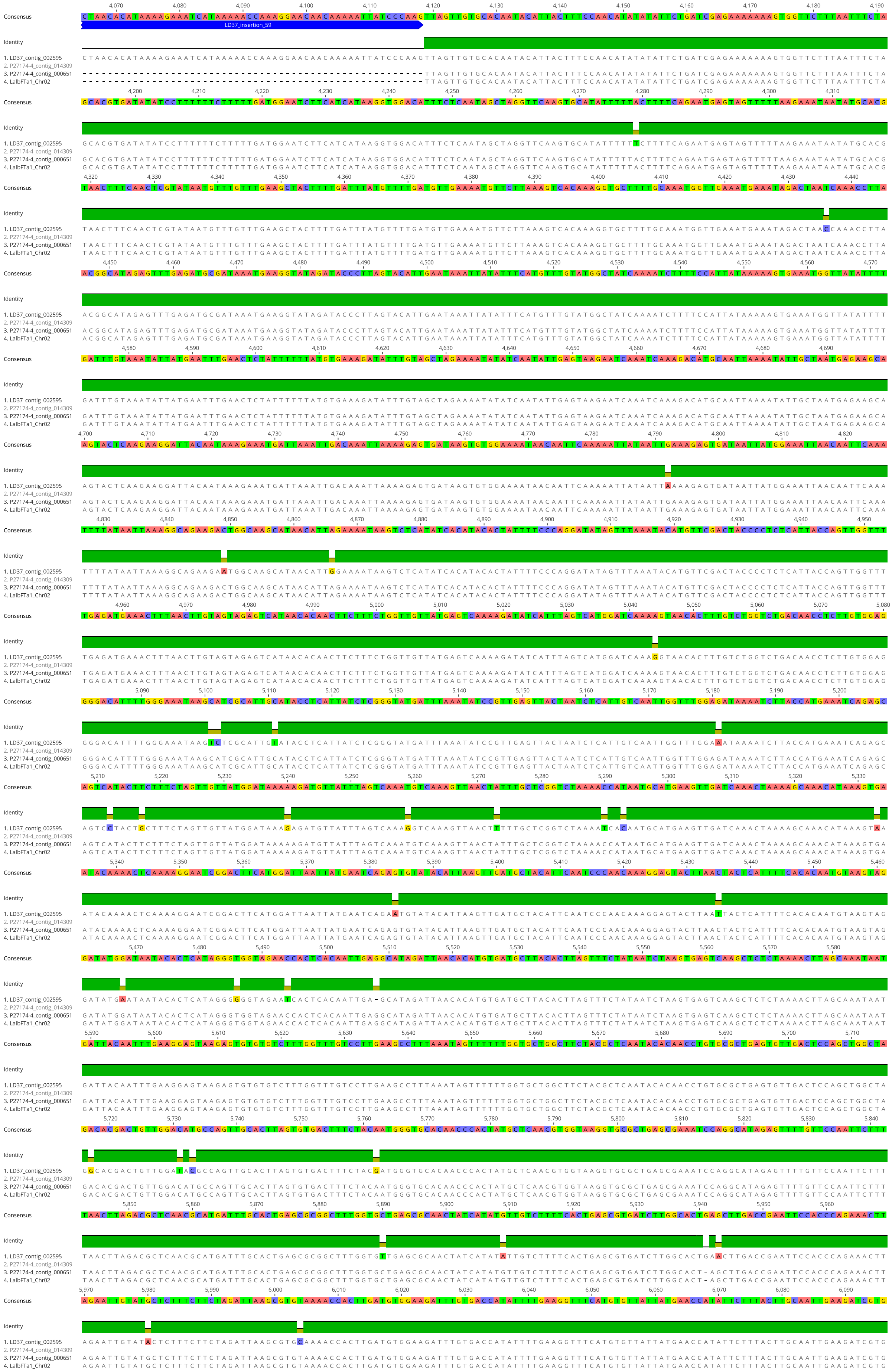

Consensus
LD37_insertion_59
Identity
1. LD37_contig_002595
2. P27174-4_contig_014309
3. P27174-4_contig_000651
4. LalbFTa1_Chr02

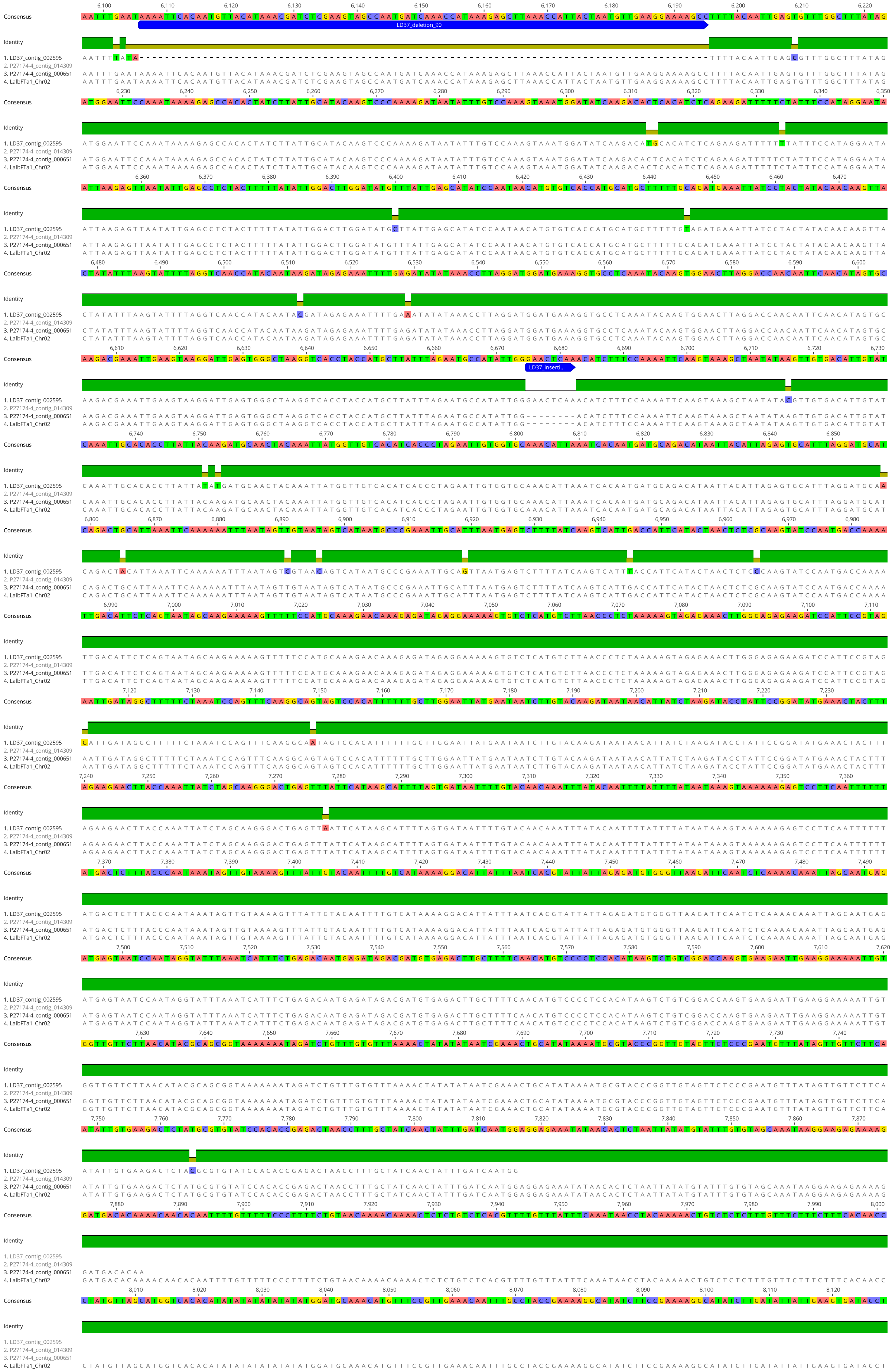
Consensus
Identity
1. LD37_contig_002595
2. P27174-4_contig_014309
3. P27174-4_contig_000651
4. LalbFTa1_Chr02
LD37_deletion_90
LD37_inserti...

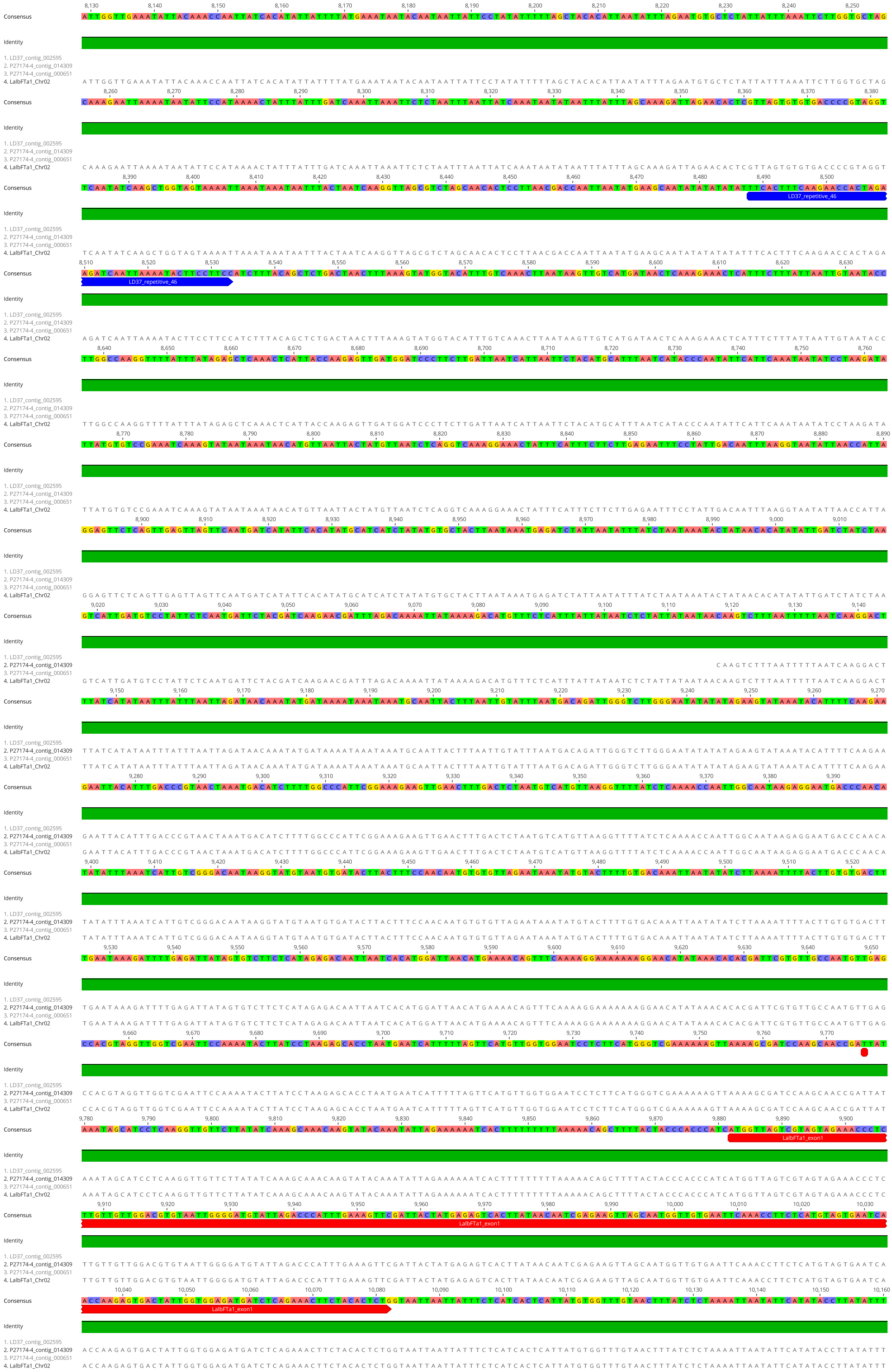
Consensus
Identity
1. LD37_contig_002595
2. P27174-4_contig_014309
3. P27174-4_contig_000651
4. LalbFTa1_Chr02
LD37_repetitive_46
LalbFTa1_exon1

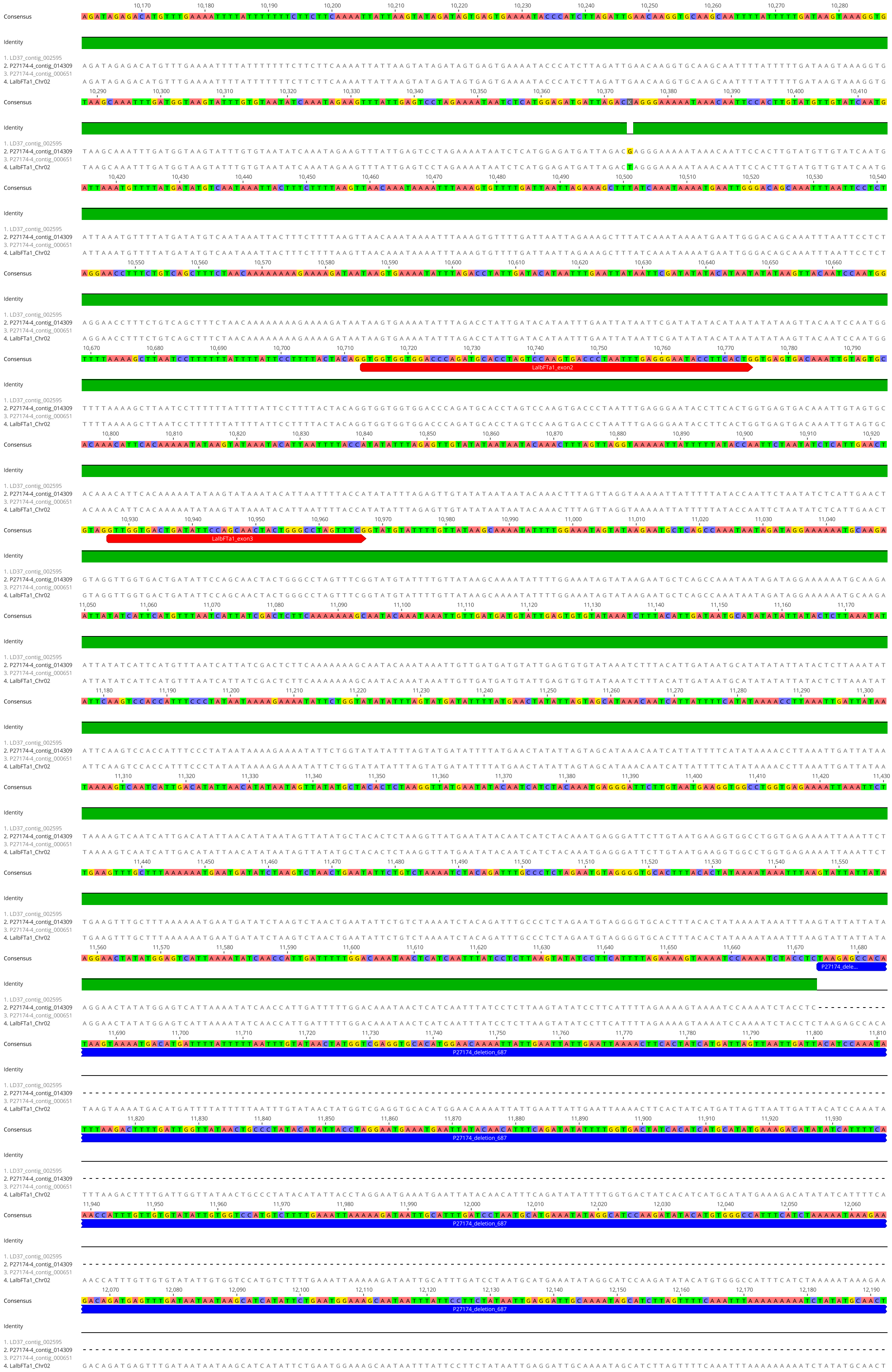
Consensus
Identity
1. LD37_contig_002595
2. P27174-4_contig_014309
3. P27174-4_contig_000651
4. LalbFTa1_Chr02
LalbFTa1_exon2
LalbFTa1_exon3
P27174_dele...
P27174_deletion_687

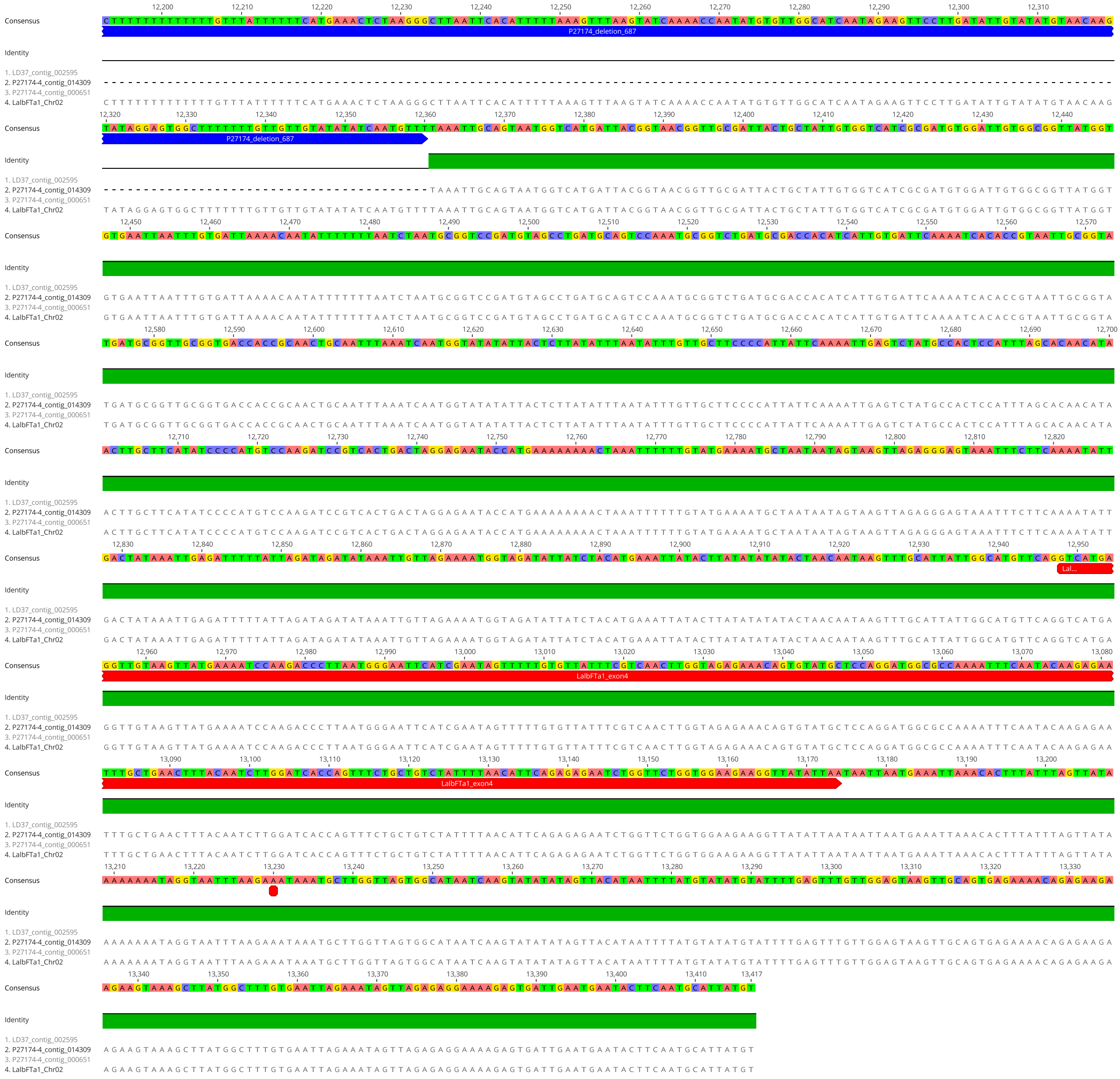

Consensus
Identity
1. LD37_contig_002595
2. P27174-4_contig_014309
3. P27174-4_contig_000651
4. LalbFTa1_Chr02
P27174_deletion_687
LalbFTa1_exon4

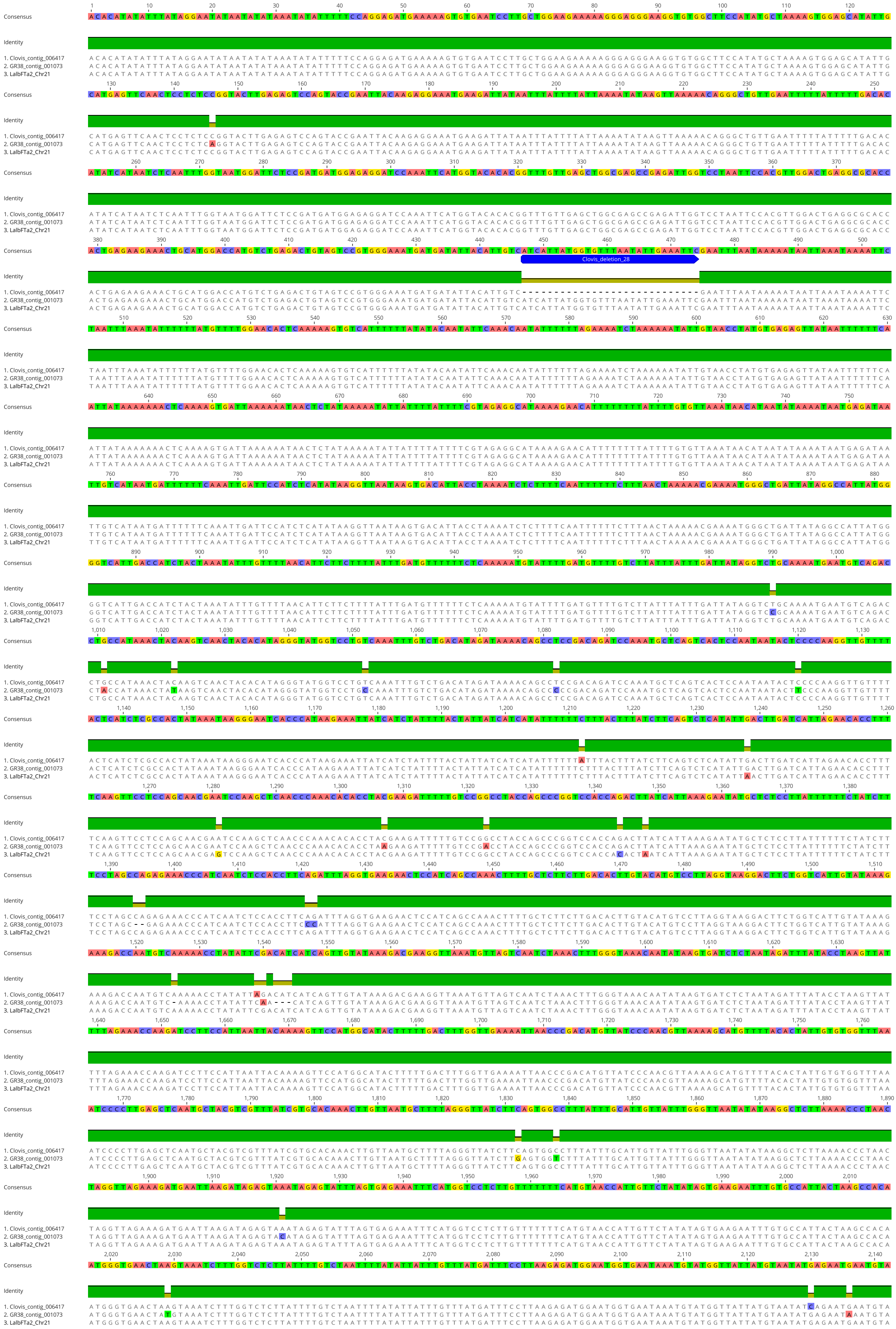
Consensus
Identity
1. Clovis_contig_006417
2. GR38_contig_001073
3. LalbFTa2_Chr21
Clovis_deletion_28

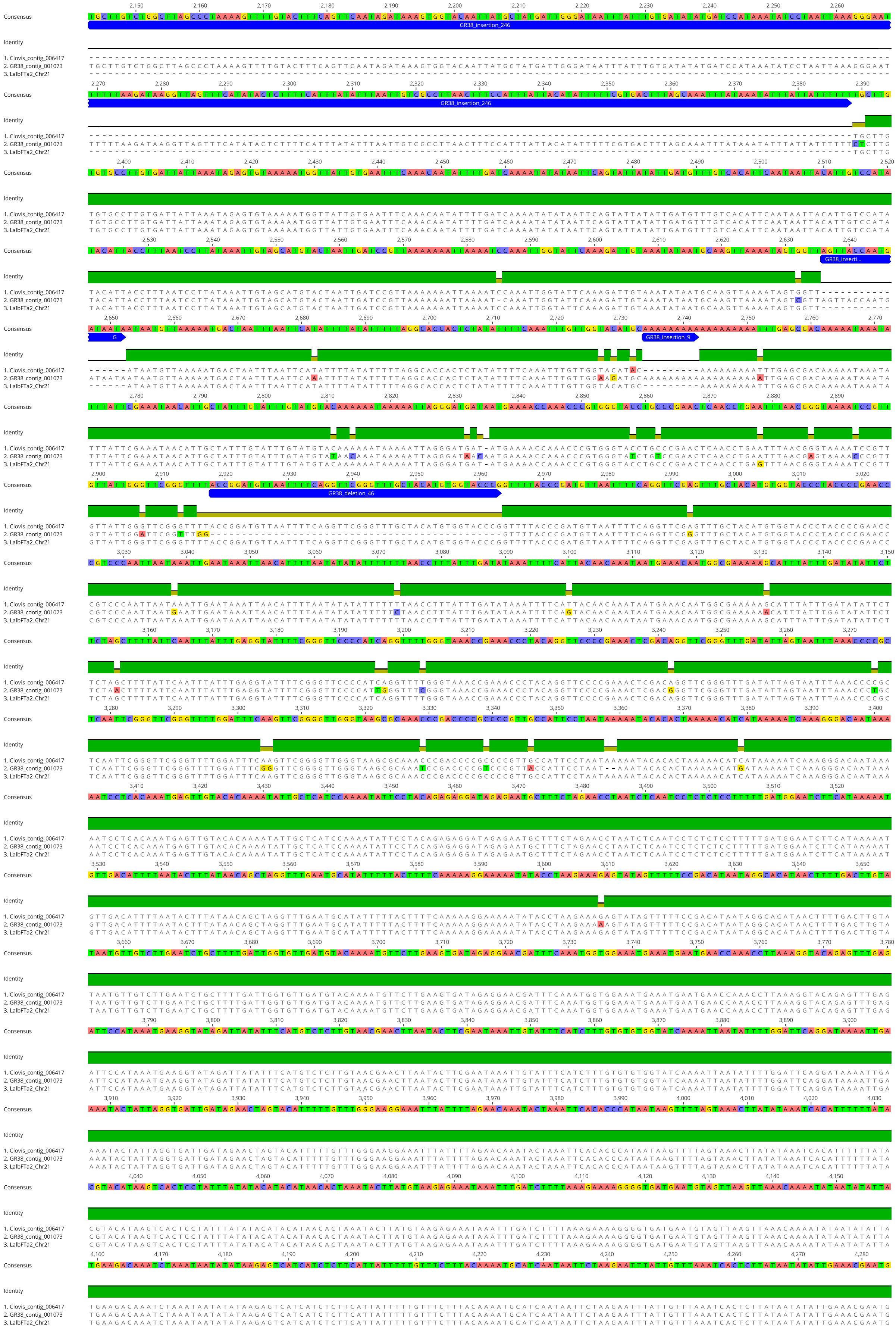
Consensus
Identity
1. Clovis_contig_006417
2. GR38_contig_001073
3. LalbFTa2_Chr21
GR38_insertion_246
GR38_insertion_9
GR38_deletion_46

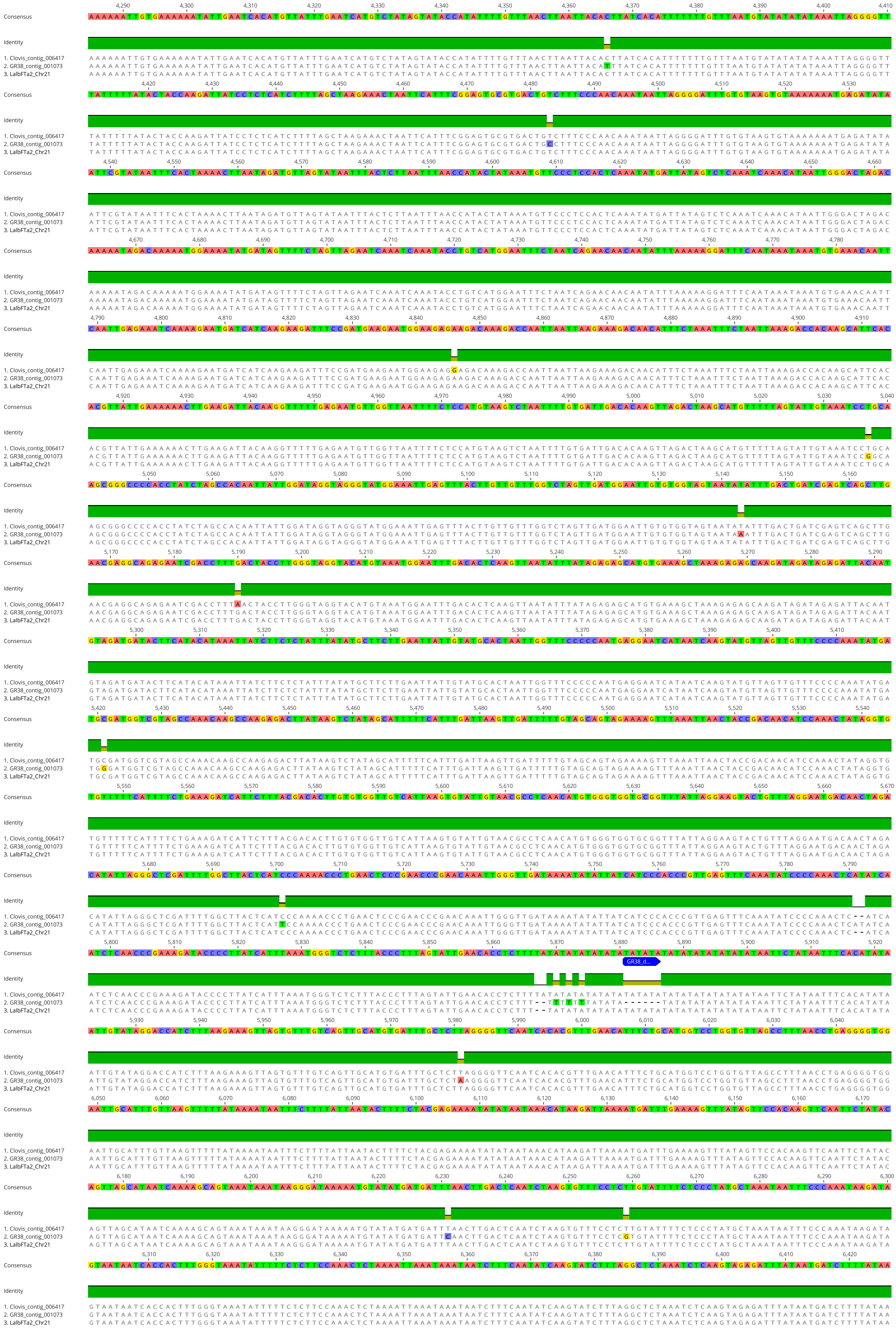

Consensus
Identity
1. Clovis_contig_006417
2. GR38_contig_001073
3. LalbFTa2_Chr21
GR38_d...

```
6,430 6,440 6,450 6,460 6,470 6,480 6,490 6,500 6,510 6,520 6,530 6,540 6,550
Consensus           CTCAATATTAGTGAGGGTACGAAGGTTTAAGATCAATTGAGTCTAAAACTCAATTATATAGGAAATATCCTAAGATTTACATAATAAATACTTAAGGATTTTCTTTTTCGTGTTTCTTAGGTGGATTC
Identity
1. Clovis_contig_006417  CTCAATATTAGTGAGGGTACGAAGGTTTAAGATCAATTGAGTCTAAAACTCAATTATATAGGAAATATCCTAAGATTTACATAATAAATACTTAAGGATTTTCTTTTTCGTGTTTCTTAGGTGGATTC
2. GR38_contig_001073     CTCAATATTAGTGAGGGTACGAAGGTTTAAGATCAATTGAGTCTAAAACTTAATTATATAGGAAATATCCTAAGATTTACATAATAAATACTTAAGGATTTTCTTTTTCGTGTTTCTTAGGTGGATTC
3. LalbFTa2_Chr21        CTCAATATTAGTGAGGGTACGAAGGTTTAAGATCAATTGAGTCTAAAACTCAATTATATAGGAAATATCCTAAGATTTACATAATAAATACTTAAGGATTTTCTTTTTCGTGTTTCTTAGGTGGATTC

6,560 6,570 6,580 6,590 6,600 6,610 6,620 6,630 6,640 6,650 6,660 6,670
Consensus           TATACAAGATATATGAAATATATATTTAAGCACTTTATCCTTAAAATAAATATACATTTCTTTAATATCCATTTAGGAATAGTGAGGTATTAGGAAGTATGTAATGCAAGGAATTCGTAGTGTCTT
Identity
1. Clovis_contig_006417  TATACAAGATATATGAAATATATATTTAAGCACTTTATCCTTAAAATAAATATACATTTCTTTAATATCCATTTAGGAATAGTGAGGTATTAGGAAGTATGTAATGCAAGGAATTCGTAGTGTCTT
2. GR38_contig_001073     TATACAAGATATATGAAATATATATTTAAGCACTTGATCCTTAAAATAAATATACATTTCTTTAATATCCATTTAGGAATAGTGAGGTATTAGGAAGTATGTAATGCAAGGAATTCGTAGTGTCTT
3. LalbFTa2_Chr21        TATACAAGATATATGAAATATATATTTAAGCACTTTATCCTTAAAATAAATATACATTTCTTTAATATCCATTTAGGAATAGTGAGGTATTAGGAAGTATGTAACGCAAGGAATTCGTAGTGTCTT

6,680 6,690 6,700 6,710 6,720 6,730 6,740 6,750 6,760 6,770 6,780 6,790 6,800
Consensus           TAACTGAATTCAGCTGTGCAATTAATGTGTTGGATCCACTATATGTTATTACTTCTTCTTCATATTTCTAAGTTCTTTCTCACAATAACTTCAAGCATAAAGGAATATCTTCTTGCACAATTCATA
Identity
1. Clovis_contig_006417  TAACTGAATTCAGCTGTGCAATTAATGTGTTGGATCCACTATATGTTATTACTTCTTCTTCATATTTCTAAGTTCTTTCTCACAATAACTTCAAGCATAAAGGAATATCTTCTTGCACAAT-----
2. GR38_contig_001073     TAACTGAATTCAGCTGTGCAATTAATGTGTTGGATCCACTATATGTTTTTACTTCTTCTTCATATTTCTAAGTTCTTTTTCACAATAACTTCAAGGATAAAGGAATATCTTCTTGCAAATTCATA
3. LalbFTa2_Chr21        TAACTGAATTCAGCTGTGCAATTAATGTGTTGGATCCACTATATGTTATTACTTCTTCTTCATATTTCTAAGTTCTTTCTCACAATAACTTCAAGCATAAAGGAATATCTTCTTGCACAAT-----

6,810 6,820 6,830 6,840 6,850 6,860 6,870 6,880 6,890 6,900 6,910 6,920 6,930
Consensus           ATTTTTAAAAGAAATTACACTCACACCCCCGAGTTTTAGTGAAATTACACAAATCACTCGTCATTTCTTGGTCATTACACCAATCATCTCTCACTCCTACACGAATCACTCATAGTTTTATACATA
Feature: GR38_inse...
Identity
1. Clovis_contig_006417  ----------GAAATTACACTCACACCCCCGAGTTTTAGTGAAATTACACAAATCACTCGTCATTTCTTGGTCATTACACCAATCATCTCTC-CTCCTACACGAATCACTCATAGTTTTATACATA
2. GR38_contig_001073     ATTTTTAAAATAAATTACACT--CACCCCCGAGTTTAATGAAATTATACAAATCACCAATCATTTCTTTGTCATTATACCAACCACCCTCATTCCTACACGAATCACCCATATTTTTATACATA
3. LalbFTa2_Chr21        ----------GAAATTACACTCACACCCCCGAGTTTTAGTGAAATTACACAAATCACTCGTCATTTCTTGGTCATTACACCAATCATCTCTC-CTCCTACACGAATCACTCATAGTTTTATACATA

6,940 6,950 6,960 6,970 6,980 6,990 7,000 7,010 7,020 7,030 7,040 7,050
Consensus           TGTTACACTGACTACCCCTGCATGTTATAAAAGTGTAATATTTTAAACAAAAAAATGTGTGATGAATGTAATTAAGTTAAACAAAATATTGTATATTGTGTAATACTTCAAATAGTACAAAAGTCA
Feature: GR...
Identity
1. Clovis_contig_006417  TGTTACACTGACTACCCCTGCATGTTATAAAAGTGTAATATTTTAAACAAAAAAATGTGTGATGAATGTAATTAAGTTAAACAAAATATTGTATATTGTGTAATACTTCAAATAGTACAAAAGTCA
2. GR38_contig_001073     TGTTACACTGACTACCCATGCATGTTATAAAAGTGTAATATTTT----AAAAAAATGTGTAATGAATATAATTAAGTTAAACAAAATATTGTATATTGTGTAATACTTCAAATAGTACAAAAGTCA
3. LalbFTa2_Chr21        TGTTACACTGACTACCCCTGCATGTTATAAAAGTGTAATATTTTAAACAAAAAAATGTGTGATGAATGTAATTAAGTTAAACAAAATATTGTATATTGTGTAATACTTCAAATAGTACAAAAGTCA

7,060 7,070 7,080 7,090 7,100 7,110 7,120 7,130 7,140 7,150 7,160 7,170 7,180
Consensus           GTGTTTTTTTTATTATTTTTGTGTCATTACAAAATACATTAATAATTTTAAAGAAATGTTTAAATAATTCTTATACTATTTTATAACGAGTCAAATAATGAAGACGTATTGACTCTTATATTATTT
Identity
1. Clovis_contig_006417  GTGTTTTTTTTATTATTTTTGTGTCATTACAAAATACATTAATAATTTTAAAGAAATGTTTAAATAATTCTTATACTATTTTATAACGAGTCAAATAATGAAGACGTATTGACTCTTATATTATTT
2. GR38_contig_001073     GTG-TTTTTTTATTATTTTTGTGTCATTACAAAATACATTAATAATTTCAAAAAAATGTTTAAATAATTCTTATACTATTTTATAACGAGTCAAATAATGAAGACGTATTGACTCTTATATTATTT
3. LalbFTa2_Chr21        GTGTTTTTTTTATTATTTTTGTGTCATTACAAAATACATTAATAATTTTAAAGAAATGTTTAAATAATTCTTATACTATTTTATAACGAGTCAAATAATGAAGACGTATTGACTCTTATATTATTT

7,190 7,200 7,210 7,220 7,230 7,240 7,250 7,260 7,270 7,280 7,290 7,300
Consensus           GAATTTCTCTATACAATATACAATATTTTTTTAATTAATTACACACGTCACACATTTTTTGTTTAACATATTACATTTTTATATCATGCAGGGGTAGTCAGTGTAACATATGTACAAAATCAGGGG
Identity
1. Clovis_contig_006417  GAATTTCTCTATACAATATACAATATTTTTTTAATTAATTACACACGTCACACATTTTTTGTTTAACATATTACATTTTTATATCATGCAGGGGTAGTCAGTGTAACATATGTACAAAATCAGGGG
2. GR38_contig_001073     GAATTTCTCTATACAATATACAATATTTTTTTAATTAATTACACACGTCACACATTTTTTGTTTAACATATTACACTTTTATATCATGCAGGGGTAGTCAGTGTAACATATGTACAAAATCAGGGG
3. LalbFTa2_Chr21        GAATTTCTCTATACAATATACAATATTTTTTTAATTAATTACACACGTCACACATTTTTTGTTTAACATATTACATTTTTATATCATGCAGGGGTAGTCAGTGTAACATATGTACAAAATCAGGGG

7,310 7,320 7,330 7,340 7,350 7,360 7,370 7,380 7,390 7,400 7,410 7,420 7,430
Consensus           TGGTTCGTGTAAAGACAAAAAAAGTGAGTGATGATTTATATAATTTCACTAAAACTCAGGGAGGTGTGAATGTAATTTACTCTAATTTTTTATGATTATTCATGCCTGTTCACGATTTAATCTCCT
Feature: GR38_insertion_13
Identity
1. Clovis_contig_006417  TGGTTCGTGTAAAGACAAAAAAAGTGAGTGATGATTTATATAATTTCACTAAAACTCAGGGAGGTGTGAATGTAA-------------TTTATGATTAT---TGCCTGTTCACGATTTAATCTCCT
2. GR38_contig_001073     TGGTTCGTGTAAGGACAAAAAATG-GAGTGGTGATTTATATAATTTCACTAAAATTCAGGG-GGTGTGAGTGTAATTTACTCTAATTTTTTATGATTATTCATGTATGTGCACGATTTAATCTCCT
3. LalbFTa2_Chr21        TGGTTCGTGTAAAGACAAAAAAAGTGAGTGATGATTTATATAATTTCACTAAAACTCAGGGAAGTGTGAATGTAA-------------TTTATGATTAT---TGCCTGTTCACGATTTAATCTCCT

7,440 7,450 7,460 7,470 7,480 7,490 7,500 7,510 7,520 7,530 7,540 7,550 7,560
Consensus           TTGTTTGCAAAGCGGTTAGAGTACAAGATTTGCATTTCAGAGTACTAAGTTTTTCTTACTAAAAACTTAACATTTAACGTCCTAATAATAATTGAAAAACAAACATTCTCATTAAATTTTAATATC
Identity
1. Clovis_contig_006417  TTGTTTGCAAAGCGGTTAGAGTACAAGATTTGCATTTCAGAGTACTAAGTTTTTCTTACTAAAAACTTAACATTTAACGTCCTAATAATAATTGAAAAACAAACATTCTCATTAAATTTTAATATC
2. GR38_contig_001073     TTGTTTGCAAAGCGGTTAGAGTACAAGATTTGCATTTCAGAGTACTAAGTTTTTCTTACTAAAAACTTAACATTTAATGTCCTAATAATAATTGAAAAACAAACATTCTCATTAAATTTTAATATC
3. LalbFTa2_Chr21        TTGTTTGCAAAGCGGTTAGAGTACAAGATTTGCATTTCAGAGTACTAAGTTTTTCTTACTAAAAACTTAACATTTAACGTCCTAATAATAATTGAAAAACAAACATTCTCATTAAATTTTAATATC

7,570 7,580 7,590 7,600 7,610 7,620 7,630 7,640 7,650 7,660 7,670 7,680
Consensus           AATTATATGAGATAAACTCAAATAATTGGACGAGTAAGATCATATATGCATGTAACTTTATATATACATTTCACTATACGCATTATCAGTAAACATATTTACTCATTTAGATGTGATGACTCGTTG
Identity
1. Clovis_contig_006417  AATTATATGAGATAAACTCAAATAATTGGACGAGTAAGATCATATATGCATGTAACTTTATATATACATTTCACTATACGCATTATCAGTAAACATATTTACTCATTTAGATGTGATGACTCGTTG
2. GR38_contig_001073     AATTATATGAGATAAACTCAAATAATTGGACGAGTAAGATCATATATGCATGTAACTTTATATATACATTTCACTATACGCATTATCAGTAAACATATTTACTCATTTAGATGTGATGACTCGTTG
3. LalbFTa2_Chr21        AATTATATGAGATAAACTCAAATAATTGGACGAGTAAGATCATATATGCATGTAACTTTATATATACATTTCACTATACGCATTATCAGTAAACATATTTACTCATTTAGATGTGATGACTCGTTG

7,690 7,700 7,710 7,720 7,730 7,740 7,750 7,760 7,770 7,780 7,790 7,800 7,810
Consensus           GATGAACTTAATCACCAGAAACAAACTTAATATTTCATAAGGATTTTGTAACATATATTAGTATGGAATCCTATGAAAATCTTTTCCCTTAGATTATATTTTTTAAATTAATAACATCTTATGCAT
Identity
1. Clovis_contig_006417  GATGAACTTAATCACCAGAAACAAACTTAATATTTCATAAGGATTTTGTAACATATATTAGTATGGAATCCTATGAAAATCTTTTCCCTTAGATTATATTTTTTAAATTAATAACATCTTATGCAT
2. GR38_contig_001073     GATGAACTTAATCACCAGAAACAAACTTAATATTTCATAAGGATTTTGTAACATATATTAGTATGGAATCCTATGAAAAACTTTTTCCTTAGATTATATTTTTTAAATTAATAACATCTTATGCAT
3. LalbFTa2_Chr21        GATGAACTTAATCACCAGAAACAAACTTAATATTTCATAAGGATTTTGTAACATATATTAGTATGGAATCCTATGAAAATCTTTTCCCTTAGATTATATTTTTTAAATTAATAACATCTTATGCAT

7,820 7,830 7,840 7,850 7,860 7,870 7,880 7,890 7,900 7,910 7,920 7,930
Consensus           AAGTAATCACAGAAATATAATTAATTATTTGTTATTCATGAAATCAAGTAAAATTAGATGTGCTCCTCATGGTAGATATGGAGTAACGCAAATTATAACACATATATATTTAAGTTAAAAGTTT
Identity
1. Clovis_contig_006417  AAGTAATCACAGAAATATAATTAATTATTTGTTATTCATGAAATCAAGTAAAATTAGATGTGCTCCTCATGGTAGATATGGAGTAACGCAAATTATAACAC--ATATATATTTAAGTTAAAAGTTT
2. GR38_contig_001073     AAATAATCACAGAAATATAATTAATTATTTGTTATTCATGAAATCAAGTAAAATTAGATGTGCTCCTCATGGTAGATATGGAGTAACGCAAATTATAACAC--ATATATATTTAAGTTAAAAGTTT
3. LalbFTa2_Chr21        AAGTAATCACAGAAATATAATTAATTATTTGTTATTCATGAAATCAAGTAAAATTAGATGTGCTCCTCATGGTAGATATGGAGTAACGCAAATTATAACACATATATATTTAAGTTAAAAGTTT

7,940 7,950 7,960 7,970 7,980 7,990 8,000 8,010 8,020 8,030 8,040 8,050 8,060
Consensus           AATTATTAAATATTGTTTCTAACCAATTATATCTCATGGTACATGAAGTAATACAAATTGTGGGAAAAAAGATGTGCTGTCAAATAAGGCTGGACTCCAAATAAGGATCATGTGTAACAATATGAT
Identity
1. Clovis_contig_006417  AATTATTAAATATTGTTTCTAACCAATTATATCTCATGGTACATGAAGTAATACAAATTGTGGGAAAAAAGATGTGCTGTCAAATAAGGCTGGACTCCAAATAAGGATCATGTGTAACAATATGAT
2. GR38_contig_001073     AATTATTAAATATTTTTTCTAACCAATTATATCTCATGGTACATGAAGTAATACAAATTGTGGGAAAAAAGATGTGCTGTCAAATAAGGCTGGACTCCAAATAAGGATCATGTGTAACAATATGAT
3. LalbFTa2_Chr21        AATTATTAAATATTGTTTCTAACCAATTATATCTCATGGTACATGAAGTAATACAAATTGTGGGAAAAAAGATGTGTTGTCAAATAAGGCTGGACTCCAAATAAGGATCATGTGTAACAATATGAT

8,070 8,080 8,090 8,100 8,110 8,120 8,130 8,140 8,150 8,160 8,170 8,180 8,190
Consensus           TATGTTCTAATGTTTCAAGCGAATTAACTTTTTTCACGTAATATAAAATTTTTATTTTGTATGGTAGATATTTTTATTGTAATAATGGCTAACATGCATGAAAACAATATTTATTTTATGGACAAA
Identity
1. Clovis_contig_006417  TATGTTCTAATGTTTCAAGCGAATTAACTTTTTTCACGTAATATAAAATTTTTATTTTGTATGGTAGATATTTTTATTGTAATAATGGCTAACATGCATGAAAACAATATTTATTTTATGGACAAA
2. GR38_contig_001073     TATGTTCTAATGTTTCAAGCGAATTAACTTTTTTCACGTAATATAAAATTTTTATTTTGTATGGTAGATATTTTTATTGTAATAATGGCTAACATGCATGAAAACAATATTTATTTTATGGACAAA
3. LalbFTa2_Chr21        TATGTTCTAATGTTTCAAGCGAATTAACTTTTTTCACGTAATATAAAATTTTTATTTTGTATGGTAGATATTTTTATTGTAATAATGGCTAACATGCATGAAAACAATATTTATTTTATGGACAAA

8,200 8,210 8,220 8,230 8,240 8,250 8,260 8,270 8,280 8,290 8,300 8,310
Consensus           ACTTAGGTACAATTCCTTAGGTACAAATGGTGTTTATTACCTATTAAAAGGTGACACGTGTTCATTATCACCTATATTTTGAAGAGAGAGATTCTTAATTACAAAAAAAGAGAAAGACTTAATGAT
Identity
1. Clovis_contig_006417  ACTTAGGTACAATTCCTTAGGTACAAATGGTGTTTATTACCTATTAAAAGGTGACACGTGTTCATTATCACCTATATTTTGAAGAGAGAGATTCTTAATTACAAAAAAAGAGAAAGACTTAATGAT
2. GR38_contig_001073     ACTTAGGTACAATTCCTTAGGTACAAATAGTGTTTATTACCTATTAAAAGGTGACACGTGTTCATTATCACCTATATTTTGAAGAGAGAGATTCTTAATTACAAAAAAAGAGAAAGACTTAATGAT
3. LalbFTa2_Chr21        ACTTAGGTACAATTCCTTAGGTACAAATGGTGTTTATTACCTATTAAAAGGTGACACGTGTTCATTATCACCTATATTTTGAAGAGAGAGATTCTTAATTACAAAAAAAGAGAAAGACTTAATGAT

8,320 8,330 8,340 8,350 8,360 8,370 8,380 8,390 8,400 8,410 8,420 8,430 8,440
Consensus           ATAAATGAACACGTGTCACATTTTAATAGGCTACAAACACCATTTGTACCTAAGGAATTGTACCTAAGTTTTGTCCTTATTTTATTTAGAAAAAAGAGTAACATGAAAACACGATTCGTGTTGCCA
Identity
1. Clovis_contig_006417  ATAAATGAACACGTGTCACATTTTAATAGGCTACAAACACCATTTGTACCTAAGGAATTGTACCTAAGTTTTGTCCTTATTTTATTTAGAAAAAAGAGTAACATGAAAACACGATTCGTGTTGCCA
2. GR38_contig_001073     ATAAATGAACACGTGTCACATTTTAATAGGCTACAAACACCATTTGTACCTAAGGAATTGTACCTAAGTTTTGTCCTTATTTTATTTAGAAAAAAGAGTAACATGAAAACACGATTCGTGTTGCCA
3. LalbFTa2_Chr21        ATAAATGAACACGTGTCACATTTTAATAGGCTACAAACACCATTTGTACCTAAGGAATTGTACCTAAGTTTTGTCCTTATTTTATTTAGAAAAAAGAGTAACATGAAAACACGATTCGTGTTGCCA

8,450 8,460 8,470 8,480 8,490 8,500 8,510 8,520 8,530 8,540 8,550 8,560
Consensus           ATGTTGAGCCACGTACATTGGACATTAAGTCCAAATACTTACTAATACAGAAAAGAAGTGATTCTTTCGAGCAACAAATTATTCCTTTCAATTTTTTGATTGAATCCTTTTCATGTGTCGAAAAAA
Identity
1. Clovis_contig_006417  ATGTTGAGCCACGTACATTGGACATTAAGTCCAAATACTTACTAATACAGAAAAGAAGTGATTCTTTCGAGCAACAAATTATTCCTTTCAATTTTTTGATTGAATCCTTTTCATGTGTCGAAAAAA
2. GR38_contig_001073     ATGTTGAGCCACGTACATTGGACATTAAGTCCAAATACTTACTAATACAGAAAAGAAGTGATTCTTTCGAGCAACAAATTATTCCTTTCAATTTTTTGATTGAATCCTTTTCATGTGTCGAAAAAA
3. LalbFTa2_Chr21        ATGTTGAGCCACGTACATTGGACATTAAGTCCAAATACTTACTAATACAGAAAAGAAGTGATTCTTTCGAGCAACAAATTATTCCTTTCAATTTTTTGATTGAATCCTTTTCATGTGTCGAAAAAA
```

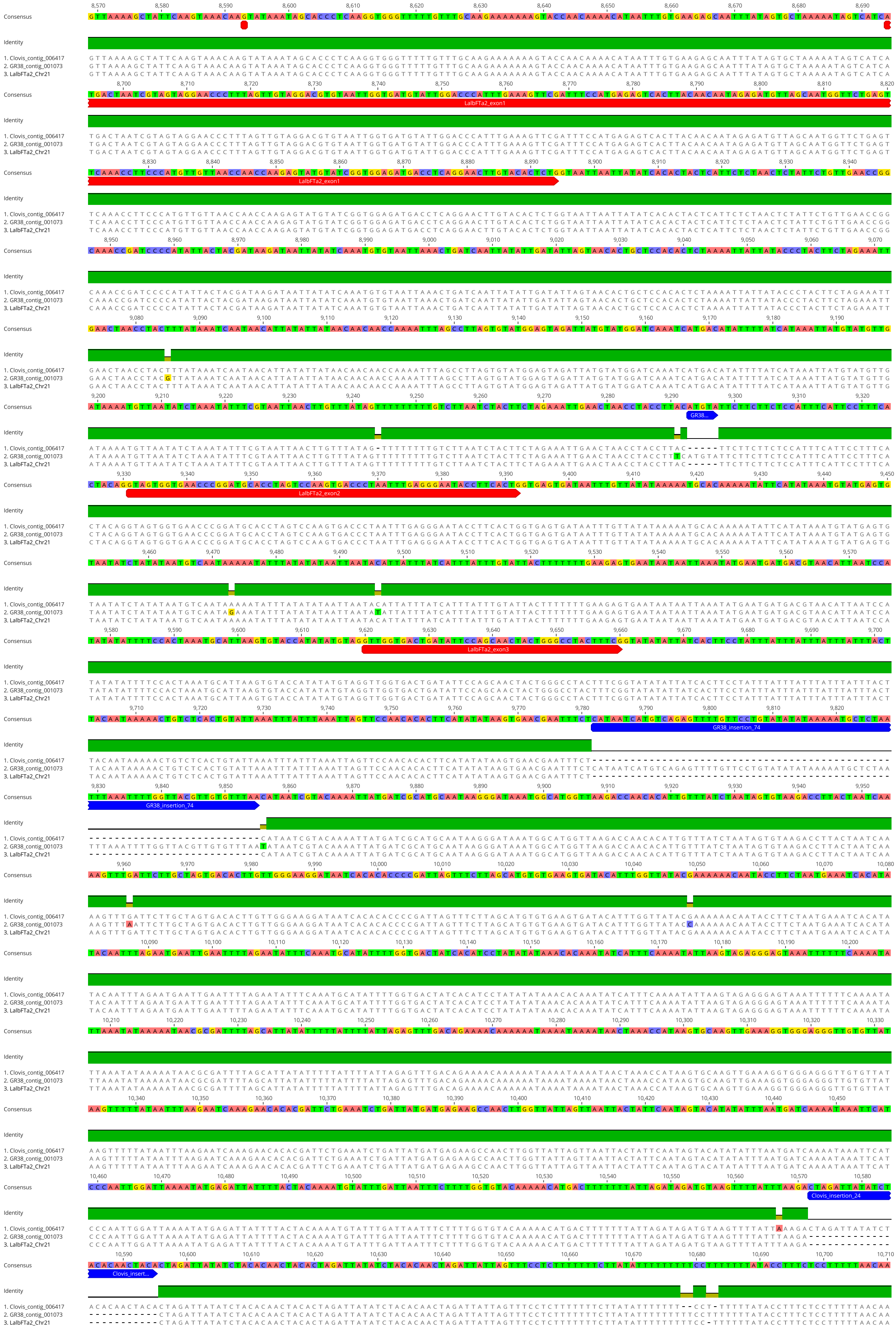
Consensus
Identity
1. Clovis_contig_006417
2. GR38_contig_001073
3. LalbFTa2_Chr21
LalbFTa2_exon1
LalbFTa2_exon2
LalbFTa2_exon3
GR38...
GR38_insertion_74
Clovis_insertion_24
Clovis_insert...

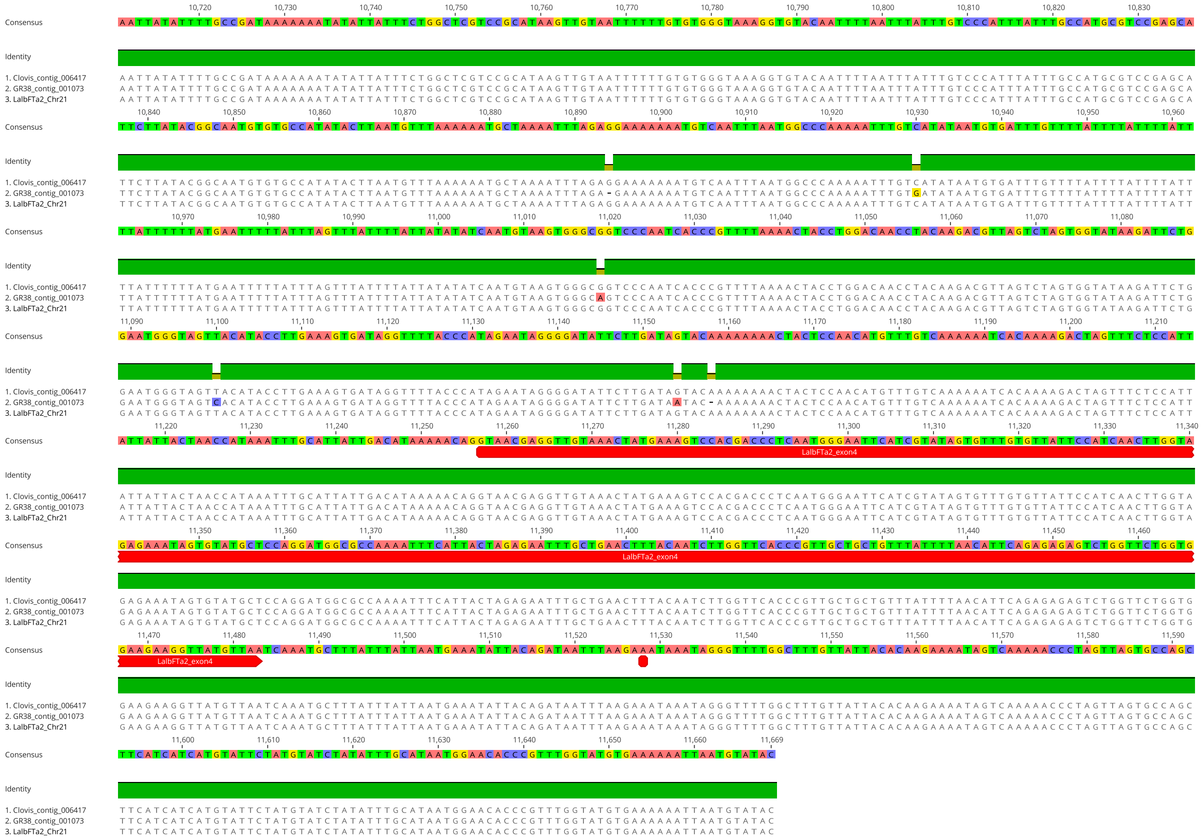

Consensus
Identity
1. Clovis_contig_006417
2. GR38_contig_001073
3. LalbFTa2_Chr21
LalbFTa2_exon4

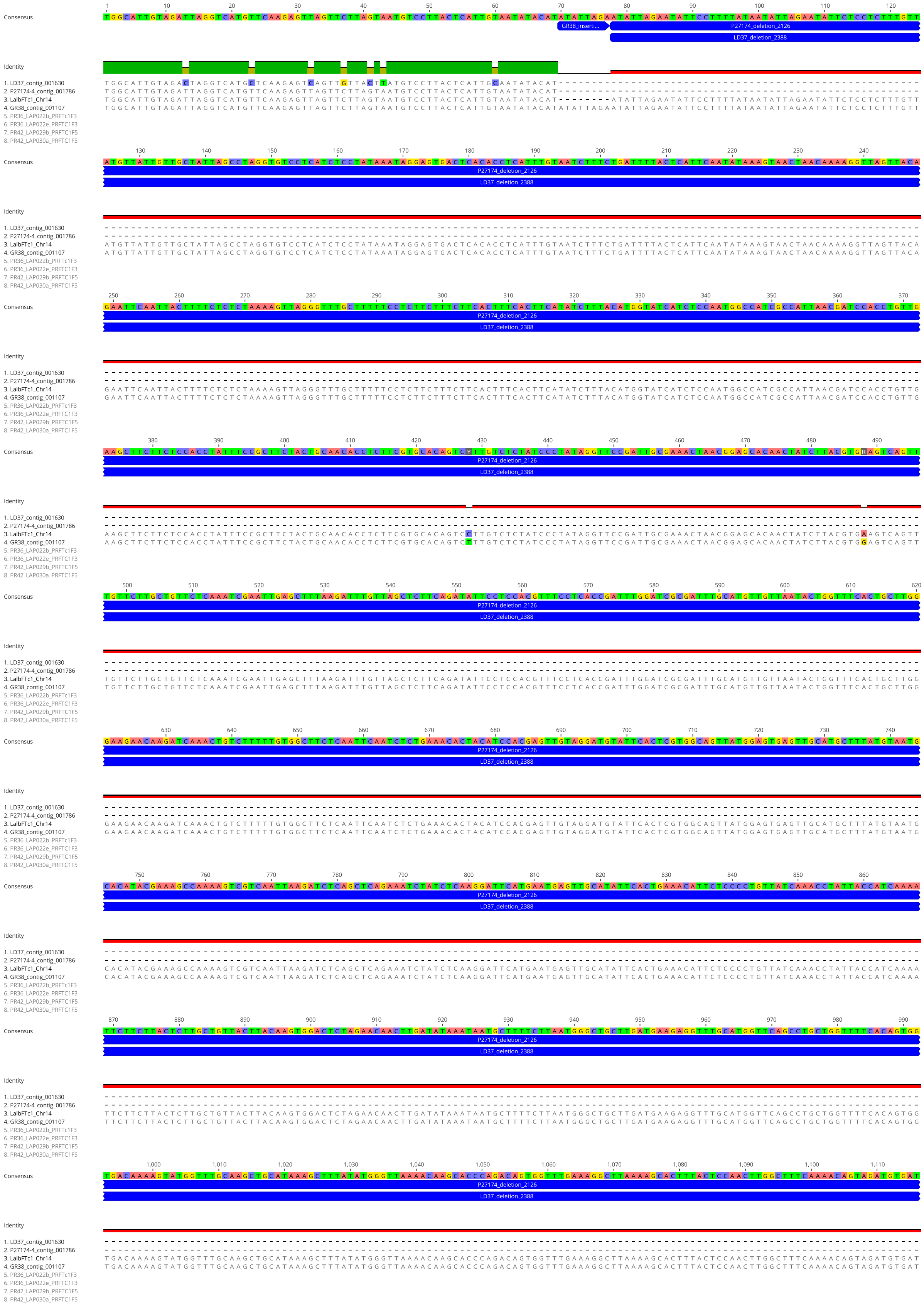
Consensus
GR38_inserti...
P27174_deletion_2126
LD37_deletion_2388
Identity
1. LD37_contig_001630
2. P27174-4_contig_001786
3. LalbFTc1_Chr14
4. GR38_contig_001107
5. PR36_LAP022b_PRFTc1F3
6. PR36_LAP022e_PRFTC1F3
7. PR42_LAP029b_PRFTC1F5
8. PR42_LAP030a_PRFTC1F5

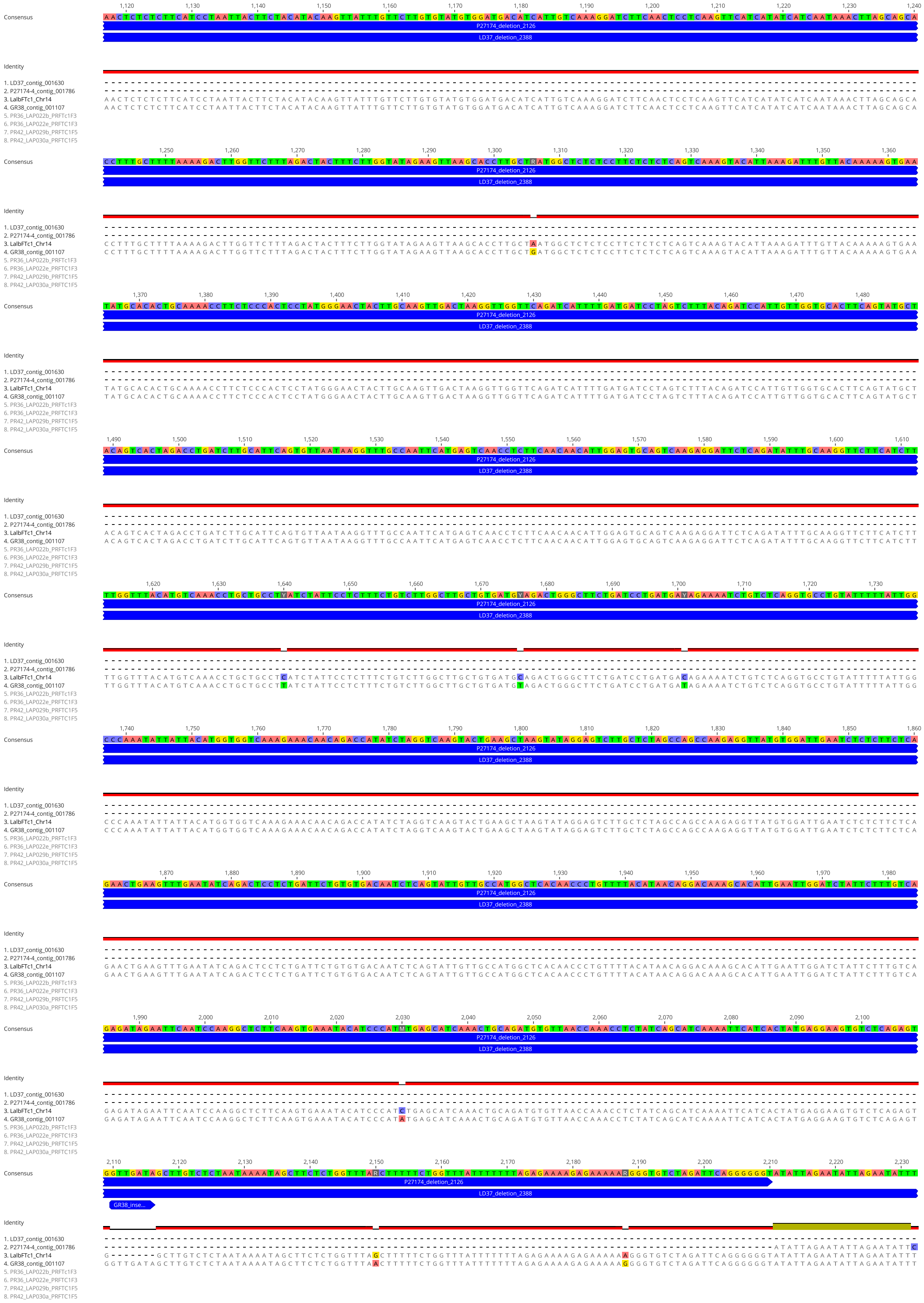

Consensus
P27174_deletion_2126
LD37_deletion_2388
GR38_inse...
Identity
1. LD37_contig_001630
2. P27174-4_contig_001786
3. LalbFTc1_Chr14
4. GR38_contig_001107
5. PR36_LAP022b_PRFTc1F3
6. PR36_LAP022e_PRFTC1F3
7. PR42_LAP029b_PRFTC1F5
8. PR42_LAP030a_PRFTC1F5
1,120 – 2,230

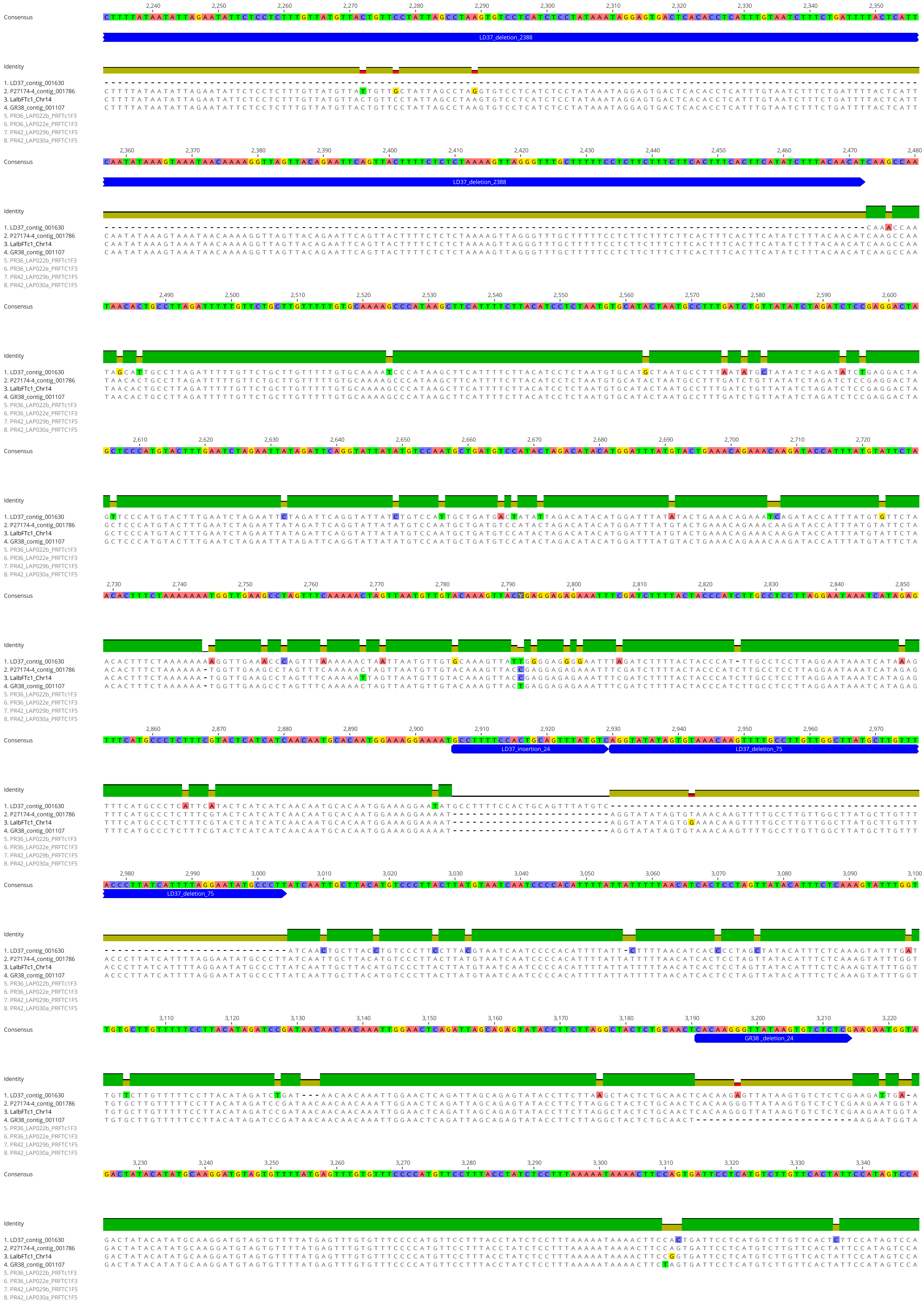

Consensus
Identity
1. LD37_contig_001630
2. P27174-4_contig_001786
3. LalbFTc1_Chr14
4. GR38_contig_001107
5. PR36_LAP022b_PRFTc1F3
6. PR36_LAP022e_PRFTC1F3
7. PR42_LAP029b_PRFTC1F5
8. PR42_LAP030a_PRFTC1F5
LD37_deletion_2388
LD37_insertion_24
LD37_deletion_75
GR38_deletion_24

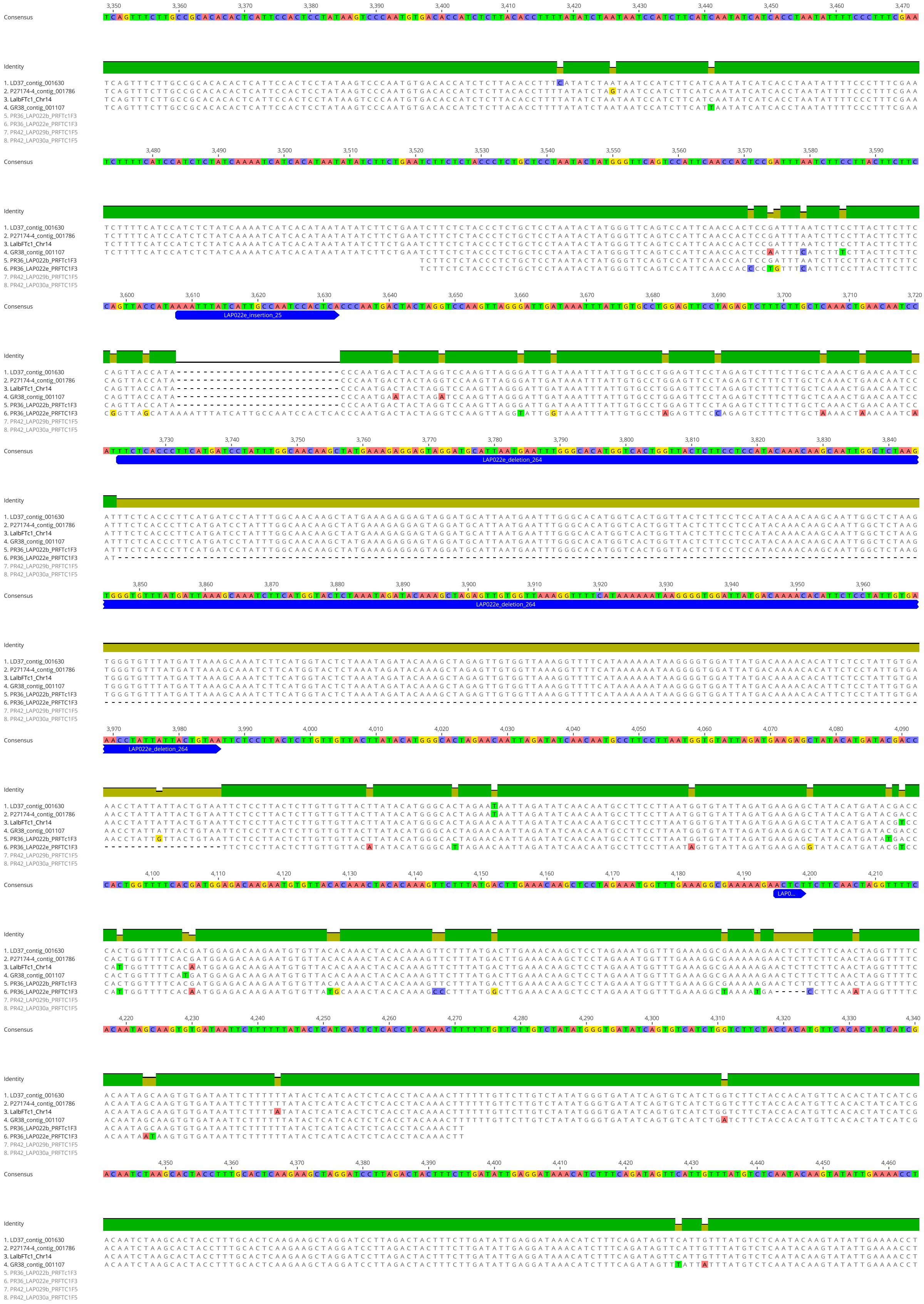
Consensus
Identity
1. LD37_contig_001630
2. P27174-4_contig_001786
3. LalbFTc1_Chr14
4. GR38_contig_001107
5. PR36_LAP022b_PRFTc1F3
6. PR36_LAP022e_PRFTC1F3
7. PR42_LAP029b_PRFTc1F5
8. PR42_LAP030a_PRFTC1F5
LAP022e_insertion_25
LAP022e_deletion_264
LAP0...

4,470 4,480 4,490 4,500 4,510 4,520 4,530 4,540 4,550 4,560 4,570 4,580

Consensus TCTCATTAGAGTCAACATAAATAATGCTAAGCATATTCCTACTCCTATAGATCTGTGGTTTGTGCACTACAGTATGCCACAATAATCAGACCTGACCTTGCCTAAGGGTGAACAAGGTGTGTCA

Identity

1. LD37_contig_001630 TCTCATTAGAGTCAACATAAATAATGCTAAGCATATTCCTACTCCTATAGATCTGTGGTTTGTGCACTACAGTATGCCACAATAATCAGACCTGACCTTGCCTAAGGGTGAACAAGGTGTGTCA
2. P27174-4_contig_001786 TCTCATTAGAGTCAACATAAATAATGCTAAGCATATTCCTACTCCTATAGATCTGTGGTTTGTGCACTACAGTATGCCACAATAATCAGACCTGACCTTGCCTAAGGGTGAACAAGGTGTGTCA
3. LalbFTc1_Chr14 TCTCATTAGAGTCAACATAAATAATGCTAAGCATATTCCTACTCCTATAGATCTGTGGTTTGTGCACTACAGTATGCCACAATAATCAGACCTGACCTTGCCTAAGGGTGAACAAGGTGTGTCA
4. GR38_contig_001107 TCTCATTAGAGTCAACATAAATAATGCTAAGCATATTCCTACTCCTATAGATCTGTGGTTTGTGCACTACAGTATGCCACAATAATCAGACCTGACCTTGCCTAAGGGTGAACAAGGTGTGTCA
5. PR36_LAP022b_PRFTc1F3
6. PR36_LAP022e_PRFTC1F3
7. PR42_LAP029b_PRFTC1F5
8. PR42_LAP030a_PRFTC1F5

4,590 4,600 4,610 4,620 4,630 4,640 4,650 4,660 4,670 4,680 4,690 4,700 4,710

Consensus GTATATGAGTCAACCTTTACCATAACACTGGAATGCAATAAAGAGAATCCTAAGACATCTACAAGGGTTATCAACCTTTGGTCTTCATGTTAAACCTACTACACCCCTTGTCCATCTGTCTCTC

Identity

1. LD37_contig_001630 GTATATGAGTCAACCTTTACCATAACACTGGAATGCAATAAAGAGAATCCTAAGACATCTACAAGGGTTATCAACCTTTGGTCTTCATGTTAAACCTACTACACCCCTTGTCCATCTGTCTCTC
2. P27174-4_contig_001786 GTATATGAGTCAACCTTTACCATAACACTGGAATGCAATAAAGAGAATCCTAAGACATCTACAAGGGTTATCAACCTTTGGTCTTCATGTTAAACCTACTACACCCCTTGTCCATCTGTCTCTC
3. LalbFTc1_Chr14 GTATATGAGTCAACCTTTACCATAACACTGGAATGCAATAAAGAGAATCCTAAGACATCTACAAGGGTTATCAACCTTTGGTCTTCATGTTAAACCTACTACACCCCTTGTCCATCTGTCTCTC
4. GR38_contig_001107 GTATATGAGTCAACCTTTACCATAACACTGGAATGCAATAAAGAGAATCCTAAGACATCTACAAGGGTTATCAACCTTTGGTCTTCATGTTAAACCTGCTACACCCCTTGTCCATCTGTCTCTC
5. PR36_LAP022b_PRFTc1F3
6. PR36_LAP022e_PRFTC1F3
7. PR42_LAP029b_PRFTC1F5
8. PR42_LAP030a_PRFTC1F5

4,720 4,730 4,740 4,750 4,760 4,770 4,780 4,790 4,800 4,810 4,820 4,830

Consensus TTAGCTTAATGTGATGCAGATTGWGCCTATGATCCAAATGATAGAAAATCTACCTCGAGGGCTTGTGTATATGTTGGACTAAACATTGTCACTTGGTGGTCCAAGAACAAACTATCTCTAGATC

Identity

1. LD37_contig_001630 TTAGCTTAATGTGATGCAGATTGTGCCTATGATCCAAATGATAGAAAATCTACCTCGAGGGCTTGTGTATATGTTGGACTAAACATTGTCACTTGGTGGTCCAAGAACAAACTATCTCTAGATC
2. P27174-4_contig_001786 TTAGCTTAATGTGATGCAGATTGTGCCTATGATCCAAATGATAGAAAATCTACCTCGAGGGCTTGTGTATATGTTGGACTAAACATTGTCACTTGGTGGTCCAAGAACAAACTATCTCTAGATC
3. LalbFTc1_Chr14 TTAGCTTAATGTGATGCAGATTGAGCCTATGATCCAAATGATAGAAAATCTACCTCGAGGGCTTGTGTATATGTTGGACTAAACATTGTCACTTGGTGGTCCAAGAACAAACTATCTCTAGATC
4. GR38_contig_001107 TTAGCTTAATGTGATGCAGATTGAGCCTATGATCCAAATGATAGAAAATCTACCTCGAGGGCTTGTGTATATGTTGGACTAAACATTGTCACTTGGTGGTCCAAGAACAAACTATCTCTAGATC
5. PR36_LAP022b_PRFTc1F3
6. PR36_LAP022e_PRFTC1F3
7. PR42_LAP029b_PRFTC1F5
8. PR42_LAP030a_PRFTC1F5

4,840 4,850 4,860 4,870 4,880 4,890 4,900 4,910 4,920 4,930 4,940 4,950 4,960

Consensus CAACACTCAACCTGAAATATAGGAGCTTGGAGTTGGATTCTCAATAAATAATTTGGATAGAGTCATTGCTTTCAGAACTTCAATTTGCTCATTAAGCTCCACTGATTTTGTGCGATAATGCAAG

Identity

1. LD37_contig_001630 CAACACTCAACCTGAAATATAGGAGCTTGGAGTTGGATTCTCAATAAATAATTTGGATAGAGTCATTGCTTTCAGAACTTCAATTTGCTCATTAAGCTCCACTGATTTTGTGCGATAATGCAAG
2. P27174-4_contig_001786 CAACACTCAACCTGAAATATAGGAGCTTGGAGTTGGATTCTCAATAAATAATTTGGATAGAGTCATTGCTTTCAGAACTTCAATTTGCTCATTAAGCTCCACTGATTTTGTGCGATAATGCAAG
3. LalbFTc1_Chr14 CAACACTCAACCTGAAATATTGGAGCTTGGAGTTGGATTCTCAATAAATAATTTGGATAGAGTCATTGCTTTCAGAACTTCAATTTGCTCATTAAGCTCCACTGATTTTGTGCGATAATGCAAG
4. GR38_contig_001107 CAACACTCAACCTGAAATATAGGAGCTTGGAGTTGGATTCTCAATAAATAATTTGGATAGAGTCATTGCTTTCAGAACTTCAATTTGCTCATTAAGCTCCACTGATTTTGTGCGATAATGCAAG
5. PR36_LAP022b_PRFTc1F3
6. PR36_LAP022e_PRFTC1F3
7. PR42_LAP029b_PRFTC1F5
8. PR42_LAP030a_PRFTC1F5

4,970 4,980 4,990 5,000 5,010 5,020 5,030 5,040 5,050 5,060 5,070 5,080

Consensus TATTGTTTCTAAGTTGAACTCTCTCAAAAAATGCCTCAAAGTTTTTTATCTTCTTTCTCTTAACATTTACCTCATGGGTAAATGGGGTGTATTAGAGAACAAAAGAGCATAGGGTGTATAGTTT

Identity

1. LD37_contig_001630 TATTGTTTCTAAGTTGAACTCTCTCAAAAAATGCCTCAAAGTTTTTTATCTTCTTTCTCTTAACATTTACCTCATGGGTAAATGGGGTGTATTAGAGAACAAAAGAGCATAGGGTGTATAGTTT
2. P27174-4_contig_001786 TATTGTTTCTAAGTTGAACTCTCTCAAAAAATGCCTCAAAGTTTTTTATCTTCTTTCTCTTAACATTTACCTCATGGGTAAATGGGGTGTATTAGAGAACAAAAGAGCATAGGGTGTATAGTTT
3. LalbFTc1_Chr14 TATTGTTTCTAAGTTGAACTCTCTCAAAAAATGCCTCAAAGTTTTTTATCTTCTTTCTCTTAACATTTACCTCATGGGTAAATGGGGTGTATTAGAGAACAAAAGAGCATAGGGTGTATAGTTT
4. GR38_contig_001107 TATTGTTTCTAAGTTGAACTCTCTCAAAAAATGCCTCAAAGTTTTTTATCTTCTTTCTCTTAACATTTACCTCATGGGTAAATGGGGTGTATTAGAGAACAAAAGAGCATAGGGTGTATAGTTT
5. PR36_LAP022b_PRFTc1F3
6. PR36_LAP022e_PRFTC1F3
7. PR42_LAP029b_PRFTC1F5
8. PR42_LAP030a_PRFTC1F5

5,090 5,100 5,110 5,120 5,130 5,140 5,150 5,160 5,170 5,180 5,190 5,200

Consensus CAAGAGGGTGTATTAGTGATTAGGGTTTACTTTAGAGCTCGTTATTGAAACTAACTAATAGCTTTCTTGAGAAGCTAACTCAACTACTATAAATAGAAGTGTGTTTTTCTCTATTGTAACTAAC

Identity

1. LD37_contig_001630 CAAGAGGGTGTATTAGTGATTAGGGTTTACTTTAGAGCTCGTTATTGAAACTAACTAATAGCTTTCTTGAGAAGCTAACTCAACTACTATAAATAGAAGTGTGTTTTTCTCTATTGTAACTAAC
2. P27174-4_contig_001786 CAAGAGGGTGTATTAGTGATTAGGGTTTACTTTAGAGCTCGTTATTGAAACTAACTAATAGCTTTCTTGAGAAGCTAACTCAACTACTATAAATAGAAGTGTGTTTTTCTCTATTGTAACTAAC
3. LalbFTc1_Chr14 CAAGAGGGTGTATTAGTGATTAGGGTTTACTTTAGAGCTCGTTATTGAAACTAACTAATAGCTTTCTTGAGAAGCTAACTCAACTACTATAAATAGAAGTGTGTTTTTCTCTATTGTAACTAAC
4. GR38_contig_001107 CAAGAGGGTGTATTAGTGATTAGGGTTTACTTTAGAGCTCGTTATTGAAACTAACTAATAGCTTTCTTGAGAAGCTAACTCAACTACTATAAATAGAAGTGTGTTTTTCTCTATTGTAACTAAC
5. PR36_LAP022b_PRFTc1F3
6. PR36_LAP022e_PRFTC1F3
7. PR42_LAP029b_PRFTC1F5
8. PR42_LAP030a_PRFTC1F5

5,210 5,220 5,230 5,240 5,250 5,260 5,270 5,280 5,290 5,300 5,310 5,320 5,330

Consensus TTTCATTTCTTTCAATAAGATTGAATCCCTCTCTCGTCTTTACCCTTCTCTCTTTTCTTCTTTTATTTCTTCAGTTCTTACACGCATCAAGAGTTGGTTTGACCAATACGAGTTTTTGACCTAT

Identity

1. LD37_contig_001630 TTTCATTTCTTTCAATAAGATTGAATCCCTCTCTCGTCTTTACCCTTCTCTCTTTTCTTCTTTTATTTCTTCAGTTCTTACACGCATCAAGAGTTGGTTTGACCAATACGAGTTTTTGACCTAT
2. P27174-4_contig_001786 TTTCATTTCTTTCAATAAGATTGAATCCCTCTCTCGTCTTTACCCTTCTCTCTTTTCTTCTTTTATTTCTTCAGTTCTTACACGCATCAAGAGTTGGTTTGACCAATACGAGTTTTTGACCTAT
3. LalbFTc1_Chr14 TTTCATTTCTTTCAATAAGATTGAATCCCTCTCTCGTCTTTACCCTTCTCTCTTTTCTTCTTTTATTTCTTCAGTTCTTACACGCATCAAGAGTTGGTTTGACCAATACGAGTTTTTGACCTAT
4. GR38_contig_001107 TTTCATTTCTTTCAATAAGATTGAATCCCTCTCTCGTCTTTACCCTTCTCTCTTTTCTTCTTTTATTTCTTCCGTTCTTACACGCATCTACAGTTGGTTTGACCAATACGAGTTTTTGACCTAT
5. PR36_LAP022b_PRFTc1F3
6. PR36_LAP022e_PRFTC1F3
7. PR42_LAP029b_PRFTC1F5
8. PR42_LAP030a_PRFTC1F5

5,340 5,350 5,360 5,370 5,380 5,390 5,400 5,410 5,420 5,430 5,440 5,450

Consensus GCATGGTATGTCAAACATTTGTTGCCTAATGAAAATGGGGATAGAATTTGCGTTTGGTTTTGTGATTGCTGAGGTTTATTATATGGGAATTTCTGGCACTCTGTTATTTGTCAAGTGAACTAGT

Identity

1. LD37_contig_001630 GCATGGTATGTCAAACATTTGTTGCCTAATGAAAATGGGGATAGAATTTGCGTTTGGTTTTGTGATTGCTGAGGTTTATTATATGGGAATTTCTGGCACTCTGTTATTTGTCAAGTGAACTAGT
2. P27174-4_contig_001786 GCATGGTATGTCAAACATTTGTTGCCTAATGAAAATGGGGATAGAATTTGCGTTTGGTTTTGTGATTGCTGAGGTTTATTATATGGGAATTTCTGGCACTCTGTTATTTGTCAAGTGAACTAGT
3. LalbFTc1_Chr14 GCATGGTATGTCAAACATTTGTTGCCTAATGAAAATGGGGATAGAATTTGCGTTTGGTTTTGTGATTGCTGAGGTTTATTATATGGGAATTTCTGGCACTCTGTTATTTGTCAAGTGAACTAGT
4. GR38_contig_001107 GCATGGTATGTCAAACATTTGTTGCCTAATGAAAATGGGGATAGAATTTGCGTTTGGTTTTGTGATTGCTGAGGTTTATTATATGGGAATTTCTGGCACTCTGTTATTTGTCAAGTGAACTAGT
5. PR36_LAP022b_PRFTc1F3
6. PR36_LAP022e_PRFTC1F3
7. PR42_LAP029b_PRFTC1F5
8. PR42_LAP030a_PRFTC1F5

5,460 5,470 5,480 5,490 5,500 5,510 5,520 5,530 5,540 5,550 5,560 5,570 5,580

Consensus TTATTAATACTTGAAAACCGTAGAAGCTGTGTTTTTTACTATTGTCCCTTCTGTTTTATGAAGAAGTTATGGCTGGGTACTGCTGAAGAGTCAAGTGAACTTCAATTGTTTCTGTGTTGTTTTT

Identity

1. LD37_contig_001630 TTATTAATACTTGAAAACCGTAGAAGCTGTGTTTTTTACTATTGTCCCTTCTGTTTTATGAAGAAGTTATGGCTGGGTACTGCTGAAGAGTCAAGTGAACTTCAATTGTTTCTGTGTTGTTTTT
2. P27174-4_contig_001786 TTATTAATACTTGAAAACCGTAGAAGCTGTGTTTTTTACTATTGTCCCTTCTGTTTTATGAAGAAGTTATGGCTGGGTACTGCTGAAGAGTCAAGTGAACTTCAATTGTTTCTGTGTTGTTTTT
3. LalbFTc1_Chr14 TTATTAATACTTGAAAACCGTAGAAGCTGTGTTTTTTACTATTGTCCCTTCTGTTTTATGAAGAAGTTATGGCTGGGTACTGCTGAAGAGTCAAGTGAACTTCAATTGTTTCTGTGTTGTTTTT
4. GR38_contig_001107 TTATTAATACTTGAAAACCGTAGAAGCTGTGTTTTTTACTATTGTCCCTTCTGTTTTATGAAGAAGTTATGGCTGGGTACTGCTGAAGAGTCAAGTGAACTTCAATTGTTTCTGTGTTGTTTTT
5. PR36_LAP022b_PRFTc1F3
6. PR36_LAP022e_PRFTC1F3
7. PR42_LAP029b_PRFTC1F5
8. PR42_LAP030a_PRFTC1F5

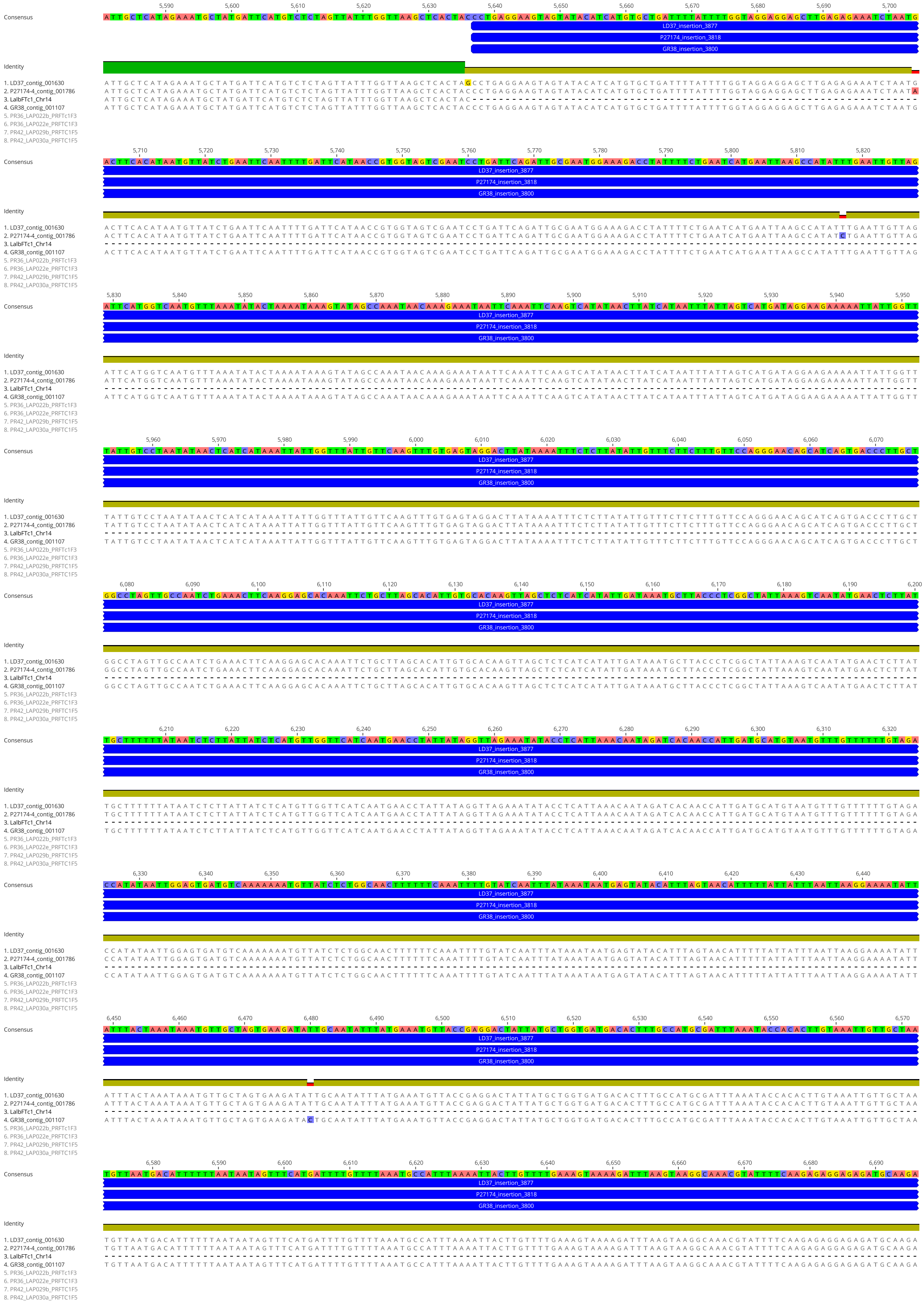
Consensus
LD37_insertion_3877
P27174_insertion_3818
GR38_insertion_3800
Identity
1. LD37_contig_001630
2. P27174-4_contig_001786
3. LalbFTc1_Chr14
4. GR38_contig_001107
5. PR36_LAP022b_PRFTc1F3
6. PR36_LAP022e_PRFTC1F3
7. PR42_LAP029b_PRFTC1F5
8. PR42_LAP030a_PRFTC1F5

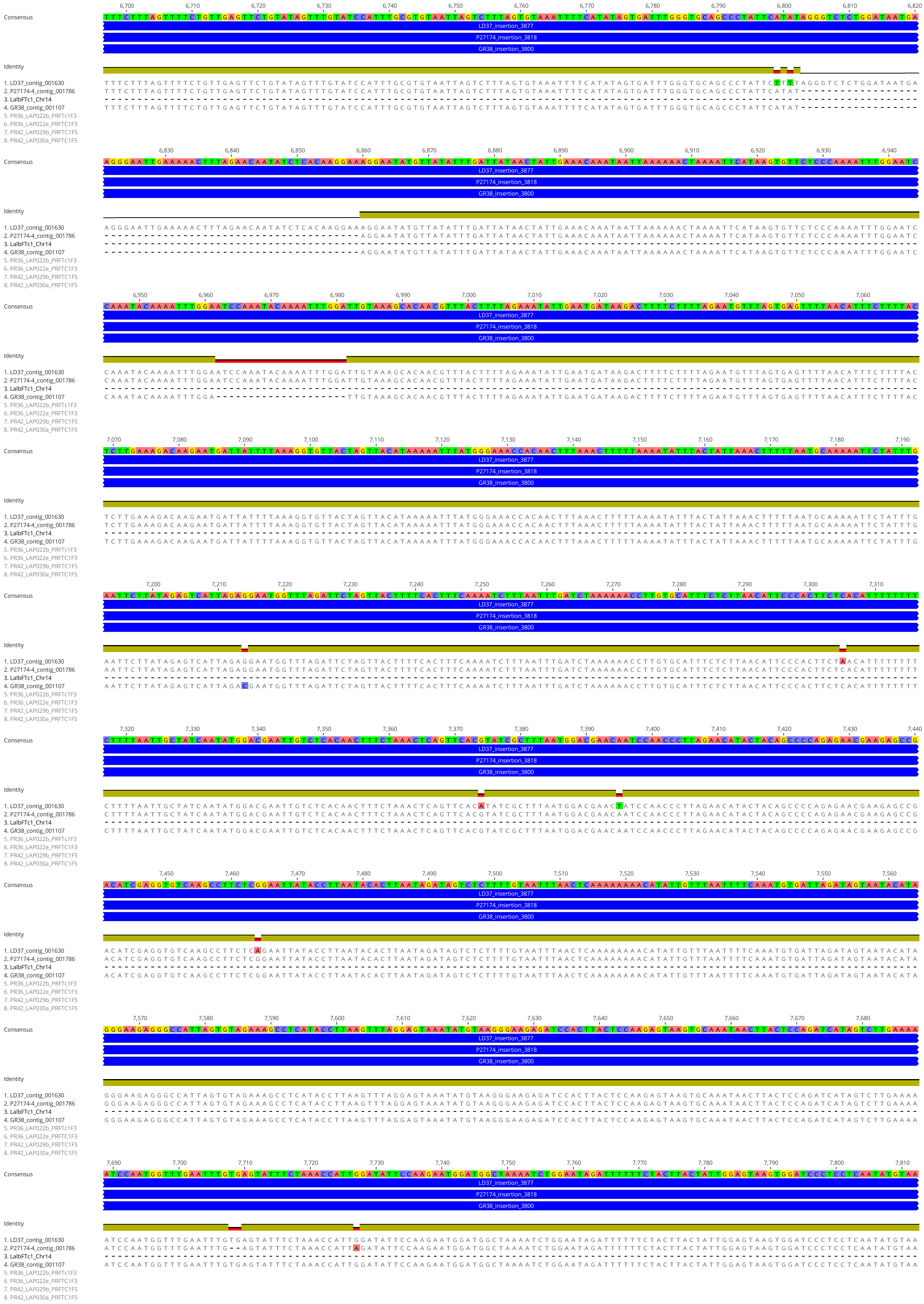
Consensus
LD37_insertion_3877
P27174_insertion_3818
GR38_insertion_3800
Identity
1. LD37_contig_001630
2. P27174-4_contig_001786
3. LalbFTc1_Chr14
4. GR38_contig_001107
5. PR36_LAP022b_PRFTc1F3
6. PR36_LAP022e_PRFTC1F3
7. PR42_LAP029b_PRFTC1F5
8. PR42_LAP030a_PRFTC1F5

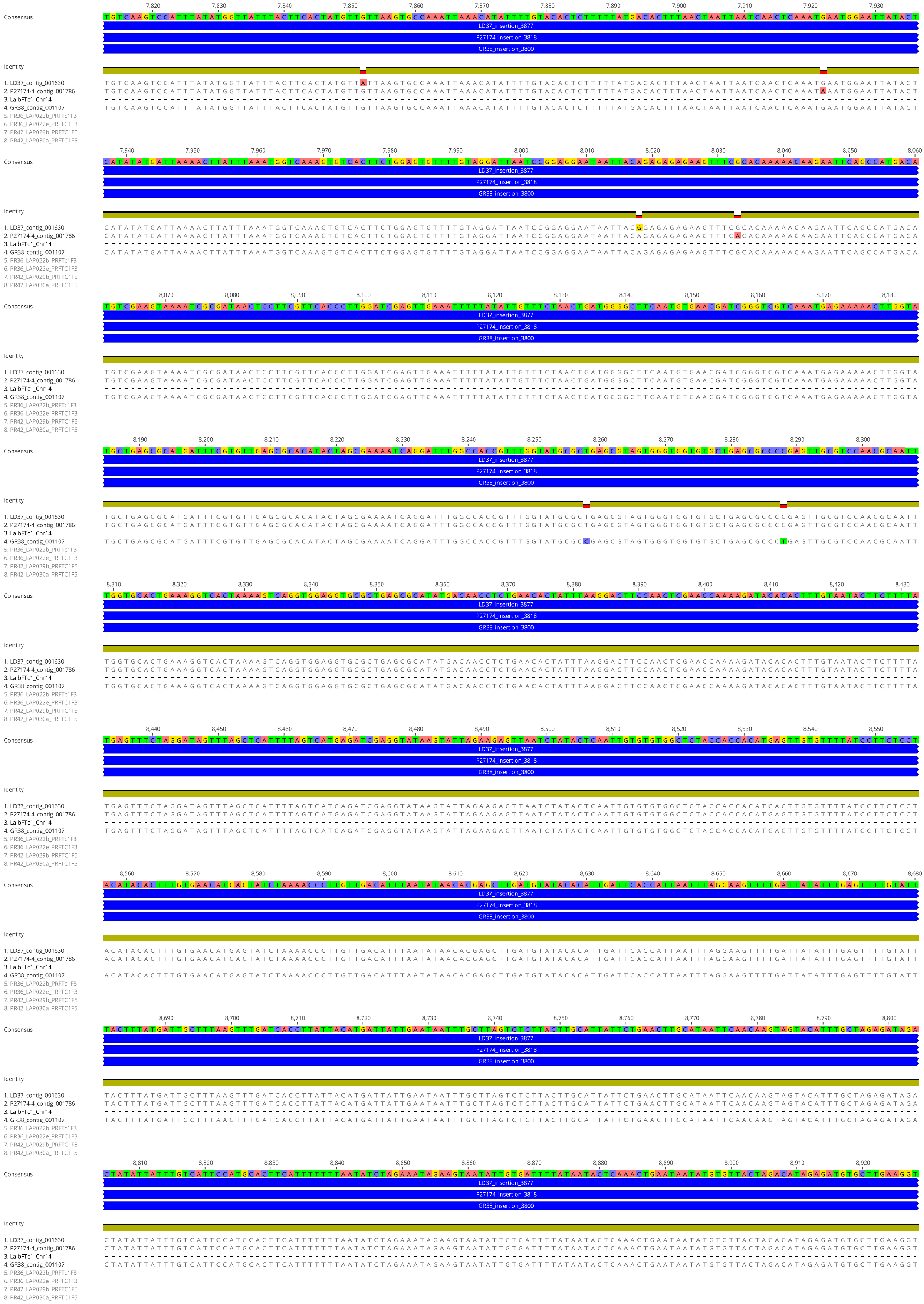
Consensus
LD37_insertion_3877
P27174_insertion_3818
GR38_insertion_3800
Identity
1. LD37_contig_001630
2. P27174-4_contig_001786
3. LalbFTc1_Chr14
4. GR38_contig_001107
5. PR36_LAP022b_PRFTc1F3
6. PR36_LAP022e_PRFTC1F3
7. PR42_LAP029b_PRFTC1F5
8. PR42_LAP030a_PRFTC1F5

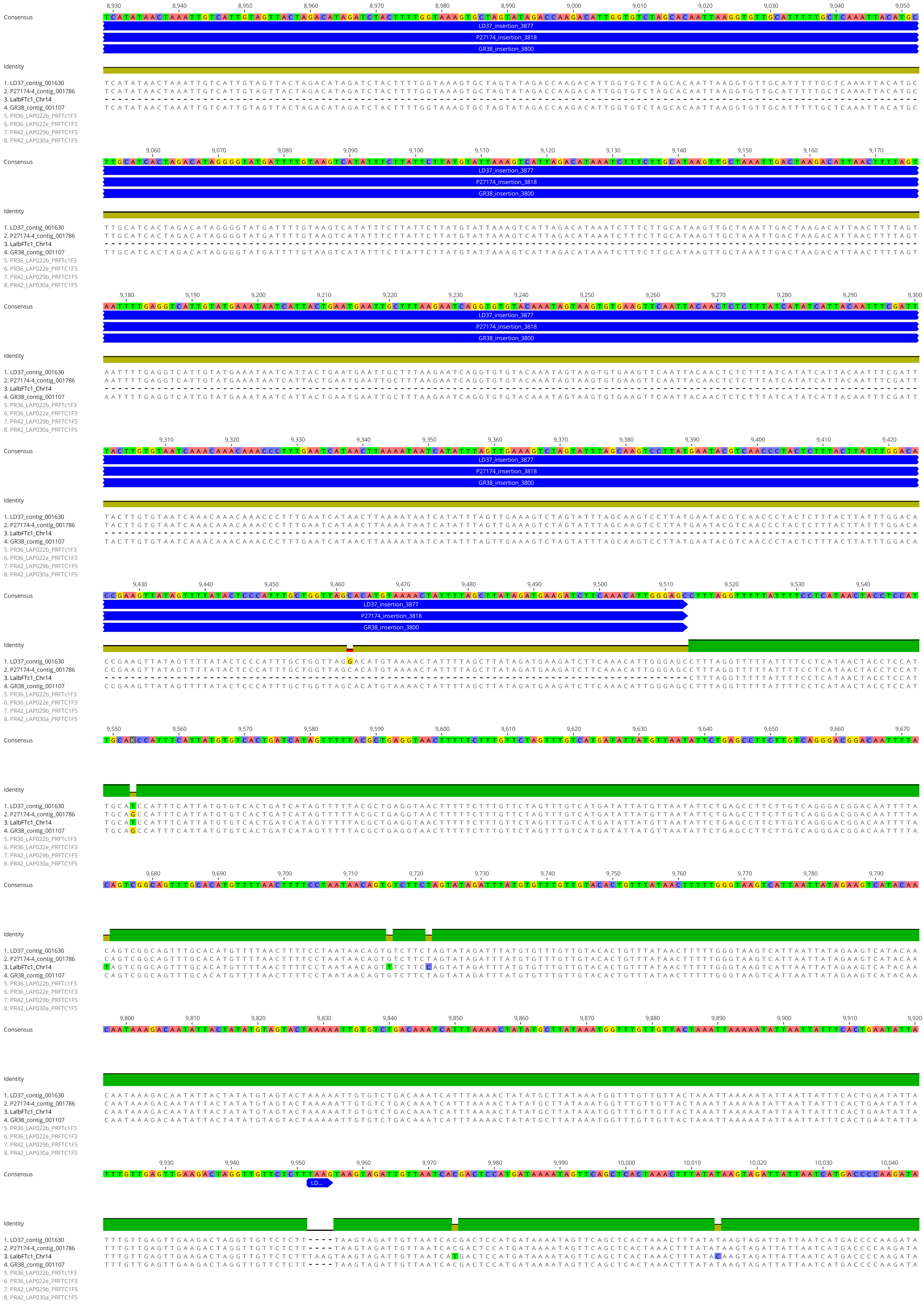
Consensus
Identity
LD37_insertion_3877
P27174_insertion_3818
GR38_insertion_3800
1. LD37_contig_001630
2. P27174-4_contig_001786
3. LalbFTc1_Chr14
4. GR38_contig_001107
5. PR36_LAP022b_PRFTc1F3
6. PR36_LAP022e_PRFTC1F3
7. PR42_LAP029b_PRFTC1F5
8. PR42_LAP030a_PRFTC1F5
LD...

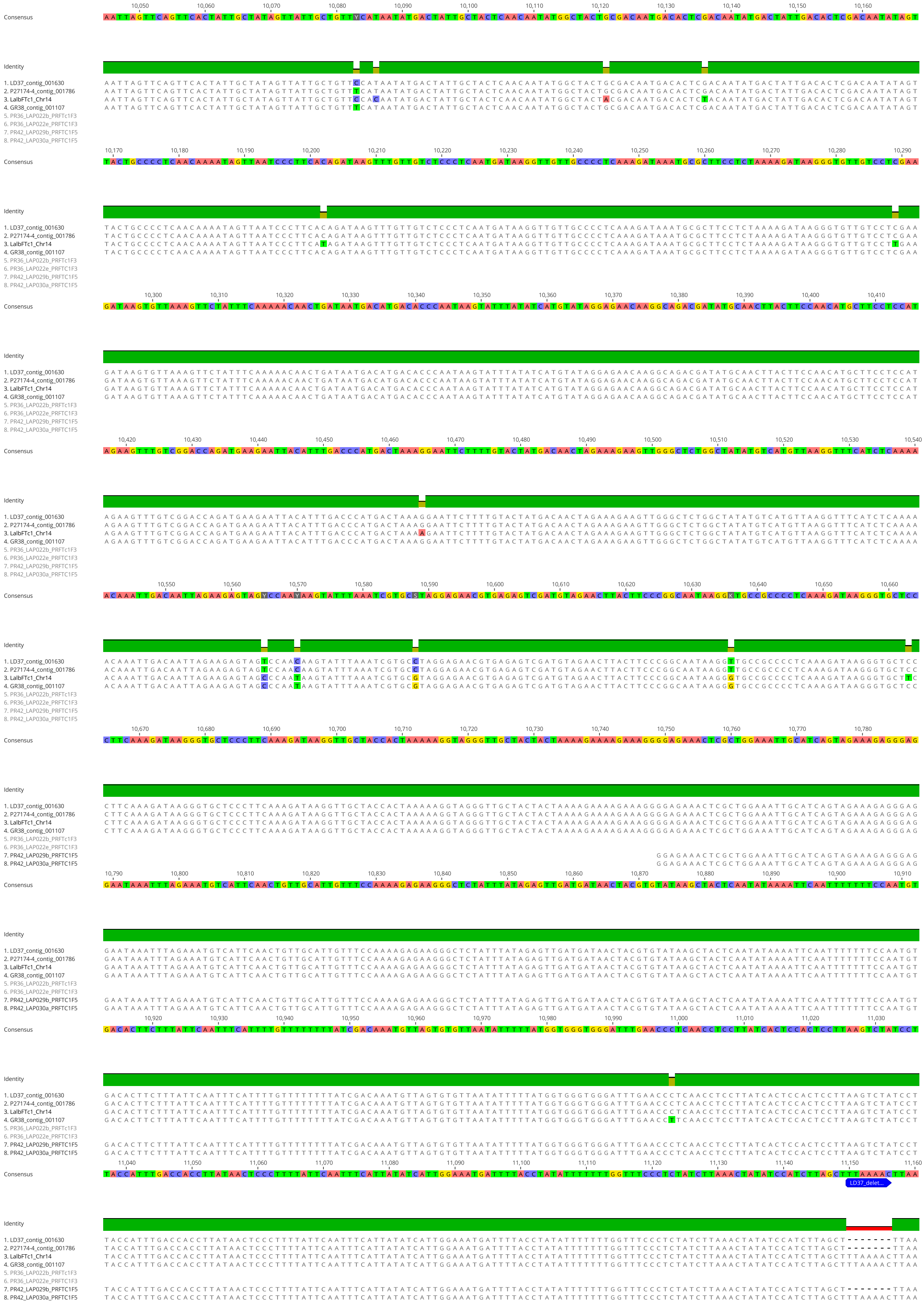
Consensus
Identity
1. LD37_contig_001630
2. P27174-4_contig_001786
3. LalbFTc1_Chr14
4. GR38_contig_001107
5. PR36_LAP022b_PRFTc1F3
6. PR36_LAP022e_PRFTC1F3
7. PR42_LAP029b_PRFTC1F5
8. PR42_LAP030a_PRFTC1F5
10,050 10,060 10,070 10,080 10,090 10,100 10,110 10,120 10,130 10,140 10,150 10,160
10,170 10,180 10,190 10,200 10,210 10,220 10,230 10,240 10,250 10,260 10,270 10,280 10,290
10,300 10,310 10,320 10,330 10,340 10,350 10,360 10,370 10,380 10,390 10,400 10,410
10,420 10,430 10,440 10,450 10,460 10,470 10,480 10,490 10,500 10,510 10,520 10,530 10,540
10,550 10,560 10,570 10,580 10,590 10,600 10,610 10,620 10,630 10,640 10,650 10,660
10,670 10,680 10,690 10,700 10,710 10,720 10,730 10,740 10,750 10,760 10,770 10,780
10,790 10,800 10,810 10,820 10,830 10,840 10,850 10,860 10,870 10,880 10,890 10,900 10,910
10,920 10,930 10,940 10,950 10,960 10,970 10,980 10,990 11,000 11,010 11,020 11,030
11,040 11,050 11,060 11,070 11,080 11,090 11,100 11,110 11,120 11,130 11,140 11,150 11,160
LD37_delet...

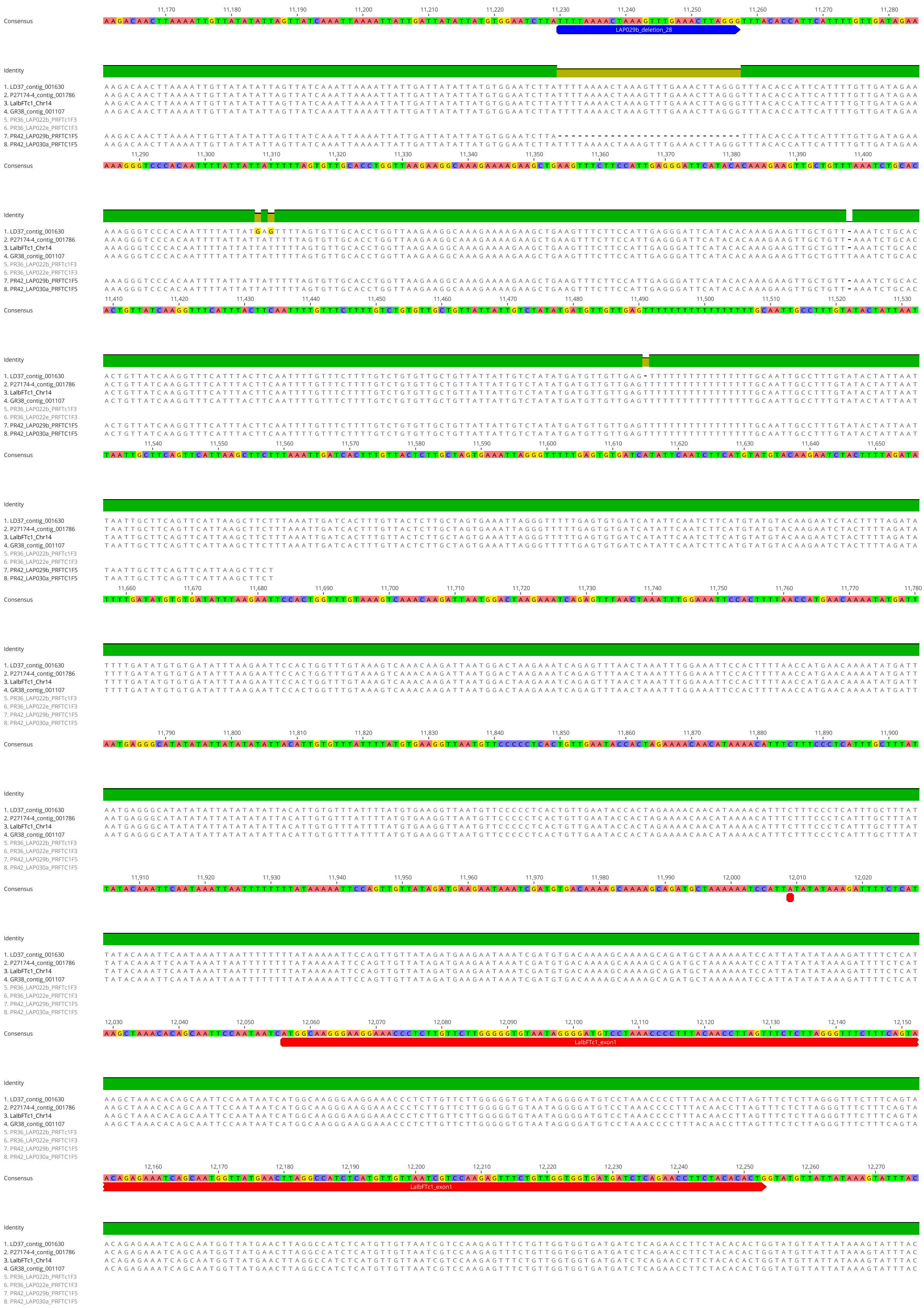
Consensus
Identity
1. LD37_contig_001630
2. P27174-4_contig_001786
3. LalbFTc1_Chr14
4. GR38_contig_001107
5. PR36_LAP022b_PRFTc1F3
6. PR36_LAP022e_PRFTC1F3
7. PR42_LAP029b_PRFTC1F5
8. PR42_LAP030a_PRFTC1F5
LAP029b_deletion_28
LalbFTc1_exon1

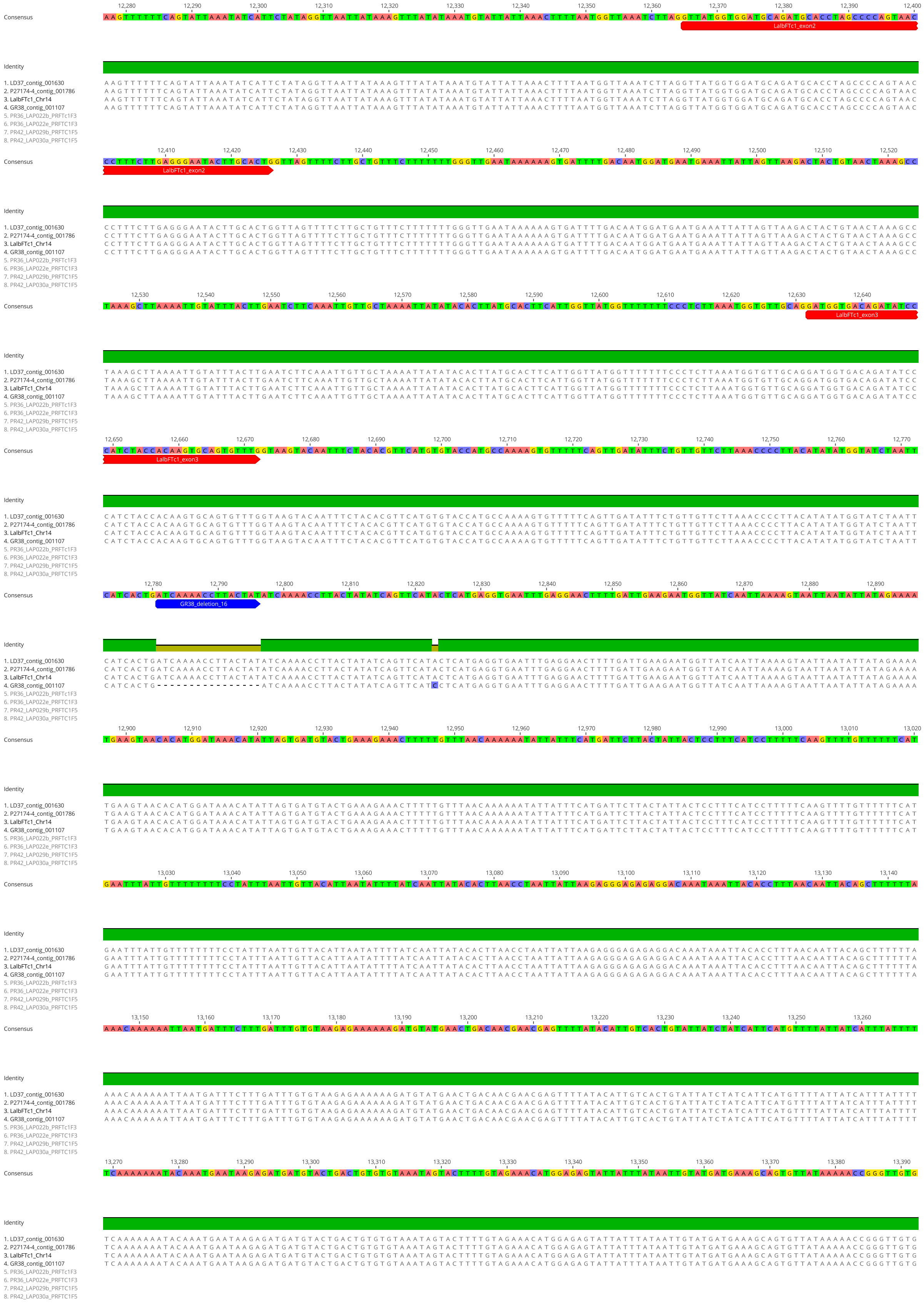
Consensus
Identity
1. LD37_contig_001630
2. P27174-4_contig_001786
3. LalbFTc1_Chr14
4. GR38_contig_001107
5. PR36_LAP022b_PRFTc1F3
6. PR36_LAP022e_PRFTc1F3
7. PR42_LAP029b_PRFTc1F5
8. PR42_LAP030a_PRFTc1F5
LalbFTc1_exon2
LalbFTc1_exon3
GR38_deletion_16

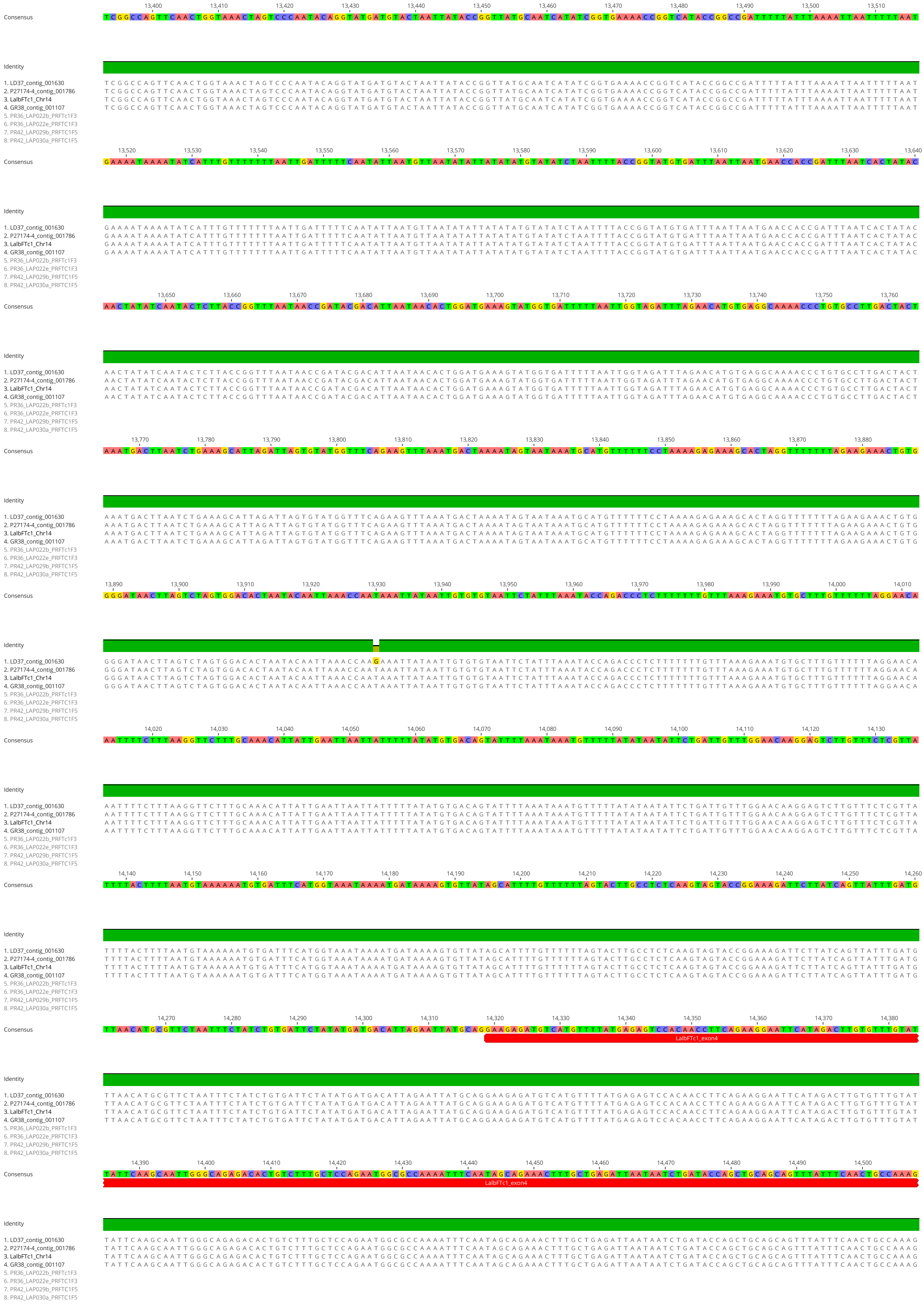
Consensus
Identity
1. LD37_contig_001630
2. P27174-4_contig_001786
3. LalbFTc1_Chr14
4. GR38_contig_001107
5. PR36_LAP022b_PRFTc1F3
6. PR36_LAP022e_PRFTC1F3
7. PR42_LAP029b_PRFTC1F5
8. PR42_LAP030a_PRFTC1F5
LalbFTc1_exon4

14,510 14,520 14,530 14,540 14,550 14,560 14,570 14,580 14,590 14,600 14,610 14,620 14,630

Consensus AGAGCGTGGTTGTGGTGGAAGGAGAACAGAGTAGAAACTACAAGTTTCCATTAAATAAATATGCACTCTCTTATTTAATAAATAAAAGTTTAAAGAGAAGGAAGGTTACCCCAGGTTAGGGCAT

LalbFTc1_exon4

Identity

1. LD37_contig_001630 AGAGCGTGGTTGTGGTGGAAGGAGAACAGAGTAGAAACTACAAGTTTCCATTAAATAAATATGCACTCTCTTATTTAATAAATAAAAGTTTAAAGAGAAGGAAGGTTACCCCAGGTTAGGGCAT
2. P27174-4_contig_001786 AGAGCGTGGTTGTGGTGGAAGGAGAACAGAGTAGAAACTACAAGTTTCCATTAAATAAATATGCACTCTCTTATTTAATAAATAAAAGTTTAAAGAGAAGGAAGGTTACCCCAGGTTAGGGCAT
3. LalbFTc1_Chr14 AGAGCGTGGTTGTGGTGGAAGGAGAACAGAGTAGAAACTACAAGTTTCCATTAAATAAATATGCACTCTCTTATTTAATAAATAAAAGTTTAAAGAGAAGGAAGGTTACCCCAGGTTAGGGCAT
4. GR38_contig_001107 AGAGCGTGGTTGTGGTGGAAGGAGAACAGAGTAGAAACTACAAGTTTCCATTAAATAAATATGCACTCTCTTATTTAATAAATAAAAGTTTAAAGAGAAGGAAGGTTACCCCAGGTTAGGGCAT
5. PR36_LAP022b_PRFTc1F3
6. PR36_LAP022e_PRFTC1F3
7. PR42_LAP029b_PRFTC1F5
8. PR42_LAP030a_PRFTC1F5

14,640 14,650 14,660 14,670 14,680 14,690 14,700 14,708

Consensus GTTATTCACAAACTACATCAAAGTAGGGCTTATATATCTTCCTACCAATACTCTTTCTTATATTGAAGAAACTTTC

Identity

1. LD37_contig_001630 GTTATTCACAAACTACATCAAAGTAGGGCTTATATATCTTCCTACCAATACTCTTTCTTATATTGAAGAAACTTTC
2. P27174-4_contig_001786 GTTATTCACAAACTACATCAAAGTAGGGCTTATATATCTTCCTACCAATACTCTTTCTTATATTGAAGAAACTTTC
3. LalbFTc1_Chr14 GTTATTCACAAACTACATCAAAGTAGGGCTTATATATCTTCCTACCAATACTCTTTCTTATATTGAAGAAACTTTC
4. GR38_contig_001107 GTTATTCACAAACTACATCAAAGTAGGGCTTATATATCTTCCTACCAATACTCTTTCTTATATTGAAGAAACTTTC
5. PR36_LAP022b_PRFTc1F3
6. PR36_LAP022e_PRFTC1F3
7. PR42_LAP029b_PRFTC1F5
8. PR42_LAP030a_PRFTC1F5

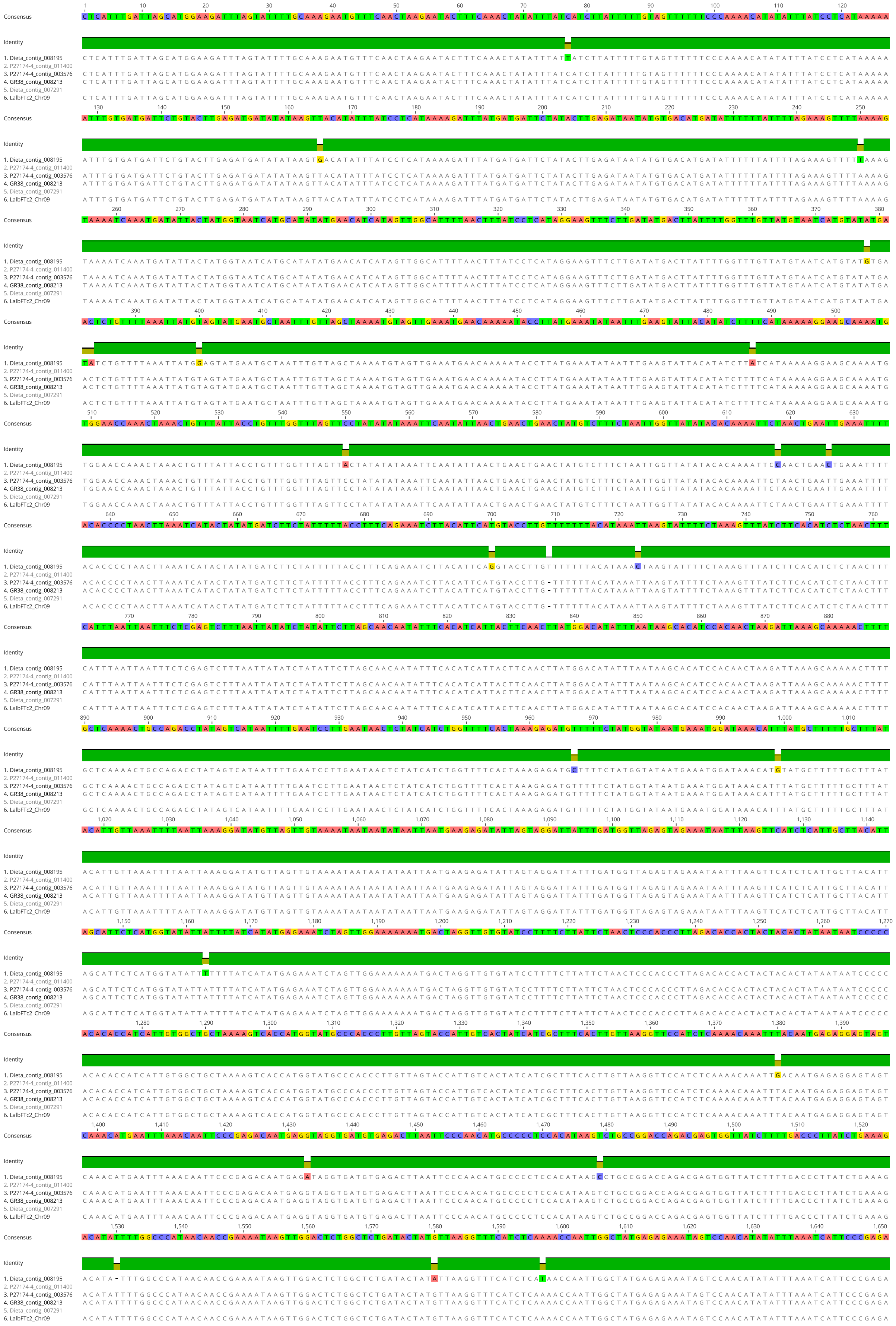
Consensus
Identity
1. Dieta_contig_008195
2. P27174-4_contig_011400
3. P27174-4_contig_003576
4. GR38_contig_008213
5. Dieta_contig_007291
6. LalbFTc2_Chr09

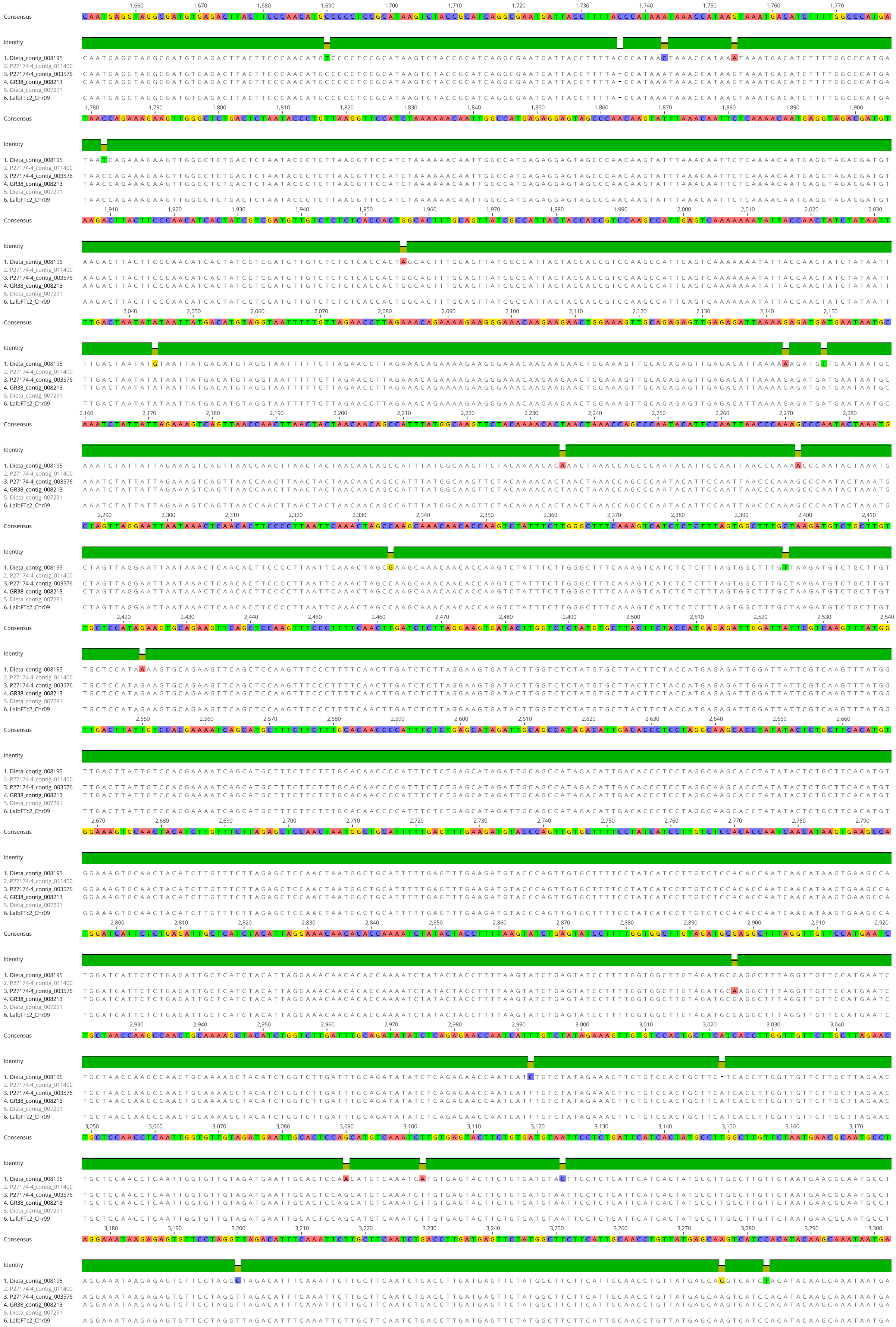
Consensus
Identity
1. Dieta_contig_008195
2. P27174-4_contig_011400
3. P27174-4_contig_003576
4. GR38_contig_008213
5. Dieta_contig_007291
6. LalbFTc2_Chr09

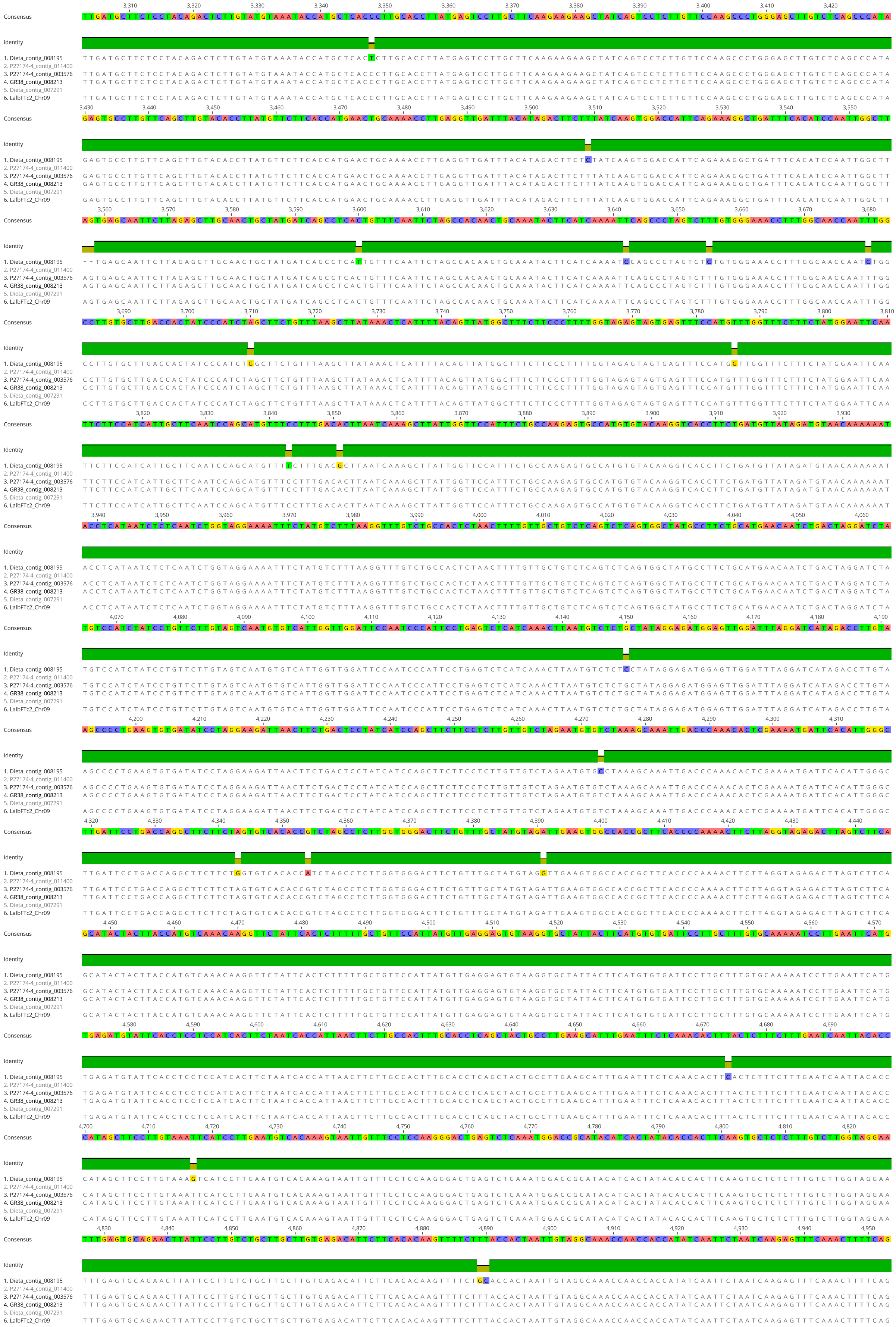
Consensus
Identity
1. Dieta_contig_008195
2. P27174-4_contig_011400
3. P27174-4_contig_003576
4. GR38_contig_008213
5. Dieta_contig_007291
6. LalbFTc2_Chr09
3,310
4,950

```
        4,960 4,970 4,980 4,990 5,000 5,010 5,020 5,030 5,040 5,050 5,060 5,070 5,080
Consensus                    ATTCAAATGACCAAATCTCTGATGCCAAATCCTTGTGTCATCTGCTACTGTTTTAAGGCATGTATGATTTTCTAAGTGAATTCCAATCTTGAAGGTCTTATTGTTTGAGAGAGGTGCCTTCAAGATT
Identity
1. Dieta_contig_008195       ATTCAAATGACCAAATCTCTGATGCCAAATCCTTGTGTCATCTGCTACTGTTTTAAGGCATGTATGATTTTCTAAGTGAATTCCAATCTTGAAGGTCTTATTGTTTGAGAGAGGTGCCTTCAAGATT
2. P27174-4_contig_011400
3. P27174-4_contig_003576    ATTCAAATGACCAAATCTCTGATGCCAAATCCTTGTGTCATCTGCTACTGTTTTAAGGCATGTATGATTTTCTAAGTGAATTCCAATCTTGAAGGTCTTATTGTTTGAGAGAGGTGCCTTCAAGATT
4. GR38_contig_008213         ATTCAAATGACCAAATCTCTGATGCCAAATCCTTGTGTCATCTGCTACTGTTTTAAGGCATGTATGATTTTCTAAGTGAATTCCAATCTTGAAGGTCTTATTGTTTGAGAGAGGTGCCTTCAAGATT
5. Dieta_contig_007291
6. LalbFTc2_Chr09             ATTCAAATGACCAAATCTCTGATGCCAAATCCTTGTGTCATCTGCTACTGTTTTAAGGCATGTATGATTTTCTAAGTGAATTCCAATCTTGAAGGTCTTATTGTTTGAGAGAGGTGCCTTCAAGATT
```

```
        5,090 5,100 5,110 5,120 5,130 5,140 5,150 5,160 5,170 5,180 5,190 5,200
Consensus                    AGTCCTCCACTTTTGTTAAAGATTTTCAACATTTTGTCTTCCATATTGATATTGTATCCCATCTCTAAGAGCTGTCCAAGACTGATCAGATTGTTCTTCATTCTTGGCACATACAACACATTGGAGA
Identity
1. Dieta_contig_008195       AGTCCTCCACTTTTGTTAAAGATTTTCAACATTTTGTCTTCCATATTGATATTGTATCCCATCTCTAAGAGCTGTCCAAGACTGATCAGATTGTTCTTCATTCTTGGCACATACAACACATTGGAGA
2. P27174-4_contig_011400
3. P27174-4_contig_003576    AGTCCTCCACTTTTGTTAAAGATTTTCAACATTTTGTCTTCCATATTGATATTGTATCCCATCTCTAAGAGCTGTCCAAGACTGATCAGATTGTTCTTCATTCTTGGCACATACAACACATTGGAGA
4. GR38_contig_008213         AGTCCTCCACTTTTGTTAAAGATTTTCAACATTTTGTCTTCCATATTGATATTGTATCCCATCTCTAAGAGCTGTCCAAGACTGATCAGATTGTTCTTCATTCTTGGCACATACAACACATTGGAGA
5. Dieta_contig_007291
6. LalbFTc2_Chr09             AGTCCTCCACTTTTGTTAAAGATTTTCAACATTTTGTCTTCCATATTGATATTGTATCCCATCTCTAAGAGCTGTCCAAGACTGATCAGATTGTTCTTCATTCTTGGCACATACAACACATTGGAGA
```

```
        5,210 5,220 5,230 5,240 5,250 5,260 5,270 5,280 5,290 5,300 5,310 5,320 5,330
Consensus                    AGCTTGACTTTGTACCATCTTTCTTCTTGATTTGGATTCTGCCTAGCCCTTCTGCACAAATGAAGCTATCATCTGCAAACTTGACCTTAGTTTTCACTGATTCATCAAGAAAGTTGAACCACTCCTT
Identity
1. Dieta_contig_008195       AGCTTGACTTTGTACCATCTTTCTTCTTGATTTGGATTCTGCCTAGCCCTTCTGCACAAATGAAGCTATCATCTGCAAACTTGACCTTAGTTTTCACTGATTCATCAAGAAAGTTGAACCACTCCTT
2. P27174-4_contig_011400
3. P27174-4_contig_003576    AGCTTGACTTTGTACCATCTTTCTTCTTGATTTGGATTCTGCCTAGCCCTTCTGCACAAATGAAGCTATCATCTGCAAACTTGACCTTAGTTTTCACTGATTCATCAAGAAAGTTGAACCACTCCTT
4. GR38_contig_008213         AGCTTGACTTTGTACCATCTTTCTTCTTGATTTGGATTCTGCCTAGCCCTTCTGCACAAATGAAGCTATCATCTGCAAACTTGACCTTAGTTTTCACTGATTCATCAAGAAAGTTGAACCACTCCTT
5. Dieta_contig_007291
6. LalbFTc2_Chr09             AGCTTGACTTTGTACCATCTTTCTTCTTGATTTGGATTCTGCCTAGCCCTTCTGCACAAATGAAGCTATCATCTGCAAACTTGACCTTAGTTTTCACTGATTCATCAAGAAAGTTGAACCACTCCTT
```

```
        5,340 5,350 5,360 5,370 5,380 5,390 5,400 5,410 5,420 5,430 5,440 5,450 5,460
Consensus                    ATGTGCAGTCATGTGATTGGAACACCCTGTATCAAGATACCAACTCTCAGATTCTTGTTGTTTTATAGGCATTGTTGTGACCATCAACACTACATCACTTGTTTCTTGCACTTCTTGTTGTGAGCAC
Identity
1. Dieta_contig_008195       ATGTCCAGTCATGTGATTGGAACACCCTGTATCAAGATACCAACTCTCAGATTCTTGTTGTTTTATAGGCATTGTTGTGACCATCAACACTACATCACTTGTTTCTTGCACTTCTTGTTGTGAGCAC
2. P27174-4_contig_011400
3. P27174-4_contig_003576    ATGTGCAGTCATGTGATTGGAACACCCTGTATCAAGATACCAACTCTCAGATTCTTGTTGTTTTATAGGCATTGTTGTGACCATCAACACTACATCACTTGTTTCTTGCACTTCTTGTTGTGAGCAC
4. GR38_contig_008213         ATGTGCAGTCATGTGATTGGAACACCCTGTATCAAGATACCAACTCTCAGATTCTTGTTGTTTTATAGGCATTGTTGTGACCATCAACACTACATCACTTGTTTCTTGCACTTCTTGTTGTGAGCAC
5. Dieta_contig_007291
6. LalbFTc2_Chr09             ATGTGCAGTCATGTGATTGGAACACCCTGTATCAAGATACCAACTCTCAGATTCTTGTTGTTTTATAGGCATTGTTGTGACCATCAACACTACATCACTTGTTTCTTGCACTTCTTGTTGTGAGCAC
```

```
        5,470 5,480 5,490 5,500 5,510 5,520 5,530 5,540 5,550 5,560 5,570 5,580
Consensus                    ACCTCAATGGTGTCACTTTCGCTTGAGACCATTTCCTCAGCCAACAACAAAACTTCTTCTGAATCTTCTTCTTTGGCCAACCTTGCCTCAGCCTCTTTGTACTTTATTTCAATGTGCACTCAGAAG
Identity
1. Dieta_contig_008195       ACCTCAATGGTGTCACTTTCGCTTGAGACCATTTCCTCAGCCAACAACAAAACTTCTTCTGAATCTTCTTCTTTGGCCAACCTTGCCTCAGCCTCTTTGTACTTTATTTCAATGTGCACTCAGAAG
2. P27174-4_contig_011400
3. P27174-4_contig_003576    ACCTCAATGGTGTCACTTTCGCTTGAGACCATTTCCTCAGCCAACAACAAAACTTCTTCTGAATCTTCTTCTTTGGCCAACCTTGCCTCAGCCTCTTTGTACTTTATTTCAATGTGCACTCAGAAG
4. GR38_contig_008213         ACCTCAATGGTGTCACTTTCGCTTGAGACCATTTCCTCAGCCAACAACAAAACTTCTTCTGAATCTTCTTCTTTGGCCAACCTTGCCTCAGCCTCTTTGTACTTTATTTCAATGTGCACTCAGAAG
5. Dieta_contig_007291
6. LalbFTc2_Chr09             ACCTCAATGGTGTCACTTTCGCTTGAGACCATTTCCTCAGCCAACAACAAAACTTCTTCTGAATCTTCTTCTTTGGCCAACCTTGCCTCAGCCTCTTTGTACTTTATTTCAATGTGCACTCAGAAG
```

```
        5,590 5,600 5,610 5,620 5,630 5,640 5,650 5,660 5,670 5,680 5,690 5,700 5,710
Consensus                    CATAGTGACCCCAATTCTTACAAGCAAAGCACTAAATCTTGCTCTCATCTTTCTTCTTTTTCCCACCAAGATTGCTTCTGTTCTTGGTGTATTTTCGGTCTGCATCAAGCCTTTGGTGGCTAGACTC
Identity
1. Dieta_contig_008195       CATAGTGACCCCAATTCTTACAAGCAAAACACTGAATCTTGCTCTCATCTTTCTTCTTTTTCCCACCAAGATTGCTTCTGTTCTTGGTGTATTTTCGGTCTGCATCAAGCCTTTGGTGGCTAGACTC
2. P27174-4_contig_011400
3. P27174-4_contig_003576    CATAGTGACCCCAATTCTTACAAGCAAAGCACTAAATCTTGCTCTCATCTTTCTTCTTTTTCCCACCAAGATTGCTTCTGTTCTTGGTGTATTTTCGGTCTGCATCAAGCCTTTGGTGGCTAGACTC
4. GR38_contig_008213         CATAGTGACCCCAATTCTTACAAGCAAAGCACTAAATCTTGCTCTCATCTTTCTTCTTTTTCCCACCAAGATTGCTTCTGTTCTTGGTGTATTTTCGGTCTGCATCAAGCCTTTGGTGGCTAGACTC
5. Dieta_contig_007291
6. LalbFTc2_Chr09             CATAGTGACCCCAATTCTTACAAGCAAAGCACTAAATCTTGCTCTCATCTTTCTTCTTTTTCCCACCAAGATTGCTTCTGTTCTTGGTGTATTTTCGGTCTGCATCAAGCCTTTGGTGGCTAGACTC
```

```
        5,720 5,730 5,740 5,750 5,760 5,770 5,780 5,790 5,800 5,810 5,820 5,830 5,840
Consensus                    AGGTCTTGATCTTTCTAAGTCATTAGACTTCTTGCCTTCAAGCCACTTATCATGAGACTTGTTGTTTTCCACCTGATCCAAGAAGCCATGCTTCTTCTTGAATTTAGCTATCAAAGCTTGCTAAGAT
Identity
1. Dieta_contig_008195       AGGTCTTGATTTTTCTGAGTCATTAGACTTCTTGCCTTAAAGCCACTTATCATGAGACTTGTTGTTTTCCACCTGATCCAAGAAGCCATGCTTCTTCTTGAATTTAGCTATCAAAGCTTGCTAAGAT
2. P27174-4_contig_011400
3. P27174-4_contig_003576    AGGTCTTGATCTTTCTAAGTCATTAGACTTCTTGCCTTCAAGCCACTTATCATGAGACTTGTTGTTTTCCACCTGATCCAAGAAGCCATGCTTCTTCTTGAATTTAGCTATCAAAGCTTGCTAAGAT
4. GR38_contig_008213         AGGTCTTGATCTTTCTAAGTCATTAGACTTCTTGCCTTCAAGCCACTTATCATGAGACTTGTTGTTTTCCACCTGATCCAAGAAGCCATGCTTCTTCTTGAATTTAGCTATCAAAGCTTGCTAAGAT
5. Dieta_contig_007291
6. LalbFTc2_Chr09             AGGTCTTGATCTTTCTAAGTCATTAGACTTCTTGCCTTCAAGCCACTTATCATGAGACTTGTTGTTTTCCACCTGATCCAAGAAGCCATGCTTCTTCTTGAATTTAGCTATCAAAGCTTGCTAAGAT
```

```
        5,850 5,860 5,870 5,880 5,890 5,900 5,910 5,920 5,930 5,940 5,950 5,960
Consensus                    TGTCTGCCTGCAACTCTCTCAAGCAATCTCTGCTCATGTGCCTCCAATGAGCCTTGCAATTCATCAAGCCTCAAGTTCTCTAAGTCCTTTGACTCCTCAATTGCAACAATCACATGGCCAAATCTAT
Identity
1. Dieta_contig_008195       TGTCTGCCTGCAACTCTCTCAAGCAATCTCTGCTCATGTGCCTCCAATGAGCCTTGCAATTCATCAAGCCTCAAGTTCTCTAAGTCCTTTGACTCCTCAATTGCAACAATCACATGGCCAAATCTAT
2. P27174-4_contig_011400
3. P27174-4_contig_003576    TGTCTGCCTGCAACTCTCTCAAGCAATCTCTGCTCATGTGCCTCCAATGAGCCTTGCAATTCATCAAGCCTCAAGTTCTCTAAGTCCTTTGACTCCTCAATTGCAACAATCACATGGCCAAATCTAT
4. GR38_contig_008213         TGTCTGCCTGCAACTCTCTCAAGCAATCTCTGCTCATGTGCCTCCAATGAGCCTTGCAATTCATCAAGCCTCAAGTTCTCTAAGTCCTTTGACTCCTCAATTGCAACAATCACATGGCCAAATCTAT
5. Dieta_contig_007291
6. LalbFTc2_Chr09             TGTCTGCCTGCAACTCTCTCAAGCAATCTCTGCTCATGTGCCTCCAATGAGCCTTGCAATTCATCAAGCCTCAAGTTCTCTAAGTCCTTTGACTCCTCAATTGCAACAATCACATGGCCAAATCTAT
```

```
        5,970 5,980 5,990 6,000 6,010 6,020 6,030 6,040 6,050 6,060 6,070 6,080 6,090
Consensus                    CTGATAAGGTTTTGAGGATTTTTTTCAACATTTCCCTGCTCTGCCACTTATTCACCATGAGTTTTCATCAAATTTATCAACTCACAAATTCTTGTGAAGTATTCTGAGATTGTCTCGTGATCCTCCA
Identity
1. Dieta_contig_008195       CTGATAAGGTTTTGAGGA-TTTTTTCAACATTTCCCTGCTCTGCCACTTATTCACCATGAGTTTTCATCAAATTTATCAACTCACAAATTCTTGTGAAGTATTCTGAGATTGTCTCGTGATCCTCCA
2. P27174-4_contig_011400
3. P27174-4_contig_003576    CTGATAAGGTTTTGAGGATTTTTTTCAACATTTCCCTGCTCTGCCACTTATTCACCATGAGTTTTCATCAAATTTATCAACTCACAAATTCTTGTGAAGTATTCTGAGATTGTCTCGTGATCCTCCA
4. GR38_contig_008213         CTGATAAGGTTTTGAGGATTTTTTTCAACATTTCCCTGCTCTGCCACTTATTCACCATGAGTTTTCATCAAATTTATCAACTCACAAATTCTTGTGAAGTATTCTGAGATTGTCTCGTGATCCTCCA
5. Dieta_contig_007291
6. LalbFTc2_Chr09             CTGATAAGGTTTTGAGGATTTTTTTCAACATTTCCCTGCTCTGCCACTTATTCACCATGAGTTTTCATCAAATTTATCAACTCACAAATTCTTGTGAAGTATTCTGAGATTGTCTCGTGATCCTCCA
```

```
        6,100 6,110 6,120 6,130 6,140 6,150 6,160 6,170 6,180 6,190 6,200 6,210 6,220
Consensus                    TTTGTACAAGCTCATATTGCCTCCTTAGCATCTGTAACTTCATCTTCCTAACCTTGGTTCCTTCTGAGTAGCATTTGTCAAGAATTTCCCAAGCTTCTTTGGCATTTGATGCAGTTGAGATTTTCTC
Identity
1. Dieta_contig_008195       TTTGTACAAGCTCATATTGCCTCCTTAGCATCTGTAACTTCATCTTCCTAACCTTGGTTCCTTCTGAGTAGCATTTGTCAAGAATTTCCCAAGCTTCTTTGGCATTTGATGCAGTTGAGATTTTCTC
2. P27174-4_contig_011400
3. P27174-4_contig_003576    TTTGTACAAGCTCATATTGCCTCCTTAGCATCTGTAACTTCATCTTCCTAACCTTGGTTCCTTCTGAGTAGCATTTGTCAAGAATTTCCCAAGCTTCTTTGGCATTTGATGCAGTTGAGATTTTCTC
4. GR38_contig_008213         TTTGTACAAGCTCATATTGCCTCCTTAGCATCTGTAACTTCATCTTCCTAACCTTGGTTCCTTCTGAGTAGCATTTGTCAAGAATTTCCCAAGCTTCTTTGGCATTTGATGCAGTTGAGATTTTCTC
5. Dieta_contig_007291
6. LalbFTc2_Chr09             TTTGTACAAGCTCATATTGCCTCCTTAGCATCTGTAACTTCATCTTCCTAACCTTGGTTCCTTCTGAGTAGCATTTGTCAAGAATTTCCCAAGCTTCTTTGGCATTTGATGCAGTTGAGATTTTCTC
```

```
        6,230 6,240 6,250 6,260 6,270 6,280 6,290 6,300 6,310 6,320 6,330 6,340 6,350
Consensus                    AAAATTTGCCCTATCCACCTACTGATGGATCAAGAAGATTGCCTTACAATCTCTCTTTGCGGCTTCTTTGAACTCAATATTCTCTGCTTCTGTTAGAATTTCATTCAATGGAAGAACTCCAGCTTTC
Identity
1. Dieta_contig_008195       AAAATTTGCCCTATCCACCTACTGATGGATCAAGAAGATTGCCTTACAATCTCTCTTTGTGGCTTCTTTGAACTCAATATTCTCTGCTTCTGTTAGAATTTCATTCAATGGAAGAACTCCAGCTTTC
2. P27174-4_contig_011400
3. P27174-4_contig_003576    AAAATTTGCCCTATCCACCTACTGATGGATCAAGAAGATTGCCTTACAATCTCTCTTTGCGGCTTCTTTGAACTCAATATTCTCTGCTTCTGTTAGAATTTCATTCAATGGAAGAACTCCAGCTTTC
4. GR38_contig_008213         AAAATTTGCCCTATCCACCTACTGATGGATCAAGAAGATTGCCTTACAATCTCTCTTTGCGGCTTCTTTGAACTCAATATTCTCTGCTTCTGTTAGAATTTCATTCAATGGAAGAACTCCAGCTTTC
5. Dieta_contig_007291
6. LalbFTc2_Chr09             AAAATTTGCCCTATCCACCTACTGATGGATCAAGAAGATTGCCTTACAATCTCTCTTTGCGGCTTCTTTGAACTCAATATTCTCTGCTTCTGTTAGAATTTCATTCAATGGAAGAACTCCAGCTTTC
```

```
        6,360 6,370 6,380 6,390 6,400 6,410 6,420 6,430 6,440 6,450 6,460 6,470
Consensus                    ACCACATCCAAACAATCTTGGAACCCGAAAATGACTTTCATTTGGATGCTCCAATGGTCATAATTCTTGTCATCAAGAATGGGTAGATTCATTGCAAACCCATTTGAAGTTGTCATTCTTGTGCTTC
Identity
1. Dieta_contig_008195       ACCACATCCAAACAATCTTGGAACCCGAAAATGACTTTCATTTGGATGCTCCAATGGTCATAATTCTTGTCATCAAGAATGGGTAGATTCATCGCAAACCCATTTGAAGTTGTCATTCTTGTGCTTC
2. P27174-4_contig_011400
3. P27174-4_contig_003576    ACCACATCCAAACAATCTTGGAACCCGAAAATGACTTTCATTTGGATGCTCCAATGGTCATAATTCTTGTCATCAAGAATGGGTAGATTCATTGCAAACCCATTTGAAGTTGTCATTCTTGTGCTTC
4. GR38_contig_008213         ACCACATCCAAACAATCTTGGAACCCGAAAATGACTTTCATTTGGATGCTCCAATGGTCATAATTCTTGTCATCAAGAATGGGTAGATTCATTGCAAACCCATTTGAAGTTGTCATTCTTGTGCTTC
5. Dieta_contig_007291
6. LalbFTc2_Chr09             ACCACATCCAAACAATCTTGGAACCCGAAAATGACTTTCATTTGGATGCTCCAATGGTCATAATTCTTGTCATCAAGAATGGGTAGATTCATTGCAAACCCATTTGAAGTTGTCATTCTTGTGCTTC
```

```
        6,480 6,490 6,500 6,510 6,520 6,530 6,540 6,550 6,560 6,570 6,580 6,590 6,600
Consensus                    TTGATTCAAATTGACTCTCCTTCTTCCTCCTTTCTTTCTCCCAAATCTGATCACCACCGTGAGCTTATTCTTGAAGCCTCTTGCACCACCTTAGGCACCACCACAGGTTCTGAATCGACGTGATGGC
Identity
1. Dieta_contig_008195       TTGATTCAAATTGACTCTCCTTATTCCTCCTTTCTTTCTCCCAAATCTGATCACCACCGTGAGCTTATTCTTGAAGCCTCTTGCACCACCTTAGGCACCACCACAGGTTCTGGATCGACGTGATGGC
2. P27174-4_contig_011400
3. P27174-4_contig_003576    TTGATTCAAATTGACTCTCCTTCTTCCTCCTTTCTTTCTCCCAAATCTGATCACCACCGTGAGCTTATTCTTGAAGCCTCTTGCACCACCTTAGGCACCACCACAGGTTCTGAATCGACGTGATGGC
4. GR38_contig_008213         TTGATTCAAATTGACTCTCCTTCTTCCTCCTTTCTTTCTCCCAAATCTGATCACCACCGTGAGCTTATTCTTGAAGCCTCTTGCACCACCTTAGGCACCACCACAGGTTCTGAATCGACGTGATGGC
5. Dieta_contig_007291
6. LalbFTc2_Chr09             TTGATTCAAATTGACTCTCCTTCTTCCTCCTTTCTTTCTCCCAAATCTGATCACCACCGTGAGCTTATTCTTGAAGCCTCTTGCACCACCTTAGGCACCACCACAGGTTCTGAATCGACGTGATGGC
```

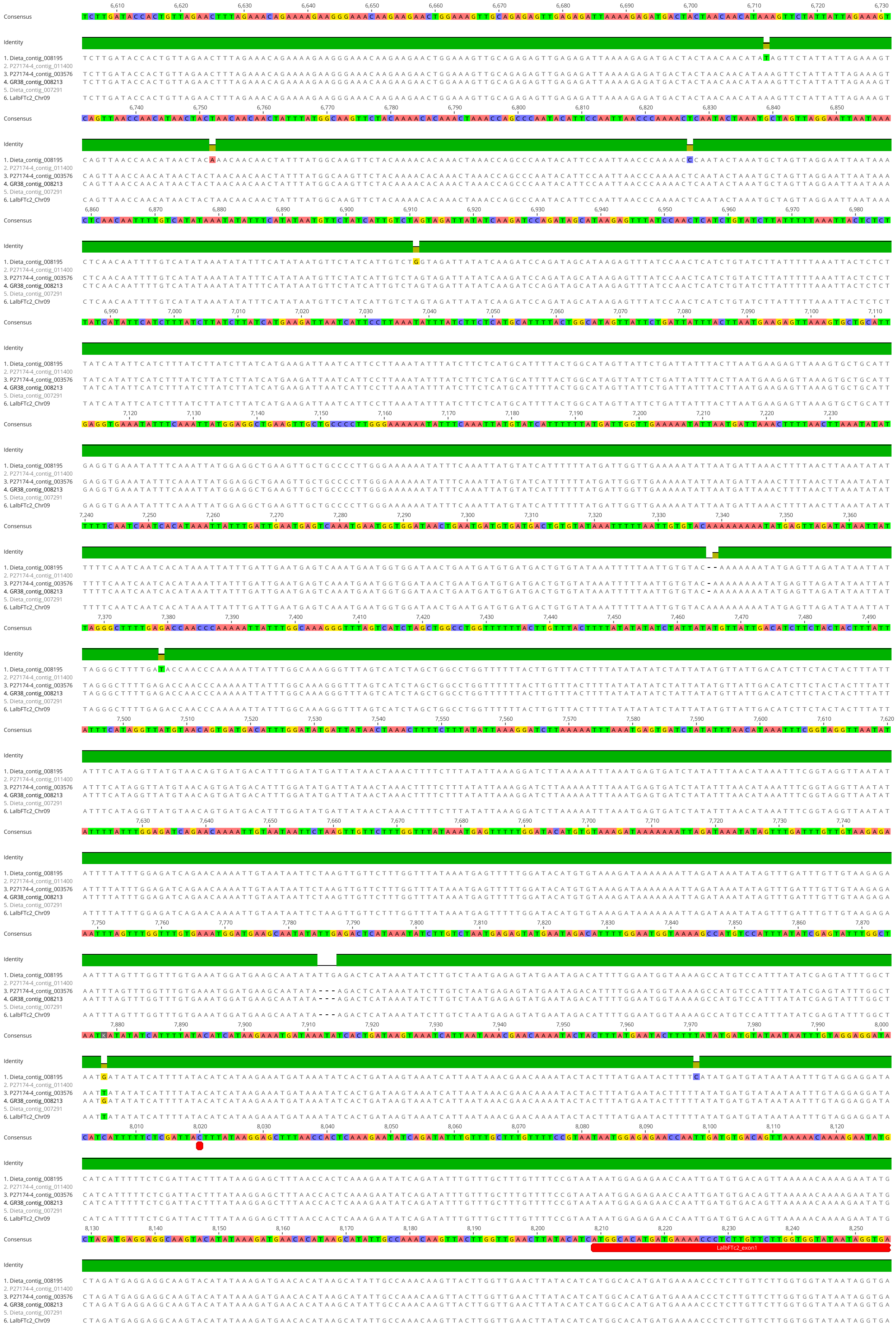
Consensus
Identity
1. Dieta_contig_008195
2. P27174-4_contig_011400
3. P27174-4_contig_003576
4. GR38_contig_008213
5. Dieta_contig_007291
6. LalbFTc2_Chr09
LalbFTc2_exon1

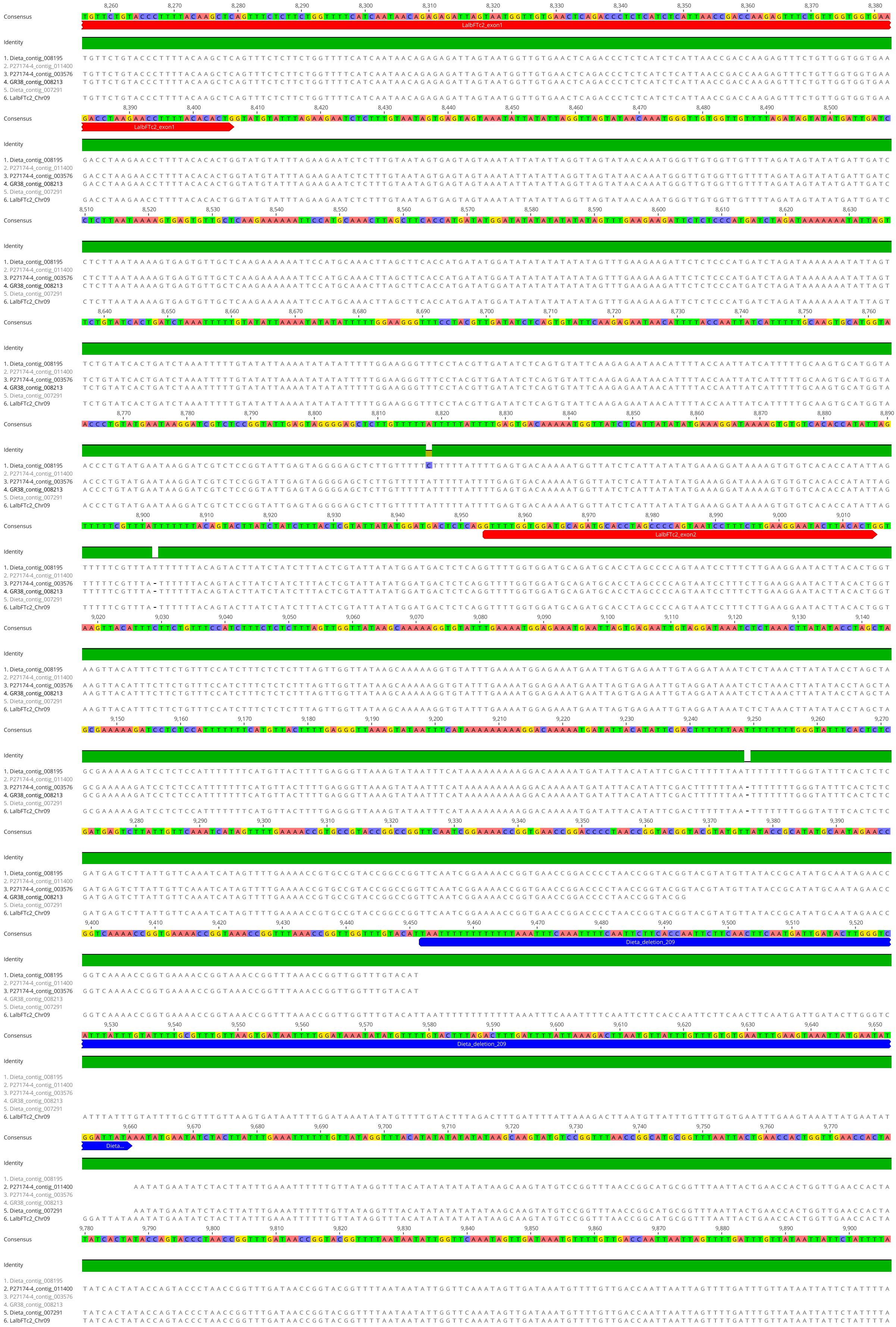
Consensus
Identity
1. Dieta_contig_008195
2. P27174-4_contig_011400
3. P27174-4_contig_003576
4. GR38_contig_008213
5. Dieta_contig_007291
6. LalbFTc2_Chr09
LalbFTc2_exon1
LalbFTc2_exon2
Dieta_deletion_209
Dieta...
8,260
9,900

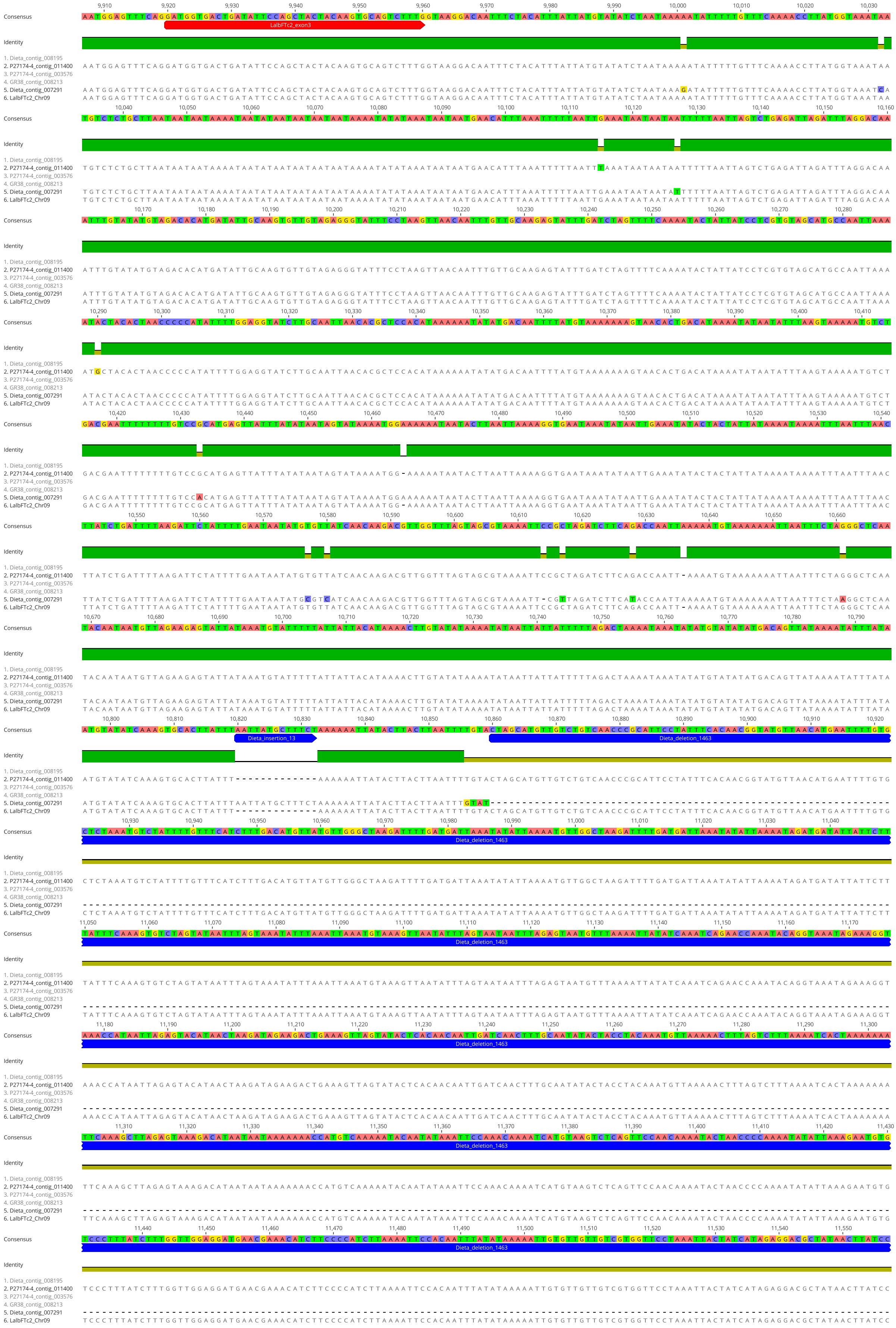
Consensus
Identity
1. Dieta_contig_008195
2. P27174-4_contig_011400
3. P27174-4_contig_003576
4. GR38_contig_008213
5. Dieta_contig_007291
6. LalbFTc2_Chr09
LalbFTc2_exon3
Dieta_insertion_13
Dieta_deletion_1463

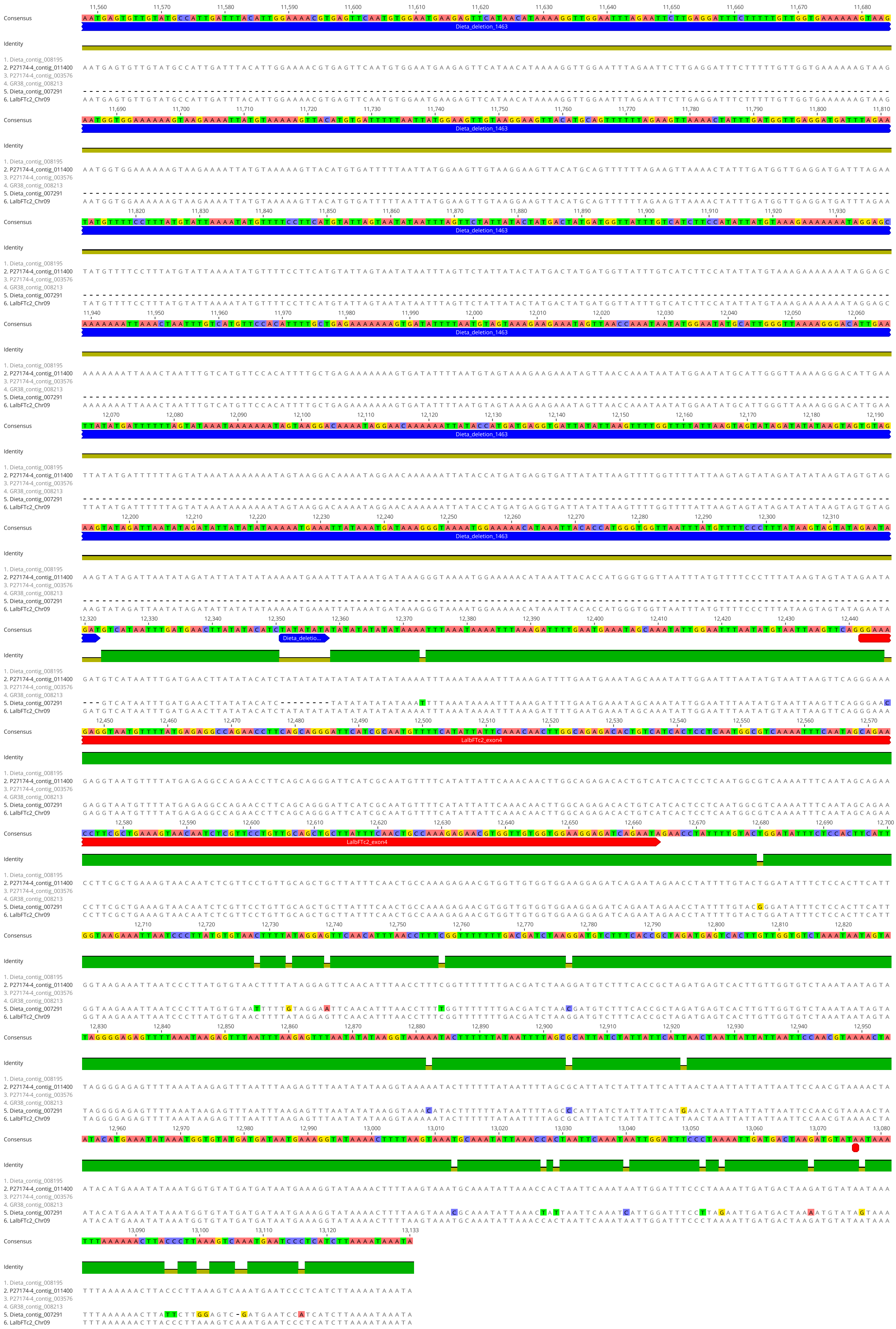
Consensus
Identity
1. Dieta_contig_008195
2. P27174-4_contig_011400
3. P27174-4_contig_003576
4. GR38_contig_008213
5. Dieta_contig_007291
6. LalbFTc2_Chr09
Dieta_deletion_1463
LalbFTc2_exon4
