# Supplementary material for: Evaluation of the Global White Lupin Collection Reveals Significant Associations Between Homologous FLOWERING LOCUS T Indels and Flowering Time, Providing Validated Markers for Tracking Spring Ecotypes Within a Large Gene Pool
Source: Int J Mol Sci. 2025 Jul 17;26(14):6858. doi: 10.3390/ijms26146858 (PMC12295241; doi:10.3390/ijms26146858)
Supplement: Supplementary file 1 [file ijms-26-06858-s001.zip › Supplementary_Figure_S2_Agarose gel electrophoregrams showing polymorphism of PCR-based markers targeting LalbFTa1 indels.pdf]

Wojciech Bielski, Anna Surma, Michał Książkiewicz, Sandra Rychel-Bielska

Evaluation of the global white lupin collection reveals significant associations between homologous *FLOWERING LOCUS T* indels and flowering time, providing validated markers for tracking spring ecotypes within a large gene pool

International Journal of Molecular Sciences

Supplementary Figure S2. Agarose gel electrophoregrams showing polymorphism of PCR-based markers targeting *LalbFTa1* indels.

PR_01

PRFTa1_F TGAAATTGATTTGCTAGGTTTCTCCC

PRFTa1_R TGTCTAGTAGTCTATGCCTGTGAAA

Plate 1

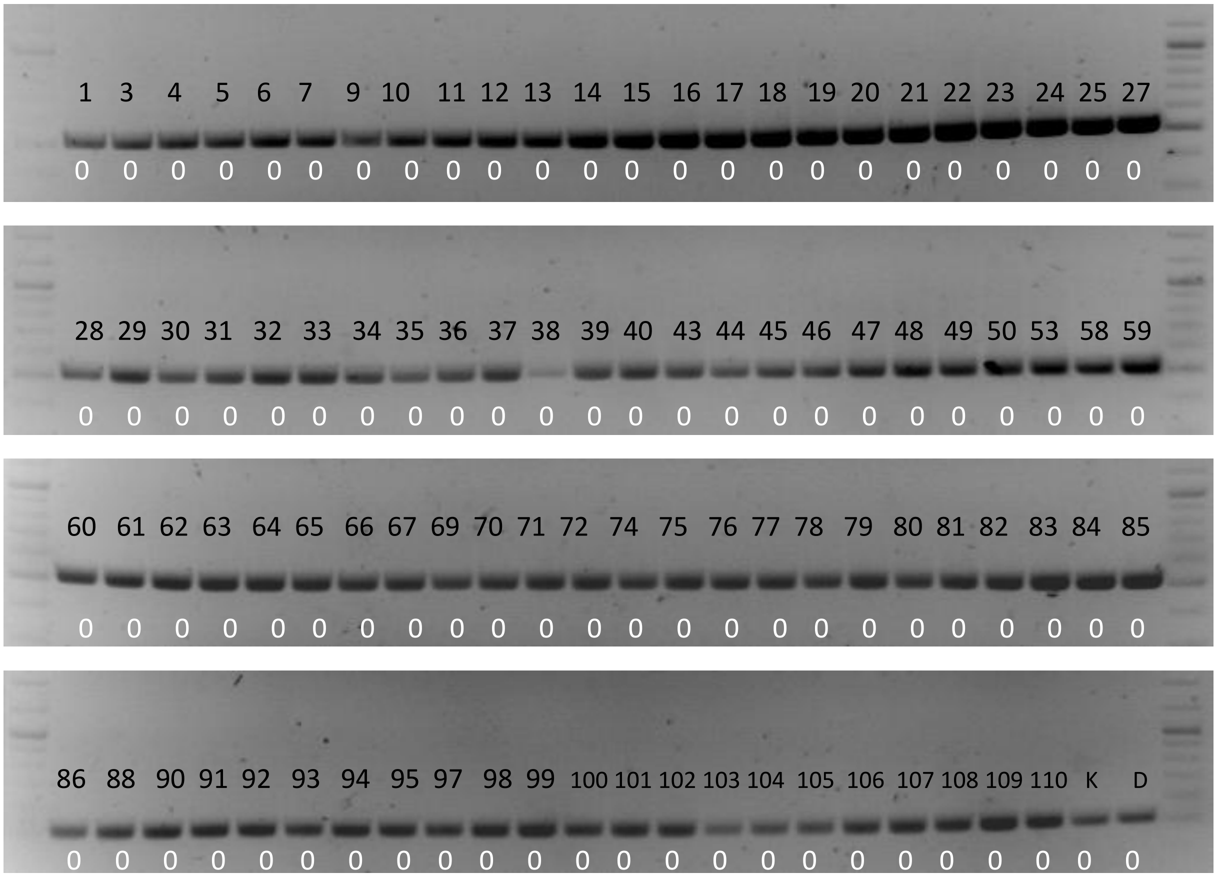

Plate 7

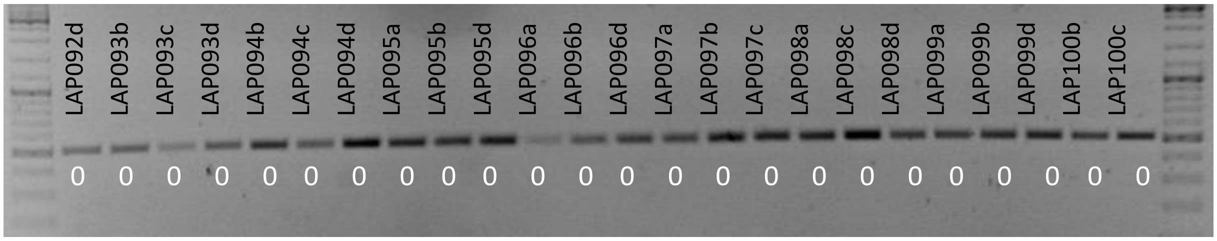
LAP092d LAP093b LAP093c LAP093d LAP094b LAP094c LAP094d LAP095a LAP095b LAP095d LAP096a LAP096b LAP096d LAP097a LAP097b LAP097c LAP098a LAP098c LAP098d LAP099a LAP099b LAP099d LAP100b LAP100c
0 0 0 0 0 0 0 0 0 0 0 0 0 0 0 0 0 0 0 0 0 0 0 0

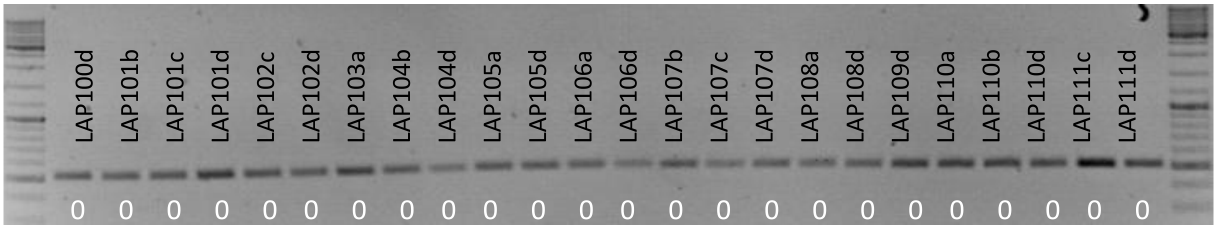
LAP100d LAP101b LAP101c LAP101d LAP102c LAP102d LAP103a LAP104b LAP104d LAP105a LAP105d LAP106a LAP106d LAP107b LAP107c LAP107d LAP108a LAP108d LAP109d LAP110a LAP110b LAP110d LAP111c LAP111d
0 0 0 0 0 0 0 0 0 0 0 0 0 0 0 0 0 0 0 0 0 0 0 0

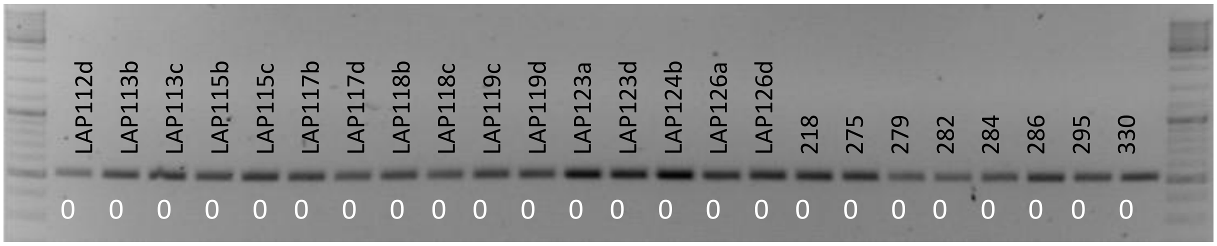
LAP112d LAP113b LAP113c LAP115b LAP115c LAP117b LAP117d LAP118b LAP118c LAP119c LAP119d LAP123a LAP123d LAP124b LAP126a LAP126d 218 275 279 282 284 286 295 330
0 0 0 0 0 0 0 0 0 0 0 0 0 0 0 0 0 0 0 0 0 0 0 0

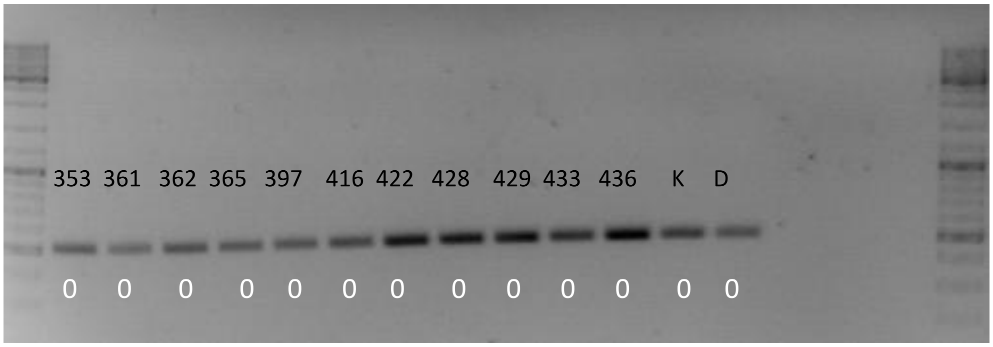
353 361 362 365 397 416 422 428 429 433 436 K D
0 0 0 0 0 0 0 0 0 0 0 0 0

PR_02

PRFTA1F1 ACAAGGAGCTATTTTAACAAACTACT

PRFTa1_R1b TGACACTTAACAATTCACAAGGACT

Plate 1

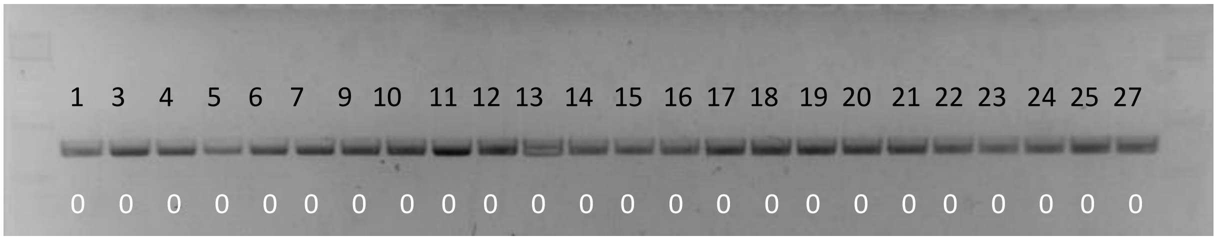

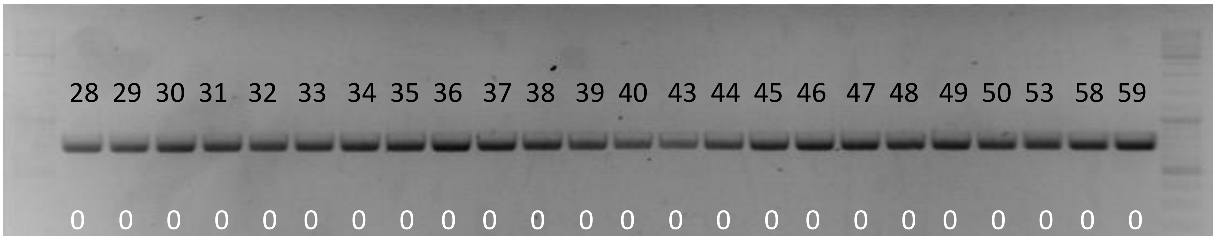

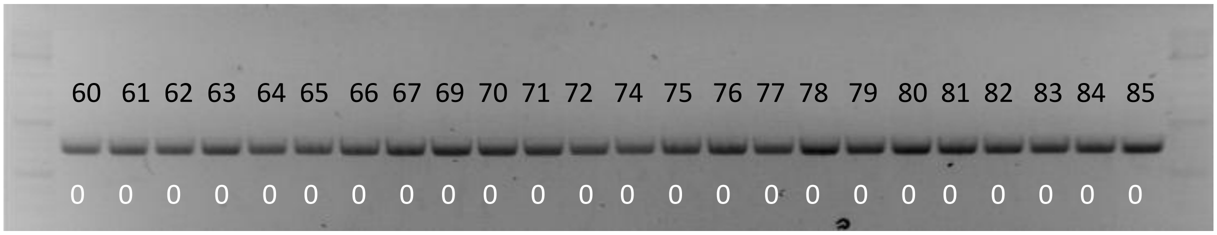

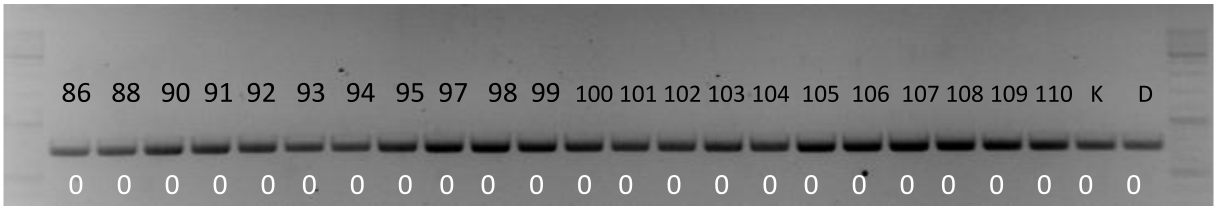

Plate 7

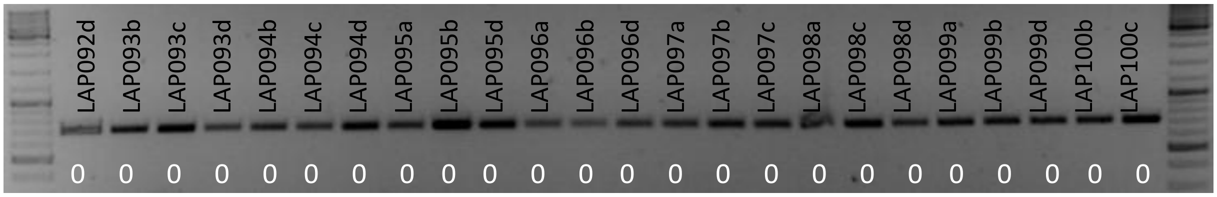
LAP092d LAP093b LAP093c LAP093d LAP094b LAP094c LAP094d LAP095a LAP095b LAP095d LAP096a LAP096b LAP096d LAP097a LAP097b LAP097c LAP098a LAP098c LAP098d LAP099a LAP099b LAP099d LAP100b LAP100c
0 0 0 0 0 0 0 0 0 0 0 0 0 0 0 0 0 0 0 0 0 0 0 0

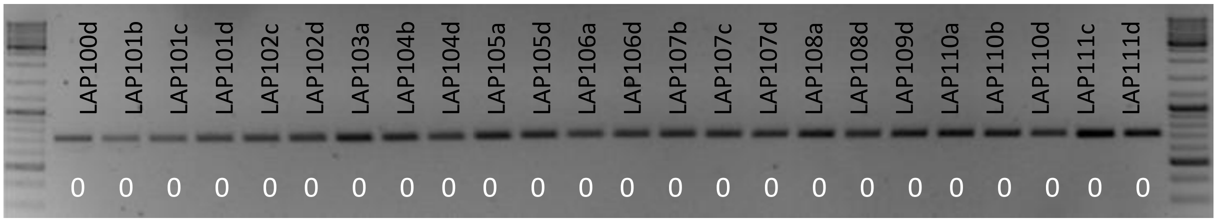
LAP100d LAP101b LAP101c LAP101d LAP102c LAP102d LAP103a LAP104b LAP104d LAP105a LAP105d LAP106a LAP106d LAP107b LAP107c LAP107d LAP108a LAP108d LAP109d LAP110a LAP110b LAP110d LAP111c LAP111d
0 0 0 0 0 0 0 0 0 0 0 0 0 0 0 0 0 0 0 0 0 0 0 0

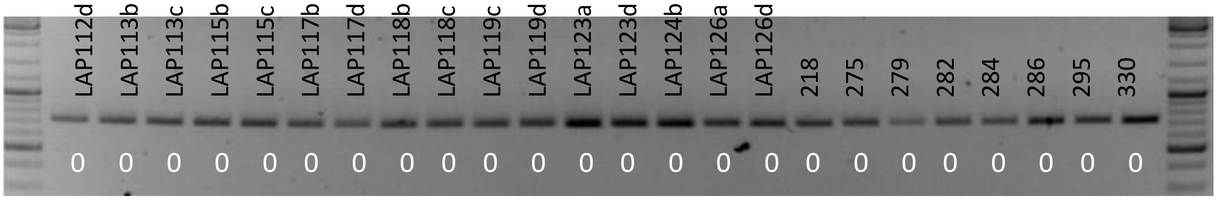
LAP112d LAP113b LAP113c LAP115b LAP115c LAP117b LAP117d LAP118b LAP118c LAP119c LAP119d LAP123a LAP123d LAP124b LAP126a LAP126d 218 275 279 282 284 286 295 330
0 0 0 0 0 0 0 0 0 0 0 0 0 0 0 0 0 0 0 0 0 0 0 0

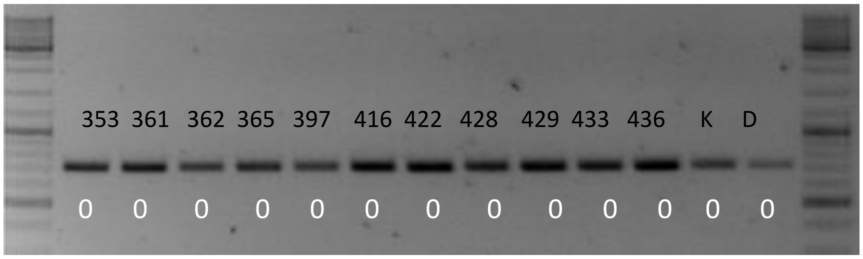
353 361 362 365 397 416 422 428 429 433 436 K D
0 0 0 0 0 0 0 0 0 0 0 0 0

PR_03

PRFTa1_F1b ATTCAACAATTCGATCACACCATTT

PRFTa1_R1c GTTCAATGCATCGCCTATGTTCTAG

Plate 1

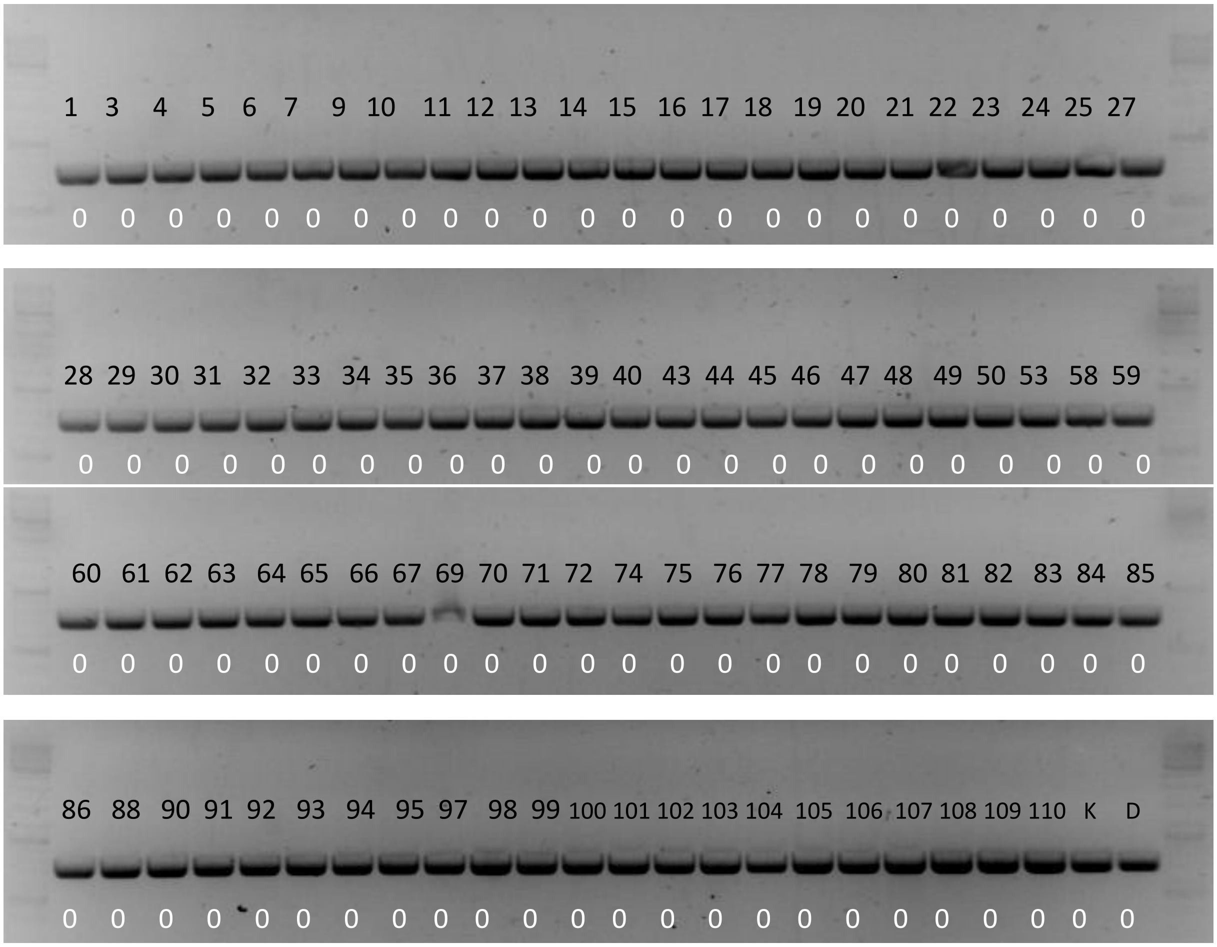

Plate 7

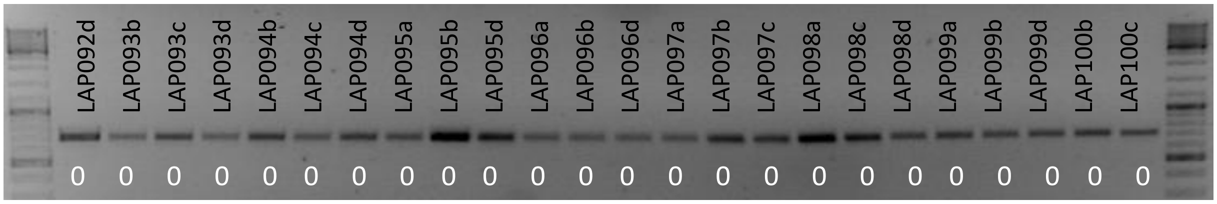
LAP092d LAP093b LAP093c LAP093d LAP094b LAP094c LAP094d LAP095a LAP095b LAP095d LAP096a LAP096b LAP096d LAP097a LAP097b LAP097c LAP098a LAP098c LAP098d LAP099a LAP099b LAP099d LAP100b LAP100c
0 0 0 0 0 0 0 0 0 0 0 0 0 0 0 0 0 0 0 0 0 0 0 0

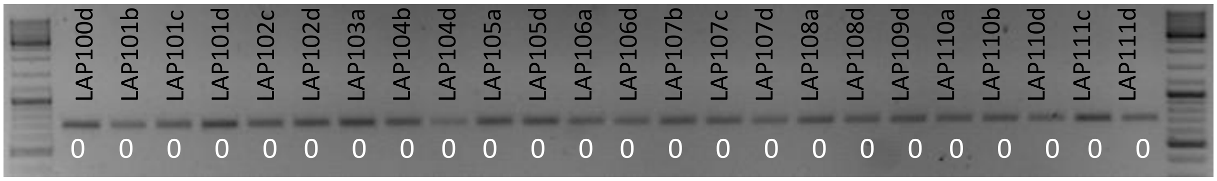
LAP100d LAP101b LAP101c LAP101d LAP102c LAP102d LAP103a LAP104b LAP104d LAP105a LAP105d LAP106a LAP106d LAP107b LAP107c LAP107d LAP108a LAP108d LAP109d LAP110a LAP110b LAP110d LAP111c LAP111d
0 0 0 0 0 0 0 0 0 0 0 0 0 0 0 0 0 0 0 0 0 0 0 0

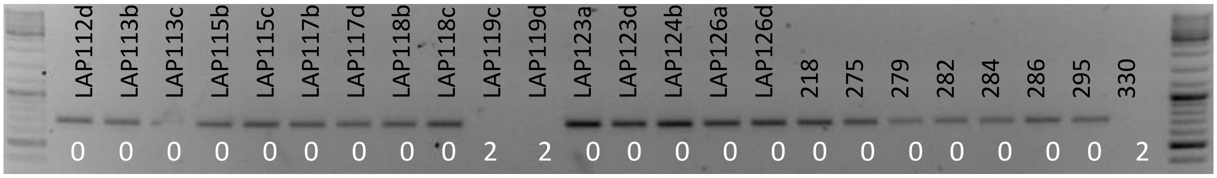
LAP112d LAP113b LAP113c LAP115b LAP115c LAP117b LAP117d LAP118b LAP118c LAP119c LAP119d LAP123a LAP123d LAP124b LAP126a LAP126d 218 275 279 282 284 286 295 330
0 0 0 0 0 0 0 0 0 2 2 0 0 0 0 0 0 0 0 0 0 0 0 2

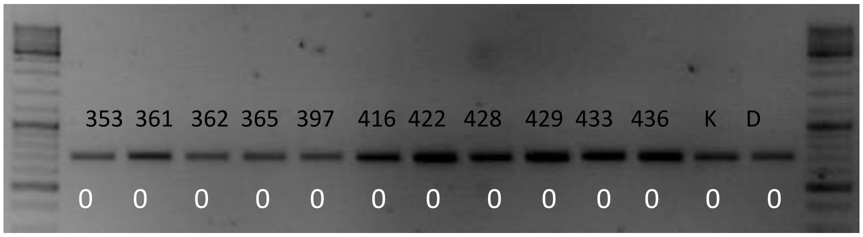
353 361 362 365 397 416 422 428 429 433 436 K D
0 0 0 0 0 0 0 0 0 0 0 0 0

PR_04

PRFTa1_F1c AAGAATTCAACCATGACTAGTCCGA

PRFTA1R1 TGTCTAGTAGTCTATGCCATTCAGT

Plate 1

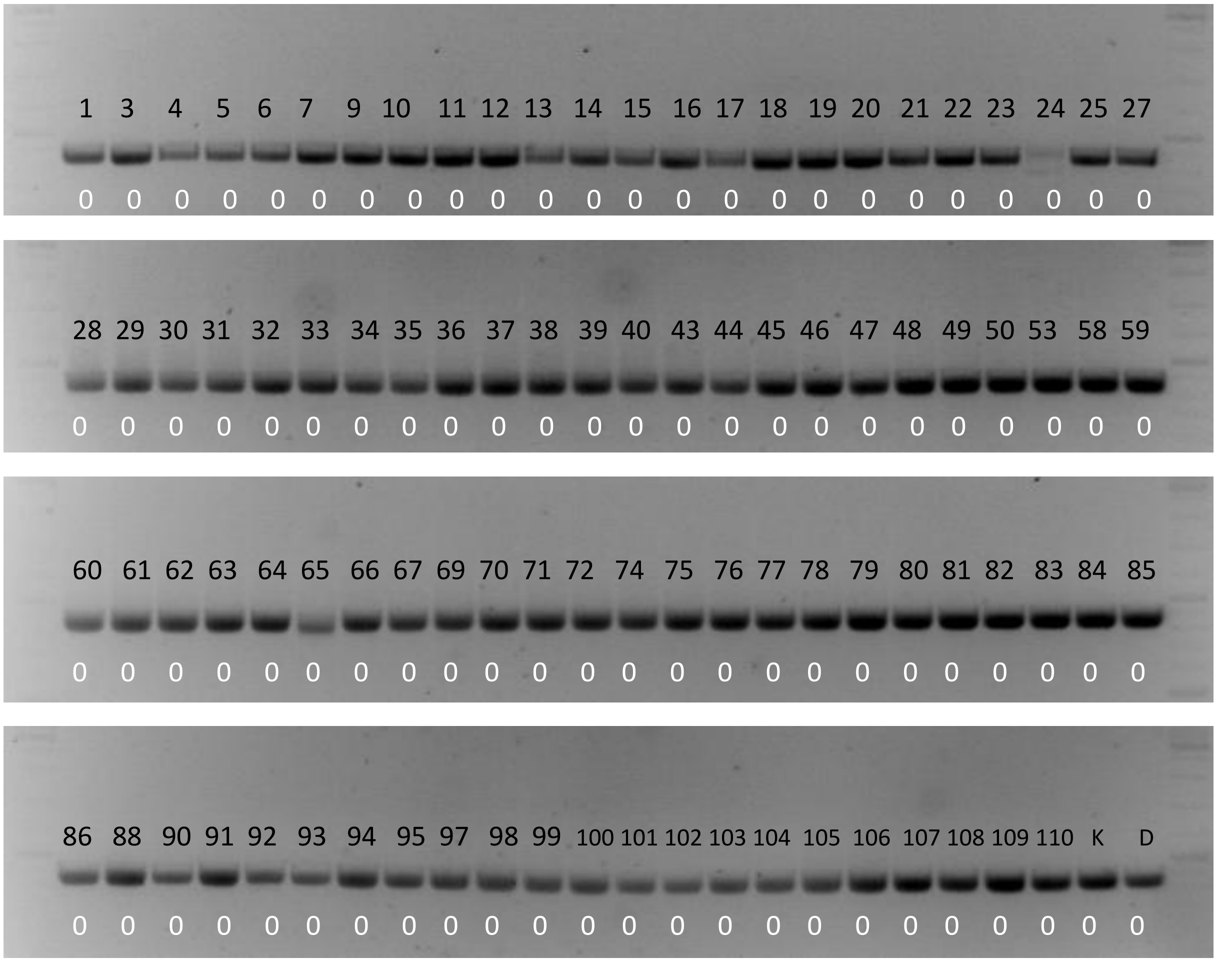

Plate 7

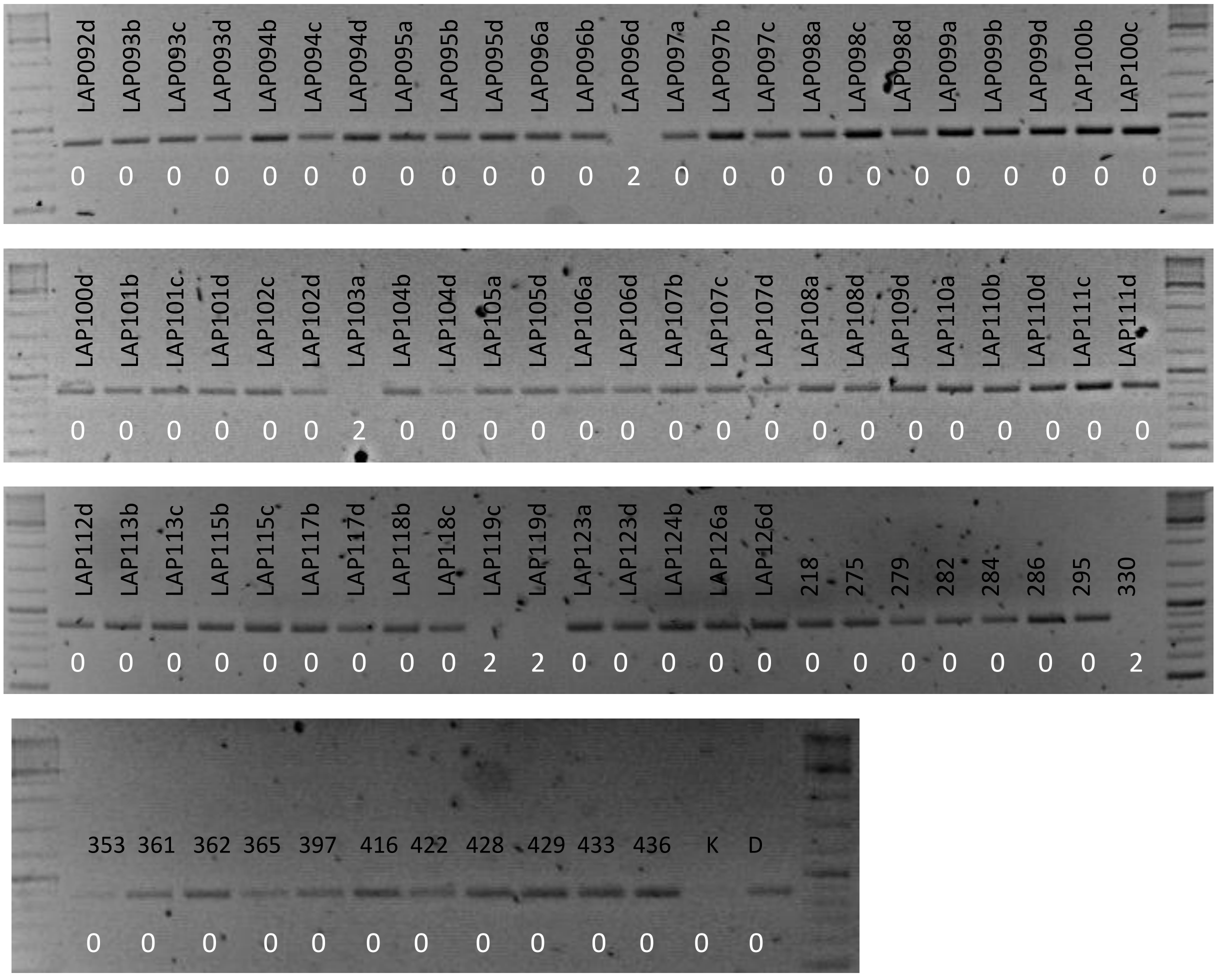
LAP092d LAP093b LAP093c LAP093d LAP094b LAP094c LAP094d LAP095a LAP095b LAP095d LAP096a LAP096b LAP096d LAP097a LAP097b LAP097c LAP098a LAP098c LAP098d LAP099a LAP099b LAP099d LAP100b LAP100c
0 0 0 0 0 0 0 0 0 0 0 0 2 0 0 0 0 0 0 0 0 0 0 0
LAP100d LAP101b LAP101c LAP101d LAP102c LAP102d LAP103a LAP104b LAP104d LAP105a LAP105d LAP106a LAP106d LAP107b LAP107c LAP107d LAP108a LAP108d LAP109d LAP110a LAP110b LAP110d LAP111c LAP111d
0 0 0 0 0 0 2 0 0 0 0 0 0 0 0 0 0 0 0 0 0 0 0 0
LAP112d LAP113b LAP113c LAP115b LAP115c LAP117b LAP117d LAP118b LAP118c LAP119c LAP119d LAP123a LAP123d LAP124b LAP126a LAP126d 218 275 279 282 284 286 295 330
0 0 0 0 0 0 0 0 0 2 2 0 0 0 0 0 0 0 0 0 0 0 0 2
353 361 362 365 397 416 422 428 429 433 436 K D
0 0 0 0 0 0 0 0 0 0 0 0 0

PR_05a

PRFTA1F2 TTGTTATCTCATGCGTATTACTCATT

PRFTa1_R2b TTATACGAGTTGAAAGTTACGTGCA

Plate 1

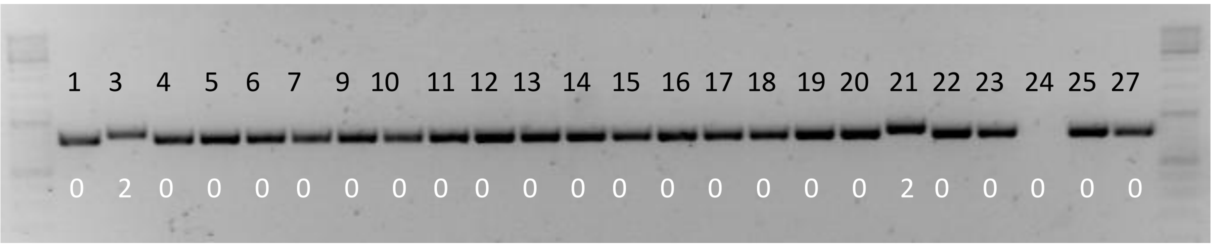

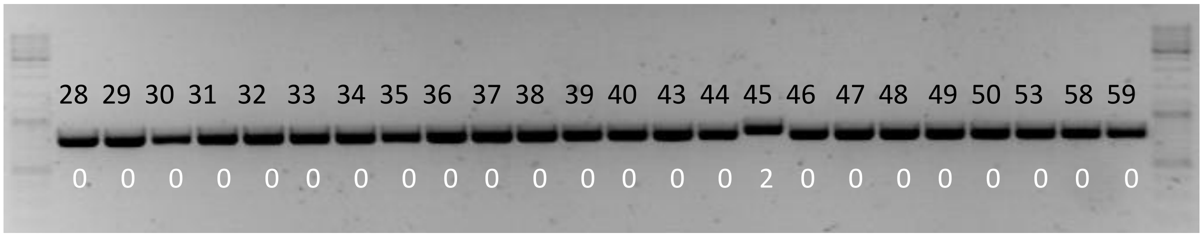

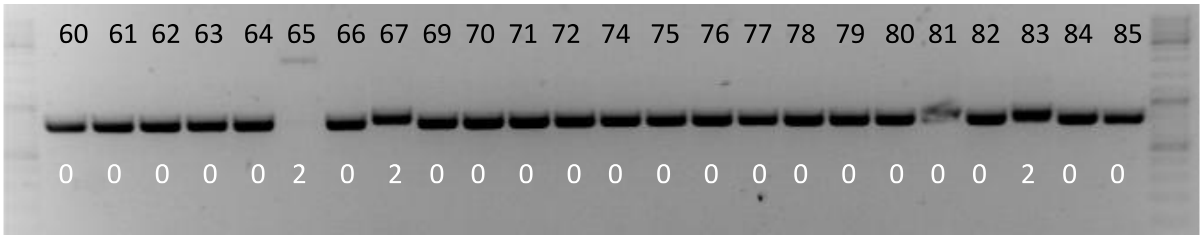

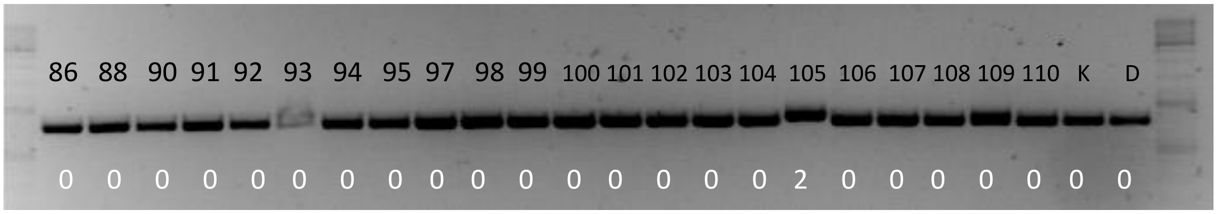

Plate 7

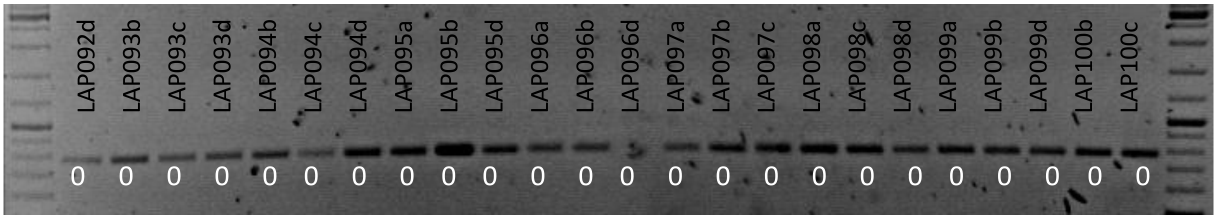
LAP092d LAP093b LAP093c LAP093d LAP094b LAP094c LAP094d LAP095a LAP095b LAP095d LAP096a LAP096b LAP096d LAP097a LAP097b LAP097c LAP098a LAP098c LAP098d LAP099a LAP099b LAP099d LAP100b LAP100c
0 0 0 0 0 0 0 0 0 0 0 0 0 0 0 0 0 0 0 0 0 0 0 0

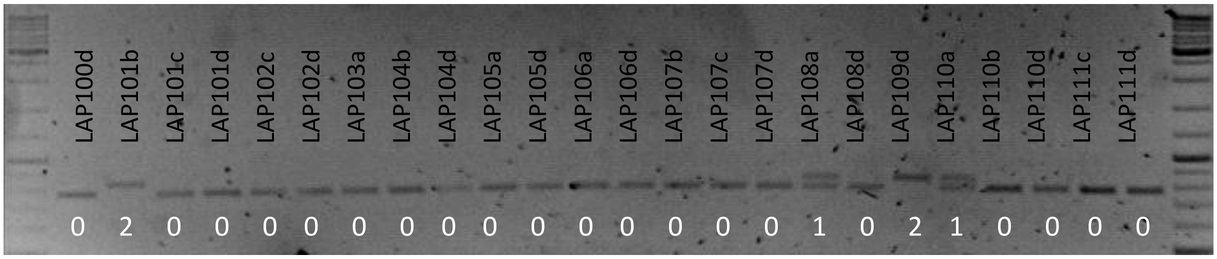
LAP100d LAP101b LAP101c LAP101d LAP102c LAP102d LAP103a LAP104b LAP104d LAP105a LAP105d LAP106a LAP106d LAP107b LAP107c LAP107d LAP108a LAP108d LAP109d LAP110a LAP110b LAP110d LAP111c LAP111d
0 2 0 0 0 0 0 0 0 0 0 0 0 0 0 0 1 0 2 1 0 0 0 0

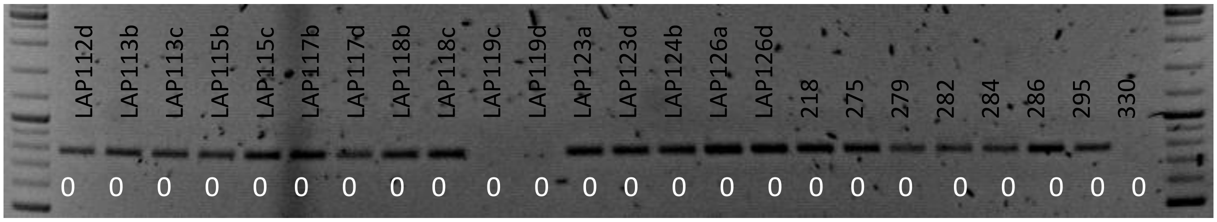
LAP112d LAP113b LAP113c LAP115b LAP115c LAP117b LAP117d LAP118b LAP118c LAP119c LAP119d LAP123a LAP123d LAP124b LAP126a LAP126d 218 275 279 282 284 286 295 330
0 0 0 0 0 0 0 0 0 0 0 0 0 0 0 0 0 0 0 0 0 0 0 0

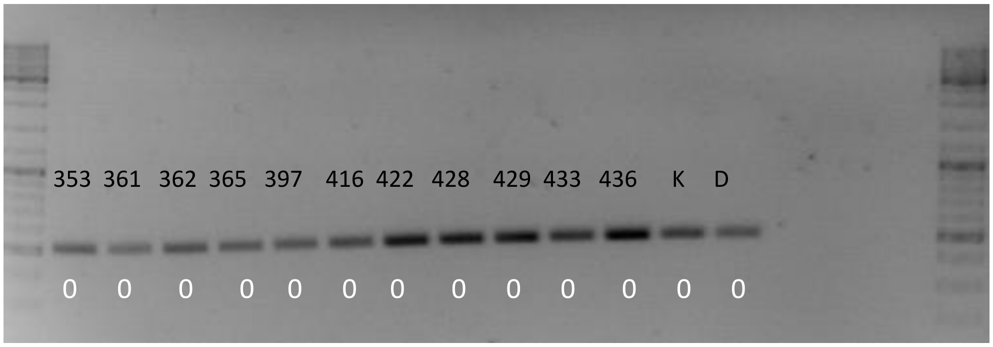
353 361 362 365 397 416 422 428 429 433 436 K D
0 0 0 0 0 0 0 0 0 0 0 0 0

PR_05b

PRFTA1F2 TTGTTATCTCATGCGTATTACTCATT

PRFTa1_R2b TTATACGAGTTGAAAGTTACGTGCA

Plate 1

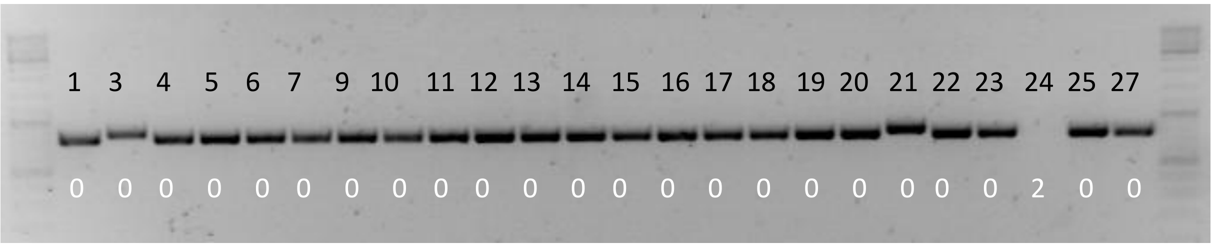

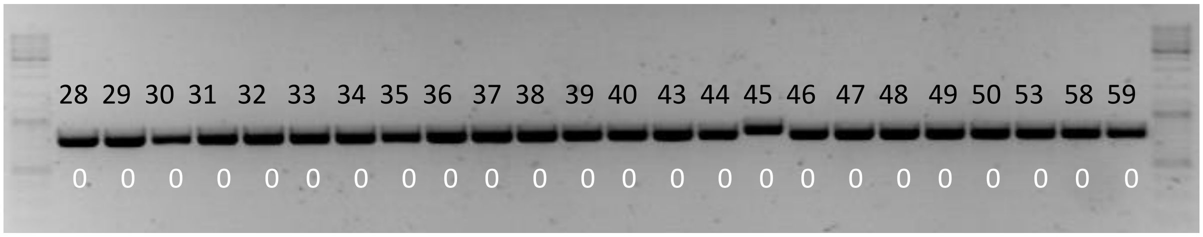

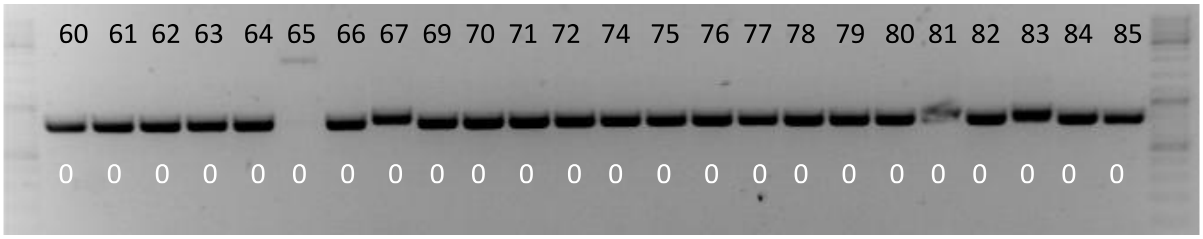

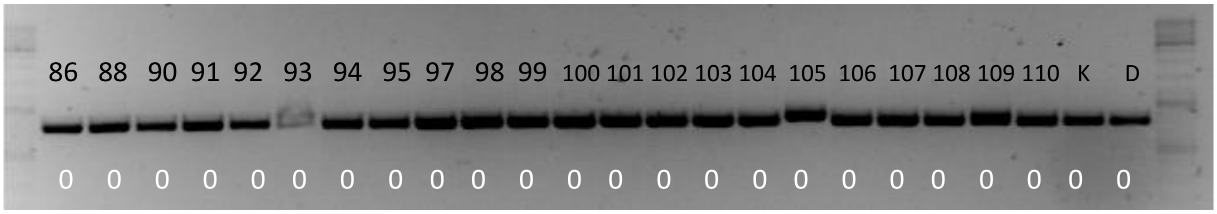

Plate 7

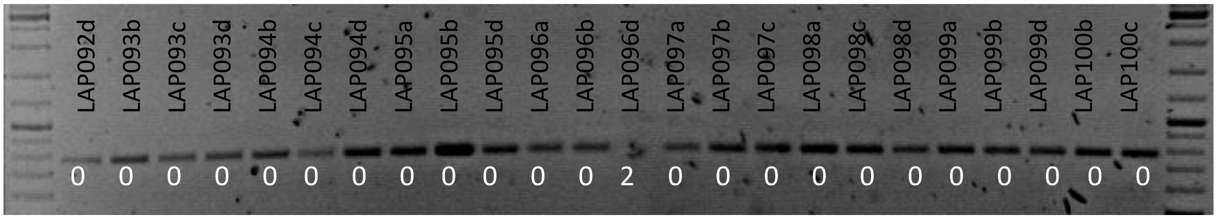
LAP092d LAP093b LAP093c LAP093d LAP094b LAP094c LAP094d LAP095a LAP095b LAP095d LAP096a LAP096b LAP096d LAP097a LAP097b LAP097c LAP098a LAP098c LAP098d LAP099a LAP099b LAP099d LAP100b LAP100c
0 0 0 0 0 0 0 0 0 0 0 0 2 0 0 0 0 0 0 0 0 0 0 0

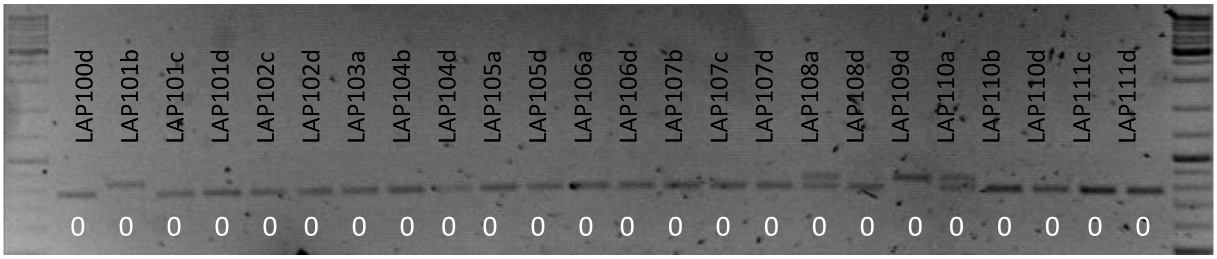
LAP100d LAP101b LAP101c LAP101d LAP102c LAP102d LAP103a LAP104b LAP104d LAP105a LAP105d LAP106a LAP106d LAP107b LAP107c LAP107d LAP108a LAP108d LAP109d LAP110a LAP110b LAP110d LAP111c LAP111d
0 0 0 0 0 0 0 0 0 0 0 0 0 0 0 0 0 0 0 0 0 0 0 0

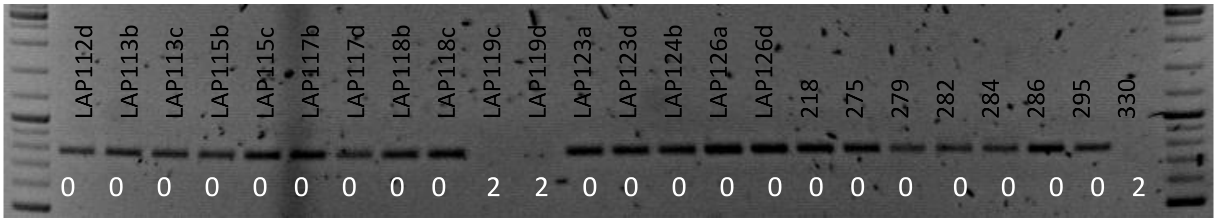
LAP112d LAP113b LAP113c LAP115b LAP115c LAP117b LAP117d LAP118b LAP118c LAP119c LAP119d LAP123a LAP123d LAP124b LAP126a LAP126d 218 275 279 282 284 286 295 330
0 0 0 0 0 0 0 0 0 2 2 0 0 0 0 0 0 0 0 0 0 0 0 2

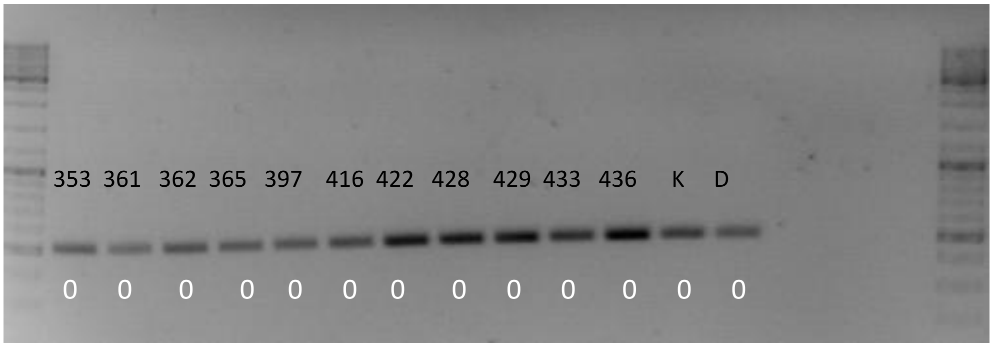
353 361 362 365 397 416 422 428 429 433 436 K D
0 0 0 0 0 0 0 0 0 0 0 0 0

PR74

PRFTa1_F1c AAGAATTCAACCATGACTAGTCCGA

PRFTa1_F2aR0 AGCACTTTACGAAAAATAGTTATATGTCT

Plate 1

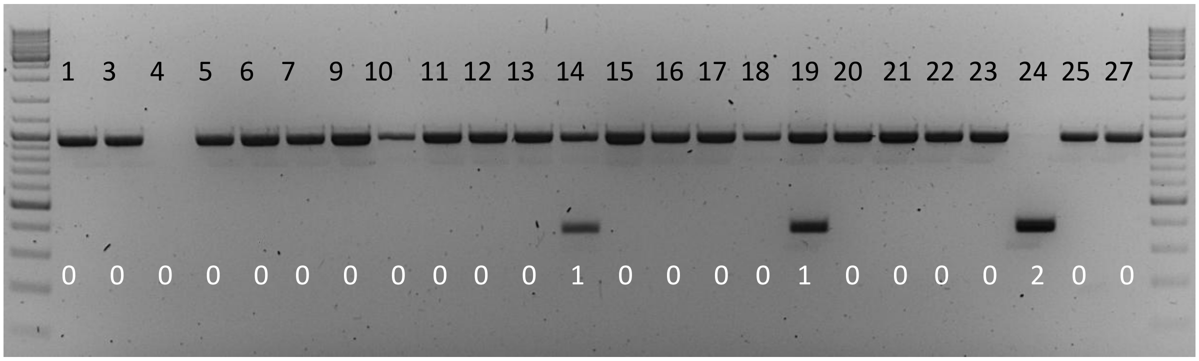

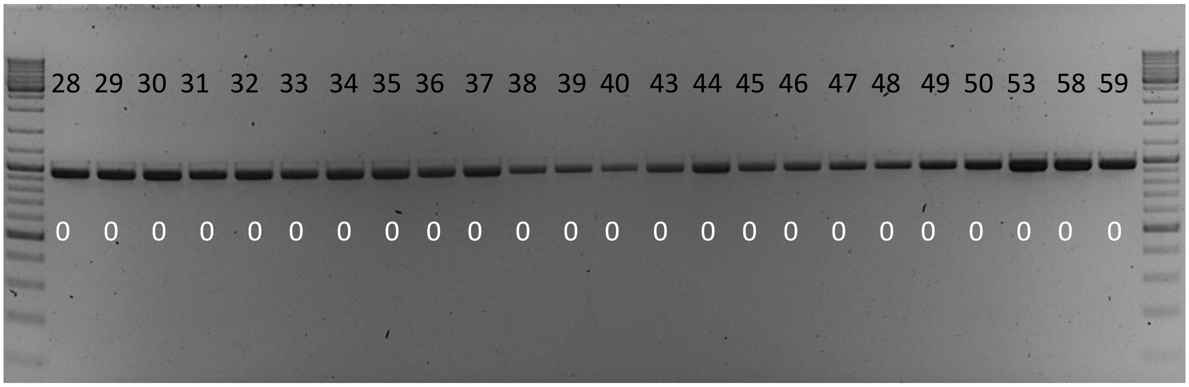

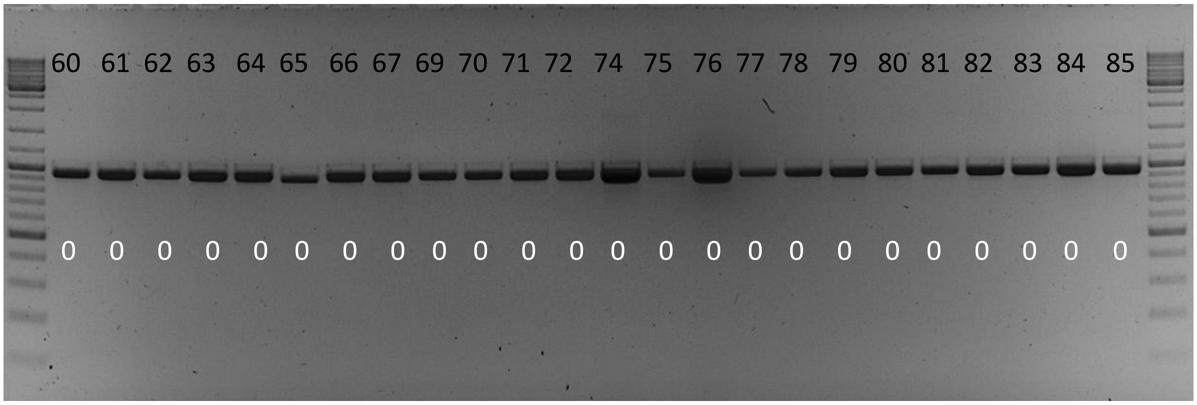

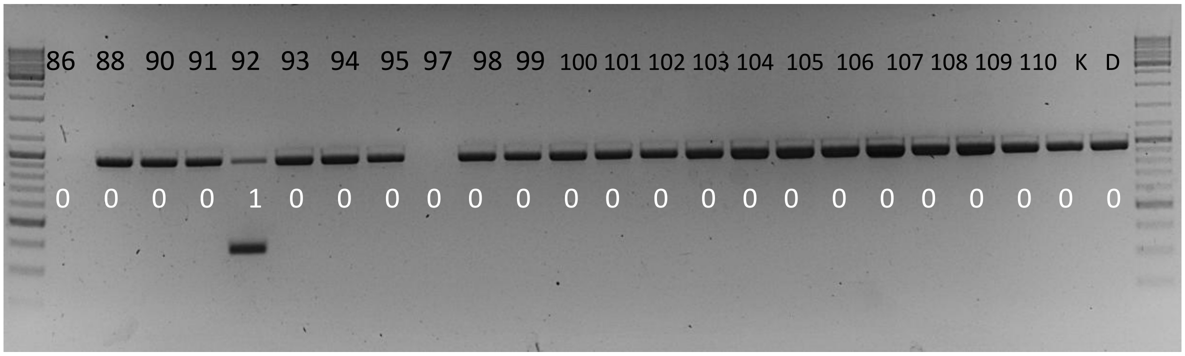

Plate 7

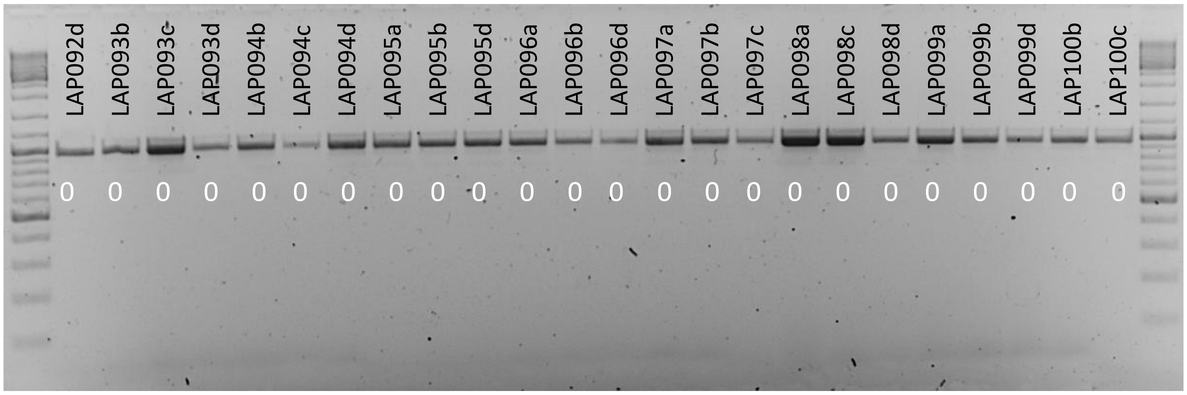
LAP092d LAP093b LAP093c LAP093d LAP094b LAP094c LAP094d LAP095a LAP095b LAP095d LAP096a LAP096b LAP096d LAP097a LAP097b LAP097c LAP098a LAP098c LAP098d LAP099a LAP099b LAP099d LAP100b LAP100c
0 0 0 0 0 0 0 0 0 0 0 0 0 0 0 0 0 0 0 0 0 0 0 0

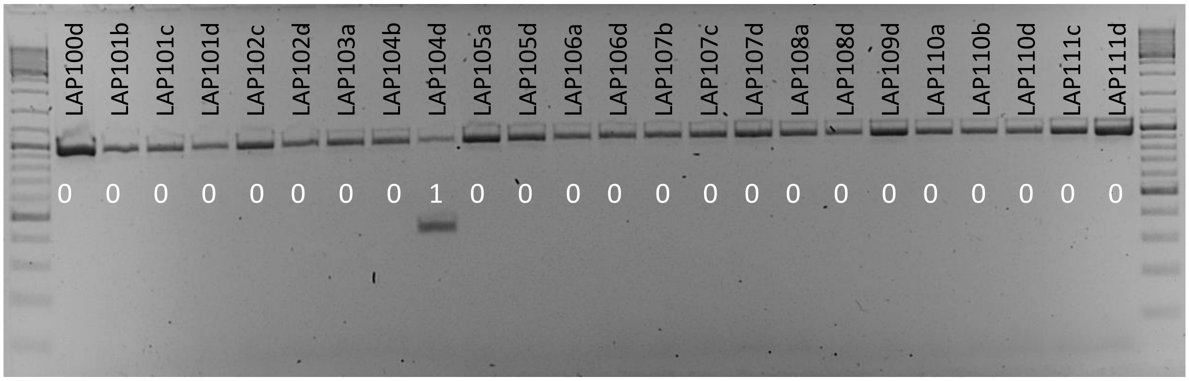
LAP100d LAP101b LAP101c LAP101d LAP102c LAP102d LAP103a LAP104b LAP104d LAP105a LAP105d LAP106a LAP106d LAP107b LAP107c LAP107d LAP108a LAP108d LAP109d LAP110a LAP110b LAP110d LAP111c LAP111d
0 0 0 0 0 0 0 0 1 0 0 0 0 0 0 0 0 0 0 0 0 0 0 0

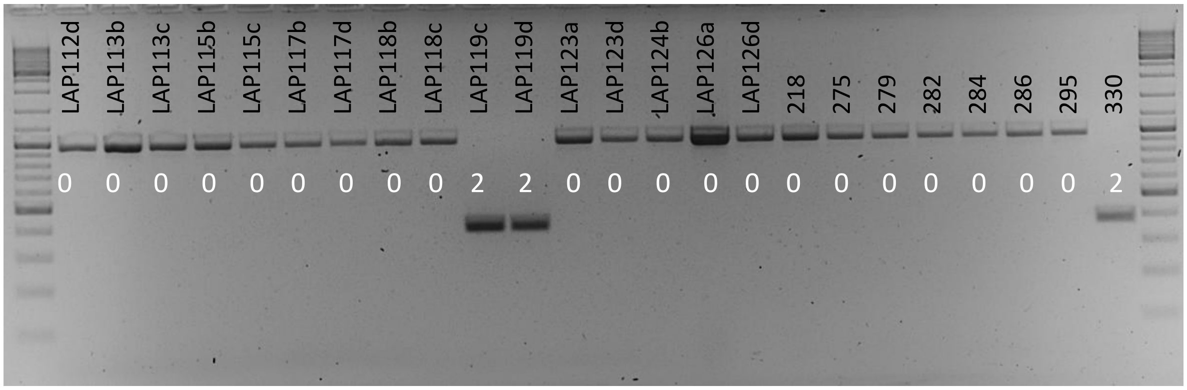
LAP112d LAP113b LAP113c LAP115b LAP115c LAP117b LAP117d LAP118b LAP118c LAP119c LAP119d LAP123a LAP123d LAP124b LAP126a LAP126d 218 275 279 282 284 286 295 330
0 0 0 0 0 0 0 0 0 2 2 0 0 0 0 0 0 0 0 0 0 0 0 2

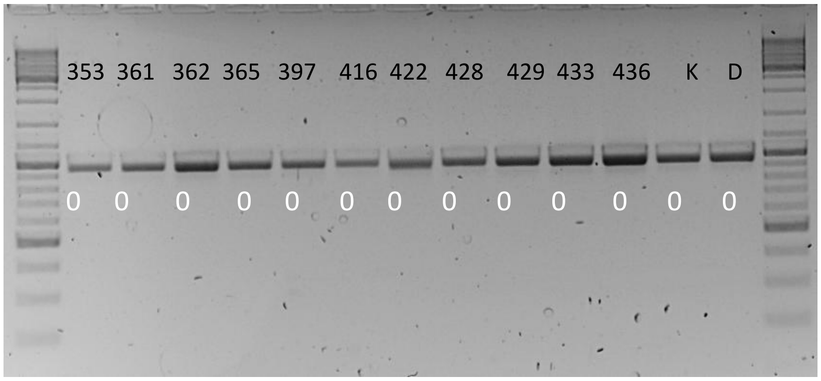
353 361 362 365 397 416 422 428 429 433 436 K D
0 0 0 0 0 0 0 0 0 0 0 0 0

Repeat

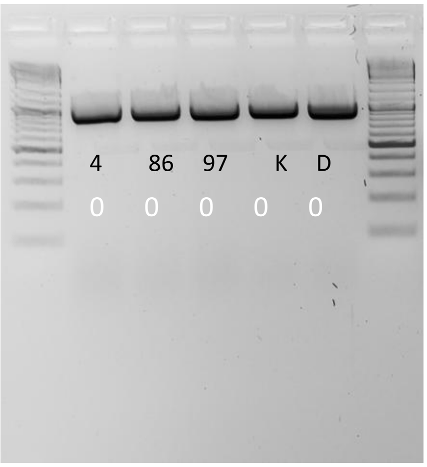
4
86
97
K
D
0 0 0 0 0

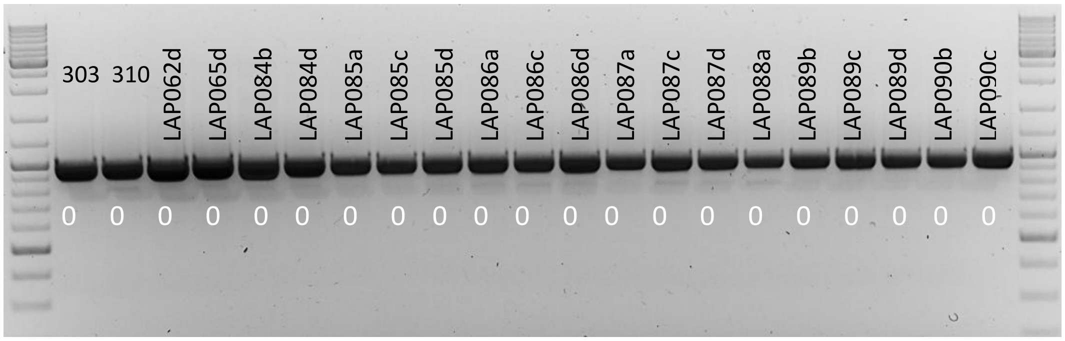
303
310
LAP062d
LAP065d
LAP084b
LAP084d
LAP085a
LAP085c
LAP085d
LAP086a
LAP086c
LAP086d
LAP087a
LAP087c
LAP087d
LAP088a
LAP089b
LAP089c
LAP089d
LAP090b
LAP090c
0 0 0 0 0 0 0 0 0 0 0 0 0 0 0 0 0 0 0 0 0

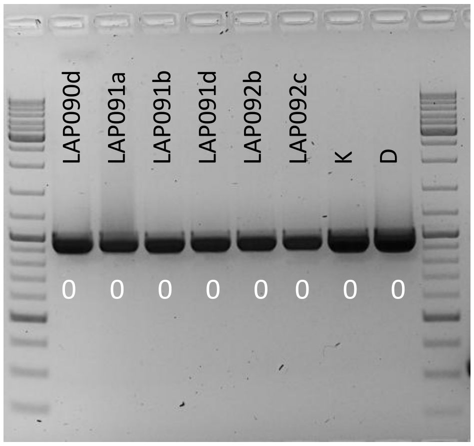
LAP090d
LAP091a
LAP091b
LAP091d
LAP092b
LAP092c
K
D
0 0 0 0 0 0 0 0

PR75

PRFTa1_F2a1 AGACATATAACTATTTTTCGTAAAGTGCT

PRFTa1_R2a1 TCCGATACCATGTAGAGGAAAAA

Plate 1

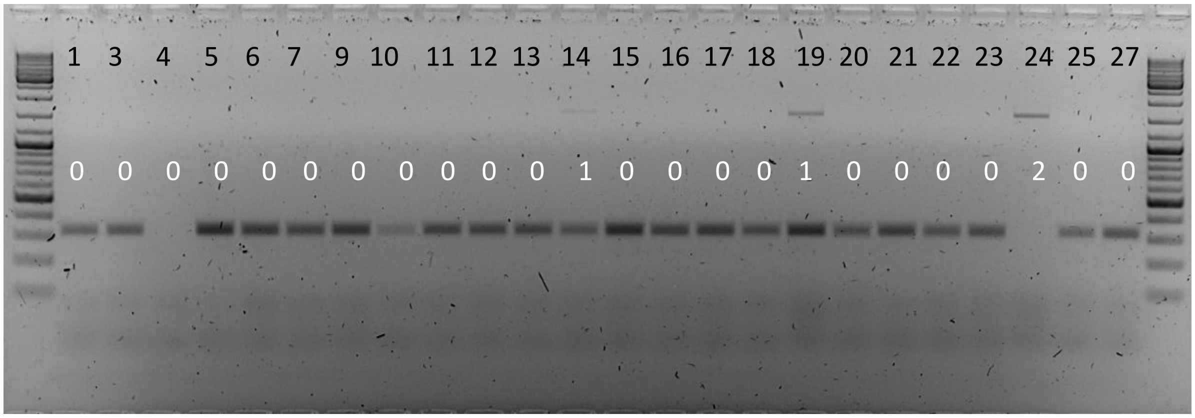

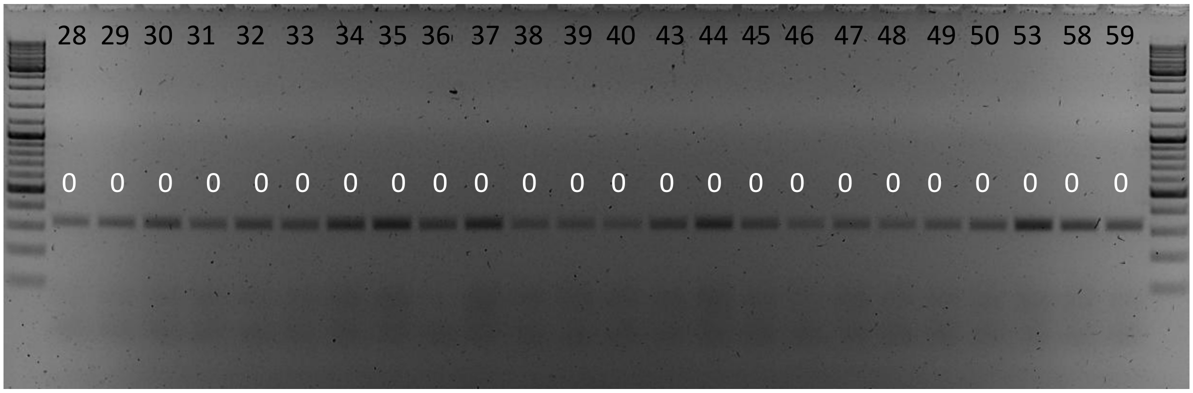

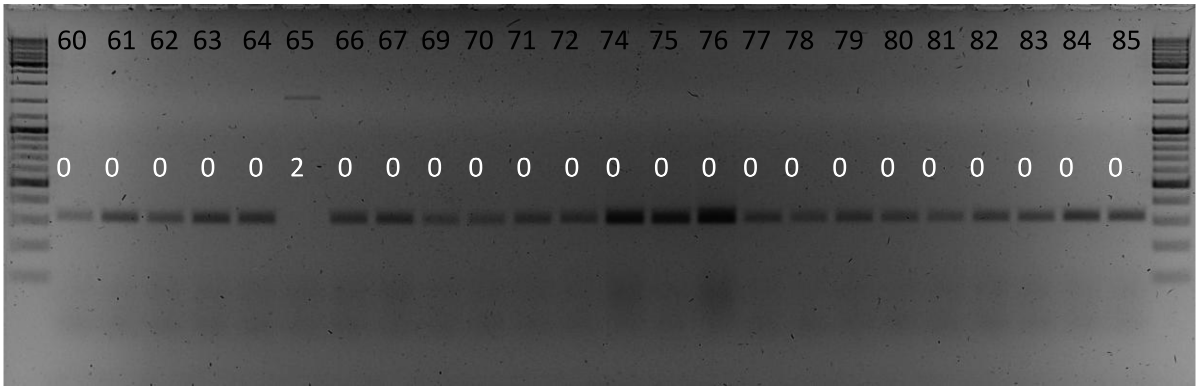

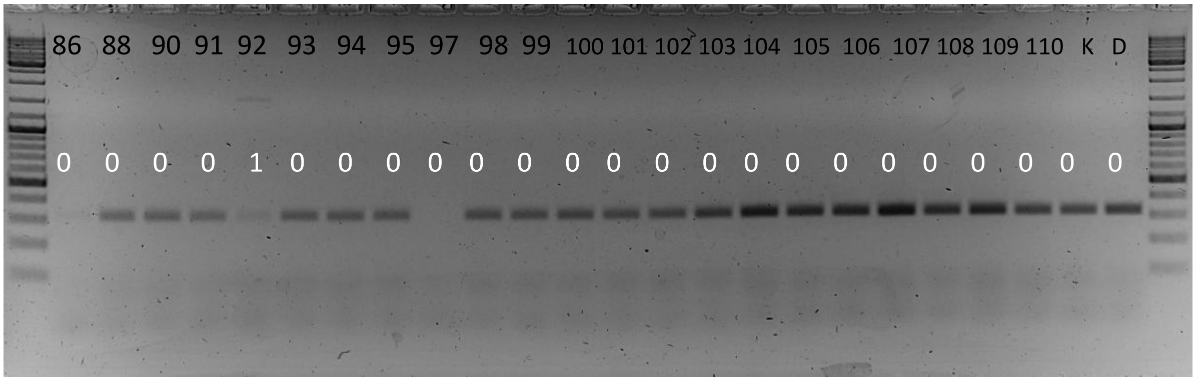

Plate 7

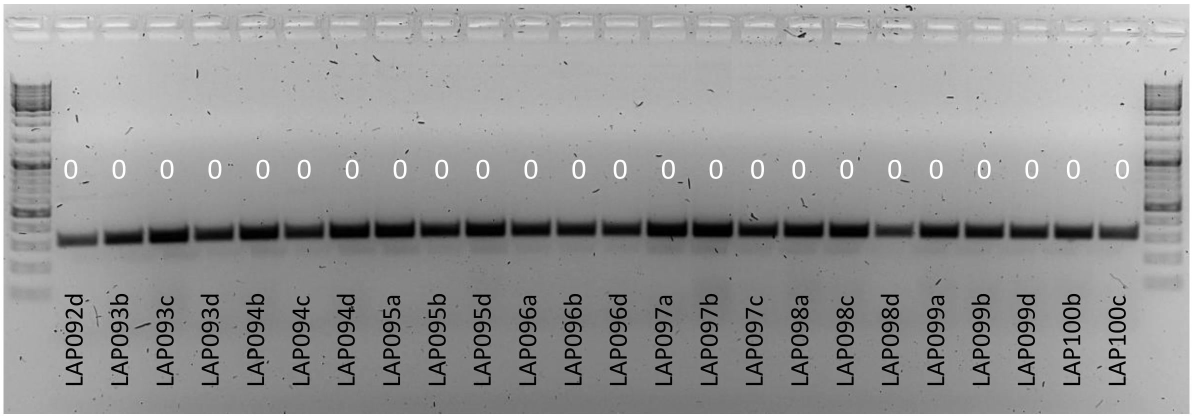
0 0 0 0 0 0 0 0 0 0 0 0 0 0 0 0 0 0 0 0 0 0 0 0
LAP092d LAP093b LAP093c LAP093d LAP094b LAP094c LAP094d LAP095a LAP095b LAP095d LAP096a LAP096b LAP096d LAP097a LAP097b LAP097c LAP098a LAP098c LAP098d LAP099a LAP099b LAP099d LAP100b LAP100c

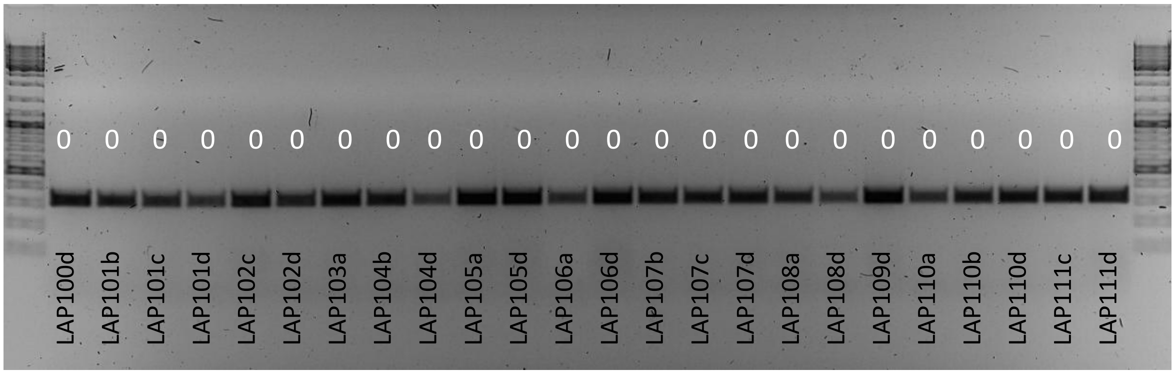
0 0 0 0 0 0 0 0 0 0 0 0 0 0 0 0 0 0 0 0 0 0 0 0
LAP100d LAP101b LAP101c LAP101d LAP102c LAP102d LAP103a LAP104b LAP104d LAP105a LAP105d LAP106a LAP106d LAP107b LAP107c LAP107d LAP108a LAP108d LAP109d LAP110a LAP110b LAP110d LAP111c LAP111d

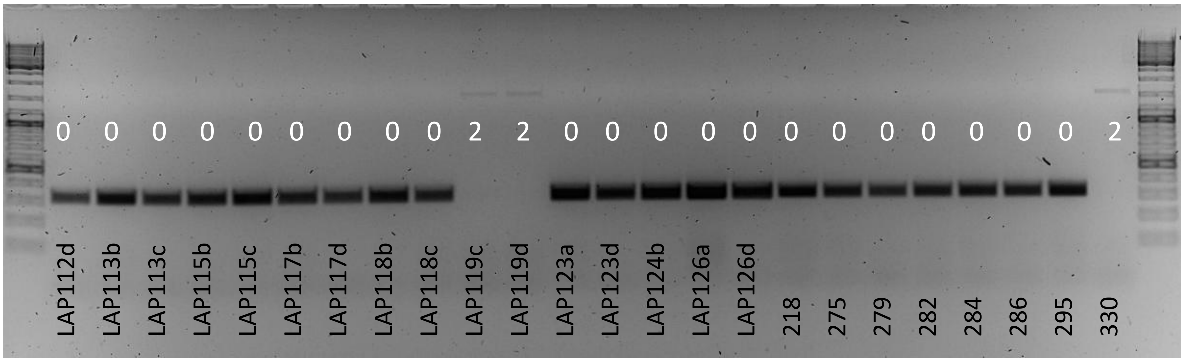
0 0 0 0 0 0 0 0 0 2 2 0 0 0 0 0 0 0 0 0 0 0 0 2
LAP112d LAP113b LAP113c LAP115b LAP115c LAP117b LAP117d LAP118b LAP118c LAP119c LAP119d LAP123a LAP123d LAP124b LAP126a LAP126d 218 275 279 282 284 286 295 330

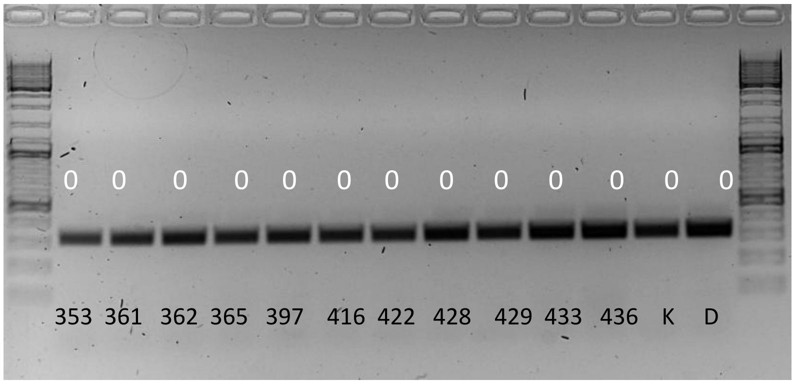
0 0 0 0 0 0 0 0 0 0 0 0 0
353 361 362 365 397 416 422 428 429 433 436 K D

Repeat

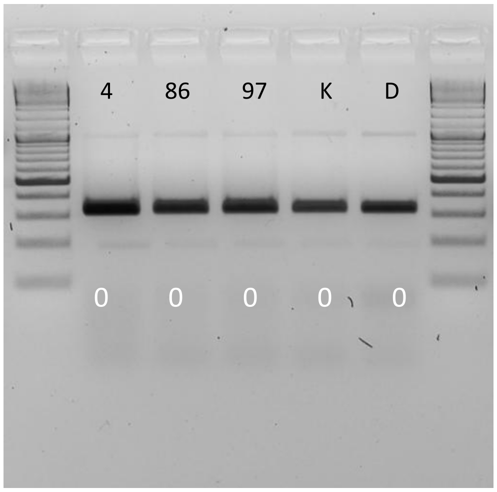

PR76

PRFTa1_F2a2 AGTATTTTCCCTGTATGGTCCCT

PRFTa1_R2b TTATACGAGTTGAAAGTTACGTGCA

Plate 1

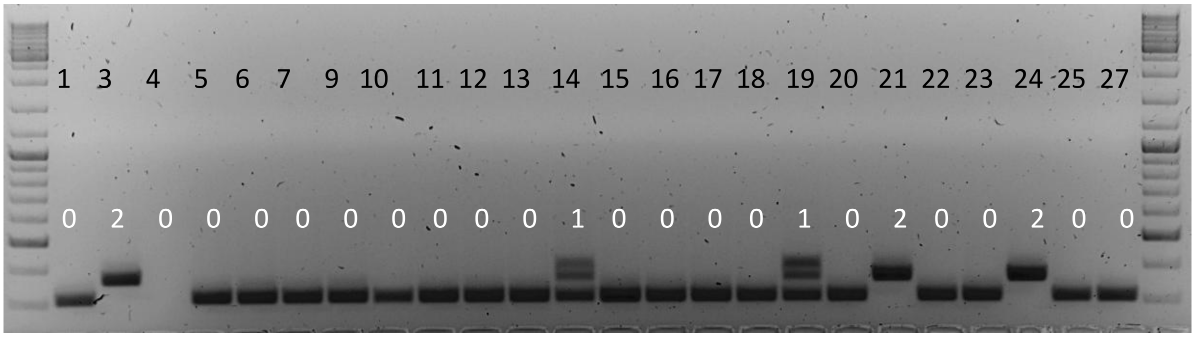

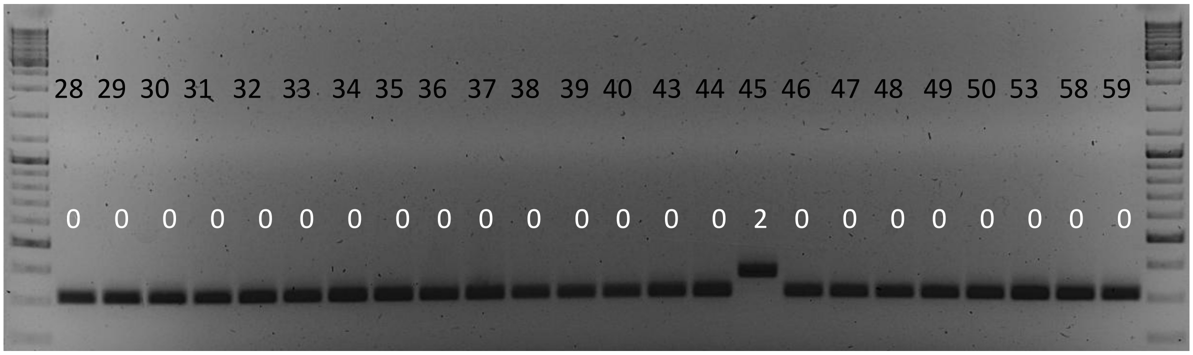

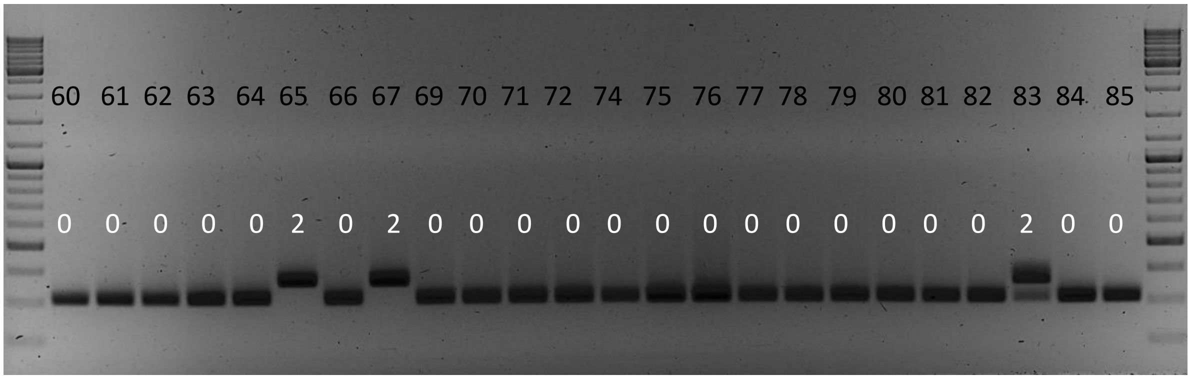

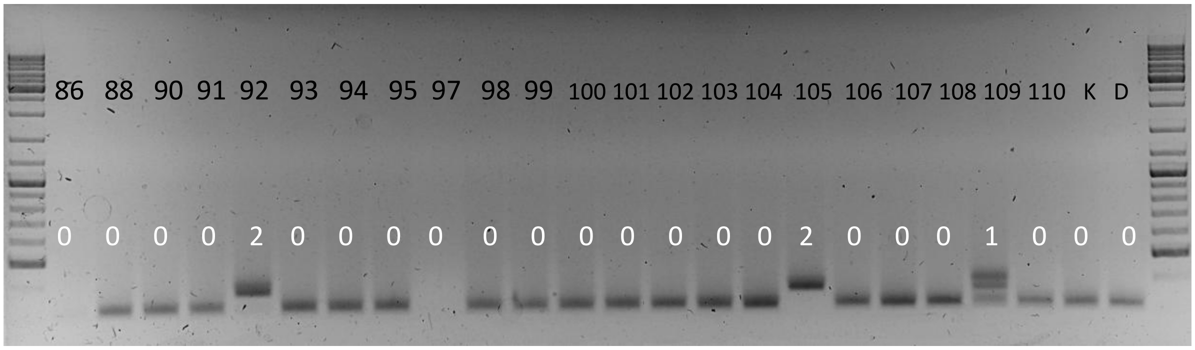

Plate 7

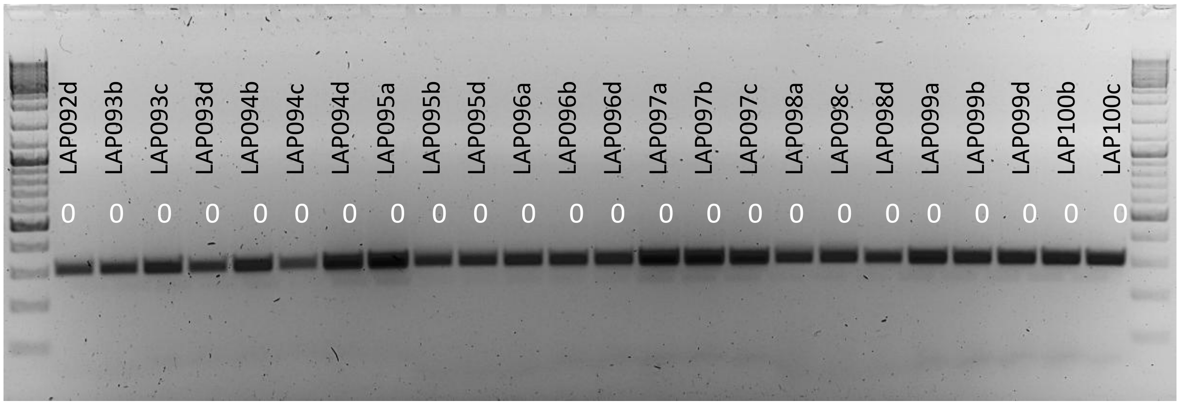
LAP092d LAP093b LAP093c LAP093d LAP094b LAP094c LAP094d LAP095a LAP095b LAP095d LAP096a LAP096b LAP096d LAP097a LAP097b LAP097c LAP098a LAP098c LAP098d LAP099a LAP099b LAP099d LAP100b LAP100c
0 0 0 0 0 0 0 0 0 0 0 0 0 0 0 0 0 0 0 0 0 0 0 0

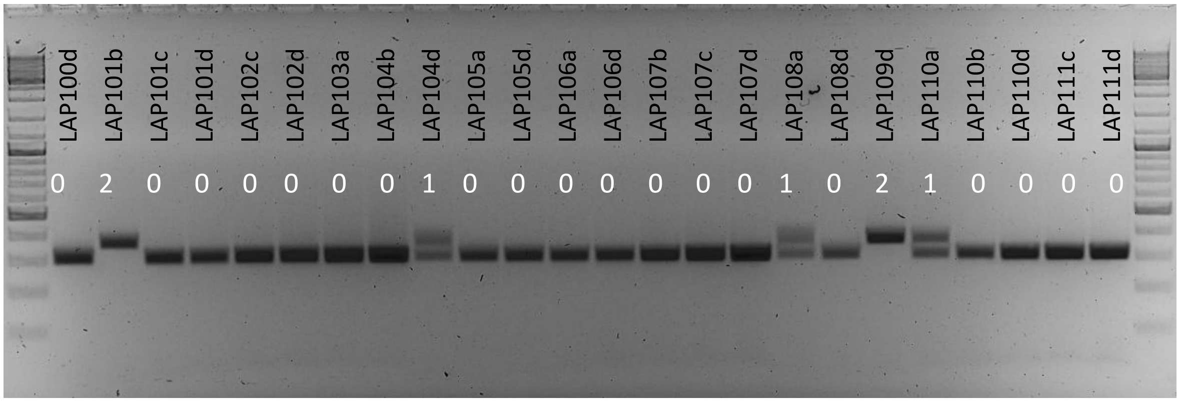
LAP100d LAP101b LAP101c LAP101d LAP102c LAP102d LAP103a LAP104b LAP104d LAP105a LAP105d LAP106a LAP106d LAP107b LAP107c LAP107d LAP108a LAP108d LAP109d LAP110a LAP110b LAP110d LAP111c LAP111d
0 2 0 0 0 0 0 0 1 0 0 0 0 0 0 0 1 0 2 1 0 0 0 0

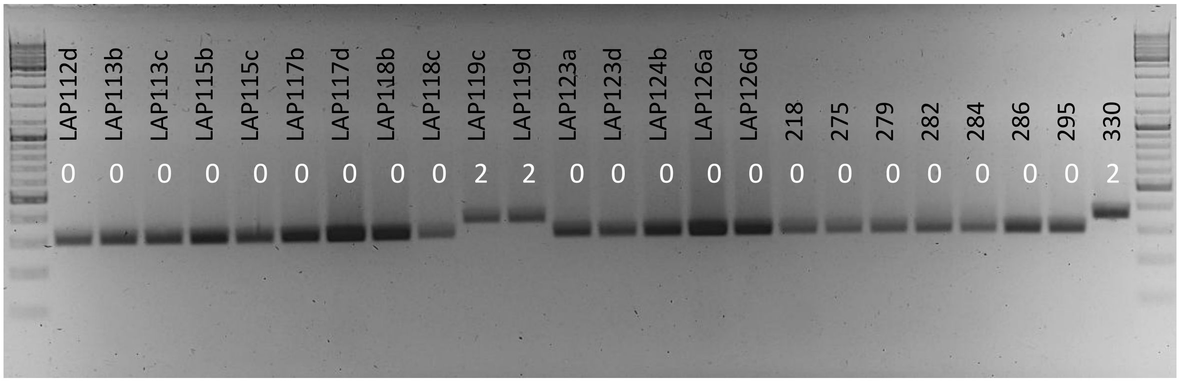
LAP112d LAP113b LAP113c LAP115b LAP115c LAP117b LAP117d LAP118b LAP118c LAP119c LAP119d LAP123a LAP123d LAP124b LAP126a LAP126d 218 275 279 282 284 286 295 330
0 0 0 0 0 0 0 0 0 2 2 0 0 0 0 0 0 0 0 0 0 0 0 2

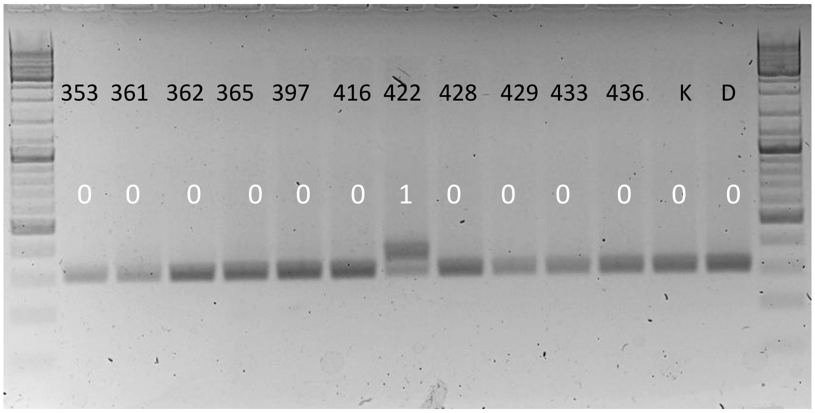
353 361 362 365 397 416 422 428 429 433 436 K D
0 0 0 0 0 0 1 0 0 0 0 0 0

Repeat

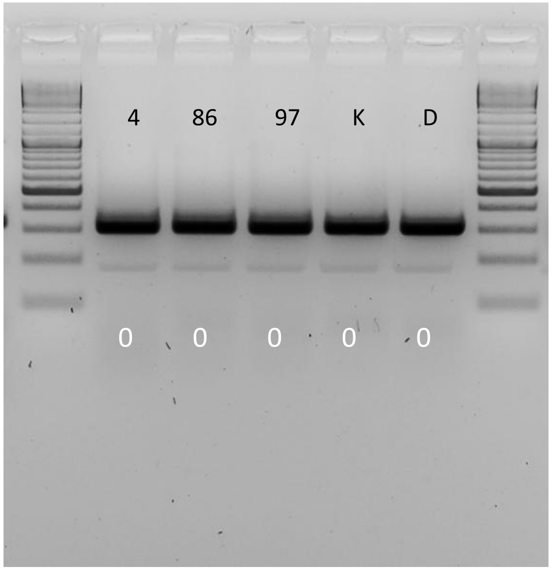
4
86
97
K
D
0 0 0 0 0

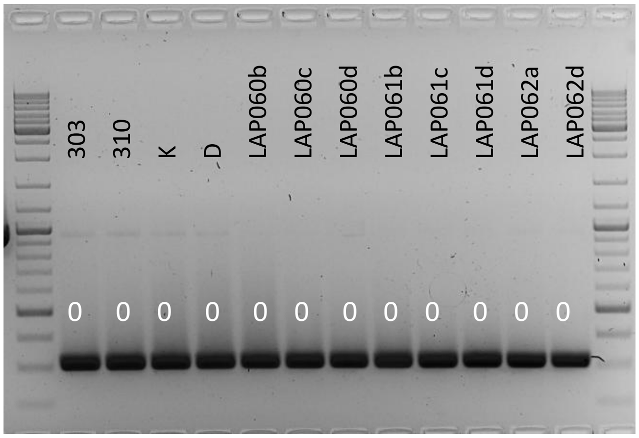
303
310
K
D
LAP060b
LAP060c
LAP060d
LAP061b
LAP061c
LAP061d
LAP062a
LAP062d
0 0 0 0 0 0 0 0 0 0 0 0

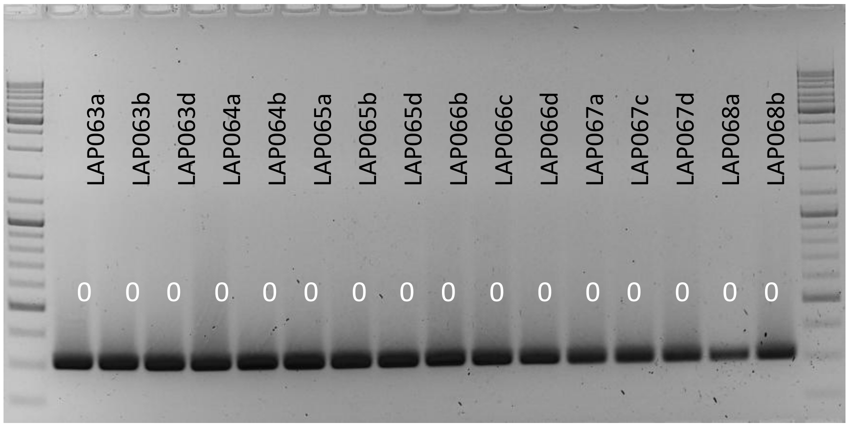
LAP063a
LAP063b
LAP063d
LAP064a
LAP064b
LAP065a
LAP065b
LAP065d
LAP066b
LAP066c
LAP066d
LAP067a
LAP067c
LAP067d
LAP068a
LAP068b
0 0 0 0 0 0 0 0 0 0 0 0 0 0 0 0

PR_06

PRFT1a_F2b TCTCAATAGCTAGGTTCAAGTGCAT

PRFTa1_R2c ACCAGAAAGAAGTTGTGTTATGACT

Plate 1

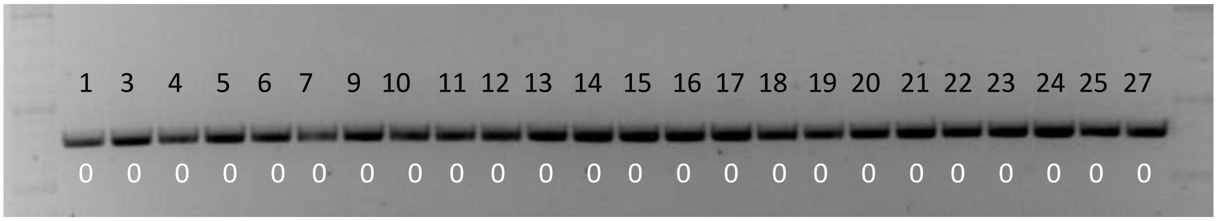

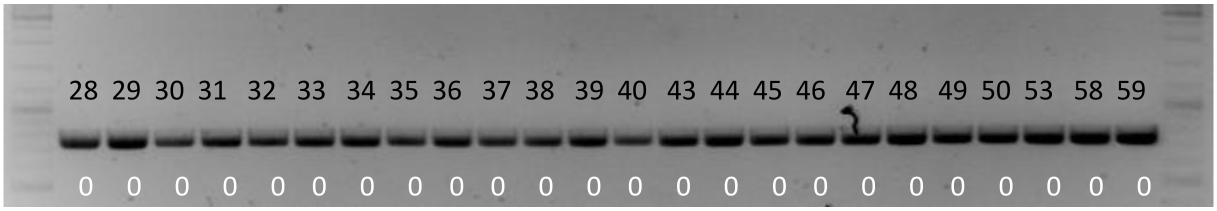

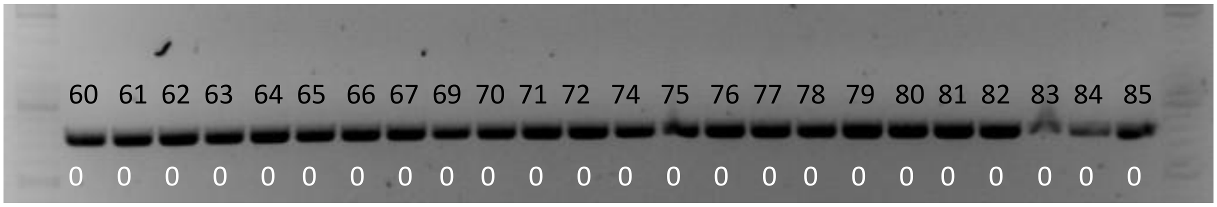

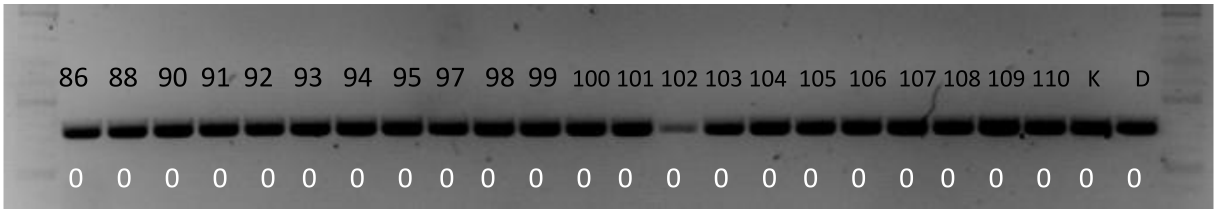

Plate 7

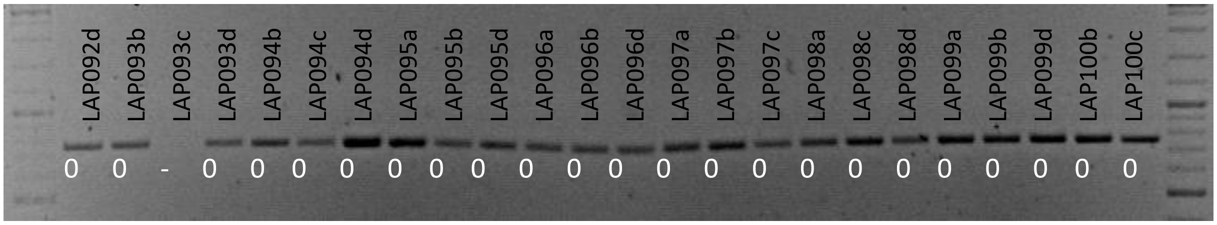
LAP092d LAP093b LAP093c LAP093d LAP094b LAP094c LAP094d LAP095a LAP095b LAP095d LAP096a LAP096b LAP096d LAP097a LAP097b LAP097c LAP098a LAP098c LAP098d LAP099a LAP099b LAP099d LAP100b LAP100c
0 0 - 0 0 0 0 0 0 0 0 0 0 0 0 0 0 0 0 0 0 0 0 0

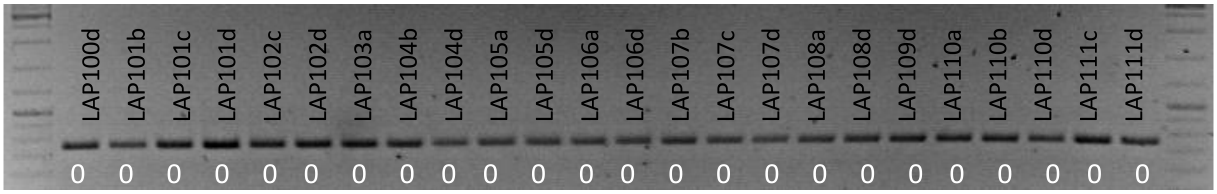
LAP100d LAP101b LAP101c LAP101d LAP102c LAP102d LAP103a LAP104b LAP104d LAP105a LAP105d LAP106a LAP106d LAP107b LAP107c LAP107d LAP108a LAP108d LAP109d LAP110a LAP110b LAP110d LAP111c LAP111d
0 0 0 0 0 0 0 0 0 0 0 0 0 0 0 0 0 0 0 0 0 0 0 0

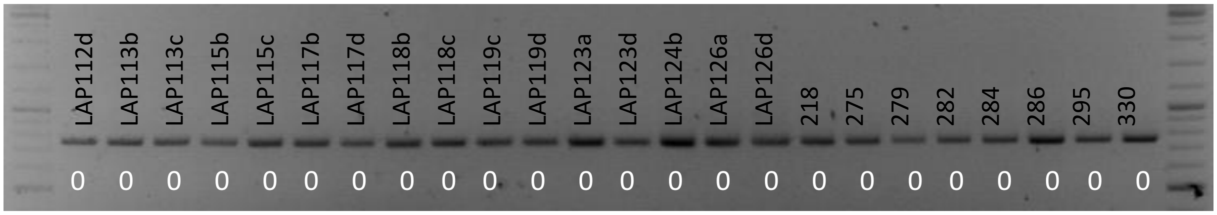
LAP112d LAP113b LAP113c LAP115b LAP115c LAP117b LAP117d LAP118b LAP118c LAP119c LAP119d LAP123a LAP123d LAP124b LAP126a LAP126d 218 275 279 282 284 286 295 330
0 0 0 0 0 0 0 0 0 0 0 0 0 0 0 0 0 0 0 0 0 0 0 0

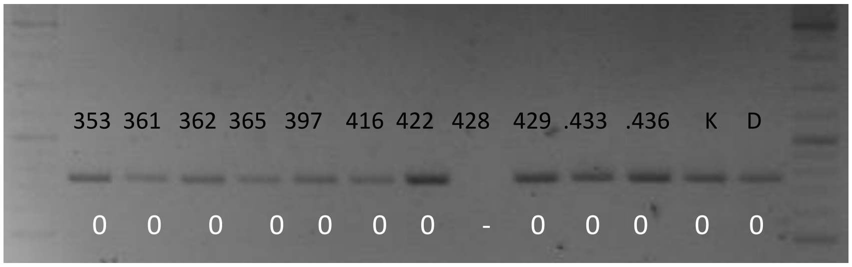
353 361 362 365 397 416 422 428 429 .433 .436 K D
0 0 0 0 0 0 0 - 0 0 0 0 0

PR_07

PRFTA1F3 CATGTTCGACTACCCCTCTCA

PRFTA1R2 CCATGAAGTCCGATTCCTTTTGA

Plate 1

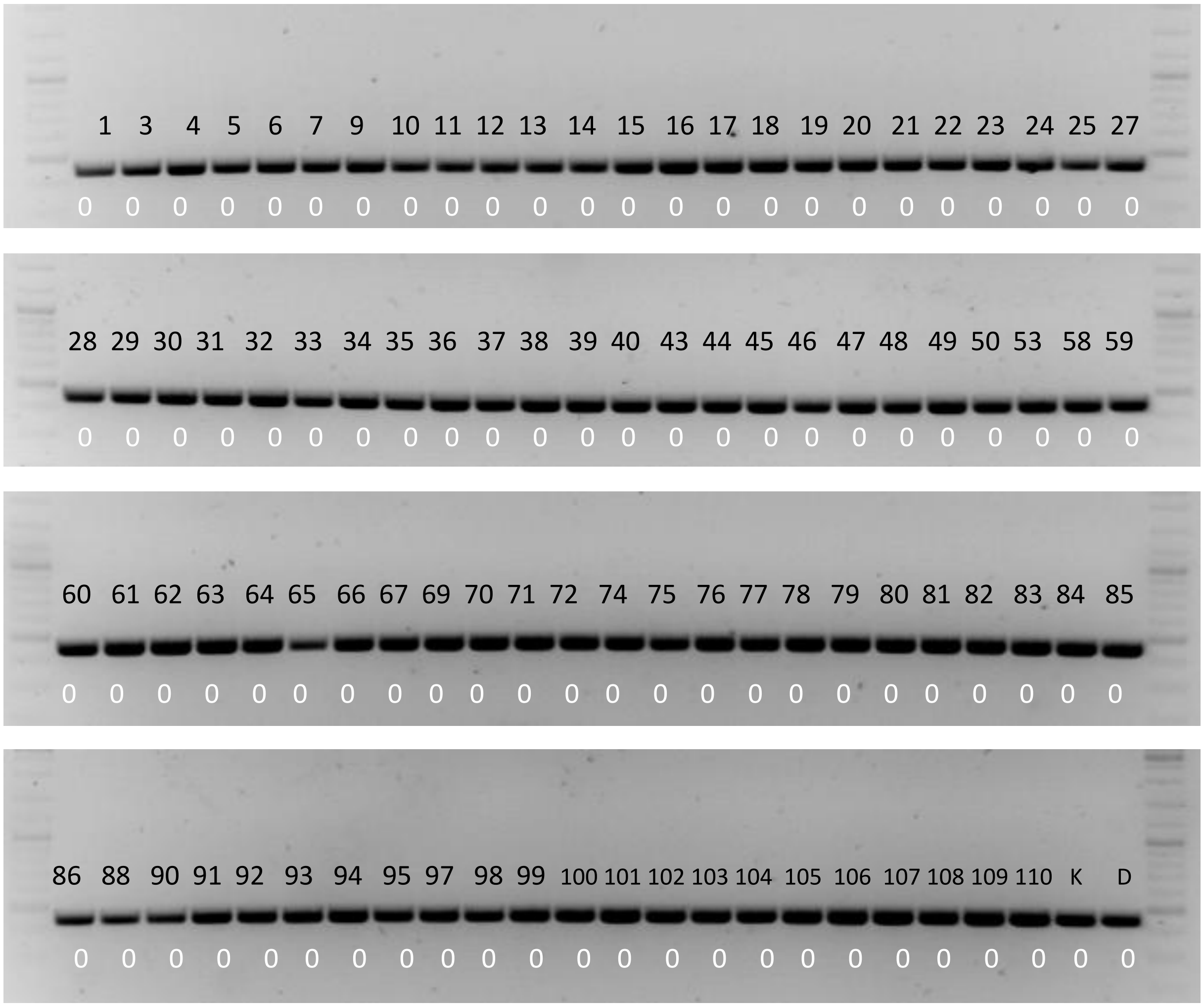

Plate 7

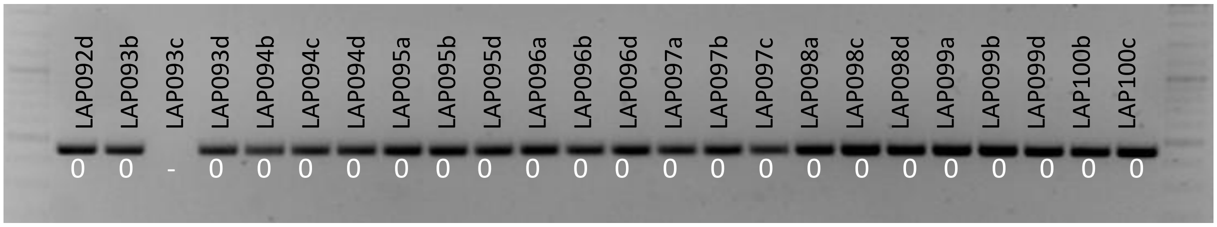
LAP092d LAP093b LAP093c LAP093d LAP094b LAP094c LAP094d LAP095a LAP095b LAP095d LAP096a LAP096b LAP096d LAP097a LAP097b LAP097c LAP098a LAP098c LAP098d LAP099a LAP099b LAP099d LAP100b LAP100c
0 0 - 0 0 0 0 0 0 0 0 0 0 0 0 0 0 0 0 0 0 0 0 0

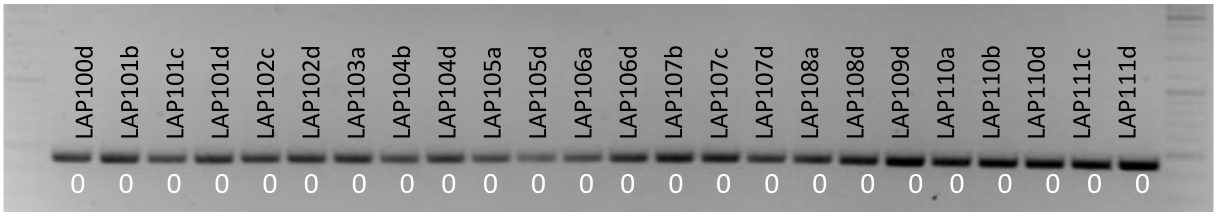
LAP100d LAP101b LAP101c LAP101d LAP102c LAP102d LAP103a LAP104b LAP104d LAP105a LAP105d LAP106a LAP106d LAP107b LAP107c LAP107d LAP108a LAP108d LAP109d LAP110a LAP110b LAP110d LAP111c LAP111d
0 0 0 0 0 0 0 0 0 0 0 0 0 0 0 0 0 0 0 0 0 0 0 0

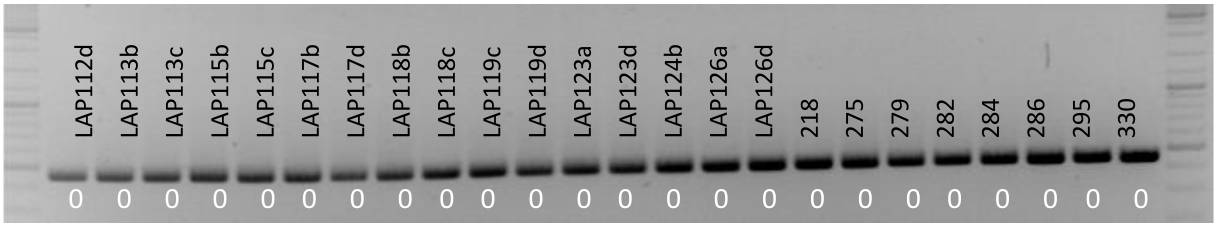
LAP112d LAP113b LAP113c LAP115b LAP115c LAP117b LAP117d LAP118b LAP118c LAP119c LAP119d LAP123a LAP123d LAP124b LAP126a LAP126d 218 275 279 282 284 286 295 330
0 0 0 0 0 0 0 0 0 0 0 0 0 0 0 0 0 0 0 0 0 0 0 0

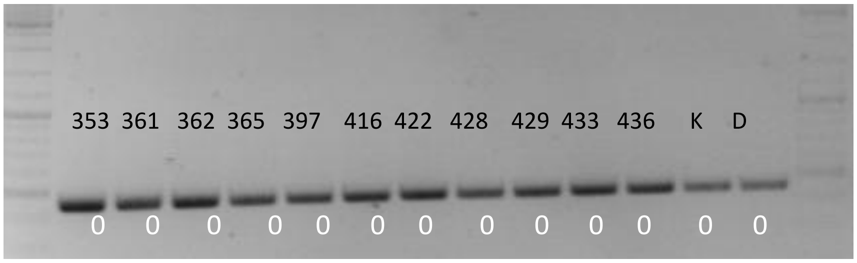
353 361 362 365 397 416 422 428 429 433 436 K D
0 0 0 0 0 0 0 0 0 0 0 0 0

PR_08

PRFTA1F3 CATGTTCGACTACCCCTCTCA

PRFTa1_R3b ACAGGTTGTGTATTGAGCGTAGAAG

Plate 1

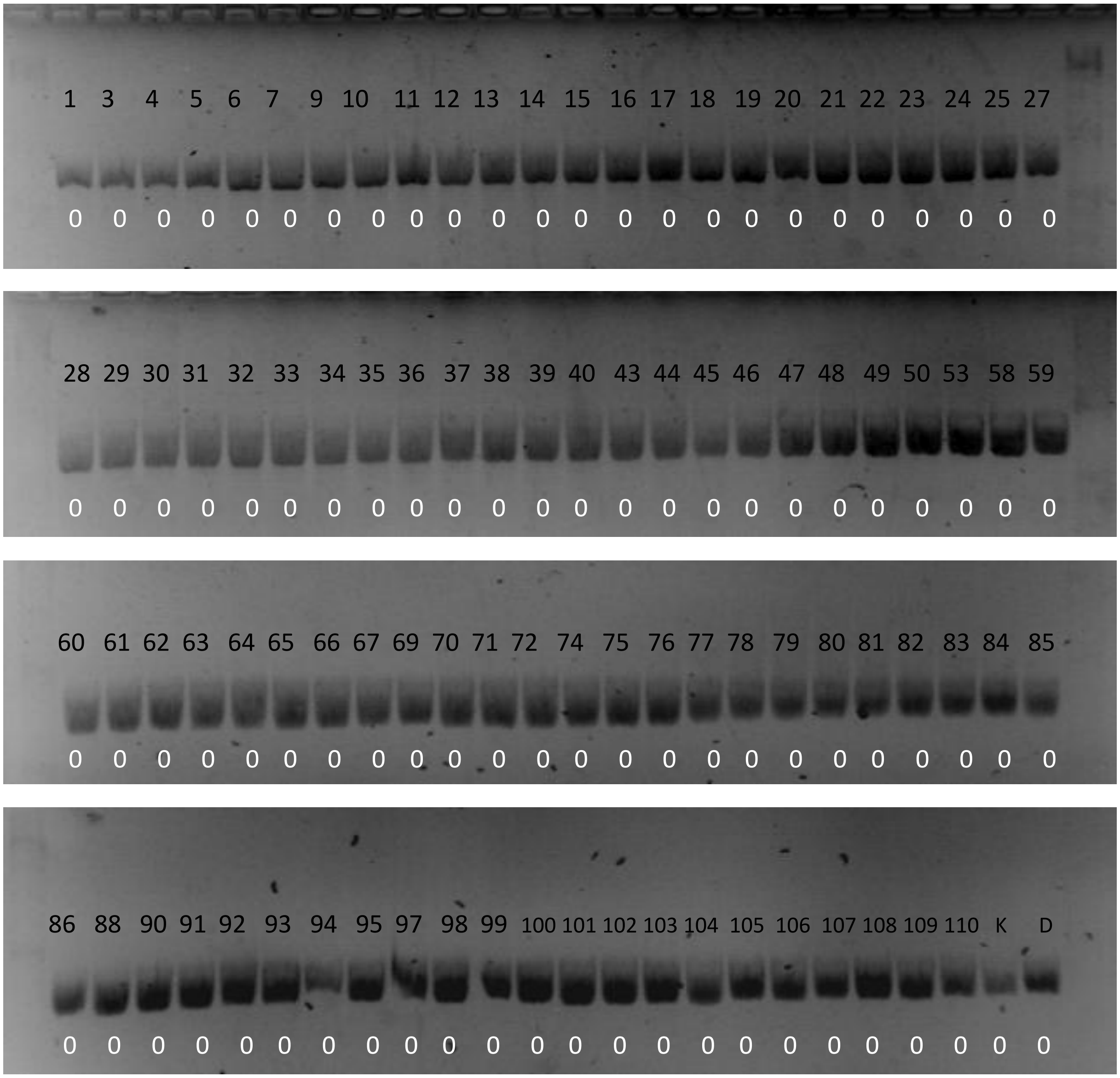

Plate 7

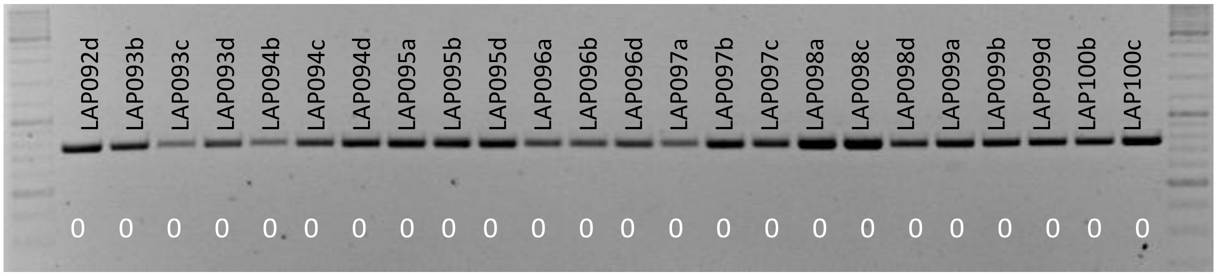
LAP092d LAP093b LAP093c LAP093d LAP094b LAP094c LAP094d LAP095a LAP095b LAP095d LAP096a LAP096b LAP096d LAP097a LAP097b LAP097c LAP098a LAP098c LAP098d LAP099a LAP099b LAP099d LAP100b LAP100c
0 0 0 0 0 0 0 0 0 0 0 0 0 0 0 0 0 0 0 0 0 0 0 0

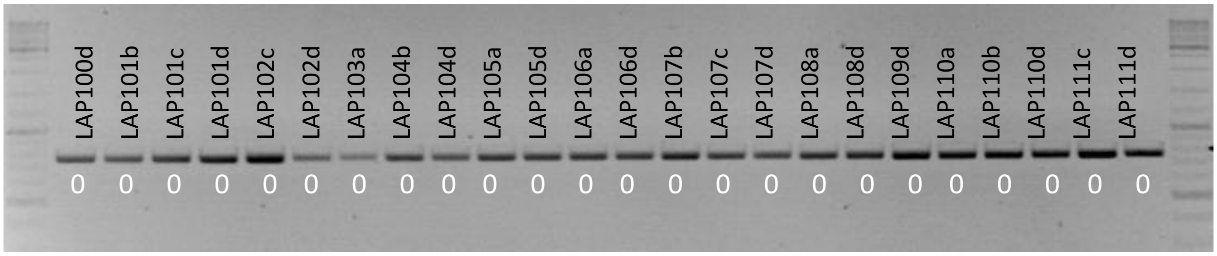
LAP100d LAP101b LAP101c LAP101d LAP102c LAP102d LAP103a LAP104b LAP104d LAP105a LAP105d LAP106a LAP106d LAP107b LAP107c LAP107d LAP108a LAP108d LAP109d LAP110a LAP110b LAP110d LAP111c LAP111d
0 0 0 0 0 0 0 0 0 0 0 0 0 0 0 0 0 0 0 0 0 0 0 0

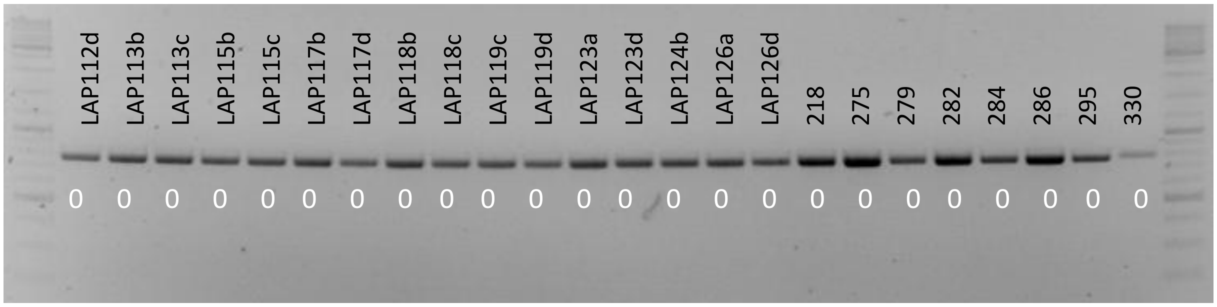
LAP112d LAP113b LAP113c LAP115b LAP115c LAP117b LAP117d LAP118b LAP118c LAP119c LAP119d LAP123a LAP123d LAP124b LAP126a LAP126d 218 275 279 282 284 286 295 330
0 0 0 0 0 0 0 0 0 0 0 0 0 0 0 0 0 0 0 0 0 0 0 0

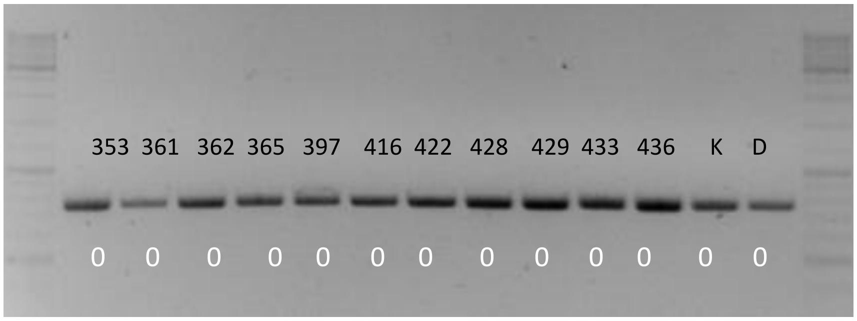
353 361 362 365 397 416 422 428 429 433 436 K D
0 0 0 0 0 0 0 0 0 0 0 0 0

PR_09

PRFTa1_F3b GTAAGAGTGTGTGTCTTTGGTTTGT

PRFTa1_R3c TGGTGACACATGTTATTGGATATGC

Plate 1

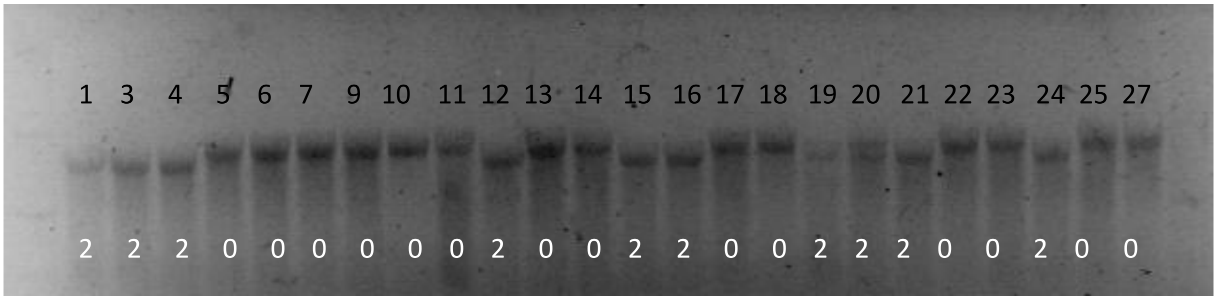

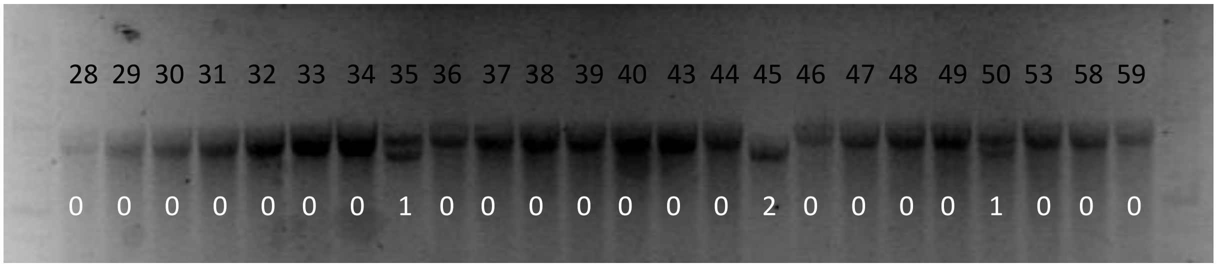

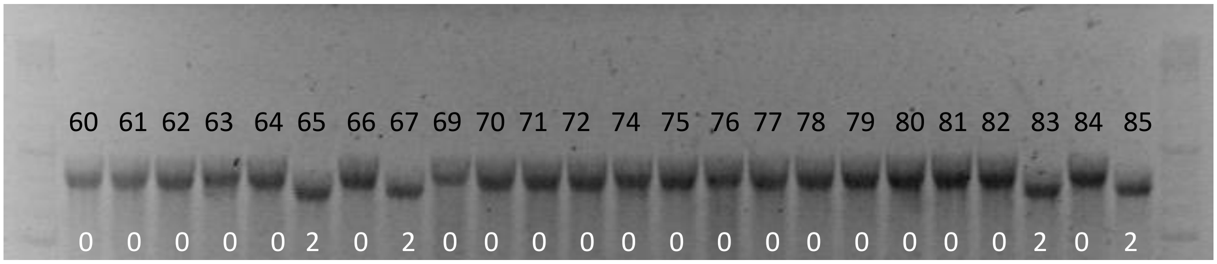

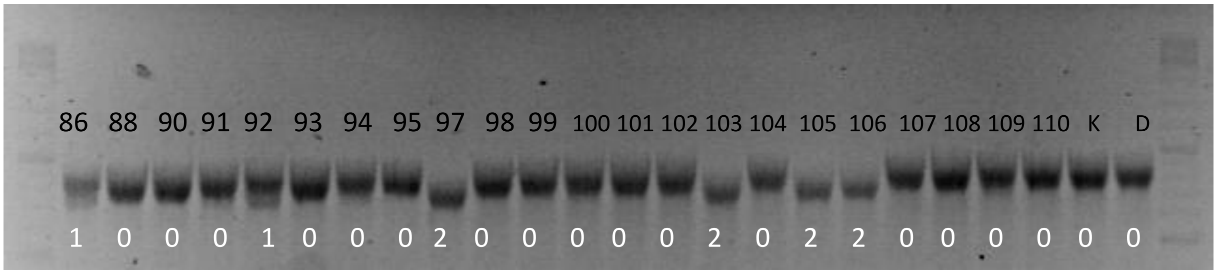

Plate 7

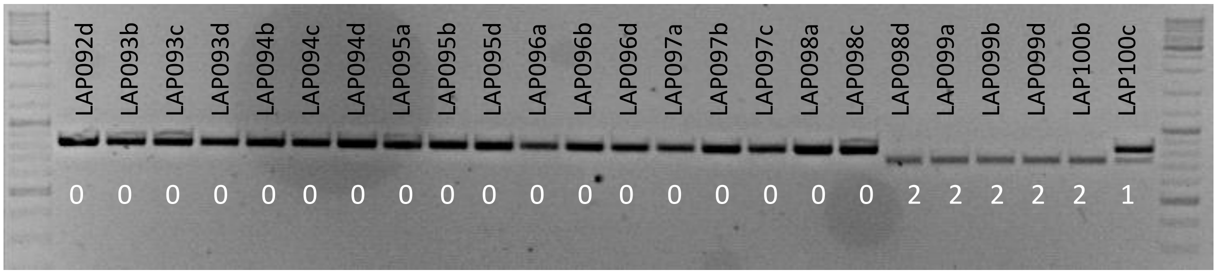

LAP092d LAP093b LAP093c LAP093d LAP094b LAP094c LAP094d LAP095a LAP095b LAP095d LAP096a LAP096b LAP096d LAP097a LAP097b LAP097c LAP098a LAP098c LAP098d LAP099a LAP099b LAP099d LAP100b LAP100c
0 0 0 0 0 0 0 0 0 0 0 0 0 0 0 0 0 0 2 2 2 2 2 1

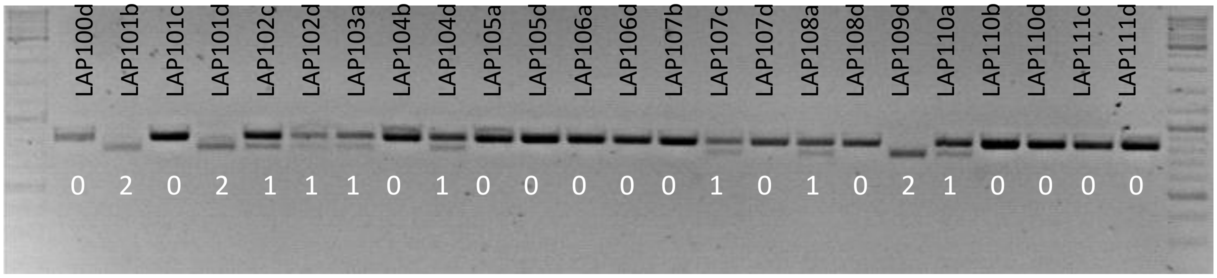

LAP100d LAP101b LAP101c LAP101d LAP102c LAP102d LAP103a LAP104b LAP104d LAP105a LAP105d LAP106a LAP106d LAP107b LAP107c LAP107d LAP108a LAP108d LAP109d LAP110a LAP110b LAP110d LAP111c LAP111d
0 2 0 2 1 1 1 0 1 0 0 0 0 0 1 0 1 0 2 1 0 0 0 0

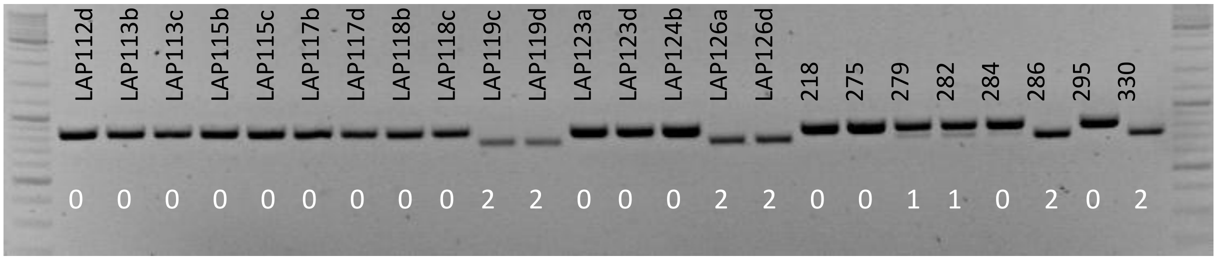

LAP112d LAP113b LAP113c LAP115b LAP115c LAP117b LAP117d LAP118b LAP118c LAP119c LAP119d LAP123a LAP123d LAP124b LAP126a LAP126d 218 275 279 282 284 286 295 330
0 0 0 0 0 0 0 0 0 2 2 0 0 0 2 2 0 0 1 1 0 2 0 2

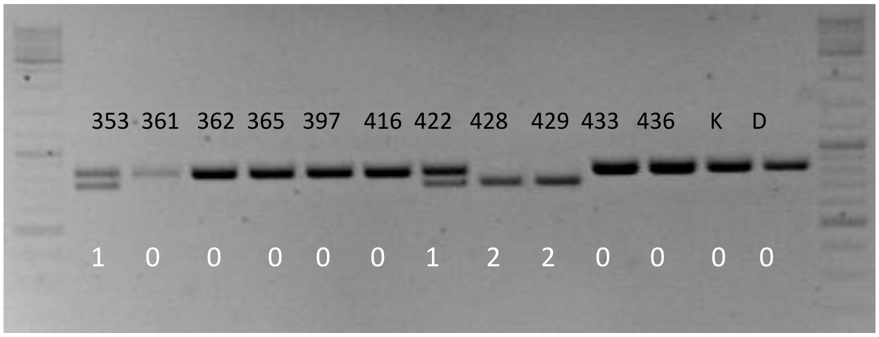

353 361 362 365 397 416 422 428 429 433 436 K D
1 0 0 0 0 0 1 2 2 0 0 0 0

PR_10

PRFTa1_F3c GAGCCACACTATCTTATTGCATACA

PRFTA1R3 TCTATCTCTTTGTTCTTTGCATGGA

Plate 1

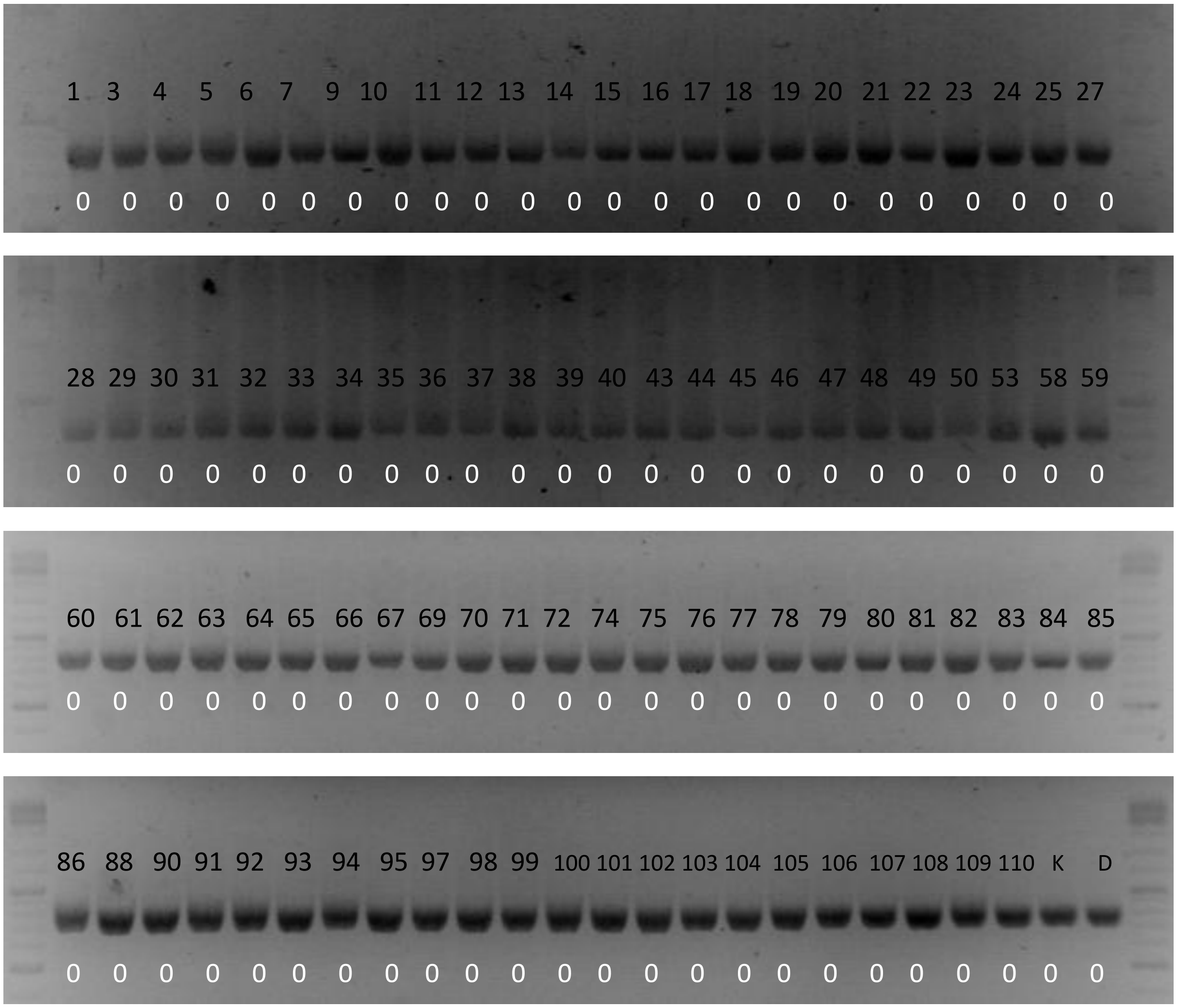

Plate 7

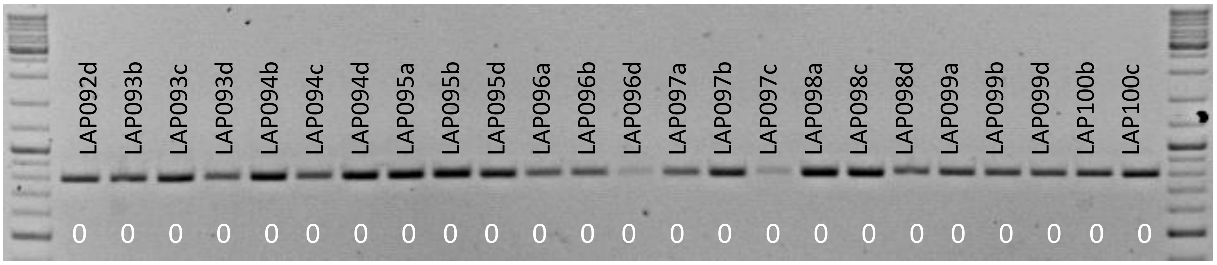
LAP092d LAP093b LAP093c LAP093d LAP094b LAP094c LAP094d LAP095a LAP095b LAP095d LAP096a LAP096b LAP096d LAP097a LAP097b LAP097c LAP098a LAP098c LAP098d LAP099a LAP099b LAP099d LAP100b LAP100c
0 0 0 0 0 0 0 0 0 0 0 0 0 0 0 0 0 0 0 0 0 0 0 0

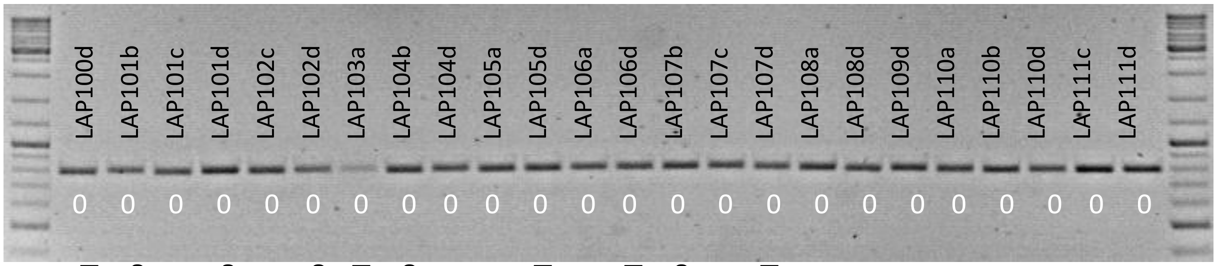
LAP100d LAP101b LAP101c LAP101d LAP102c LAP102d LAP103a LAP104b LAP104d LAP105a LAP105d LAP106a LAP106d LAP107b LAP107c LAP107d LAP108a LAP108d LAP109d LAP110a LAP110b LAP110d LAP111c LAP111d
0 0 0 0 0 0 0 0 0 0 0 0 0 0 0 0 0 0 0 0 0 0 0 0

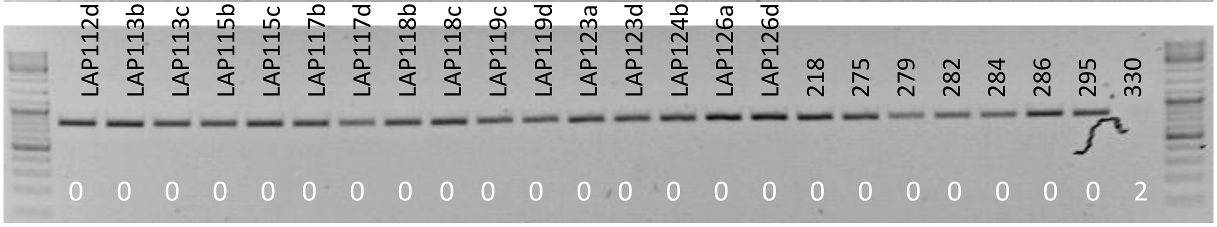
LAP112d LAP113b LAP113c LAP115b LAP115c LAP117b LAP117d LAP118b LAP118c LAP119c LAP119d LAP123a LAP123d LAP124b LAP126a LAP126d 218 275 279 282 284 286 295 330
0 0 0 0 0 0 0 0 0 0 0 0 0 0 0 0 0 0 0 0 0 0 0 2

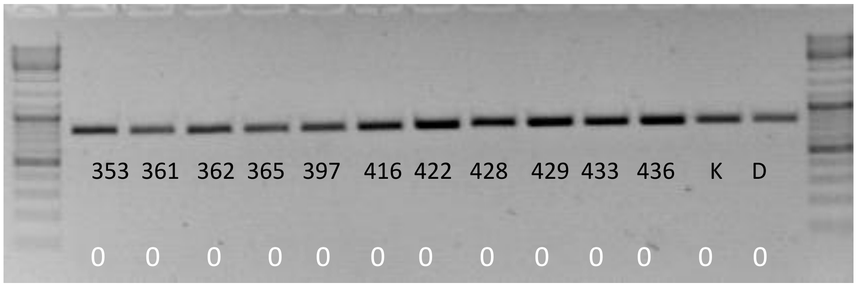
353 361 362 365 397 416 422 428 429 433 436 K D
0 0 0 0 0 0 0 0 0 0 0 0 0

PR_11

PRFTA1F4 ATGACCAAAATTGACATTCTCAGT

PRFTa1_R4b TTTACCGCTGCGTATGTTAAGAAC

Plate 1

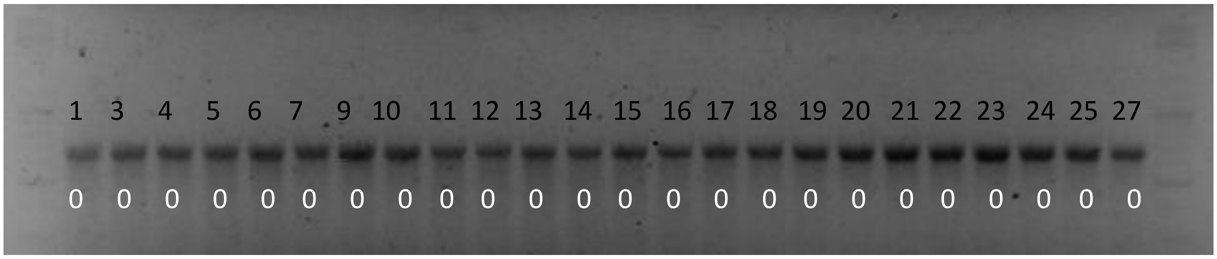

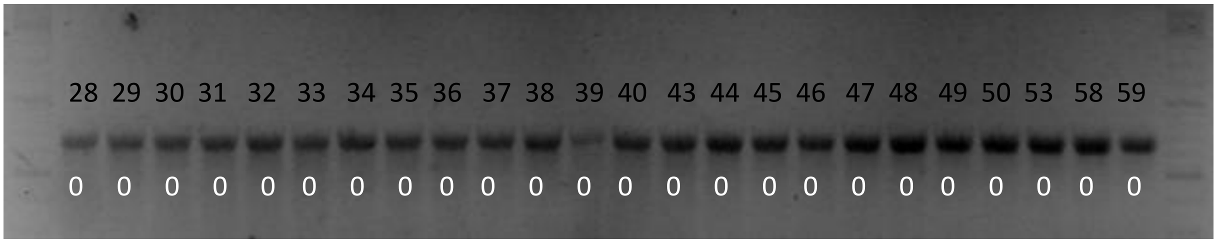

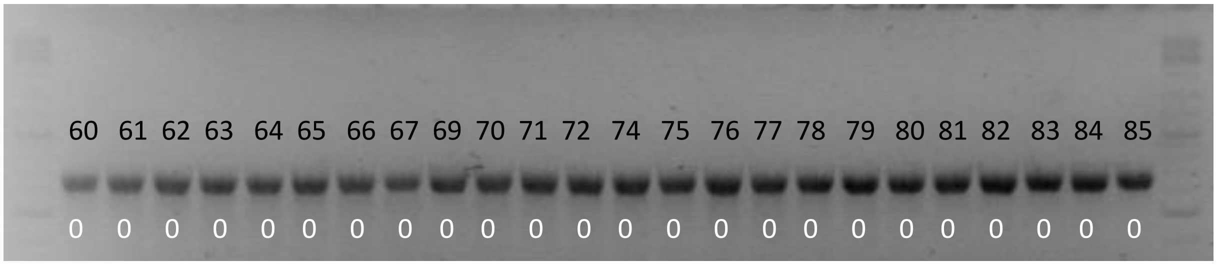

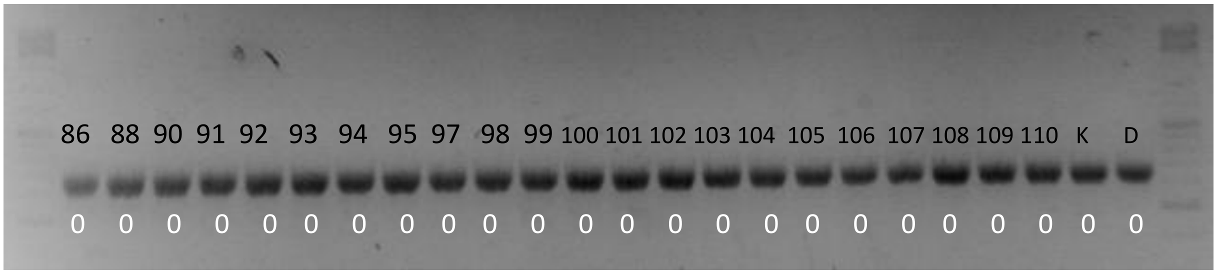

Plate 7

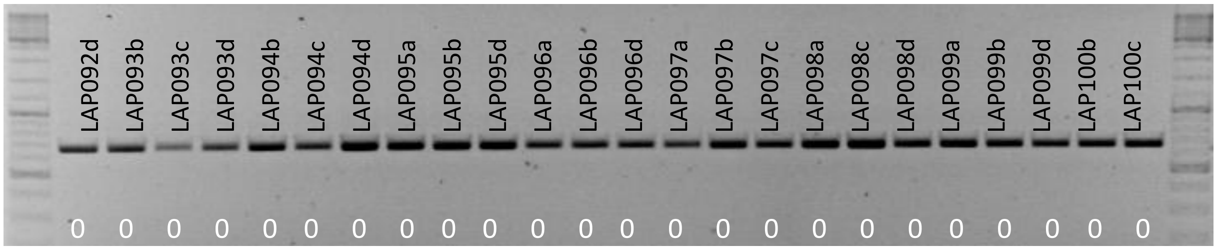
LAP092d LAP093b LAP093c LAP093d LAP094b LAP094c LAP094d LAP095a LAP095b LAP095d LAP096a LAP096b LAP096d LAP097a LAP097b LAP097c LAP098a LAP098c LAP098d LAP099a LAP099b LAP099d LAP100b LAP100c
0 0 0 0 0 0 0 0 0 0 0 0 0 0 0 0 0 0 0 0 0 0 0 0

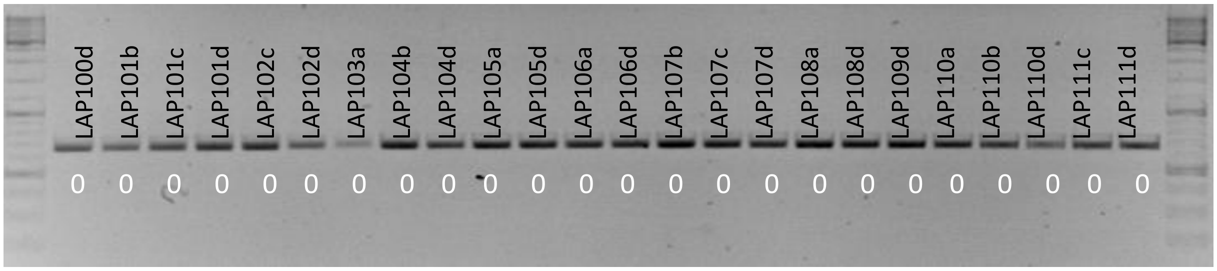
LAP100d LAP101b LAP101c LAP101d LAP102c LAP102d LAP103a LAP104b LAP104d LAP105a LAP105d LAP106a LAP106d LAP107b LAP107c LAP107d LAP108a LAP108d LAP109d LAP110a LAP110b LAP110d LAP111c LAP111d
0 0 0 0 0 0 0 0 0 0 0 0 0 0 0 0 0 0 0 0 0 0 0 0

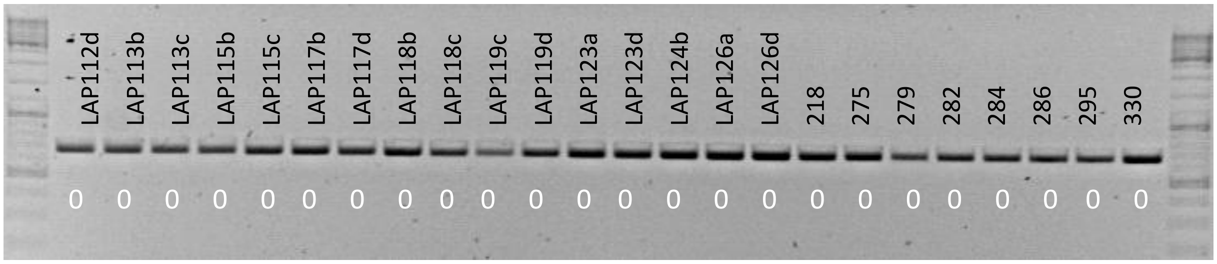
LAP112d LAP113b LAP113c LAP115b LAP115c LAP117b LAP117d LAP118b LAP118c LAP119c LAP119d LAP123a LAP123d LAP124b LAP126a LAP126d 218 275 279 282 284 286 295 330
0 0 0 0 0 0 0 0 0 0 0 0 0 0 0 0 0 0 0 0 0 0 0 0

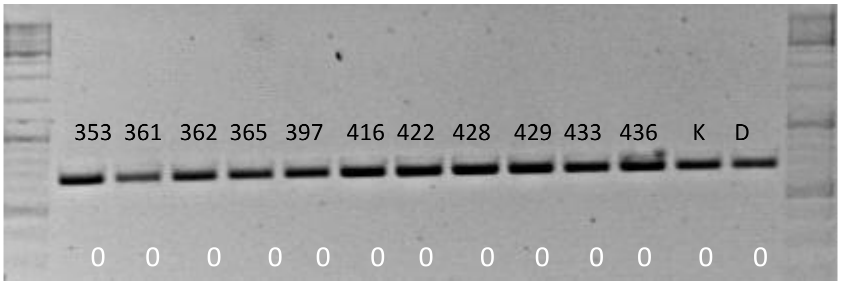
353 361 362 365 397 416 422 428 429 433 436 K D
0 0 0 0 0 0 0 0 0 0 0 0 0

PR_12

PRFTa1_F4b ATCATTTCTGAGACAATGAGATAGACG

PRFTa1_R4c TTTAATTCTTTGCTAGCACCAAGAA

Plate 1

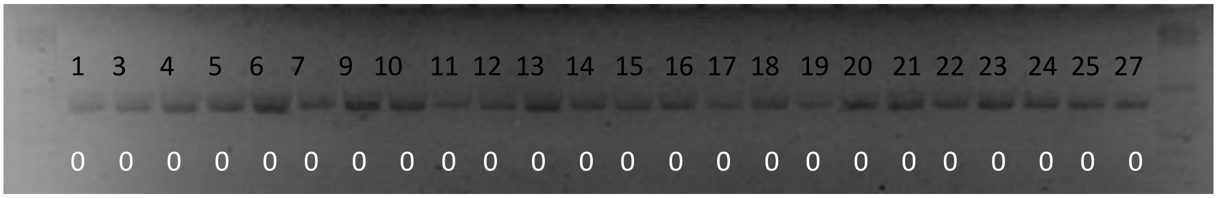

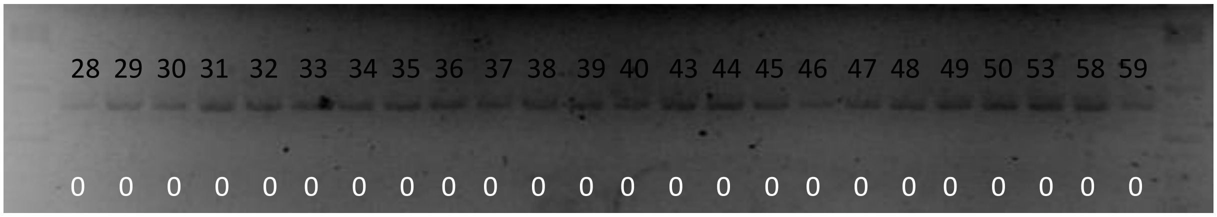

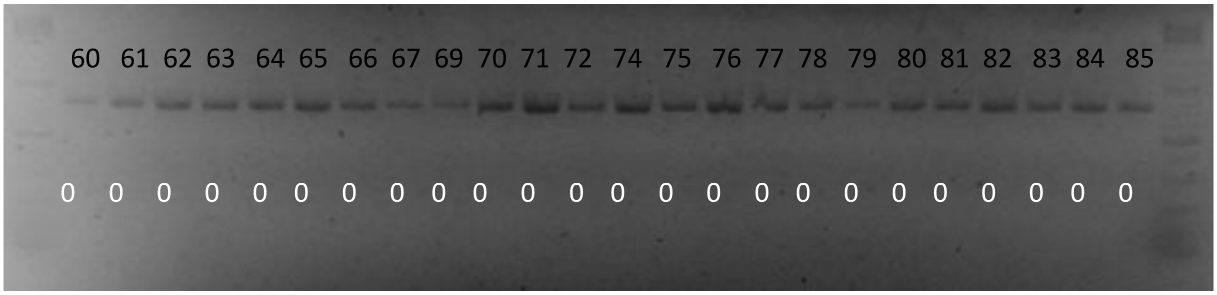

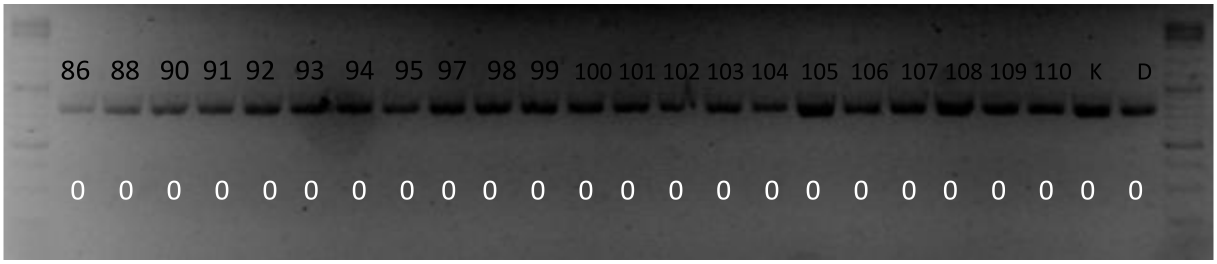

Plate 7

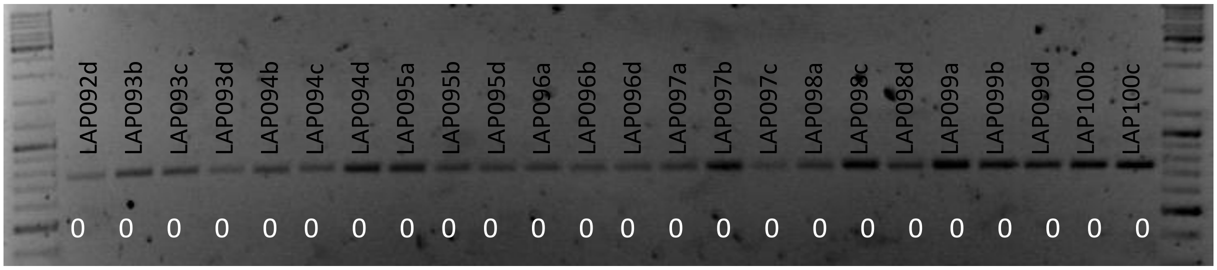
LAP092d LAP093b LAP093c LAP093d LAP094b LAP094c LAP094d LAP095a LAP095b LAP095d LAP096a LAP096b LAP096d LAP097a LAP097b LAP097c LAP098a LAP098c LAP098d LAP099a LAP099b LAP099d LAP100b LAP100c
0 0 0 0 0 0 0 0 0 0 0 0 0 0 0 0 0 0 0 0 0 0 0 0

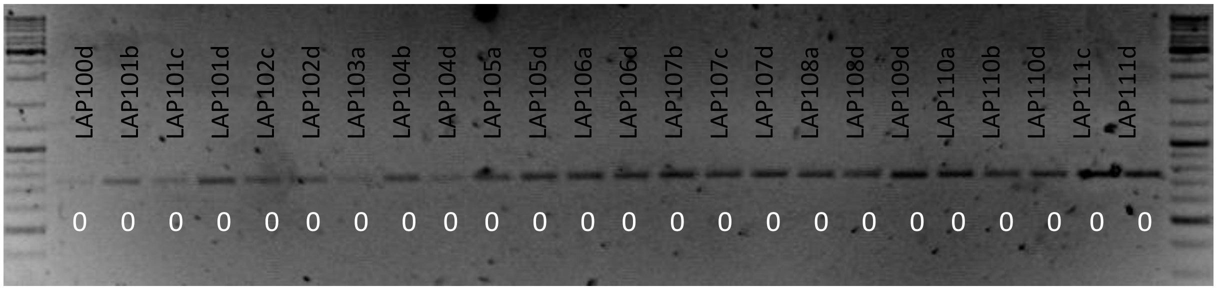
LAP100d LAP101b LAP101c LAP101d LAP102c LAP102d LAP103a LAP104b LAP104d LAP105a LAP105d LAP106a LAP106d LAP107b LAP107c LAP107d LAP108a LAP108d LAP109d LAP110a LAP110b LAP110d LAP111c LAP111d
0 0 0 0 0 0 0 0 0 0 0 0 0 0 0 0 0 0 0 0 0 0 0 0

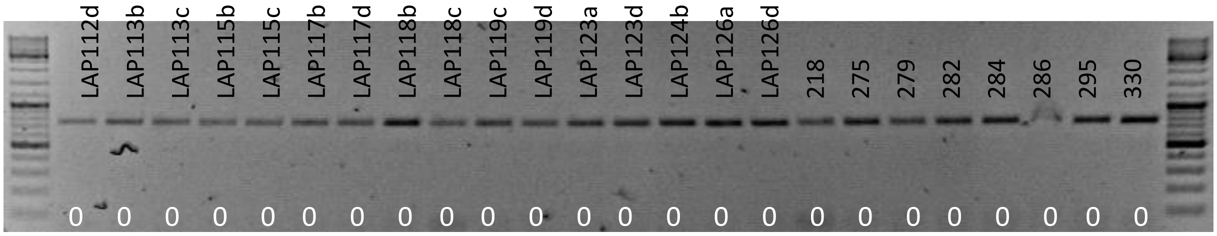
LAP112d LAP113b LAP113c LAP115b LAP115c LAP117b LAP117d LAP118b LAP118c LAP119c LAP119d LAP123a LAP123d LAP124b LAP126a LAP126d 218 275 279 282 284 286 295 330
0 0 0 0 0 0 0 0 0 0 0 0 0 0 0 0 0 0 0 0 0 0 0 0

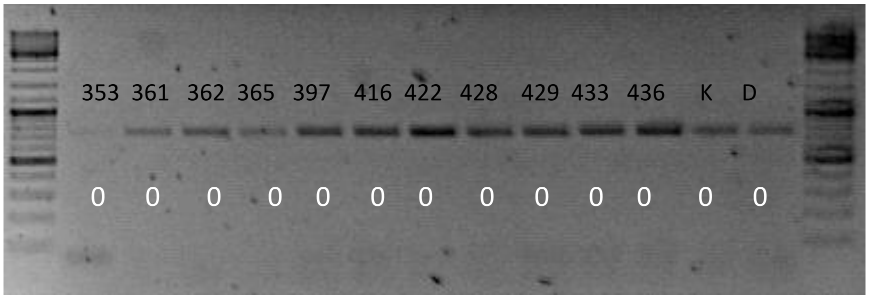
353 361 362 365 397 416 422 428 429 433 436 K D
0 0 0 0 0 0 0 0 0 0 0 0 0

PR_13

PRFTa1_F4c TTTCCGTTGAAACAATTTGCCTACC

PRFTA1R4 CTCTTGGTAATGAGTTTGAGCT

Plate 1

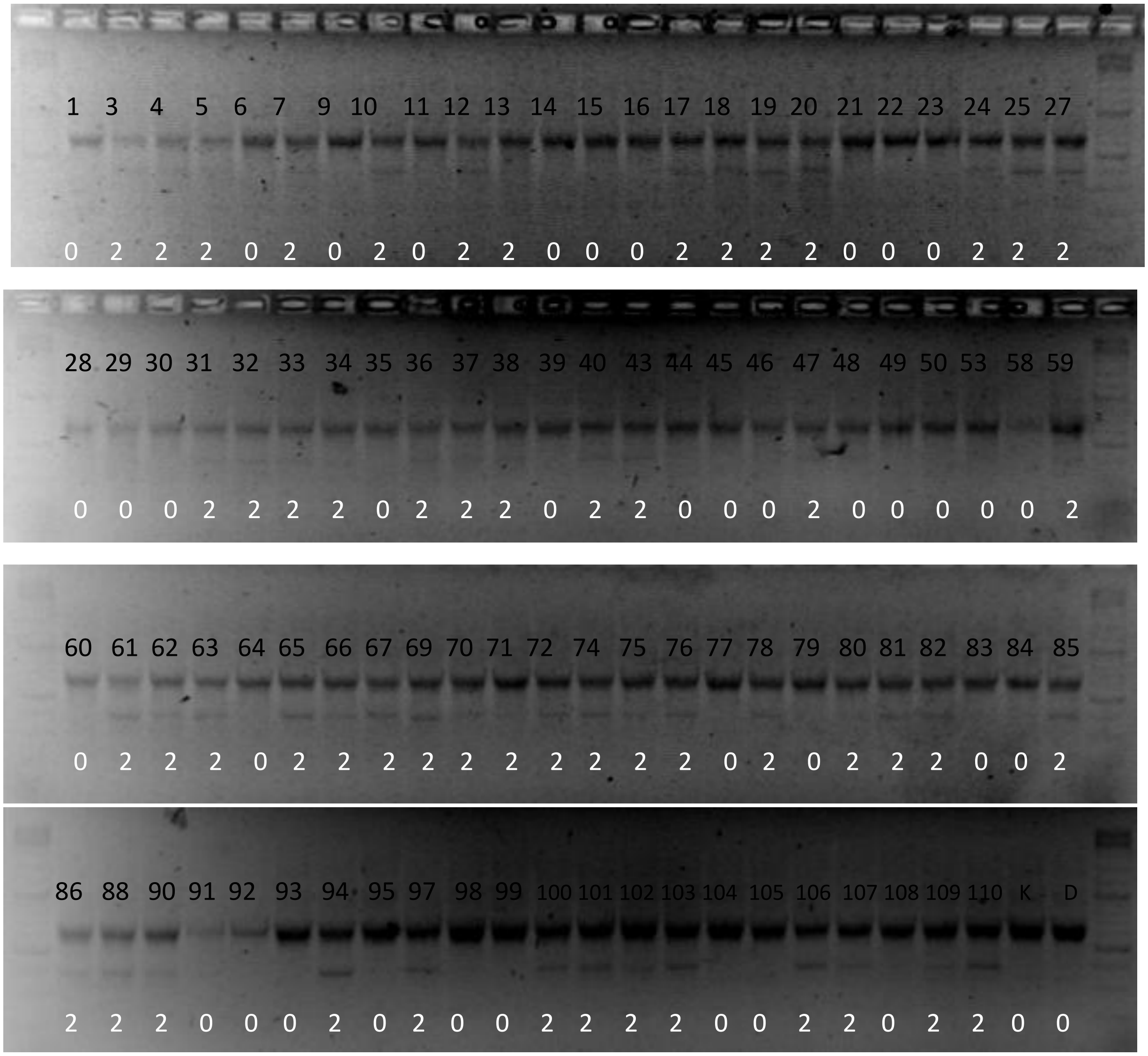

Plate 7

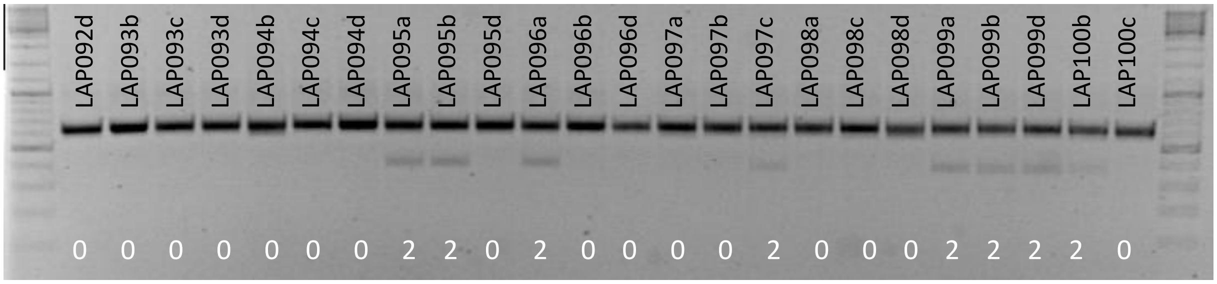
LAP092d LAP093b LAP093c LAP093d LAP094b LAP094c LAP094d LAP095a LAP095b LAP095d LAP096a LAP096b LAP096d LAP097a LAP097b LAP097c LAP098a LAP098c LAP098d LAP099a LAP099b LAP099d LAP100b LAP100c
0 0 0 0 0 0 0 2 2 0 2 0 0 0 0 2 0 0 0 2 2 2 2 0

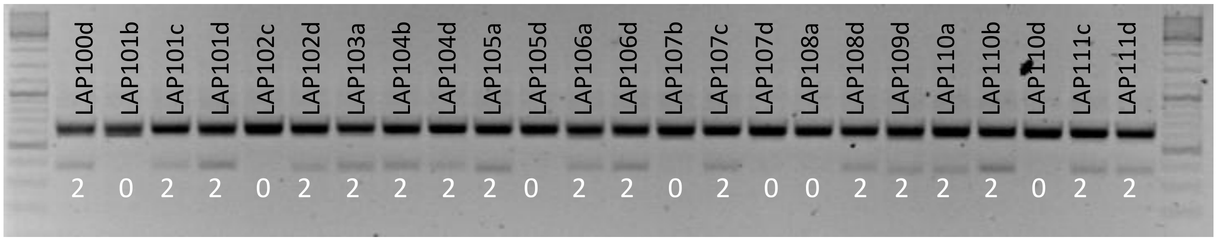
LAP100d LAP101b LAP101c LAP101d LAP102c LAP102d LAP103a LAP104b LAP104d LAP105a LAP105d LAP106a LAP106d LAP107b LAP107c LAP107d LAP108a LAP108d LAP109d LAP110a LAP110b LAP110d LAP111c LAP111d
2 0 2 2 0 2 2 2 2 2 0 2 2 0 2 0 0 2 2 2 2 0 2 2

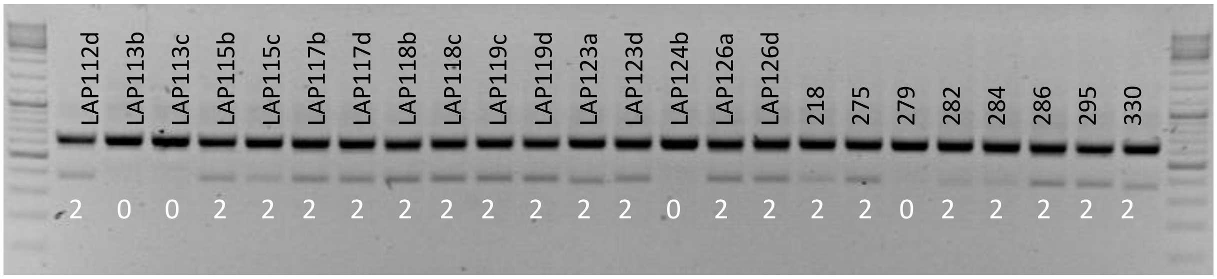
LAP112d LAP113b LAP113c LAP115b LAP115c LAP117b LAP117d LAP118b LAP118c LAP119c LAP119d LAP123a LAP123d LAP124b LAP126a LAP126d 218 275 279 282 284 286 295 330
2 0 0 2 2 2 2 2 2 2 2 2 2 0 2 2 2 2 0 2 2 2 2 2

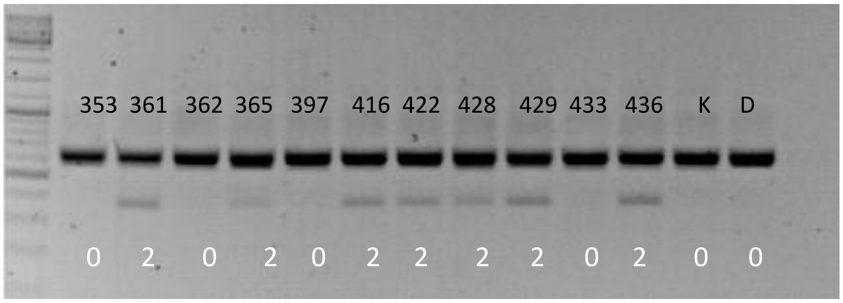
353 361 362 365 397 416 422 428 429 433 436 K D
0 2 0 2 0 2 2 2 2 0 2 0 0

PR_14

PRFTA1F5 TCTTTACAGCTCTGACTAACTTTAAA

PRFTa1_R5b AAAGTTCAACTTCTTTCCGAATGGG

Plate 1

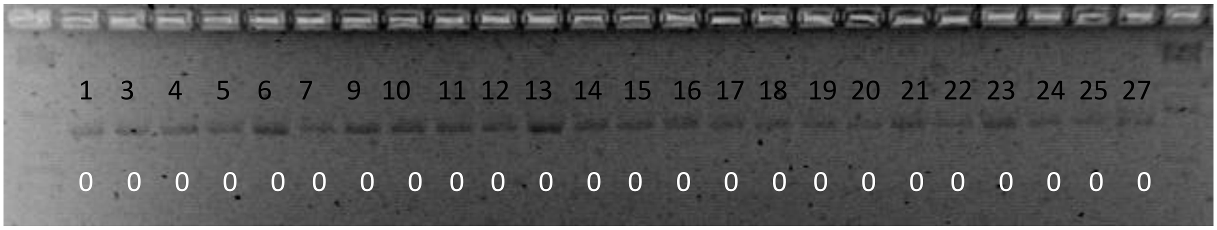

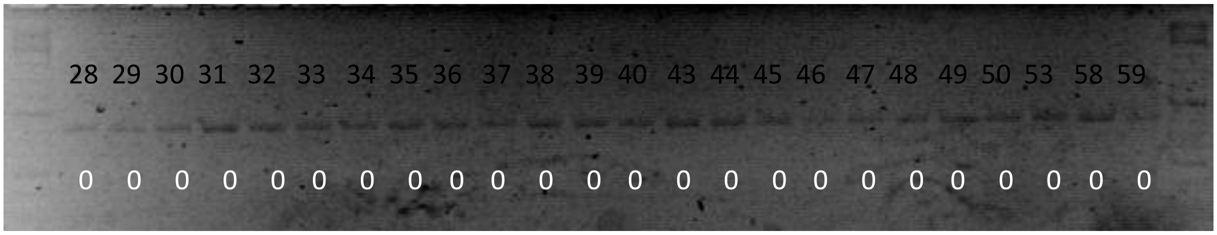

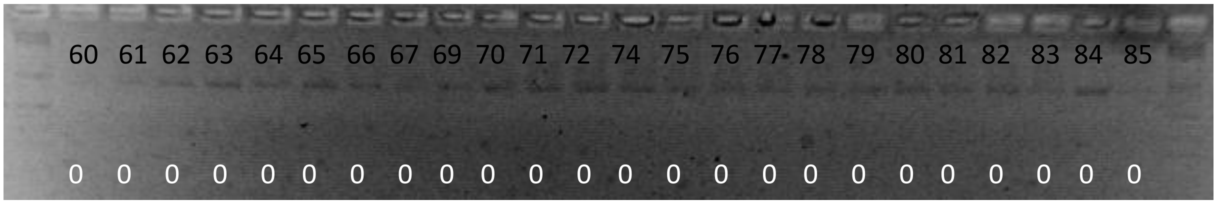

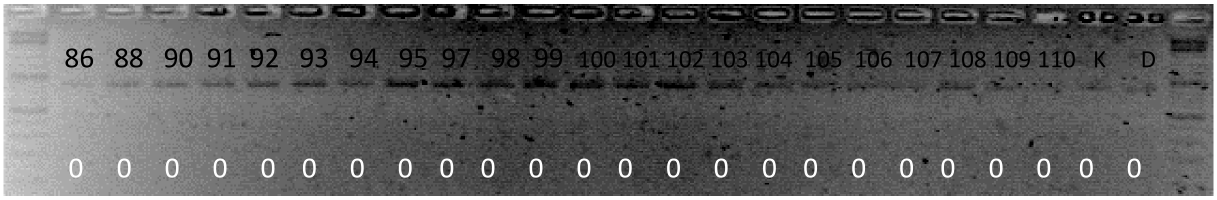

Plate 7

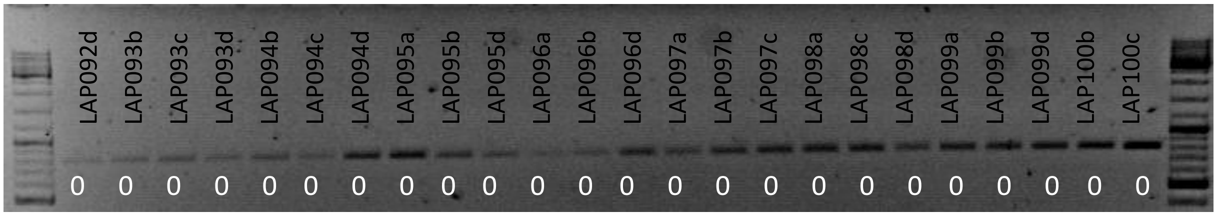
LAP092d LAP093b LAP093c LAP093d LAP094b LAP094c LAP094d LAP095a LAP095b LAP095d LAP096a LAP096b LAP096d LAP097a LAP097b LAP097c LAP098a LAP098c LAP098d LAP099a LAP099b LAP099d LAP100b LAP100c
0 0 0 0 0 0 0 0 0 0 0 0 0 0 0 0 0 0 0 0 0 0 0 0

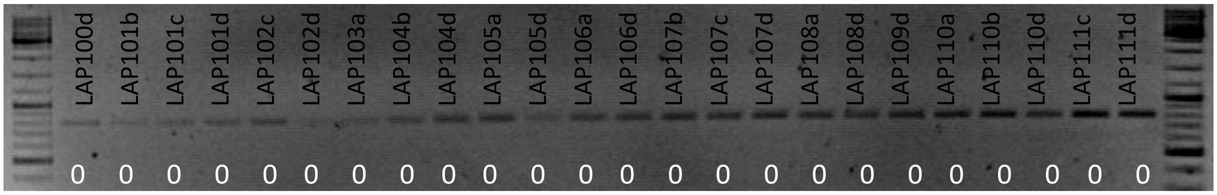
LAP100d LAP101b LAP101c LAP101d LAP102c LAP102d LAP103a LAP104b LAP104d LAP105a LAP105d LAP106a LAP106d LAP107b LAP107c LAP107d LAP108a LAP108d LAP109d LAP110a LAP110b LAP110d LAP111c LAP111d
0 0 0 0 0 0 0 0 0 0 0 0 0 0 0 0 0 0 0 0 0 0 0 0

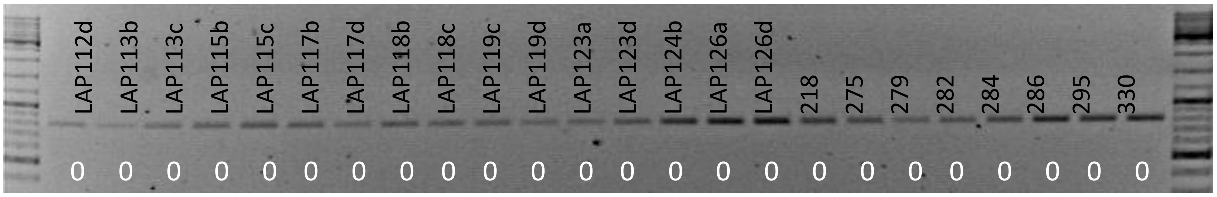
LAP112d LAP113b LAP113c LAP115b LAP115c LAP117b LAP117d LAP118b LAP118c LAP119c LAP119d LAP123a LAP123d LAP124b LAP126a LAP126d 218 275 279 282 284 286 295 330
0 0 0 0 0 0 0 0 0 0 0 0 0 0 0 0 0 0 0 0 0 0 0 0

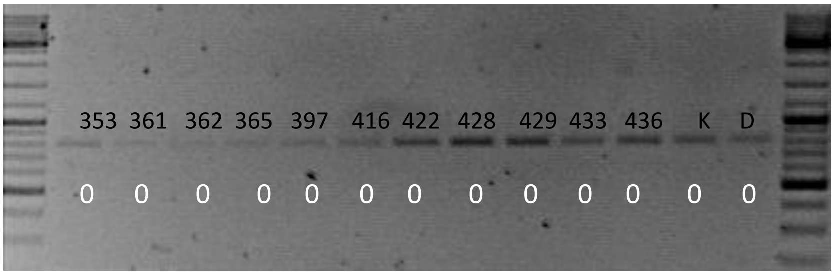
353 361 362 365 397 416 422 428 429 433 436 K D
0 0 0 0 0 0 0 0 0 0 0 0 0

PR_15

PRFTa1_F5b TCAAGAAGAATTACATTTGACCCGT

PRFTA1R5 CACAACCATTGCTAACTTCTCGA

Plate 1

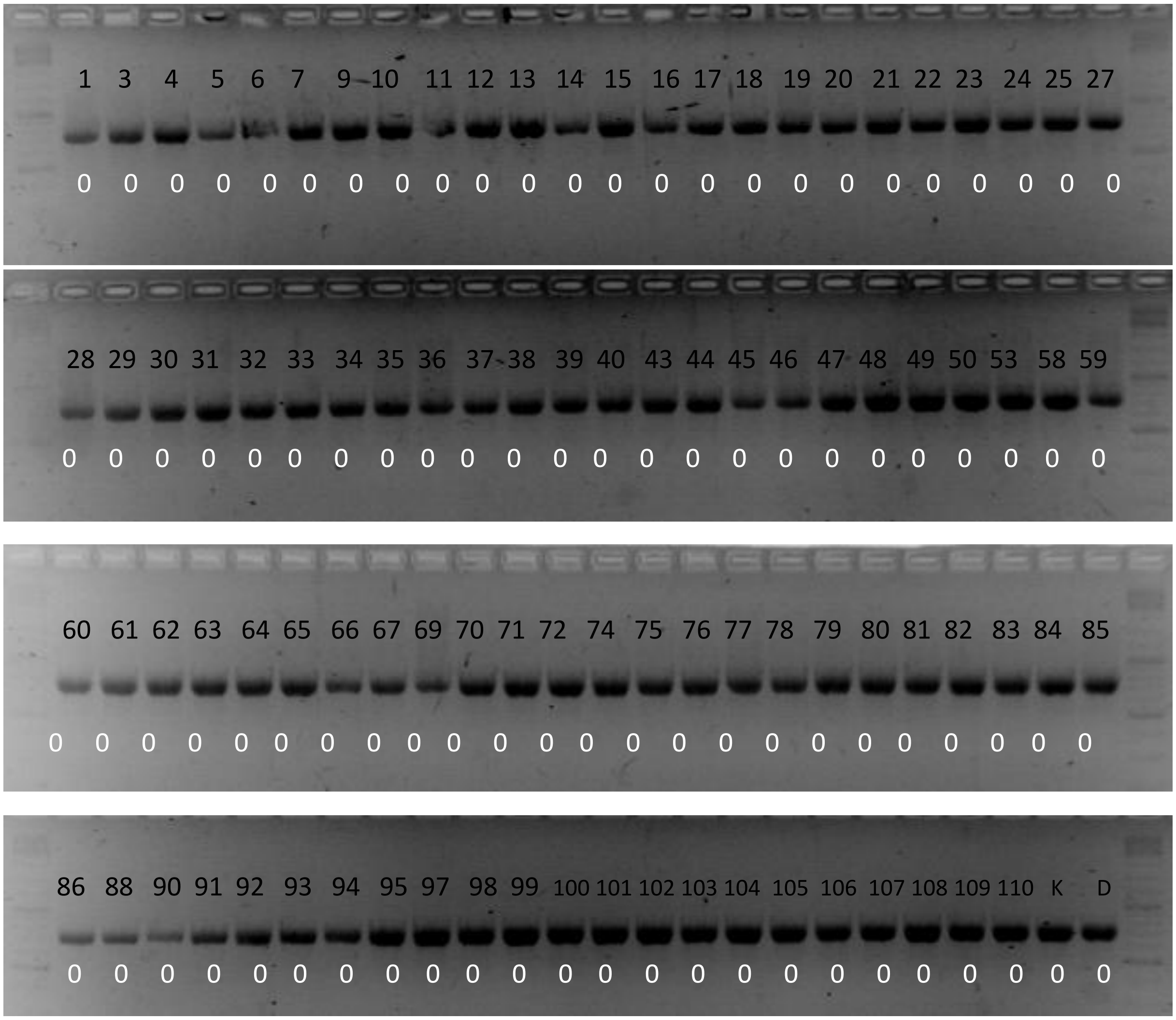

Plate 7

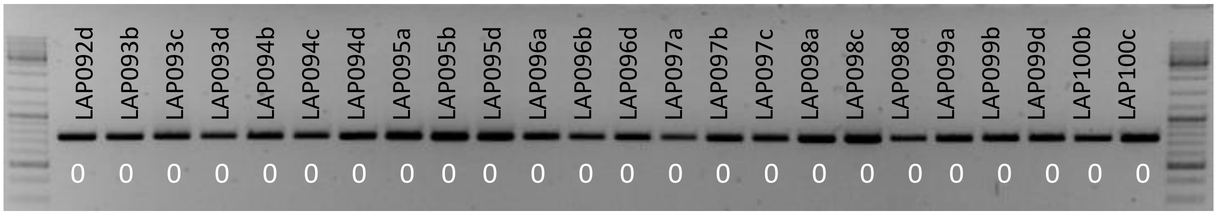
LAP092d
LAP093b
LAP093c
LAP093d
LAP094b
LAP094c
LAP094d
LAP095a
LAP095b
LAP095d
LAP096a
LAP096b
LAP096d
LAP097a
LAP097b
LAP097c
LAP098a
LAP098c
LAP098d
LAP099a
LAP099b
LAP099d
LAP100b
LAP100c
0 0 0 0 0 0 0 0 0 0 0 0 0 0 0 0 0 0 0 0 0 0 0 0

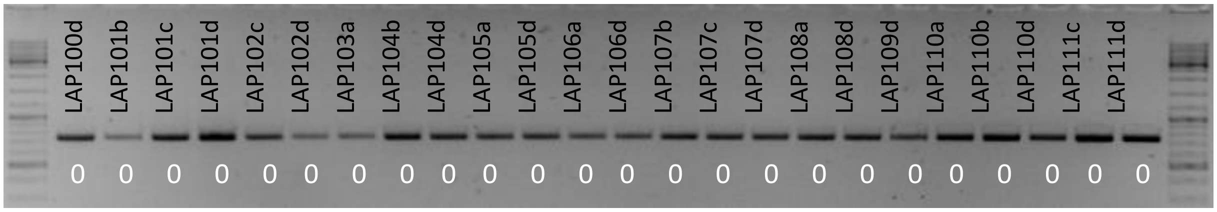
LAP100d
LAP101b
LAP101c
LAP101d
LAP102c
LAP102d
LAP103a
LAP104b
LAP104d
LAP105a
LAP105d
LAP106a
LAP106d
LAP107b
LAP107c
LAP107d
LAP108a
LAP108d
LAP109d
LAP110a
LAP110b
LAP110d
LAP111c
LAP111d
0 0 0 0 0 0 0 0 0 0 0 0 0 0 0 0 0 0 0 0 0 0 0 0

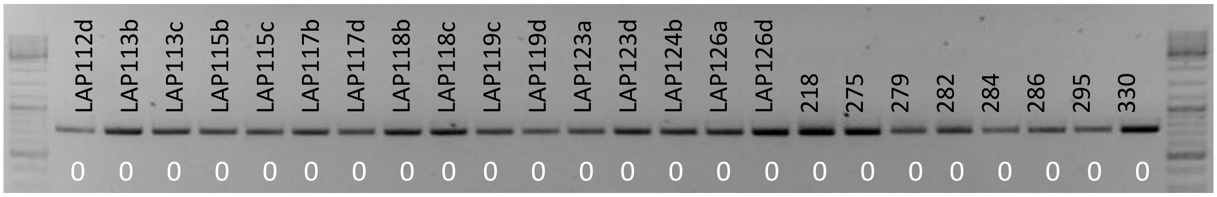
LAP112d
LAP113b
LAP113c
LAP115b
LAP115c
LAP117b
LAP117d
LAP118b
LAP118c
LAP119c
LAP119d
LAP123a
LAP123d
LAP124b
LAP126a
LAP126d
218
275
279
282
284
286
295
330
0 0 0 0 0 0 0 0 0 0 0 0 0 0 0 0 0 0 0 0 0 0 0 0

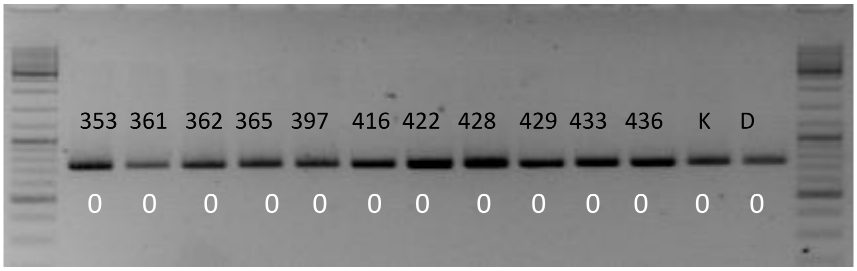
353 361 362 365 397 416 422 428 429 433 436 K D
0 0 0 0 0 0 0 0 0 0 0 0 0

QTL11_FTa1-F2

FTa1-R2 AGCAACTACTGGGCCTAGT

FTa1-F2 ACCTTCTTCCACCAGAACCAG

Plate 1

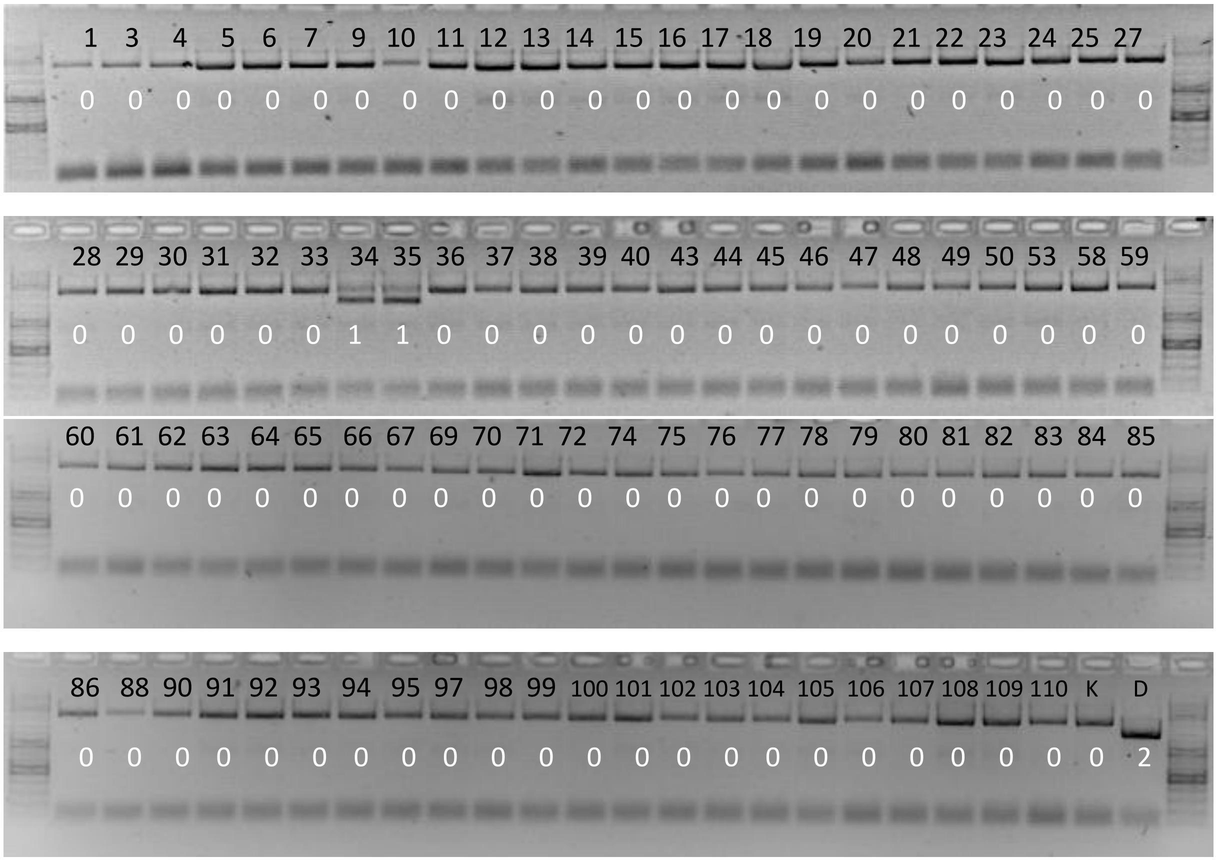

Plate 7

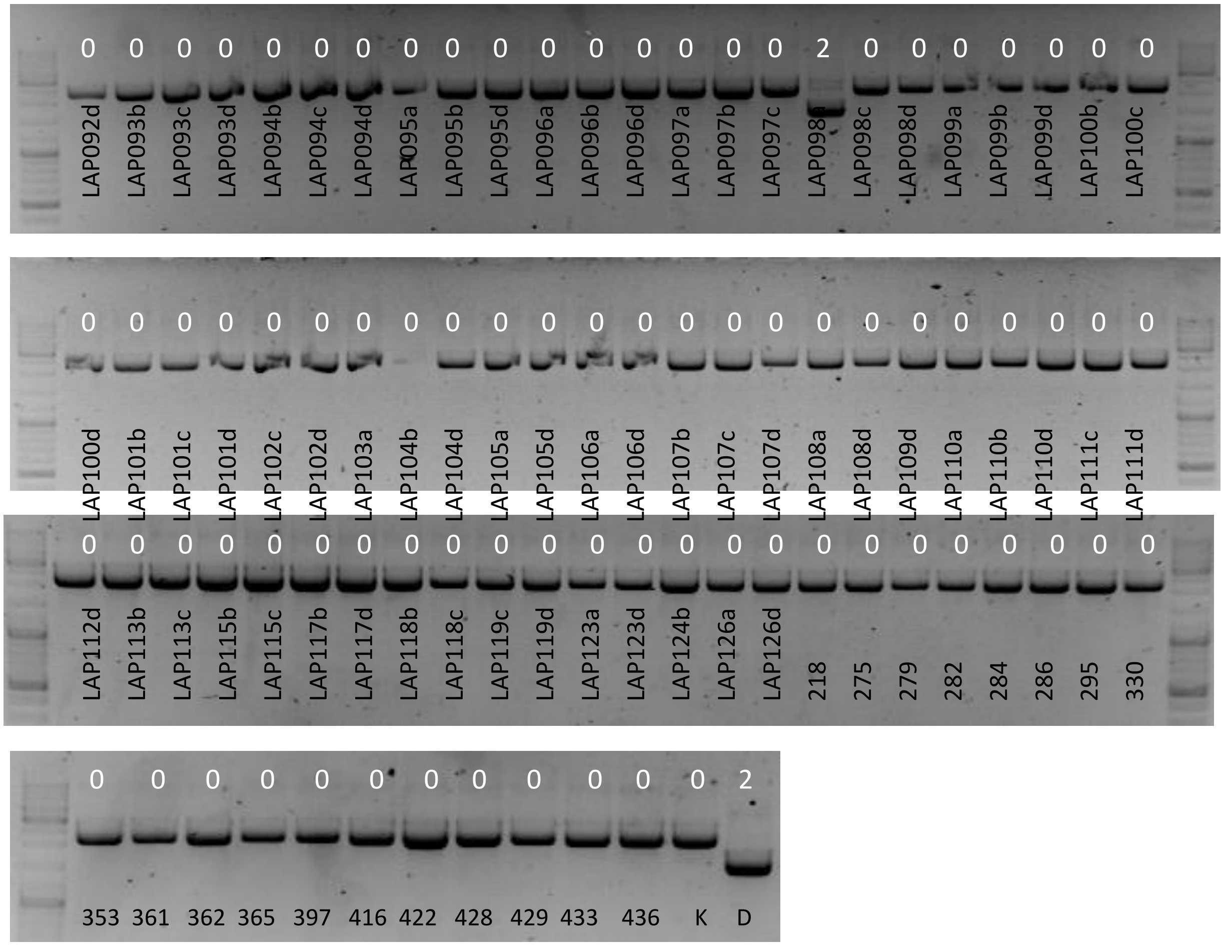
0 0 0 0 0 0 0 0 0 0 0 0 0 0 0 0 2 0 0 0 0 0 0 0
LAP092d LAP093b LAP093c LAP093d LAP094b LAP094c LAP094d LAP095a LAP095b LAP095d LAP096a LAP096b LAP096d LAP097a LAP097b LAP097c LAP098 LAP098c LAP098d LAP099a LAP099b LAP099d LAP100b LAP100c
0 0 0 0 0 0 0 0 0 0 0 0 0 0 0 0 0 0 0 0 0 0 0 0
LAP100d LAP101b LAP101c LAP101d LAP102c LAP102d LAP103a LAP104b LAP104d LAP105a LAP105d LAP106a LAP106d LAP107b LAP107c LAP107d LAP108a LAP108d LAP109d LAP110a LAP110b LAP110d LAP111c LAP111d
0 0 0 0 0 0 0 0 0 0 0 0 0 0 0 0 0 0 0 0 0 0 0 0
LAP112d LAP113b LAP113c LAP115b LAP115c LAP117b LAP117d LAP118b LAP118c LAP119c LAP119d LAP123a LAP123d LAP124b LAP126a LAP126d 218 275 279 282 284 286 295 330
0 0 0 0 0 0 0 0 0 0 0 0 2
353 361 362 365 397 416 422 428 429 433 436 K D
