# Supplementary material for: Evaluation of the Global White Lupin Collection Reveals Significant Associations Between Homologous FLOWERING LOCUS T Indels and Flowering Time, Providing Validated Markers for Tracking Spring Ecotypes Within a Large Gene Pool
Source: Int J Mol Sci. 2025 Jul 17;26(14):6858. doi: 10.3390/ijms26146858 (PMC12295241; doi:10.3390/ijms26146858)
Supplement: Supplementary file 1 [file ijms-26-06858-s001.zip › Supplementary_Figure_S4_Agarose gel electrophoregrams showing polymorphism of PCR-based markers targeting LalbFTa2 indels.pdf]

Wojciech Bielski, Anna Surma, Michał Książkiewicz, Sandra Rychel-Bielska

Evaluation of the global white lupin collection reveals significant associations between homologous *FLOWERING LOCUS T* indels and flowering time, providing validated markers for tracking spring ecotypes within a large gene pool

International Journal of Molecular Sciences

Supplementary Figure S4. Agarose gel electrophoregrams showing polymorphism of PCR-based markers targeting *LalbFTa2* indels.

PR_16

PRFTA2F1 TCCGTGGGAAATGATGATATTACA

PRFTa2_R1b GGAGTATTATTGGAGTGACTGAGCA

Plate 1

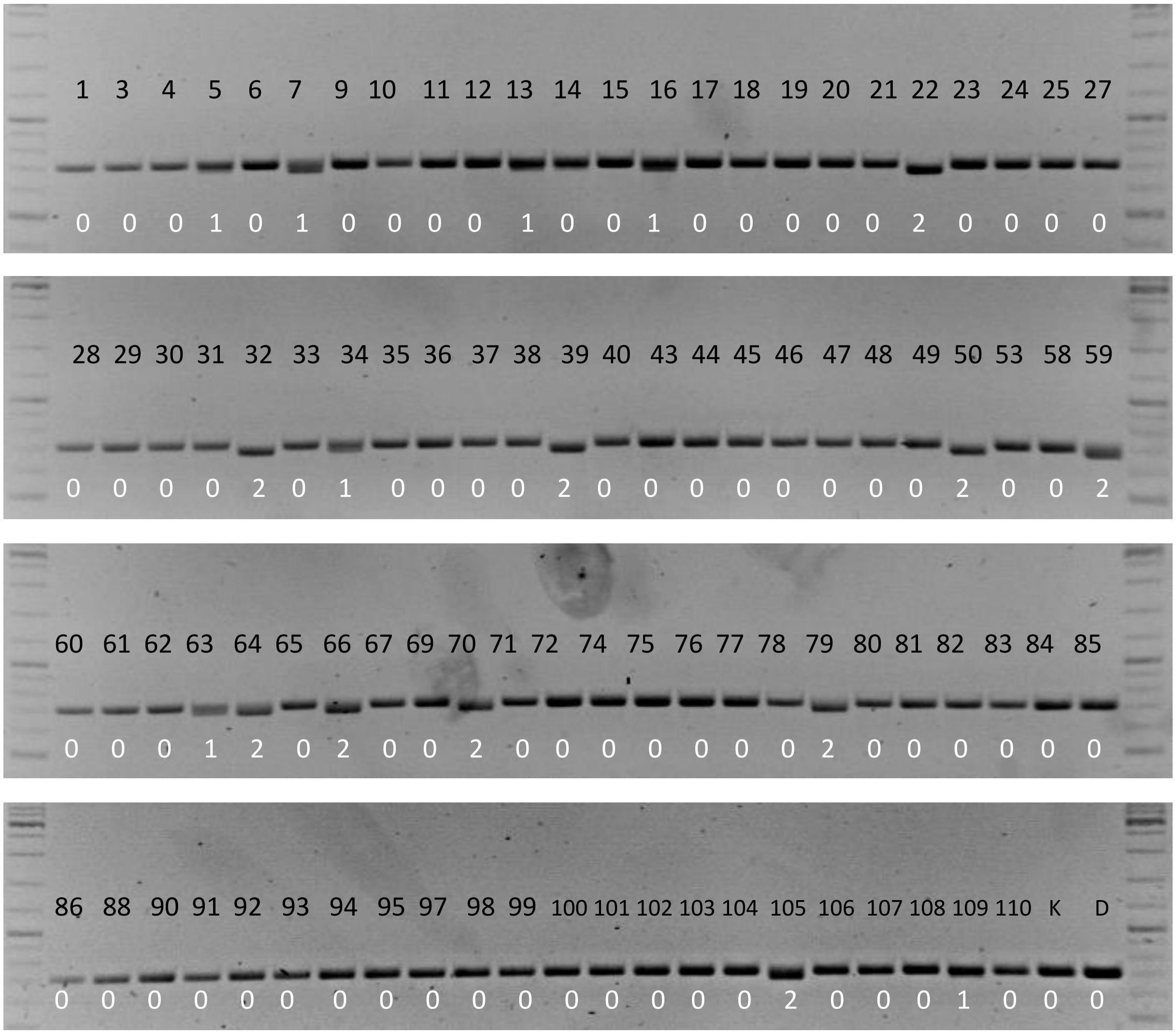

Plate 7

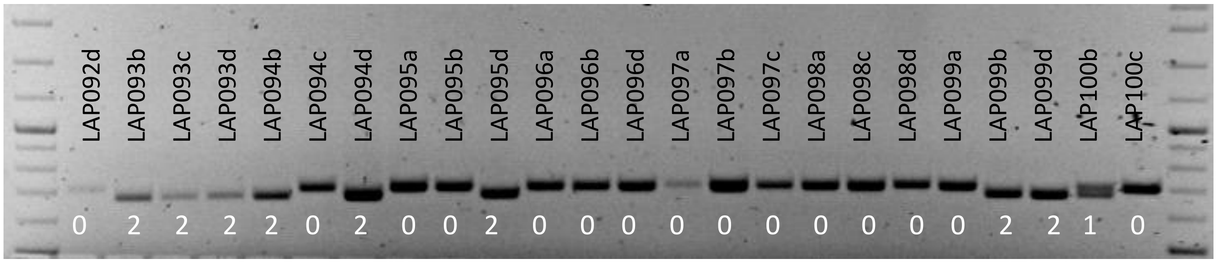
LAP092d LAP093b LAP093c LAP093d LAP094b LAP094c LAP094d LAP095a LAP095b LAP095d LAP096a LAP096b LAP096d LAP097a LAP097b LAP097c LAP098a LAP098c LAP098d LAP099a LAP099b LAP099d LAP100b LAP100c
0 2 2 2 2 0 2 0 0 2 0 0 0 0 0 0 0 0 0 0 2 2 1 0

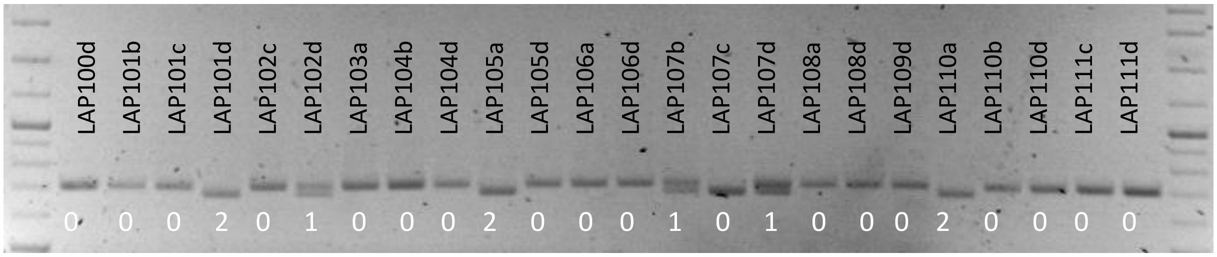
LAP100d LAP101b LAP101c LAP101d LAP102c LAP102d LAP103a LAP104b LAP104d LAP105a LAP105d LAP106a LAP106d LAP107b LAP107c LAP107d LAP108a LAP108d LAP109d LAP110a LAP110b LAP110d LAP111c LAP111d
0 0 0 2 0 1 0 0 0 2 0 0 0 1 0 1 0 0 0 2 0 0 0 0

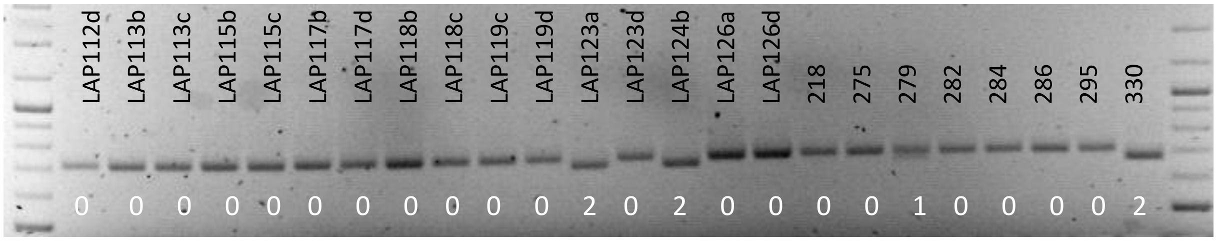
LAP112d LAP113b LAP113c LAP115b LAP115c LAP117b LAP117d LAP118b LAP118c LAP119c LAP119d LAP123a LAP123d LAP124b LAP126a LAP126d 218 275 279 282 284 286 295 330
0 0 0 0 0 0 0 0 0 0 0 2 0 2 0 0 0 0 1 0 0 0 0 2

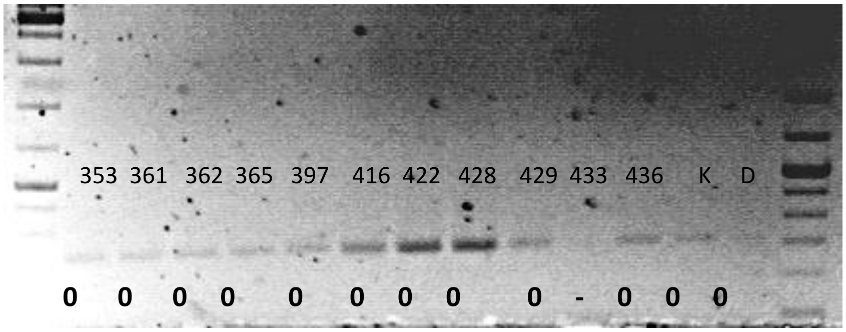
353 361 362 365 397 416 422 428 429 433 436 K D
0 0 0 0 0 0 0 0 0 - 0 0 0

PR_17

PRFTa2_F1b TGAATGTCAGACCTGCCATAAACTA

PRFTa2_R1c TGTAATTAATGGAAGGATCTTGGTTT

Plate 1

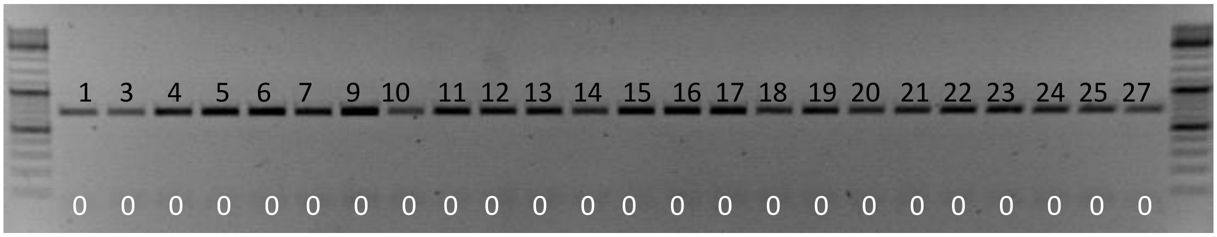

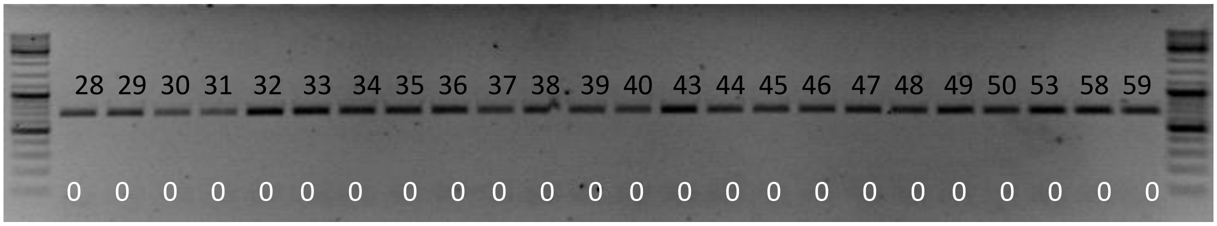

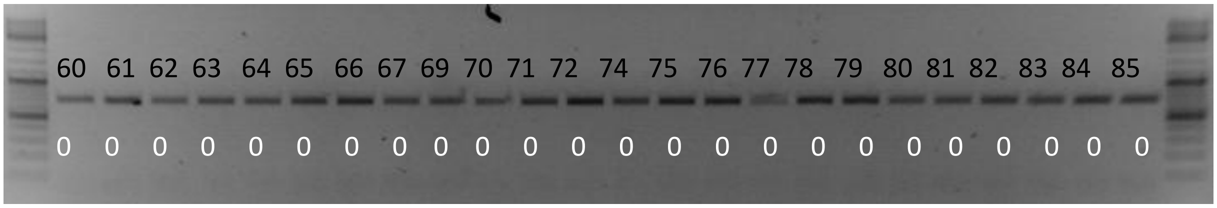

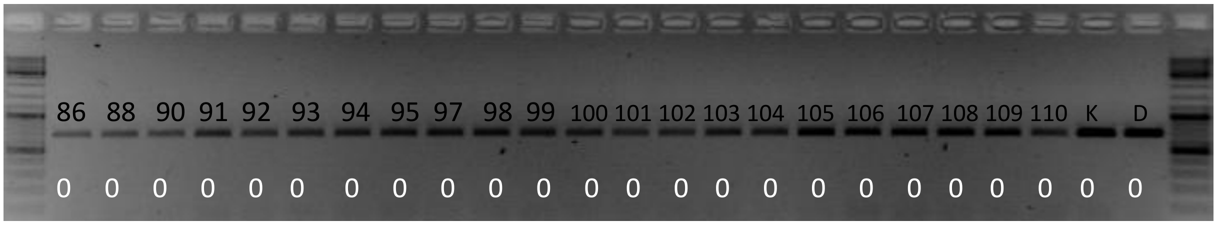

Plate 9

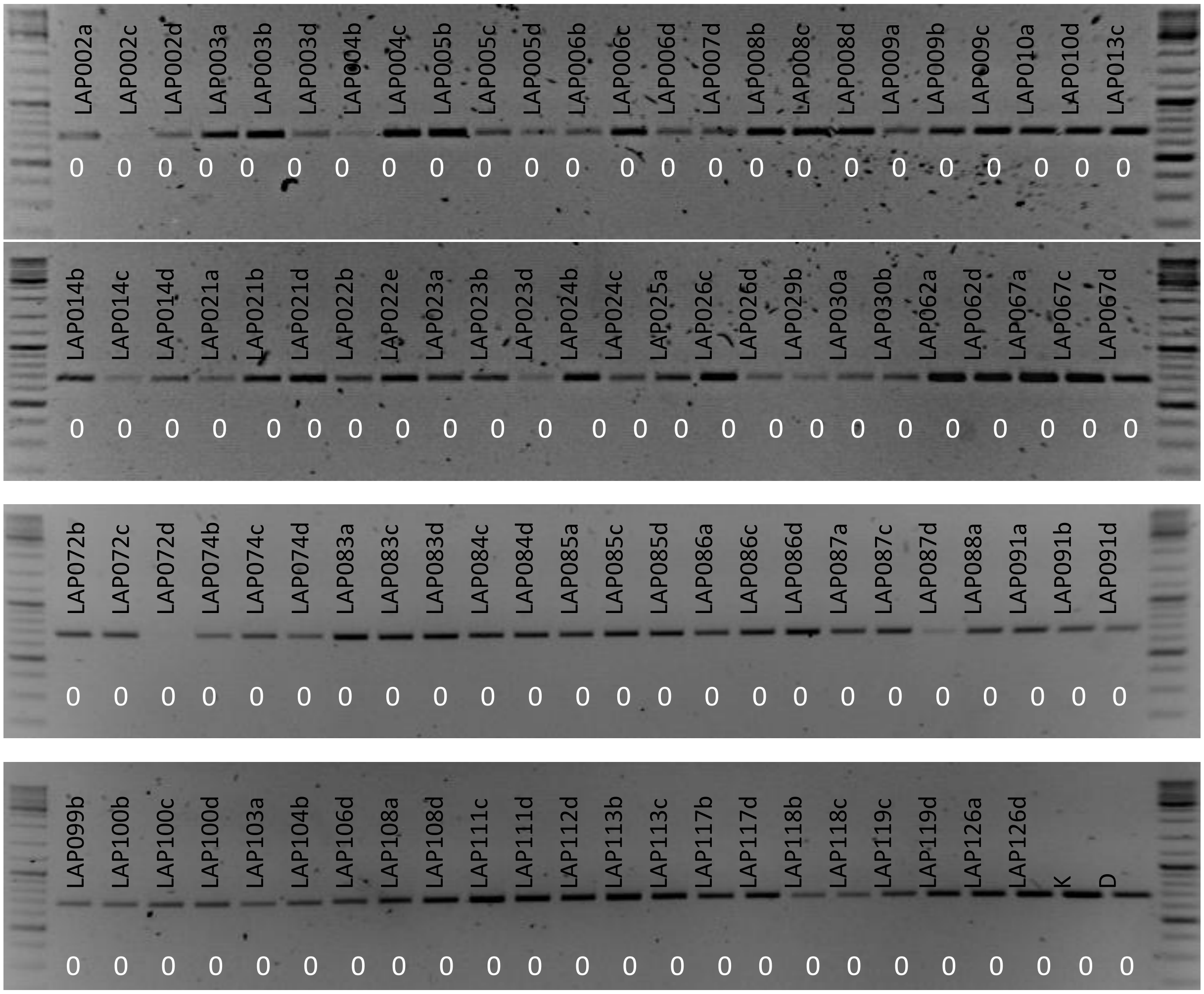
LAP002a LAP002c LAP002d LAP003a LAP003b LAP003d LAP004b LAP004c LAP005b LAP005c LAP005d LAP006b LAP006c LAP006d LAP007d LAP008b LAP008c LAP008d LAP009a LAP009b LAP009c LAP010a LAP010d LAP013c
0 0 0 0 0 0 0 0 0 0 0 0 0 0 0 0 0 0 0 0 0 0 0 0
LAP014b LAP014c LAP014d LAP021a LAP021b LAP021d LAP022b LAP022e LAP023a LAP023b LAP023d LAP024b LAP024c LAP025a LAP026c LAP026d LAP029b LAP030a LAP030b LAP062a LAP062d LAP067a LAP067c LAP067d
0 0 0 0 0 0 0 0 0 0 0 0 0 0 0 0 0 0 0 0 0 0 0 0
LAP072b LAP072c LAP072d LAP074b LAP074c LAP074d LAP083a LAP083c LAP083d LAP084c LAP084d LAP085a LAP085c LAP085d LAP086a LAP086c LAP086d LAP087a LAP087c LAP087d LAP088a LAP091a LAP091b LAP091d
0 0 0 0 0 0 0 0 0 0 0 0 0 0 0 0 0 0 0 0 0 0 0 0
LAP099b LAP100b LAP100c LAP100d LAP103a LAP104b LAP106d LAP108a LAP108d LAP111c LAP111d LAP112d LAP113b LAP113c LAP117b LAP117d LAP118b LAP118c LAP119c LAP119d LAP126a LAP126d K D
0 0 0 0 0 0 0 0 0 0 0 0 0 0 0 0 0 0 0 0 0 0 0 0

PR_18a

PRFTa2_F1c TCTTCTTGACACTTGTACATGTCCT

PRFTA2R1 ATAATCACAAGGCACACAAGC

Plate 1

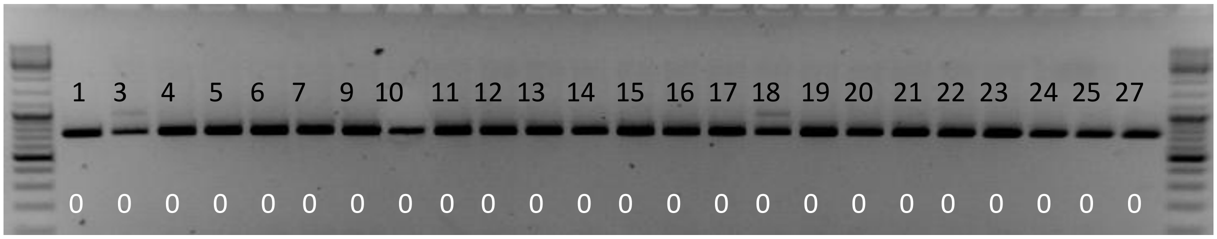

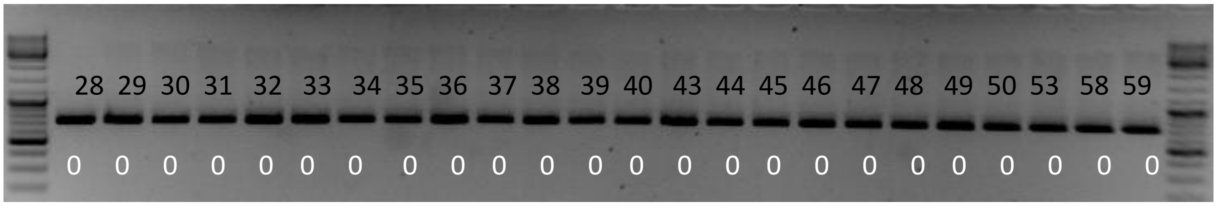

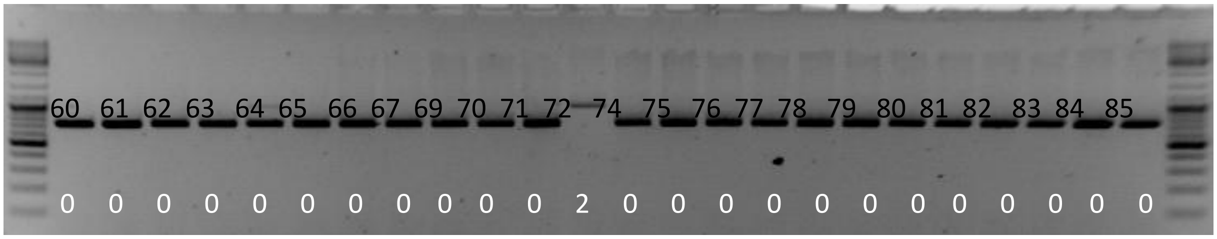

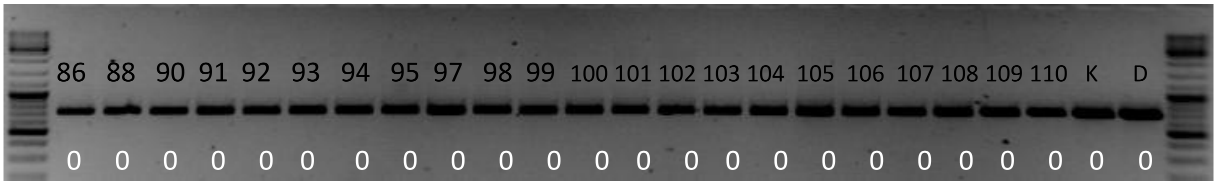

Plate 7

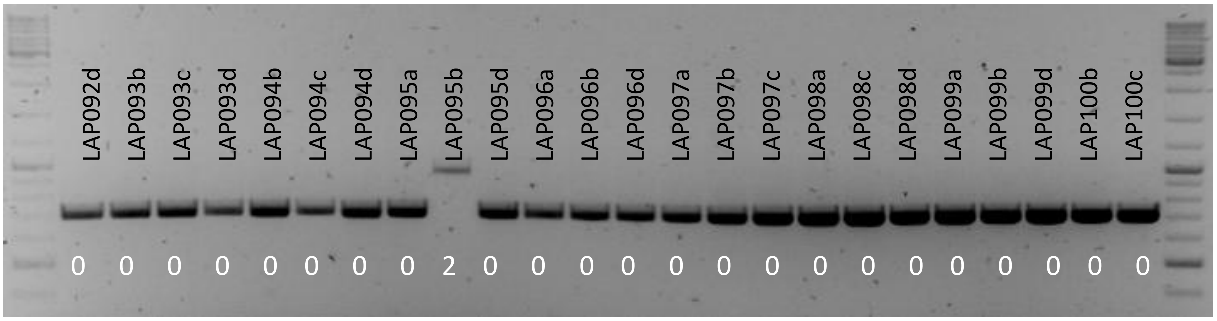
LAP092d LAP093b LAP093c LAP093d LAP094b LAP094c LAP094d LAP095a LAP095b LAP095d LAP096a LAP096b LAP096d LAP097a LAP097b LAP097c LAP098a LAP098c LAP098d LAP099a LAP099b LAP099d LAP100b LAP100c
0 0 0 0 0 0 0 0 2 0 0 0 0 0 0 0 0 0 0 0 0 0 0 0

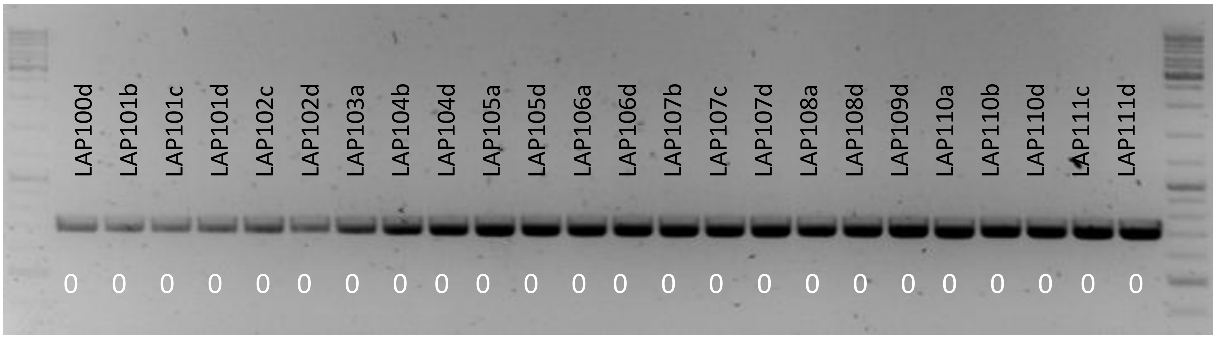
LAP100d LAP101b LAP101c LAP101d LAP102c LAP102d LAP103a LAP104b LAP104d LAP105a LAP105d LAP106a LAP106d LAP107b LAP107c LAP107d LAP108a LAP108d LAP109d LAP110a LAP110b LAP110d LAP111c LAP111d
0 0 0 0 0 0 0 0 0 0 0 0 0 0 0 0 0 0 0 0 0 0 0 0

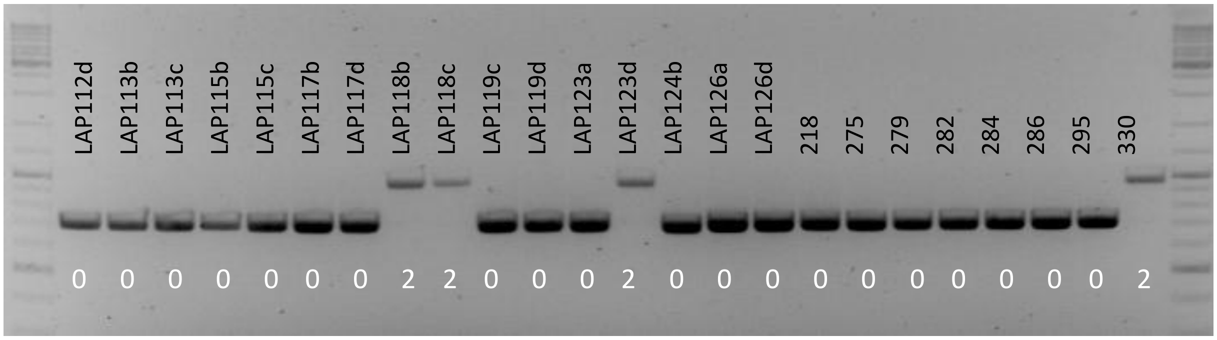
LAP112d LAP113b LAP113c LAP115b LAP115c LAP117b LAP117d LAP118b LAP118c LAP119c LAP119d LAP123a LAP123d LAP124b LAP126a LAP126d 218 275 279 282 284 286 295 330
0 0 0 0 0 0 0 2 2 0 0 0 2 0 0 0 0 0 0 0 0 0 0 2

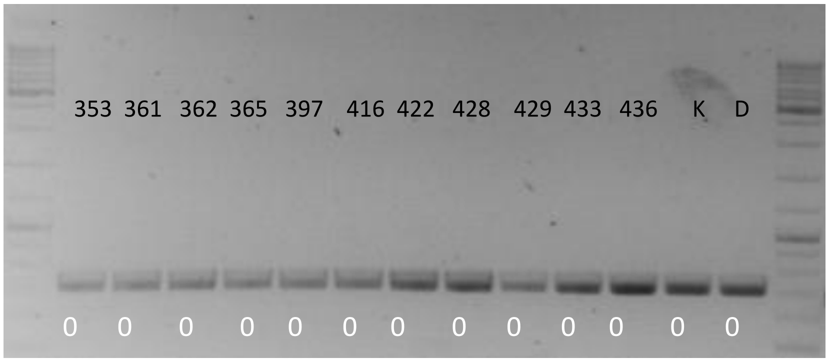
353 361 362 365 397 416 422 428 429 433 436 K D
0 0 0 0 0 0 0 0 0 0 0 0 0

PR_18b

PRFTa2_F1c TCTTCTTGACACTTGTACATGTCCT

PRFTA2R1 ATAATCACAAGGCACACAAGC

Plate 1

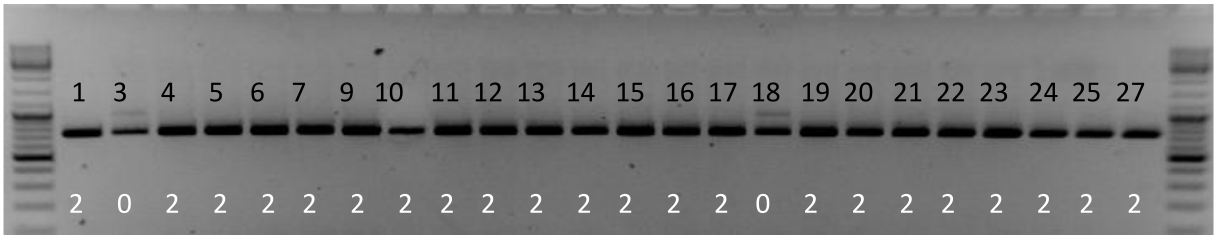

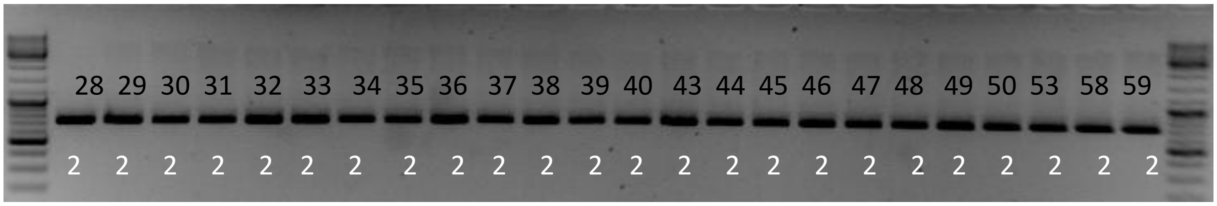

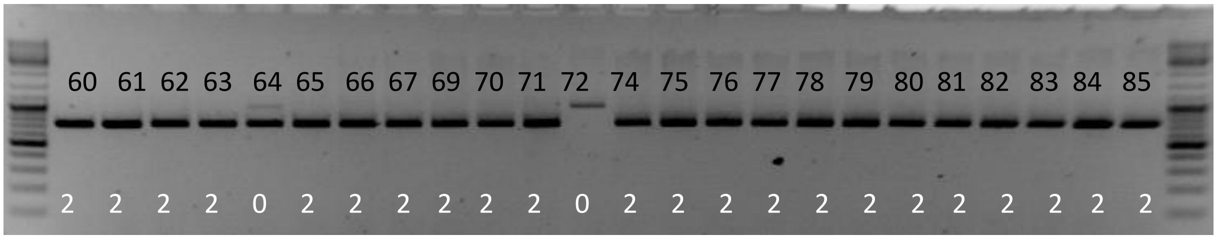

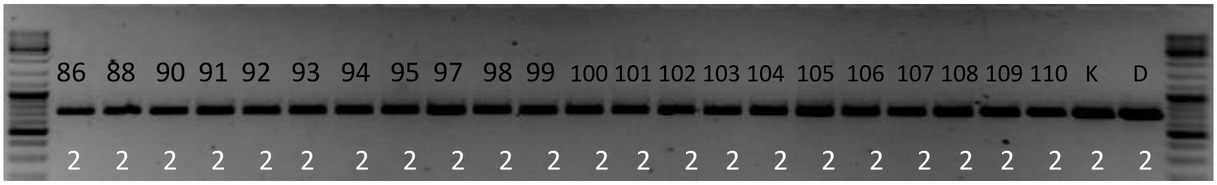

Plate 7

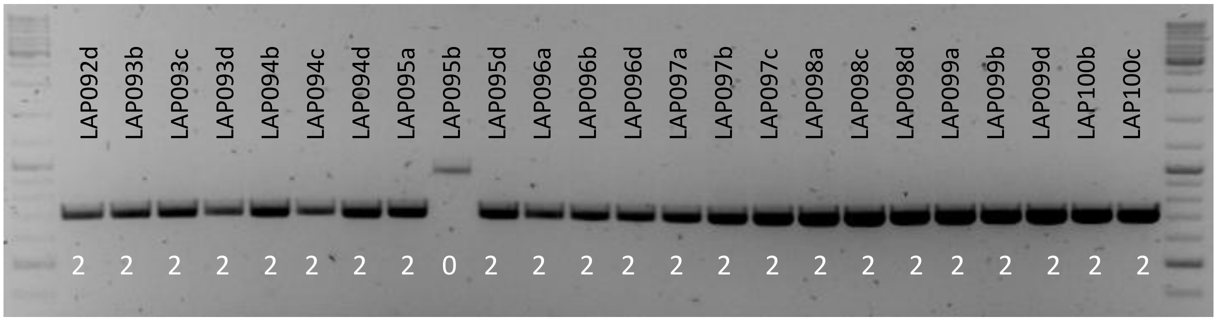
LAP092d LAP093b LAP093c LAP093d LAP094b LAP094c LAP094d LAP095a LAP095b LAP095d LAP096a LAP096b LAP096d LAP097a LAP097b LAP097c LAP098a LAP098c LAP098d LAP099a LAP099b LAP099d LAP100b LAP100c
2 2 2 2 2 2 2 2 0 2 2 2 2 2 2 2 2 2 2 2 2 2 2 2

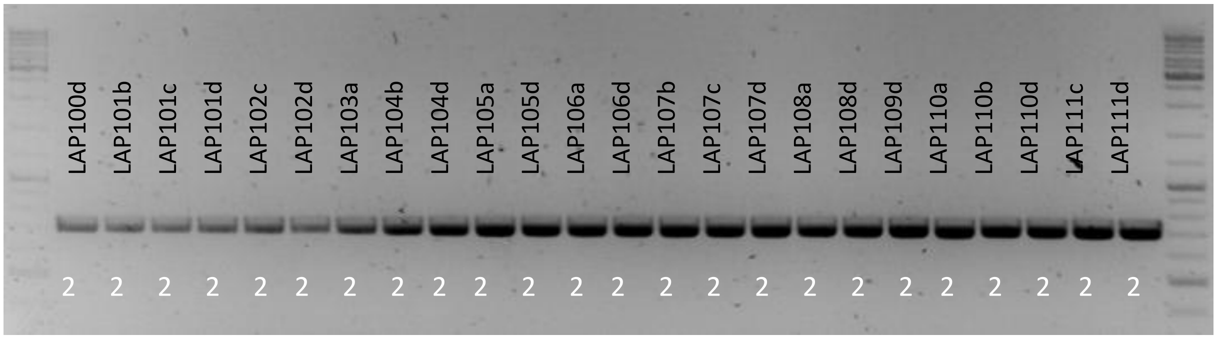
LAP100d LAP101b LAP101c LAP101d LAP102c LAP102d LAP103a LAP104b LAP104d LAP105a LAP105d LAP106a LAP106d LAP107b LAP107c LAP107d LAP108a LAP108d LAP109d LAP110a LAP110b LAP110d LAP111c LAP111d
2 2 2 2 2 2 2 2 2 2 2 2 2 2 2 2 2 2 2 2 2 2 2 2

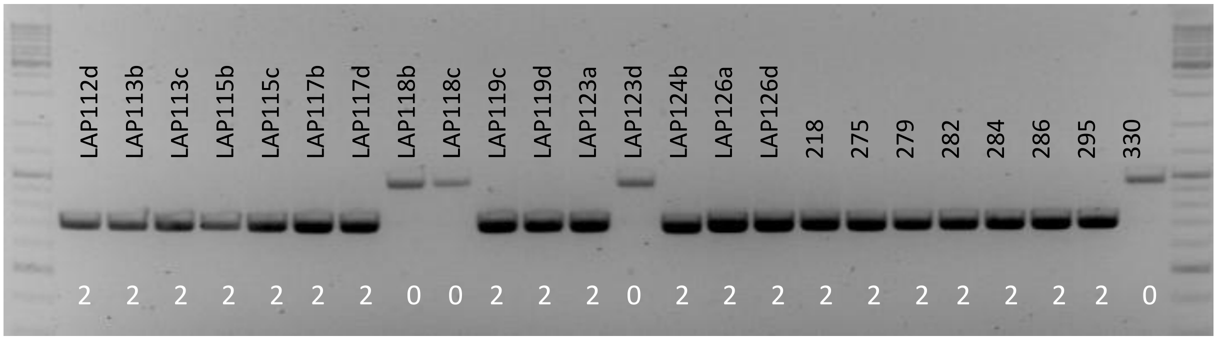
LAP112d LAP113b LAP113c LAP115b LAP115c LAP117b LAP117d LAP118b LAP118c LAP119c LAP119d LAP123a LAP123d LAP124b LAP126a LAP126d 218 275 279 282 284 286 295 330
2 2 2 2 2 2 2 0 0 2 2 2 0 2 2 2 2 2 2 2 2 2 2 0

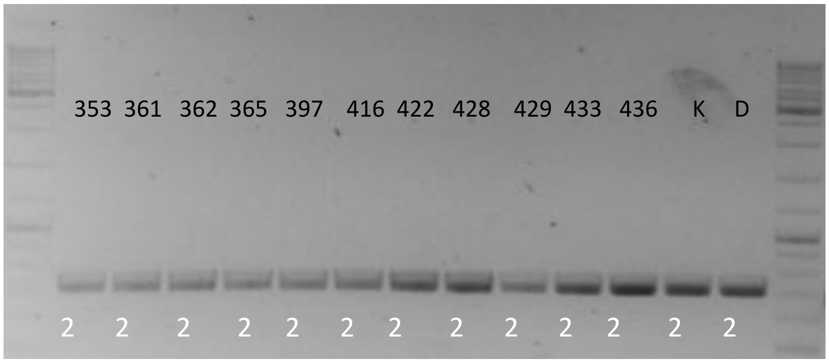
353 361 362 365 397 416 422 428 429 433 436 K D
2 2 2 2 2 2 2 2 2 2 2 2 2

PR_19a

PRFTA2F2 TTGTTTATGATTTCCTTAAGAGATGGA

PRFTa2_R2b AATTTATTAATTGGGACGGGTTCGG

Plate 1

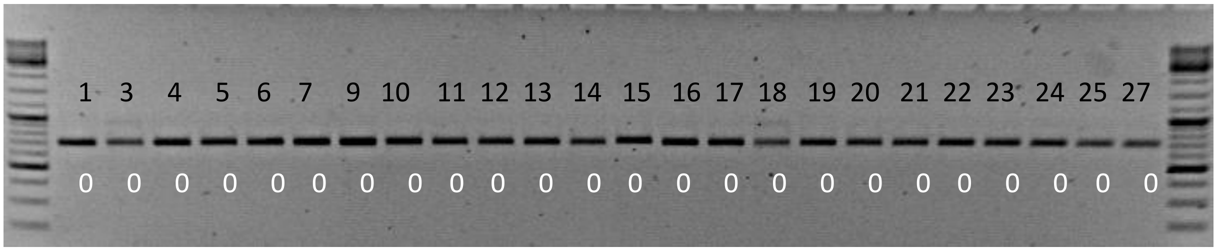

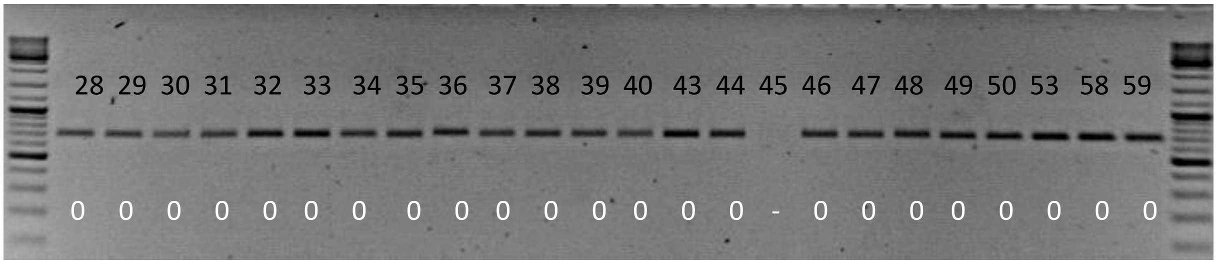

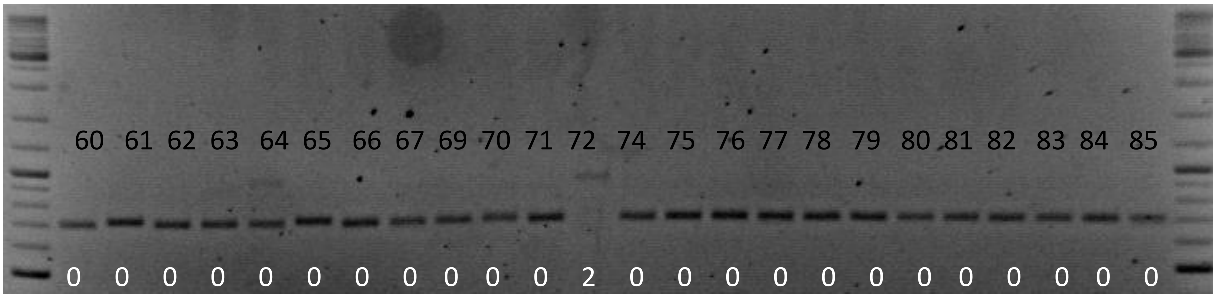

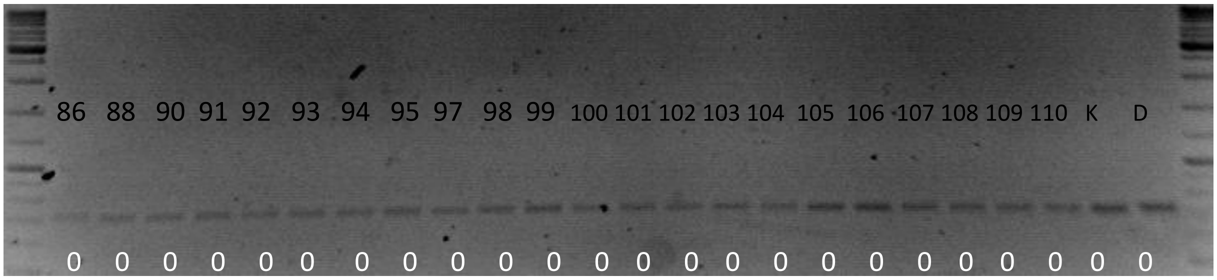

Plate 7

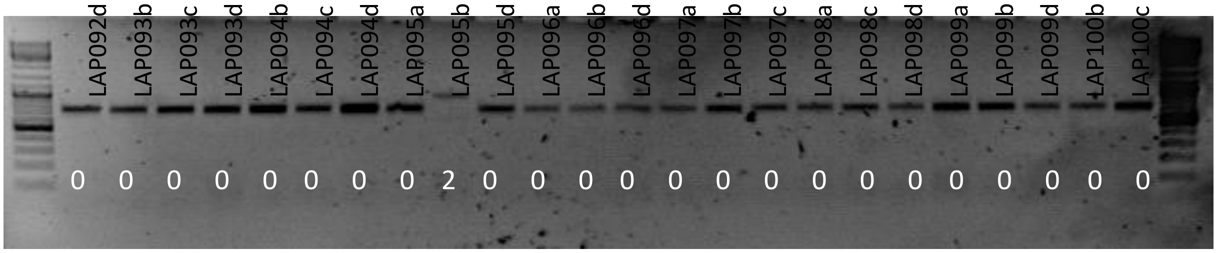
LAP092d LAP093b LAP093c LAP093d LAP094b LAP094c LAP094d LAP095a LAP095b LAP095d LAP096a LAP096b LAP096d LAP097a LAP097b LAP097c LAP098a LAP098c LAP098d LAP099a LAP099b LAP099d LAP100b LAP100c
0 0 0 0 0 0 0 0 2 0 0 0 0 0 0 0 0 0 0 0 0 0 0 0

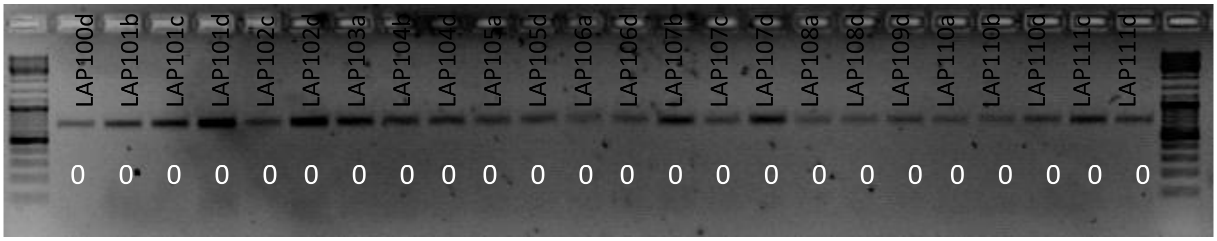
LAP100d LAP101b LAP101c LAP101d LAP102c LAP102d LAP103a LAP104b LAP104d LAP105a LAP105d LAP106a LAP106d LAP107b LAP107c LAP107d LAP108a LAP108d LAP109d LAP110a LAP110b LAP110d LAP111c LAP111d
0 0 0 0 0 0 0 0 0 0 0 0 0 0 0 0 0 0 0 0 0 0 0 0

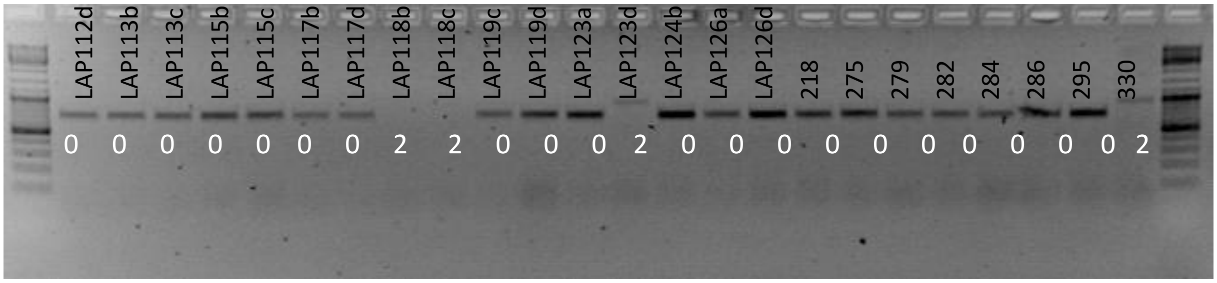
LAP112d LAP113b LAP113c LAP115b LAP115c LAP117b LAP117d LAP118b LAP118c LAP119c LAP119d LAP123a LAP123d LAP124b LAP126a LAP126d 218 275 279 282 284 286 295 330
0 0 0 0 0 0 0 2 2 0 0 0 2 0 0 0 0 0 0 0 0 0 0 2

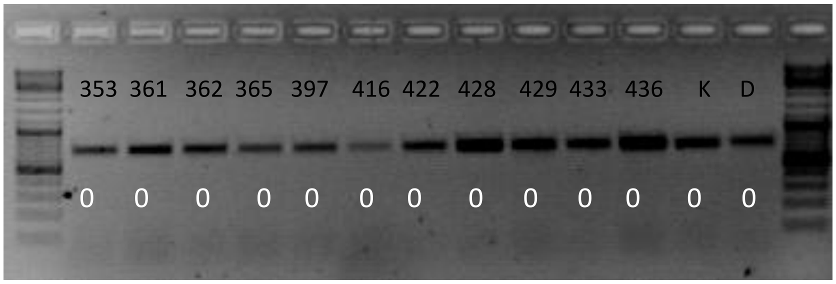
353 361 362 365 397 416 422 428 429 433 436 K D
0 0 0 0 0 0 0 0 0 0 0 0 0

PR_19b

PRFTA2F2 TTGTTTATGATTTCCTTAAGAGATGGA

PRFTa2_R2b AATTTATTAATTGGGACGGGTTCGG

Plate 1

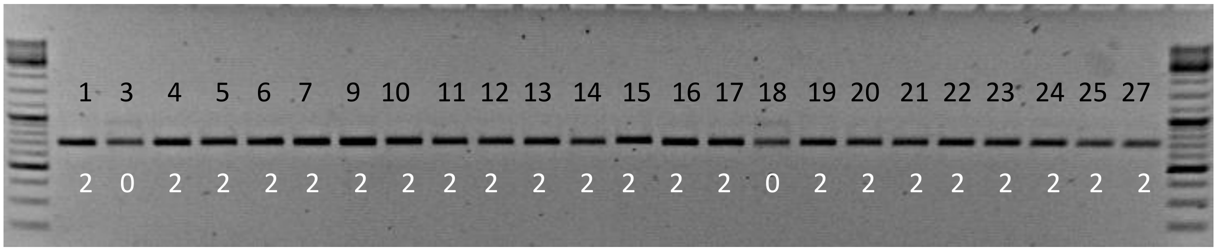

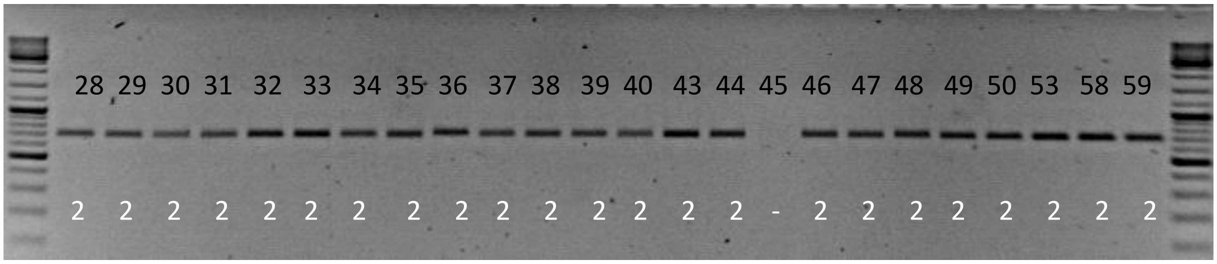

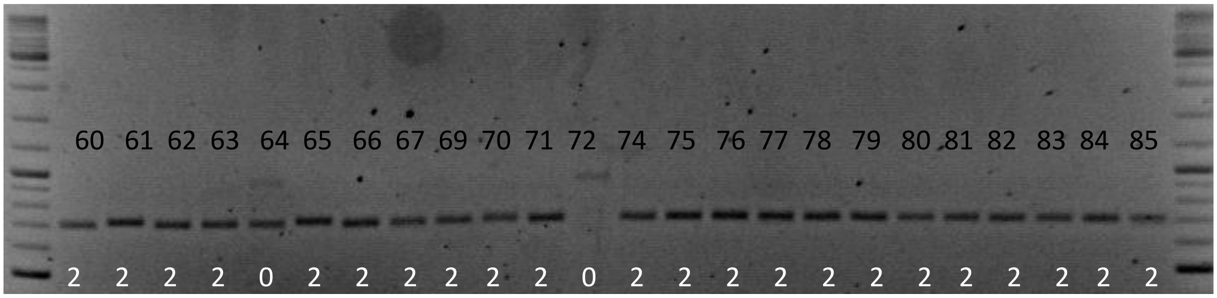

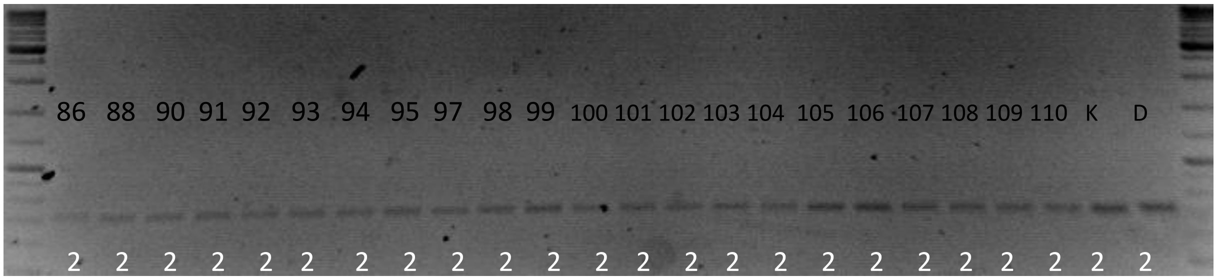

Plate 7

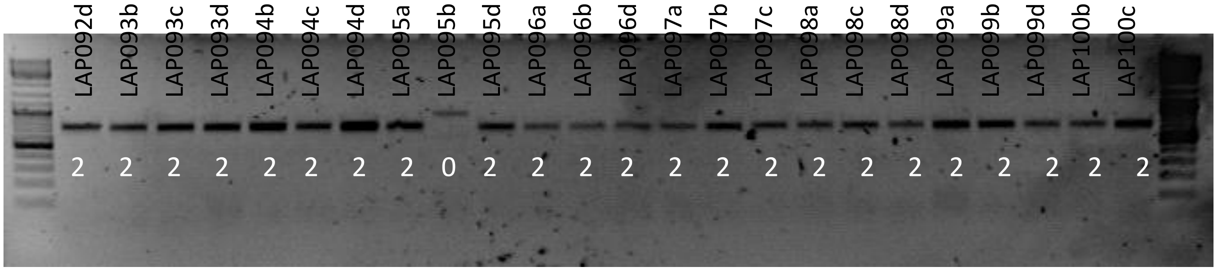
LAP092d LAP093b LAP093c LAP093d LAP094b LAP094c LAP094d LAP095a LAP095b LAP095d LAP096a LAP096b LAP096d LAP097a LAP097b LAP097c LAP098a LAP098c LAP098d LAP099a LAP099b LAP099d LAP100b LAP100c
2 2 2 2 2 2 2 2 0 2 2 2 2 2 2 2 2 2 2 2 2 2 2 2

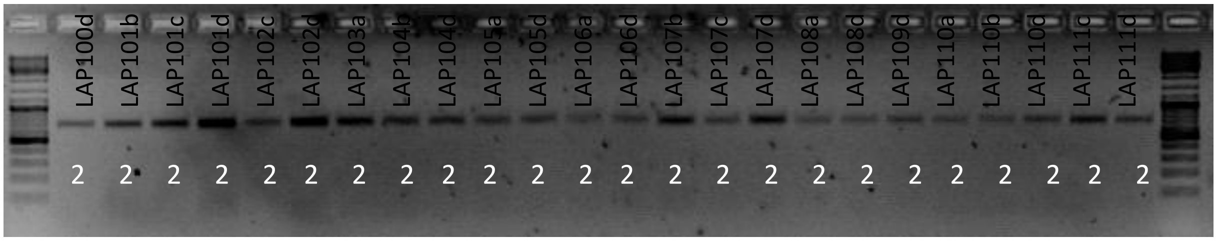
LAP100d LAP101b LAP101c LAP101d LAP102c LAP102d LAP103a LAP104b LAP104d LAP105a LAP105d LAP106a LAP106d LAP107b LAP107c LAP107d LAP108a LAP108d LAP109d LAP110a LAP110b LAP110d LAP111c LAP111d
2 2 2 2 2 2 2 2 2 2 2 2 2 2 2 2 2 2 2 2 2 2 2 2

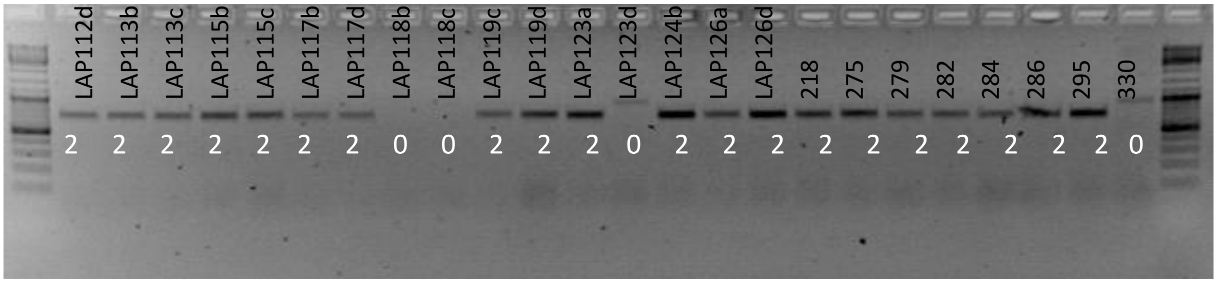
LAP112d LAP113b LAP113c LAP115b LAP115c LAP117b LAP117d LAP118b LAP118c LAP119c LAP119d LAP123a LAP123d LAP124b LAP126a LAP126d 218 275 279 282 284 286 295 330
2 2 2 2 2 2 2 0 0 2 2 2 0 2 2 2 2 2 2 2 2 2 2 0

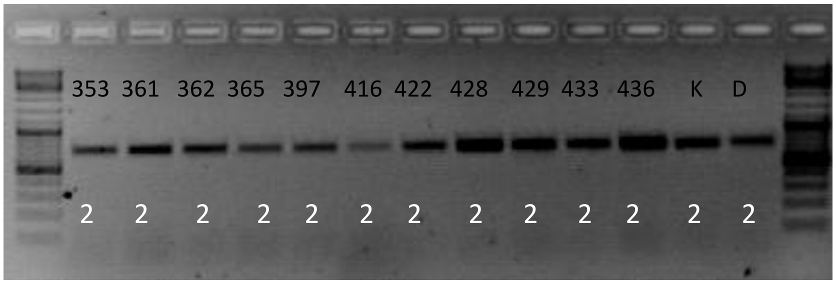
353 361 362 365 397 416 422 428 429 433 436 K D
2 2 2 2 2 2 2 2 2 2 2 2 2

PR_20

PRFTa2_F2b TTTCAGGTTCGGGTTTGCTACAT

PRFTa2_R2c AGTTATGTGCCTATTATGTCGGAAA

Plate 1

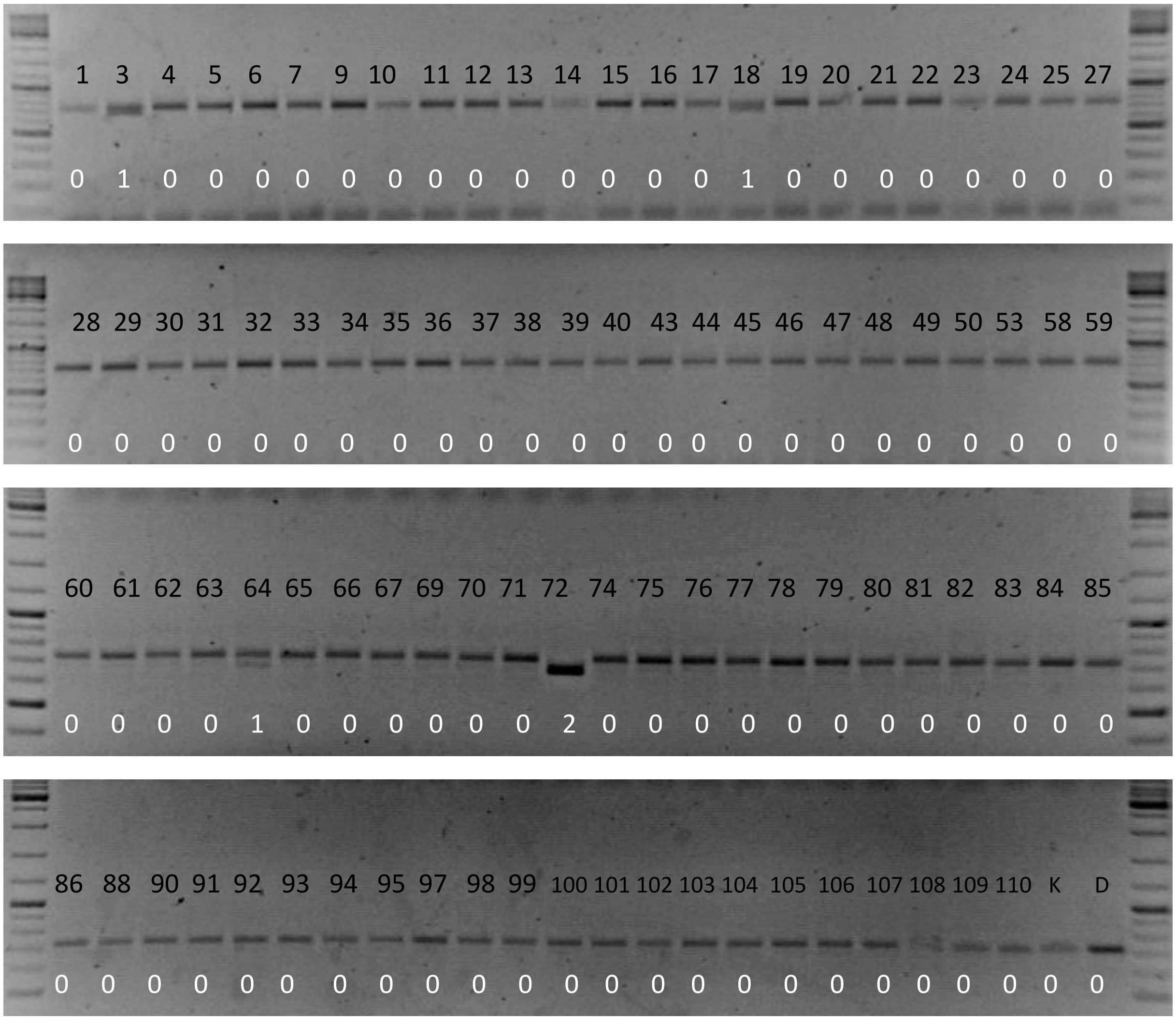

Plate 7

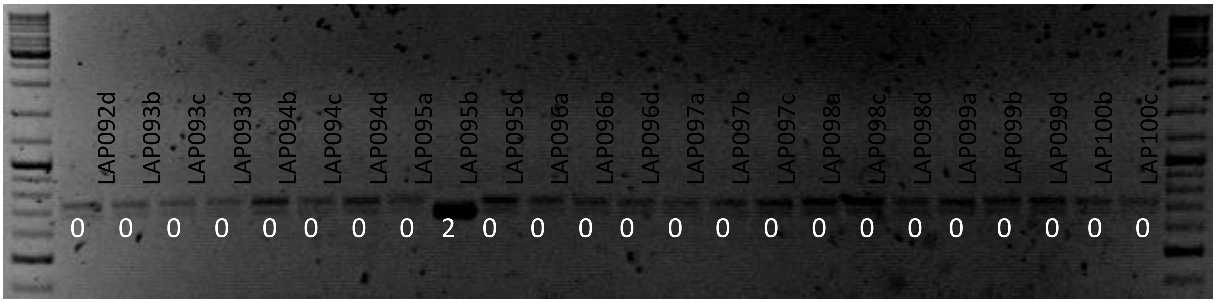
LAP092d LAP093b LAP093c LAP093d LAP094b LAP094c LAP094d LAP095a LAP095b LAP095d LAP096a LAP096b LAP096d LAP097a LAP097b LAP097c LAP098a LAP098c LAP098d LAP099a LAP099b LAP099d LAP100b LAP100c
0 0 0 0 0 0 0 0 2 0 0 0 0 0 0 0 0 0 0 0 0 0 0 0

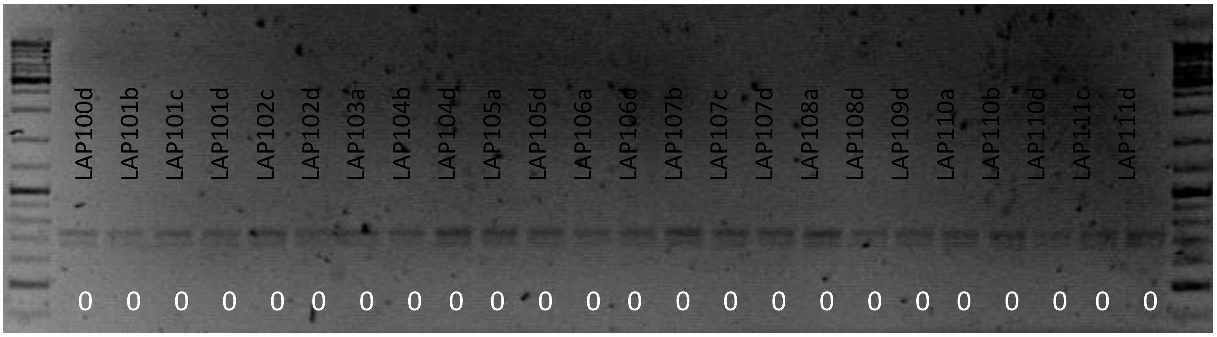
LAP100d LAP101b LAP101c LAP101d LAP102c LAP102d LAP103a LAP104b LAP104d LAP105a LAP105d LAP106a LAP106d LAP107b LAP107c LAP107d LAP108a LAP108d LAP109d LAP110a LAP110b LAP110d LAP111c LAP111d
0 0 0 0 0 0 0 0 0 0 0 0 0 0 0 0 0 0 0 0 0 0 0 0

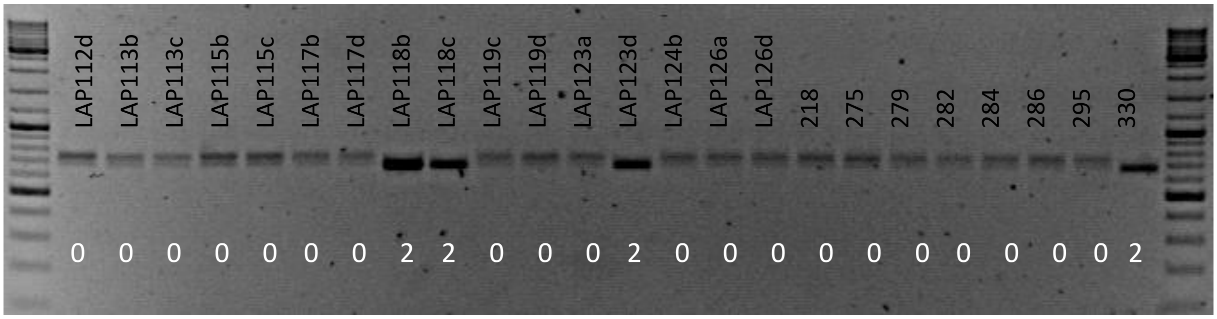
LAP112d LAP113b LAP113c LAP115b LAP115c LAP117b LAP117d LAP118b LAP118c LAP119c LAP119d LAP123a LAP123d LAP124b LAP126a LAP126d 218 275 279 282 284 286 295 330
0 0 0 0 0 0 0 2 2 0 0 0 2 0 0 0 0 0 0 0 0 0 0 2

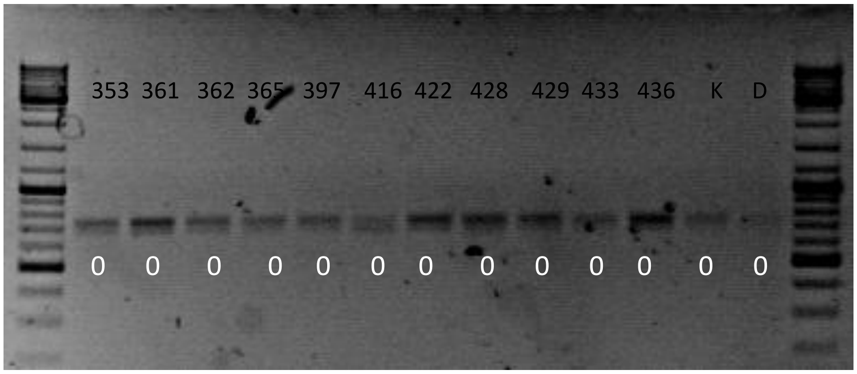
353 361 362 365 397 416 422 428 429 433 436 K D
0 0 0 0 0 0 0 0 0 0 0 0 0

PR_21

PRFTa2_F2c ACTTTATAACAGCTAGGTTTGAATGCA

PRFTA2R2 AACAAAAATAATGAAGAGATGATGACT

Plate 1

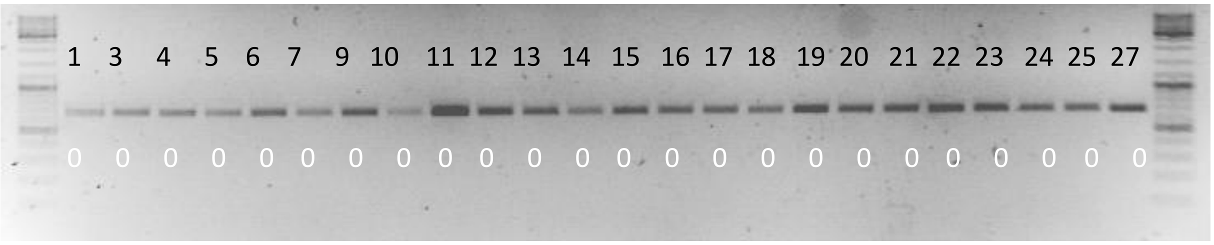

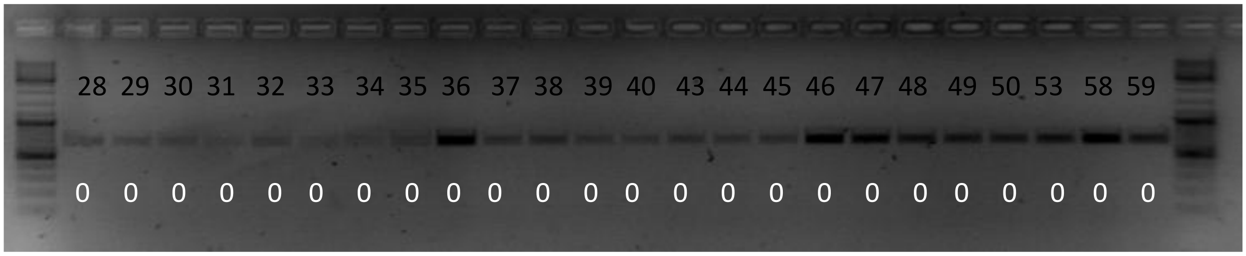

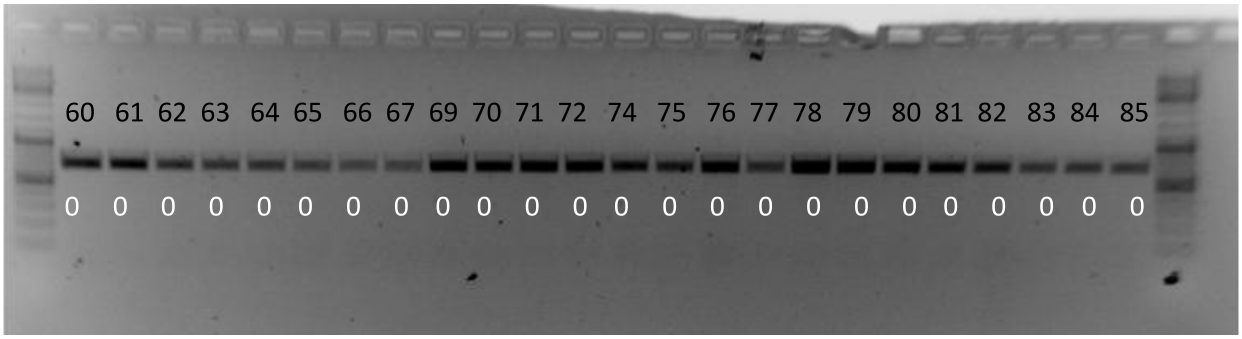

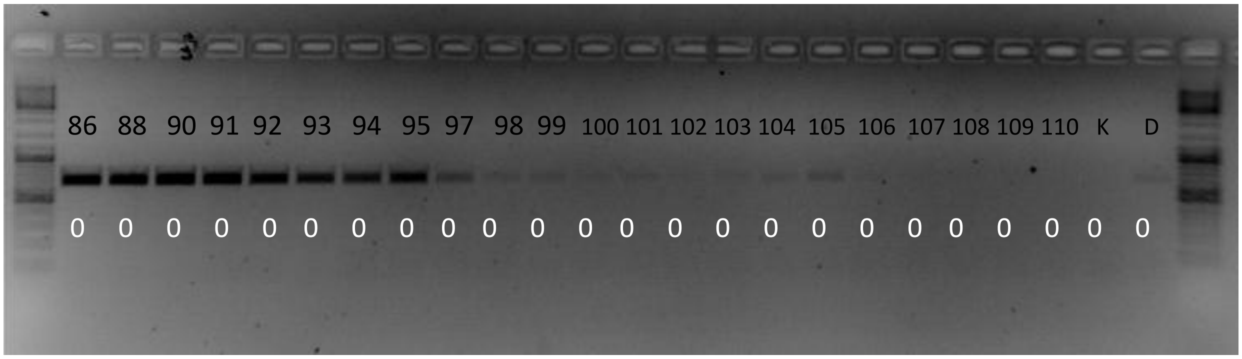

Plate 9

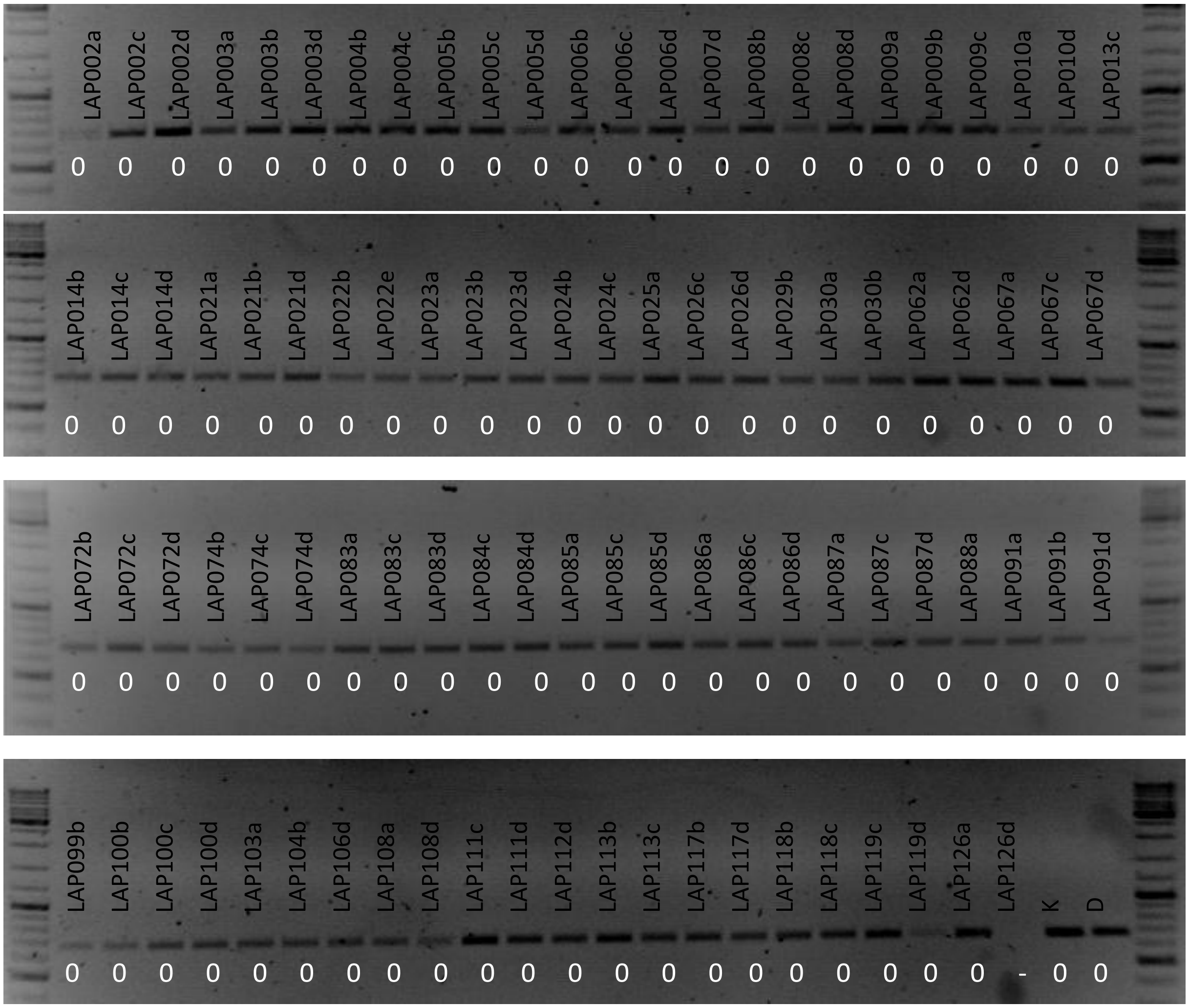
LAP002a LAP002c LAP002d LAP003a LAP003b LAP003d LAP004b LAP004c LAP005b LAP005c LAP005d LAP006b LAP006c LAP006d LAP007d LAP008b LAP008c LAP008d LAP009a LAP009b LAP009c LAP010a LAP010d LAP013c
0 0 0 0 0 0 0 0 0 0 0 0 0 0 0 0 0 0 0 0 0 0 0 0
LAP014b LAP014c LAP014d LAP021a LAP021b LAP021d LAP022b LAP022e LAP023a LAP023b LAP023d LAP024b LAP024c LAP025a LAP026c LAP026d LAP029b LAP030a LAP030b LAP062a LAP062d LAP067a LAP067c LAP067d
0 0 0 0 0 0 0 0 0 0 0 0 0 0 0 0 0 0 0 0 0 0 0 0
LAP072b LAP072c LAP072d LAP074b LAP074c LAP074d LAP083a LAP083c LAP083d LAP084c LAP084d LAP085a LAP085c LAP085d LAP086a LAP086c LAP086d LAP087a LAP087c LAP087d LAP088a LAP091a LAP091b LAP091d
0 0 0 0 0 0 0 0 0 0 0 0 0 0 0 0 0 0 0 0 0 0 0 0
LAP099b LAP100b LAP100c LAP100d LAP103a LAP104b LAP106d LAP108a LAP108d LAP111c LAP111d LAP112d LAP113b LAP113c LAP117b LAP117d LAP118b LAP118c LAP119c LAP119d LAP126a LAP126d K D
0 0 0 0 0 0 0 0 0 0 0 0 0 0 0 0 0 0 0 0 0 - 0 0

PR_22

PRFTA2F3 ACTAGTACATTTTTGTTTGGGAAGGA

PRFTa2_R3b TTGTCTAGTCCCAATTATGTTTGATT

Plate 1

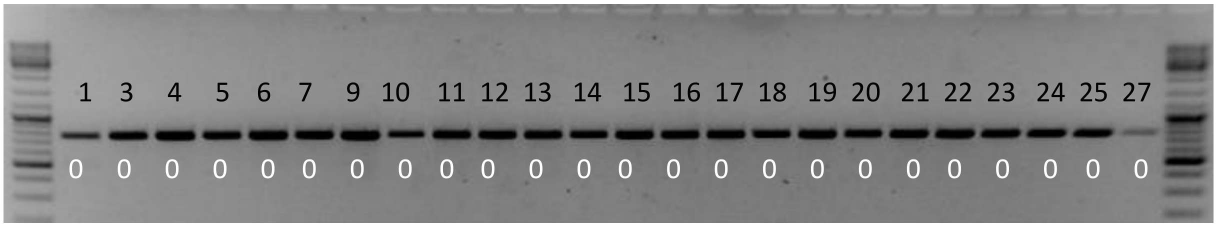

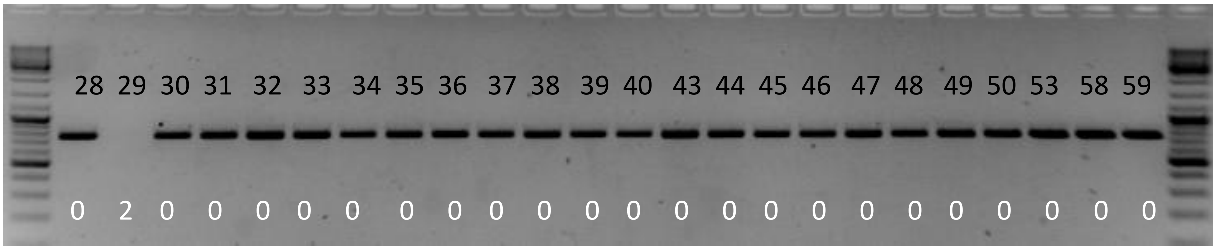

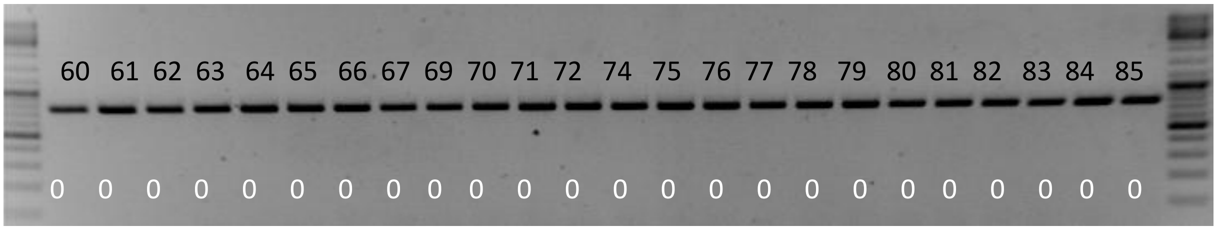

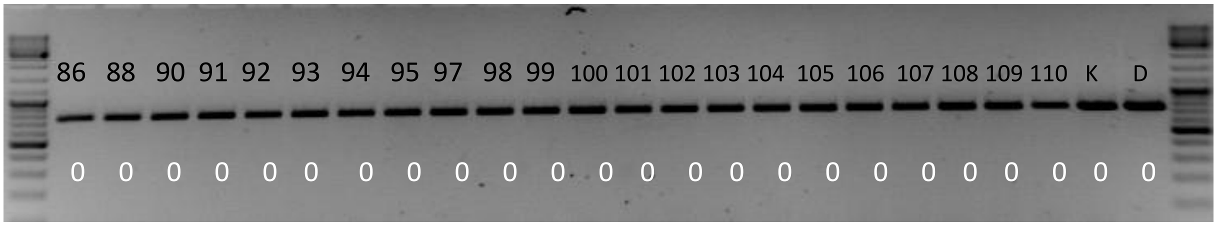

Plate 9

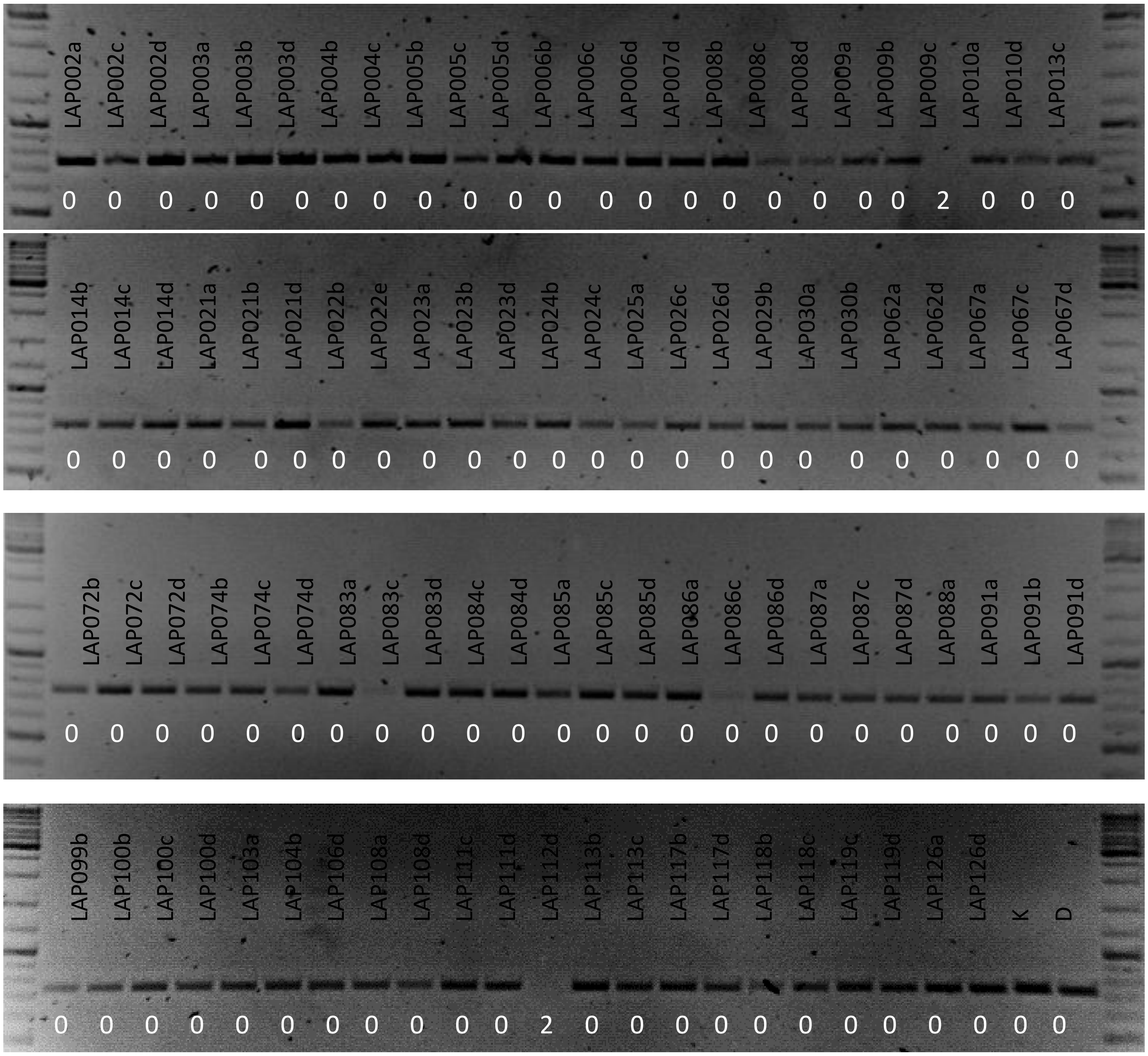

Repeat

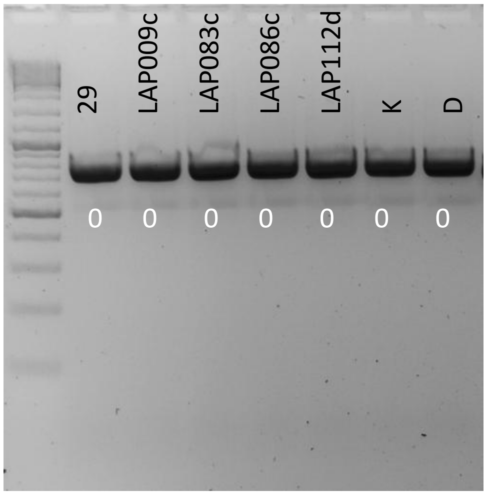

PR_23

PRFTa2_F3b AACCATACTATAAATGTTCCCTCCACT

PRFTa2_R3c TTGTAATCTCTATCTATCTTGCTCTCT

Plate 1

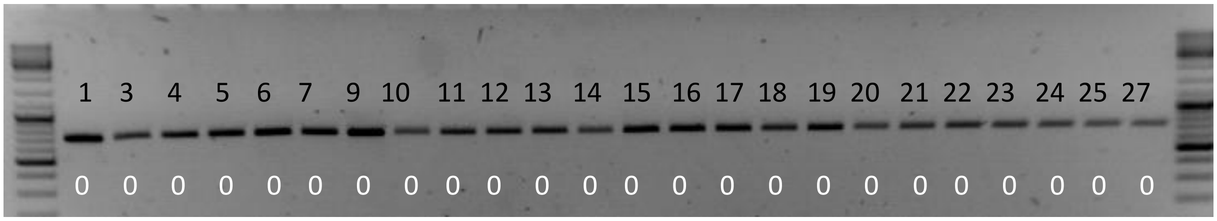

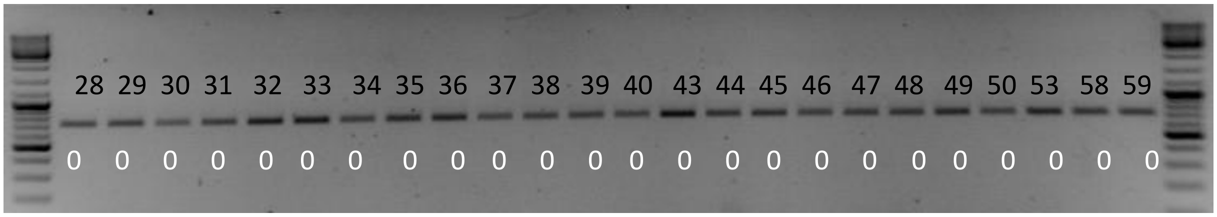

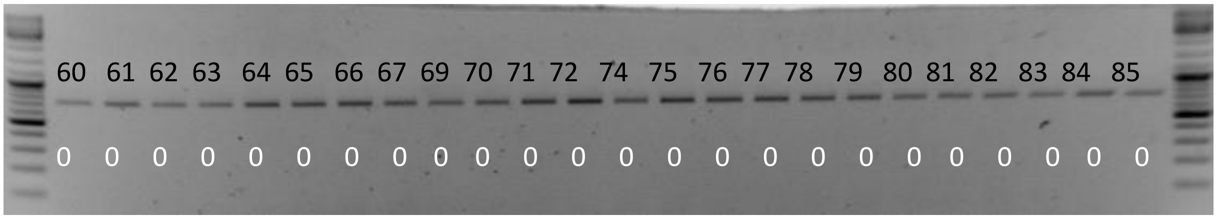

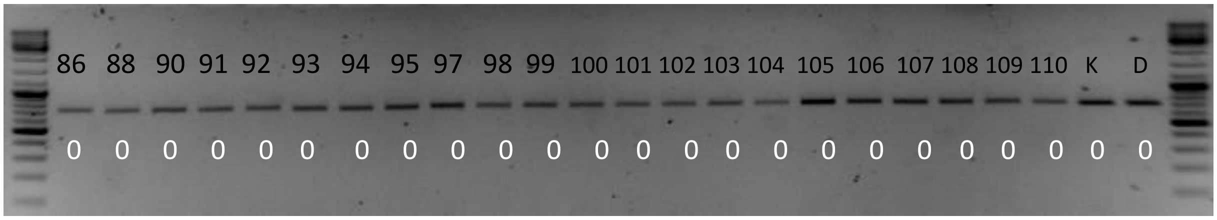

Plate 9

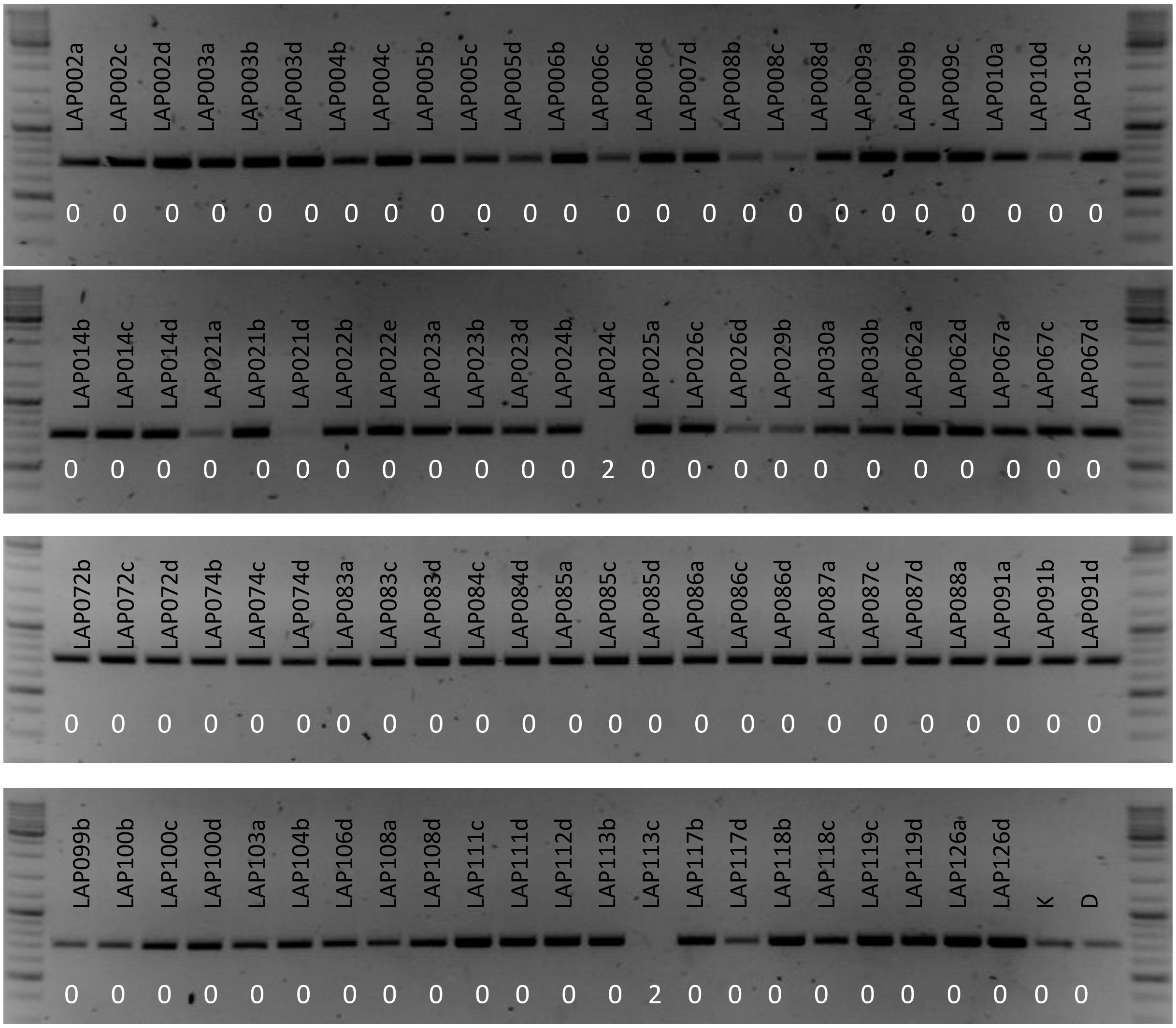

Repeat

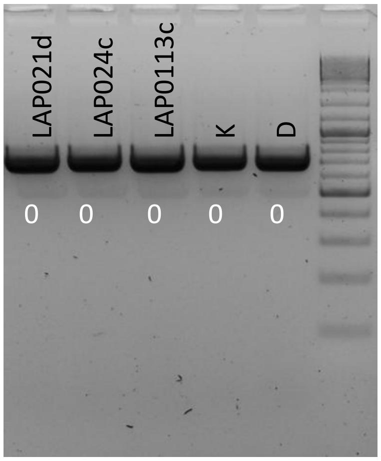

PR_24

PRFTa2_F3c CAGAGAATCGACCTTTGACTACCTT

PRFTA2R3 GAAACTCAACGGGTGGGATGA

Plate 1

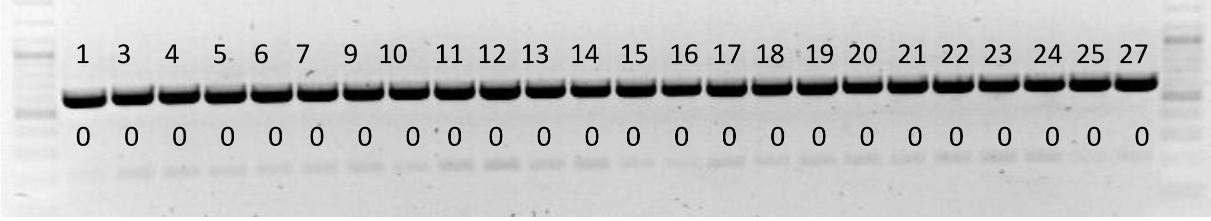

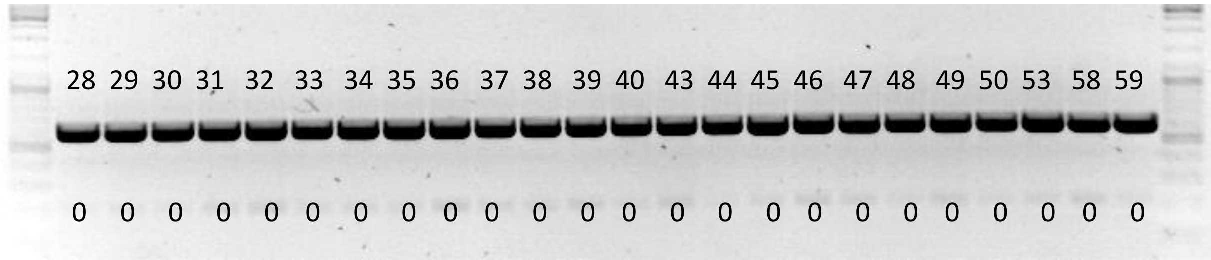

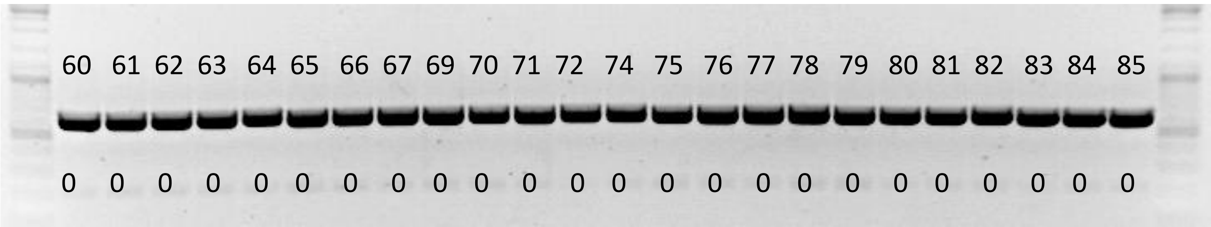

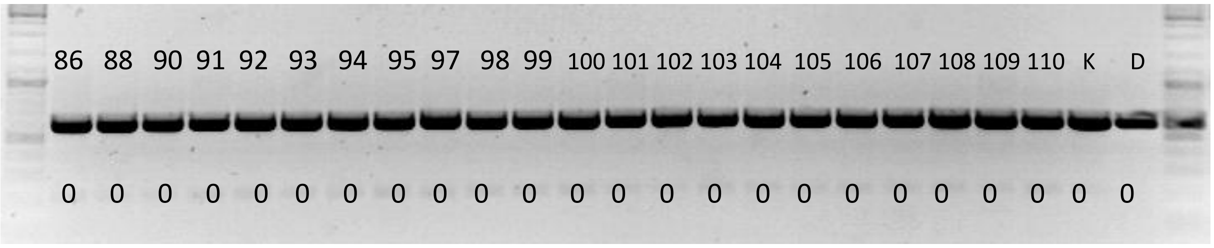

Plate 9

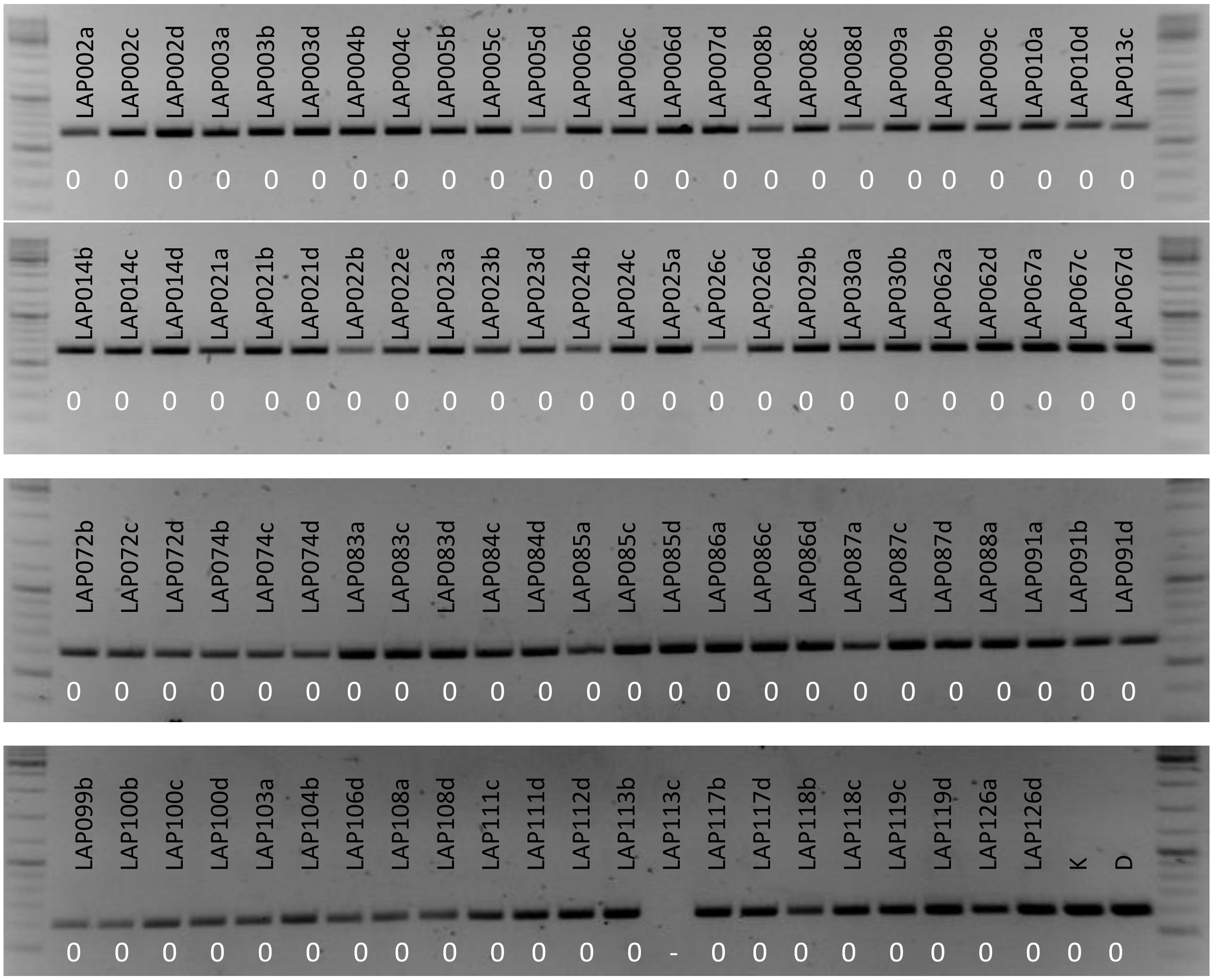
LAP002a LAP002c LAP002d LAP003a LAP003b LAP003d LAP004b LAP004c LAP005b LAP005c LAP005d LAP006b LAP006c LAP006d LAP007d LAP008b LAP008c LAP008d LAP009a LAP009b LAP009c LAP010a LAP010d LAP013c
0 0 0 0 0 0 0 0 0 0 0 0 0 0 0 0 0 0 0 0 0 0 0 0
LAP014b LAP014c LAP014d LAP021a LAP021b LAP021d LAP022b LAP022e LAP023a LAP023b LAP023d LAP024b LAP024c LAP025a LAP026c LAP026d LAP029b LAP030a LAP030b LAP062a LAP062d LAP067a LAP067c LAP067d
0 0 0 0 0 0 0 0 0 0 0 0 0 0 0 0 0 0 0 0 0 0 0 0
LAP072b LAP072c LAP072d LAP074b LAP074c LAP074d LAP083a LAP083c LAP083d LAP084c LAP084d LAP085a LAP085c LAP085d LAP086a LAP086c LAP086d LAP087a LAP087c LAP087d LAP088a LAP091a LAP091b LAP091d
0 0 0 0 0 0 0 0 0 0 0 0 0 0 0 0 0 0 0 0 0 0 0 0
LAP099b LAP100b LAP100c LAP100d LAP103a LAP104b LAP106d LAP108a LAP108d LAP111c LAP111d LAP112d LAP113b LAP113c LAP117b LAP117d LAP118b LAP118c LAP119c LAP119d LAP126a LAP126d K D
0 0 0 0 0 0 0 0 0 0 0 0 0 - 0 0 0 0 0 0 0 0 0 0

PR_25

PRFTA2F4 AGGGCTCGATTTTGGCTTACT

PRFTa2_R4b GATCTTAAACCTTCGTACCCTCACT

Plate 1

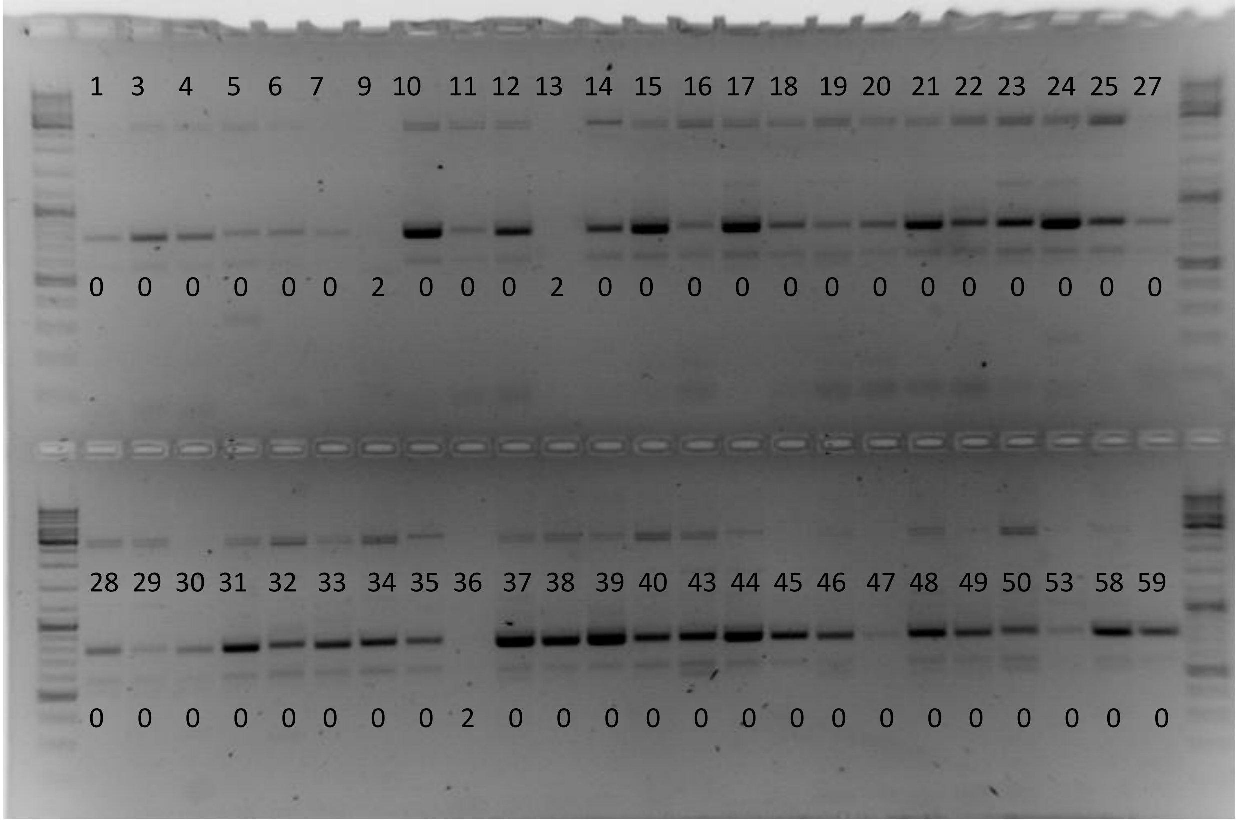

Plate 9

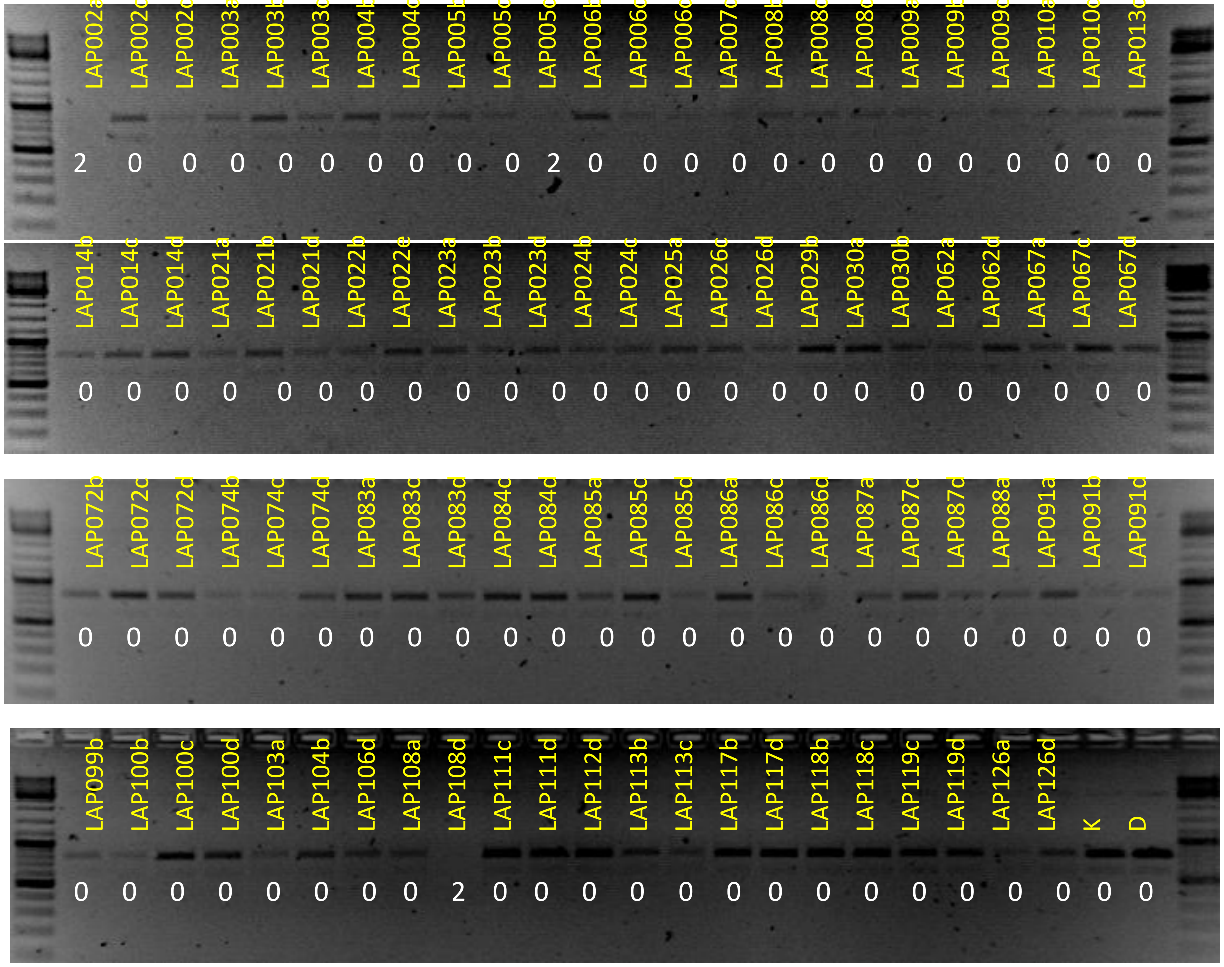

Repeat

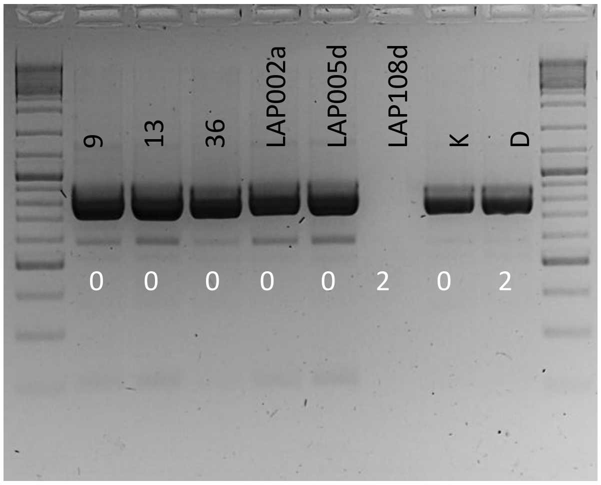

PR_26

PRFTa2_F4b TCTTTAGGCTCTAAATCTCAAGTAGA

PRFTa2_R4c TCAATACGTCTTCATTATTTGACTCGT

Plate 1

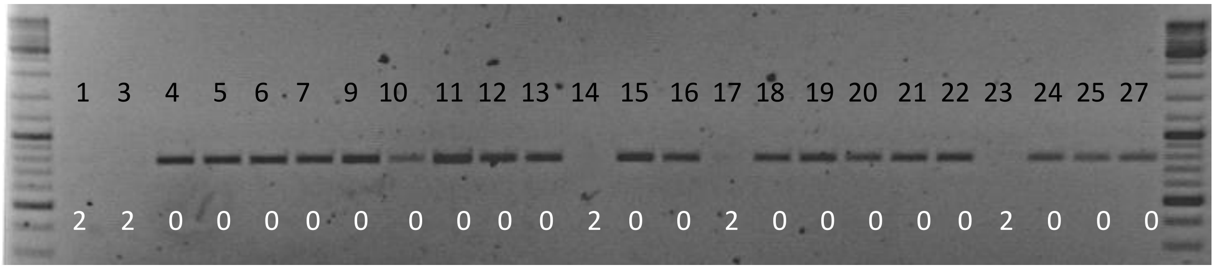

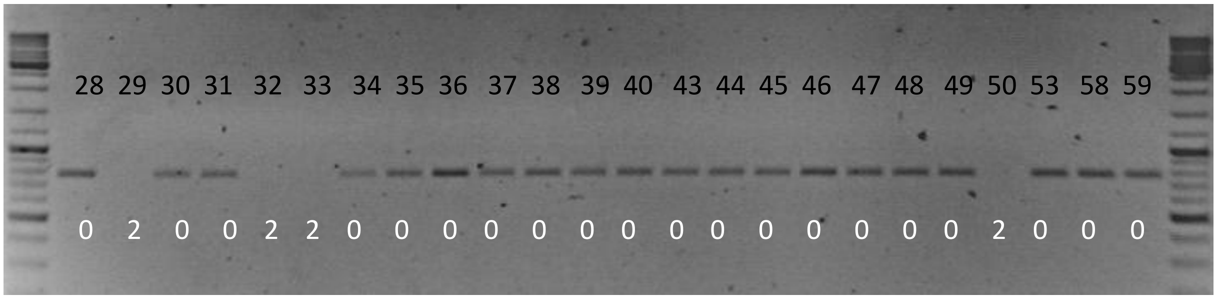

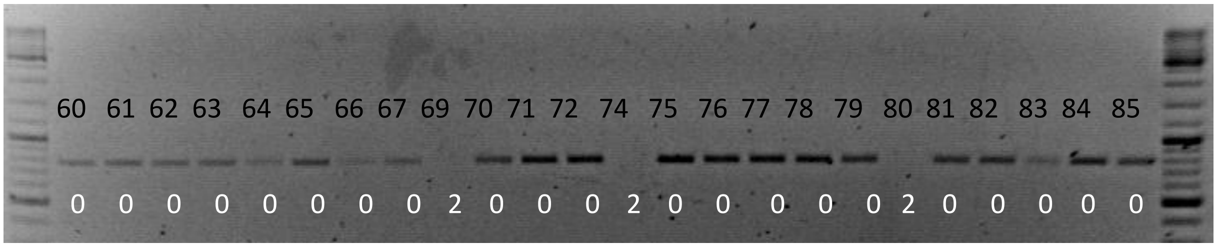

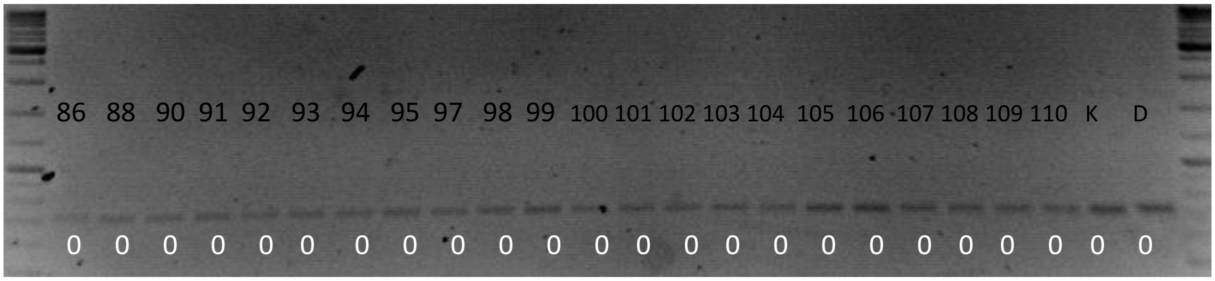

Plate 9

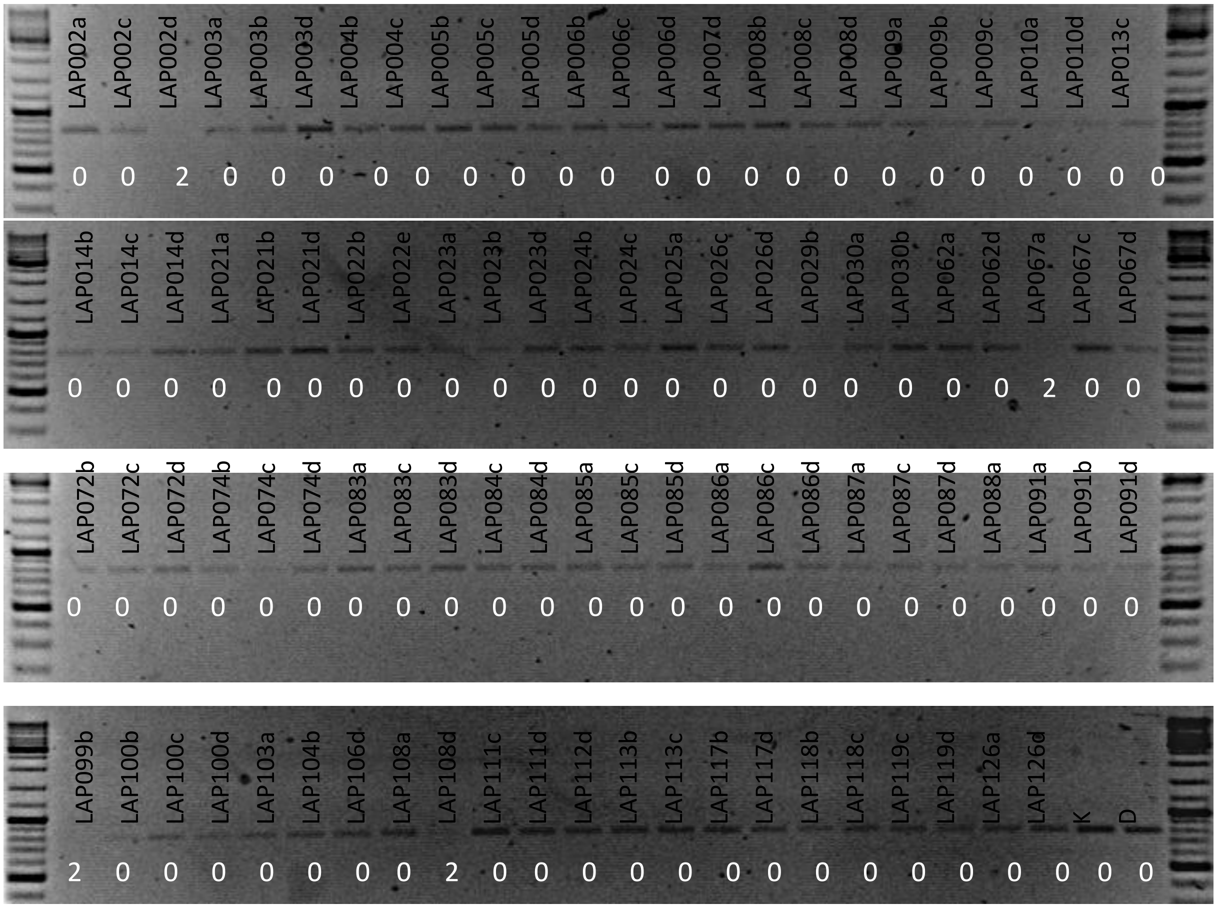

Repeat

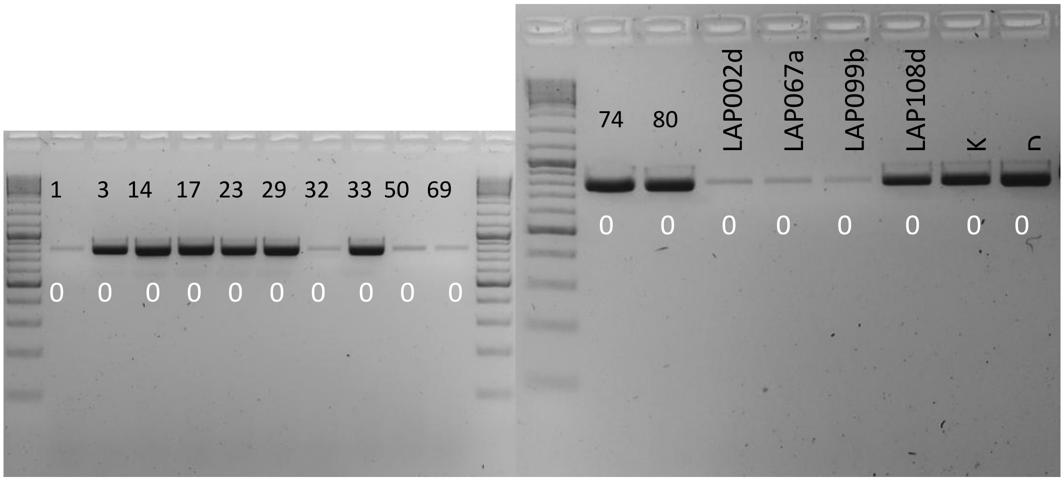

PR_27

PRFTa2_F4c CAATCATCTCTCCTCCTACACGAAT

PRFTA2R4 ACTCTAACCGCTTTGCAAACA

Plate 1

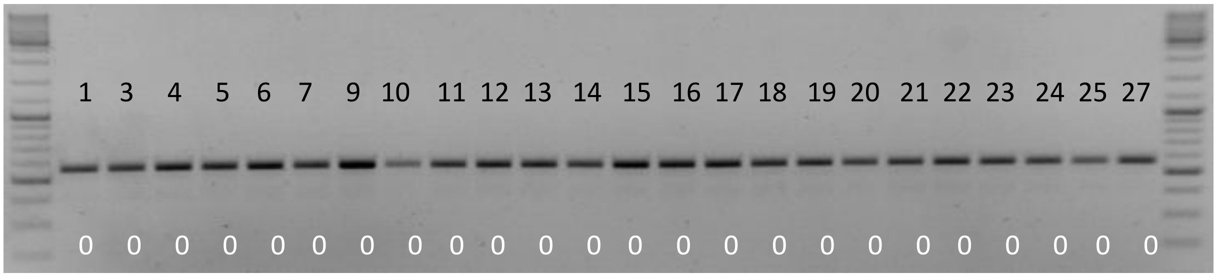

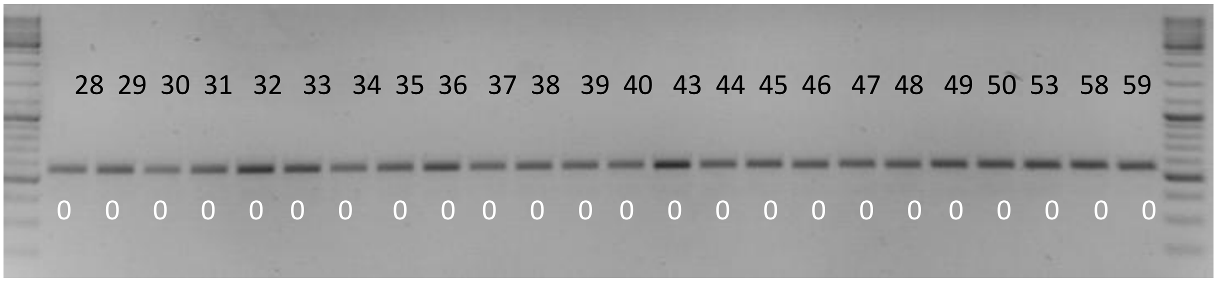

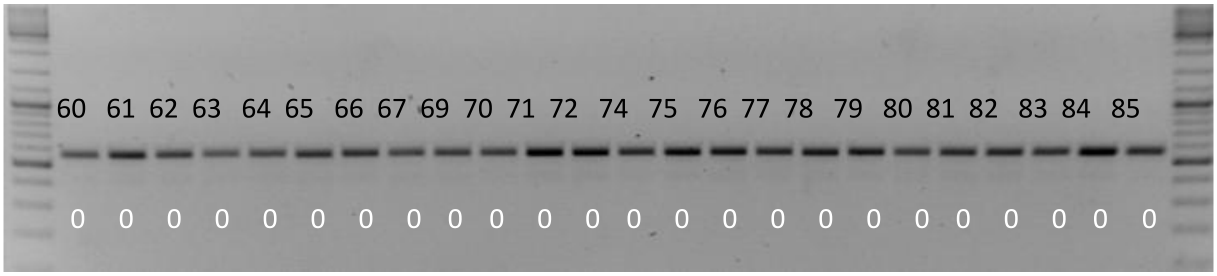

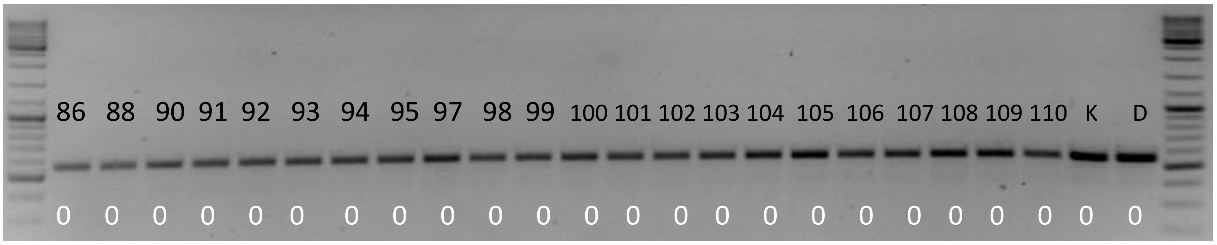

Plate 9

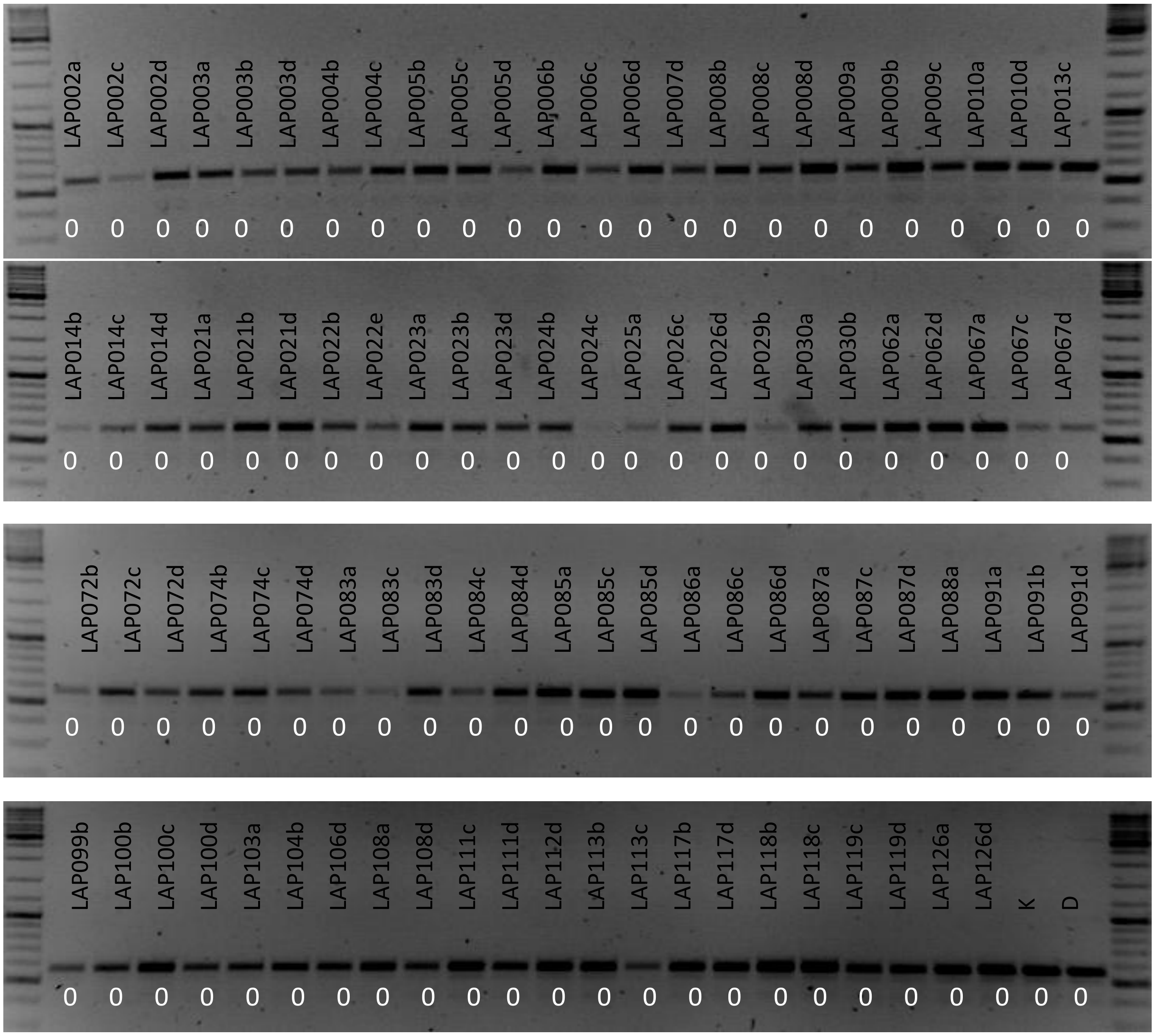

LAP002a LAP002c LAP002d LAP003a LAP003b LAP003d LAP004b LAP004c LAP005b LAP005c LAP005d LAP006b LAP006c LAP006d LAP007d LAP008b LAP008c LAP008d LAP009a LAP009b LAP009c LAP010a LAP010d LAP013c
0 0 0 0 0 0 0 0 0 0 0 0 0 0 0 0 0 0 0 0 0 0 0 0
LAP014b LAP014c LAP014d LAP021a LAP021b LAP021d LAP022b LAP022e LAP023a LAP023b LAP023d LAP024b LAP024c LAP025a LAP026c LAP026d LAP029b LAP030a LAP030b LAP062a LAP062d LAP067a LAP067c LAP067d
0 0 0 0 0 0 0 0 0 0 0 0 0 0 0 0 0 0 0 0 0 0 0 0
LAP072b LAP072c LAP072d LAP074b LAP074c LAP074d LAP083a LAP083c LAP083d LAP084c LAP084d LAP085a LAP085c LAP085d LAP086a LAP086c LAP086d LAP087a LAP087c LAP087d LAP088a LAP091a LAP091b LAP091d
0 0 0 0 0 0 0 0 0 0 0 0 0 0 0 0 0 0 0 0 0 0 0 0
LAP099b LAP100b LAP100c LAP100d LAP103a LAP104b LAP106d LAP108a LAP108d LAP111c LAP111d LAP112d LAP113b LAP113c LAP117b LAP117d LAP118b LAP118c LAP119c LAP119d LAP126a LAP126d K D
0 0 0 0 0 0 0 0 0 0 0 0 0 0 0 0 0 0 0 0 0 0 0 0

PR_28

PRFTA2F5 AAAATCAGGGGTGGTTCGTGT

PRFTa2_R5b TCATGCATGTTAGCCATTATTACAA

Plate 1

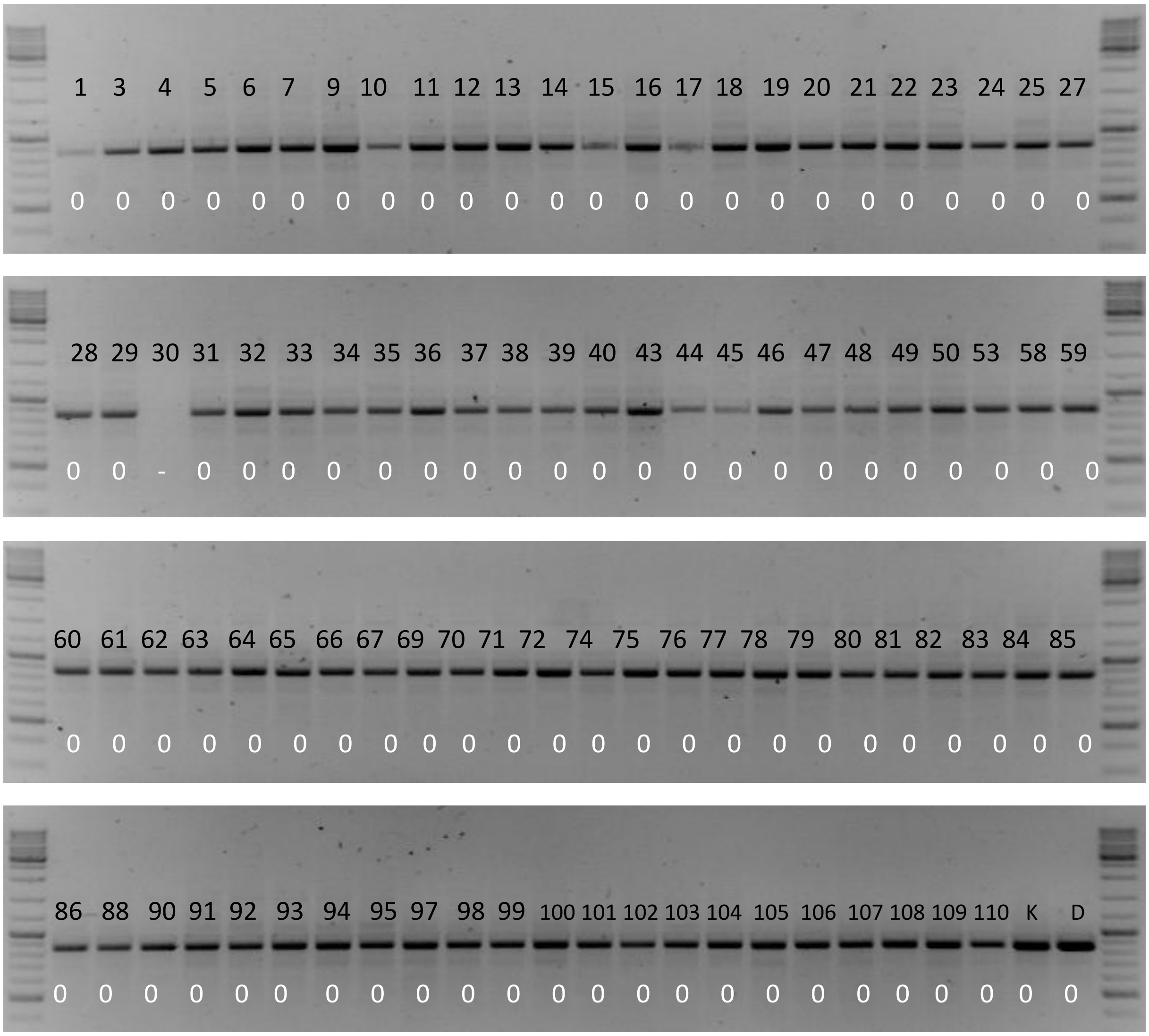

Plate 9

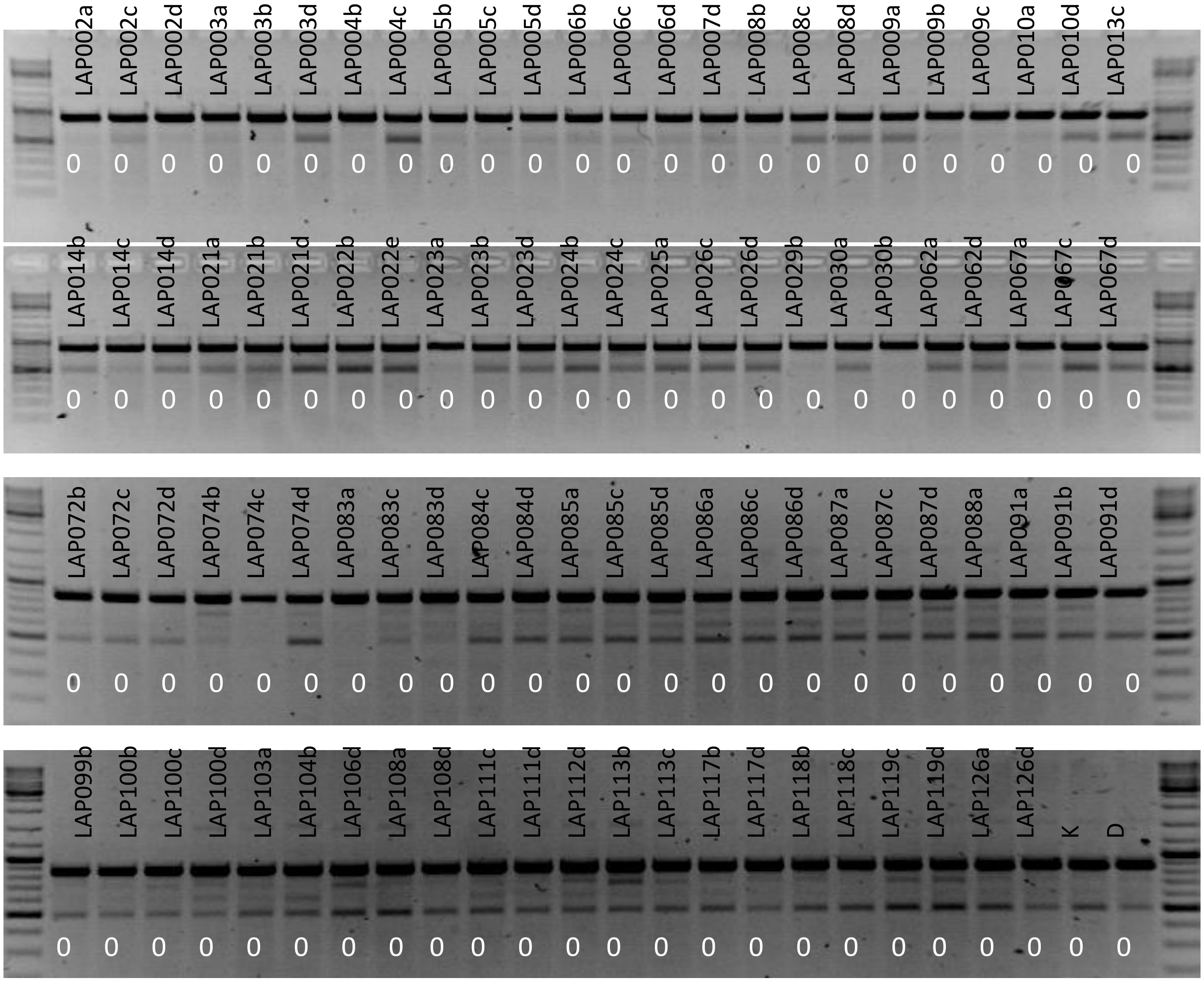
LAP002a LAP002c LAP002d LAP003a LAP003b LAP003d LAP004b LAP004c LAP005b LAP005c LAP005d LAP006b LAP006c LAP006d LAP007d LAP008b LAP008c LAP008d LAP009a LAP009b LAP009c LAP010a LAP010d LAP013c
0 0 0 0 0 0 0 0 0 0 0 0 0 0 0 0 0 0 0 0 0 0 0 0
LAP014b LAP014c LAP014d LAP021a LAP021b LAP021d LAP022b LAP022e LAP023a LAP023b LAP023d LAP024b LAP024c LAP025a LAP026c LAP026d LAP029b LAP030a LAP030b LAP062a LAP062d LAP067a LAP067c LAP067d
0 0 0 0 0 0 0 0 0 0 0 0 0 0 0 0 0 0 0 0 0 0 0 0
LAP072b LAP072c LAP072d LAP074b LAP074c LAP074d LAP083a LAP083c LAP083d LAP084c LAP084d LAP085a LAP085c LAP085d LAP086a LAP086c LAP086d LAP087a LAP087c LAP087d LAP088a LAP091a LAP091b LAP091d
0 0 0 0 0 0 0 0 0 0 0 0 0 0 0 0 0 0 0 0 0 0 0 0
LAP099b LAP100b LAP100c LAP100d LAP103a LAP104b LAP106d LAP108a LAP108d LAP111c LAP111d LAP112d LAP113b LAP113c LAP117b LAP117d LAP118b LAP118c LAP119c LAP119d LAP126a LAP126d K D
0 0 0 0 0 0 0 0 0 0 0 0 0 0 0 0 0 0 0 0 0 0 0 0

PR_29

PRFTa2_F5b TGGACTCCAAATAAGGATCATGTGT

PRFTA2R5 CTCCACCGATACATACTCTTGGT

Plate 1

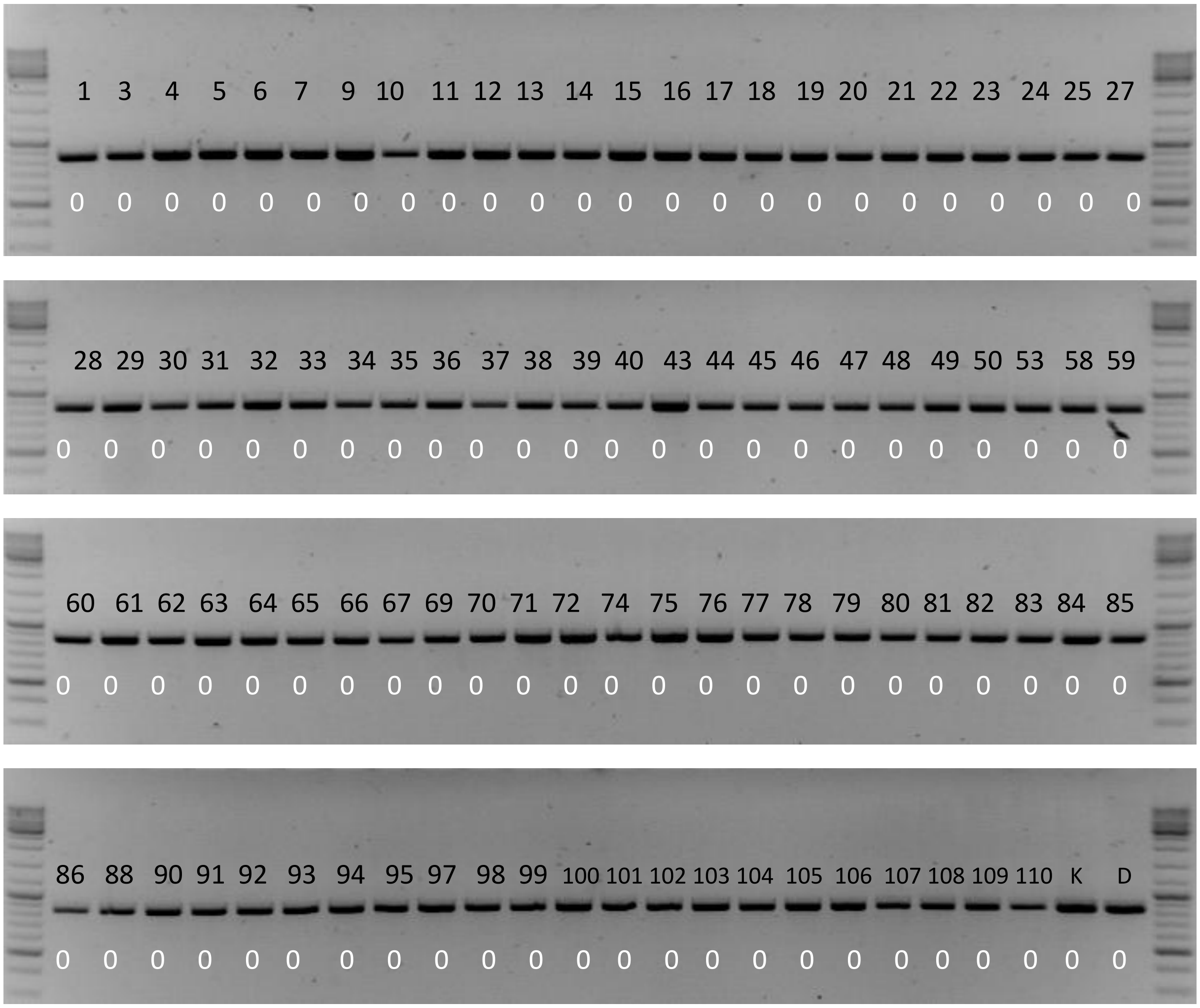

Plate 9

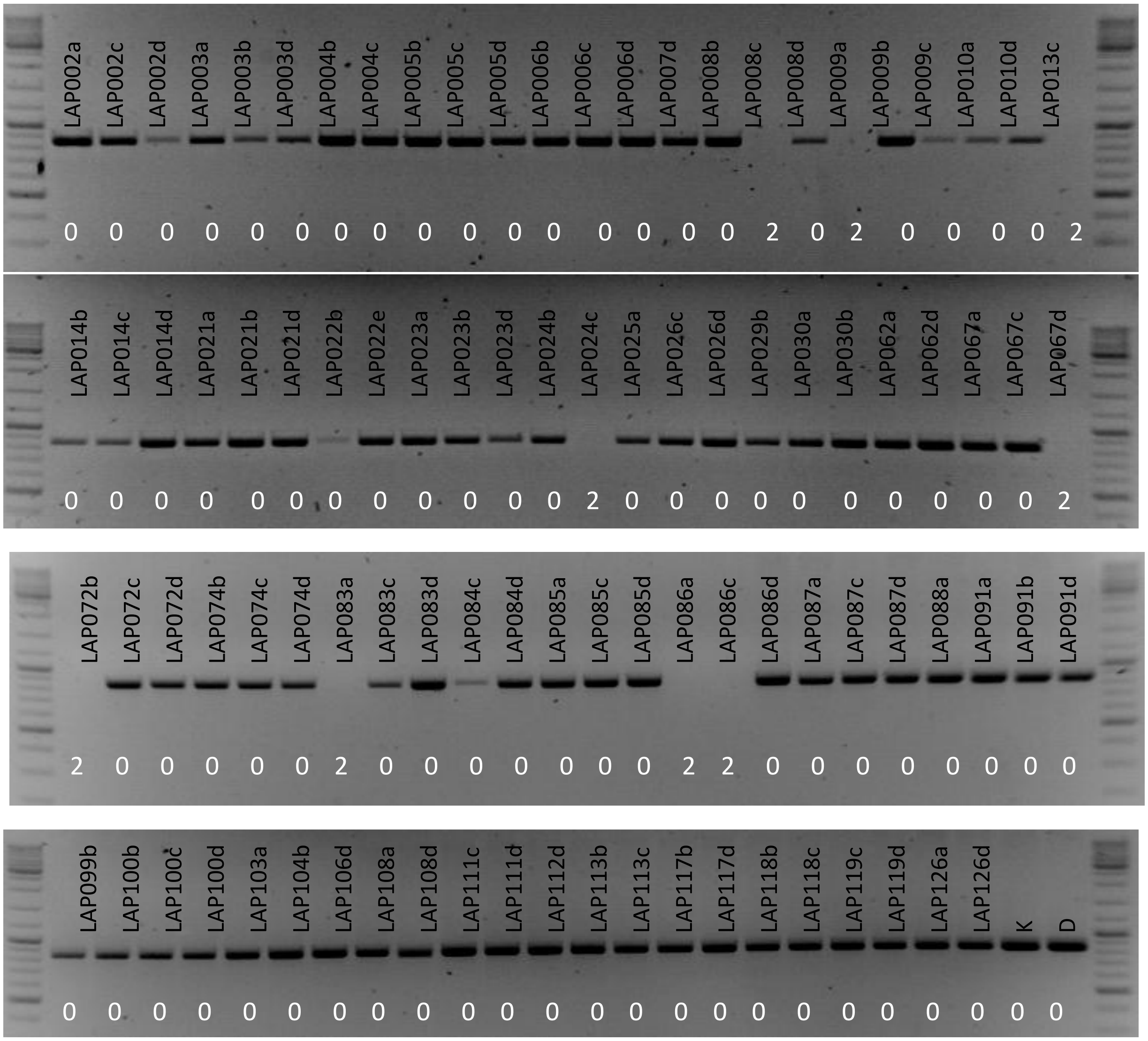

Repeat

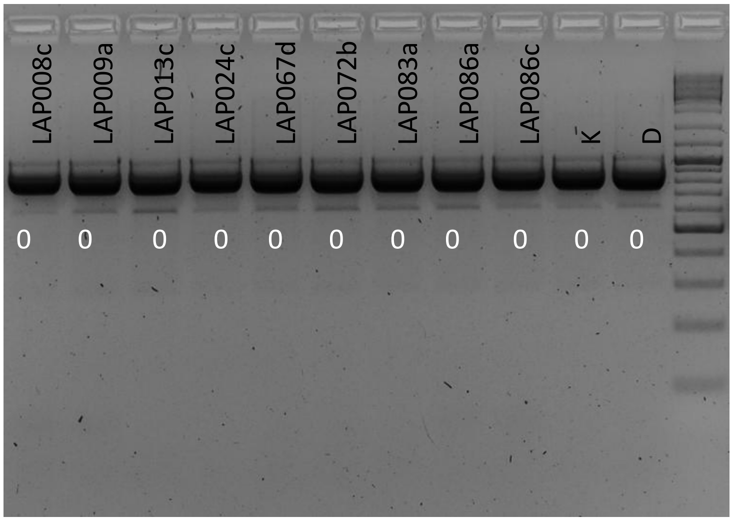

PR77

PRFTA2F5 AAAATCAGGGGTGGTTCGTGT

PRFTA2R4 ACTCTAACCGCTTTGCAAACA

Plate 1

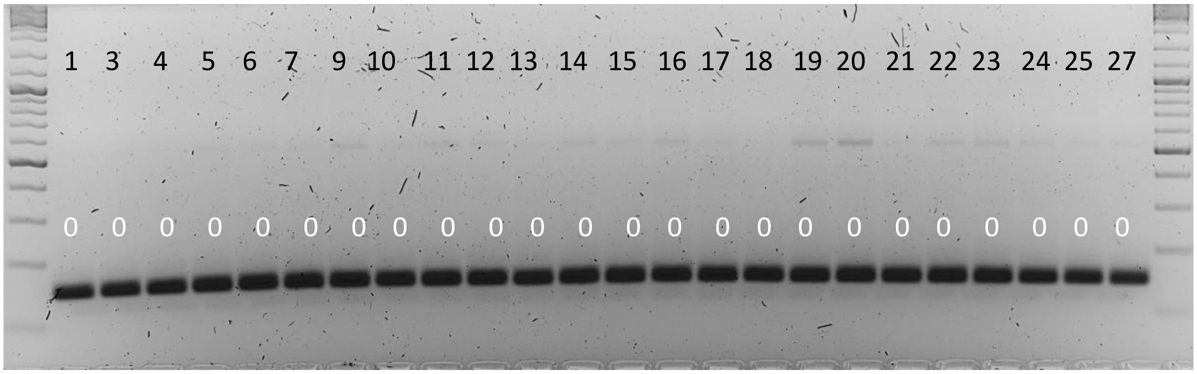

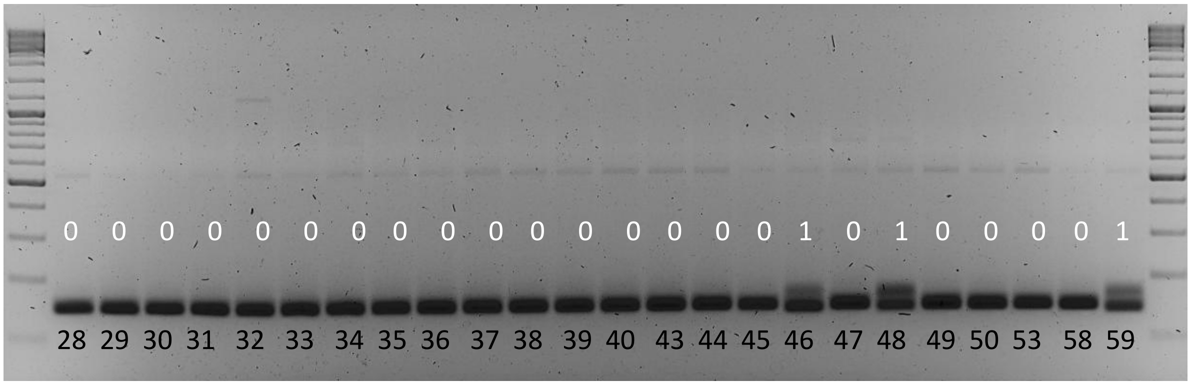

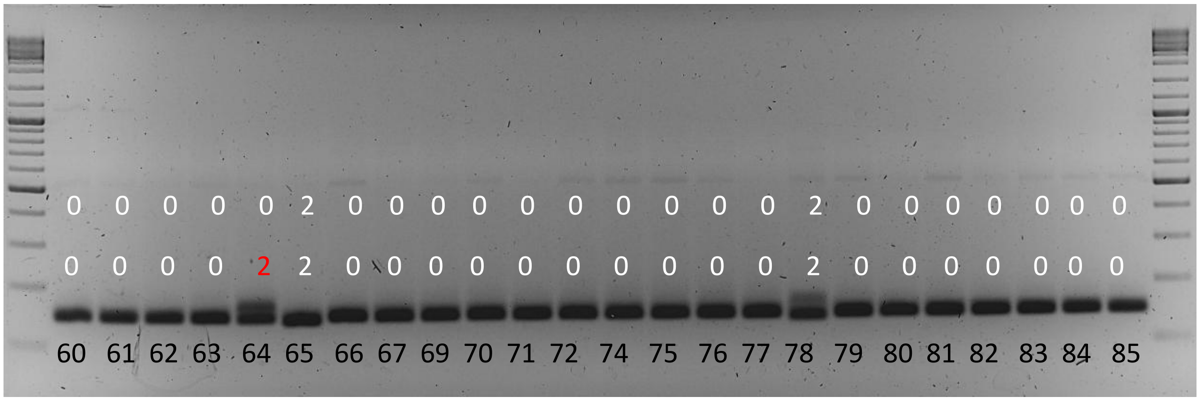

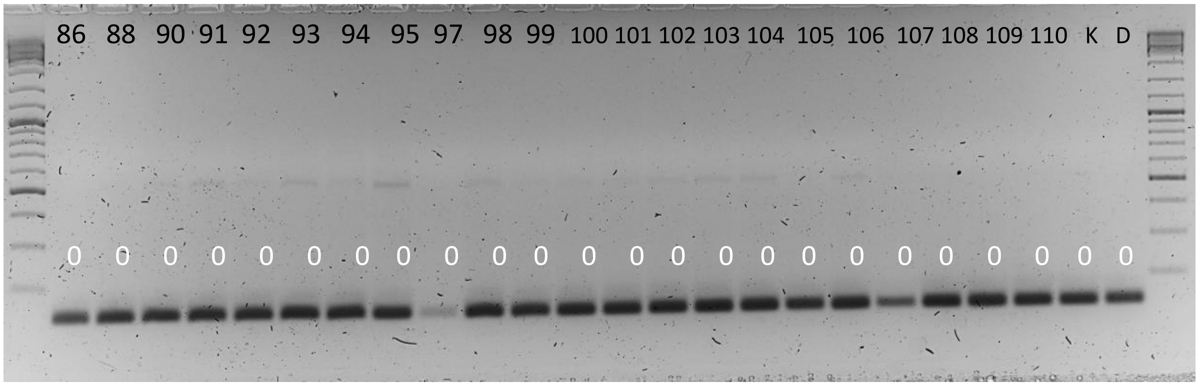

Plate 7

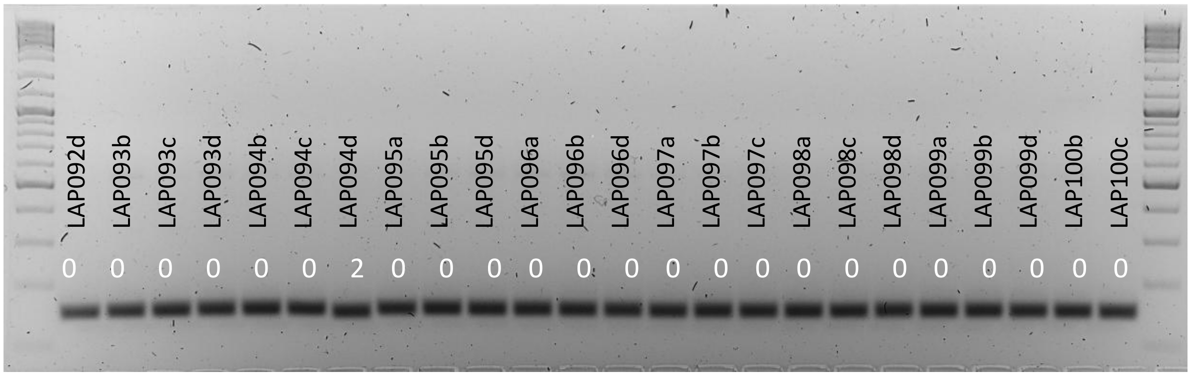
LAP092d LAP093b LAP093c LAP093d LAP094b LAP094c LAP094d LAP095a LAP095b LAP095d LAP096a LAP096b LAP096d LAP097a LAP097b LAP097c LAP098a LAP098c LAP098d LAP099a LAP099b LAP099d LAP100b LAP100c
0 0 0 0 0 0 2 0 0 0 0 0 0 0 0 0 0 0 0 0 0 0 0 0

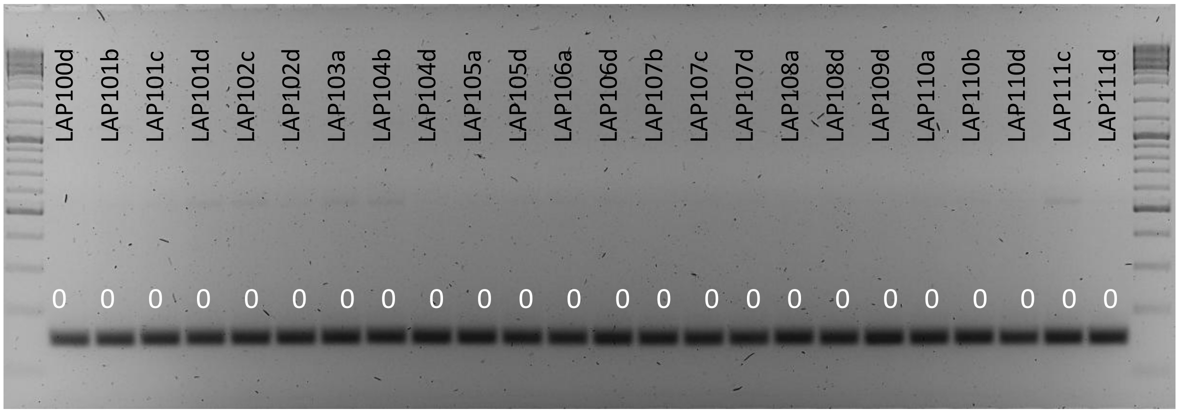
LAP100d LAP101b LAP101c LAP101d LAP102c LAP102d LAP103a LAP104b LAP104d LAP105a LAP105d LAP106a LAP106d LAP107b LAP107c LAP107d LAP108a LAP108d LAP109d LAP110a LAP110b LAP110d LAP111c LAP111d
0 0 0 0 0 0 0 0 0 0 0 0 0 0 0 0 0 0 0 0 0 0 0 0

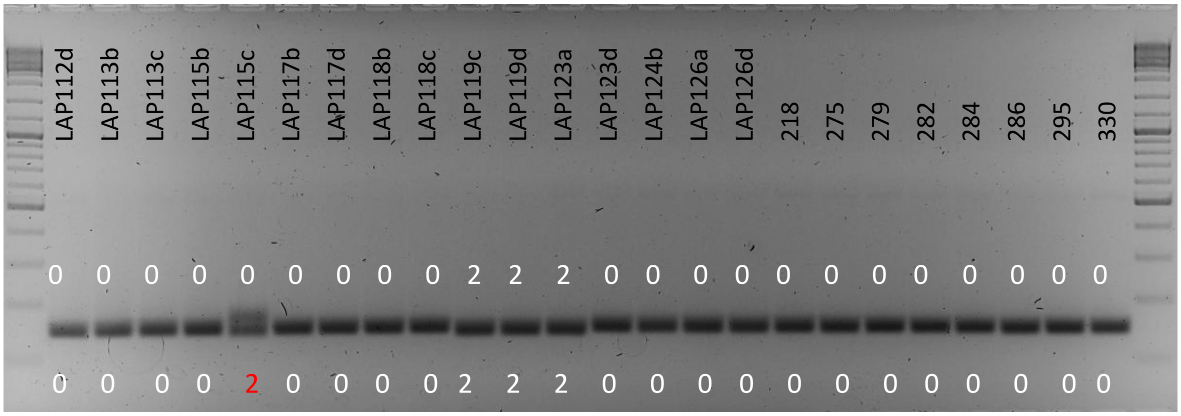
LAP112d LAP113b LAP113c LAP115b LAP115c LAP117b LAP117d LAP118b LAP118c LAP119c LAP119d LAP123a LAP123d LAP124b LAP126a LAP126d 218 275 279 282 284 286 295 330
0 0 0 0 0 0 0 0 0 2 2 2 0 0 0 0 0 0 0 0 0 0 0 0
0 0 0 0 2 0 0 0 0 2 2 2 0 0 0 0 0 0 0 0 0 0 0 0

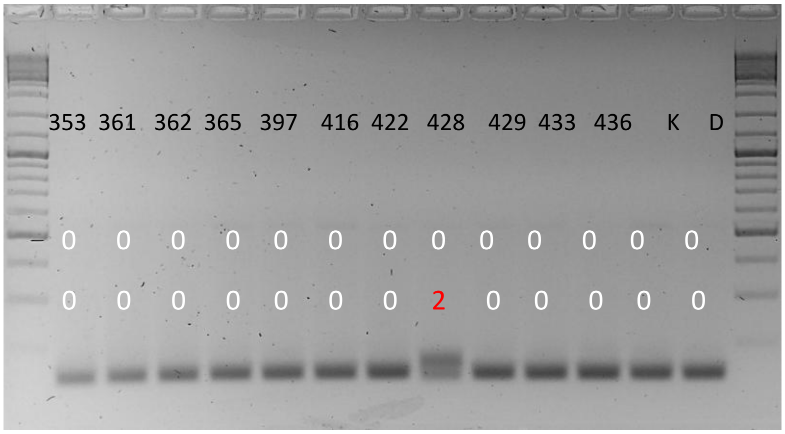
353 361 362 365 397 416 422 428 429 433 436 K D
0 0 0 0 0 0 0 0 0 0 0 0 0
0 0 0 0 0 0 0 2 0 0 0 0 0

PR78

PRFTA2F6 GGGCCTACTTTCGGTATATATTATCA

PRFTA2R6 TCGGGGTGTGTGATTATCCTT

Plate 1

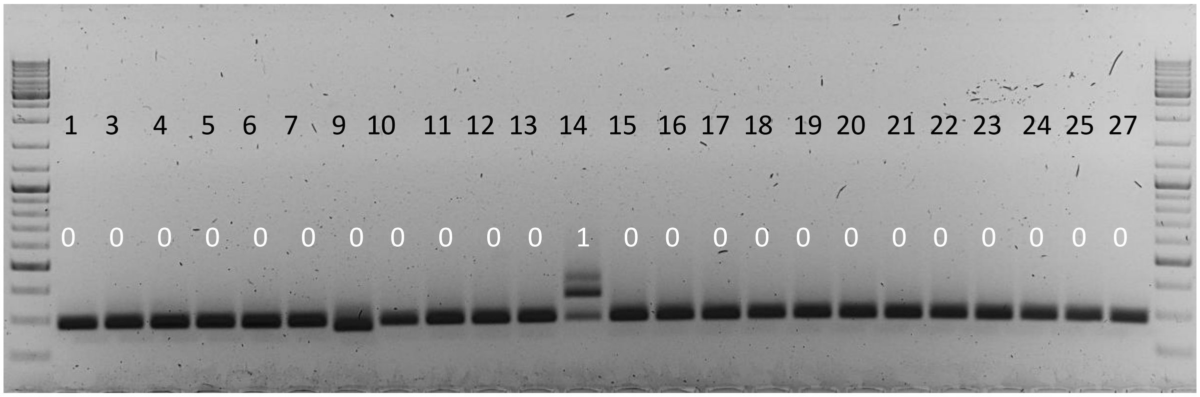

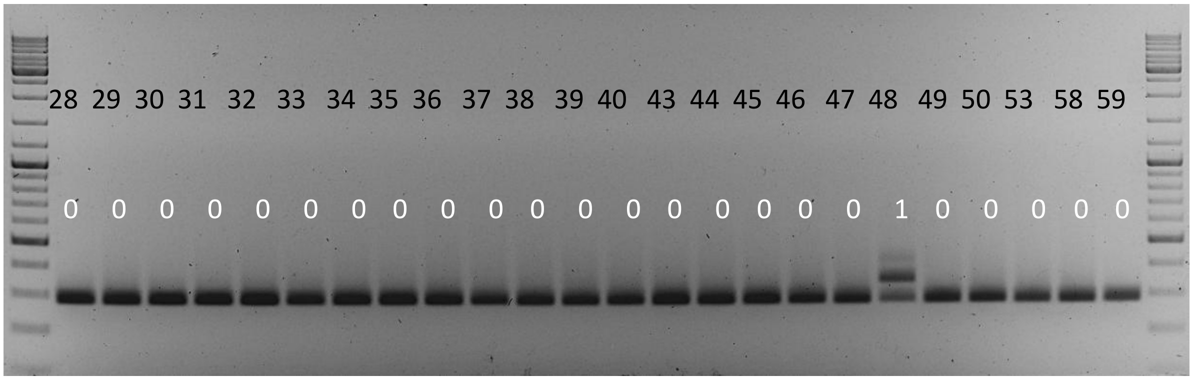

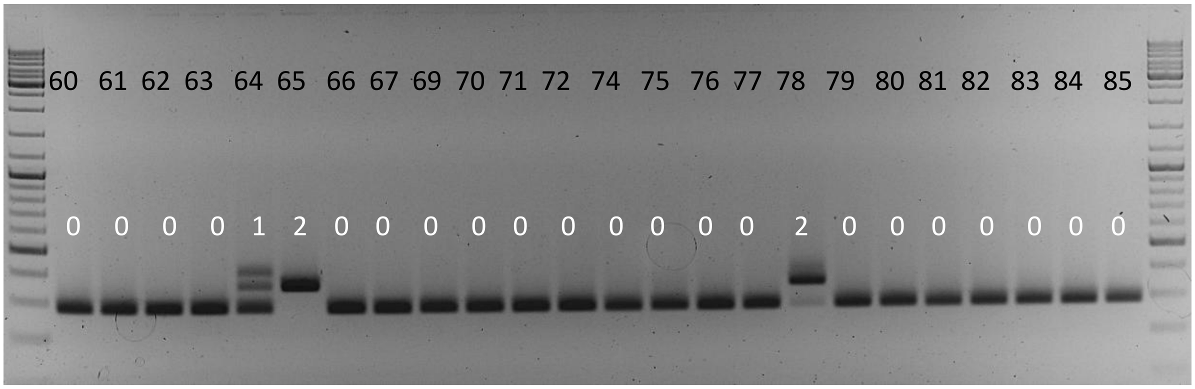

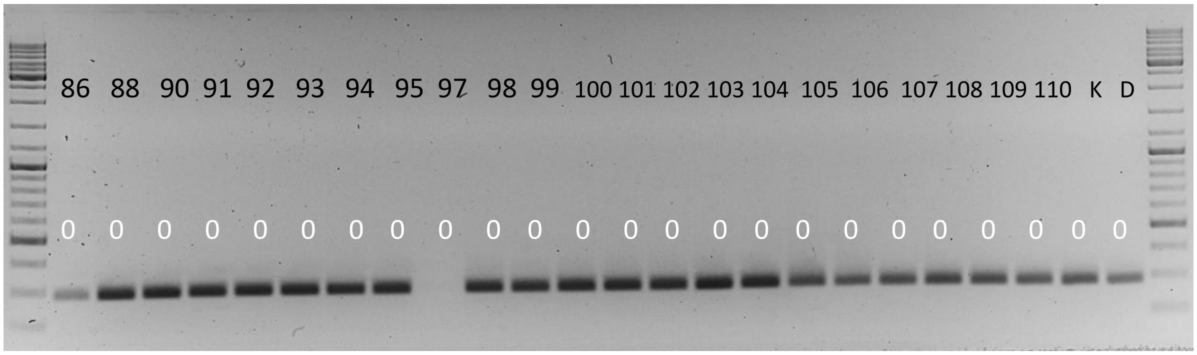

Plate 7

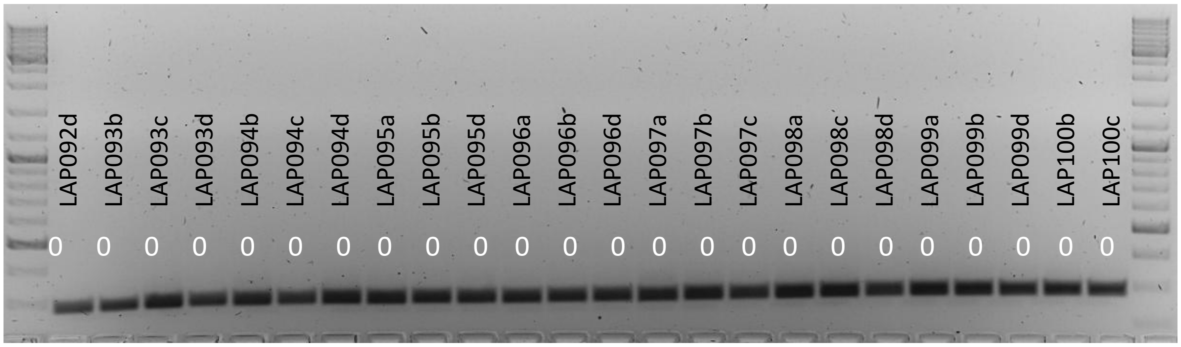
LAP092d LAP093b LAP093c LAP093d LAP094b LAP094c LAP094d LAP095a LAP095b LAP095d LAP096a LAP096b LAP096d LAP097a LAP097b LAP097c LAP098a LAP098c LAP098d LAP099a LAP099b LAP099d LAP100b LAP100c
0 0 0 0 0 0 0 0 0 0 0 0 0 0 0 0 0 0 0 0 0 0 0 0

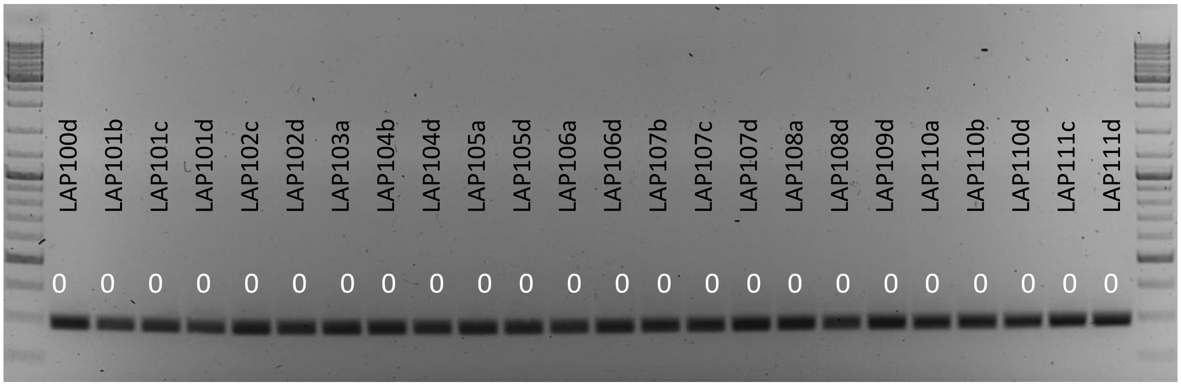
LAP100d LAP101b LAP101c LAP101d LAP102c LAP102d LAP103a LAP104b LAP104d LAP105a LAP105d LAP106a LAP106d LAP107b LAP107c LAP107d LAP108a LAP108d LAP109d LAP110a LAP110b LAP110d LAP111c LAP111d
0 0 0 0 0 0 0 0 0 0 0 0 0 0 0 0 0 0 0 0 0 0 0 0

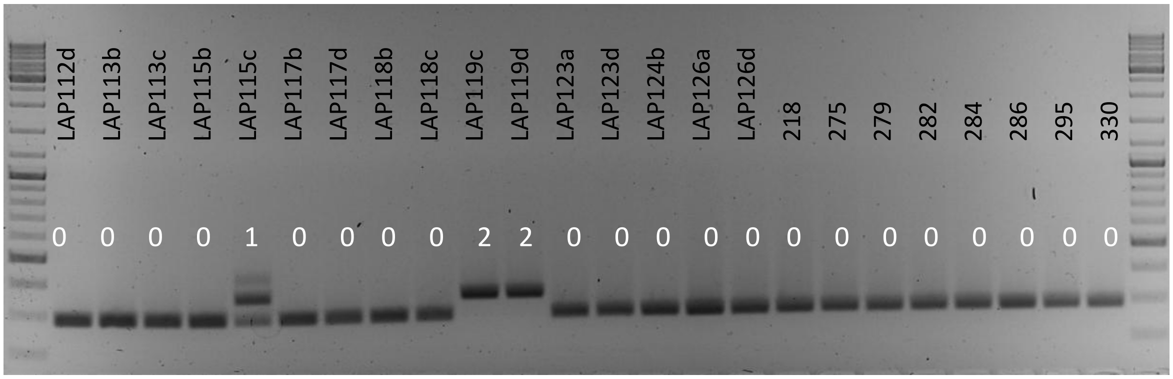
LAP112d LAP113b LAP113c LAP115b LAP115c LAP117b LAP117d LAP118b LAP118c LAP119c LAP119d LAP123a LAP123d LAP124b LAP126a LAP126d 218 275 279 282 284 286 295 330
0 0 0 0 1 0 0 0 0 2 2 0 0 0 0 0 0 0 0 0 0 0 0 0

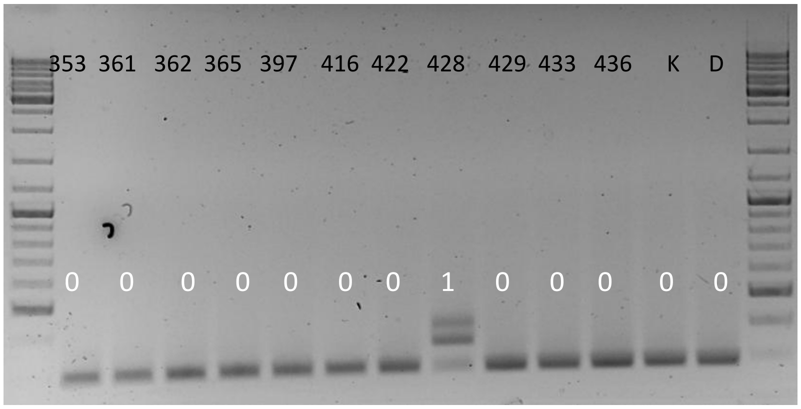
353 361 362 365 397 416 422 428 429 433 436 K D
0 0 0 0 0 0 0 1 0 0 0 0 0

Repeat

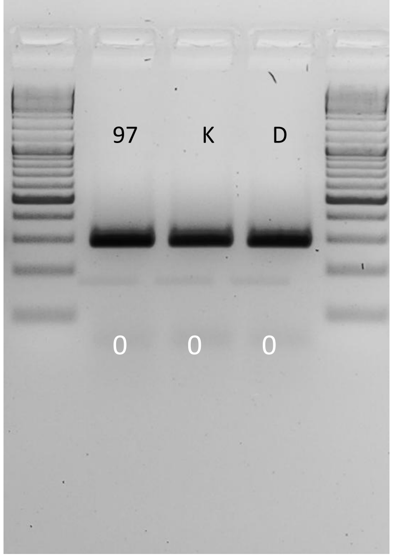

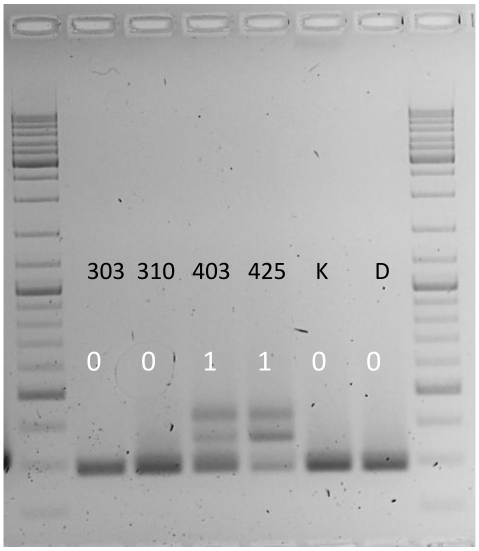

PR79

PRFTA2F7 TGTATTTGATTAATTTCTTTTGGTGTACA

PRFTA2R7 AAATTACAACTTATGCGGACGAG

Plate 1

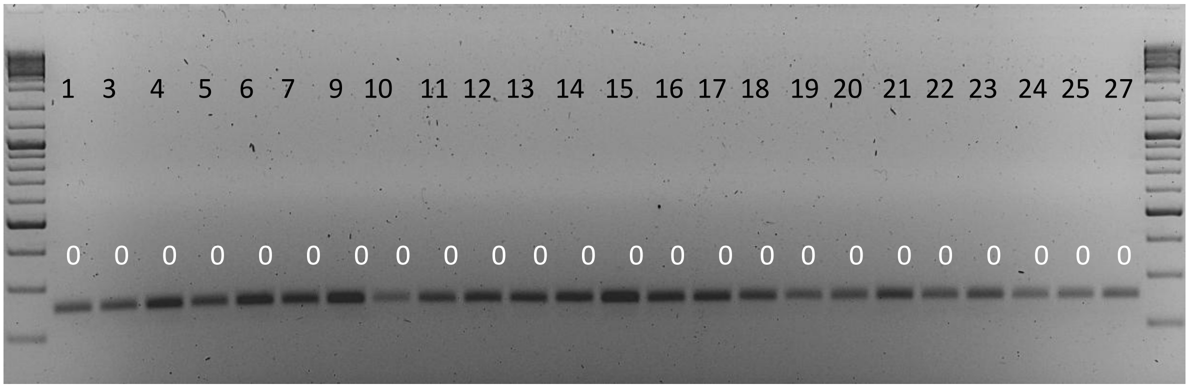

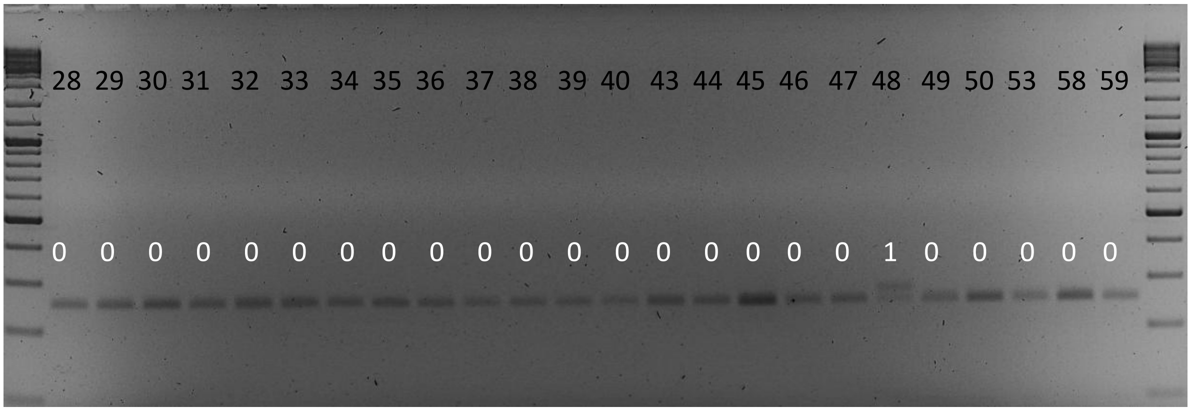

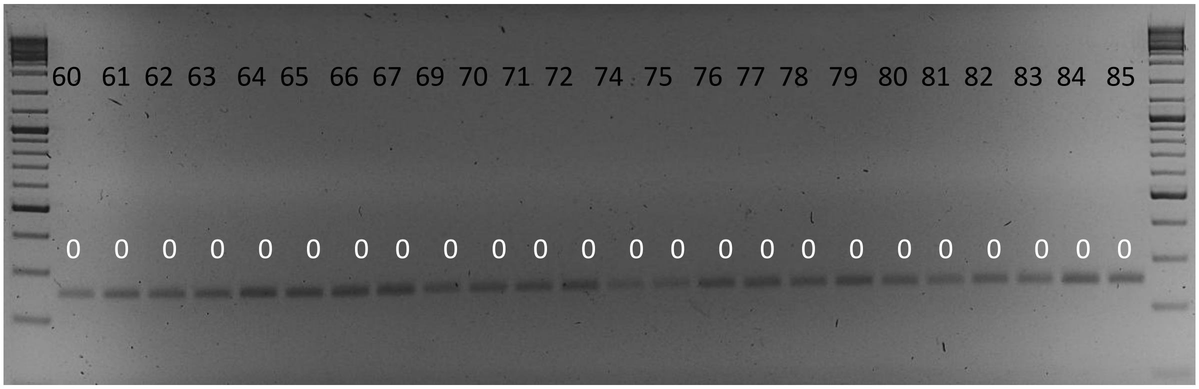

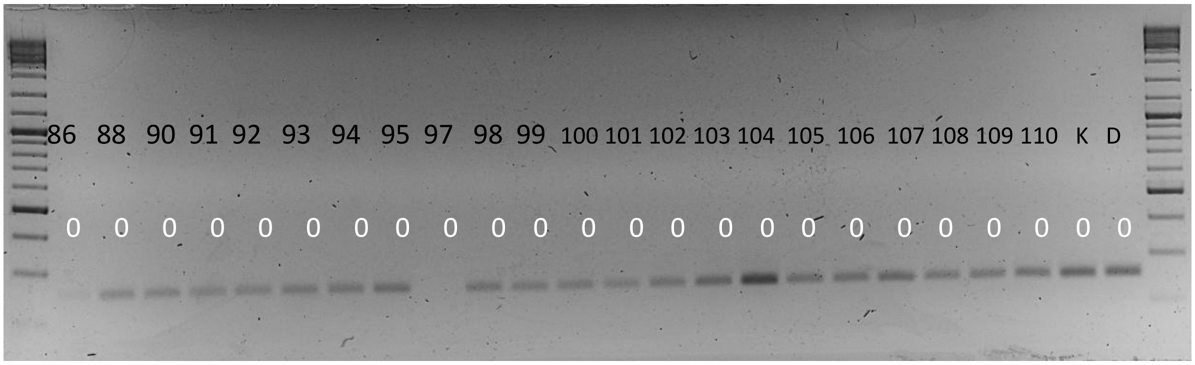

Plate 7

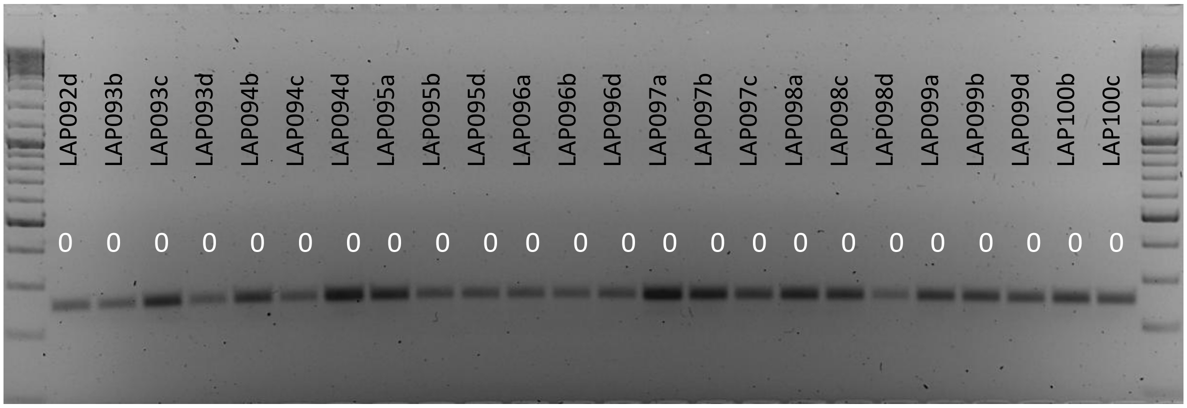
LAP092d LAP093b LAP093c LAP093d LAP094b LAP094c LAP094d LAP095a LAP095b LAP095d LAP096a LAP096b LAP096d LAP097a LAP097b LAP097c LAP098a LAP098c LAP098d LAP099a LAP099b LAP099d LAP100b LAP100c
0 0 0 0 0 0 0 0 0 0 0 0 0 0 0 0 0 0 0 0 0 0 0 0

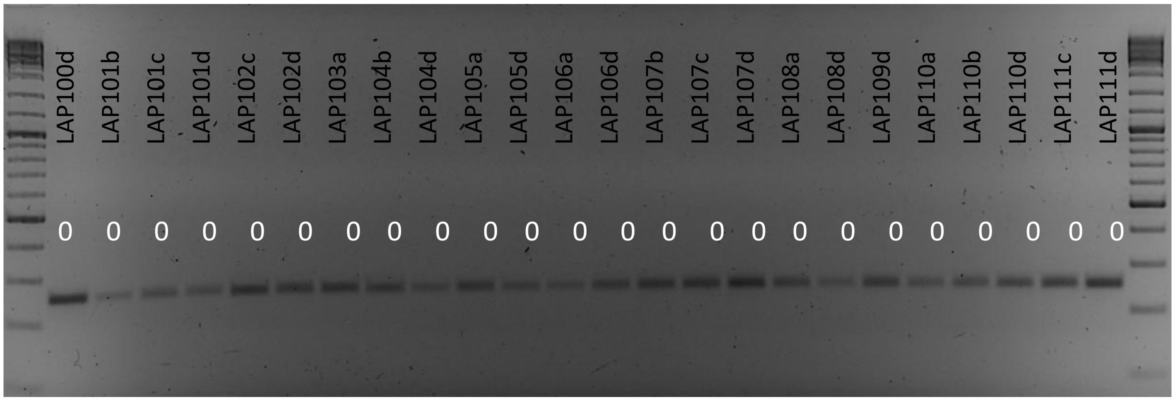
LAP100d LAP101b LAP101c LAP101d LAP102c LAP102d LAP103a LAP104b LAP104d LAP105a LAP105d LAP106a LAP106d LAP107b LAP107c LAP107d LAP108a LAP108d LAP109d LAP110a LAP110b LAP110d LAP111c LAP111d
0 0 0 0 0 0 0 0 0 0 0 0 0 0 0 0 0 0 0 0 0 0 0 0

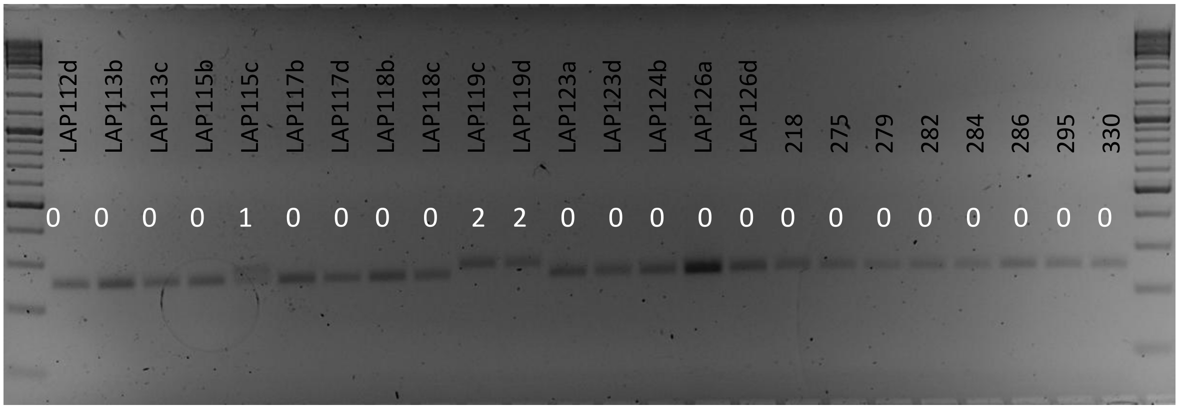
LAP112d LAP113b LAP113c LAP115b LAP115c LAP117b LAP117d LAP118b LAP118c LAP119c LAP119d LAP123a LAP123d LAP124b LAP126a LAP126d 218 275 279 282 284 286 295 330
0 0 0 0 1 0 0 0 0 2 2 0 0 0 0 0 0 0 0 0 0 0 0 0

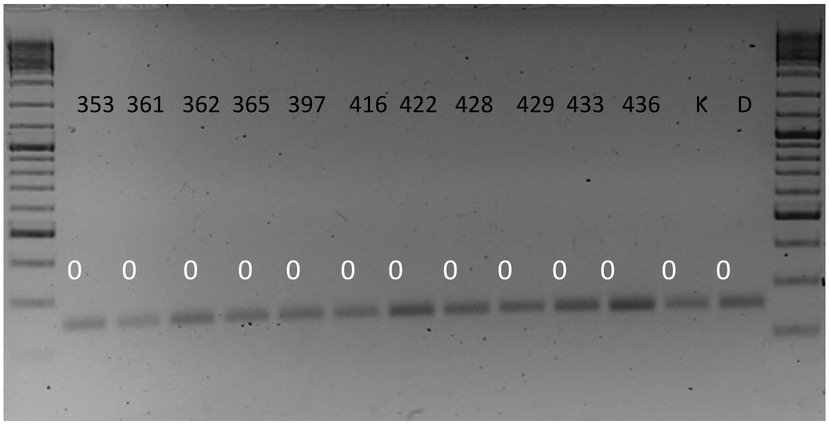
353 361 362 365 397 416 422 428 429 433 436 K D
0 0 0 0 0 0 0 0 0 0 0 0 0

Repeat

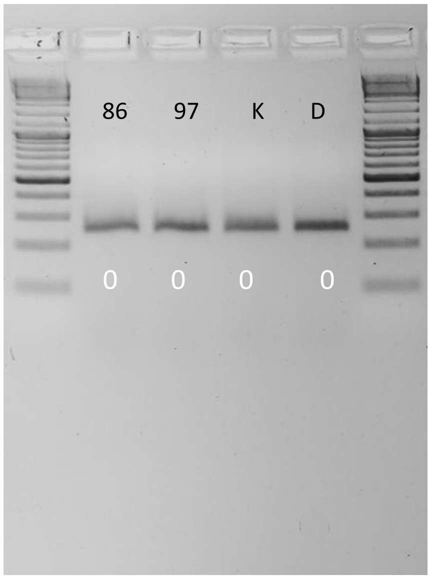
86
97
K
D
0
0
0
0

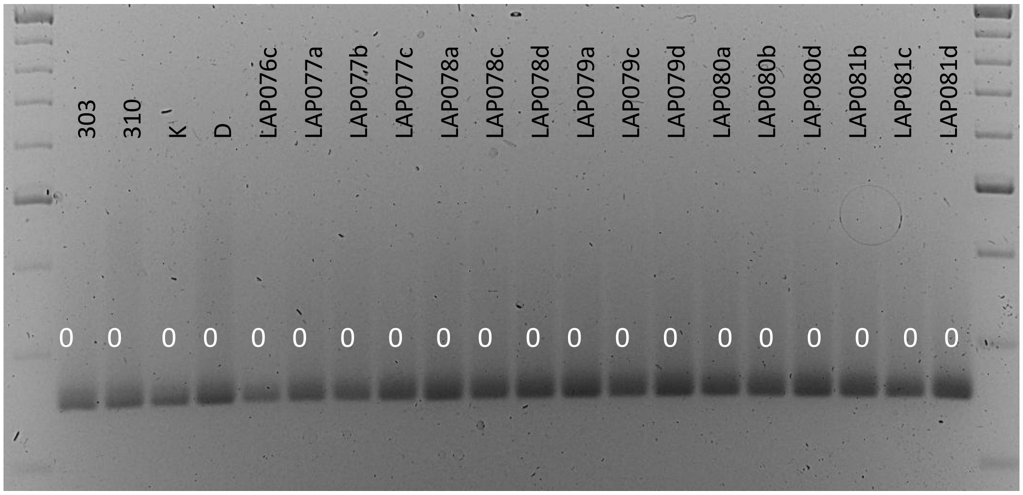
303
310
K
D
LAP076c
LAP077a
LAP077b
LAP077c
LAP078a
LAP078c
LAP078d
LAP079a
LAP079c
LAP079d
LAP080a
LAP080b
LAP080d
LAP081b
LAP081c
LAP081d
0 0 0 0 0 0 0 0 0 0 0 0 0 0 0 0 0 0 0 0

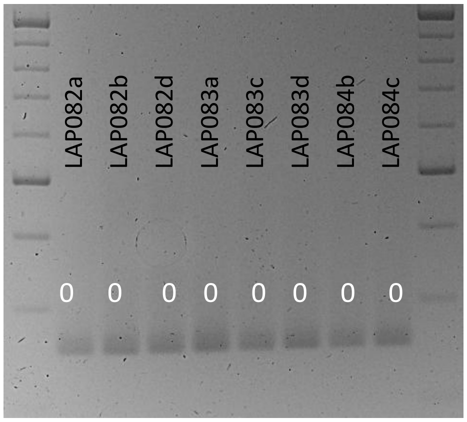
LAP082a
LAP082b
LAP082d
LAP083a
LAP083c
LAP083d
LAP084b
LAP084c
0 0 0 0 0 0 0 0
