# Supplementary material for: Evaluation of the Global White Lupin Collection Reveals Significant Associations Between Homologous FLOWERING LOCUS T Indels and Flowering Time, Providing Validated Markers for Tracking Spring Ecotypes Within a Large Gene Pool
Source: Int J Mol Sci. 2025 Jul 17;26(14):6858. doi: 10.3390/ijms26146858 (PMC12295241; doi:10.3390/ijms26146858)
Supplement: Supplementary file 1 [file ijms-26-06858-s001.zip › Supplementary_Figure_S6_Agarose gel electrophoregrams showing polymorphism of PCR-based markers targeting LalbFTc1 indels.pdf]

Wojciech Bielski, Anna Surma, Michał Książkiewicz, Sandra Rychel-Bielska

Evaluation of the global white lupin collection reveals significant associations between homologous *FLOWERING LOCUS T* indels and flowering time, providing validated markers for tracking spring ecotypes within a large gene pool

International Journal of Molecular Sciences

Supplementary Figure S6. Agarose gel electrophoregrams showing polymorphism of PCR-based markers targeting *LalbFTc1* indels.

PR_30

PRFTC1F1 TGGCATTGTAGATTAGGTCATGT

PRFTc1_R1b TGCAACTCATTCATGAATCCTTGAG

Plate 1

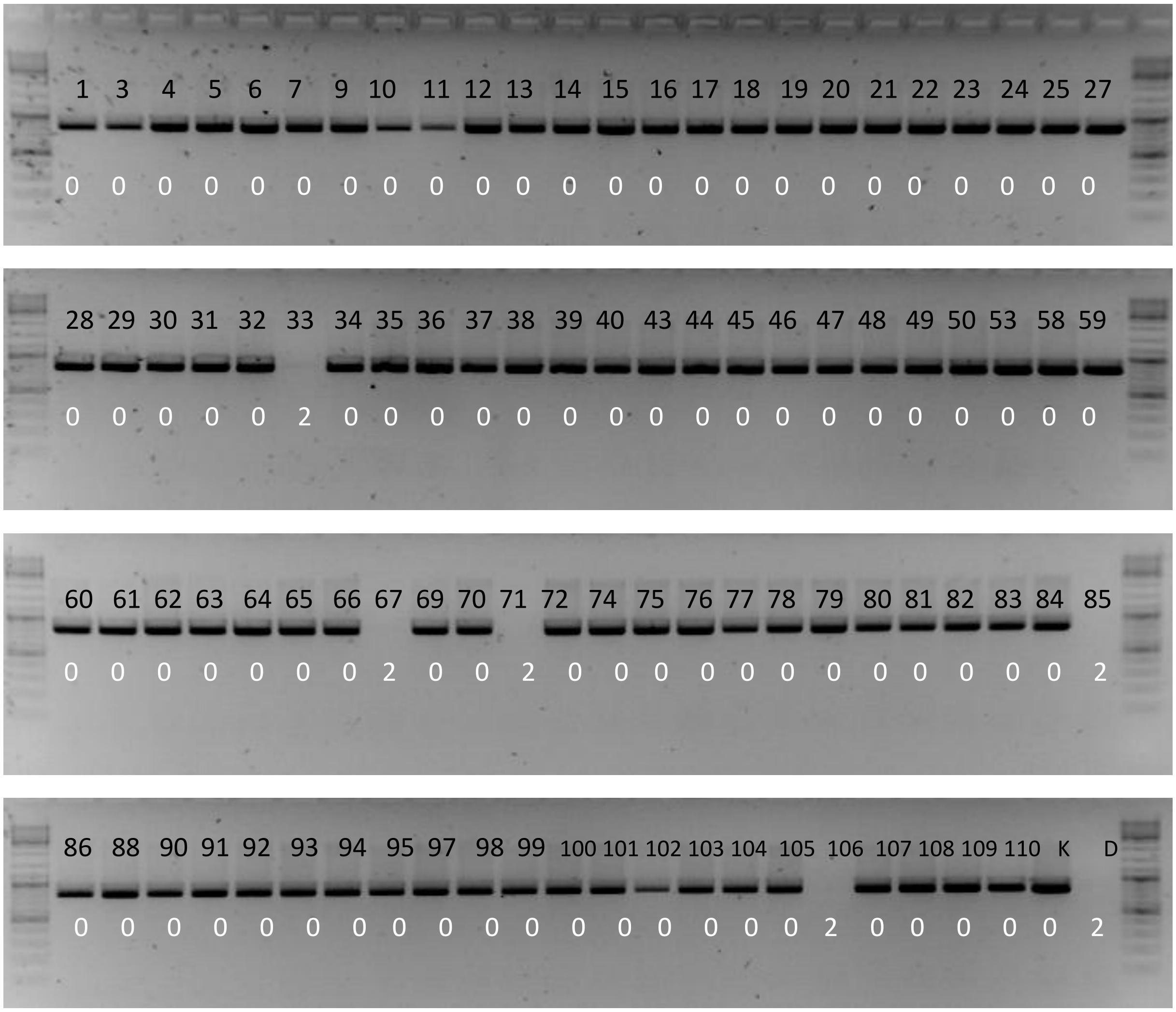

Plate 9

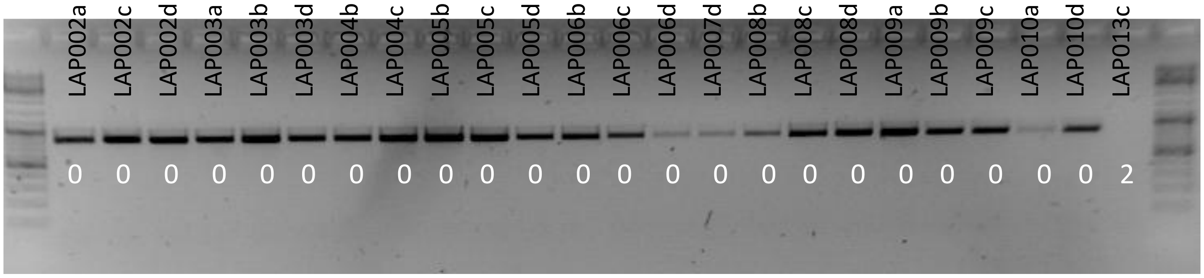
LAP002a LAP002c LAP002d LAP003a LAP003b LAP003d LAP004b LAP004c LAP005b LAP005c LAP005d LAP006b LAP006c LAP006d LAP007d LAP008b LAP008c LAP008d LAP009a LAP009b LAP009c LAP010a LAP010d LAP013c
0 0 0 0 0 0 0 0 0 0 0 0 0 0 0 0 0 0 0 0 0 0 0 2

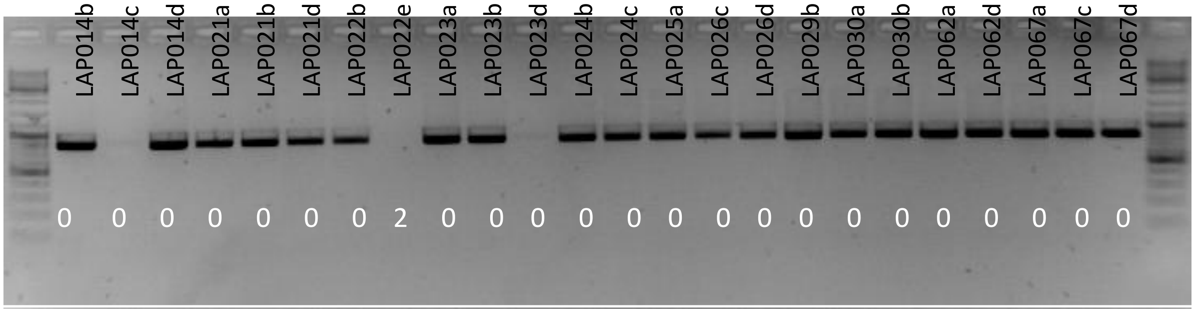
LAP014b LAP014c LAP014d LAP021a LAP021b LAP021d LAP022b LAP022e LAP023a LAP023b LAP023d LAP024b LAP024c LAP025a LAP026c LAP026d LAP029b LAP030a LAP030b LAP062a LAP062d LAP067a LAP067c LAP067d
0 0 0 0 0 0 0 2 0 0 0 0 0 0 0 0 0 0 0 0 0 0 0 0

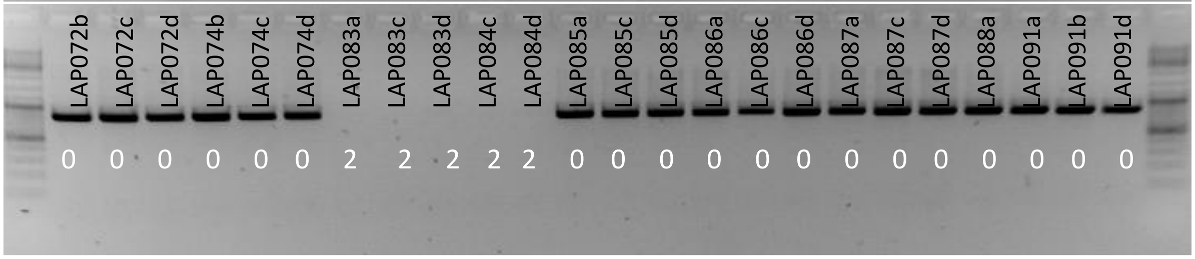
LAP072b LAP072c LAP072d LAP074b LAP074c LAP074d LAP083a LAP083c LAP083d LAP084c LAP084d LAP085a LAP085c LAP085d LAP086a LAP086c LAP086d LAP087a LAP087c LAP087d LAP088a LAP091a LAP091b LAP091d
0 0 0 0 0 0 2 2 2 2 2 0 0 0 0 0 0 0 0 0 0 0 0 0

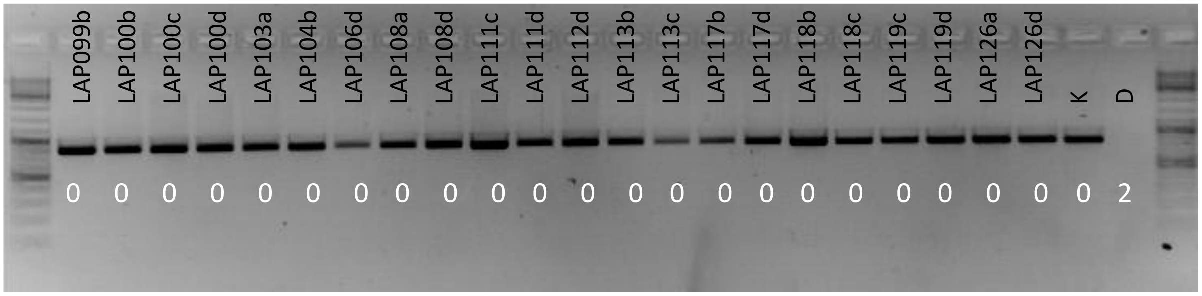
LAP099b LAP100b LAP100c LAP100d LAP103a LAP104b LAP106d LAP108a LAP108d LAP111c LAP111d LAP112d LAP113b LAP113c LAP117b LAP117d LAP118b LAP118c LAP119c LAP119d LAP126a LAP126d K D
0 0 0 0 0 0 0 0 0 0 0 0 0 0 0 0 0 0 0 0 0 0 0 2

PR_31

PRFTc1_F1b AGTCGTCAATTAAGATCTCAGCTCA

PRFTc1_R1c AACACTGAATGCAAGATCAGGTCTA

Plate 1

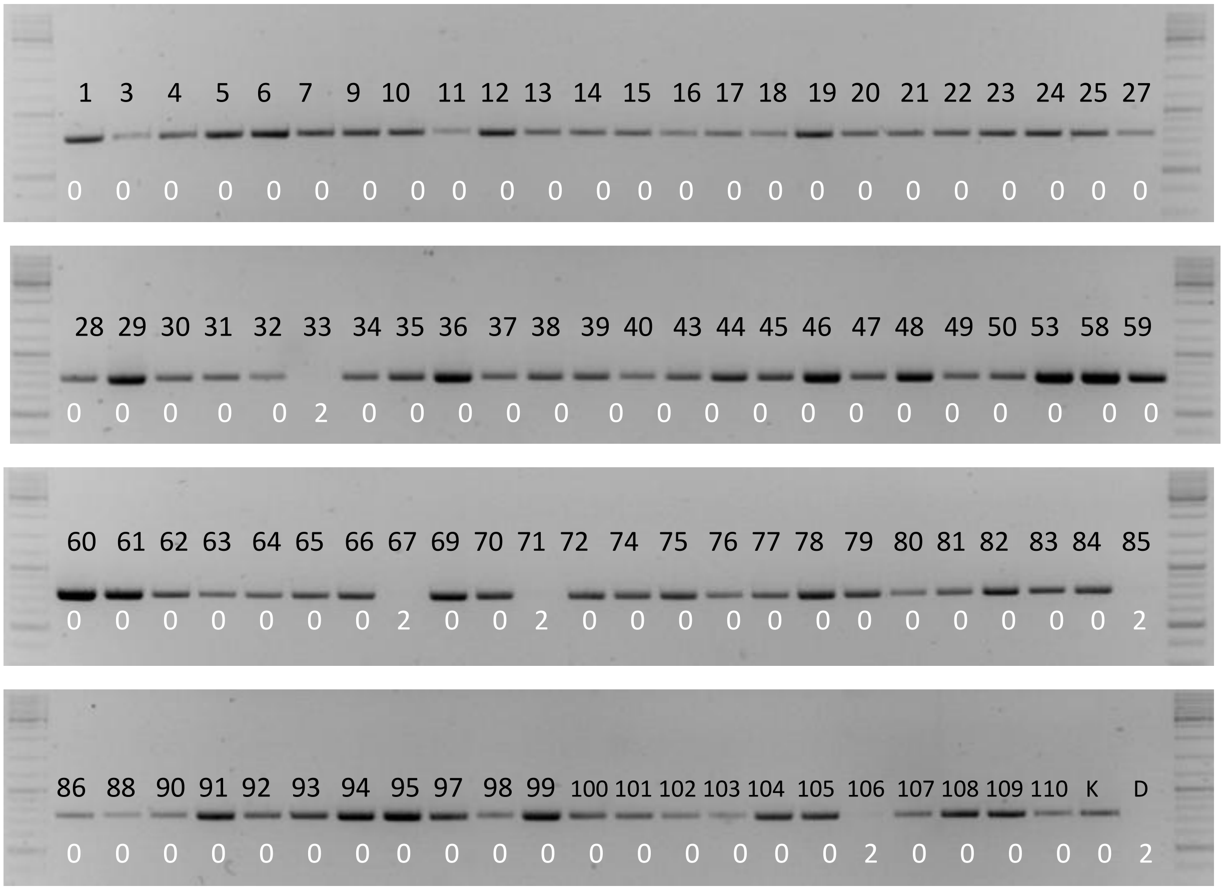

Plate 9

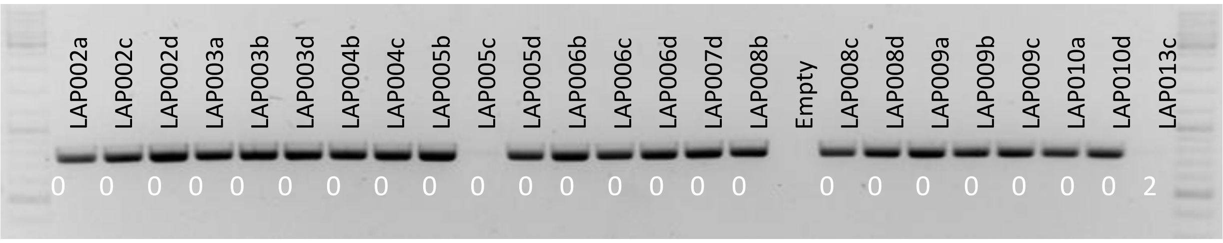
LAP002a LAP002c LAP002d LAP003a LAP003b LAP003d LAP004b LAP004c LAP005b LAP005c LAP005d LAP006b LAP006c LAP006d LAP007d LAP008b Empty LAP008c LAP008d LAP009a LAP009b LAP009c LAP010a LAP010d LAP013c
0 0 0 0 0 0 0 0 0 0 0 0 0 0 0 0 0 0 0 0 0 0 0 2

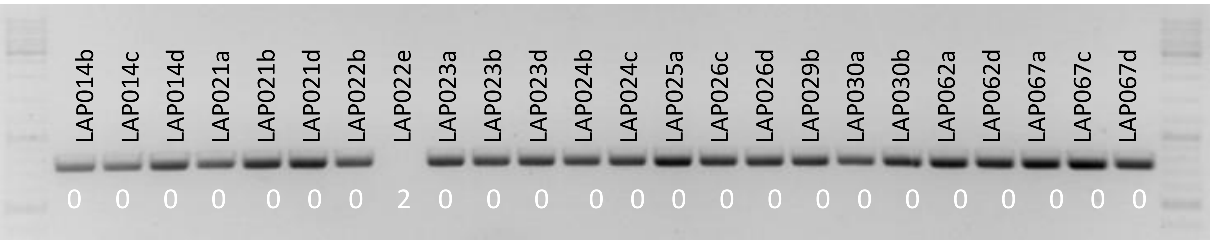
LAP014b LAP014c LAP014d LAP021a LAP021b LAP021d LAP022b LAP022e LAP023a LAP023b LAP023d LAP024b LAP024c LAP025a LAP026c LAP026d LAP029b LAP030a LAP030b LAP062a LAP062d LAP067a LAP067c LAP067d
0 0 0 0 0 0 0 2 0 0 0 0 0 0 0 0 0 0 0 0 0 0 0 0

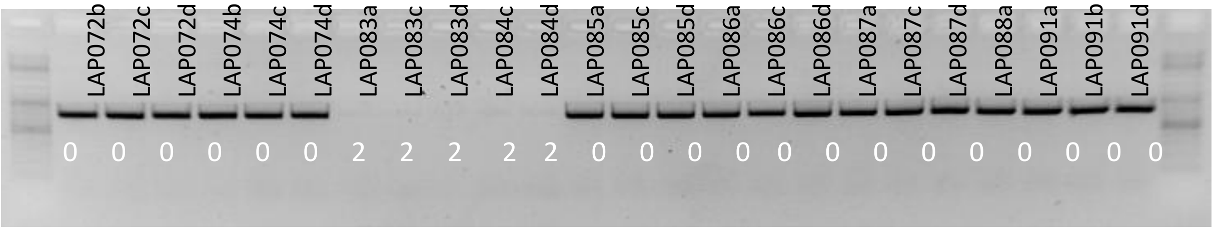
LAP072b LAP072c LAP072d LAP074b LAP074c LAP074d LAP083a LAP083c LAP083d LAP084c LAP084d LAP085a LAP085c LAP085d LAP086a LAP086c LAP086d LAP087a LAP087c LAP087d LAP088a LAP091a LAP091b LAP091d
0 0 0 0 0 0 2 2 2 2 2 0 0 0 0 0 0 0 0 0 0 0 0 0

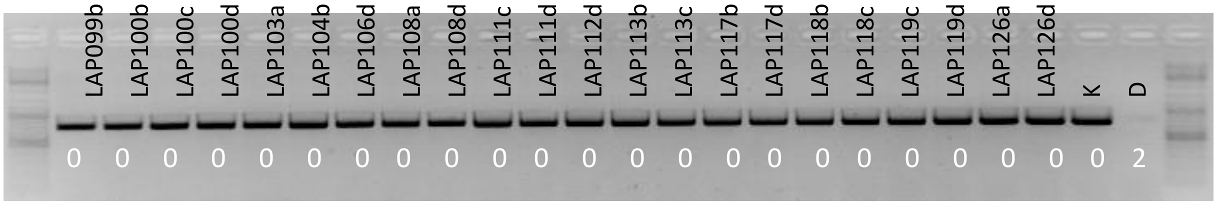
LAP099b LAP100b LAP100c LAP100d LAP103a LAP104b LAP106d LAP108a LAP108d LAP111c LAP111d LAP112d LAP113b LAP113c LAP117b LAP117d LAP118b LAP118c LAP119c LAP119d LAP126a LAP126d K D
0 0 0 0 0 0 0 0 0 0 0 0 0 0 0 0 0 0 0 0 0 0 0 2

PR_33

PRFTc1_F1d AGGTTATGTGGATTGAATCTCTCT

PRFTC1R1 GCACAAAAACAAGCAGAACAA

Plate 1

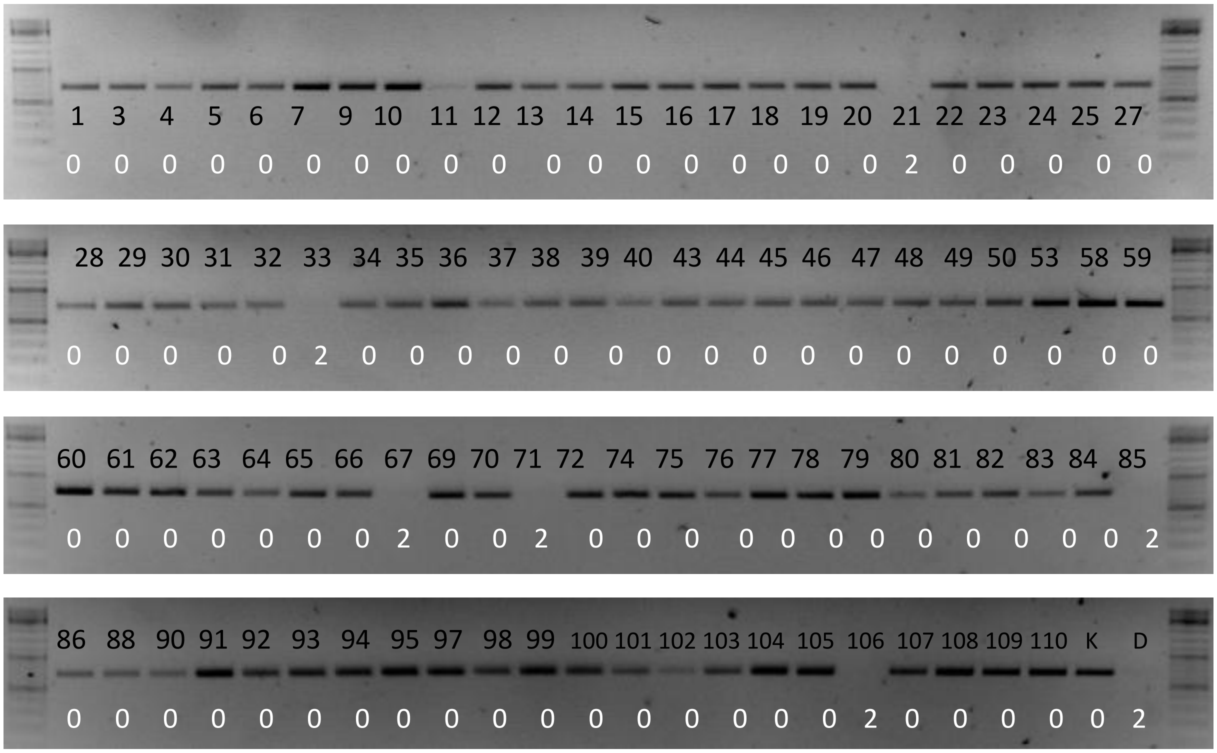

Plate 9

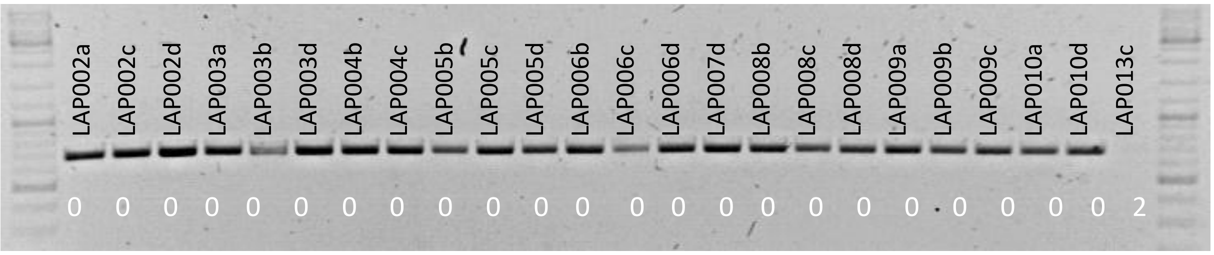
LAP002a
LAP002c
LAP002d
LAP003a
LAP003b
LAP003d
LAP004b
LAP004c
LAP005b
LAP005c
LAP005d
LAP006b
LAP006c
LAP006d
LAP007d
LAP008b
LAP008c
LAP008d
LAP009a
LAP009b
LAP009c
LAP010a
LAP010d
LAP013c
0 0 0 0 0 0 0 0 0 0 0 0 0 0 0 0 0 0 0 0 0 0 0 2

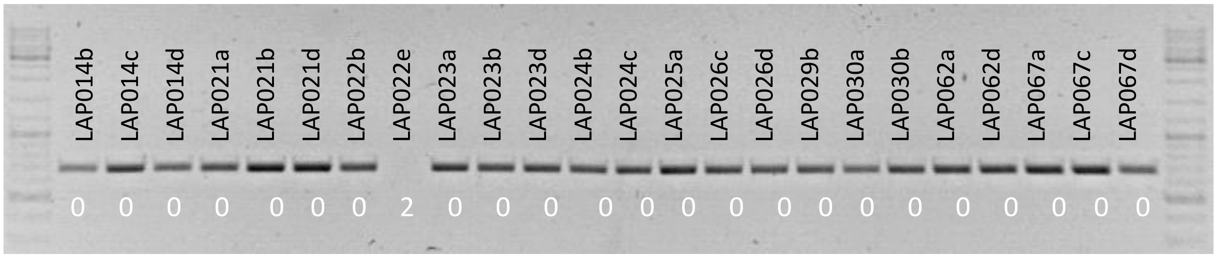
LAP014b
LAP014c
LAP014d
LAP021a
LAP021b
LAP021d
LAP022b
LAP022e
LAP023a
LAP023b
LAP023d
LAP024b
LAP024c
LAP025a
LAP026c
LAP026d
LAP029b
LAP030a
LAP030b
LAP062a
LAP062d
LAP067a
LAP067c
LAP067d
0 0 0 0 0 0 0 2 0 0 0 0 0 0 0 0 0 0 0 0 0 0 0 0

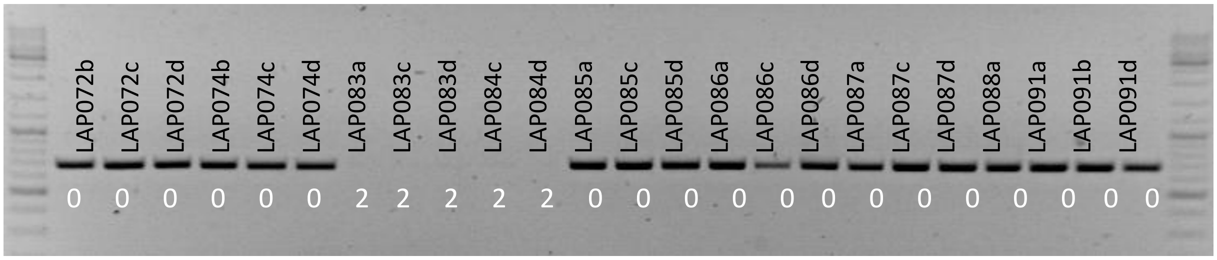
LAP072b
LAP072c
LAP072d
LAP074b
LAP074c
LAP074d
LAP083a
LAP083c
LAP083d
LAP084c
LAP084d
LAP085a
LAP085c
LAP085d
LAP086a
LAP086c
LAP086d
LAP087a
LAP087c
LAP087d
LAP088a
LAP091a
LAP091b
LAP091d
0 0 0 0 0 0 2 2 2 2 2 0 0 0 0 0 0 0 0 0 0 0 0 0

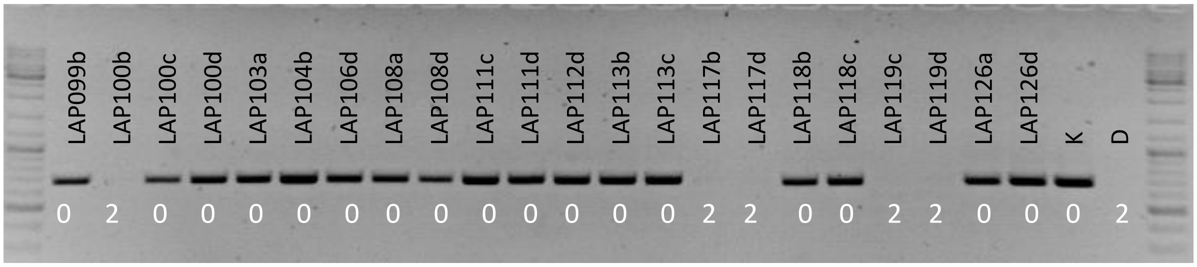
LAP099b
LAP100b
LAP100c
LAP100d
LAP103a
LAP104b
LAP106d
LAP108a
LAP108d
LAP111c
LAP111d
LAP112d
LAP113b
LAP113c
LAP117b
LAP117d
LAP118b
LAP118c
LAP119c
LAP119d
LAP126a
LAP126d
K
D
0 2 0 0 0 0 0 0 0 0 0 0 0 0 2 2 0 0 2 2 0 0 0 2

PR_34

PRFTC1F2 ACAGAATTCAGTTACTTTTCTCTCT

PRFTc1_R2b CACTTATAACCCTTGTGAGTTGCAG

Plate 1

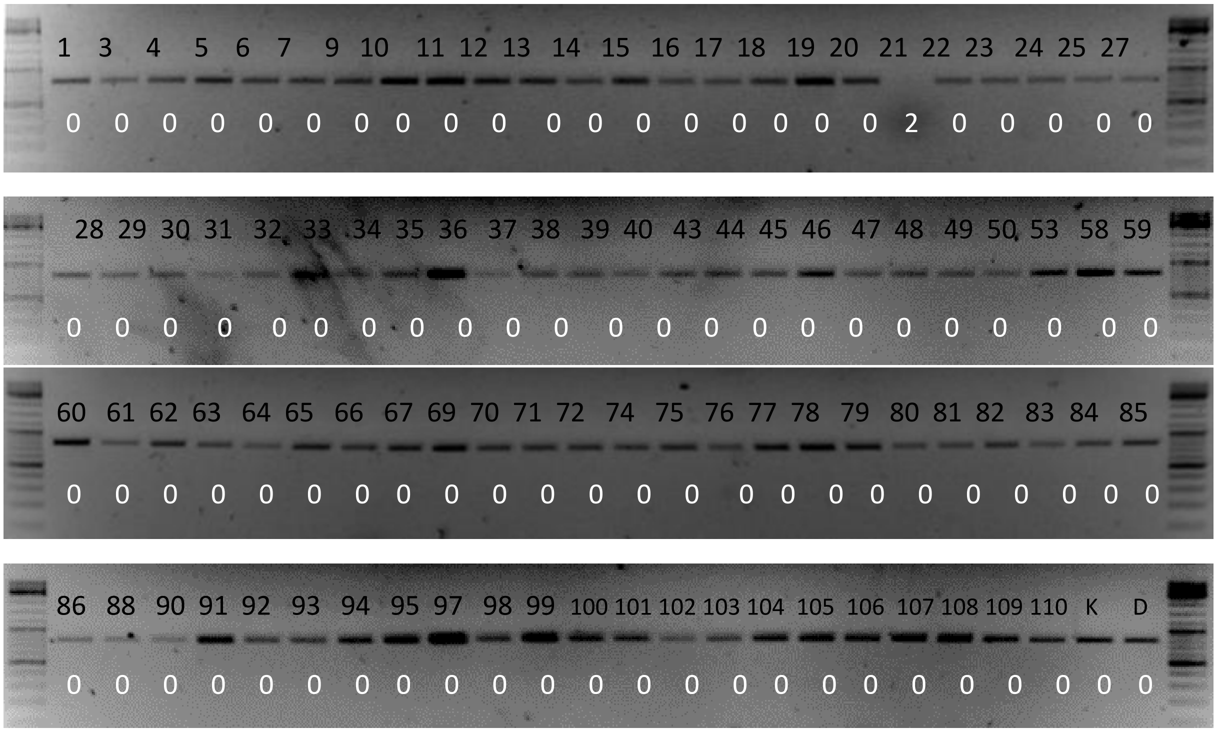

Plate 9

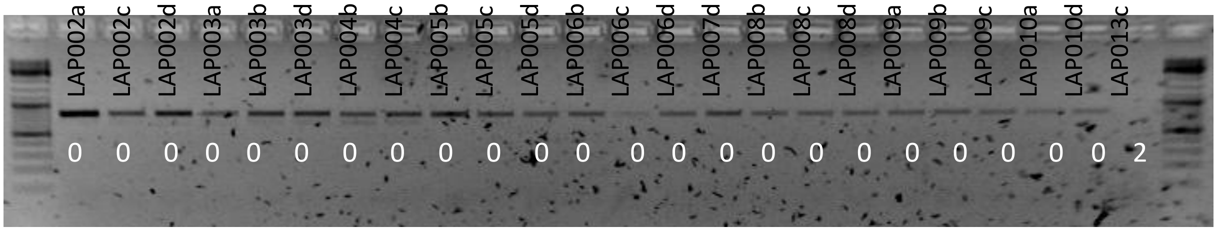
LAP002a
LAP002c
LAP002d
LAP003a
LAP003b
LAP003d
LAP004b
LAP004c
LAP005b
LAP005c
LAP005d
LAP006b
LAP006c
LAP006d
LAP007d
LAP008b
LAP008c
LAP008d
LAP009a
LAP009b
LAP009c
LAP010a
LAP010d
LAP013c
0 0 0 0 0 0 0 0 0 0 0 0 0 0 0 0 0 0 0 0 0 0 0 2

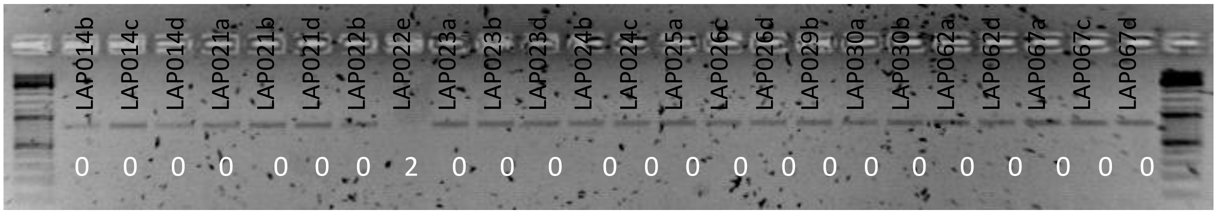
LAP014b
LAP014c
LAP014d
LAP021a
LAP021b
LAP021d
LAP022b
LAP022e
LAP023a
LAP023b
LAP023d
LAP024b
LAP024c
LAP025a
LAP026c
LAP026d
LAP029b
LAP030a
LAP030b
LAP062a
LAP062d
LAP067a
LAP067c
LAP067d
0 0 0 0 0 0 0 2 0 0 0 0 0 0 0 0 0 0 0 0 0 0 0 0

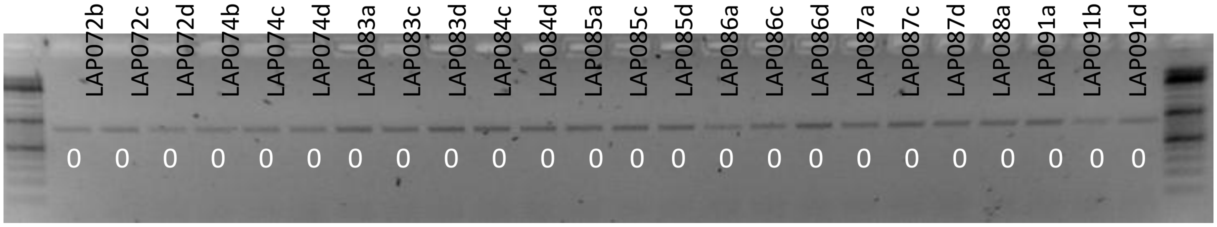
LAP072b
LAP072c
LAP072d
LAP074b
LAP074c
LAP074d
LAP083a
LAP083c
LAP083d
LAP084c
LAP084d
LAP085a
LAP085c
LAP085d
LAP086a
LAP086c
LAP086d
LAP087a
LAP087c
LAP087d
LAP088a
LAP091a
LAP091b
LAP091d
0 0 0 0 0 0 0 0 0 0 0 0 0 0 0 0 0 0 0 0 0 0 0 0

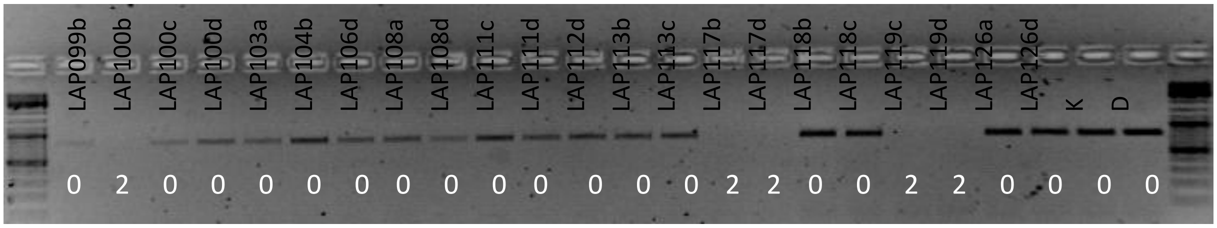
LAP099b
LAP100b
LAP100c
LAP100d
LAP103a
LAP104b
LAP106d
LAP108a
LAP108d
LAP111c
LAP111d
LAP112d
LAP113b
LAP113c
LAP117b
LAP117d
LAP118b
LAP118c
LAP119c
LAP119d
LAP126a
LAP126d
K
D
0 2 0 0 0 0 0 0 0 0 0 0 0 0 2 2 0 0 2 2 0 0 0 0

PR_35a

PRFTc1_F2b ACAAATTGGAACTCAGATTAGCAGA

PRFTC1R2 AGTAACCAGTGACCATGTGCC

Plate 1

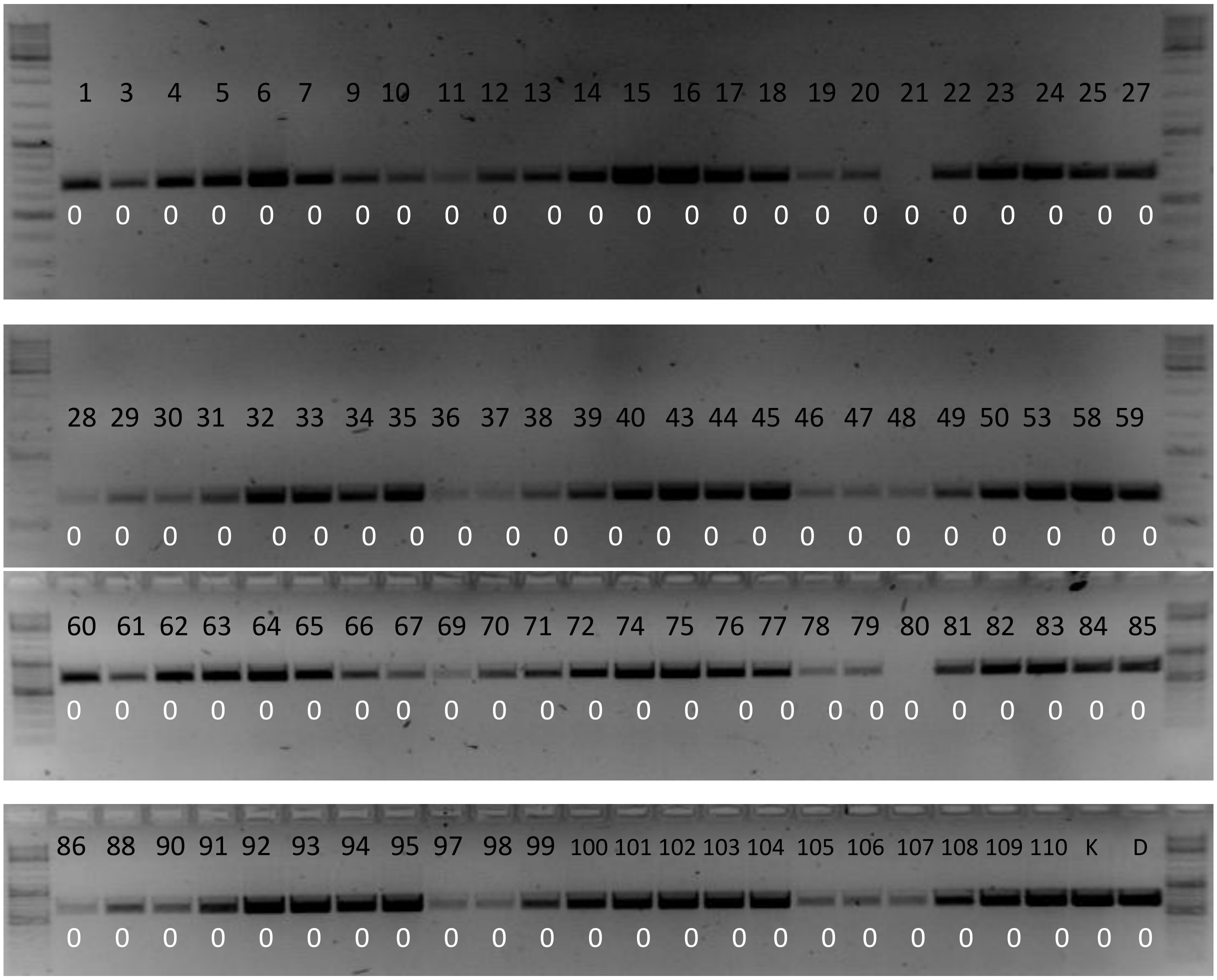

Plate 9

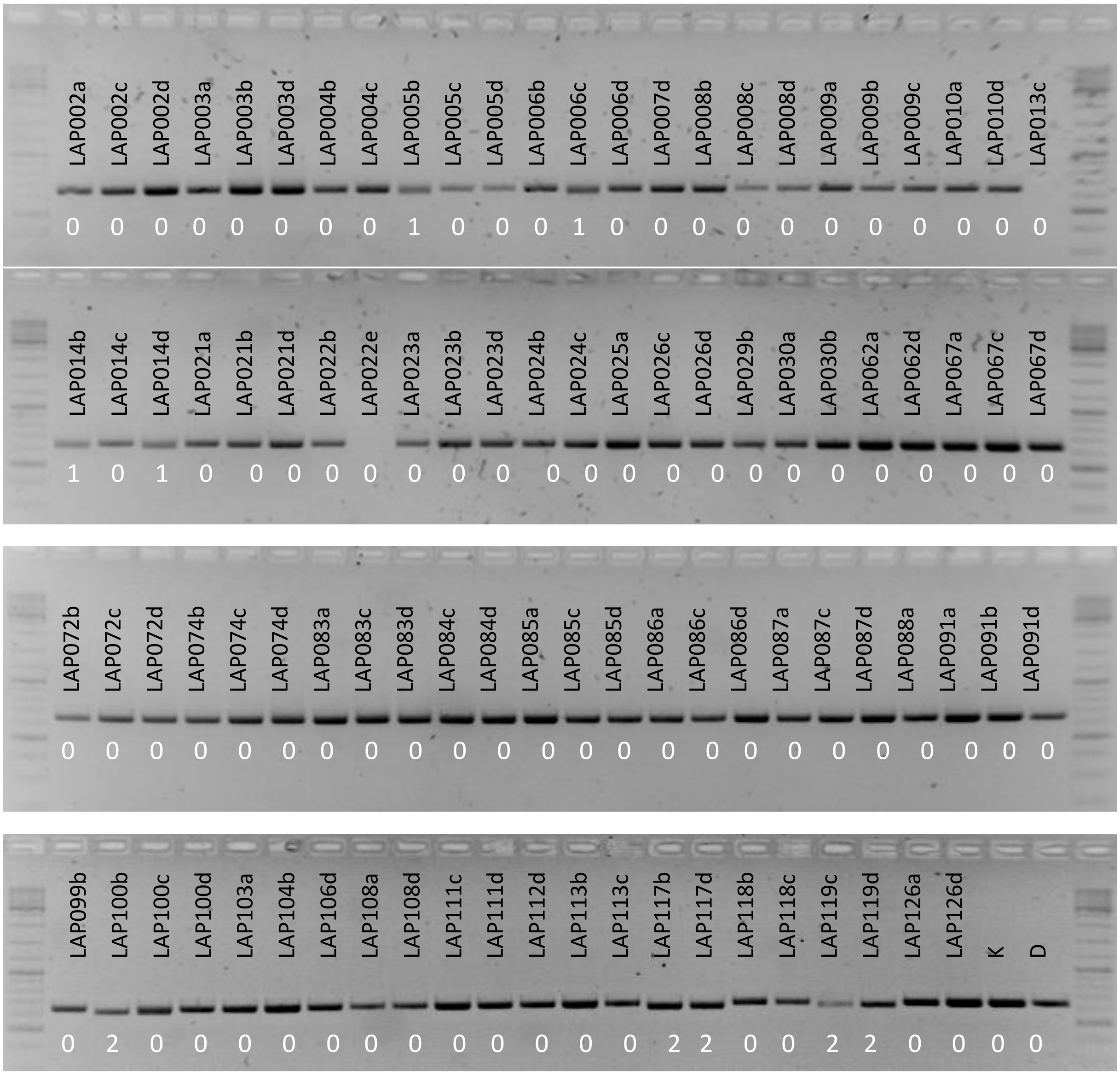
LAP002a LAP002c LAP002d LAP003a LAP003b LAP003d LAP004b LAP004c LAP005b LAP005c LAP005d LAP006b LAP006c LAP006d LAP007d LAP008b LAP008c LAP008d LAP009a LAP009b LAP009c LAP010a LAP010d LAP013c
0 0 0 0 0 0 0 0 1 0 0 0 1 0 0 0 0 0 0 0 0 0 0 0
LAP014b LAP014c LAP014d LAP021a LAP021b LAP021d LAP022b LAP022e LAP023a LAP023b LAP023d LAP024b LAP024c LAP025a LAP026c LAP026d LAP029b LAP030a LAP030b LAP062a LAP062d LAP067a LAP067c LAP067d
1 0 1 0 0 0 0 0 0 0 0 0 0 0 0 0 0 0 0 0 0 0 0 0
LAP072b LAP072c LAP072d LAP074b LAP074c LAP074d LAP083a LAP083c LAP083d LAP084c LAP084d LAP085a LAP085c LAP085d LAP086a LAP086c LAP086d LAP087a LAP087c LAP087d LAP088a LAP091a LAP091b LAP091d
0 0 0 0 0 0 0 0 0 0 0 0 0 0 0 0 0 0 0 0 0 0 0 0
LAP099b LAP100b LAP100c LAP100d LAP103a LAP104b LAP106d LAP108a LAP108d LAP111c LAP111d LAP112d LAP113b LAP113c LAP117b LAP117d LAP118b LAP118c LAP119c LAP119d LAP126a LAP126d K D
0 2 0 0 0 0 0 0 0 0 0 0 0 0 2 2 0 0 2 2 0 0 0 0

PR_35b

PRFTc1_F2b ACAAATTGGAACTCAGATTAGCAGA

PRFTC1R2 AGTAACCAGTGACCATGTGCC

Plate 1

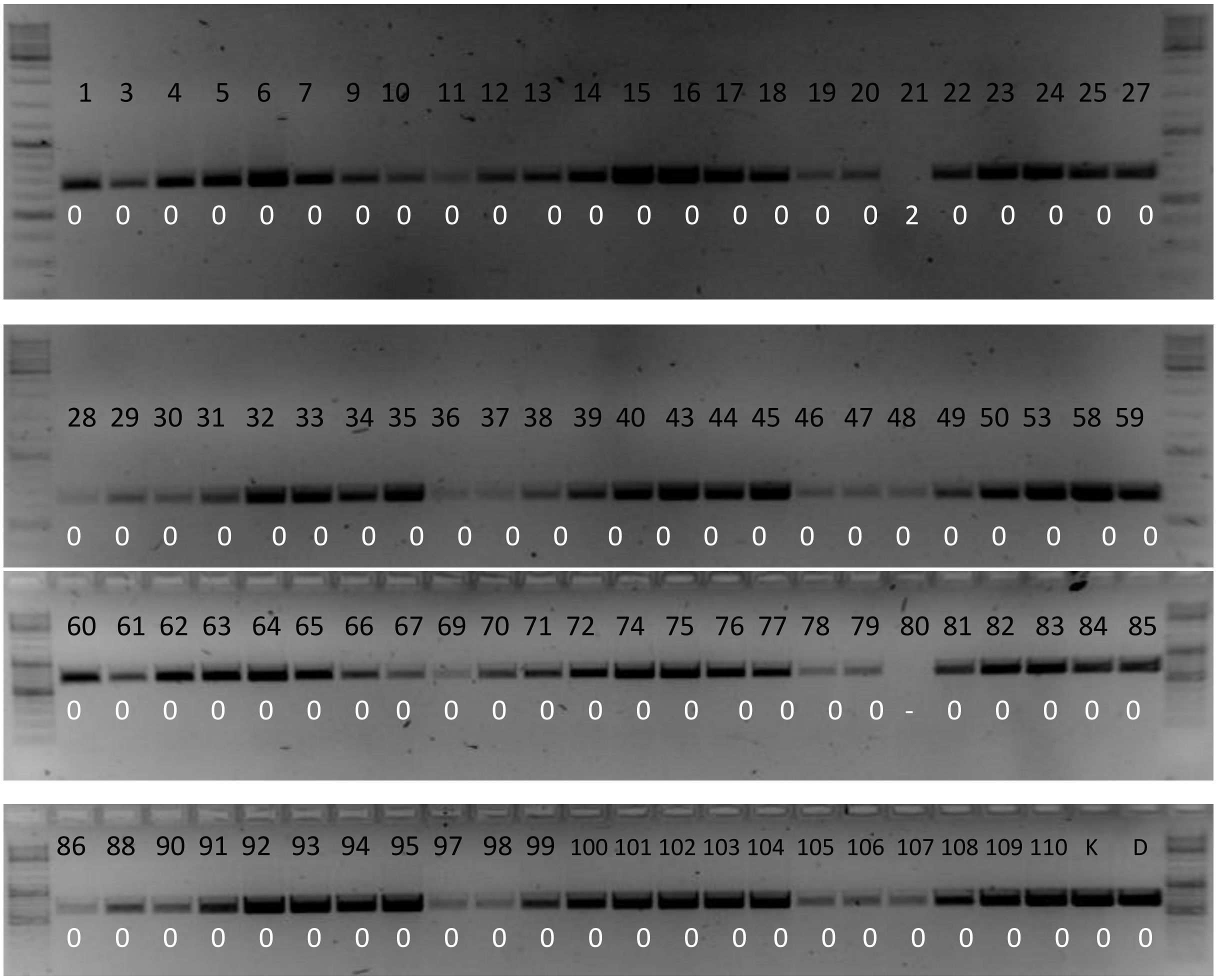

Plate 9

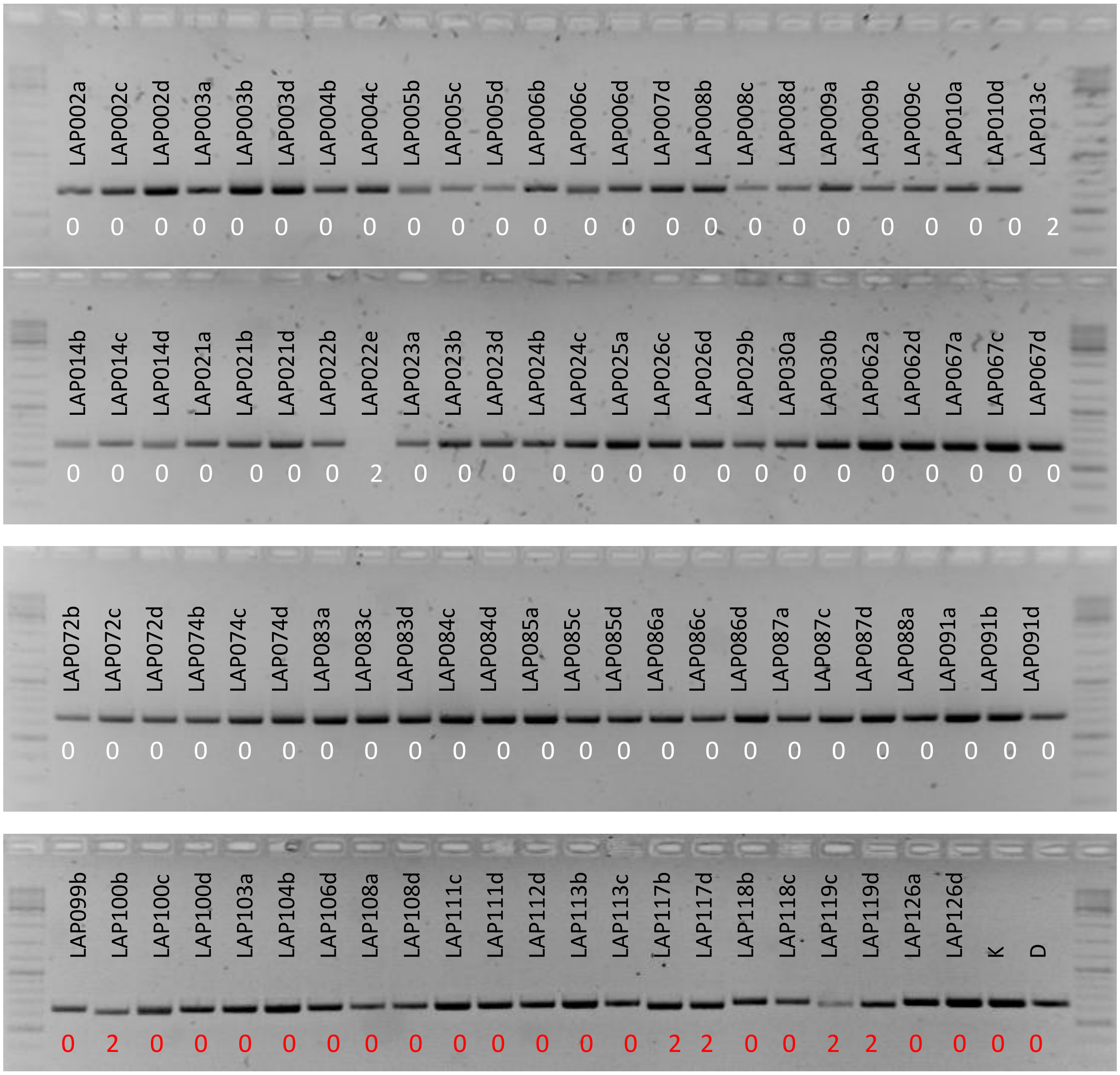
LAP002a LAP002c LAP002d LAP003a LAP003b LAP003d LAP004b LAP004c LAP005b LAP005c LAP005d LAP006b LAP006c LAP006d LAP007d LAP008b LAP008c LAP008d LAP009a LAP009b LAP009c LAP010a LAP010d LAP013c
0 0 0 0 0 0 0 0 0 0 0 0 0 0 0 0 0 0 0 0 0 0 0 2
LAP014b LAP014c LAP014d LAP021a LAP021b LAP021d LAP022b LAP022e LAP023a LAP023b LAP023d LAP024b LAP024c LAP025a LAP026c LAP026d LAP029b LAP030a LAP030b LAP062a LAP062d LAP067a LAP067c LAP067d
0 0 0 0 0 0 0 2 0 0 0 0 0 0 0 0 0 0 0 0 0 0 0 0
LAP072b LAP072c LAP072d LAP074b LAP074c LAP074d LAP083a LAP083c LAP083d LAP084c LAP084d LAP085a LAP085c LAP085d LAP086a LAP086c LAP086d LAP087a LAP087c LAP087d LAP088a LAP091a LAP091b LAP091d
0 0 0 0 0 0 0 0 0 0 0 0 0 0 0 0 0 0 0 0 0 0 0 0
LAP099b LAP100b LAP100c LAP100d LAP103a LAP104b LAP106d LAP108a LAP108d LAP111c LAP111d LAP112d LAP113b LAP113c LAP117b LAP117d LAP118b LAP118c LAP119c LAP119d LAP126a LAP126d K D
0 2 0 0 0 0 0 0 0 0 0 0 0 0 2 2 0 0 2 2 0 0 0 0

PR_36a

PRFTC1F3 TCTTCTCTACCCTCTGCTCCT

PRFTc1_R3b AAGTTTGTAGGTGAGAGTGATGAGT

Plate 1

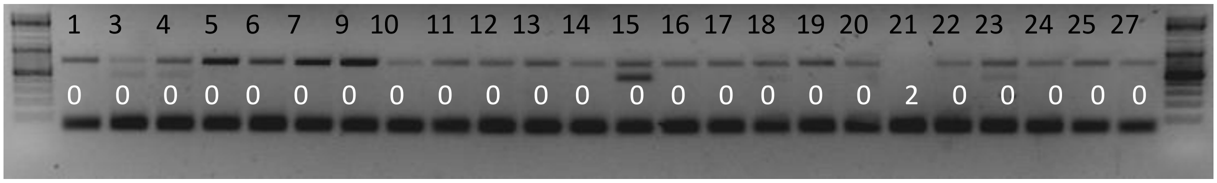

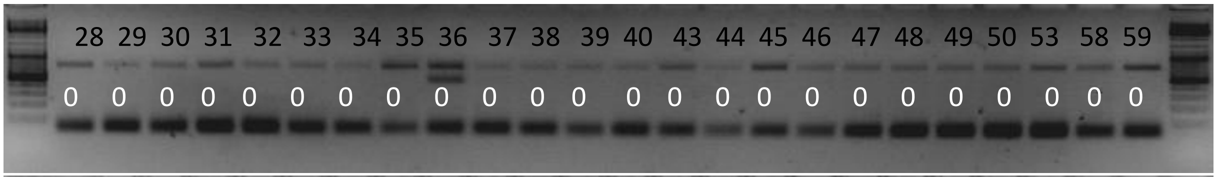

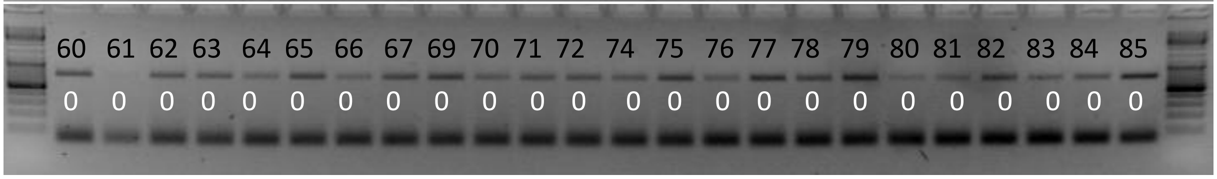

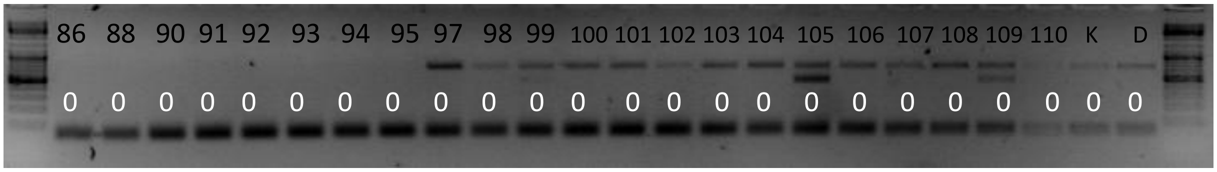

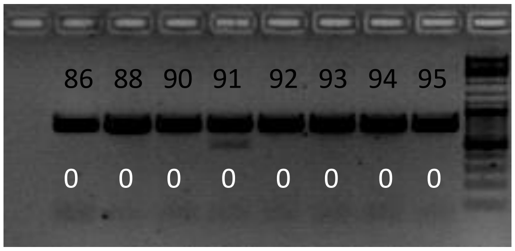

Plate 9

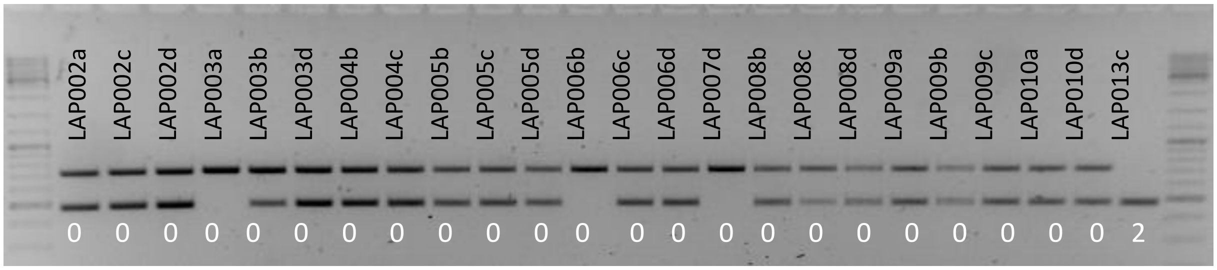
LAP002a LAP002c LAP002d LAP003a LAP003b LAP003d LAP004b LAP004c LAP005b LAP005c LAP005d LAP006b LAP006c LAP006d LAP007d LAP008b LAP008c LAP008d LAP009a LAP009b LAP009c LAP010a LAP010d LAP013c
0 0 0 0 0 0 0 0 0 0 0 0 0 0 0 0 0 0 0 0 0 0 0 2

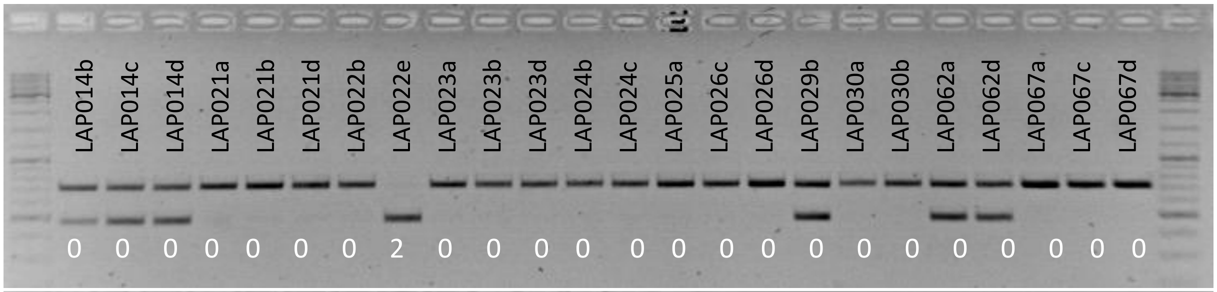
LAP014b LAP014c LAP014d LAP021a LAP021b LAP021d LAP022b LAP022e LAP023a LAP023b LAP023d LAP024b LAP024c LAP025a LAP026c LAP026d LAP029b LAP030a LAP030b LAP062a LAP062d LAP067a LAP067c LAP067d
0 0 0 0 0 0 0 2 0 0 0 0 0 0 0 0 0 0 0 0 0 0 0 0

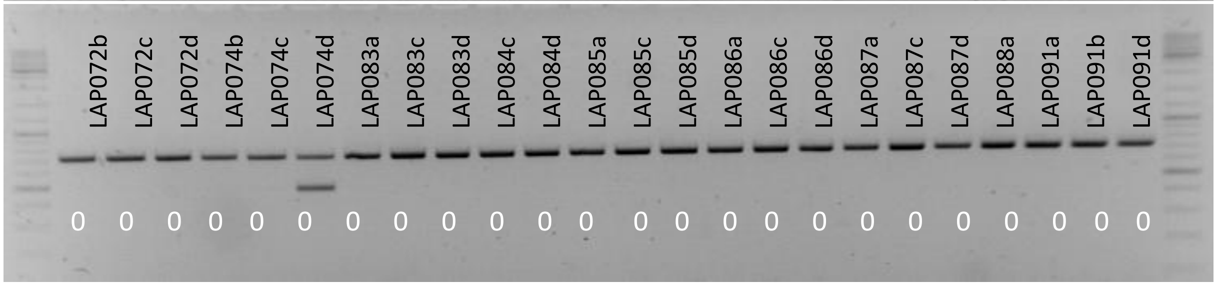
LAP072b LAP072c LAP072d LAP074b LAP074c LAP074d LAP083a LAP083c LAP083d LAP084c LAP084d LAP085a LAP085c LAP085d LAP086a LAP086c LAP086d LAP087a LAP087c LAP087d LAP088a LAP091a LAP091b LAP091d
0 0 0 0 0 0 0 0 0 0 0 0 0 0 0 0 0 0 0 0 0 0 0 0

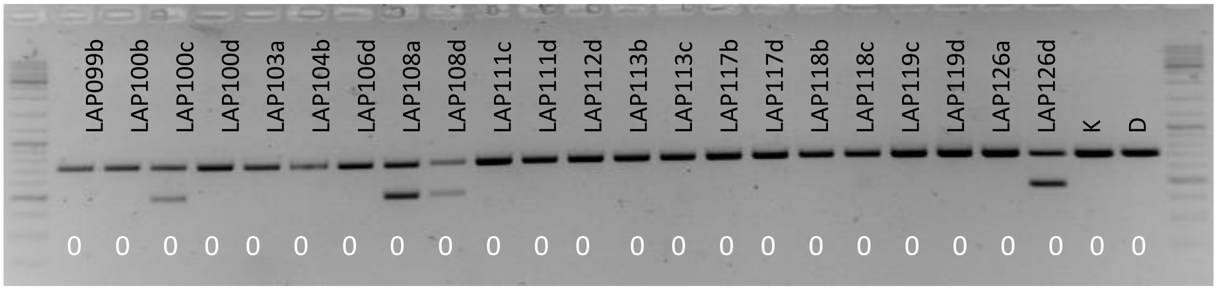
LAP099b LAP100b LAP100c LAP100d LAP103a LAP104b LAP106d LAP108a LAP108d LAP111c LAP111d LAP112d LAP113b LAP113c LAP117b LAP117d LAP118b LAP118c LAP119c LAP119d LAP126a LAP126d K D
0 0 0 0 0 0 0 0 0 0 0 0 0 0 0 0 0 0 0 0 0 0 0 0

PR_36b

PRFTC1F3 TCTTCTCTACCCTCTGCTCCT

PRFTc1_R3b AAGTTTGTAGGTGAGAGTGATGAGT

Plate 1

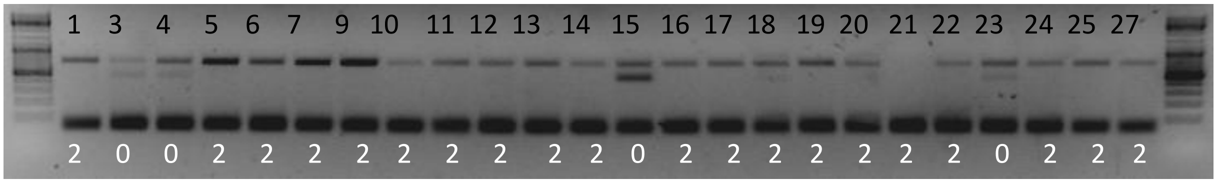

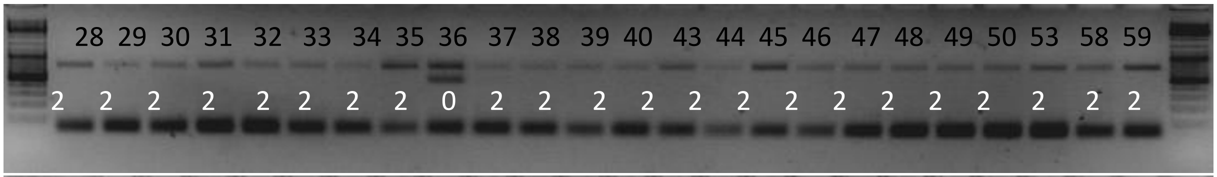

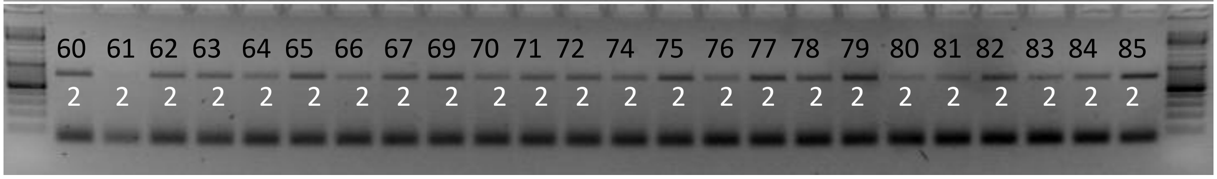

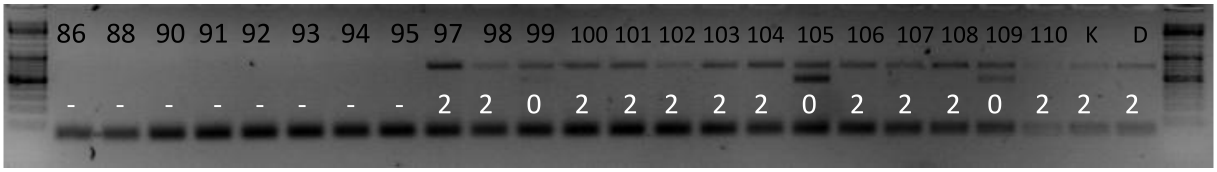

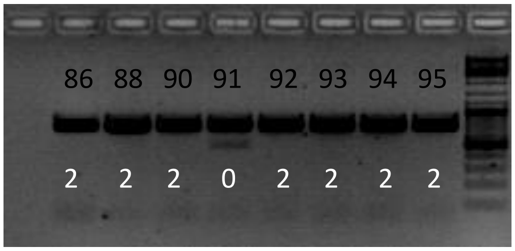

Plate 9

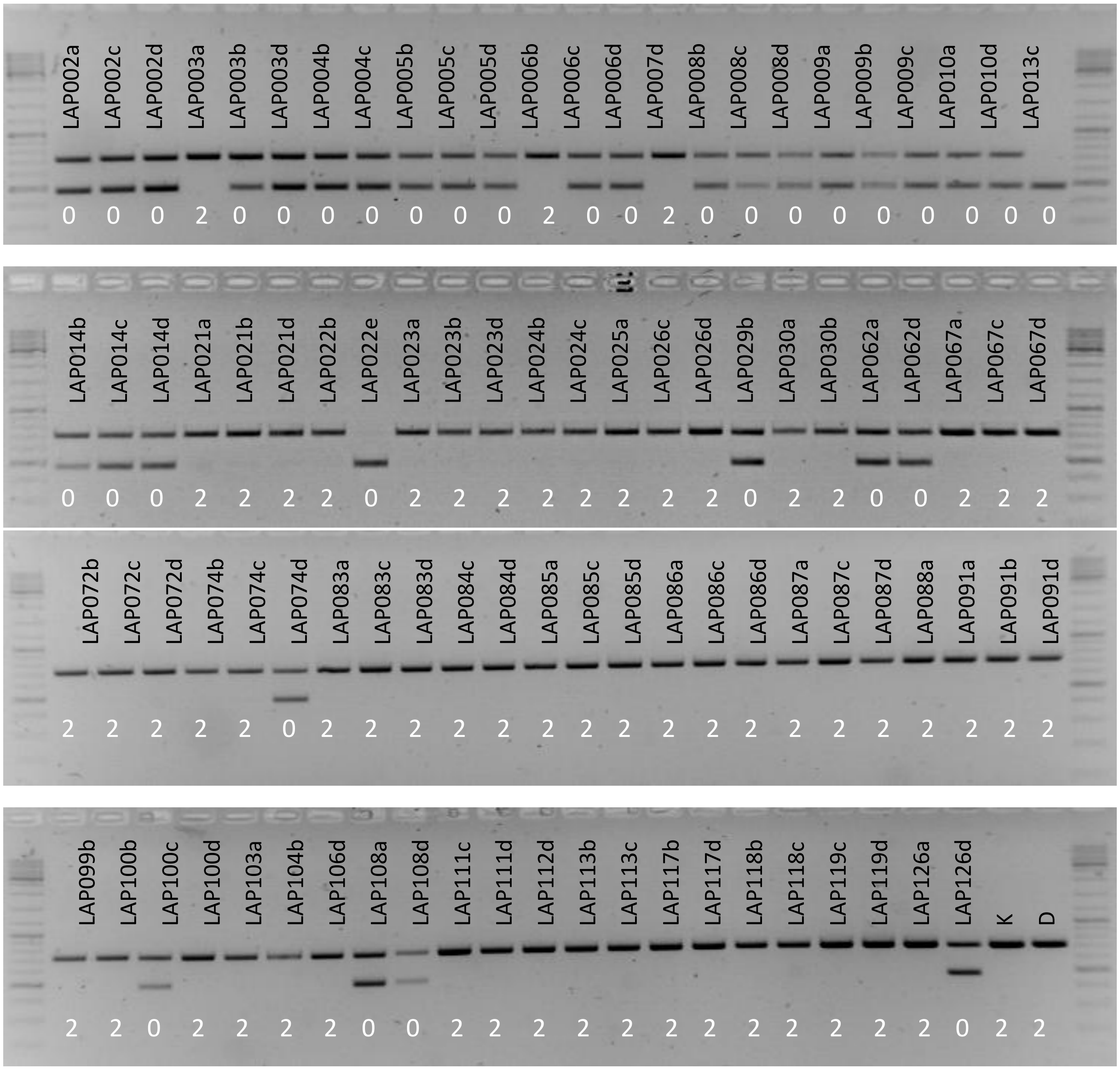
LAP002a LAP002c LAP002d LAP003a LAP003b LAP003d LAP004b LAP004c LAP005b LAP005c LAP005d LAP006b LAP006c LAP006d LAP007d LAP008b LAP008c LAP008d LAP009a LAP009b LAP009c LAP010a LAP010d LAP013c
0 0 0 2 0 0 0 0 0 0 0 2 0 0 2 0 0 0 0 0 0 0 0 0
LAP014b LAP014c LAP014d LAP021a LAP021b LAP021d LAP022b LAP022e LAP023a LAP023b LAP023d LAP024b LAP024c LAP025a LAP026c LAP026d LAP029b LAP030a LAP030b LAP062a LAP062d LAP067a LAP067c LAP067d
0 0 0 2 2 2 2 0 2 2 2 2 2 2 2 2 0 2 2 0 0 2 2 2
LAP072b LAP072c LAP072d LAP074b LAP074c LAP074d LAP083a LAP083c LAP083d LAP084c LAP084d LAP085a LAP085c LAP085d LAP086a LAP086c LAP086d LAP087a LAP087c LAP087d LAP088a LAP091a LAP091b LAP091d
2 2 2 2 2 0 2 2 2 2 2 2 2 2 2 2 2 2 2 2 2 2 2 2
LAP099b LAP100b LAP100c LAP100d LAP103a LAP104b LAP106d LAP108a LAP108d LAP111c LAP111d LAP112d LAP113b LAP113c LAP117b LAP117d LAP118b LAP118c LAP119c LAP119d LAP126a LAP126d K D
2 2 0 2 2 2 2 0 0 2 2 2 2 2 2 2 2 2 2 2 2 0 2 2

PR_37

PRFTc1_F3b CCTAGAAATGGTTTGAAAGGCGAAA

PRFTc1_R3c AGAATCCAACTCCAAGCTCCAATAT

Plate 1

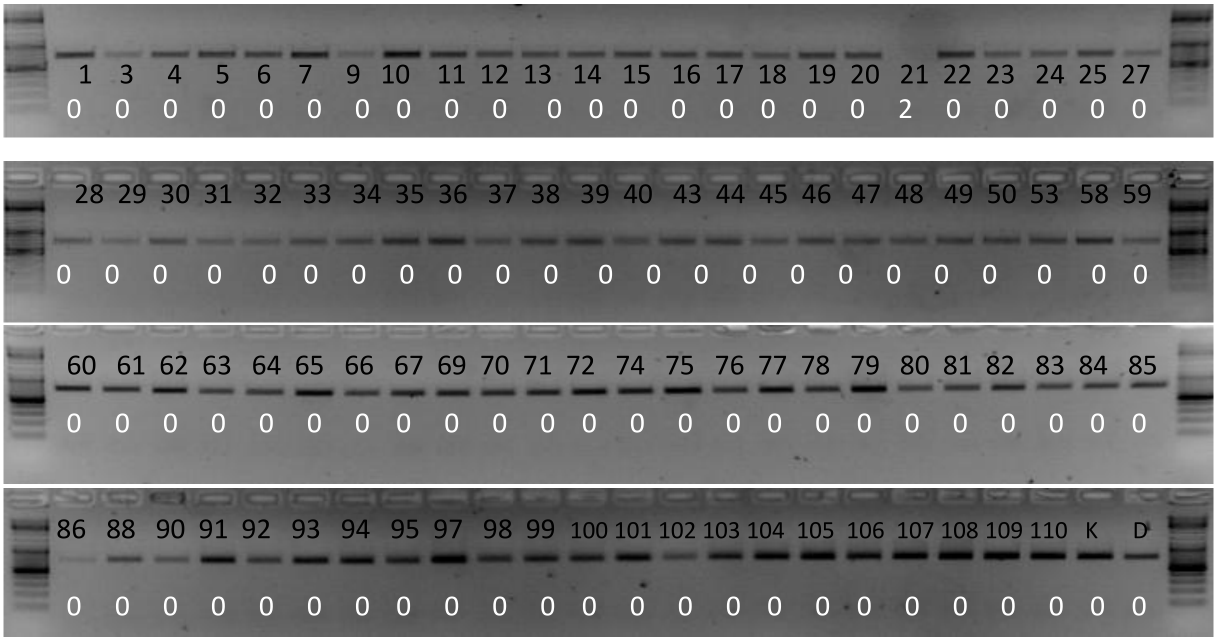

Plate 9

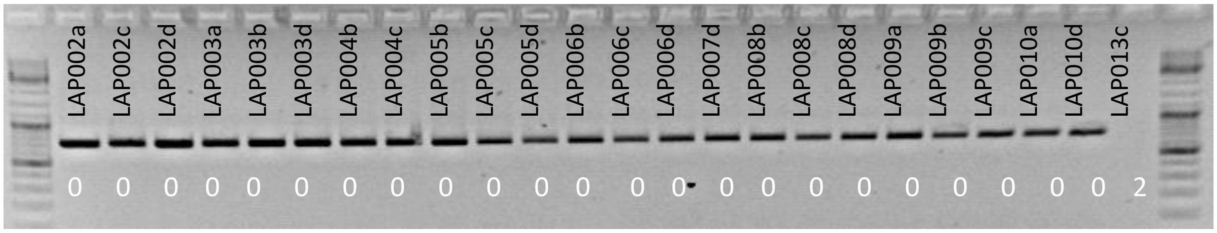
LAP002a
LAP002c
LAP002d
LAP003a
LAP003b
LAP003d
LAP004b
LAP004c
LAP005b
LAP005c
LAP005d
LAP006b
LAP006c
LAP006d
LAP007d
LAP008b
LAP008c
LAP008d
LAP009a
LAP009b
LAP009c
LAP010a
LAP010d
LAP013c
0 0 0 0 0 0 0 0 0 0 0 0 0 0 0 0 0 0 0 0 0 0 0 2

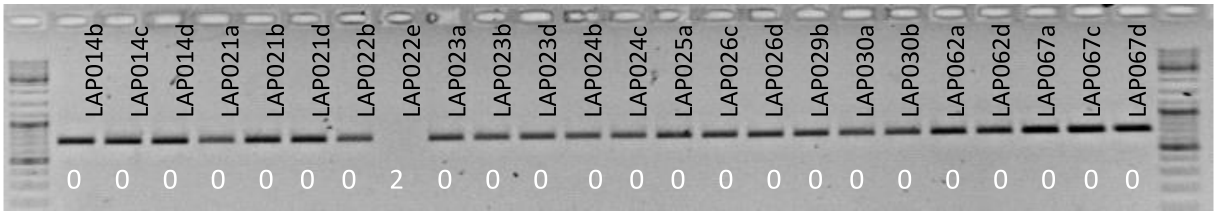
LAP014b
LAP014c
LAP014d
LAP021a
LAP021b
LAP021d
LAP022b
LAP022e
LAP023a
LAP023b
LAP023d
LAP024b
LAP024c
LAP025a
LAP026c
LAP026d
LAP029b
LAP030a
LAP030b
LAP062a
LAP062d
LAP067a
LAP067c
LAP067d
0 0 0 0 0 0 0 2 0 0 0 0 0 0 0 0 0 0 0 0 0 0 0 0

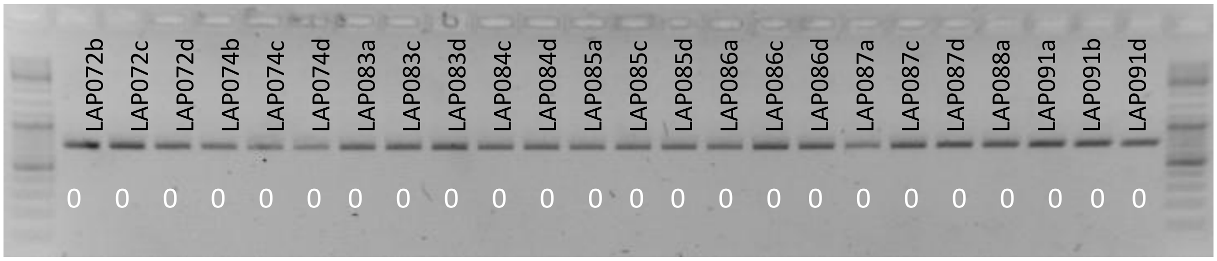
LAP072b
LAP072c
LAP072d
LAP074b
LAP074c
LAP074d
LAP083a
LAP083c
LAP083d
LAP084c
LAP084d
LAP085a
LAP085c
LAP085d
LAP086a
LAP086c
LAP086d
LAP087a
LAP087c
LAP087d
LAP088a
LAP091a
LAP091b
LAP091d
0 0 0 0 0 0 0 0 0 0 0 0 0 0 0 0 0 0 0 0 0 0 0 0

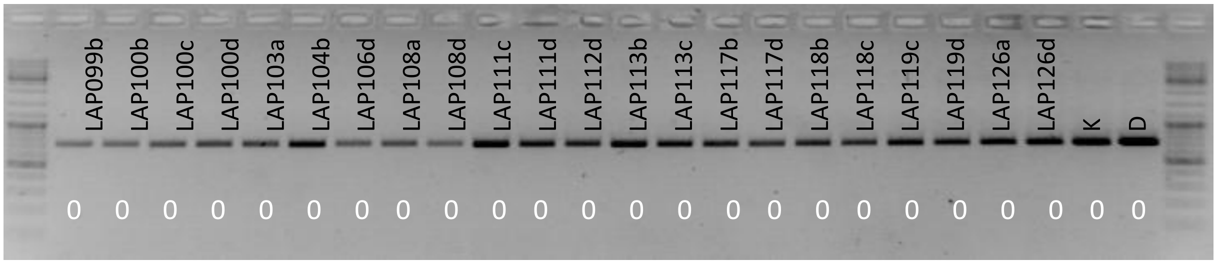
LAP099b
LAP100b
LAP100c
LAP100d
LAP103a
LAP104b
LAP106d
LAP108a
LAP108d
LAP111c
LAP111d
LAP112d
LAP113b
LAP113c
LAP117b
LAP117d
LAP118b
LAP118c
LAP119c
LAP119d
LAP126a
LAP126d
K
D
0 0 0 0 0 0 0 0 0 0 0 0 0 0 0 0 0 0 0 0 0 0 0 0

PR_38

PRFTc1_F3c GCAGATTGAGCCTATGATCCAAATG

PRFTC1R3 ACAGAGTGCCAGAAATTCCCA

Plate 1

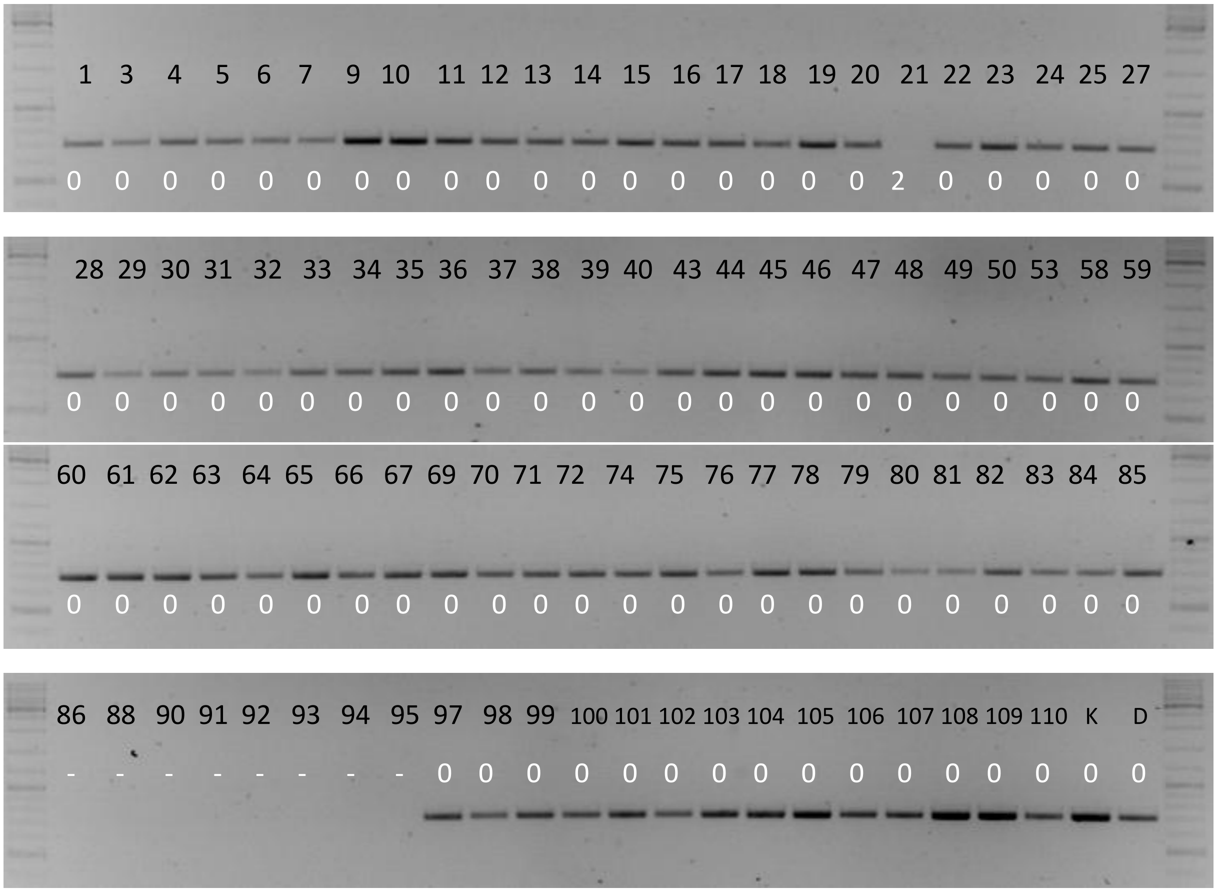

Plate 9

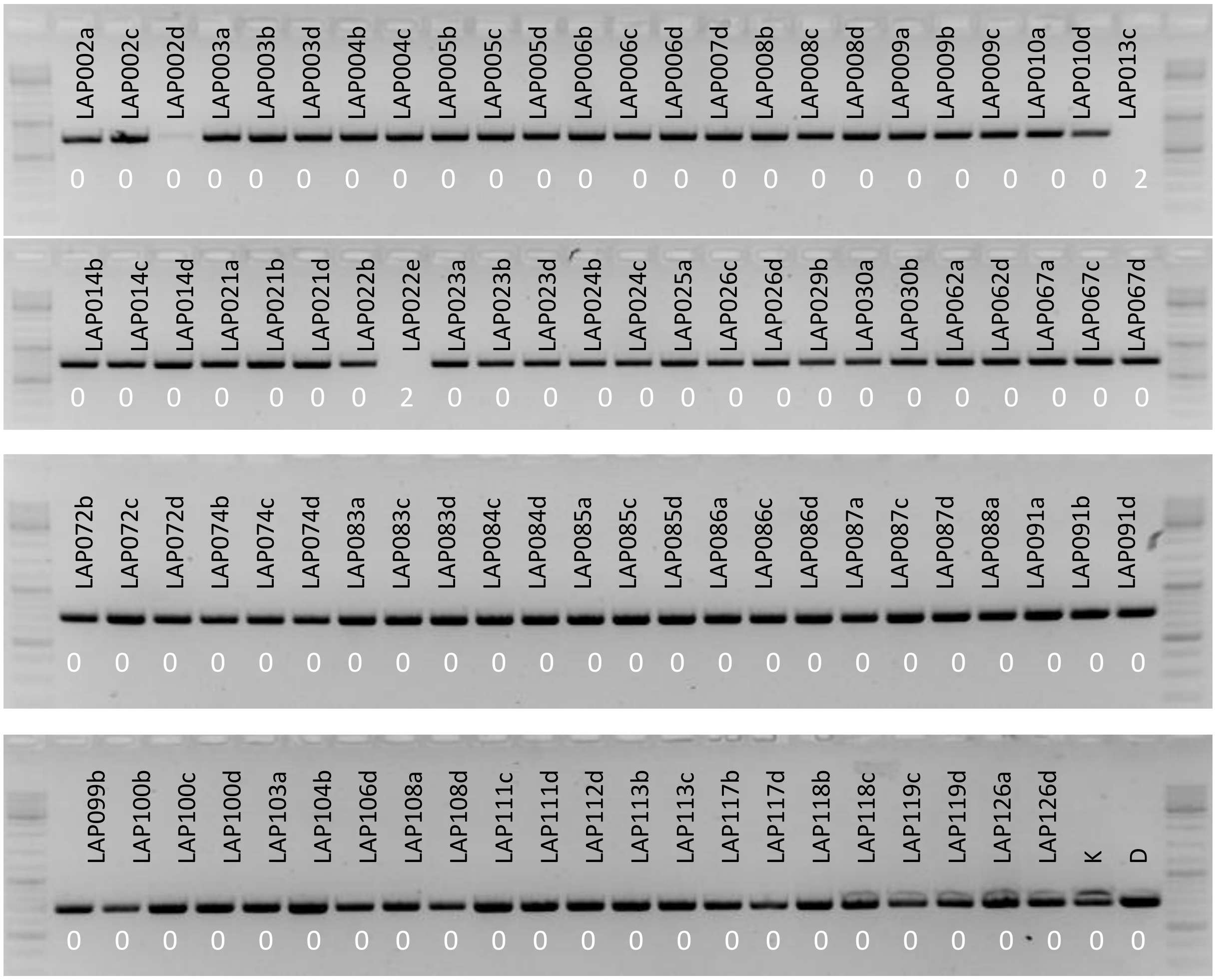
LAP002a LAP002c LAP002d LAP003a LAP003b LAP003d LAP004b LAP004c LAP005b LAP005c LAP005d LAP006b LAP006c LAP006d LAP007d LAP008b LAP008c LAP008d LAP009a LAP009b LAP009c LAP010a LAP010d LAP013c
0 0 0 0 0 0 0 0 0 0 0 0 0 0 0 0 0 0 0 0 0 0 0 2
LAP014b LAP014c LAP014d LAP021a LAP021b LAP021d LAP022b LAP022e LAP023a LAP023b LAP023d LAP024b LAP024c LAP025a LAP026c LAP026d LAP029b LAP030a LAP030b LAP062a LAP062d LAP067a LAP067c LAP067d
0 0 0 0 0 0 0 2 0 0 0 0 0 0 0 0 0 0 0 0 0 0 0 0
LAP072b LAP072c LAP072d LAP074b LAP074c LAP074d LAP083a LAP083c LAP083d LAP084c LAP084d LAP085a LAP085c LAP085d LAP086a LAP086c LAP086d LAP087a LAP087c LAP087d LAP088a LAP091a LAP091b LAP091d
0 0 0 0 0 0 0 0 0 0 0 0 0 0 0 0 0 0 0 0 0 0 0 0
LAP099b LAP100b LAP100c LAP100d LAP103a LAP104b LAP106d LAP108a LAP108d LAP111c LAP111d LAP112d LAP113b LAP113c LAP117b LAP117d LAP118b LAP118c LAP119c LAP119d LAP126a LAP126d K D
0 0 0 0 0 0 0 0 0 0 0 0 0 0 0 0 0 0 0 0 0 0 0 0

PR_39

PRFTC1F4 ATAAGATTGAATCCCTCTCTCGTCT

PRFTc1_R4b ACAACAAACACATAAATCTATACTGGA

Plate 1

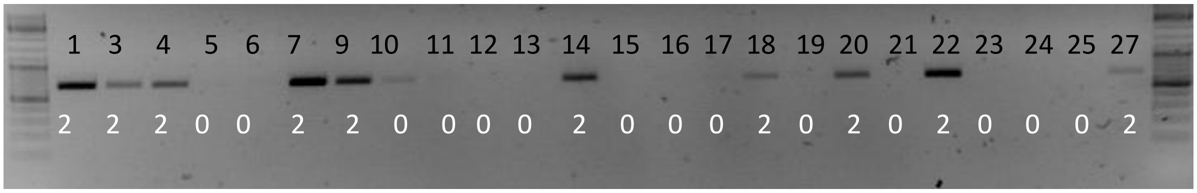

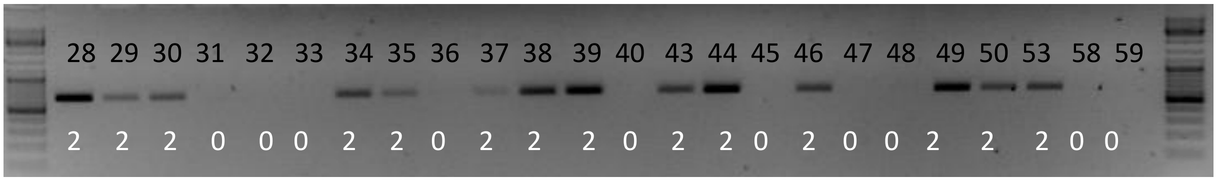

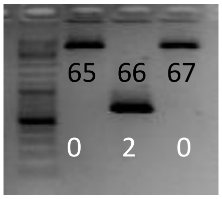

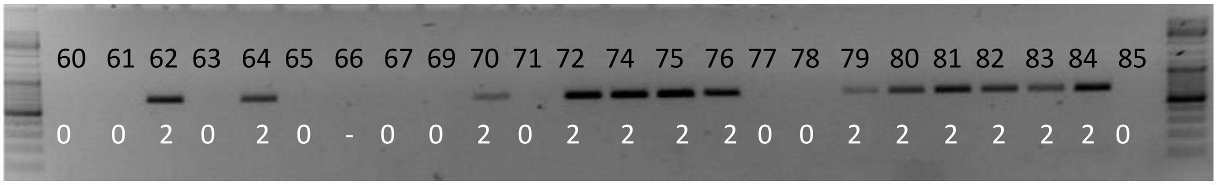

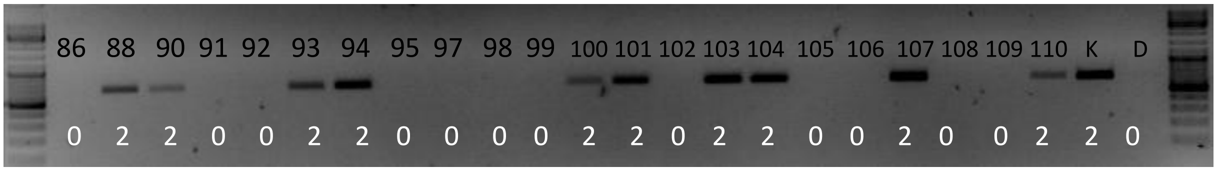

Plate 9

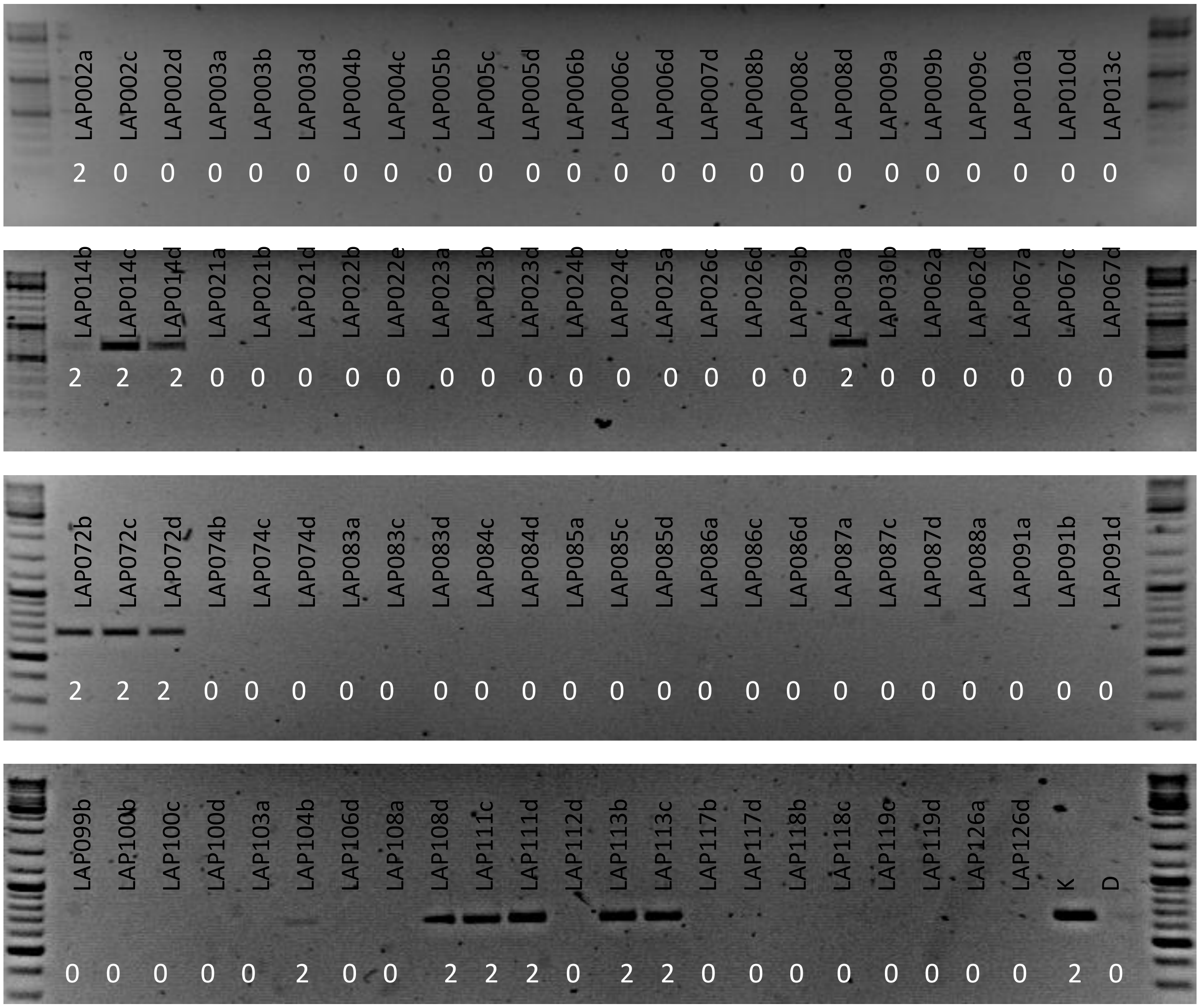
LAP002a LAP002c LAP002d LAP003a LAP003b LAP003d LAP004b LAP004c LAP005b LAP005c LAP005d LAP006b LAP006c LAP006d LAP007d LAP008b LAP008c LAP008d LAP009a LAP009b LAP009c LAP010a LAP010d LAP013c
2 0 0 0 0 0 0 0 0 0 0 0 0 0 0 0 0 0 0 0 0 0 0 0
LAP014b LAP014c LAP014d LAP021a LAP021b LAP021d LAP022b LAP022e LAP023a LAP023b LAP023d LAP024b LAP024c LAP025a LAP026c LAP026d LAP029b LAP030a LAP030b LAP062a LAP062d LAP067a LAP067c LAP067d
2 2 2 0 0 0 0 0 0 0 0 0 0 0 0 0 0 2 0 0 0 0 0 0
LAP072b LAP072c LAP072d LAP074b LAP074c LAP074d LAP083a LAP083c LAP083d LAP084c LAP084d LAP085a LAP085c LAP085d LAP086a LAP086c LAP086d LAP087a LAP087c LAP087d LAP088a LAP091a LAP091b LAP091d
2 2 2 0 0 0 0 0 0 0 0 0 0 0 0 0 0 0 0 0 0 0 0 0
LAP099b LAP100b LAP100c LAP100d LAP103a LAP104b LAP106d LAP108a LAP108d LAP111c LAP111d LAP112d LAP113b LAP113c LAP117b LAP117d LAP118b LAP118c LAP119c LAP119d LAP126a LAP126d K D
0 0 0 0 0 2 0 0 2 2 2 0 2 2 0 0 0 0 0 0 0 0 2 0

PR_40

PRFTc1_F4b GAGCCTTCTTGTCAGGGACGG

PRFTc1_R4c TATGGAGGAAGCATGTTGGAAGTAA

Plate 1

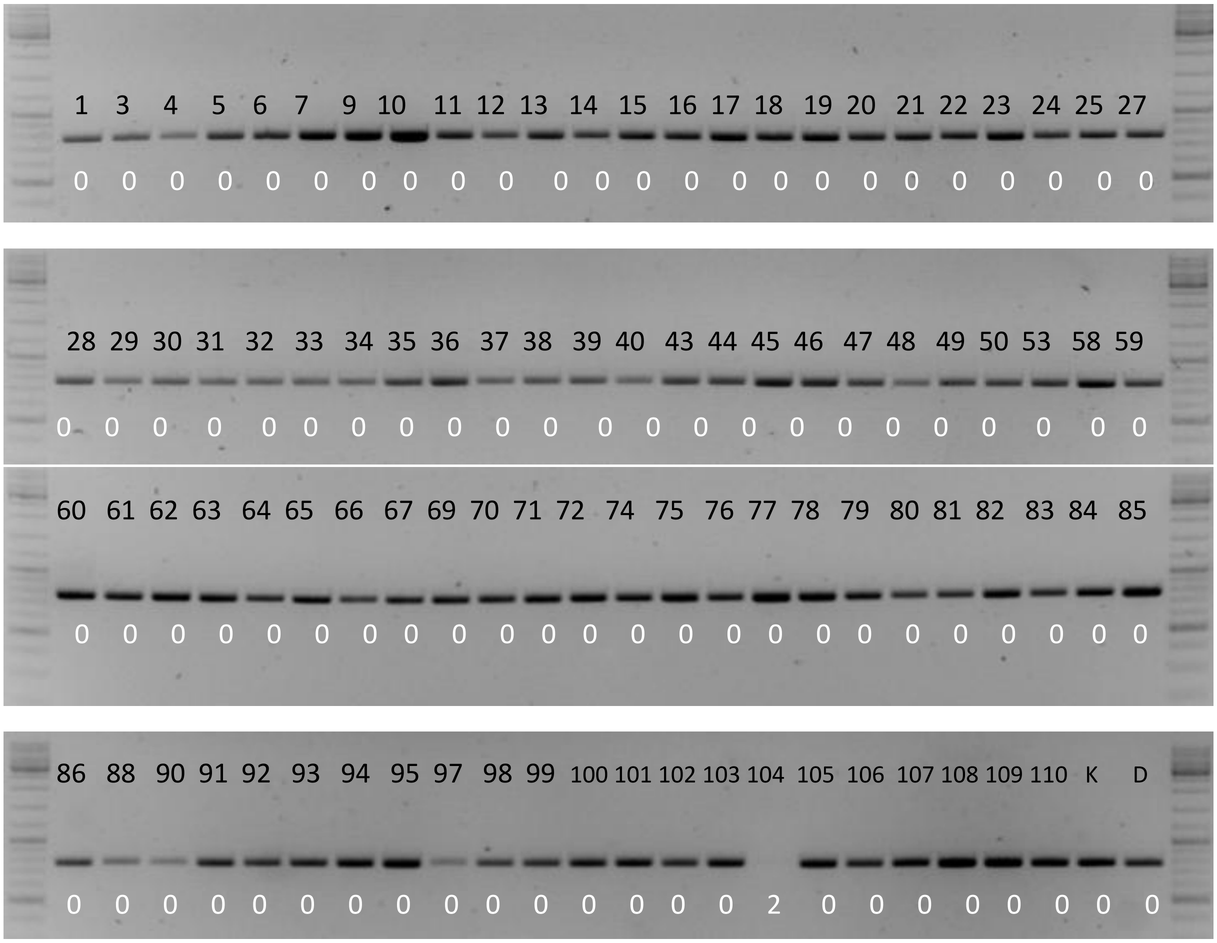

Plate 9

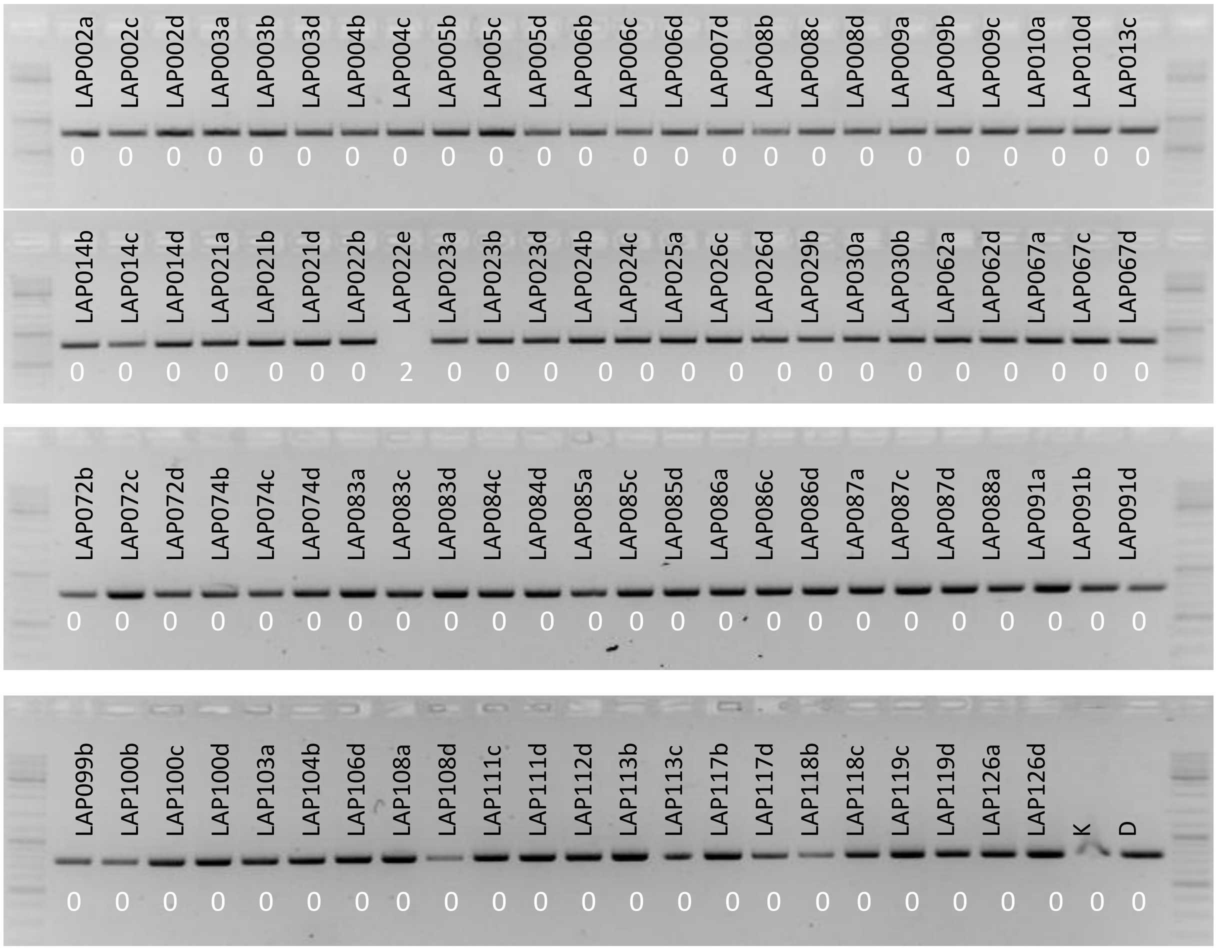
LAP002a LAP002c LAP002d LAP003a LAP003b LAP003d LAP004b LAP004c LAP005b LAP005c LAP005d LAP006b LAP006c LAP006d LAP007d LAP008b LAP008c LAP008d LAP009a LAP009b LAP009c LAP010a LAP010d LAP013c
0 0 0 0 0 0 0 0 0 0 0 0 0 0 0 0 0 0 0 0 0 0 0 0
LAP014b LAP014c LAP014d LAP021a LAP021b LAP021d LAP022b LAP022e LAP023a LAP023b LAP023d LAP024b LAP024c LAP025a LAP026c LAP026d LAP029b LAP030a LAP030b LAP062a LAP062d LAP067a LAP067c LAP067d
0 0 0 0 0 0 0 2 0 0 0 0 0 0 0 0 0 0 0 0 0 0 0 0
LAP072b LAP072c LAP072d LAP074b LAP074c LAP074d LAP083a LAP083c LAP083d LAP084c LAP084d LAP085a LAP085c LAP085d LAP086a LAP086c LAP086d LAP087a LAP087c LAP087d LAP088a LAP091a LAP091b LAP091d
0 0 0 0 0 0 0 0 0 0 0 0 0 0 0 0 0 0 0 0 0 0 0 0
LAP099b LAP100b LAP100c LAP100d LAP103a LAP104b LAP106d LAP108a LAP108d LAP111c LAP111d LAP112d LAP113b LAP113c LAP117b LAP117d LAP118b LAP118c LAP119c LAP119d LAP126a LAP126d K D
0 0 0 0 0 0 0 0 0 0 0 0 0 0 0 0 0 0 0 0 0 0 0 0

PR_41

PRFTc1_F4c TGCTATAGTTATTGCTGTTCCACAA

PRFTC1R4 GGAAACAATGCAACAGTTGAATGA

Plate 1

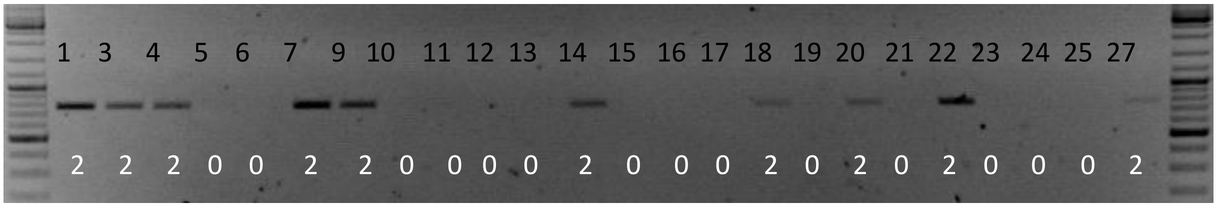

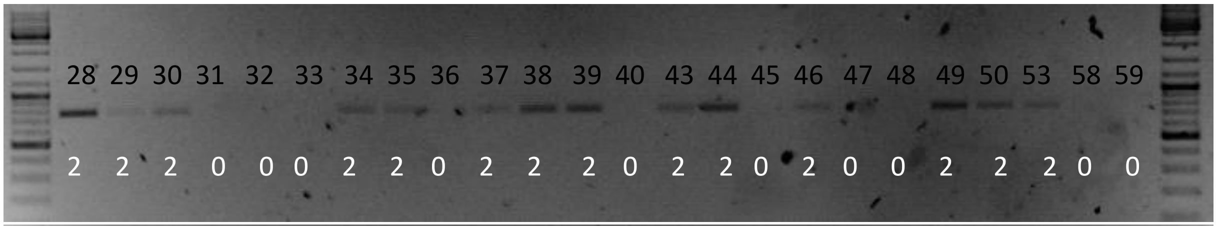

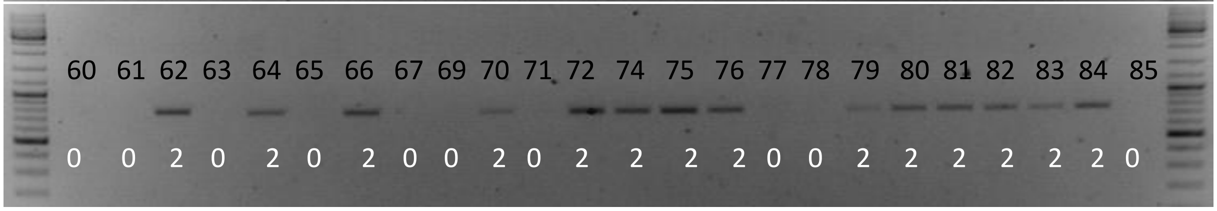

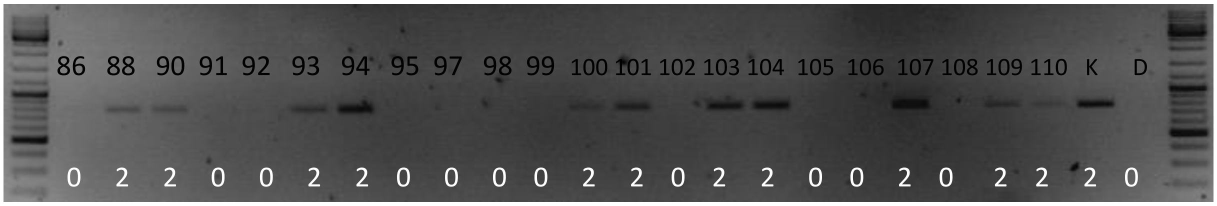

Plate 9

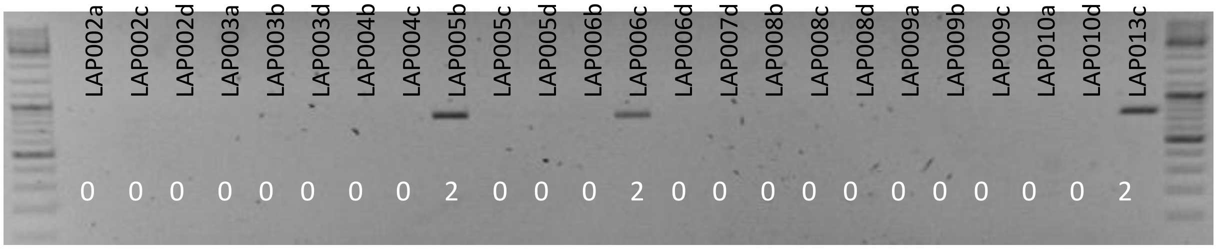
LAP002a
LAP002c
LAP002d
LAP003a
LAP003b
LAP003d
LAP004b
LAP004c
LAP005b
LAP005c
LAP005d
LAP006b
LAP006c
LAP006d
LAP007d
LAP008b
LAP008c
LAP008d
LAP009a
LAP009b
LAP009c
LAP010a
LAP010d
LAP013c
0 0 0 0 0 0 0 0 2 0 0 0 2 0 0 0 0 0 0 0 0 0 0 2

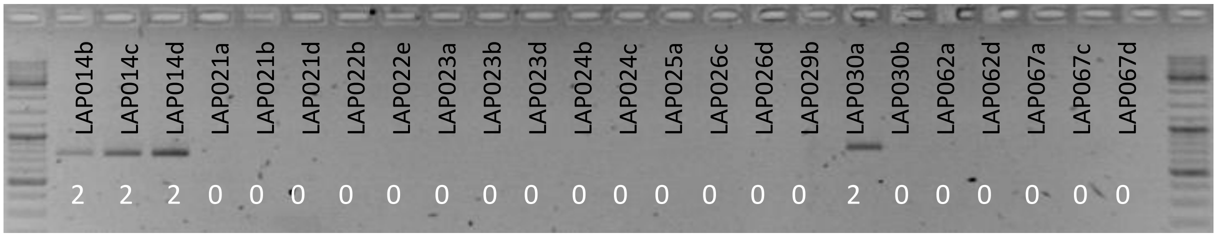
LAP014b
LAP014c
LAP014d
LAP021a
LAP021b
LAP021d
LAP022b
LAP022e
LAP023a
LAP023b
LAP023d
LAP024b
LAP024c
LAP025a
LAP026c
LAP026d
LAP029b
LAP030a
LAP030b
LAP062a
LAP062d
LAP067a
LAP067c
LAP067d
2 2 2 0 0 0 0 0 0 0 0 0 0 0 0 0 0 2 0 0 0 0 0 0

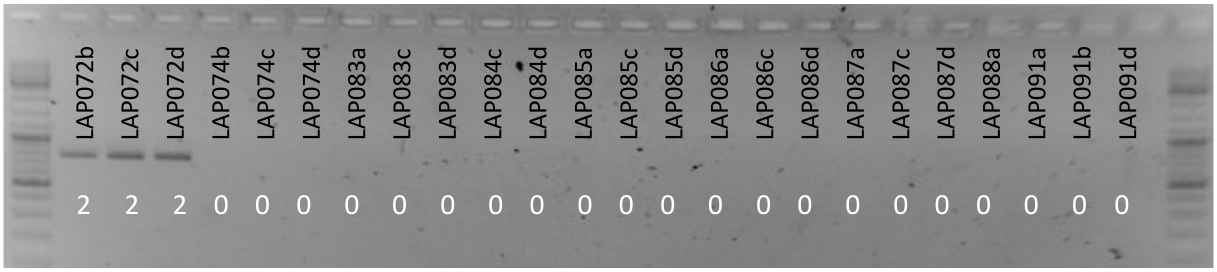
LAP072b
LAP072c
LAP072d
LAP074b
LAP074c
LAP074d
LAP083a
LAP083c
LAP083d
LAP084c
LAP084d
LAP085a
LAP085c
LAP085d
LAP086a
LAP086c
LAP086d
LAP087a
LAP087c
LAP087d
LAP088a
LAP091a
LAP091b
LAP091d
2 2 2 0 0 0 0 0 0 0 0 0 0 0 0 0 0 0 0 0 0 0 0 0

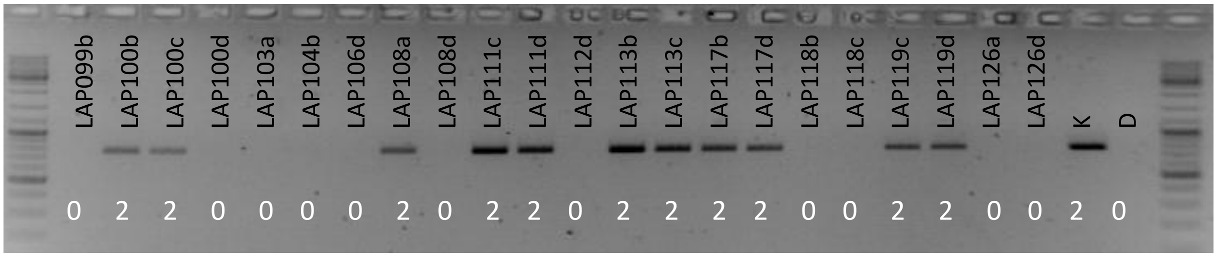
LAP099b
LAP100b
LAP100c
LAP100d
LAP103a
LAP104b
LAP106d
LAP108a
LAP108d
LAP111c
LAP111d
LAP112d
LAP113b
LAP113c
LAP117b
LAP117d
LAP118b
LAP118c
LAP119c
LAP119d
LAP126a
LAP126d
K
D
0 2 2 0 0 0 0 2 0 2 2 0 2 2 2 2 0 0 2 2 0 0 2 0

PR_42a

PRFTC1F5 GGAGAAACTCGCTGGAAATTGC

PRFTc1_R5b AGAAGCTTAATGAACTGAAGCAATT

Plate 1

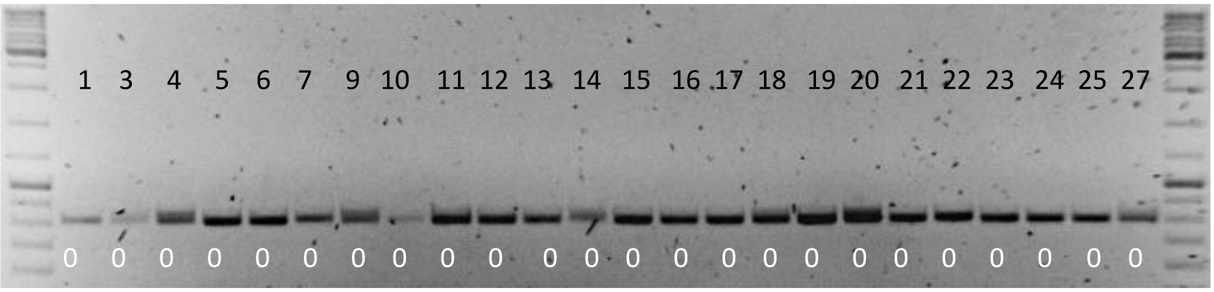

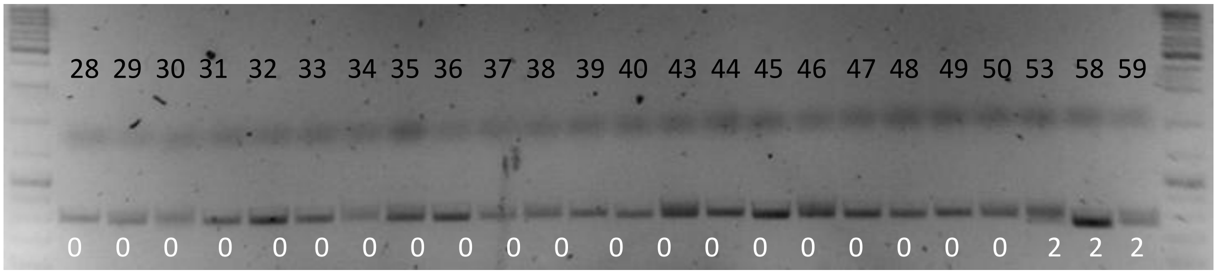

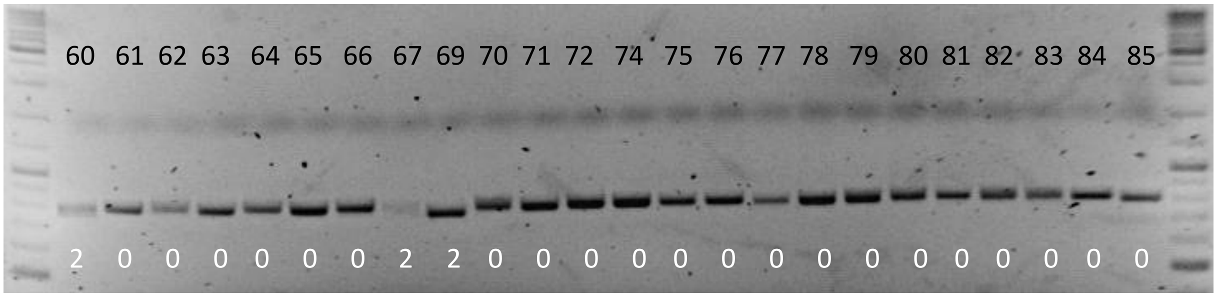

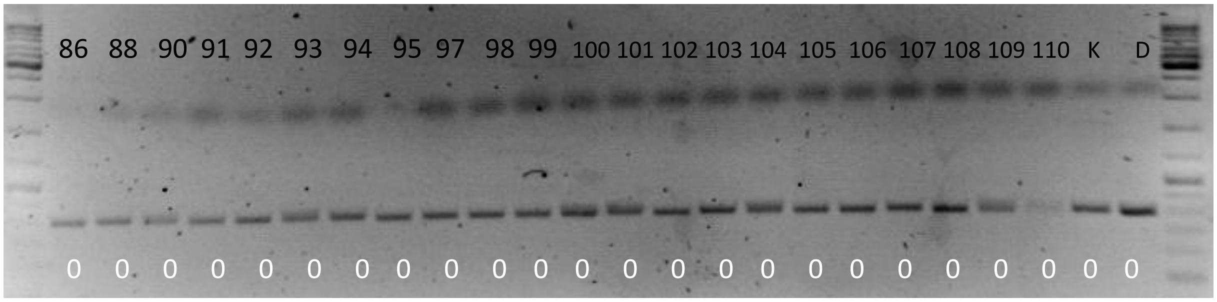

Plate 9

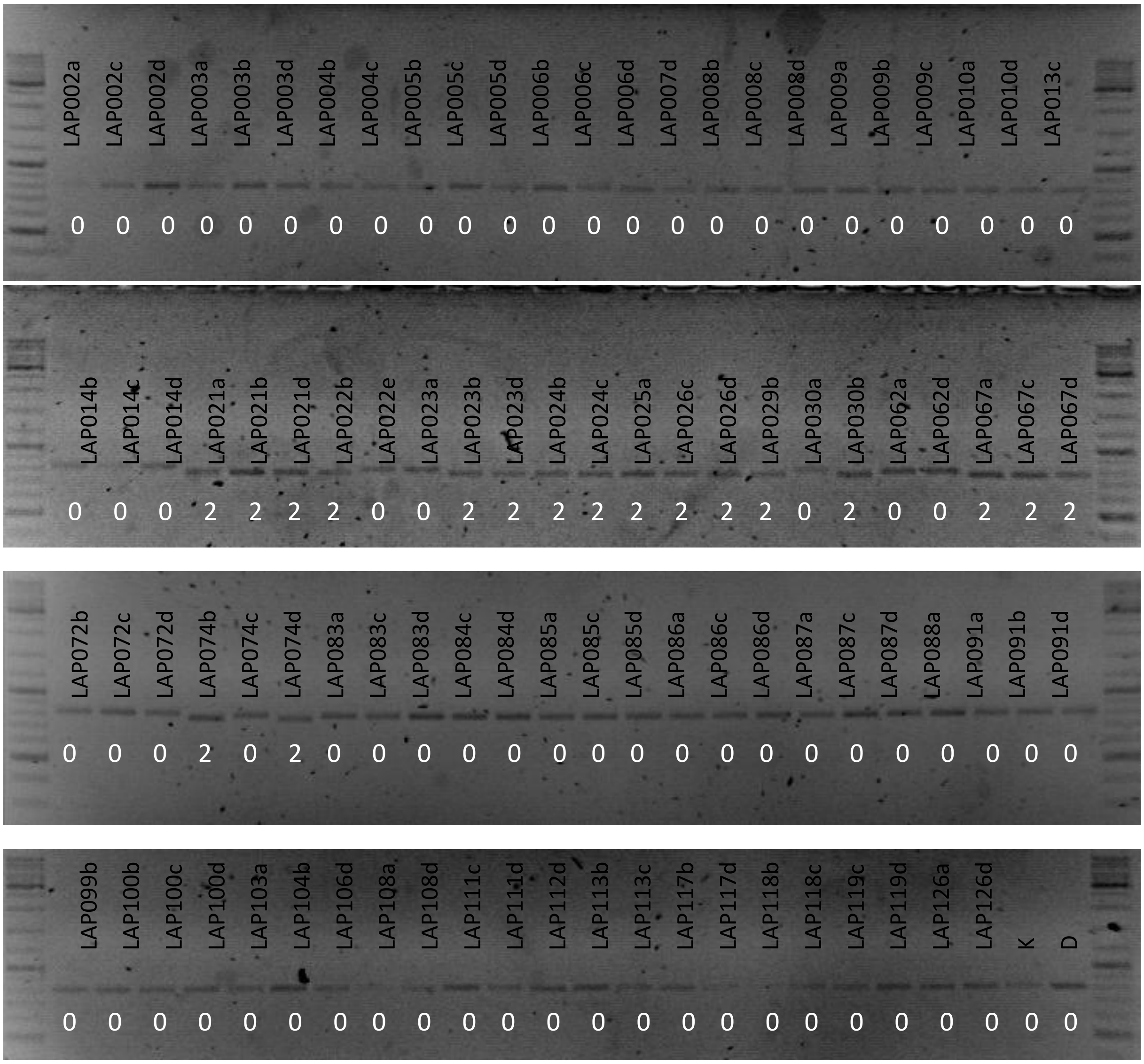
LAP002a LAP002c LAP002d LAP003a LAP003b LAP003d LAP004b LAP004c LAP005b LAP005c LAP005d LAP006b LAP006c LAP006d LAP007d LAP008b LAP008c LAP008d LAP009a LAP009b LAP009c LAP010a LAP010d LAP013c
0 0 0 0 0 0 0 0 0 0 0 0 0 0 0 0 0 0 0 0 0 0 0 0
LAP014b LAP014c LAP014d LAP021a LAP021b LAP021d LAP022b LAP022e LAP023a LAP023b LAP023d LAP024b LAP024c LAP025a LAP026c LAP026d LAP029b LAP030a LAP030b LAP062a LAP062d LAP067a LAP067c LAP067d
0 0 0 2 2 2 2 0 0 2 2 2 2 2 2 2 2 0 2 0 0 2 2 2
LAP072b LAP072c LAP072d LAP074b LAP074c LAP074d LAP083a LAP083c LAP083d LAP084c LAP084d LAP085a LAP085c LAP085d LAP086a LAP086c LAP086d LAP087a LAP087c LAP087d LAP088a LAP091a LAP091b LAP091d
0 0 0 2 0 2 0 0 0 0 0 0 0 0 0 0 0 0 0 0 0 0 0 0
LAP099b LAP100b LAP100c LAP100d LAP103a LAP104b LAP106d LAP108a LAP108d LAP111c LAP111d LAP112d LAP113b LAP113c LAP117b LAP117d LAP118b LAP118c LAP119c LAP119d LAP126a LAP126d K D
0 0 0 0 0 0 0 0 0 0 0 0 0 0 0 0 0 0 0 0 0 0 0 0

PR_42b

PRFTC1F5 GGAGAAACTCGCTGGAAATTGC

PRFTc1_R5b AGAAGCTTAATGAACTGAAGCAATT

Plate 1

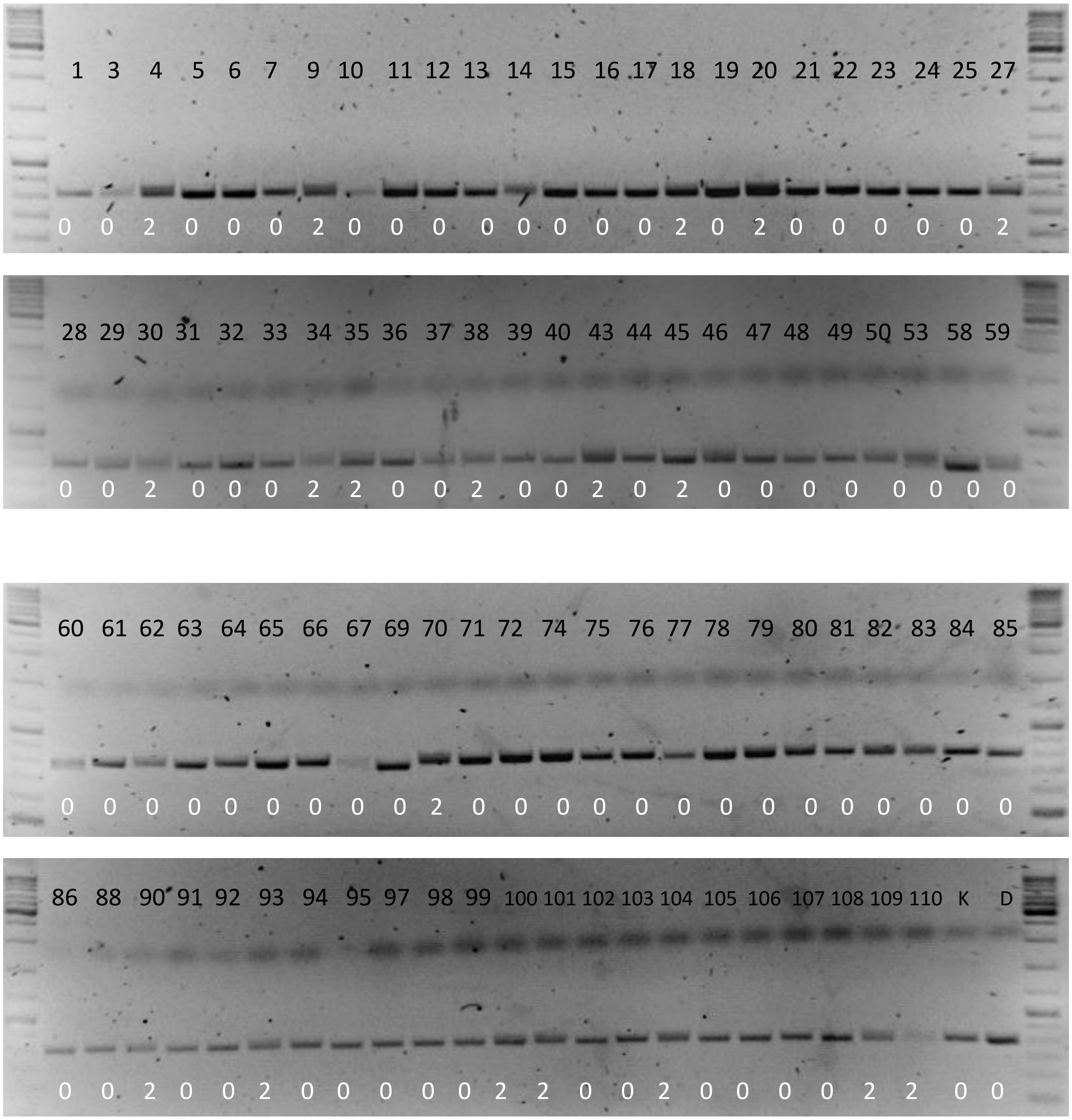

Plate 9

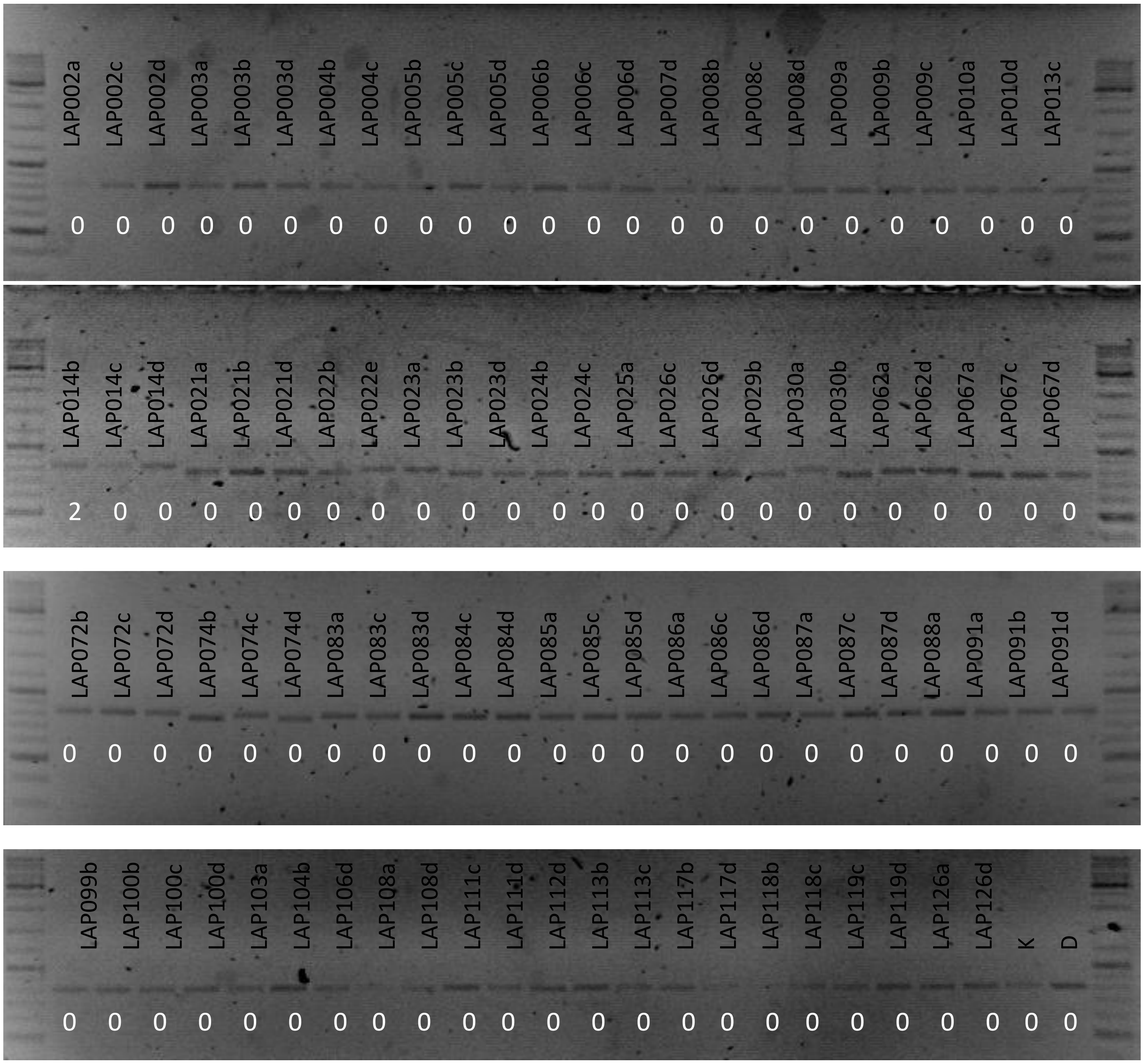
LAP002a LAP002c LAP002d LAP003a LAP003b LAP003d LAP004b LAP004c LAP005b LAP005c LAP005d LAP006b LAP006c LAP006d LAP007d LAP008b LAP008c LAP008d LAP009a LAP009b LAP009c LAP010a LAP010d LAP013c
0 0 0 0 0 0 0 0 0 0 0 0 0 0 0 0 0 0 0 0 0 0 0 0
LAP014b LAP014c LAP014d LAP021a LAP021b LAP021d LAP022b LAP022e LAP023a LAP023b LAP023d LAP024b LAP024c LAP025a LAP026c LAP026d LAP029b LAP030a LAP030b LAP062a LAP062d LAP067a LAP067c LAP067d
2 0 0 0 0 0 0 0 0 0 0 0 0 0 0 0 0 0 0 0 0 0 0 0
LAP072b LAP072c LAP072d LAP074b LAP074c LAP074d LAP083a LAP083c LAP083d LAP084c LAP084d LAP085a LAP085c LAP085d LAP086a LAP086c LAP086d LAP087a LAP087c LAP087d LAP088a LAP091a LAP091b LAP091d
0 0 0 0 0 0 0 0 0 0 0 0 0 0 0 0 0 0 0 0 0 0 0 0
LAP099b LAP100b LAP100c LAP100d LAP103a LAP104b LAP106d LAP108a LAP108d LAP111c LAP111d LAP112d LAP113b LAP113c LAP117b LAP117d LAP118b LAP118c LAP119c LAP119d LAP126a LAP126d K D
0 0 0 0 0 0 0 0 0 0 0 0 0 0 0 0 0 0 0 0 0 0 0 0

PR_43

PRFTc1_F5b TGTCTGTGTTGCTGTTATTATTGTCT

PRFTC1R5 TGAAAGAAACCCTAAGAGAAACTAAGG

Plate 1

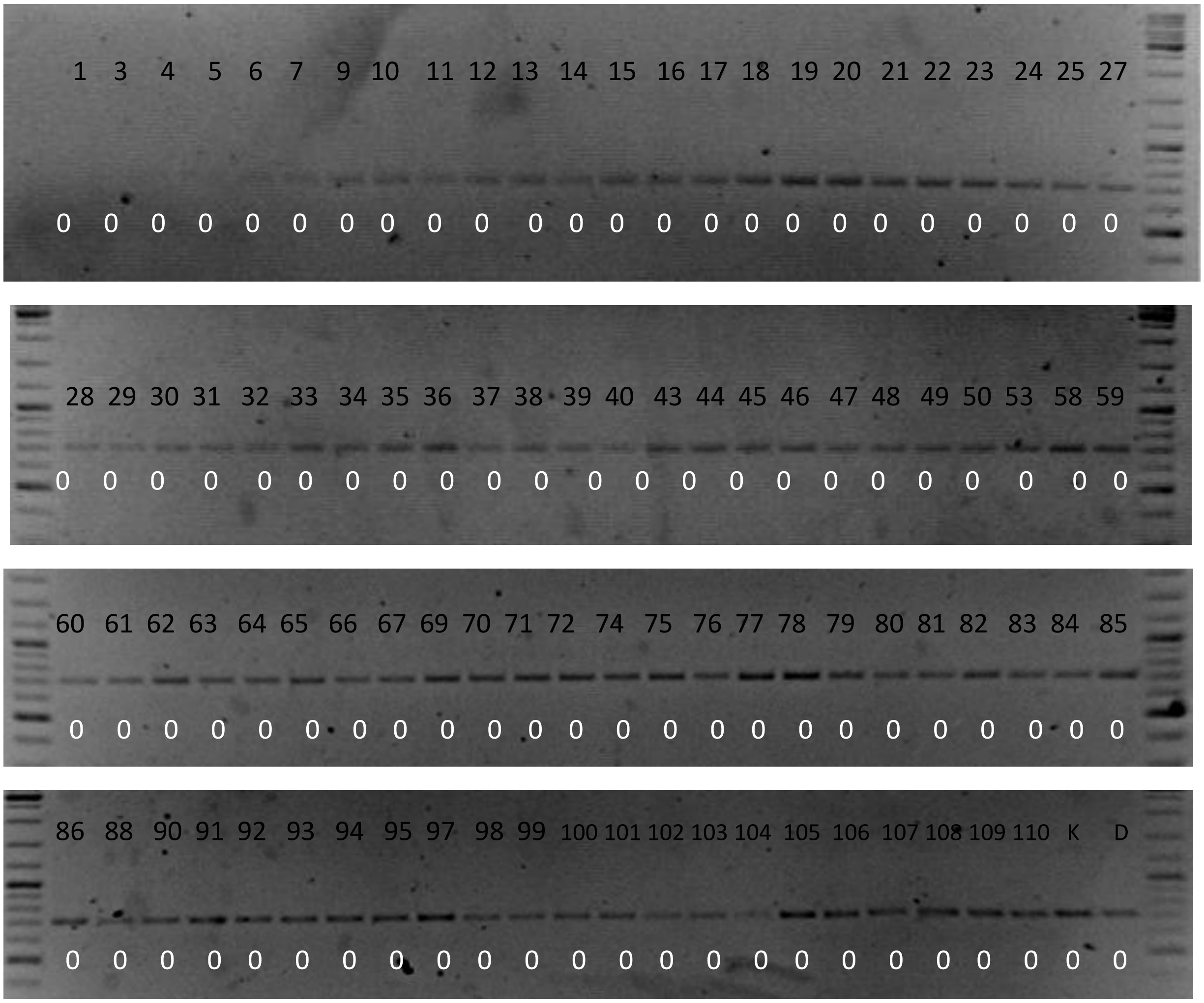

Plate 9

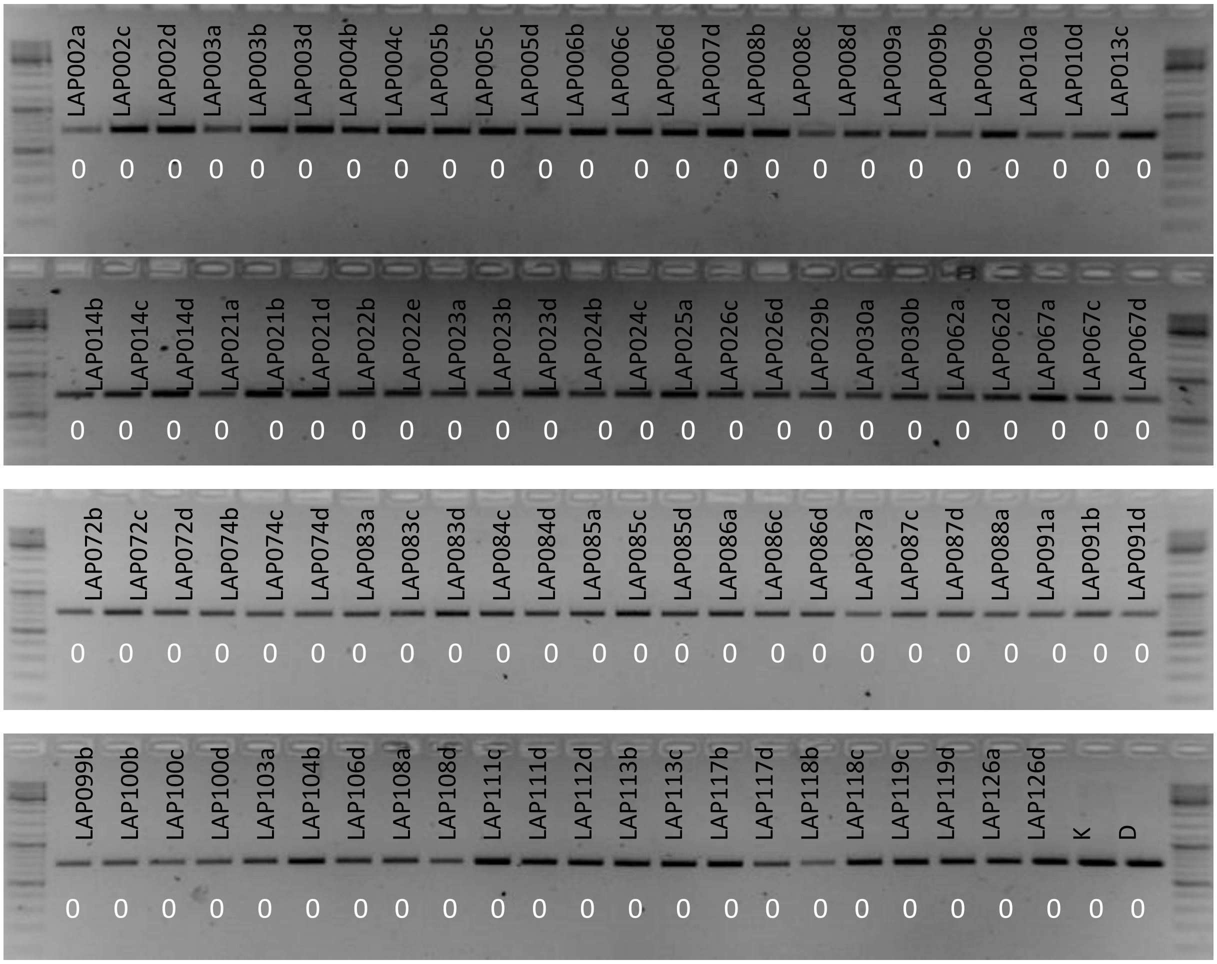
LAP002a LAP002c LAP002d LAP003a LAP003b LAP003d LAP004b LAP004c LAP005b LAP005c LAP005d LAP006b LAP006c LAP006d LAP007d LAP008b LAP008c LAP008d LAP009a LAP009b LAP009c LAP010a LAP010d LAP013c
0 0 0 0 0 0 0 0 0 0 0 0 0 0 0 0 0 0 0 0 0 0 0 0
LAP014b LAP014c LAP014d LAP021a LAP021b LAP021d LAP022b LAP022e LAP023a LAP023b LAP023d LAP024b LAP024c LAP025a LAP026c LAP026d LAP029b LAP030a LAP030b LAP062a LAP062d LAP067a LAP067c LAP067d
0 0 0 0 0 0 0 0 0 0 0 0 0 0 0 0 0 0 0 0 0 0 0 0
LAP072b LAP072c LAP072d LAP074b LAP074c LAP074d LAP083a LAP083c LAP083d LAP084c LAP084d LAP085a LAP085c LAP085d LAP086a LAP086c LAP086d LAP087a LAP087c LAP087d LAP088a LAP091a LAP091b LAP091d
0 0 0 0 0 0 0 0 0 0 0 0 0 0 0 0 0 0 0 0 0 0 0 0
LAP099b LAP100b LAP100c LAP100d LAP103a LAP104b LAP106d LAP108a LAP108d LAP111c LAP111d LAP112d LAP113b LAP113c LAP117b LAP117d LAP118b LAP118c LAP119c LAP119d LAP126a LAP126d K D
0 0 0 0 0 0 0 0 0 0 0 0 0 0 0 0 0 0 0 0 0 0 0 0

PR_58a

PRFTc1F1 TGGCATTGTAGATTAGGTCATGT

PRFTc1R1 GCACAAAAACAAGCAGAACAA

Plate 1

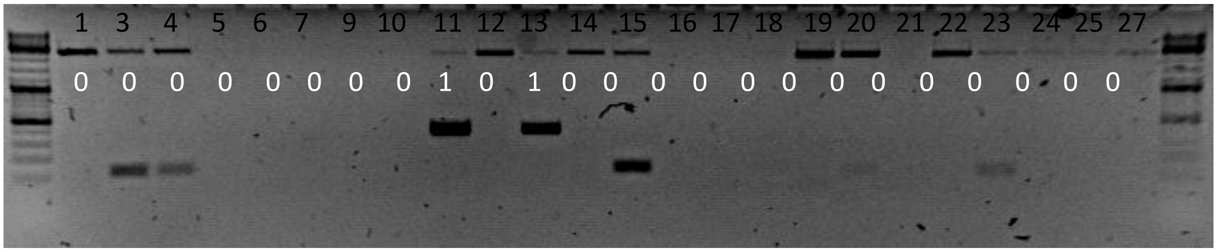

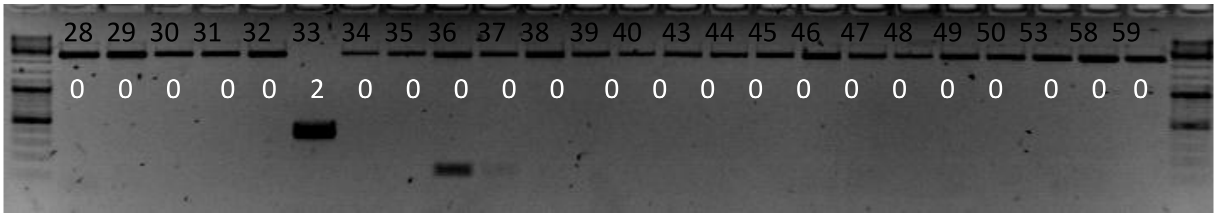

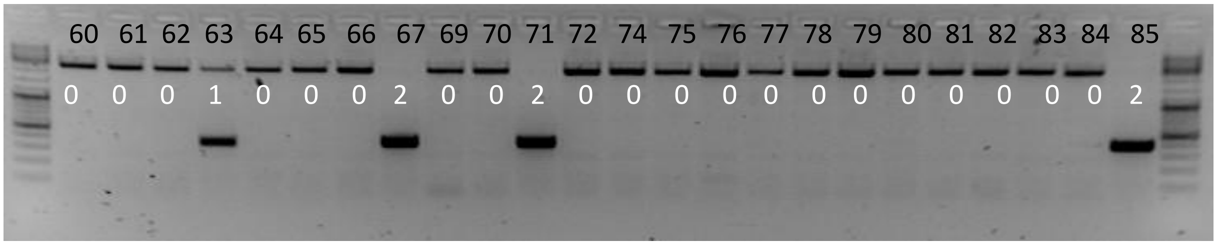

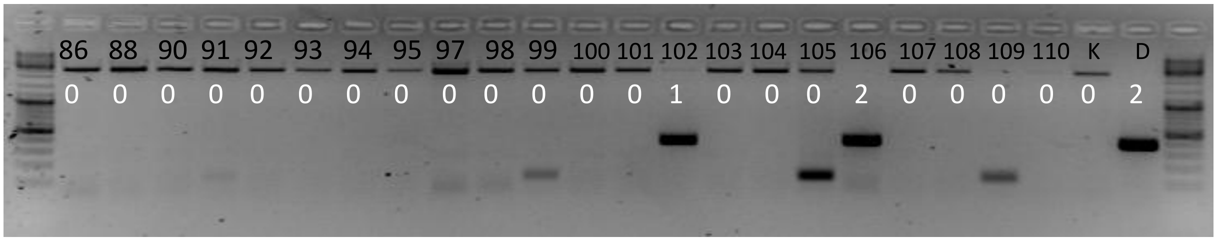

Plate 7

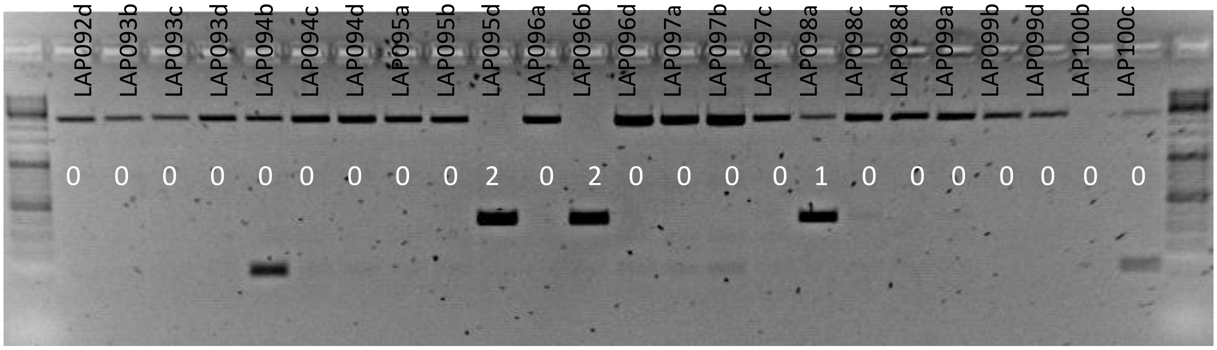
LAP092d LAP093b LAP093c LAP093d LAP094b LAP094c LAP094d LAP095a LAP095b LAP095d LAP096a LAP096b LAP096d LAP097a LAP097b LAP097c LAP098a LAP098c LAP098d LAP099a LAP099b LAP099d LAP100b LAP100c
0 0 0 0 0 0 0 0 0 2 0 2 0 0 0 0 1 0 0 0 0 0 0 0

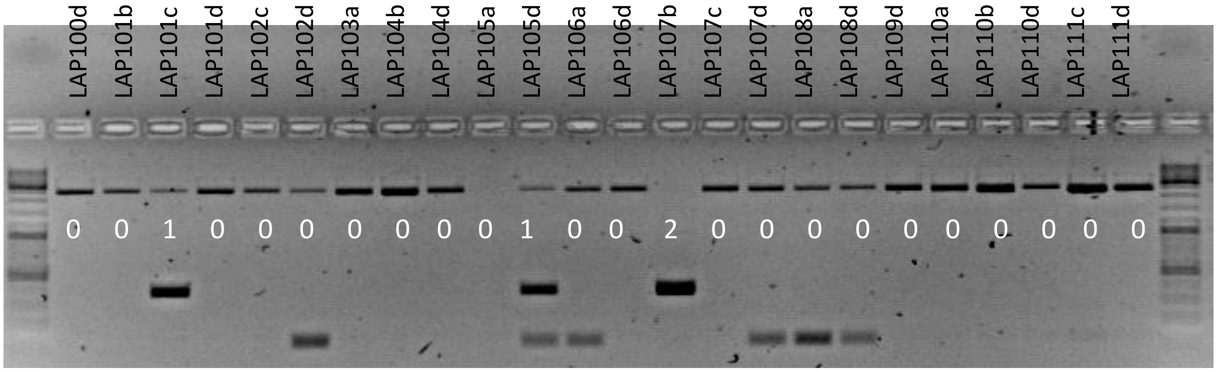
LAP100d LAP101b LAP101c LAP101d LAP102c LAP102d LAP103a LAP104b LAP104d LAP105a LAP105d LAP106a LAP106d LAP107b LAP107c LAP107d LAP108a LAP108d LAP109d LAP110a LAP110b LAP110d LAP111c LAP111d
0 0 1 0 0 0 0 0 0 0 1 0 0 2 0 0 0 0 0 0 0 0 0 0

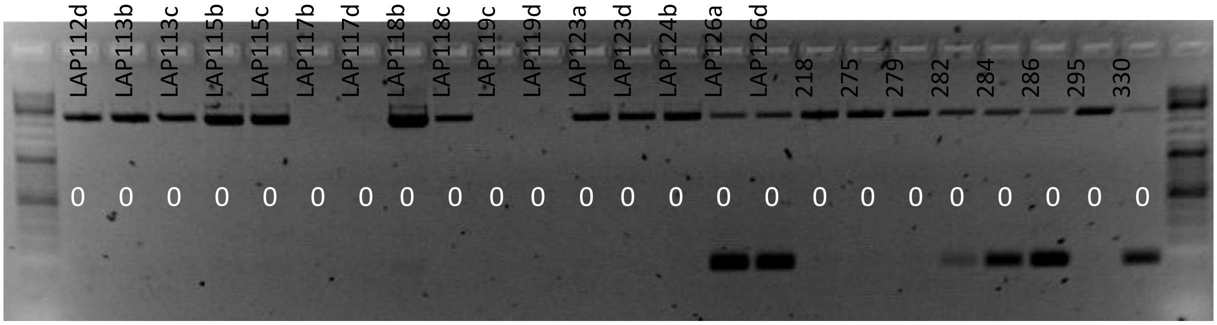
LAP112d LAP113b LAP113c LAP115b LAP115c LAP117b LAP117d LAP118b LAP118c LAP119c LAP119d LAP123a LAP123d LAP124b LAP126a LAP126d 218 275 279 282 284 286 295 330
0 0 0 0 0 0 0 0 0 0 0 0 0 0 0 0 0 0 0 0 0 0 0 0

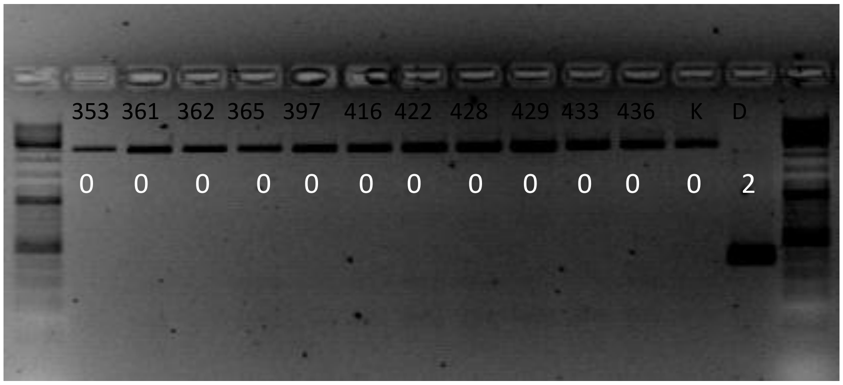
353 361 362 365 397 416 422 428 429 433 436 K D
0 0 0 0 0 0 0 0 0 0 0 0 2

PR_58b

PRFTc1F1 TGGCATTGTAGATTAGGTCATGT

PRFTc1R1 GCACAAAAACAAGCAGAACAA

Plate 1

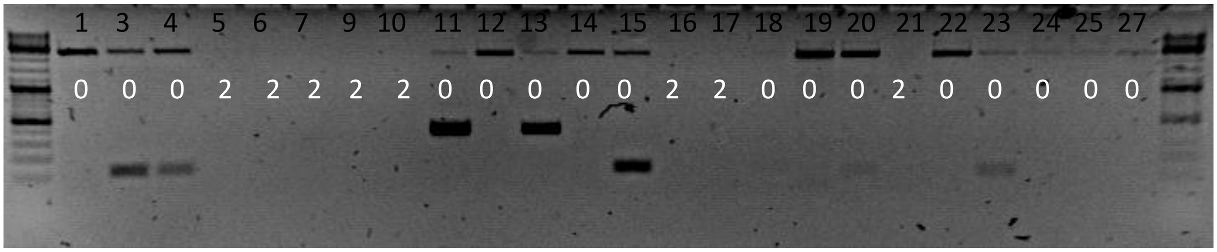

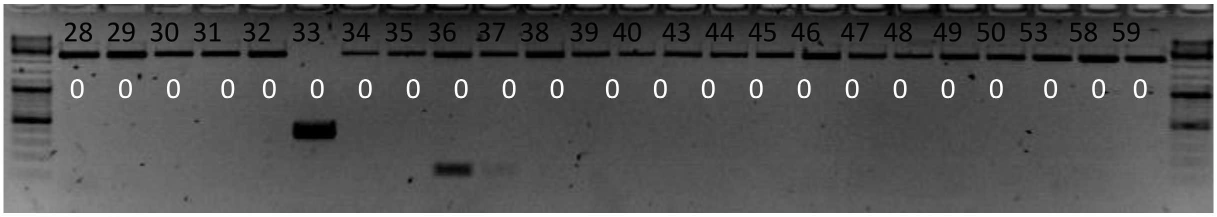

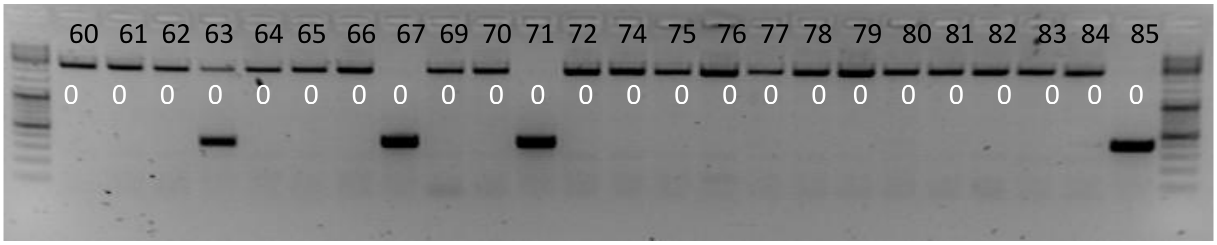

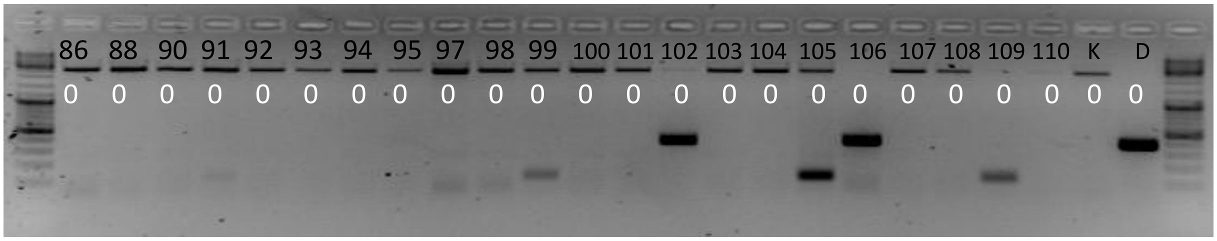

Plate 7

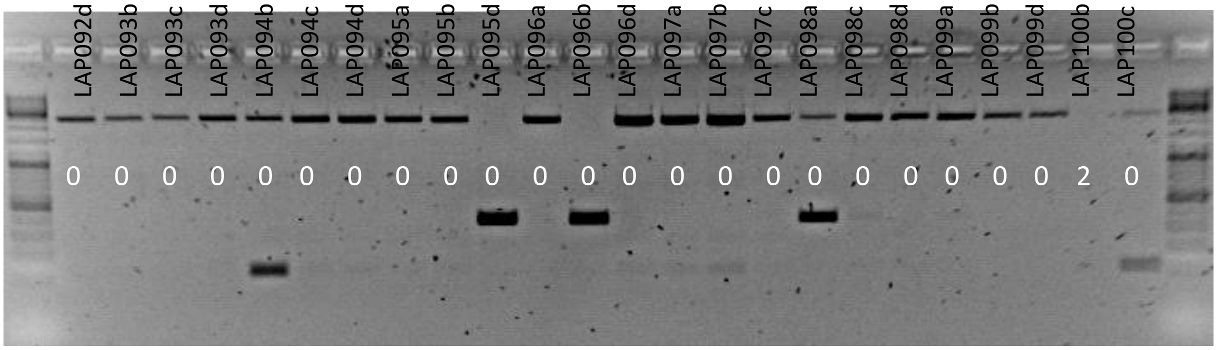

LAP092d LAP093b LAP093c LAP093d LAP094b LAP094c LAP094d LAP095a LAP095b LAP095d LAP096a LAP096b LAP096d LAP097a LAP097b LAP097c LAP098a LAP098c LAP098d LAP099a LAP099b LAP099d LAP100b LAP100c
0 0 0 0 0 0 0 0 0 0 0 0 0 0 0 0 0 0 0 0 0 0 2 0

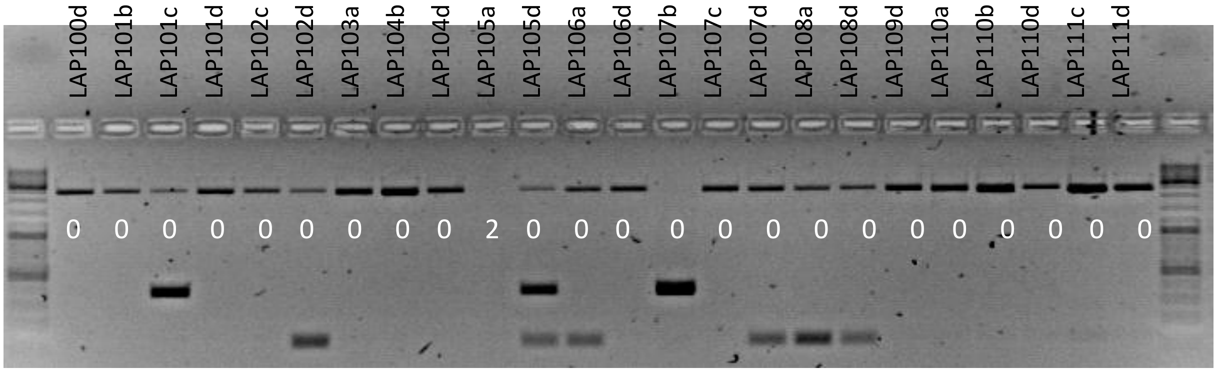

LAP100d LAP101b LAP101c LAP101d LAP102c LAP102d LAP103a LAP104b LAP104d LAP105a LAP105d LAP106a LAP106d LAP107b LAP107c LAP107d LAP108a LAP108d LAP109d LAP110a LAP110b LAP110d LAP111c LAP111d
0 0 0 0 0 0 0 0 0 2 0 0 0 0 0 0 0 0 0 0 0 0 0 0

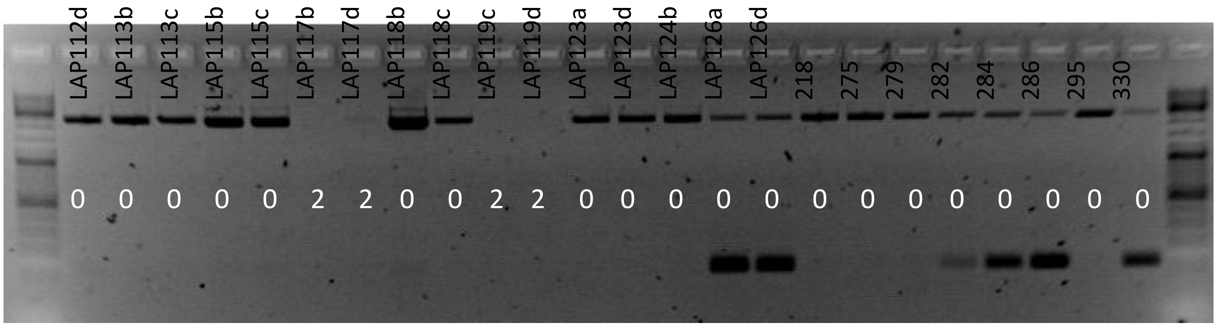

LAP112d LAP113b LAP113c LAP115b LAP115c LAP117b LAP117d LAP118b LAP118c LAP119c LAP119d LAP123a LAP123d LAP124b LAP126a LAP126d 218 275 279 282 284 286 295 330
0 0 0 0 0 2 2 0 0 2 2 0 0 0 0 0 0 0 0 0 0 0 0 0

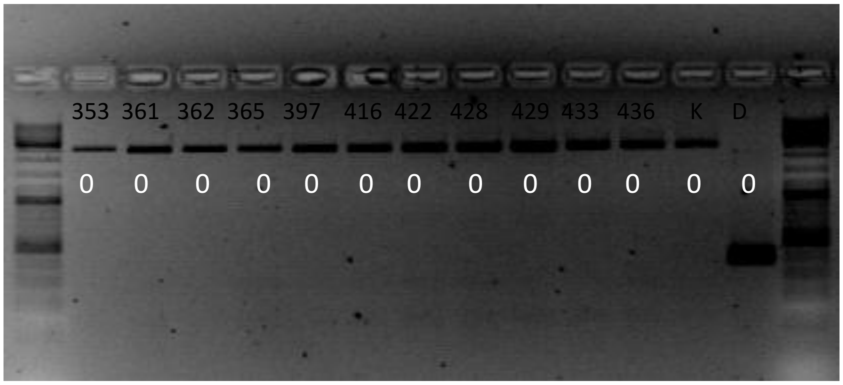

353 361 362 365 397 416 422 428 429 433 436 K D
0 0 0 0 0 0 0 0 0 0 0 0 0

PR_58c

PRFTc1F1 TGGCATTGTAGATTAGGTCATGT

PRFTc1R1 GCACAAAAACAAGCAGAACAA

Plate 1

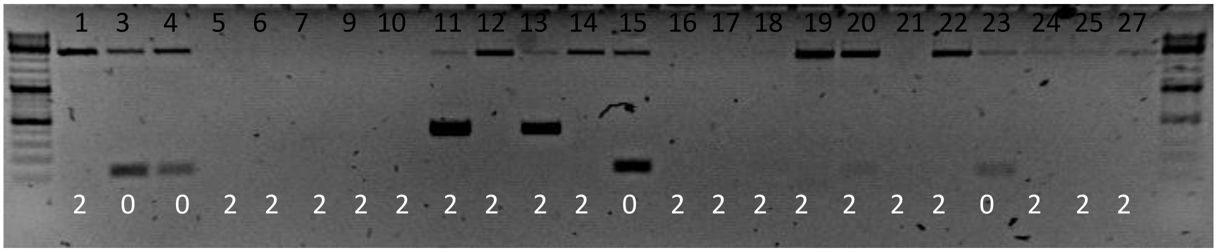

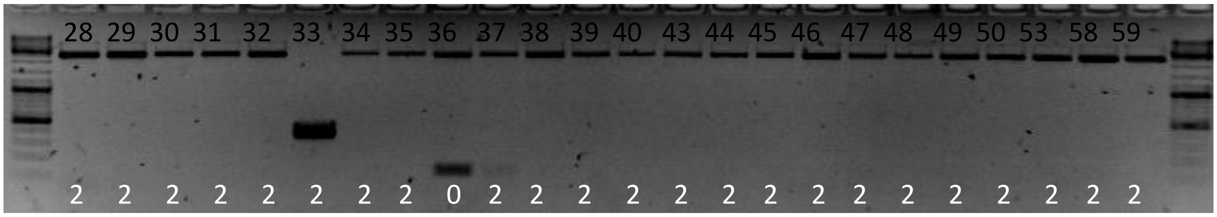

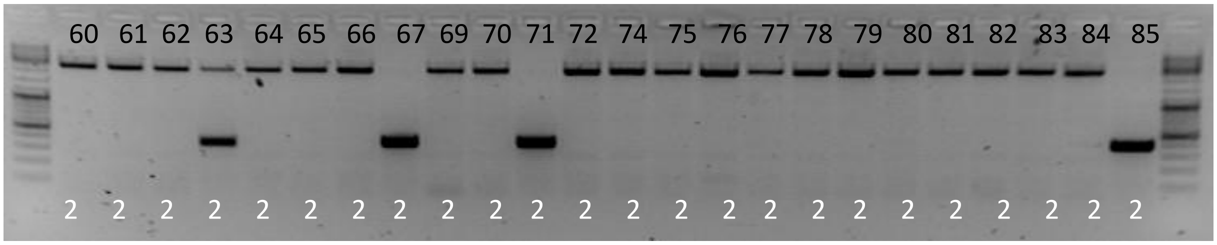

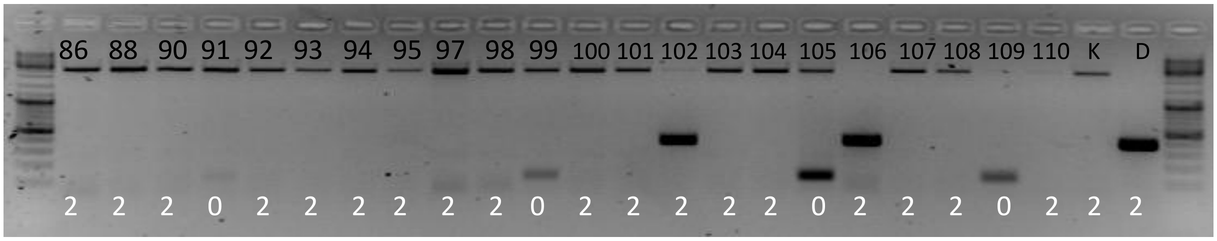

Plate 7

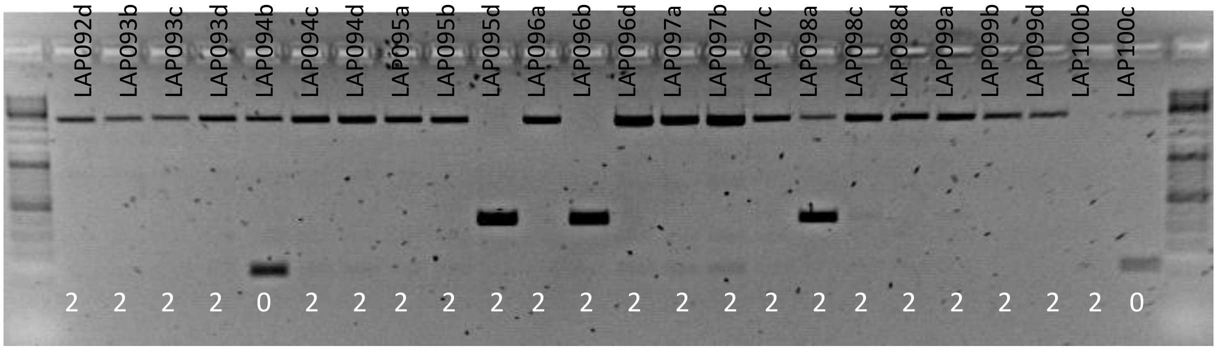
LAP092d LAP093b LAP093c LAP093d LAP094b LAP094c LAP094d LAP095a LAP095b LAP095d LAP096a LAP096b LAP096d LAP097a LAP097b LAP097c LAP098a LAP098c LAP098d LAP099a LAP099b LAP099d LAP100b LAP100c
2 2 2 2 0 2 2 2 2 2 2 2 2 2 2 2 2 2 2 2 2 2 2 0

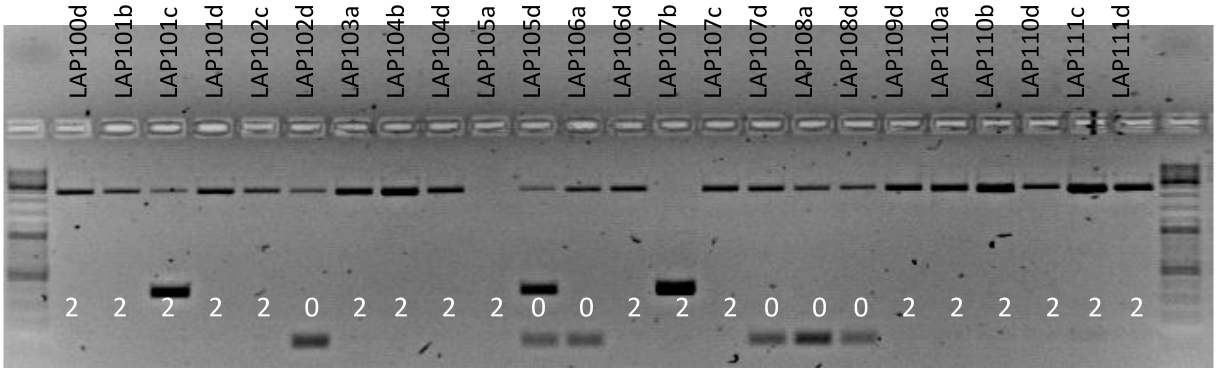
LAP100d LAP101b LAP101c LAP101d LAP102c LAP102d LAP103a LAP104b LAP104d LAP105a LAP105d LAP106a LAP106d LAP107b LAP107c LAP107d LAP108a LAP108d LAP109d LAP110a LAP110b LAP110d LAP111c LAP111d
2 2 2 2 2 0 2 2 2 2 0 0 2 2 2 0 0 0 2 2 2 2 2 2

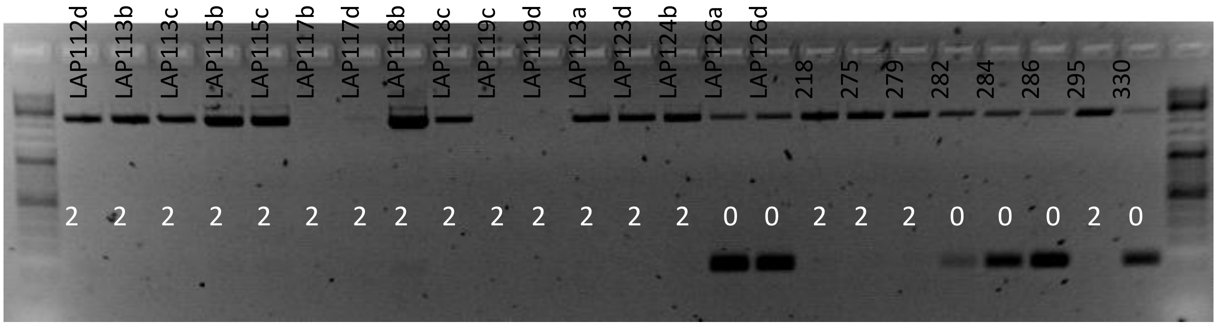
LAP112d LAP113b LAP113c LAP115b LAP115c LAP117b LAP117d LAP118b LAP118c LAP119c LAP119d LAP123a LAP123d LAP124b LAP126a LAP126d 218 275 279 282 284 286 295 330
2 2 2 2 2 2 2 2 2 2 2 2 2 2 0 0 2 2 2 0 0 0 2 0

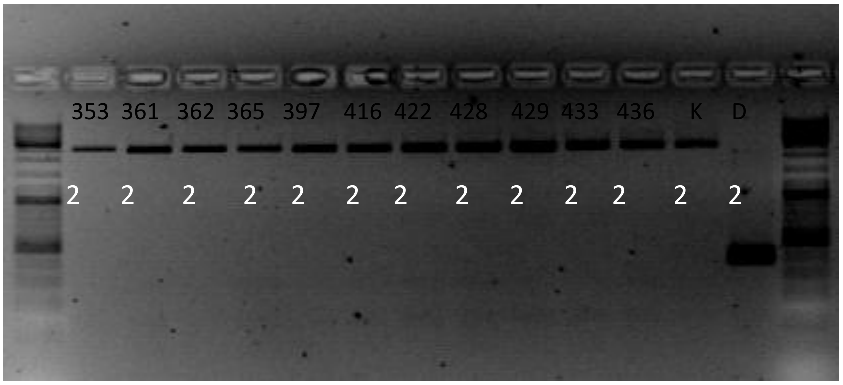
353 361 362 365 397 416 422 428 429 433 436 K D
2 2 2 2 2 2 2 2 2 2 2 2 2

PR_60a

PRFTc1F1 TGGCATTGTAGATTAGGTCATGT

PRFTc1_R2b CACTTATAACCCTTGTGAGTTGCAG

Plate 1

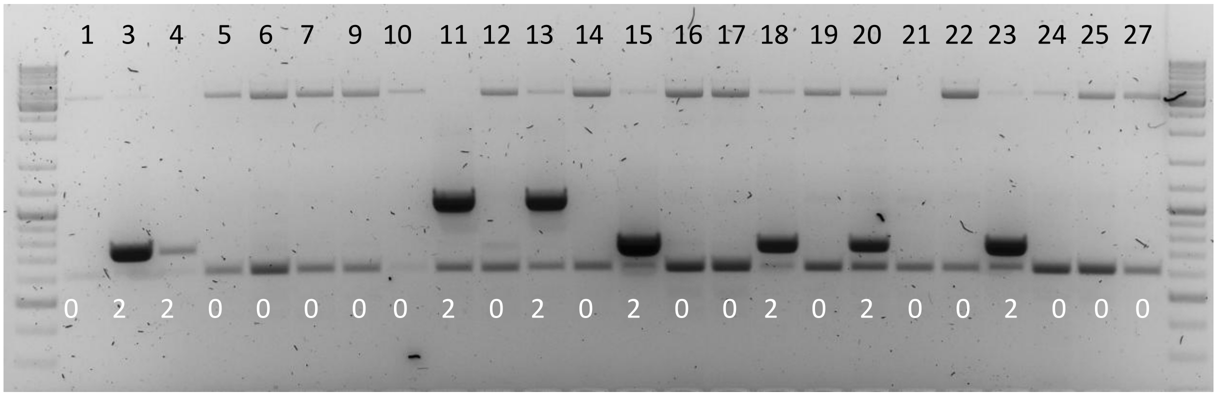

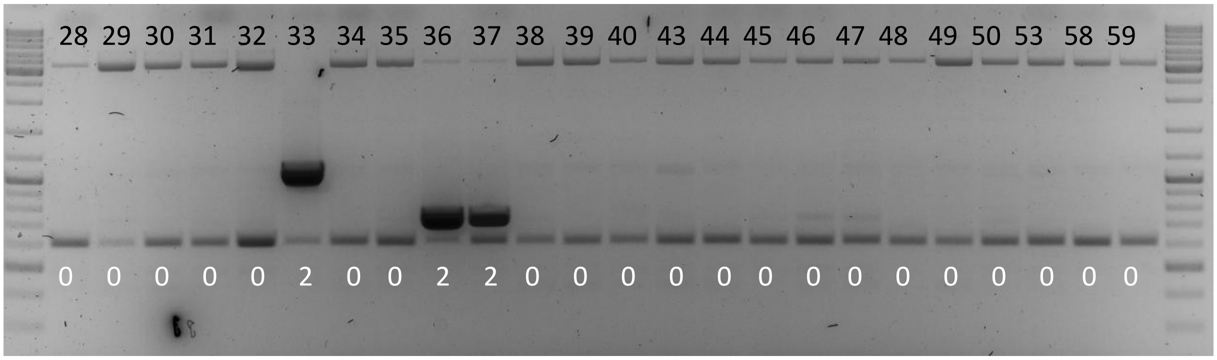

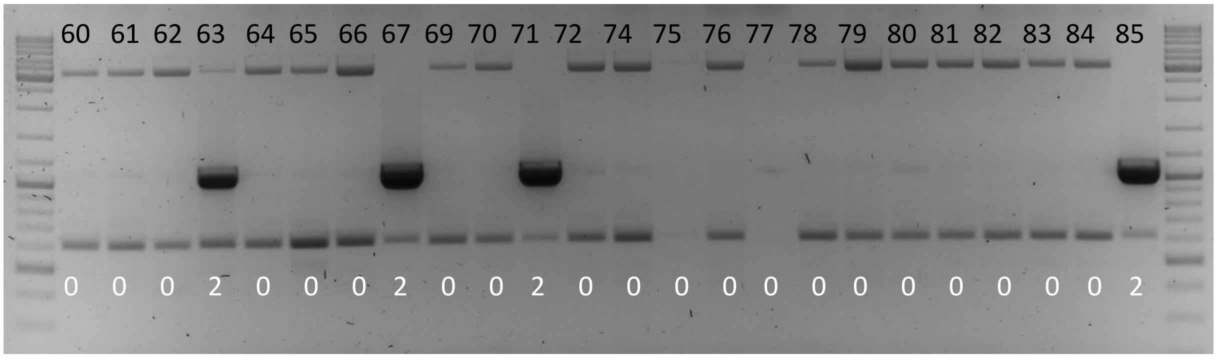

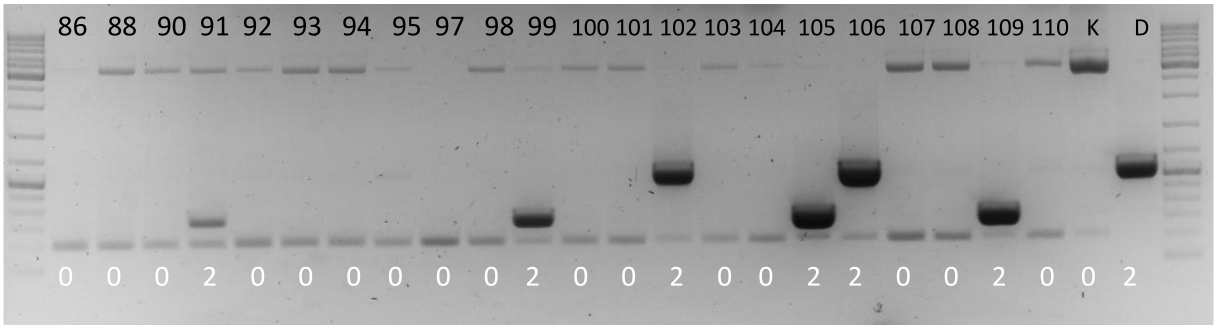

Plate 9

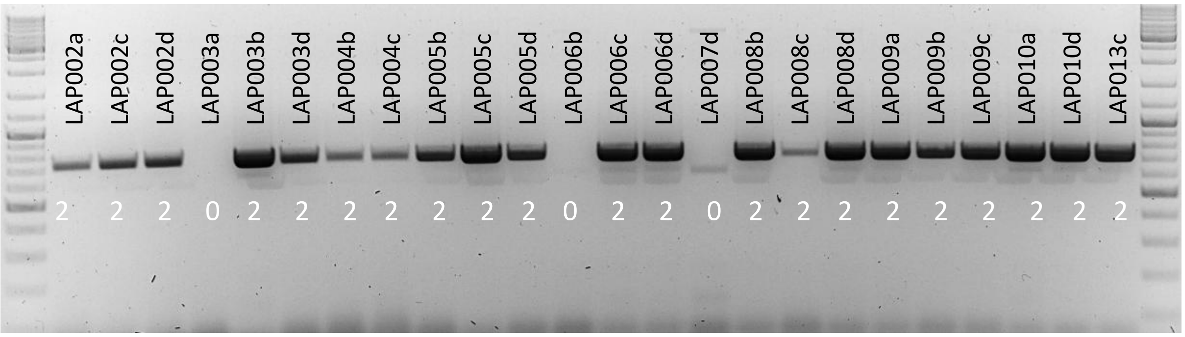
LAP002a LAP002c LAP002d LAP003a LAP003b LAP003d LAP004b LAP004c LAP005b LAP005c LAP005d LAP006b LAP006c LAP006d LAP007d LAP008b LAP008c LAP008d LAP009a LAP009b LAP009c LAP010a LAP010d LAP013c
2 2 2 0 2 2 2 2 2 2 2 0 2 2 0 2 2 2 2 2 2 2 2 2

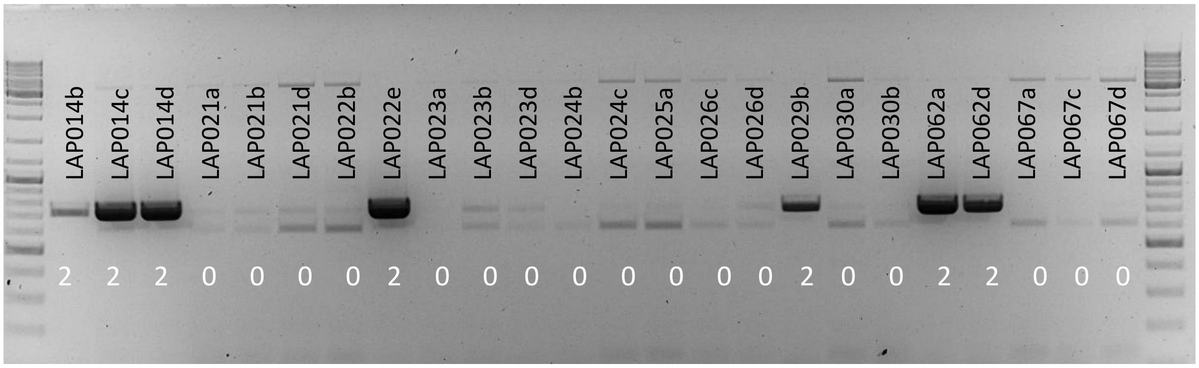
LAP014b LAP014c LAP014d LAP021a LAP021b LAP021d LAP022b LAP022e LAP023a LAP023b LAP023d LAP024b LAP024c LAP025a LAP026c LAP026d LAP029b LAP030a LAP030b LAP062a LAP062d LAP067a LAP067c LAP067d
2 2 2 0 0 0 0 2 0 0 0 0 0 0 0 0 2 0 0 2 2 0 0 0

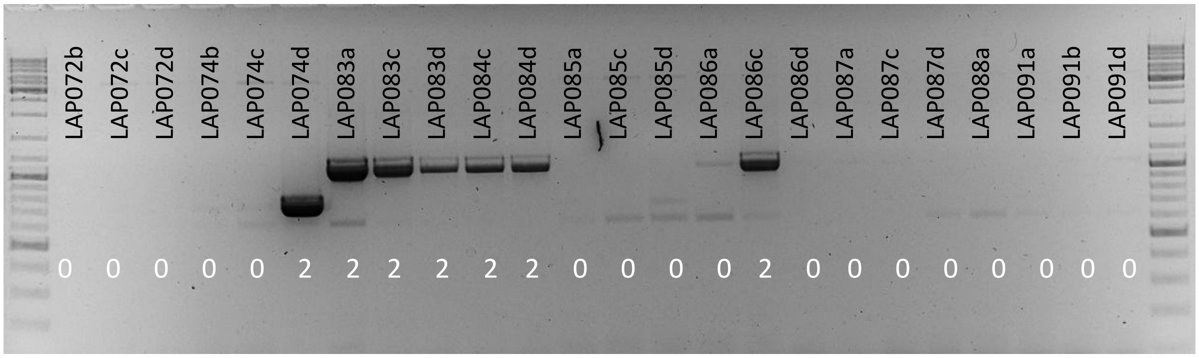
LAP072b LAP072c LAP072d LAP074b LAP074c LAP074d LAP083a LAP083c LAP083d LAP084c LAP084d LAP085a LAP085c LAP085d LAP086a LAP086c LAP086d LAP087a LAP087c LAP087d LAP088a LAP091a LAP091b LAP091d
0 0 0 0 0 2 2 2 2 2 2 0 0 0 0 2 0 0 0 0 0 0 0 0

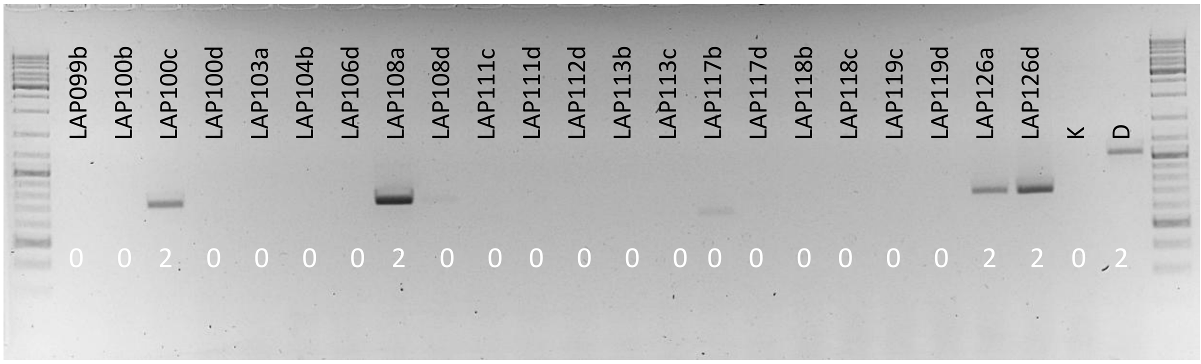
LAP099b LAP100b LAP100c LAP100d LAP103a LAP104b LAP106d LAP108a LAP108d LAP111c LAP111d LAP112d LAP113b LAP113c LAP117b LAP117d LAP118b LAP118c LAP119c LAP119d LAP126a LAP126d K D
0 0 2 0 0 0 0 2 0 0 0 0 0 0 0 0 0 0 0 0 2 2 0 2

PR_60b

PRFTc1F1 TGGCATTGTAGATTAGGTCATGT

PRFTc1_R2b CACTTATAACCCTTGTGAGTTGCAG

Plate 1

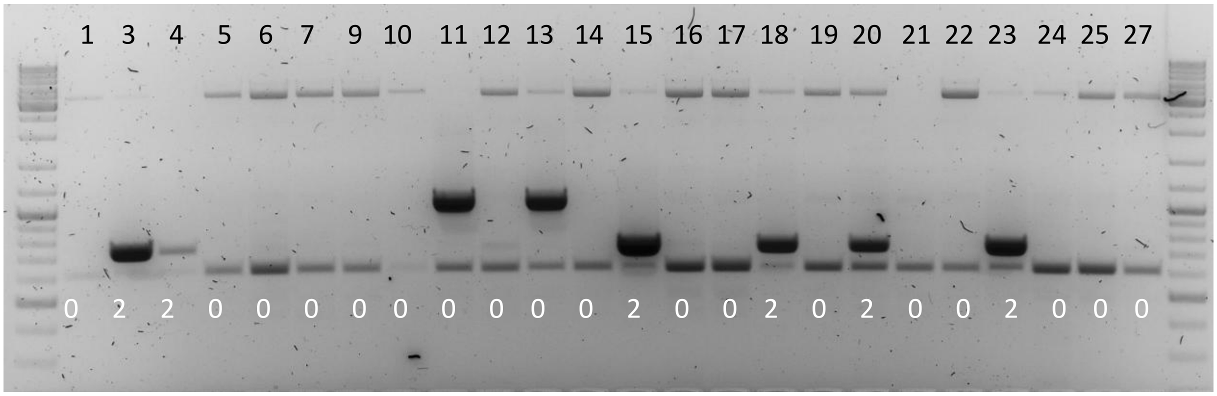

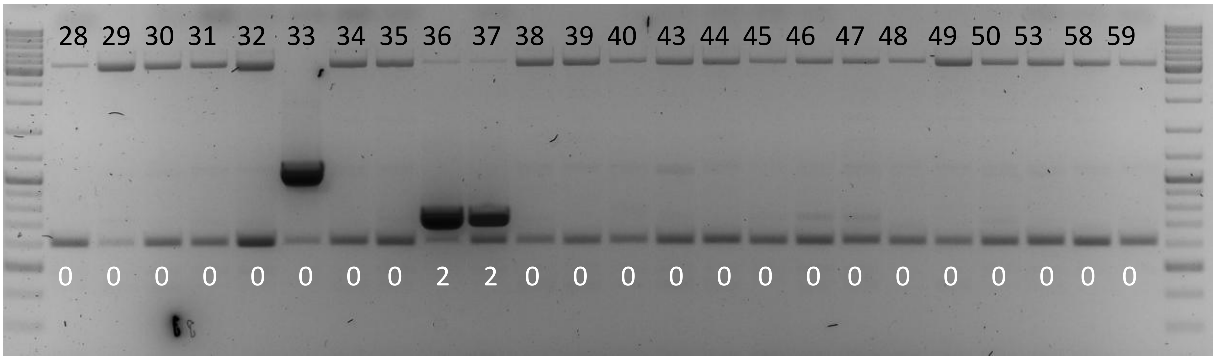

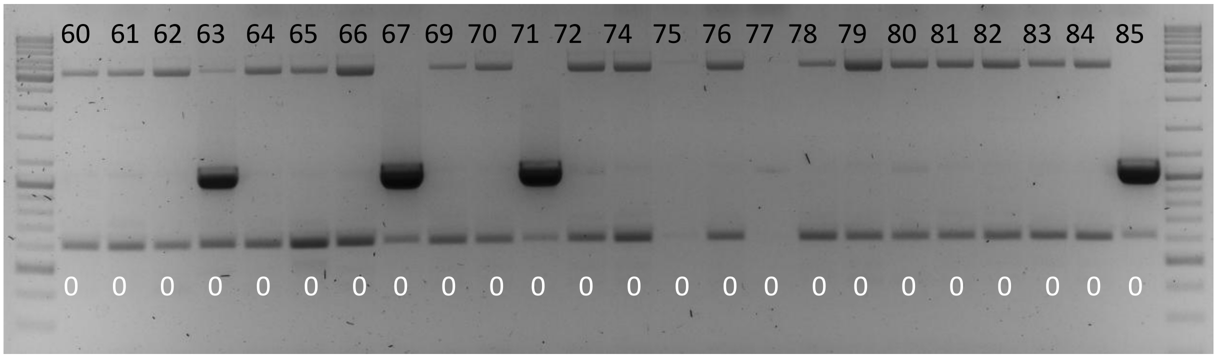

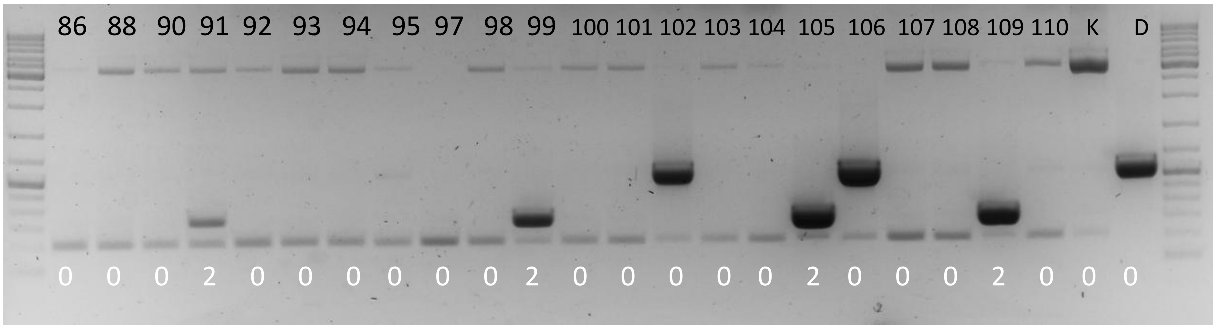

Plate 9

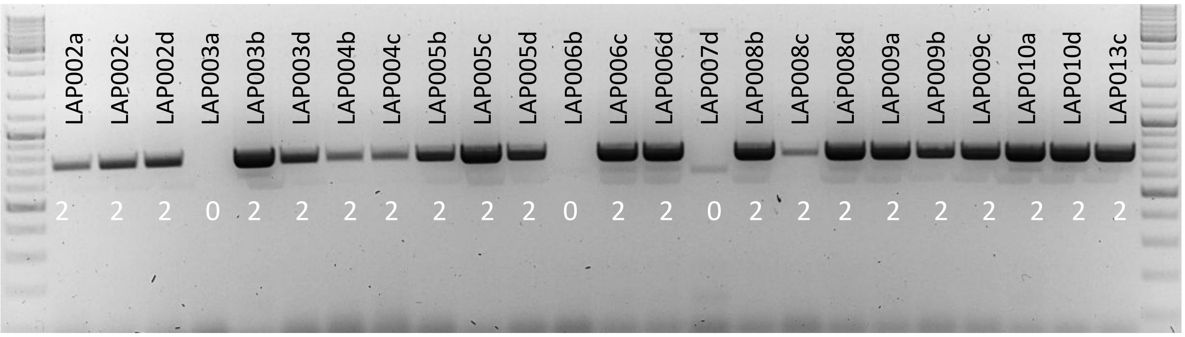
LAP002a LAP002c LAP002d LAP003a LAP003b LAP003d LAP004b LAP004c LAP005b LAP005c LAP005d LAP006b LAP006c LAP006d LAP007d LAP008b LAP008c LAP008d LAP009a LAP009b LAP009c LAP010a LAP010d LAP013c
2 2 2 0 2 2 2 2 2 2 2 0 2 2 0 2 2 2 2 2 2 2 2 2

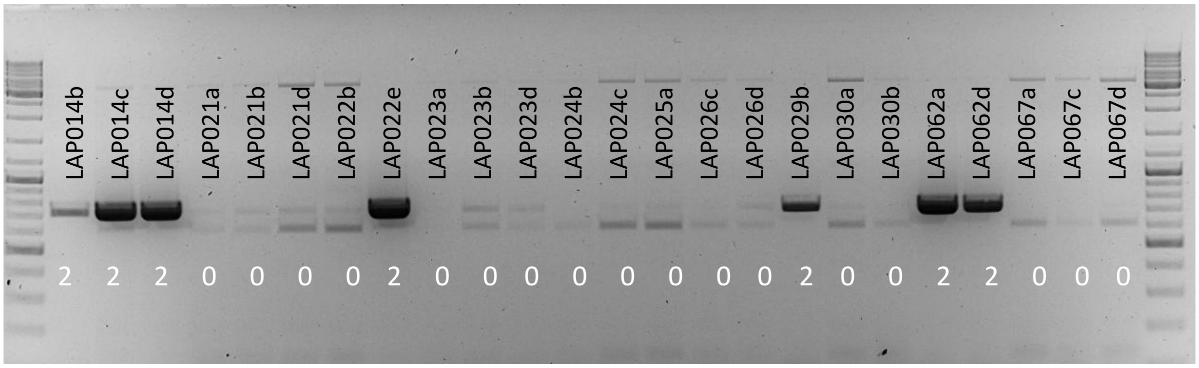
LAP014b LAP014c LAP014d LAP021a LAP021b LAP021d LAP022b LAP022e LAP023a LAP023b LAP023d LAP024b LAP024c LAP025a LAP026c LAP026d LAP029b LAP030a LAP030b LAP062a LAP062d LAP067a LAP067c LAP067d
2 2 2 0 0 0 0 2 0 0 0 0 0 0 0 0 2 0 0 2 2 0 0 0

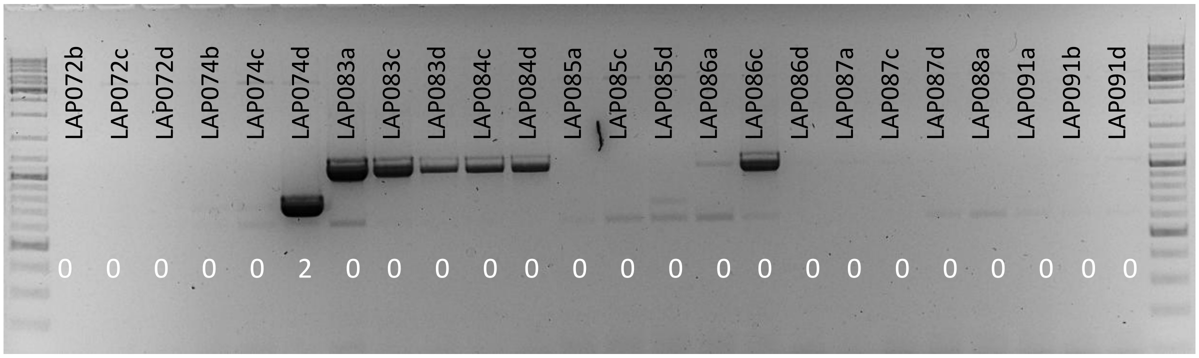
LAP072b LAP072c LAP072d LAP074b LAP074c LAP074d LAP083a LAP083c LAP083d LAP084c LAP084d LAP085a LAP085c LAP085d LAP086a LAP086c LAP086d LAP087a LAP087c LAP087d LAP088a LAP091a LAP091b LAP091d
0 0 0 0 0 2 0 0 0 0 0 0 0 0 0 0 0 0 0 0 0 0 0 0

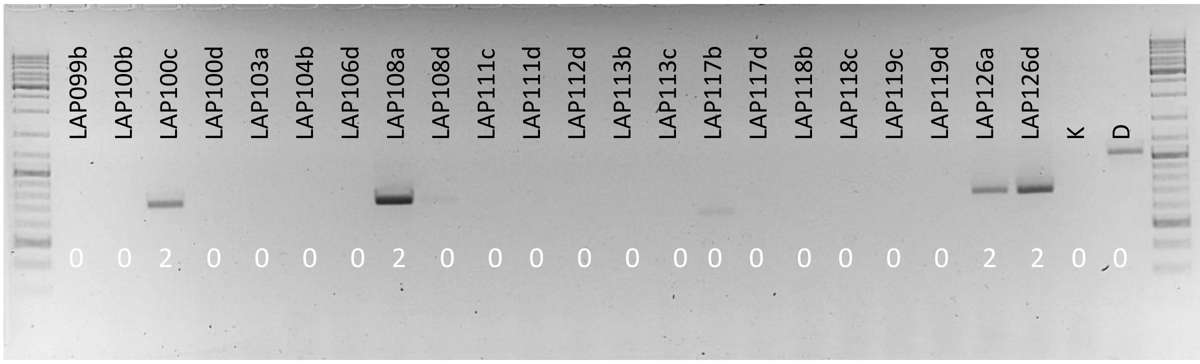
LAP099b LAP100b LAP100c LAP100d LAP103a LAP104b LAP106d LAP108a LAP108d LAP111c LAP111d LAP112d LAP113b LAP113c LAP117b LAP117d LAP118b LAP118c LAP119c LAP119d LAP126a LAP126d K D
0 0 2 0 0 0 0 2 0 0 0 0 0 0 0 0 0 0 0 0 2 2 0 0

PR_60c

PRFTc1F1 TGGCATTGTAGATTAGGTCATGT

PRFTc1_R2b CACTTATAACCCTTGTGAGTTGCAG

Plate 1

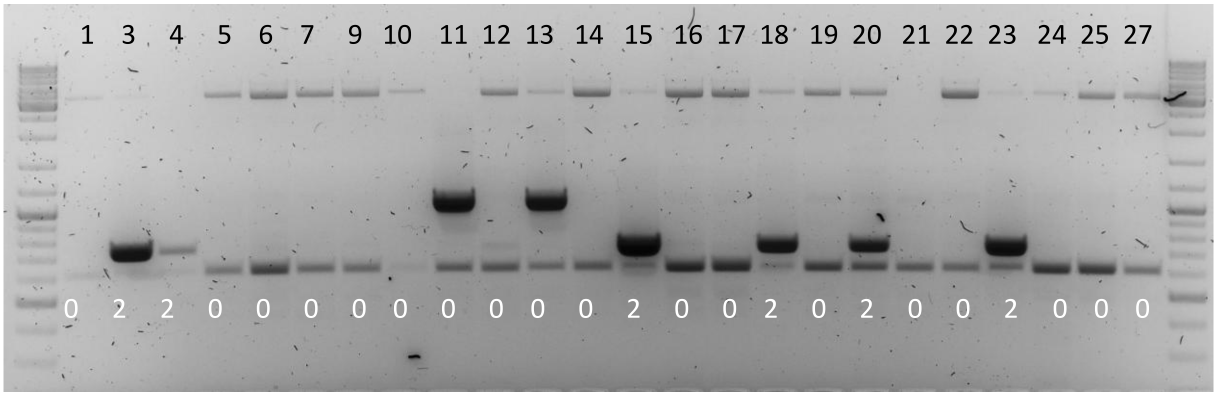

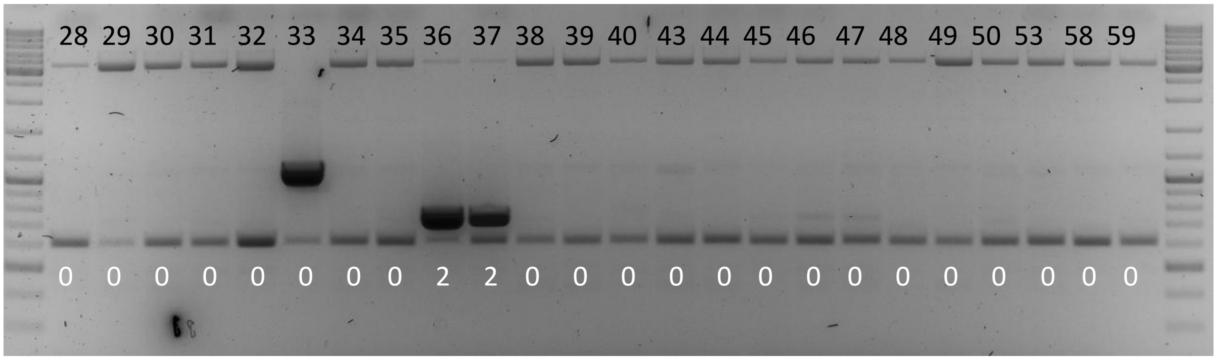

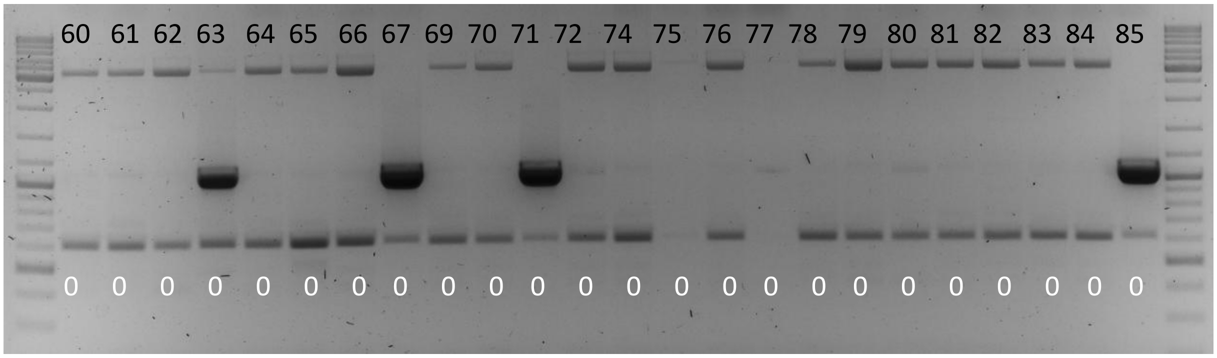

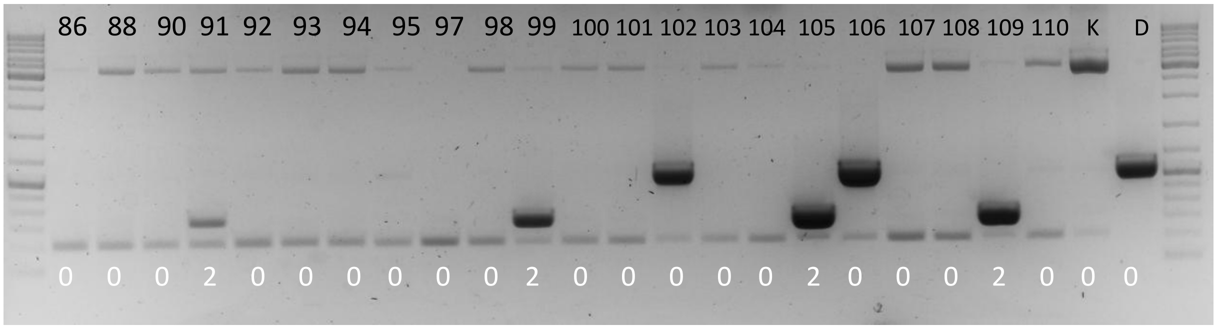

Plate 9

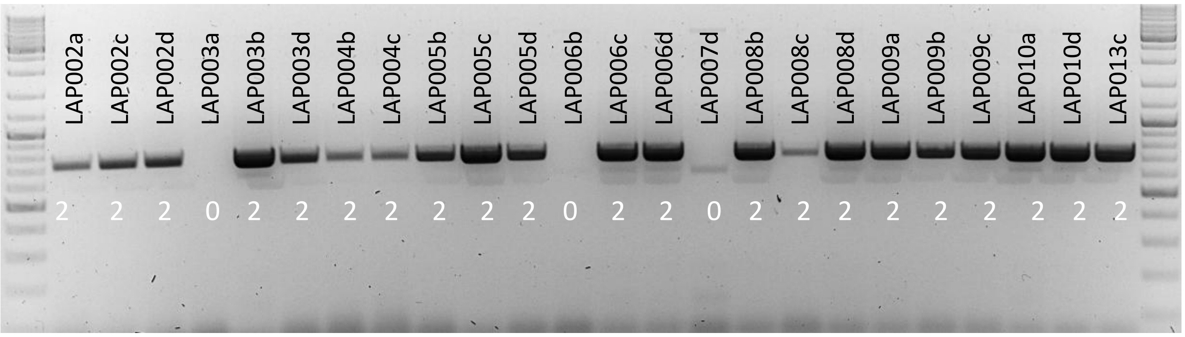
LAP002a
LAP002c
LAP002d
LAP003a
LAP003b
LAP003d
LAP004b
LAP004c
LAP005b
LAP005c
LAP005d
LAP006b
LAP006c
LAP006d
LAP007d
LAP008b
LAP008c
LAP008d
LAP009a
LAP009b
LAP009c
LAP010a
LAP010d
LAP013c
2 2 2 0 2 2 2 2 2 2 2 0 2 2 0 2 2 2 2 2 2 2 2 2

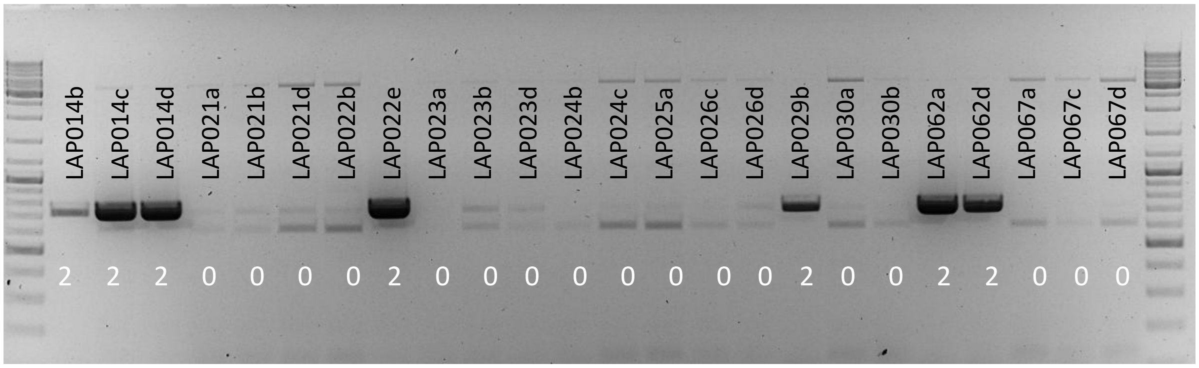
LAP014b
LAP014c
LAP014d
LAP021a
LAP021b
LAP021d
LAP022b
LAP022e
LAP023a
LAP023b
LAP023d
LAP024b
LAP024c
LAP025a
LAP026c
LAP026d
LAP029b
LAP030a
LAP030b
LAP062a
LAP062d
LAP067a
LAP067c
LAP067d
2 2 2 0 0 0 0 2 0 0 0 0 0 0 0 0 2 0 0 2 2 0 0 0

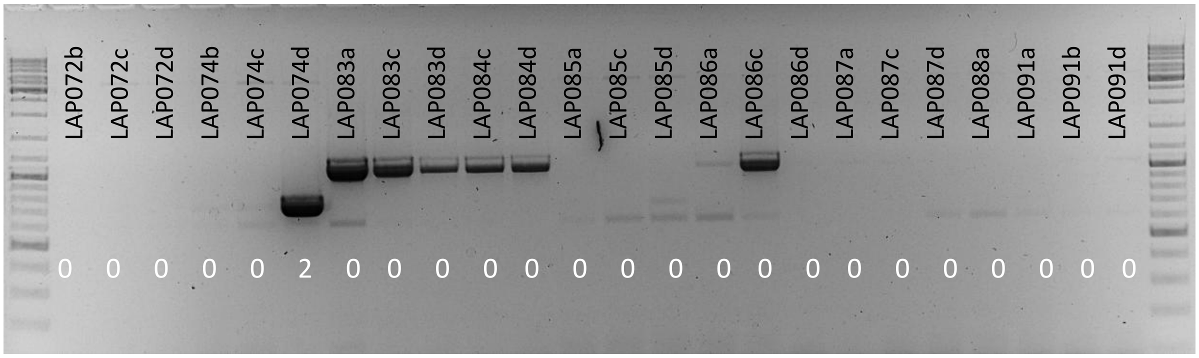
LAP072b
LAP072c
LAP072d
LAP074b
LAP074c
LAP074d
LAP083a
LAP083c
LAP083d
LAP084c
LAP084d
LAP085a
LAP085c
LAP085d
LAP086a
LAP086c
LAP086d
LAP087a
LAP087c
LAP087d
LAP088a
LAP091a
LAP091b
LAP091d
0 0 0 0 0 2 0 0 0 0 0 0 0 0 0 0 0 0 0 0 0 0 0 0

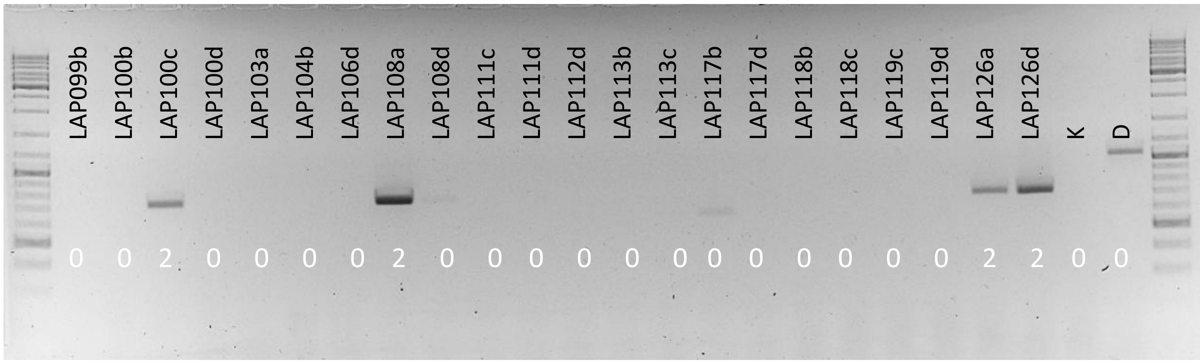
LAP099b
LAP100b
LAP100c
LAP100d
LAP103a
LAP104b
LAP106d
LAP108a
LAP108d
LAP111c
LAP111d
LAP112d
LAP113b
LAP113c
LAP117b
LAP117d
LAP118b
LAP118c
LAP119c
LAP119d
LAP126a
LAP126d
K
D
0 0 2 0 0 0 0 2 0 0 0 0 0 0 0 0 0 0 0 0 2 2 0 0

PR_61

PRFTc1_F2b ACAAATTGGAACTCAGATTAGCAGA

PRFTc1_R3c AGAATCCAACTCCAAGCTCCAATAT

Plate 1

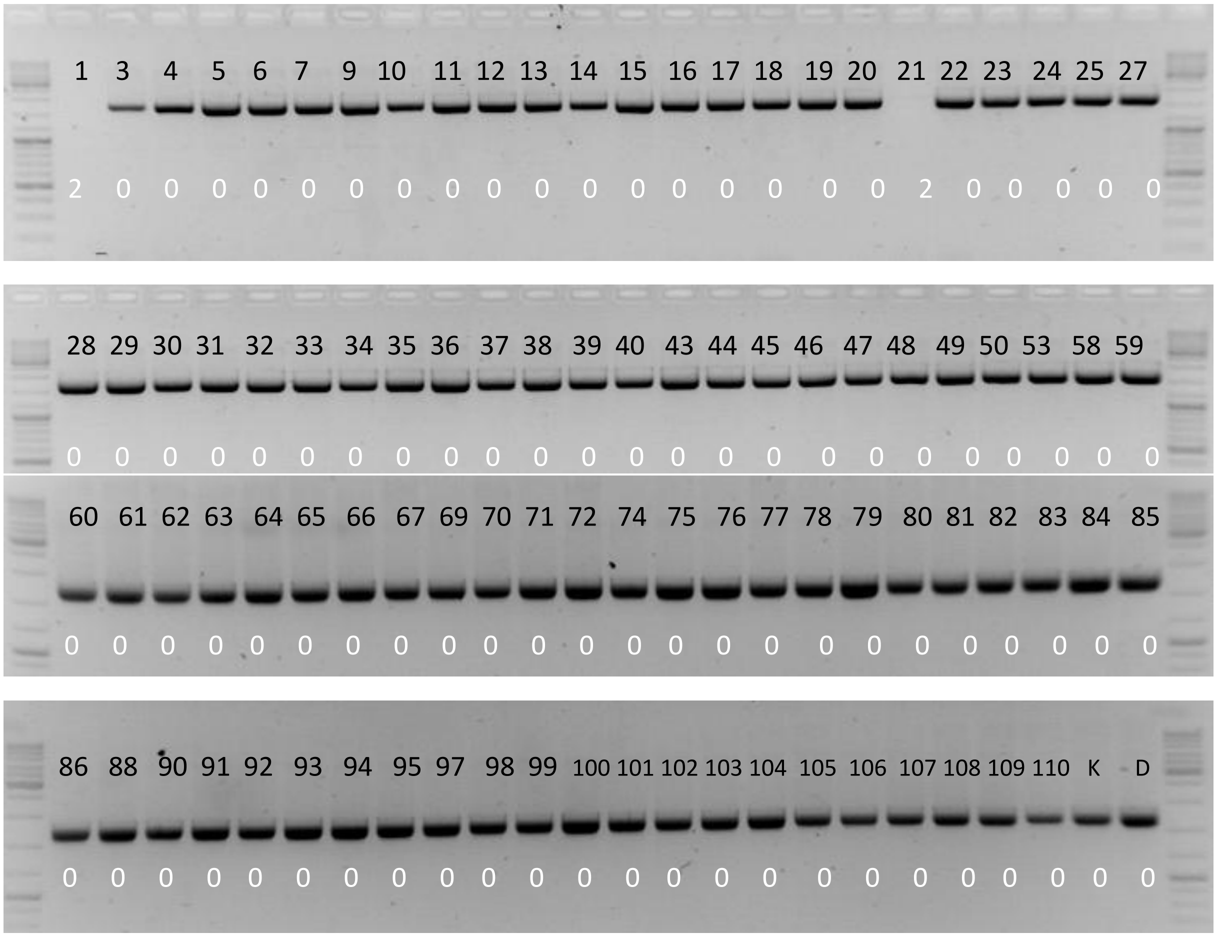

Plate 9

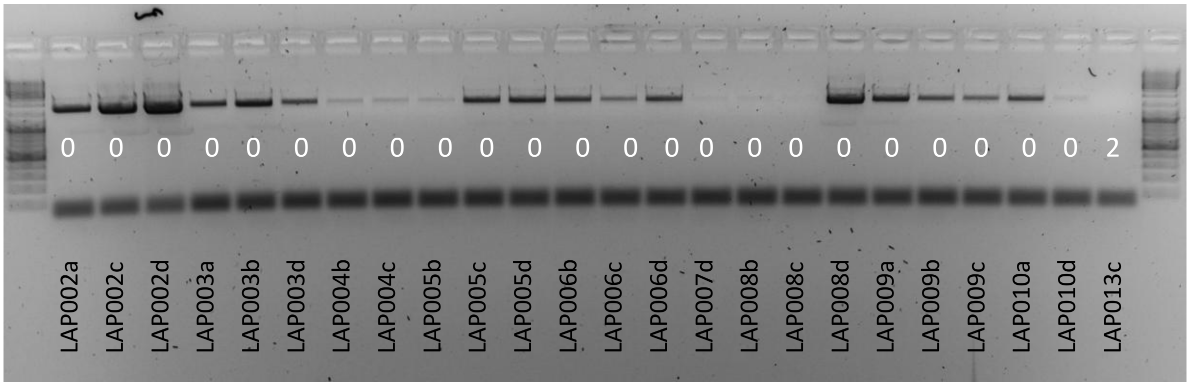
0 0 0 0 0 0 0 0 0 0 0 0 0 0 0 0 0 0 0 0 0 0 0 2
LAP002a LAP002c LAP002d LAP003a LAP003b LAP003d LAP004b LAP004c LAP005b LAP005c LAP005d LAP006b LAP006c LAP006d LAP007d LAP008b LAP008c LAP008d LAP009a LAP009b LAP009c LAP010a LAP010d LAP013c

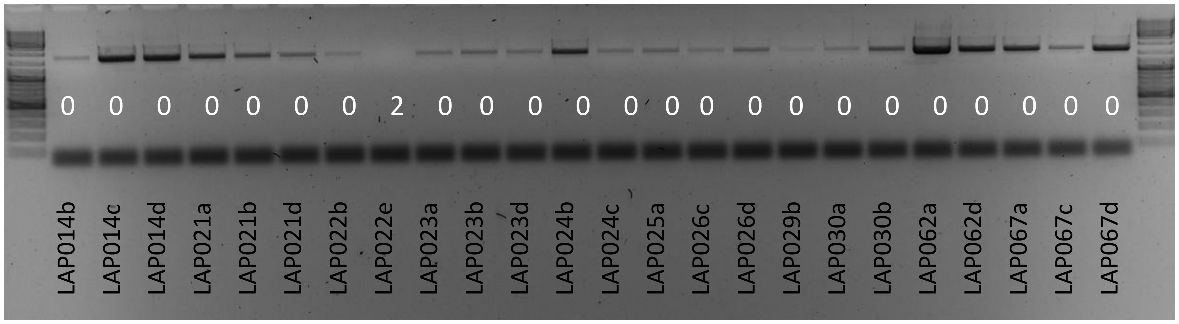
0 0 0 0 0 0 0 2 0 0 0 0 0 0 0 0 0 0 0 0 0 0 0 0
LAP014b LAP014c LAP014d LAP021a LAP021b LAP021d LAP022b LAP022e LAP023a LAP023b LAP023d LAP024b LAP024c LAP025a LAP026c LAP026d LAP029b LAP030a LAP030b LAP062a LAP062d LAP067a LAP067c LAP067d

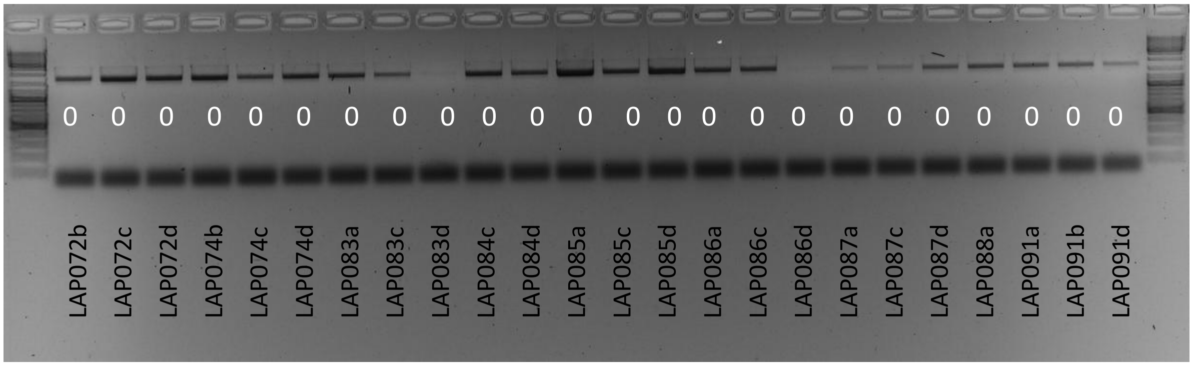
0 0 0 0 0 0 0 0 0 0 0 0 0 0 0 0 0 0 0 0 0 0 0 0
LAP072b LAP072c LAP072d LAP074b LAP074c LAP074d LAP083a LAP083c LAP083d LAP084c LAP084d LAP085a LAP085c LAP085d LAP086a LAP086c LAP086d LAP087a LAP087c LAP087d LAP088a LAP091a LAP091b LAP091d

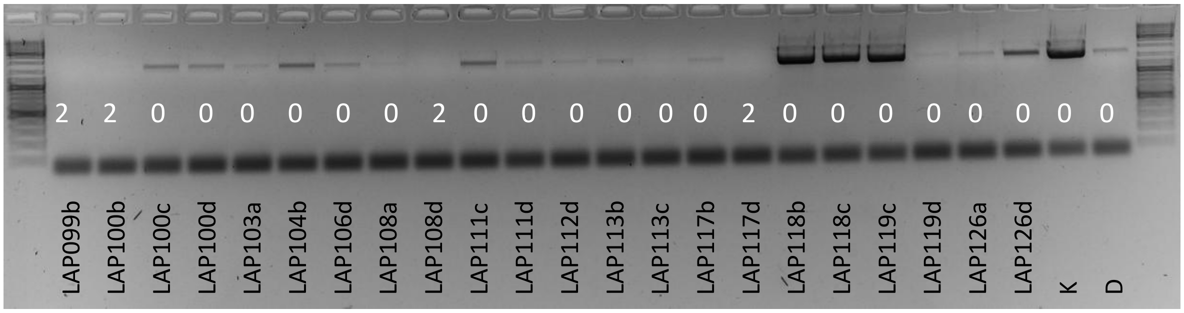
2 2 0 0 0 0 0 0 2 0 0 0 0 0 0 2 0 0 0 0 0 0 0 0
LAP099b LAP100b LAP100c LAP100d LAP103a LAP104b LAP106d LAP108a LAP108d LAP111c LAP111d LAP112d LAP113b LAP113c LAP117b LAP117d LAP118b LAP118c LAP119c LAP119d LAP126a LAP126d K D

PR_62

PRFTC1F4 ATAAGATTGAATCCCTCTCTCGTCT

PRFTC1R4 GGAAACAATGCAACAGTTGAATGA

PRFTc1_F4a AGTGCAAATAACTTACTCCAGATCA

PRFTc1_R4b ACAACAAACACATAAATCTATACTGGA

Plate 1

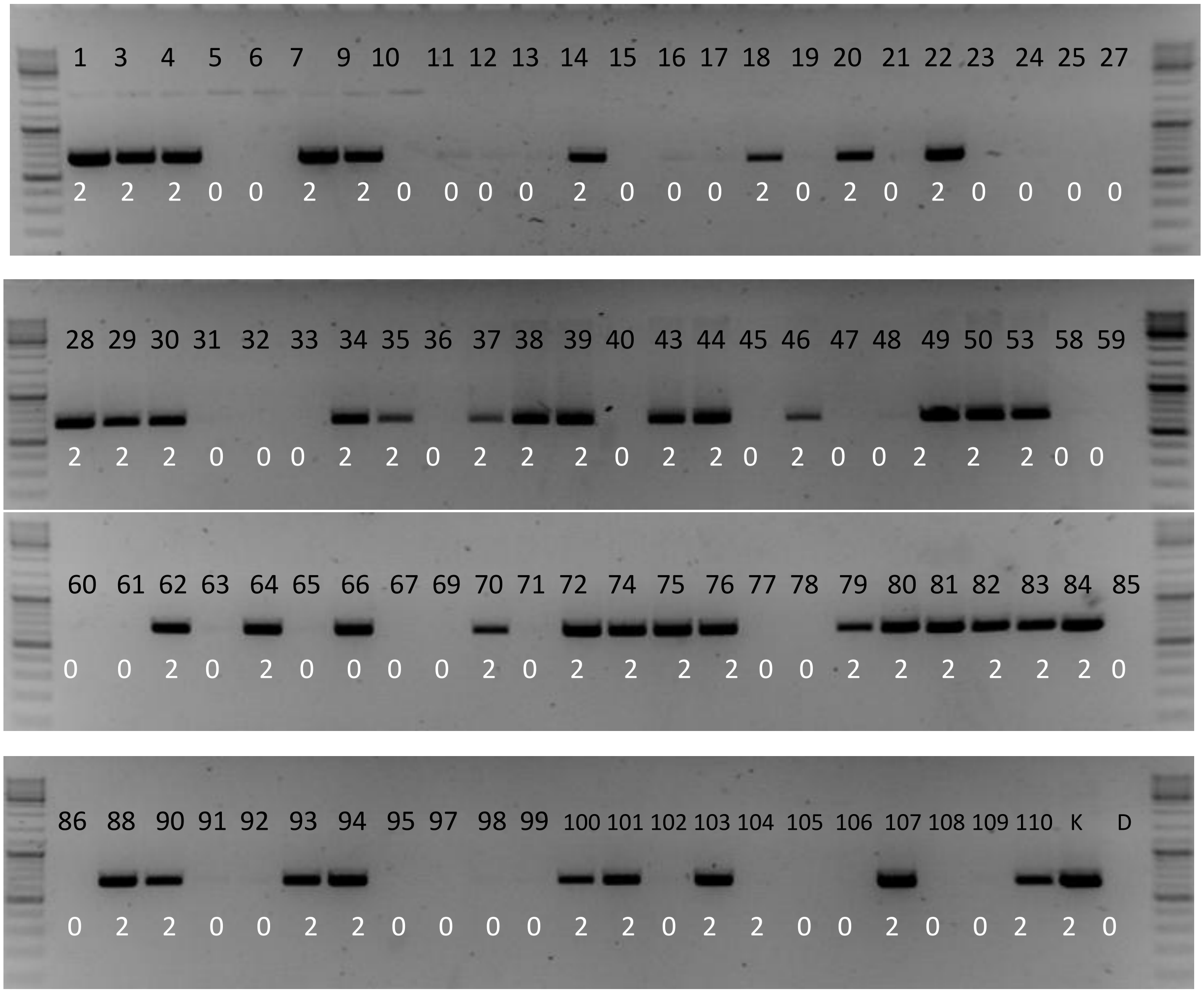

Plate 9

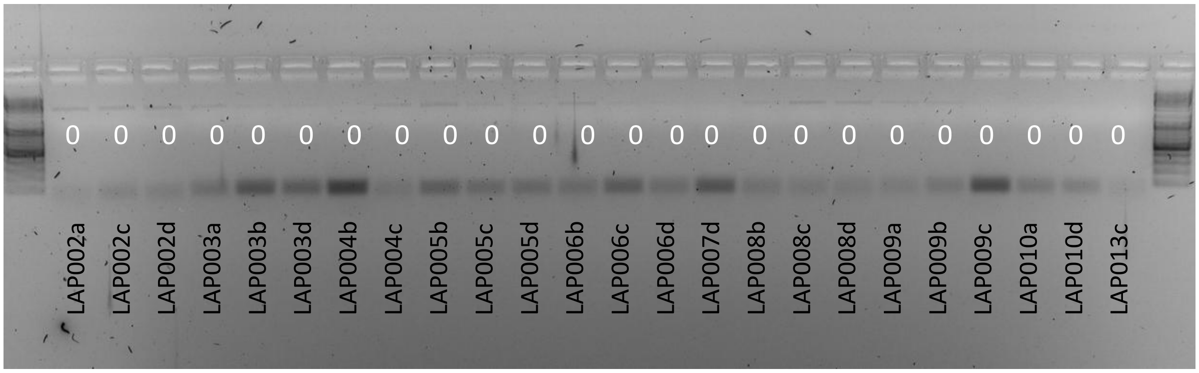
0 0 0 0 0 0 0 0 0 0 0 0 0 0 0 0 0 0 0 0 0 0 0 0
LAP002a
LAP002c
LAP002d
LAP003a
LAP003b
LAP003d
LAP004b
LAP004c
LAP005b
LAP005c
LAP005d
LAP006b
LAP006c
LAP006d
LAP007d
LAP008b
LAP008c
LAP008d
LAP009a
LAP009b
LAP009c
LAP010a
LAP010d
LAP013c

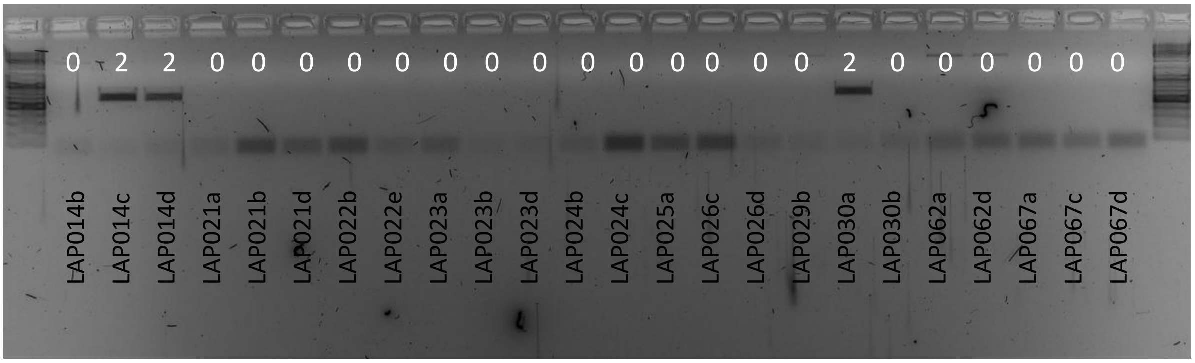
0 2 2 0 0 0 0 0 0 0 0 0 0 0 0 0 0 2 0 0 0 0 0 0
LAP014b
LAP014c
LAP014d
LAP021a
LAP021b
LAP021d
LAP022b
LAP022e
LAP023a
LAP023b
LAP023d
LAP024b
LAP024c
LAP025a
LAP026c
LAP026d
LAP029b
LAP030a
LAP030b
LAP062a
LAP062d
LAP067a
LAP067c
LAP067d

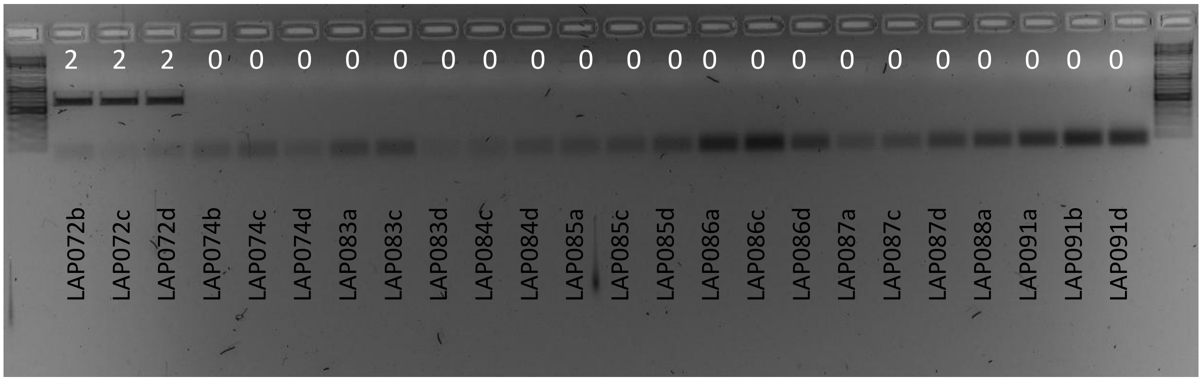
2 2 2 0 0 0 0 0 0 0 0 0 0 0 0 0 0 0 0 0 0 0 0 0
LAP072b
LAP072c
LAP072d
LAP074b
LAP074c
LAP074d
LAP083a
LAP083c
LAP083d
LAP084c
LAP084d
LAP085a
LAP085c
LAP085d
LAP086a
LAP086c
LAP086d
LAP087a
LAP087c
LAP087d
LAP088a
LAP091a
LAP091b
LAP091d

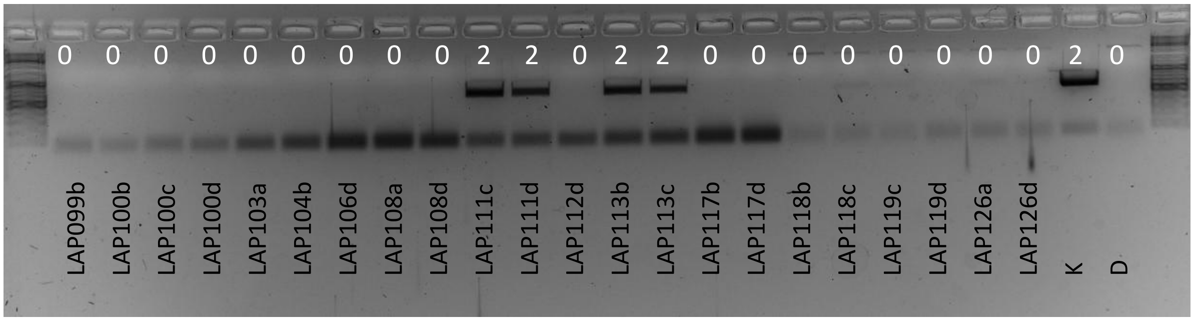
0 0 0 0 0 0 0 0 0 2 2 0 2 2 0 0 0 0 0 0 0 0 2 0
LAP099b
LAP100b
LAP100c
LAP100d
LAP103a
LAP104b
LAP106d
LAP108a
LAP108d
LAP111c
LAP111d
LAP112d
LAP113b
LAP113c
LAP117b
LAP117d
LAP118b
LAP118c
LAP119c
LAP119d
LAP126a
LAP126d
K
D

PR_66

PRFTc1_F1b AGTCGTCAATTAAGATCTCAGCTCA

PRFTC1R2 AGTAACCAGTGACCATGTGCC

Plate 1

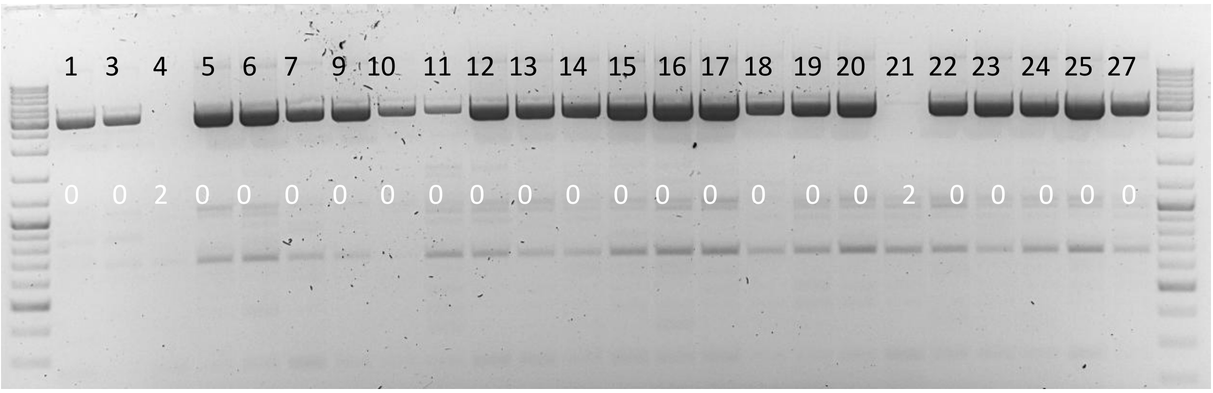

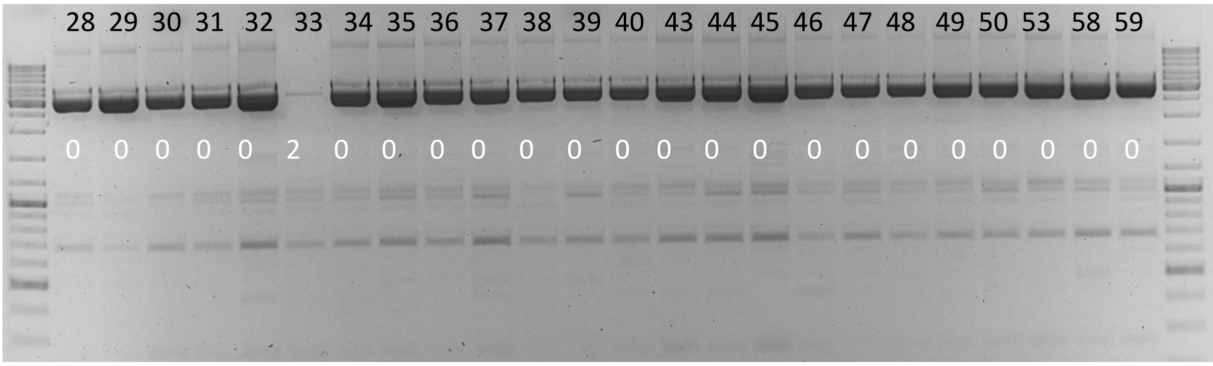

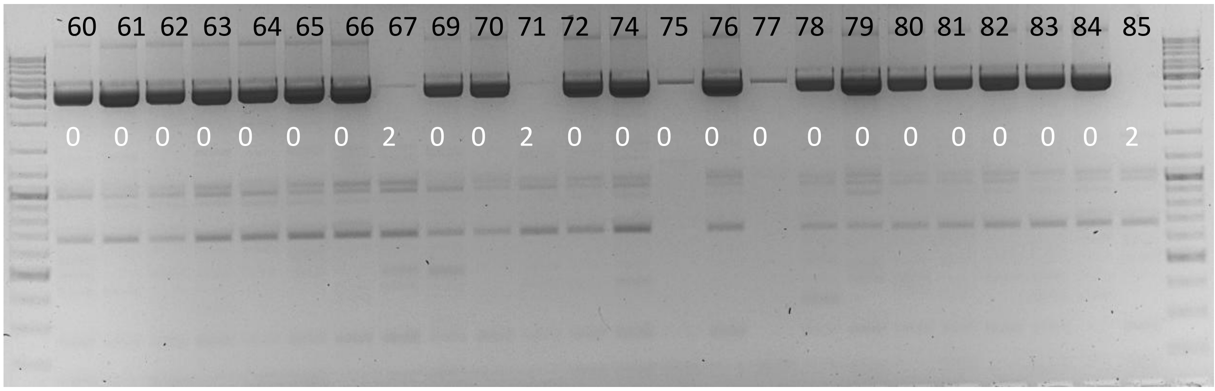

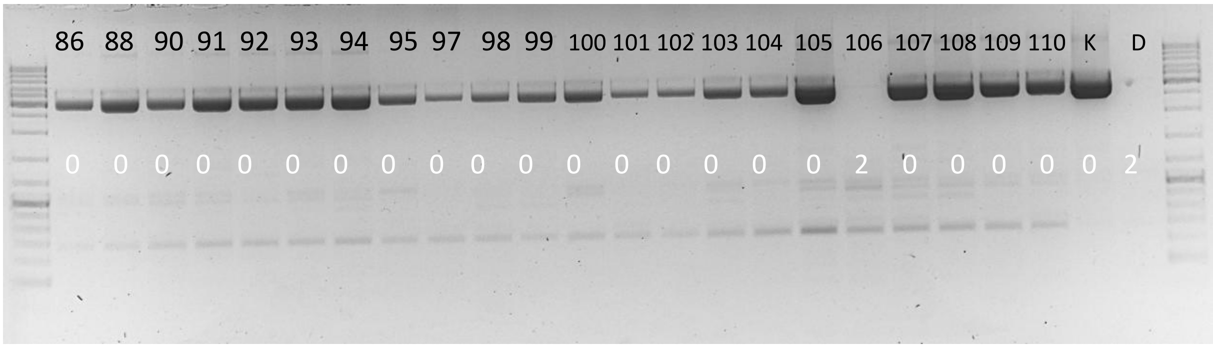

Plate 9

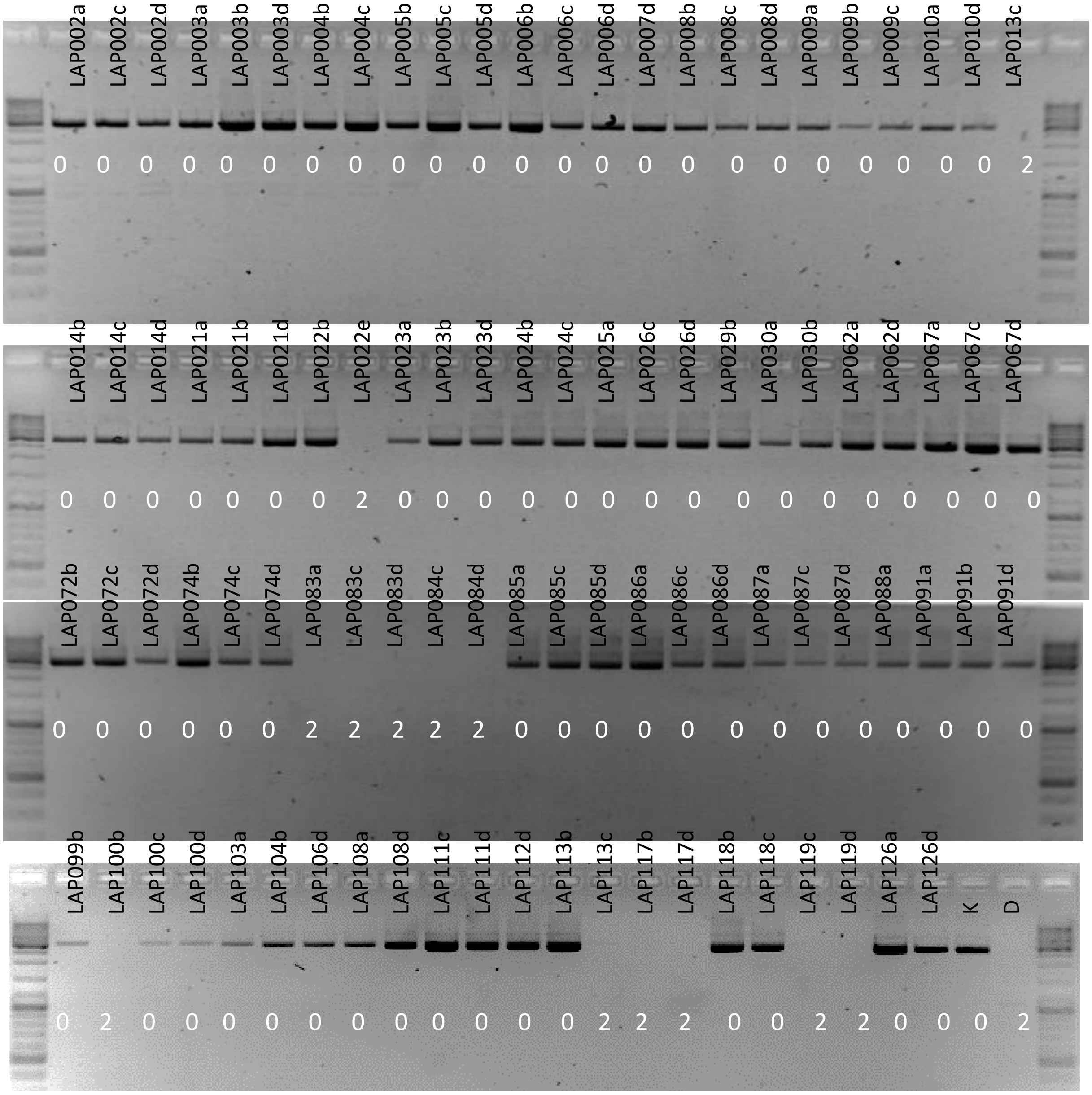
LAP002a LAP002c LAP002d LAP003a LAP003b LAP003d LAP004b LAP004c LAP005b LAP005c LAP005d LAP006b LAP006c LAP006d LAP007d LAP008b LAP008c LAP008d LAP009a LAP009b LAP009c LAP010a LAP010d LAP013c
0 0 0 0 0 0 0 0 0 0 0 0 0 0 0 0 0 0 0 0 0 0 0 2
LAP014b LAP014c LAP014d LAP021a LAP021b LAP021d LAP022b LAP022e LAP023a LAP023b LAP023d LAP024b LAP024c LAP025a LAP026c LAP026d LAP029b LAP030a LAP030b LAP062a LAP062d LAP067a LAP067c LAP067d
0 0 0 0 0 0 0 2 0 0 0 0 0 0 0 0 0 0 0 0 0 0 0 0
LAP072b LAP072c LAP072d LAP074b LAP074c LAP074d LAP083a LAP083c LAP083d LAP084c LAP084d LAP085a LAP085c LAP085d LAP086a LAP086c LAP086d LAP087a LAP087c LAP087d LAP088a LAP091a LAP091b LAP091d
0 0 0 0 0 0 2 2 2 2 2 0 0 0 0 0 0 0 0 0 0 0 0 0
LAP099b LAP100b LAP100c LAP100d LAP103a LAP104b LAP106d LAP108a LAP108d LAP111c LAP111d LAP112d LAP113b LAP113c LAP117b LAP117d LAP118b LAP118c LAP119c LAP119d LAP126a LAP126d K D
0 2 0 0 0 0 0 0 0 0 0 0 0 2 2 2 0 0 2 2 0 0 0 2

PR_67

PRFTc1_F3c GCAGATTGAGCCTATGATCCAAATG

PRFTc1_R5b AGAAGCTTAATGAACTGAAGCAATT

Plate 1

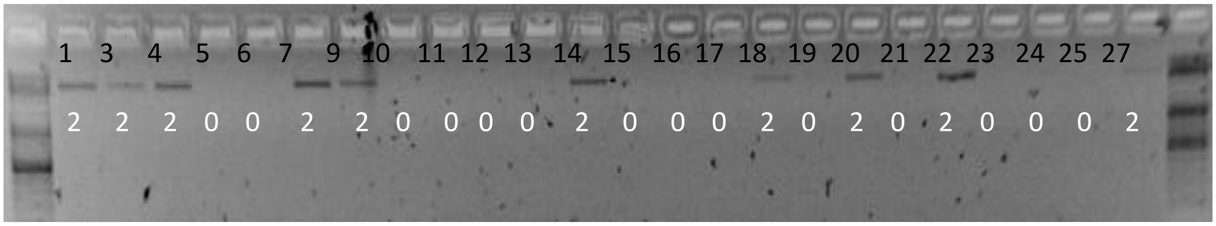

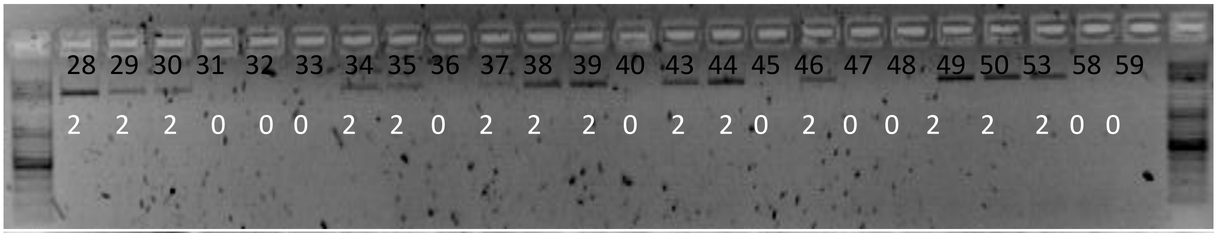

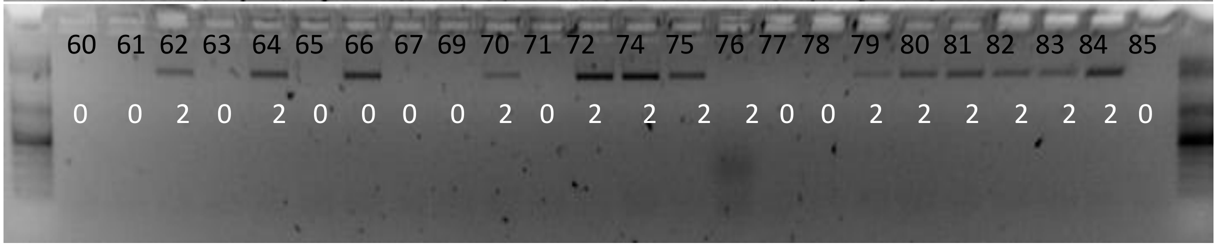

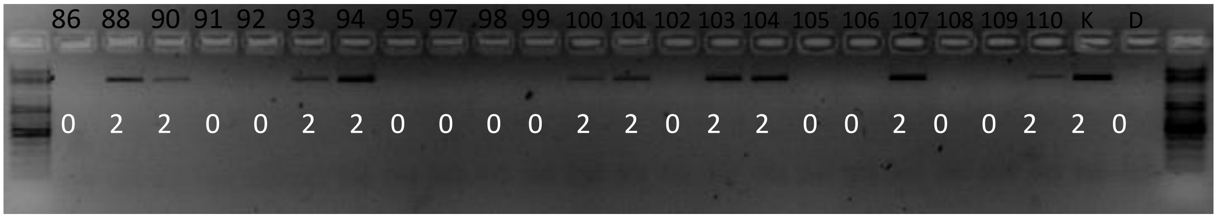

Plate 9

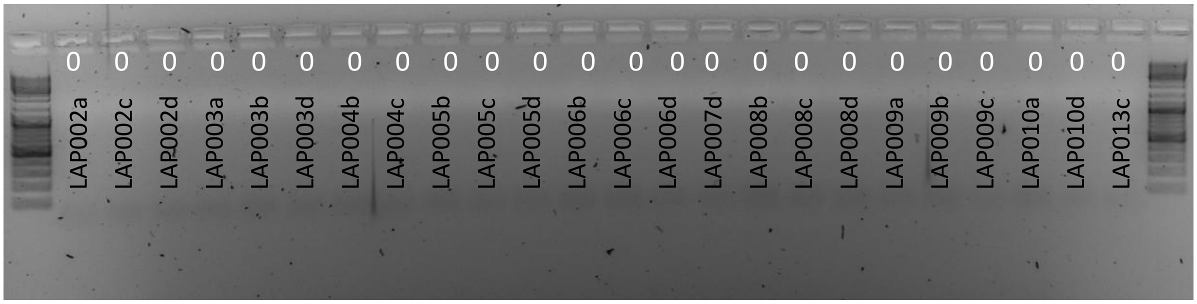
0 0 0 0 0 0 0 0 0 0 0 0 0 0 0 0 0 0 0 0 0 0 0 0
LAP002a LAP002c LAP002d LAP003a LAP003b LAP003d LAP004b LAP004c LAP005b LAP005c LAP005d LAP006b LAP006c LAP006d LAP007d LAP008b LAP008c LAP008d LAP009a LAP009b LAP009c LAP010a LAP010d LAP013c

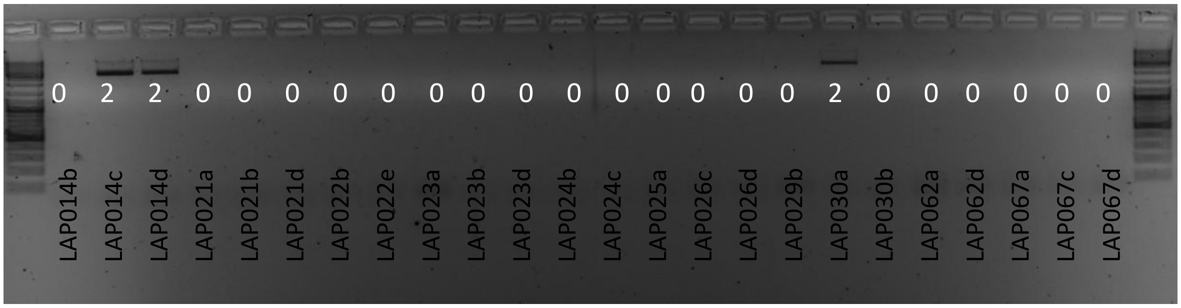
0 2 2 0 0 0 0 0 0 0 0 0 0 0 0 0 0 2 0 0 0 0 0 0
LAP014b LAP014c LAP014d LAP021a LAP021b LAP021d LAP022b LAP022e LAP023a LAP023b LAP023d LAP024b LAP024c LAP025a LAP026c LAP026d LAP029b LAP030a LAP030b LAP062a LAP062d LAP067a LAP067c LAP067d

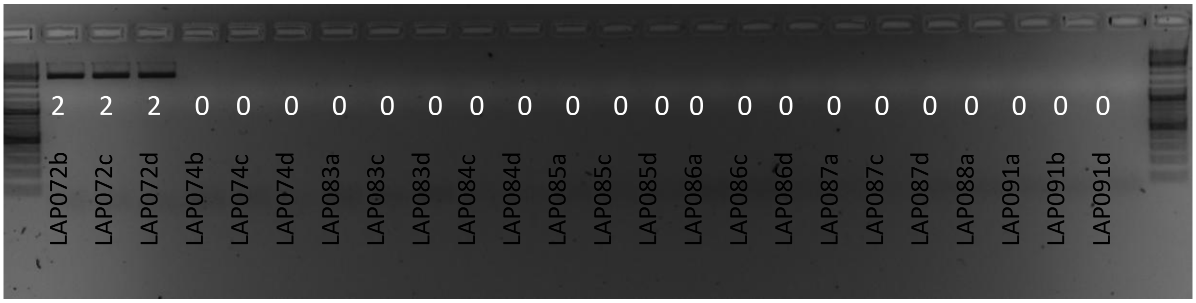
2 2 2 0 0 0 0 0 0 0 0 0 0 0 0 0 0 0 0 0 0 0 0 0
LAP072b LAP072c LAP072d LAP074b LAP074c LAP074d LAP083a LAP083c LAP083d LAP084c LAP084d LAP085a LAP085c LAP085d LAP086a LAP086c LAP086d LAP087a LAP087c LAP087d LAP088a LAP091a LAP091b LAP091d

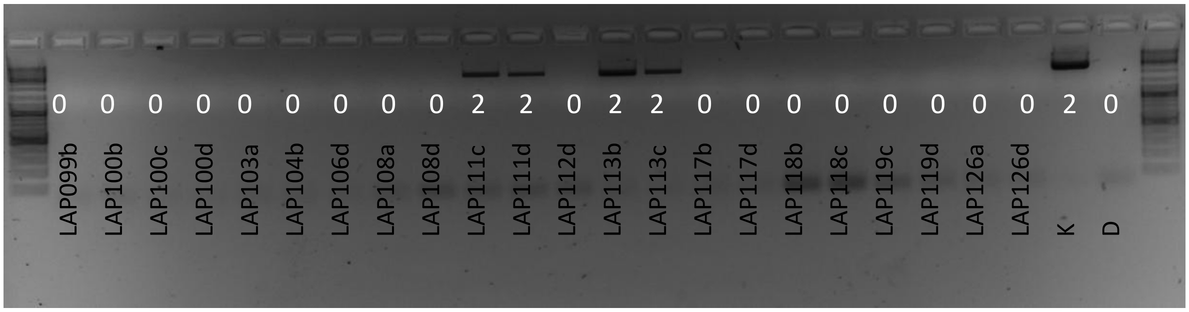
0 0 0 0 0 0 0 0 0 2 2 0 2 2 0 0 0 0 0 0 0 0 2 0
LAP099b LAP100b LAP100c LAP100d LAP103a LAP104b LAP106d LAP108a LAP108d LAP111c LAP111d LAP112d LAP113b LAP113c LAP117b LAP117d LAP118b LAP118c LAP119c LAP119d LAP126a LAP126d K D

PR_70

FTc1_F4c TGTGCAGATTTAACAGCAACTTCTT

FTc1_R4c CTATATCCATCTTAGCTTTAAAACTT

Plate 1

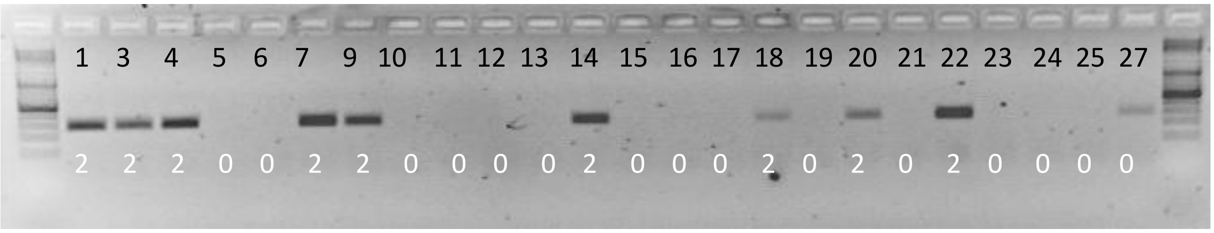

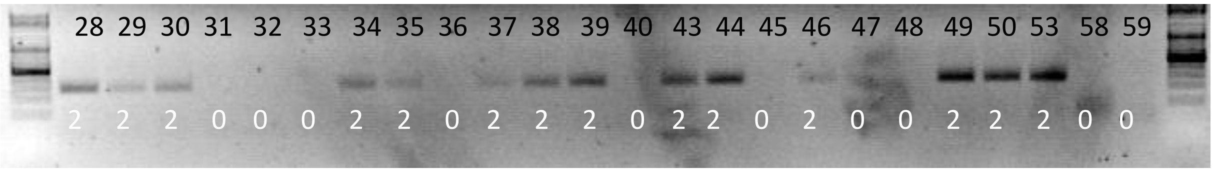

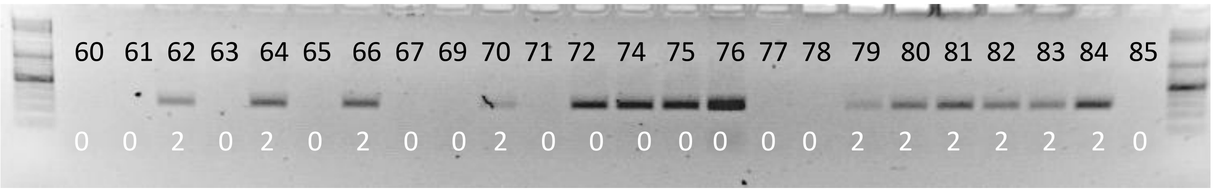

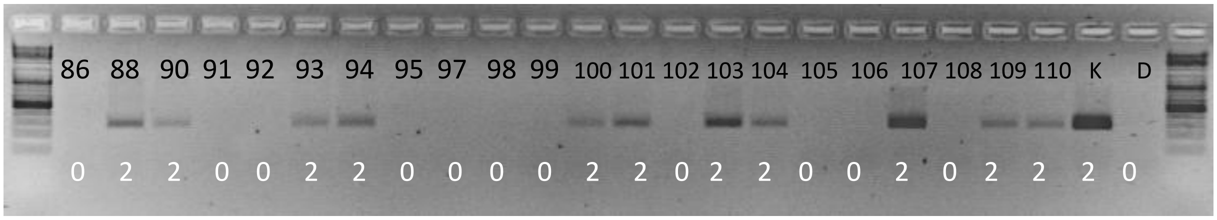

Plate 7

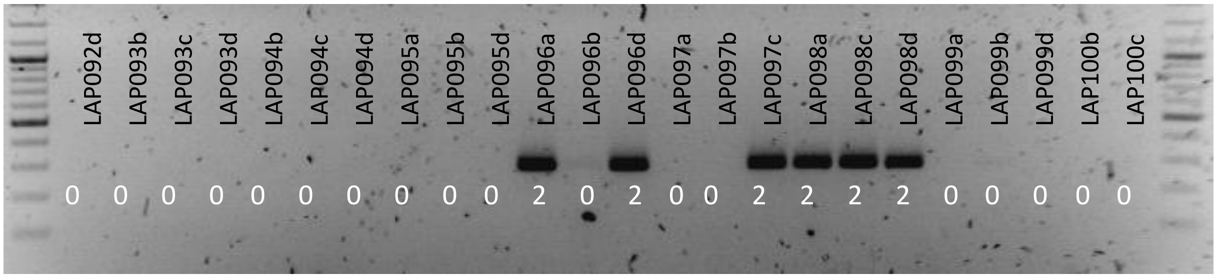
LAP092d LAP093b LAP093c LAP093d LAP094b LAP094c LAP094d LAP095a LAP095b LAP095d LAP096a LAP096b LAP096d LAP097a LAP097b LAP097c LAP098a LAP098c LAP098d LAP099a LAP099b LAP099d LAP100b LAP100c
0 0 0 0 0 0 0 0 0 0 2 0 2 0 0 2 2 2 2 0 0 0 0 0

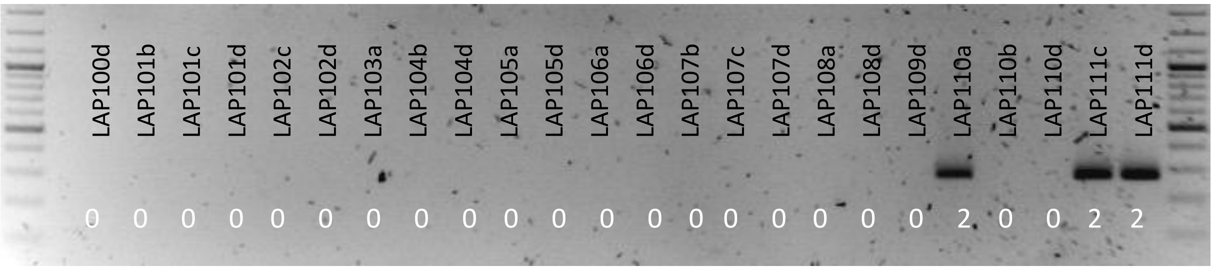
LAP100d LAP101b LAP101c LAP101d LAP102c LAP102d LAP103a LAP104b LAP104d LAP105a LAP105d LAP106a LAP106d LAP107b LAP107c LAP107d LAP108a LAP108d LAP109d LAP110a LAP110b LAP110d LAP111c LAP111d
0 0 0 0 0 0 0 0 0 0 0 0 0 0 0 0 0 0 0 2 0 0 2 2

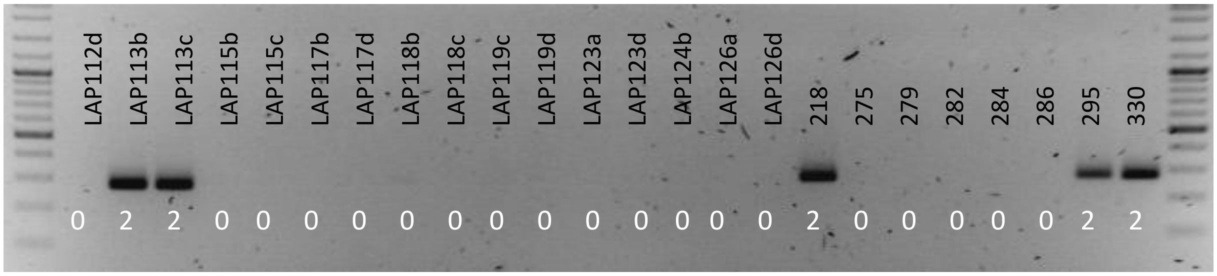
LAP112d LAP113b LAP113c LAP115b LAP115c LAP117b LAP117d LAP118b LAP118c LAP119c LAP119d LAP123a LAP123d LAP124b LAP126a LAP126d 218 275 279 282 284 286 295 330
0 2 2 0 0 0 0 0 0 0 0 0 0 0 0 0 2 0 0 0 0 0 2 2

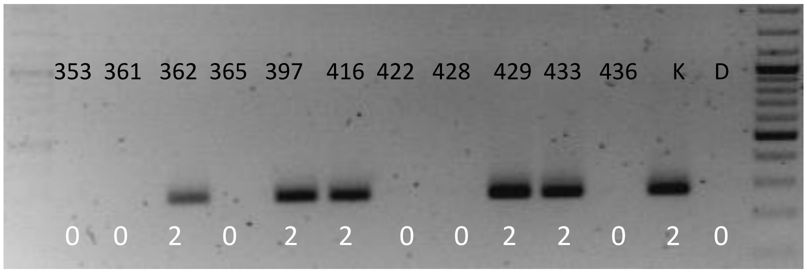
353 361 362 365 397 416 422 428 429 433 436 K D
0 0 2 0 2 2 0 0 2 2 0 2 0

PR_71a

PRFTC1F5A1 ACCATTTGACCACCTTATAACTCCC

PRFTC1R5A1 GGGACCCTTTTTCTATCAACAAAATGA

Plate 1

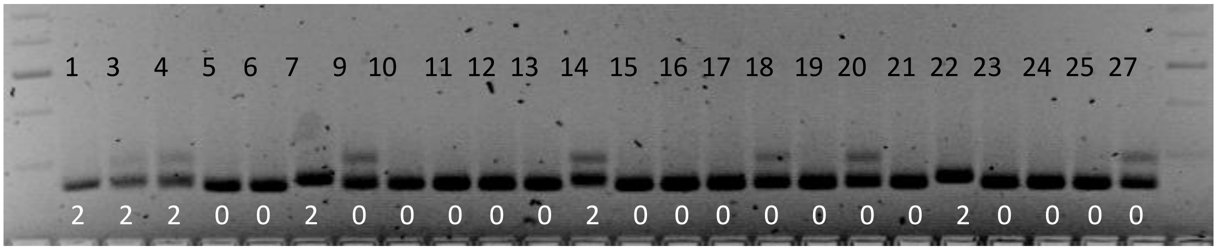

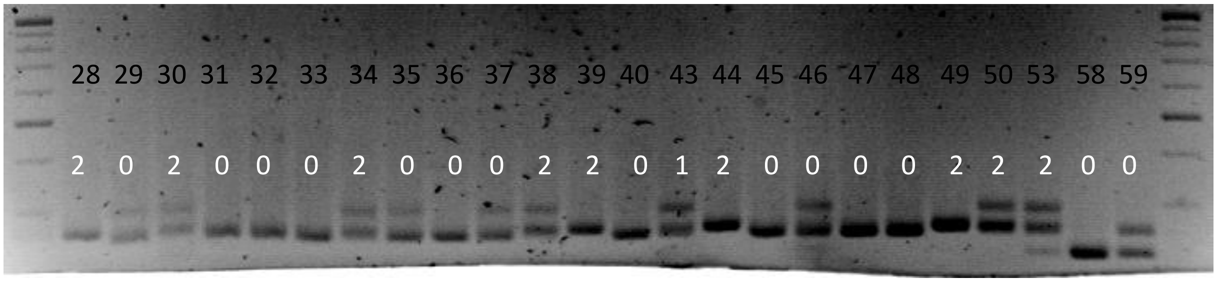

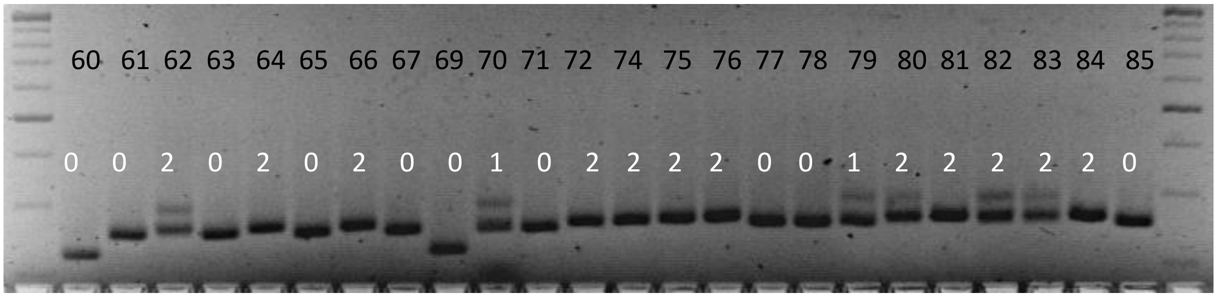

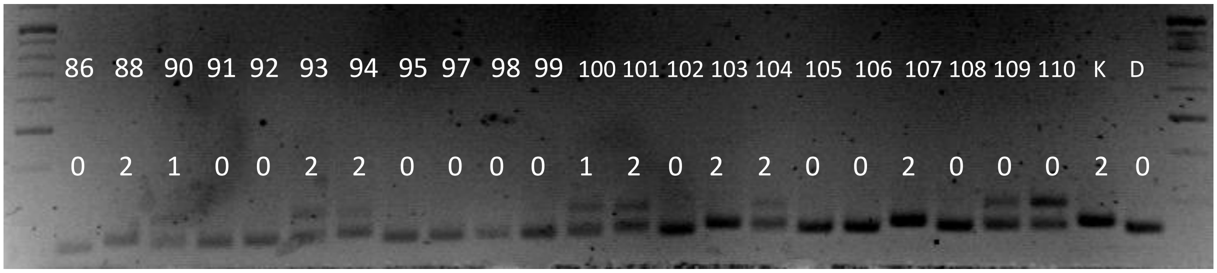

Plate 7

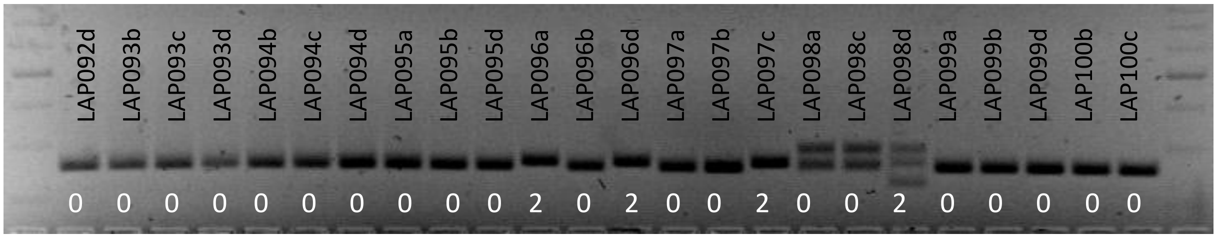
LAP092d LAP093b LAP093c LAP093d LAP094b LAP094c LAP094d LAP095a LAP095b LAP095d LAP096a LAP096b LAP096d LAP097a LAP097b LAP097c LAP098a LAP098c LAP098d LAP099a LAP099b LAP099d LAP100b LAP100c
0 0 0 0 0 0 0 0 0 0 2 0 2 0 0 2 0 0 2 0 0 0 0 0

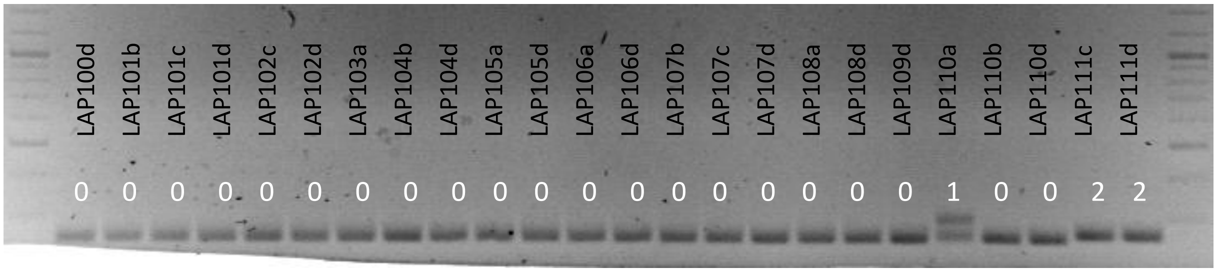
LAP100d LAP101b LAP101c LAP101d LAP102c LAP102d LAP103a LAP104b LAP104d LAP105a LAP105d LAP106a LAP106d LAP107b LAP107c LAP107d LAP108a LAP108d LAP109d LAP110a LAP110b LAP110d LAP111c LAP111d
0 0 0 0 0 0 0 0 0 0 0 0 0 0 0 0 0 0 0 1 0 0 2 2

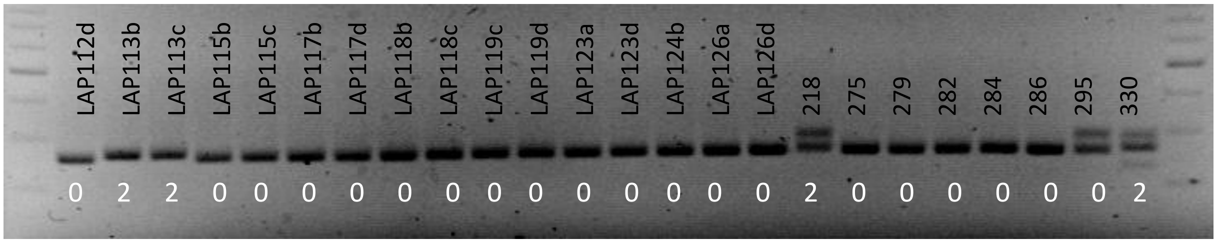
LAP112d LAP113b LAP113c LAP115b LAP115c LAP117b LAP117d LAP118b LAP118c LAP119c LAP119d LAP123a LAP123d LAP124b LAP126a LAP126d 218 275 279 282 284 286 295 330
0 2 2 0 0 0 0 0 0 0 0 0 0 0 0 0 2 0 0 0 0 0 0 2

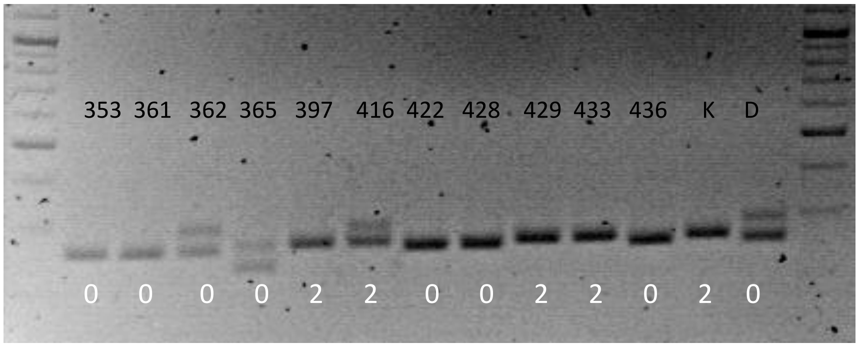
353 361 362 365 397 416 422 428 429 433 436 K D
0 0 0 0 2 2 0 0 2 2 0 2 0

PR_71b

PRFTC1F5A1 ACCATTTGACCACCTTATAACTCCC

PRFTC1R5A1 GGGACCCTTTTTCTATCAACAAAATGA

Plate 1

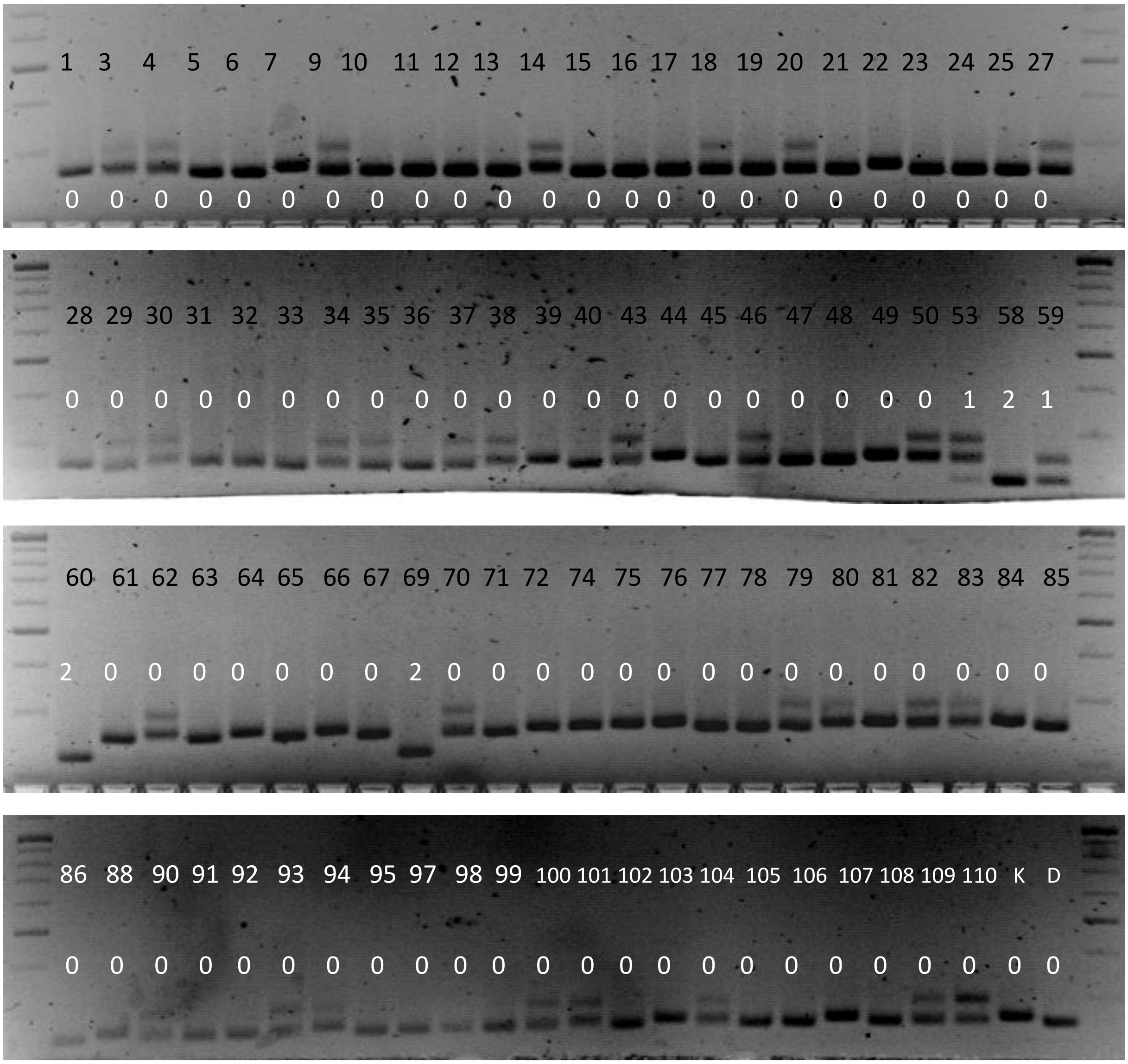

Plate 7

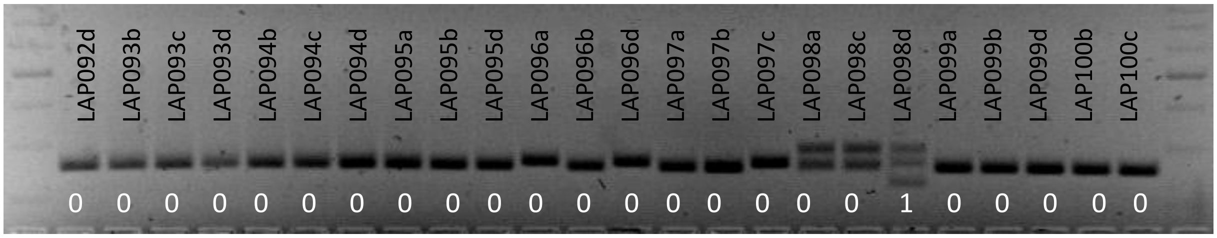
LAP092d LAP093b LAP093c LAP093d LAP094b LAP094c LAP094d LAP095a LAP095b LAP095d LAP096a LAP096b LAP096d LAP097a LAP097b LAP097c LAP098a LAP098c LAP098d LAP099a LAP099b LAP099d LAP100b LAP100c
0 0 0 0 0 0 0 0 0 0 0 0 0 0 0 0 0 0 1 0 0 0 0 0

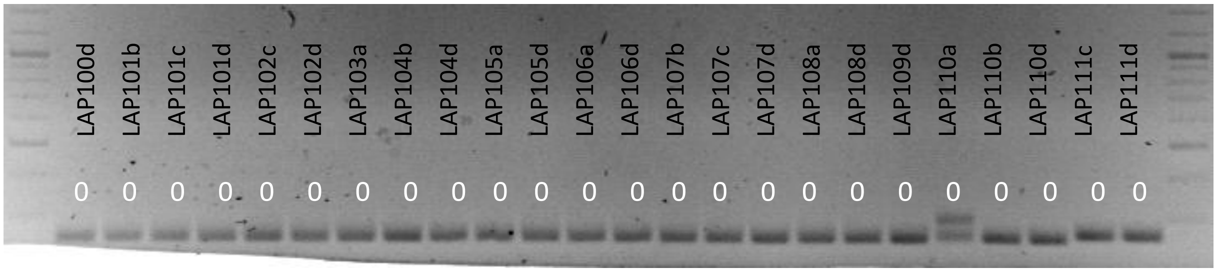
LAP100d LAP101b LAP101c LAP101d LAP102c LAP102d LAP103a LAP104b LAP104d LAP105a LAP105d LAP106a LAP106d LAP107b LAP107c LAP107d LAP108a LAP108d LAP109d LAP110a LAP110b LAP110d LAP111c LAP111d
0 0 0 0 0 0 0 0 0 0 0 0 0 0 0 0 0 0 0 0 0 0 0 0

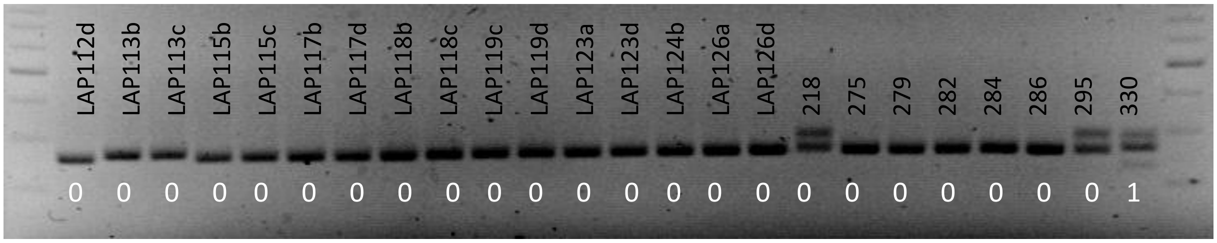
LAP112d LAP113b LAP113c LAP115b LAP115c LAP117b LAP117d LAP118b LAP118c LAP119c LAP119d LAP123a LAP123d LAP124b LAP126a LAP126d 218 275 279 282 284 286 295 330
0 0 0 0 0 0 0 0 0 0 0 0 0 0 0 0 0 0 0 0 0 0 0 1

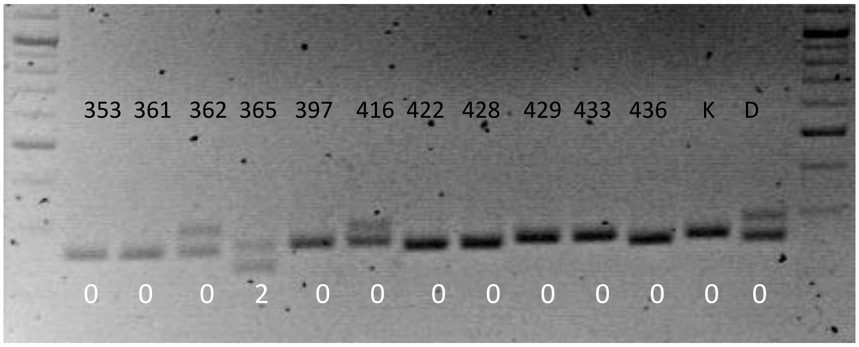
353 361 362 365 397 416 422 428 429 433 436 K D
0 0 0 2 0 0 0 0 0 0 0 0 0

PR_71c

PRFTC1F5A1 ACCATTTGACCACCTTATAACTCCC

PRFTC1R5A1 GGGACCCTTTTTCTATCAACAAAATGA

Plate 1

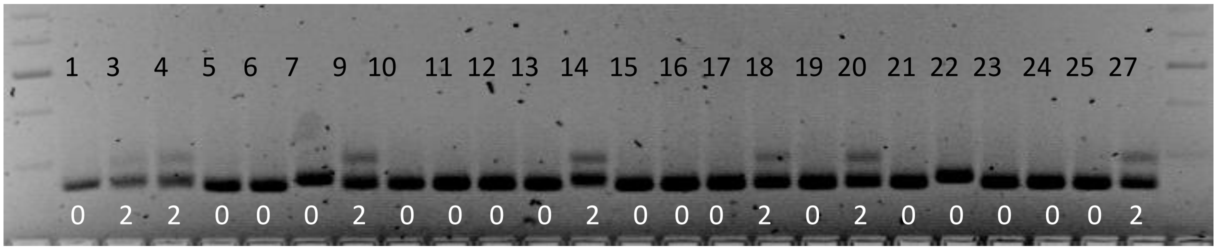

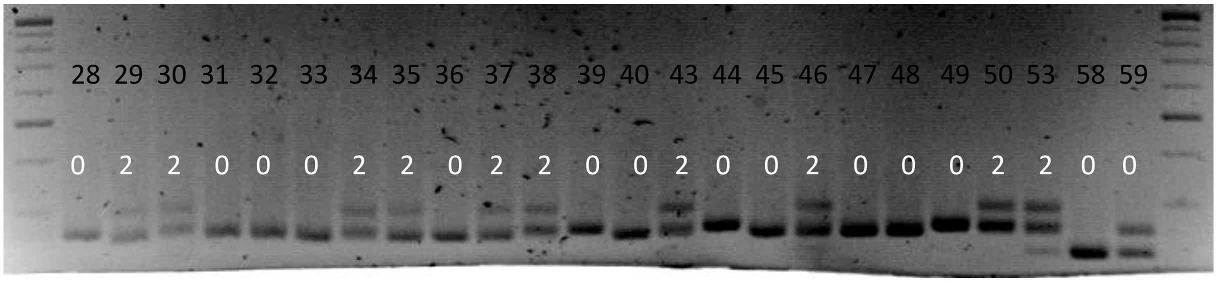

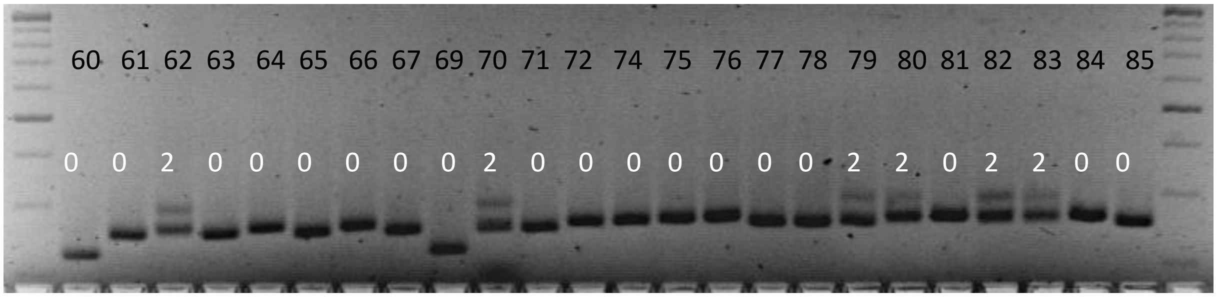

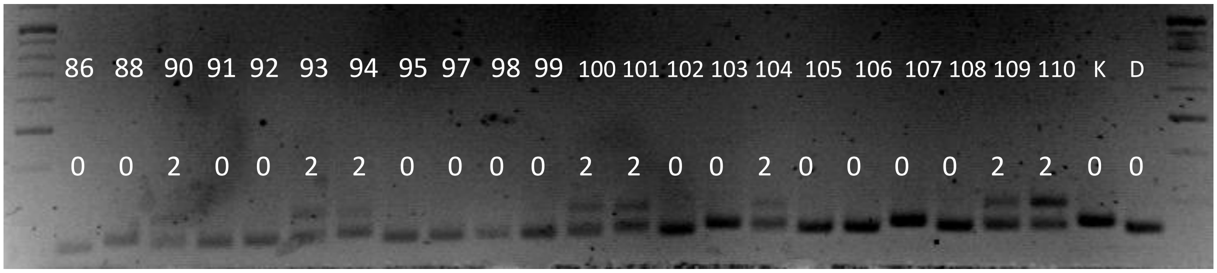

Plate 7

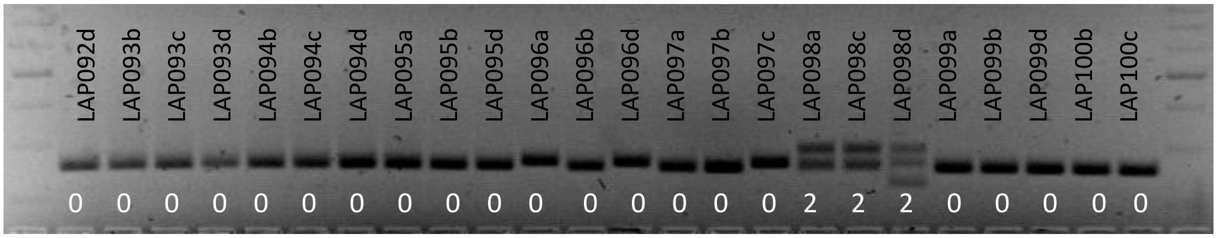
LAP092d LAP093b LAP093c LAP093d LAP094b LAP094c LAP094d LAP095a LAP095b LAP095d LAP096a LAP096b LAP096d LAP097a LAP097b LAP097c LAP098a LAP098c LAP098d LAP099a LAP099b LAP099d LAP100b LAP100c
0 0 0 0 0 0 0 0 0 0 0 0 0 0 0 0 2 2 2 0 0 0 0 0

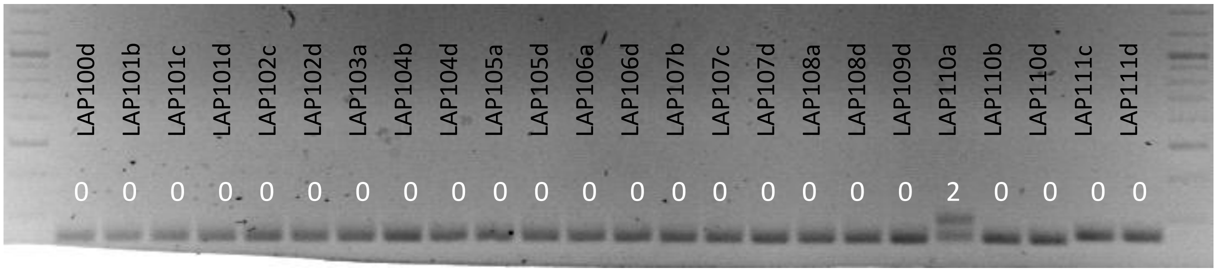
LAP100d LAP101b LAP101c LAP101d LAP102c LAP102d LAP103a LAP104b LAP104d LAP105a LAP105d LAP106a LAP106d LAP107b LAP107c LAP107d LAP108a LAP108d LAP109d LAP110a LAP110b LAP110d LAP111c LAP111d
0 0 0 0 0 0 0 0 0 0 0 0 0 0 0 0 0 0 0 2 0 0 0 0

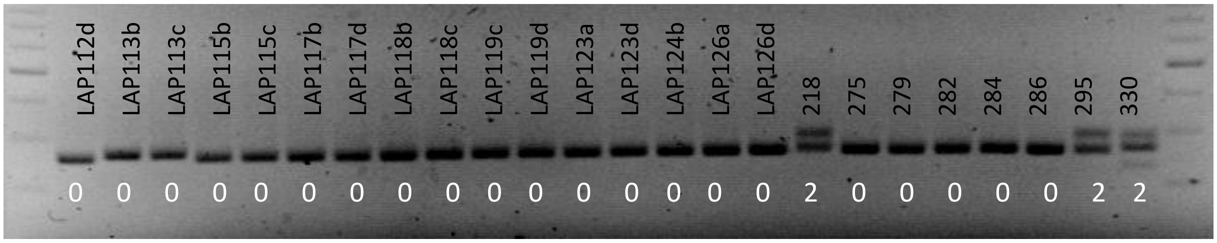
LAP112d LAP113b LAP113c LAP115b LAP115c LAP117b LAP117d LAP118b LAP118c LAP119c LAP119d LAP123a LAP123d LAP124b LAP126a LAP126d 218 275 279 282 284 286 295 330
0 0 0 0 0 0 0 0 0 0 0 0 0 0 0 0 2 0 0 0 0 0 2 2

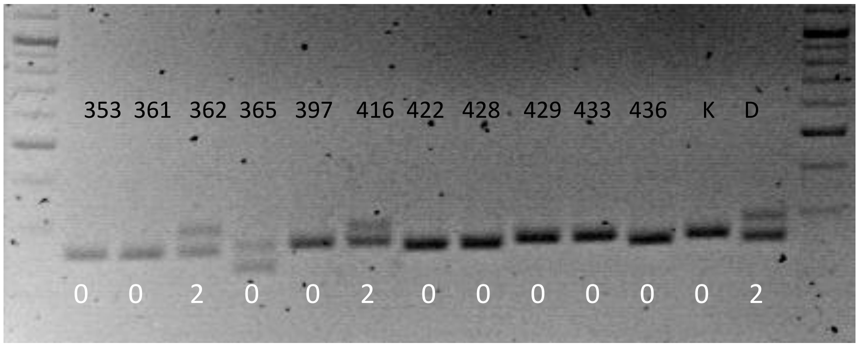
353 361 362 365 397 416 422 428 429 433 436 K D
0 0 2 0 0 2 0 0 0 0 0 0 2

PR_71d

PRFTC1F5A1 ACCATTTGACCACCTTATAACTCCC

PRFTC1R5A1 GGGACCCTTTTTCTATCAACAAAATGA

Plate 1

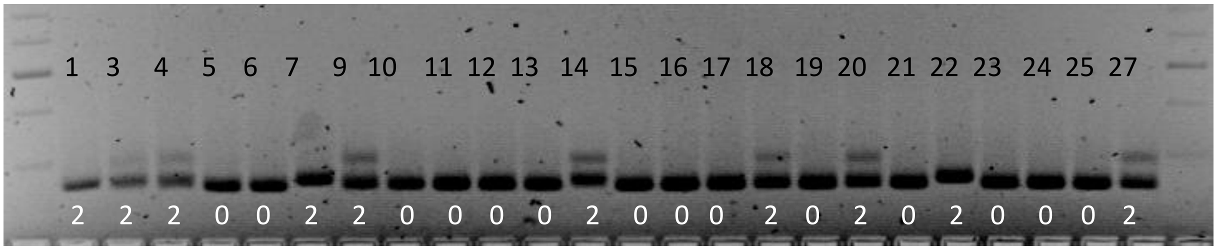

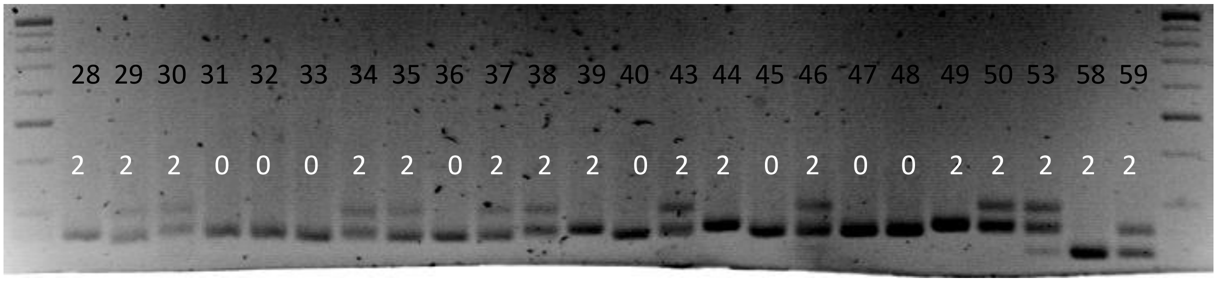

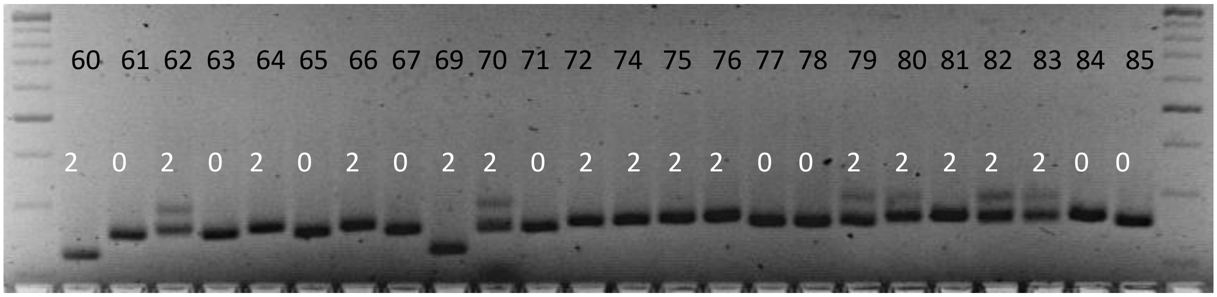

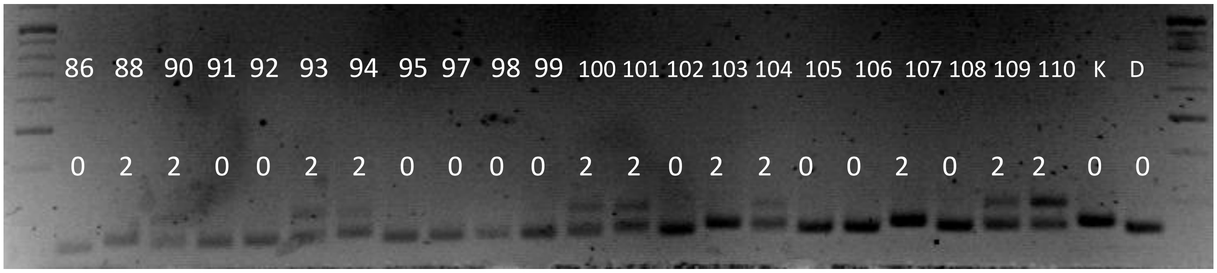

Plate 7

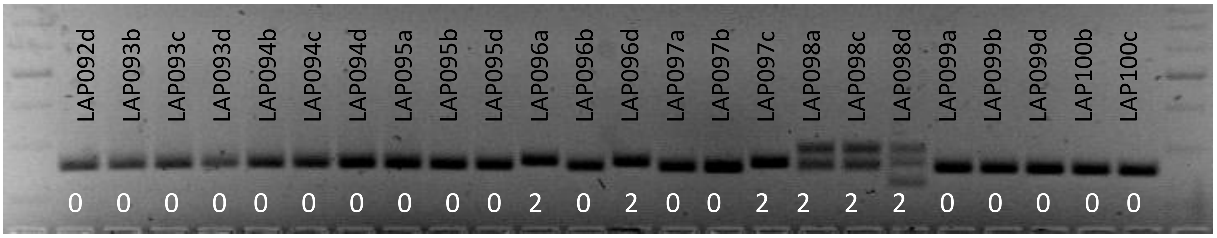
LAP092d LAP093b LAP093c LAP093d LAP094b LAP094c LAP094d LAP095a LAP095b LAP095d LAP096a LAP096b LAP096d LAP097a LAP097b LAP097c LAP098a LAP098c LAP098d LAP099a LAP099b LAP099d LAP100b LAP100c
0 0 0 0 0 0 0 0 0 0 2 0 2 0 0 2 2 2 2 0 0 0 0 0

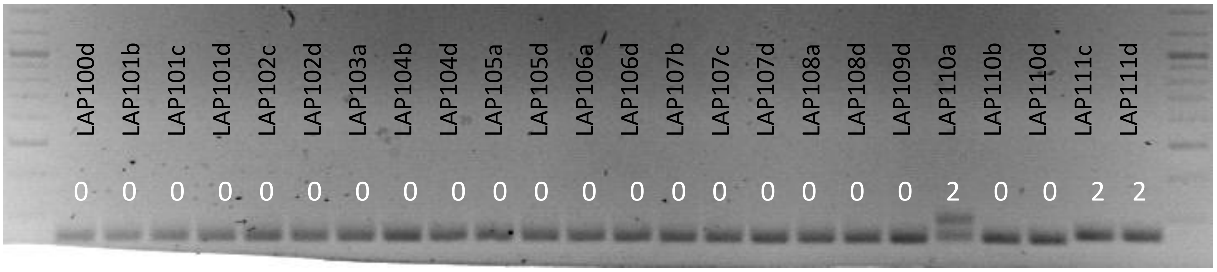
LAP100d LAP101b LAP101c LAP101d LAP102c LAP102d LAP103a LAP104b LAP104d LAP105a LAP105d LAP106a LAP106d LAP107b LAP107c LAP107d LAP108a LAP108d LAP109d LAP110a LAP110b LAP110d LAP111c LAP111d
0 0 0 0 0 0 0 0 0 0 0 0 0 0 0 0 0 0 0 2 0 0 2 2

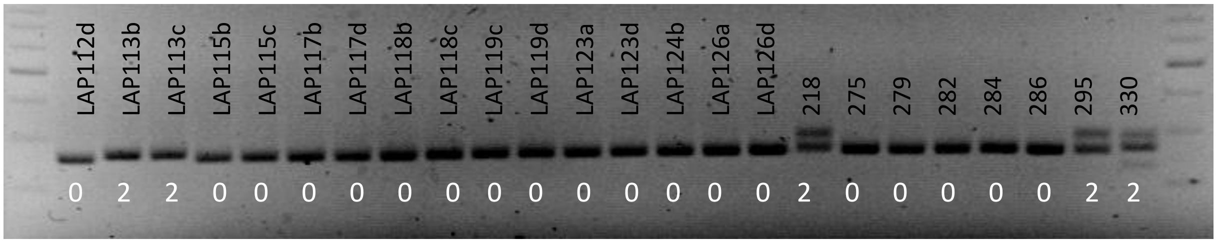
LAP112d LAP113b LAP113c LAP115b LAP115c LAP117b LAP117d LAP118b LAP118c LAP119c LAP119d LAP123a LAP123d LAP124b LAP126a LAP126d 218 275 279 282 284 286 295 330
0 2 2 0 0 0 0 0 0 0 0 0 0 0 0 0 2 0 0 0 0 0 2 2

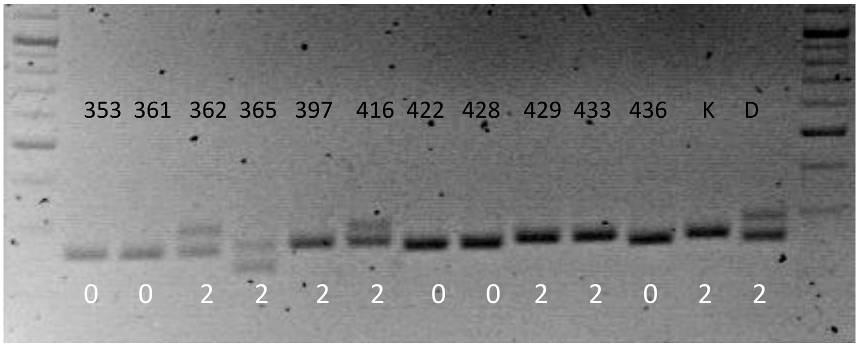
353 361 362 365 397 416 422 428 429 433 436 K D
0 0 2 2 2 2 0 0 2 2 0 2 2

PR80

PRFTc1_F2e CAACAATGCACAATGGAAAGGA

PRFTc1_R2e TCTGCTAATCTGAGTTCCAATTTGT

Plate 1

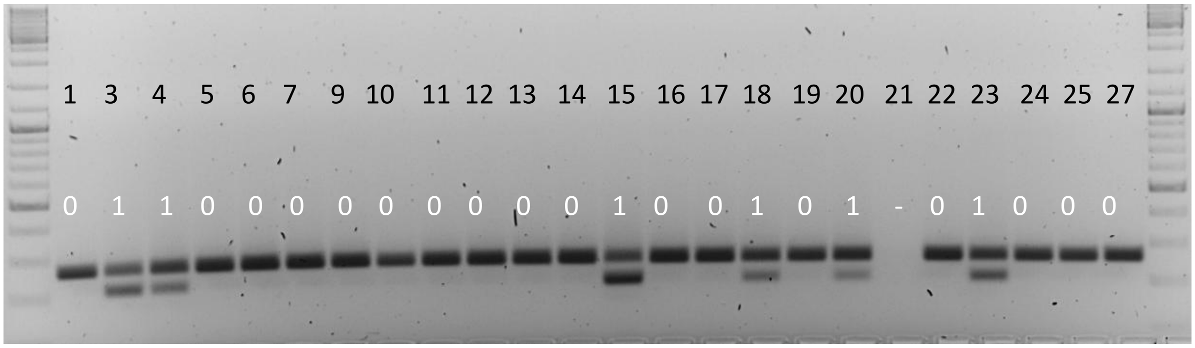

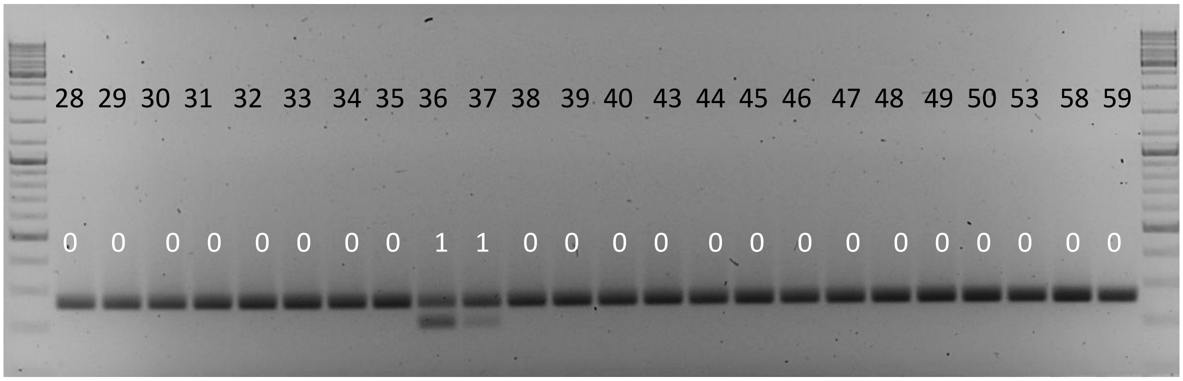

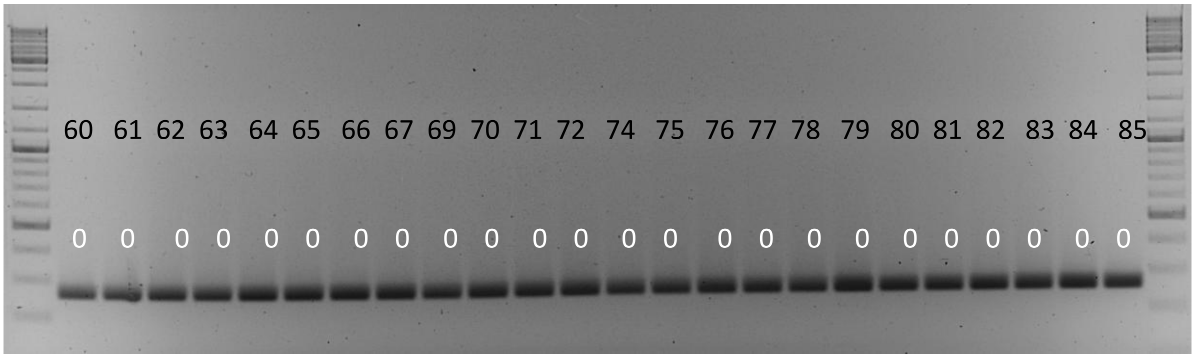

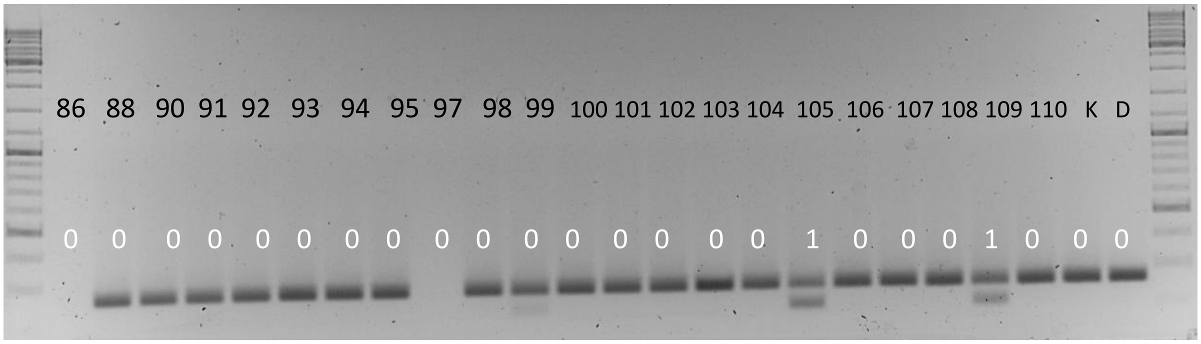

Plate 7

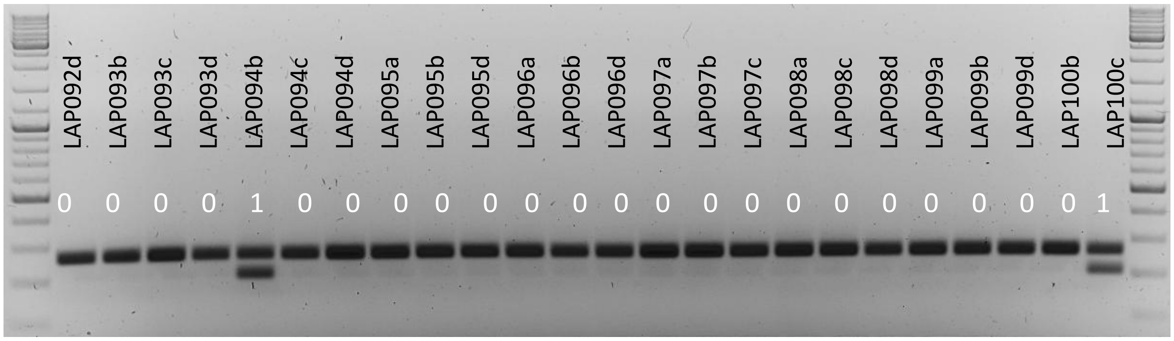
LAP092d LAP093b LAP093c LAP093d LAP094b LAP094c LAP094d LAP095a LAP095b LAP095d LAP096a LAP096b LAP096d LAP097a LAP097b LAP097c LAP098a LAP098c LAP098d LAP099a LAP099b LAP099d LAP100b LAP100c
0 0 0 0 1 0 0 0 0 0 0 0 0 0 0 0 0 0 0 0 0 0 0 1

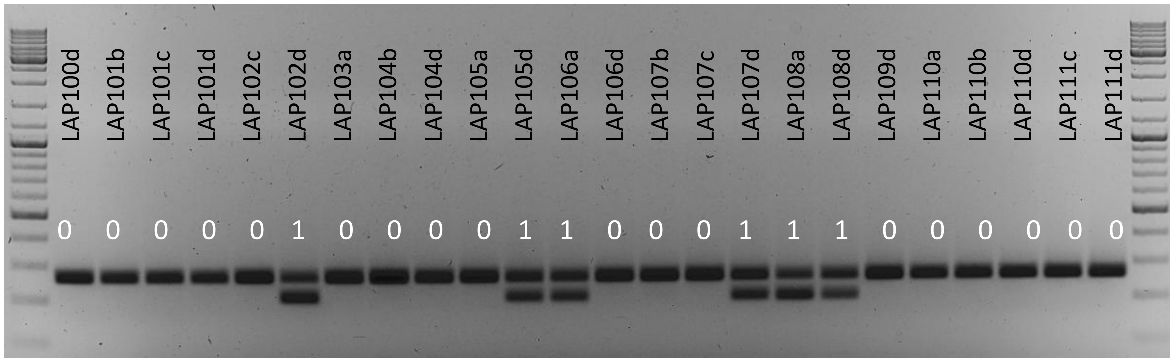
LAP100d LAP101b LAP101c LAP101d LAP102c LAP102d LAP103a LAP104b LAP104d LAP105a LAP105d LAP106a LAP106d LAP107b LAP107c LAP107d LAP108a LAP108d LAP109d LAP110a LAP110b LAP110d LAP111c LAP111d
0 0 0 0 0 1 0 0 0 0 1 1 0 0 0 1 1 1 0 0 0 0 0 0

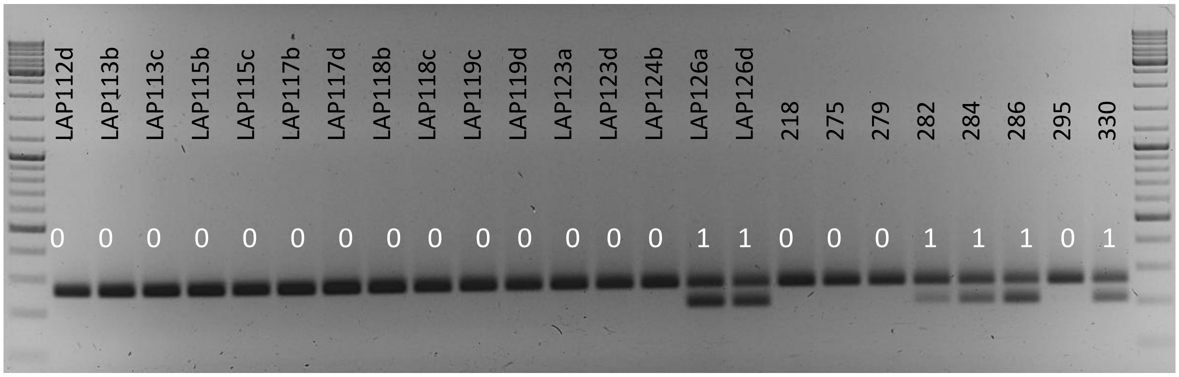
LAP112d LAP113b LAP113c LAP115b LAP115c LAP117b LAP117d LAP118b LAP118c LAP119c LAP119d LAP123a LAP123d LAP124b LAP126a LAP126d 218 275 279 282 284 286 295 330
0 0 0 0 0 0 0 0 0 0 0 0 0 0 1 1 0 0 0 1 1 1 0 1

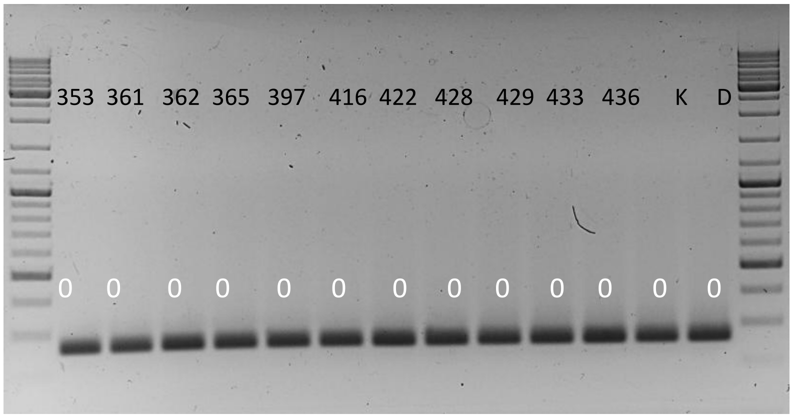
353 361 362 365 397 416 422 428 429 433 436 K D
0 0 0 0 0 0 0 0 0 0 0 0 0

Repeat

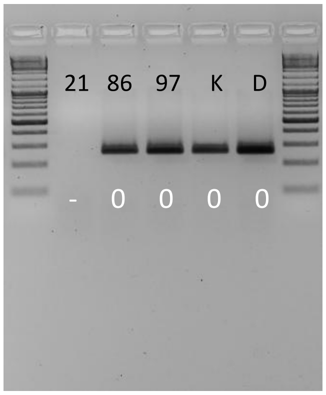
21
86
97
K
D
-
0
0
0
0

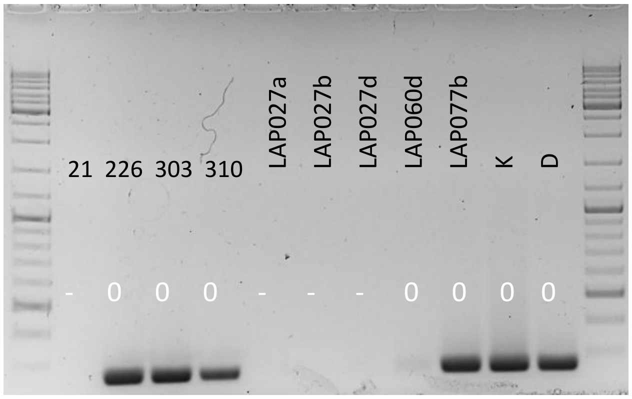
21
226
303
310
LAP027a
LAP027b
LAP027d
LAP060d
LAP077b
K
D
-
0
0
0
-
-
-
0
0
0
0
