# Supplementary material for: Evaluation of the Global White Lupin Collection Reveals Significant Associations Between Homologous FLOWERING LOCUS T Indels and Flowering Time, Providing Validated Markers for Tracking Spring Ecotypes Within a Large Gene Pool
Source: Int J Mol Sci. 2025 Jul 17;26(14):6858. doi: 10.3390/ijms26146858 (PMC12295241; doi:10.3390/ijms26146858)
Supplement: Supplementary file 1 [file ijms-26-06858-s001.zip › Supplementary_Figure_S7_Correlation of PCR markers tagging LalbFTc1 gene indels with white lupin phenology.pdf]

Wojciech Bielski, Anna Surma, Michał Książkiewicz, Sandra Rychel-Bielska

Evaluation of the global white lupin collection reveals significant associations between homologous *FLOWERING LOCUS T* indels and flowering time, providing validated markers for tracking spring ecotypes within a large gene pool

International Journal of Molecular Sciences

**Supplementary Figure S7**. Correlation of PCR markers tagging *LalbFTc1* gene indels with white lupin phenology. Phenotyping was conducted without pre-sowing vernalization during the 2020 and 2021 growing seasons in a greenhouse located at the Institute of Plant Genetics, Polish Academy of Sciences in Poznań. Spearman′s rank correlation coefficient calculated for three phenology traits (the number of days to floral bud emergence (BE), start of flowering (SF), and end of flowering (EF)) is presented in color scale from -0.5 to 0.5, whereas the Bonferroni-corrected p-value is shown in the following scheme: ***, $p < 0.0001$; **, $0.0001 \le p < 0.001$; *, $0.001 \le p \le 0.05$; no symbol, $p > 0.05$ (not significant).

| Marker | Variant | BE 2020 | SF 2020 | EF 2020 | BE 2021 | SF 2021 | EF 2021 |
|---|---|---|---|---|---|---|---|
| PR_58a | indel 2 | | | | | | |
| PR_60a | indel 2 | *** | *** | *** | *** | ** | ** |
| PR_58b | indels 2, 3 | | | * | * | * | * |
| PR_30 | indels 2, 3 | | | | | | |
| PR_58c | indel 3 | *** | *** | *** | *** | *** | *** |
| PR_60b | indel 3 | *** | *** | *** | *** | *** | *** |
| PR_60c | indel 3 | *** | *** | *** | *** | *** | *** |
| PR_80 | indels 5, 6 | *** | *** | *** | *** | *** | *** |
| PR_35b | indel 7 | ** | ** | *** | *** | *** | *** |
| PR_35a | indel 7 | ** | ** | ** | *** | *** | *** |
| PR_36b | indels 8, 9 | *** | *** | *** | *** | *** | *** |
| PR_36a | indels 8, 9 | | | | | | |
| PR_39 | indels 11-13 | *** | *** | *** | *** | *** | *** |
| PR_41 | SNP | ** | *** | ** | *** | *** | *** |
| PR_70 | indel 15 | *** | *** | *** | *** | *** | *** |
| PR_42a | indels 15, 16 | *** | *** | *** | *** | *** | *** |
| PR_42b | indels 15, 16 | | * | * | * | * | * |
| PR_71d | indels 15, 16 | *** | *** | *** | *** | *** | *** |
| PR_71a | indels 15, 16 | *** | *** | *** | *** | *** | *** |
| PR_71b | indels 15, 16 | *** | *** | *** | *** | *** | *** |
| PR_71c | indels 15, 16 | * | * | * | ** | * | * |

| Scale | -0.50 | -0.33 | -0.17 | 0.00 | 0.17 | 0.33 | 0.50 |
|---|---|---|---|---|---|---|---|
