# Supplementary material for: Evaluation of the Global White Lupin Collection Reveals Significant Associations Between Homologous FLOWERING LOCUS T Indels and Flowering Time, Providing Validated Markers for Tracking Spring Ecotypes Within a Large Gene Pool
Source: Int J Mol Sci. 2025 Jul 17;26(14):6858. doi: 10.3390/ijms26146858 (PMC12295241; doi:10.3390/ijms26146858)
Supplement: Supplementary file 1 [file ijms-26-06858-s001.zip › Supplementary_Figure_S8_Agarose gel electrophoregrams showing polymorphism of PCR-based markers targeting LalbFTc2 indels.pdf]

Wojciech Bielski, Anna Surma, Michał Książkiewicz, Sandra Rychel-Bielska

Evaluation of the global white lupin collection reveals significant associations between homologous *FLOWERING LOCUS T* indels and flowering time, providing validated markers for tracking spring ecotypes within a large gene pool

International Journal of Molecular Sciences

Supplementary Figure S8. Agarose gel electrophoregrams showing polymorphism of PCR-based markers targeting *LalbFTc2* indels.

PR_44

PRFTC2F1 GTTGGCATTTTAACTTTATCCTCATAG

PRFTc2_R1b ACCAGATGATAGAGTTATTCAAGGA

Plate 1

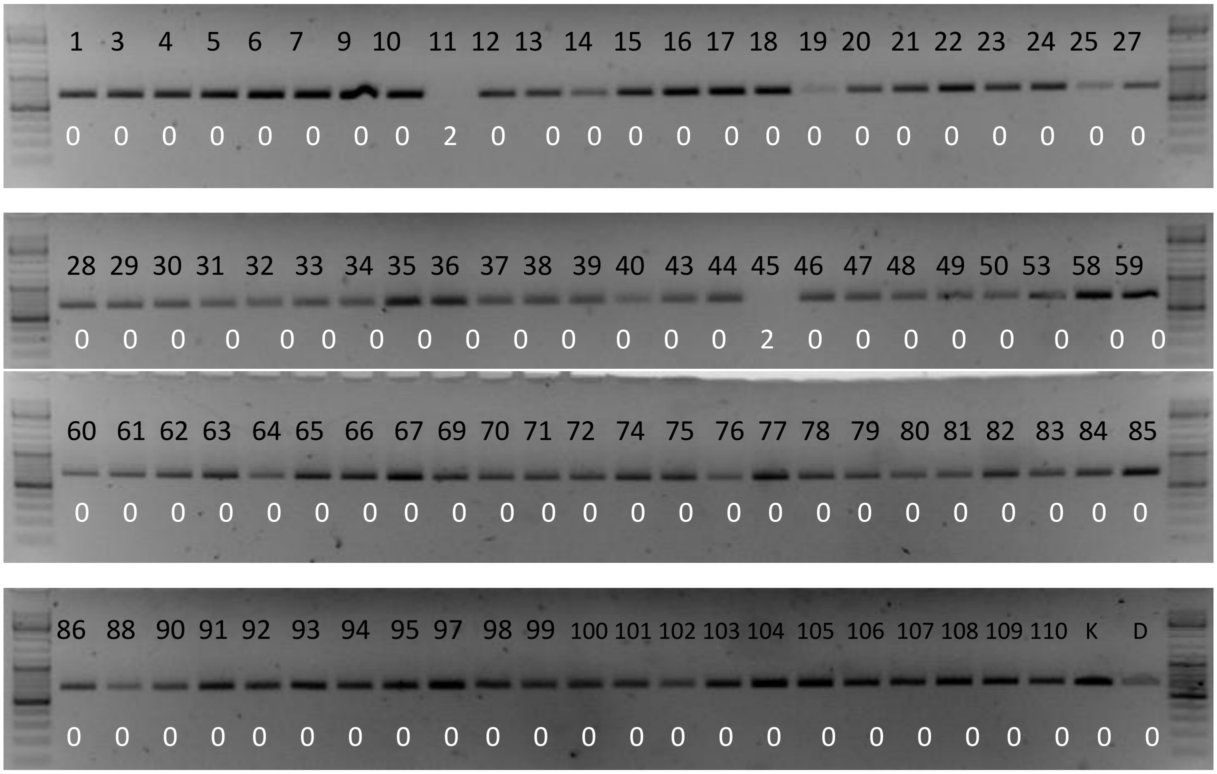

Plate 9

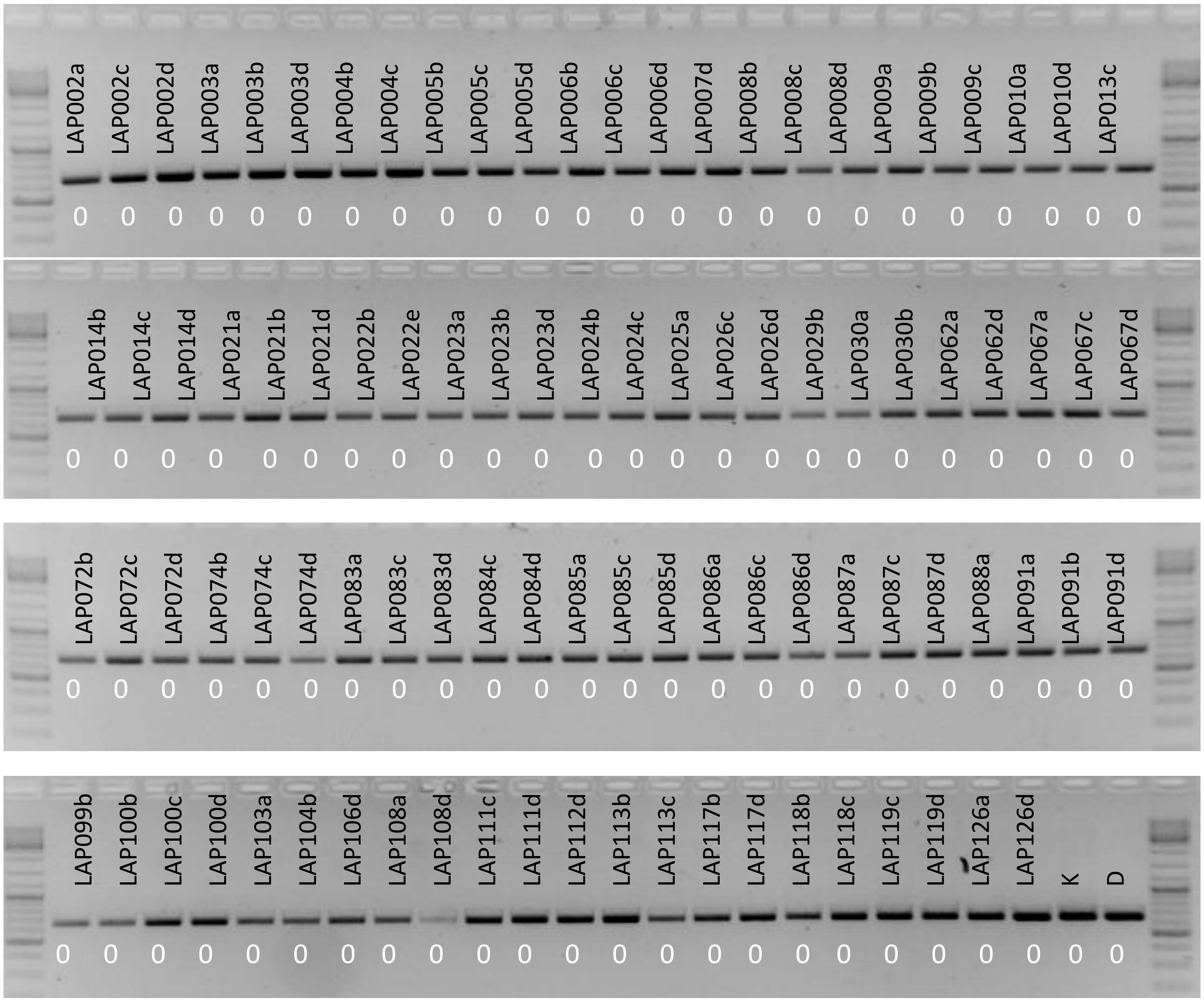

Repeat

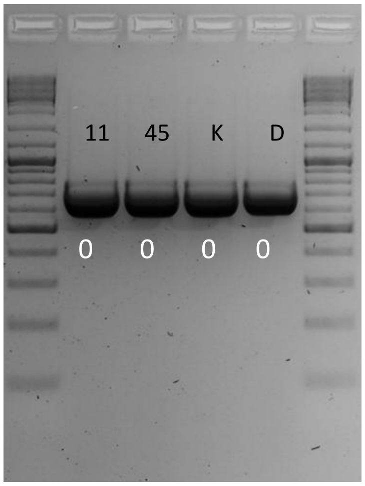

PR_45

PRFTc2_F1b GCACATCCACAACTAAGATTAAAGC

PRFTc2_R1c TTGGGAATTAAGTCTCACATCACCT

Plate 1

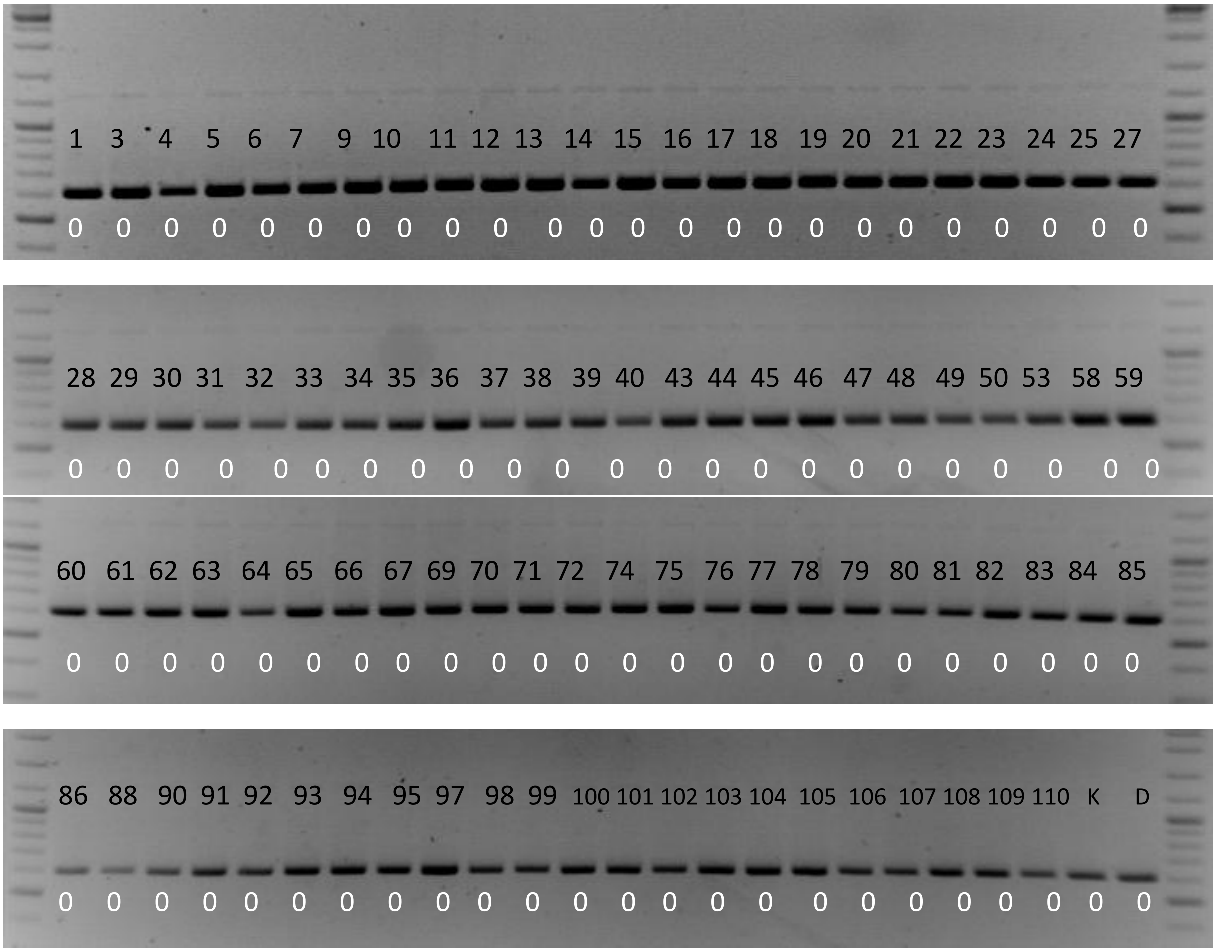

Plate 9

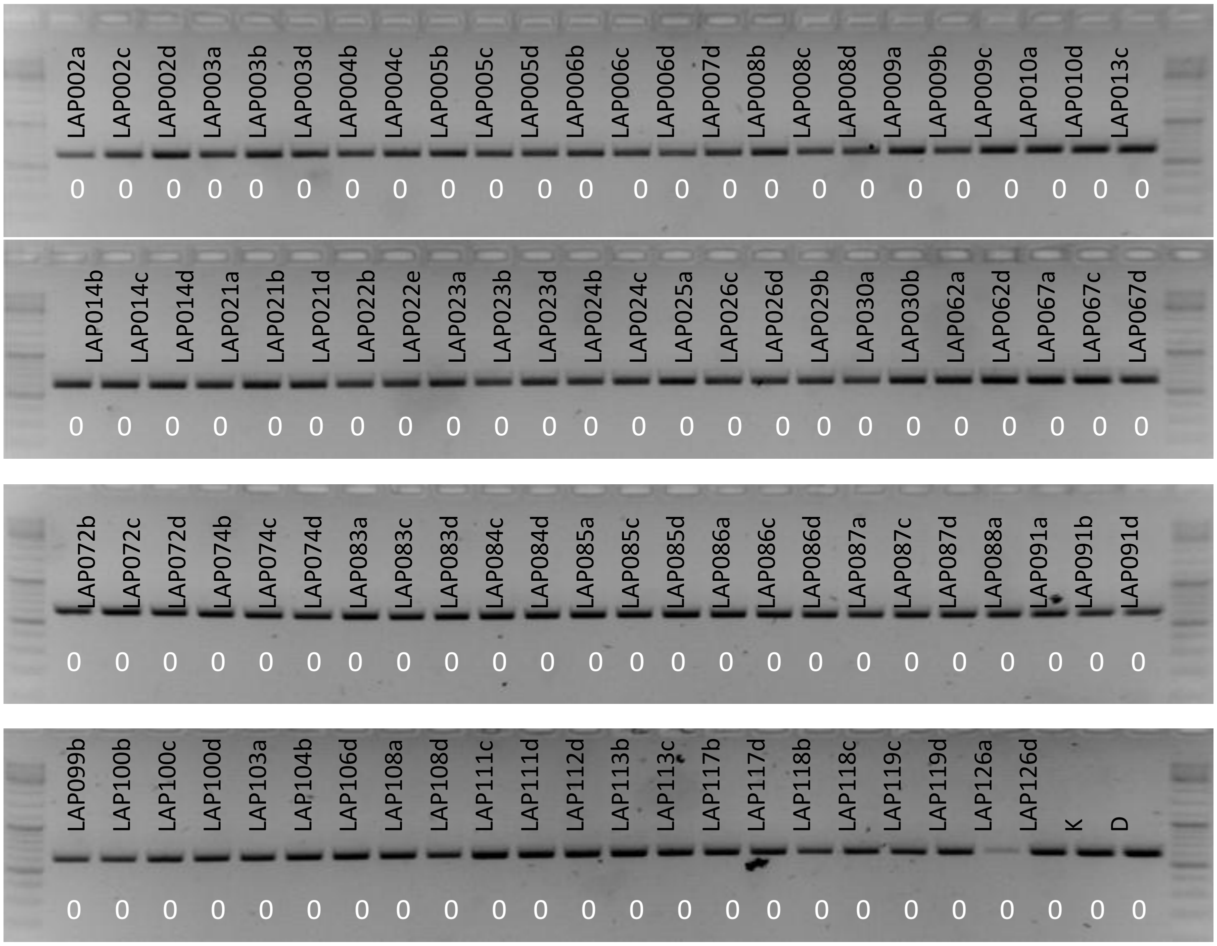
LAP002a LAP002c LAP002d LAP003a LAP003b LAP003d LAP004b LAP004c LAP005b LAP005c LAP005d LAP006b LAP006c LAP006d LAP007d LAP008b LAP008c LAP008d LAP009a LAP009b LAP009c LAP010a LAP010d LAP013c
0 0 0 0 0 0 0 0 0 0 0 0 0 0 0 0 0 0 0 0 0 0 0 0
LAP014b LAP014c LAP014d LAP021a LAP021b LAP021d LAP022b LAP022e LAP023a LAP023b LAP023d LAP024b LAP024c LAP025a LAP026c LAP026d LAP029b LAP030a LAP030b LAP062a LAP062d LAP067a LAP067c LAP067d
0 0 0 0 0 0 0 0 0 0 0 0 0 0 0 0 0 0 0 0 0 0 0 0
LAP072b LAP072c LAP072d LAP074b LAP074c LAP074d LAP083a LAP083c LAP083d LAP084c LAP084d LAP085a LAP085c LAP085d LAP086a LAP086c LAP086d LAP087a LAP087c LAP087d LAP088a LAP091a LAP091b LAP091d
0 0 0 0 0 0 0 0 0 0 0 0 0 0 0 0 0 0 0 0 0 0 0 0
LAP099b LAP100b LAP100c LAP100d LAP103a LAP104b LAP106d LAP108a LAP108d LAP111c LAP111d LAP112d LAP113b LAP113c LAP117b LAP117d LAP118b LAP118c LAP119c LAP119d LAP126a LAP126d K D
0 0 0 0 0 0 0 0 0 0 0 0 0 0 0 0 0 0 0 0 0 0 0 0

PR_46

PRFTc2_F1c ACAATGAGAGGAGTAGTCAAACATGA

PRFTC2R1 GCTTGGACGGTGGTAGTAATG

Plate 1

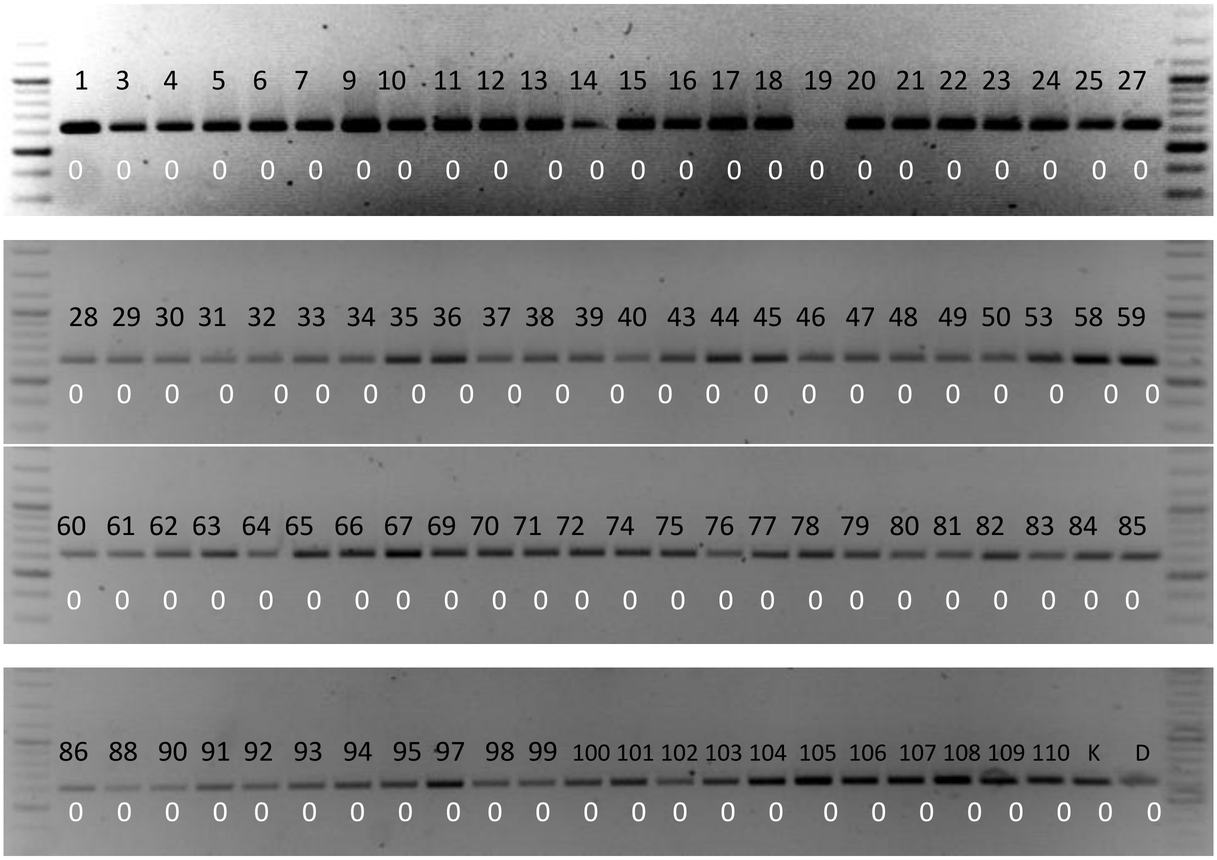

Plate 9

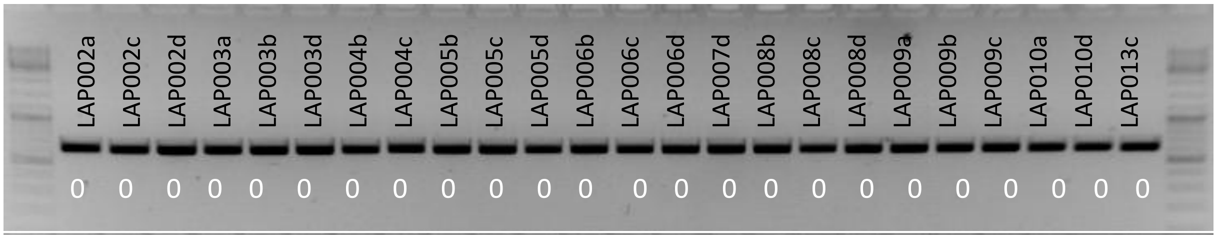
LAP002a LAP002c LAP002d LAP003a LAP003b LAP003d LAP004b LAP004c LAP005b LAP005c LAP005d LAP006b LAP006c LAP006d LAP007d LAP008b LAP008c LAP008d LAP009a LAP009b LAP009c LAP010a LAP010d LAP013c
0 0 0 0 0 0 0 0 0 0 0 0 0 0 0 0 0 0 0 0 0 0 0 0

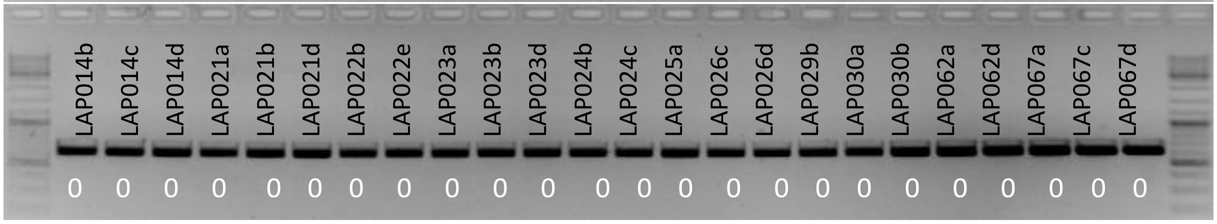
LAP014b LAP014c LAP014d LAP021a LAP021b LAP021d LAP022b LAP022e LAP023a LAP023b LAP023d LAP024b LAP024c LAP025a LAP026c LAP026d LAP029b LAP030a LAP030b LAP062a LAP062d LAP067a LAP067c LAP067d
0 0 0 0 0 0 0 0 0 0 0 0 0 0 0 0 0 0 0 0 0 0 0 0

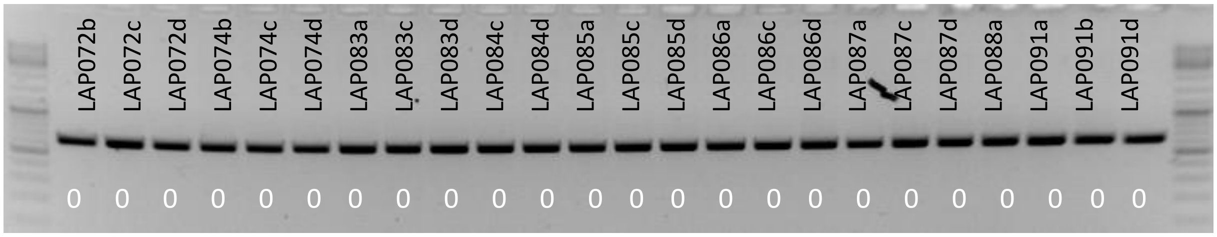
LAP072b LAP072c LAP072d LAP074b LAP074c LAP074d LAP083a LAP083c LAP083d LAP084c LAP084d LAP085a LAP085c LAP085d LAP086a LAP086c LAP086d LAP087a LAP087c LAP087d LAP088a LAP091a LAP091b LAP091d
0 0 0 0 0 0 0 0 0 0 0 0 0 0 0 0 0 0 0 0 0 0 0 0

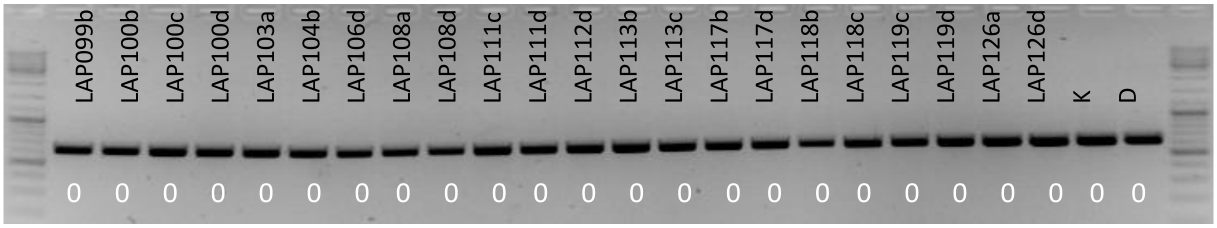
LAP099b LAP100b LAP100c LAP100d LAP103a LAP104b LAP106d LAP108a LAP108d LAP111c LAP111d LAP112d LAP113b LAP113c LAP117b LAP117d LAP118b LAP118c LAP119c LAP119d LAP126a LAP126d K D
0 0 0 0 0 0 0 0 0 0 0 0 0 0 0 0 0 0 0 0 0 0 0 0

PR_47

PRFTC2F2 GACTTACTTCCCAACATCACTATCG

PRFTc2_R2b TGTGAAGCAGAGTATATAGGTGCTT

Plate 1

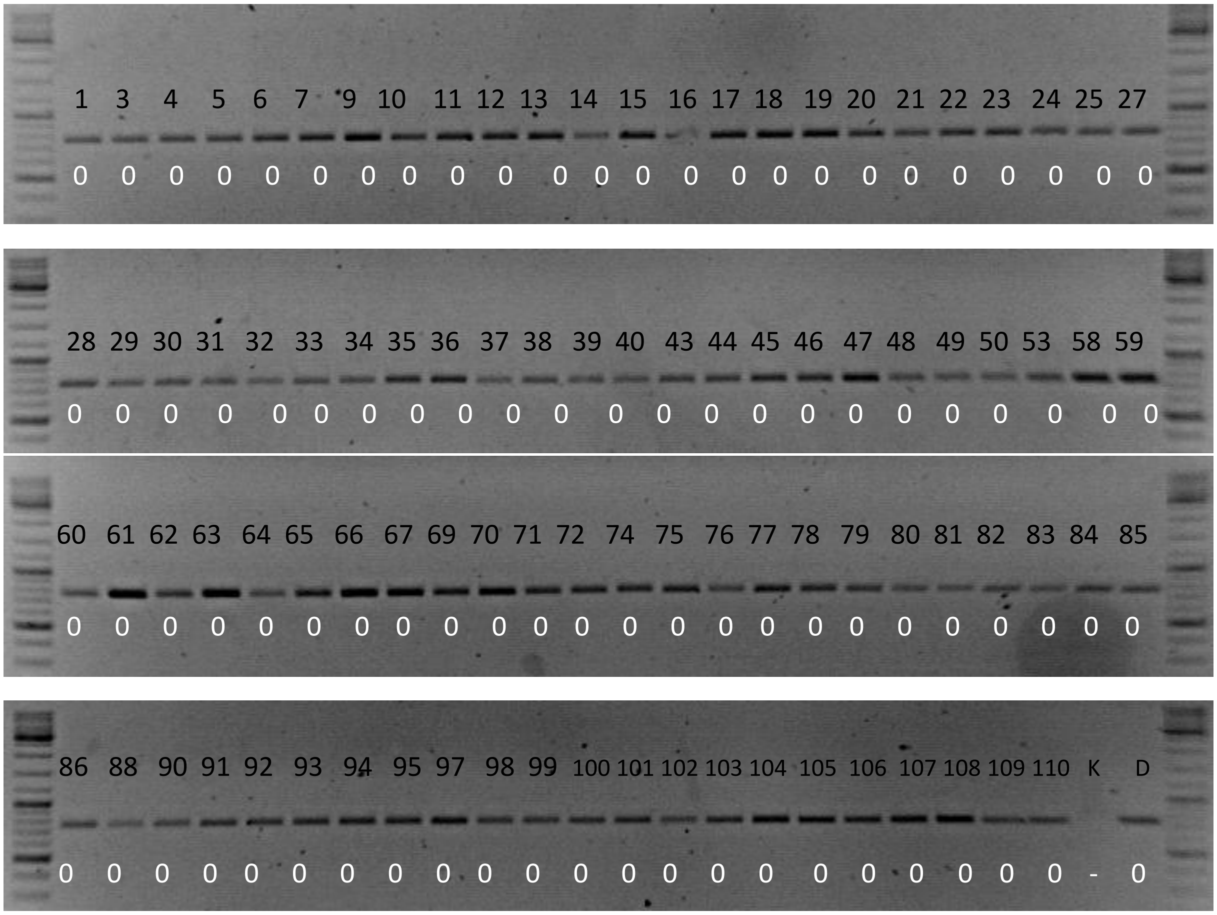

Plate 9

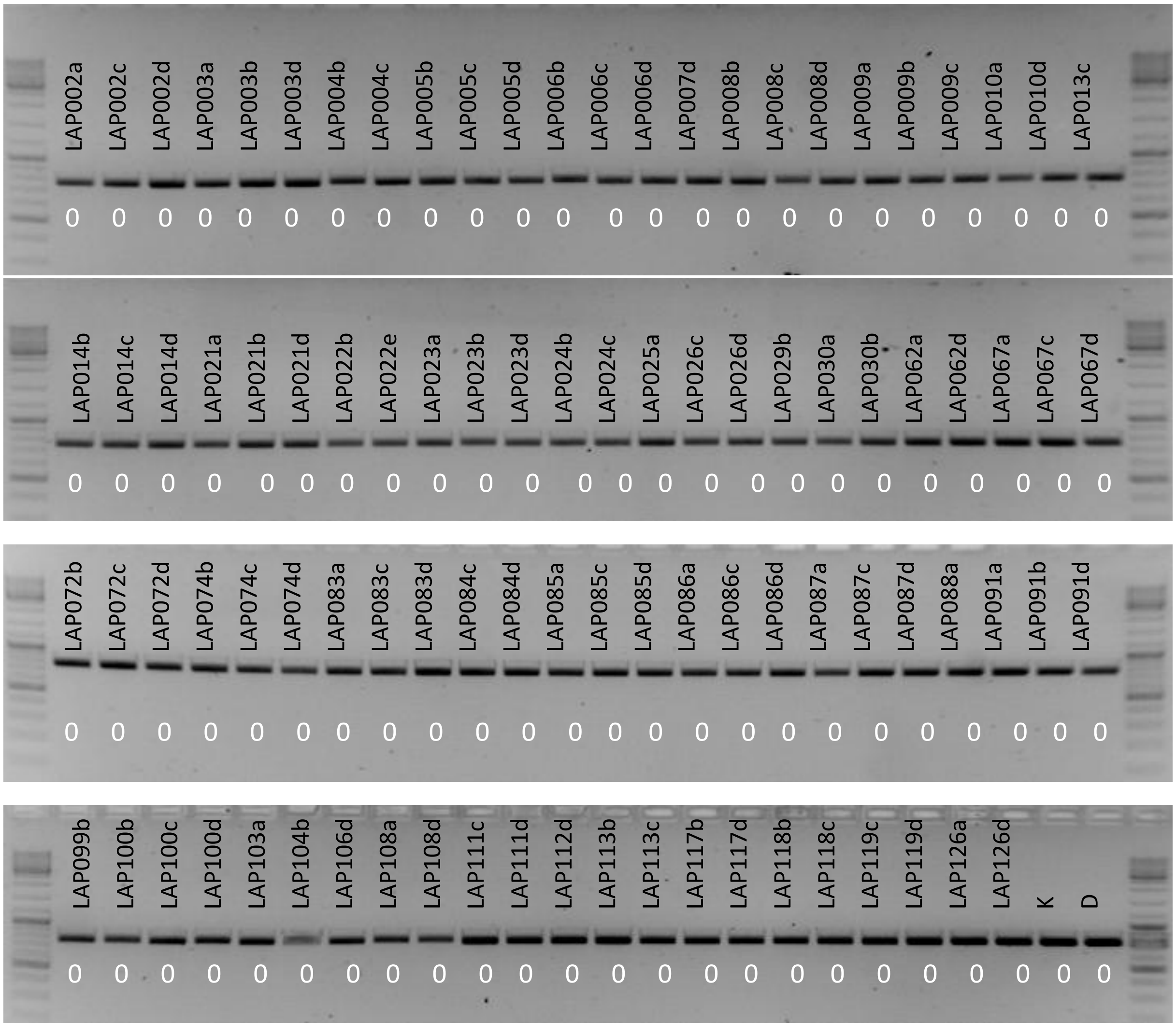
LAP002a LAP002c LAP002d LAP003a LAP003b LAP003d LAP004b LAP004c LAP005b LAP005c LAP005d LAP006b LAP006c LAP006d LAP007d LAP008b LAP008c LAP008d LAP009a LAP009b LAP009c LAP010a LAP010d LAP013c
0 0 0 0 0 0 0 0 0 0 0 0 0 0 0 0 0 0 0 0 0 0 0 0
LAP014b LAP014c LAP014d LAP021a LAP021b LAP021d LAP022b LAP022e LAP023a LAP023b LAP023d LAP024b LAP024c LAP025a LAP026c LAP026d LAP029b LAP030a LAP030b LAP062a LAP062d LAP067a LAP067c LAP067d
0 0 0 0 0 0 0 0 0 0 0 0 0 0 0 0 0 0 0 0 0 0 0 0
LAP072b LAP072c LAP072d LAP074b LAP074c LAP074d LAP083a LAP083c LAP083d LAP084c LAP084d LAP085a LAP085c LAP085d LAP086a LAP086c LAP086d LAP087a LAP087c LAP087d LAP088a LAP091a LAP091b LAP091d
0 0 0 0 0 0 0 0 0 0 0 0 0 0 0 0 0 0 0 0 0 0 0 0
LAP099b LAP100b LAP100c LAP100d LAP103a LAP104b LAP106d LAP108a LAP108d LAP111c LAP111d LAP112d LAP113b LAP113c LAP117b LAP117d LAP118b LAP118c LAP119c LAP119d LAP126a LAP126d K D
0 0 0 0 0 0 0 0 0 0 0 0 0 0 0 0 0 0 0 0 0 0 0 0

PR_48

PRFTc2_F2b TAGATTGCAGCCATAGACATTGACA

PRFTc2_R2c AGAGTCTGTAGGAGAAGCATCAATC

Plate 1

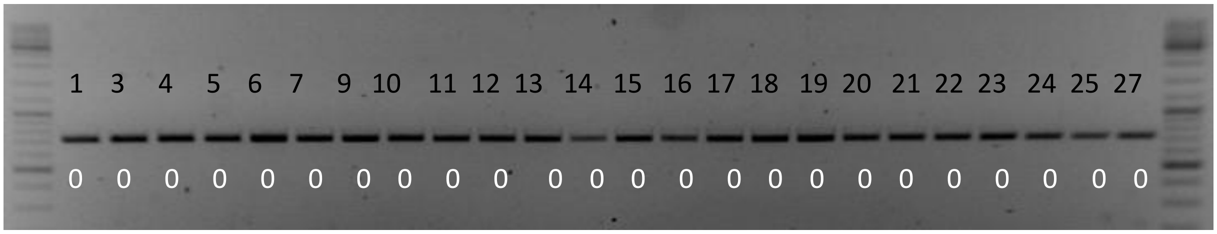

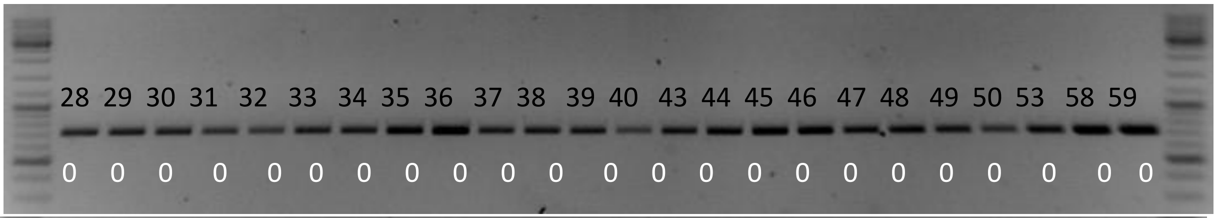

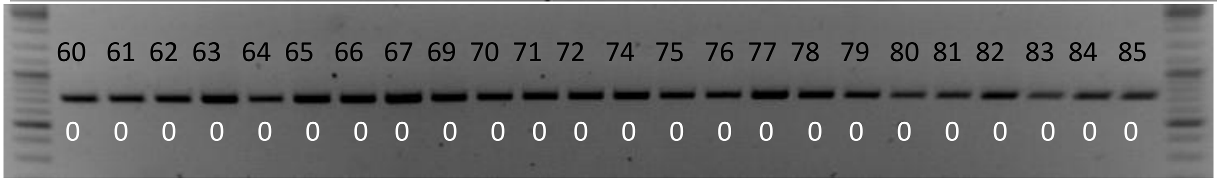

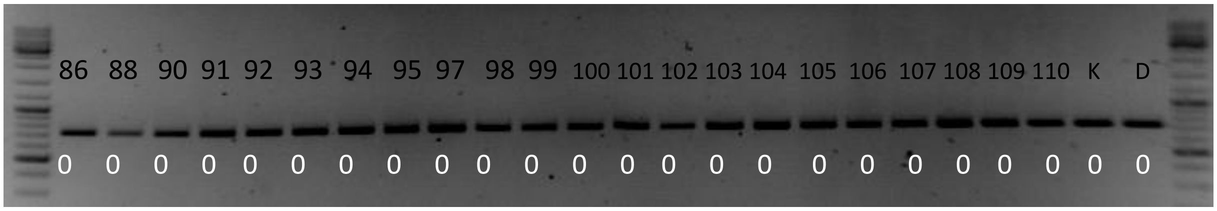

Plate 9

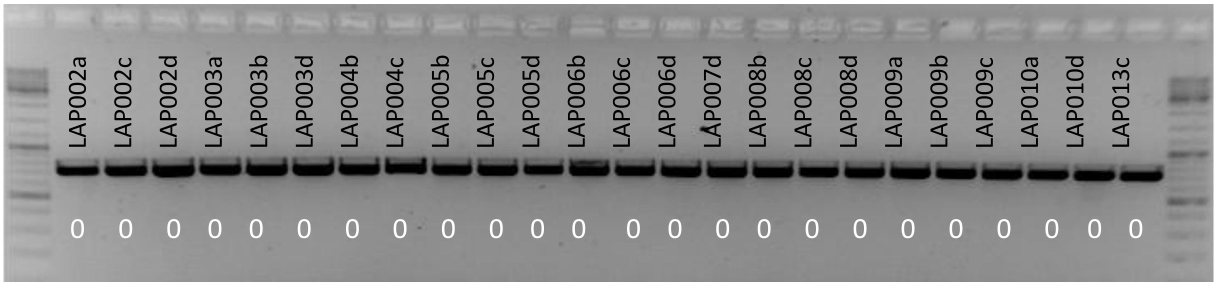
LAP002a
LAP002c
LAP002d
LAP003a
LAP003b
LAP003d
LAP004b
LAP004c
LAP005b
LAP005c
LAP005d
LAP006b
LAP006c
LAP006d
LAP007d
LAP008b
LAP008c
LAP008d
LAP009a
LAP009b
LAP009c
LAP010a
LAP010d
LAP013c
0 0 0 0 0 0 0 0 0 0 0 0 0 0 0 0 0 0 0 0 0 0 0 0

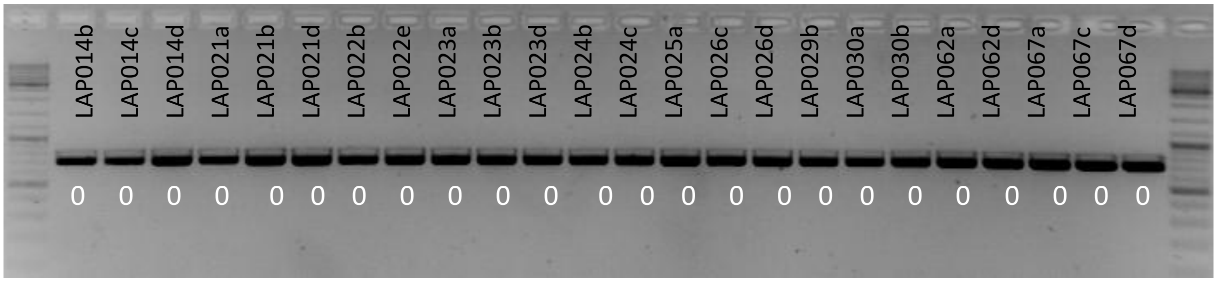
LAP014b
LAP014c
LAP014d
LAP021a
LAP021b
LAP021d
LAP022b
LAP022e
LAP023a
LAP023b
LAP023d
LAP024b
LAP024c
LAP025a
LAP026c
LAP026d
LAP029b
LAP030a
LAP030b
LAP062a
LAP062d
LAP067a
LAP067c
LAP067d
0 0 0 0 0 0 0 0 0 0 0 0 0 0 0 0 0 0 0 0 0 0 0 0

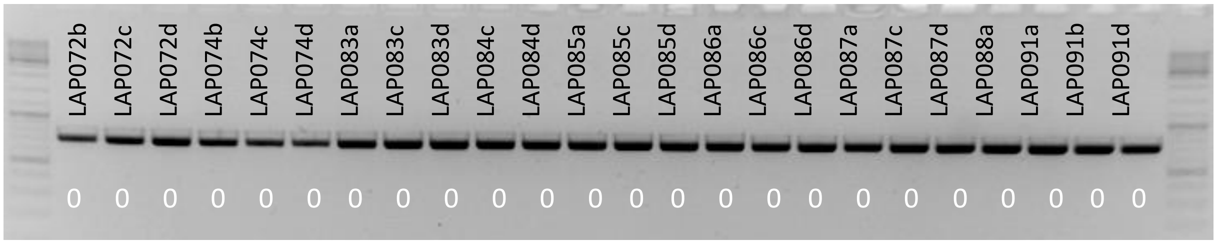
LAP072b
LAP072c
LAP072d
LAP074b
LAP074c
LAP074d
LAP083a
LAP083c
LAP083d
LAP084c
LAP084d
LAP085a
LAP085c
LAP085d
LAP086a
LAP086c
LAP086d
LAP087a
LAP087c
LAP087d
LAP088a
LAP091a
LAP091b
LAP091d
0 0 0 0 0 0 0 0 0 0 0 0 0 0 0 0 0 0 0 0 0 0 0 0

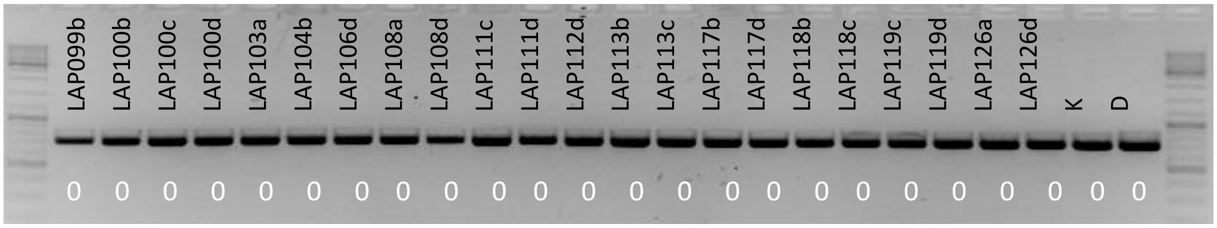
LAP099b
LAP100b
LAP100c
LAP100d
LAP103a
LAP104b
LAP106d
LAP108a
LAP108d
LAP111c
LAP111d
LAP112d
LAP113b
LAP113c
LAP117b
LAP117d
LAP118b
LAP118c
LAP119c
LAP119d
LAP126a
LAP126d
K
D
0 0 0 0 0 0 0 0 0 0 0 0 0 0 0 0 0 0 0 0 0 0 0 0

PR_49

PRFTc2_F2c ACCTTGATGAGTTCTATGGCTTCTT

PRFTC2R2 TGGTTGCCAAAGGTTTCCCA

Plate 1

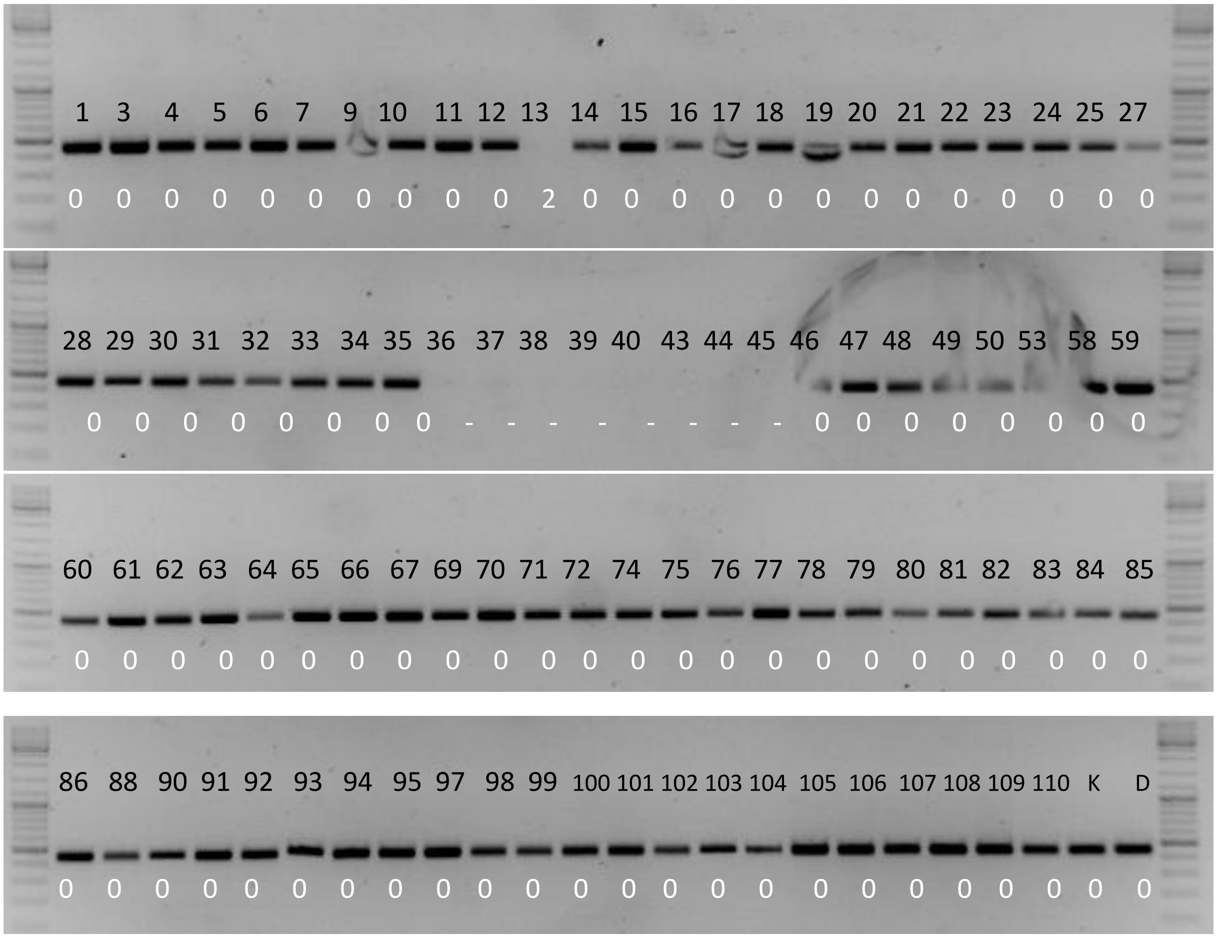

Plate 9

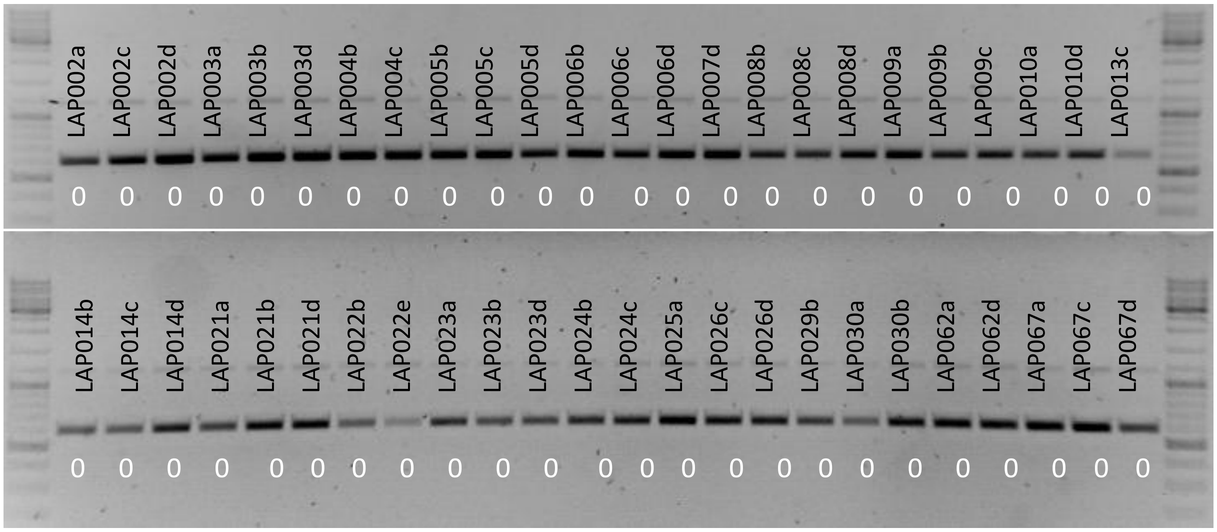

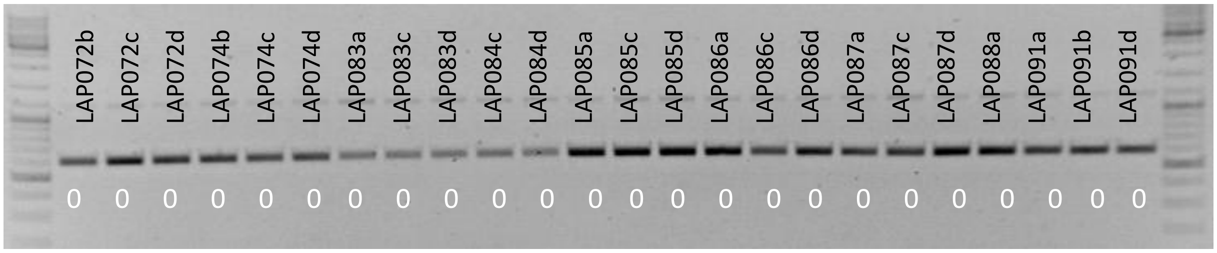

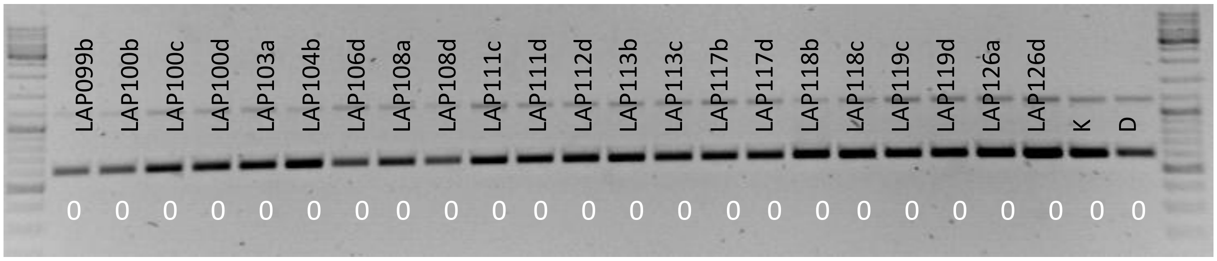

Repeat

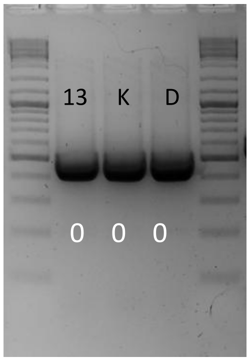

PR_50

PRFTC2F3 CAATTCTTAGAGCTTGCAACTG

PRFTc2_R3b TGTTTGGGTCAATTTGCTTTAGACA

Plate 1

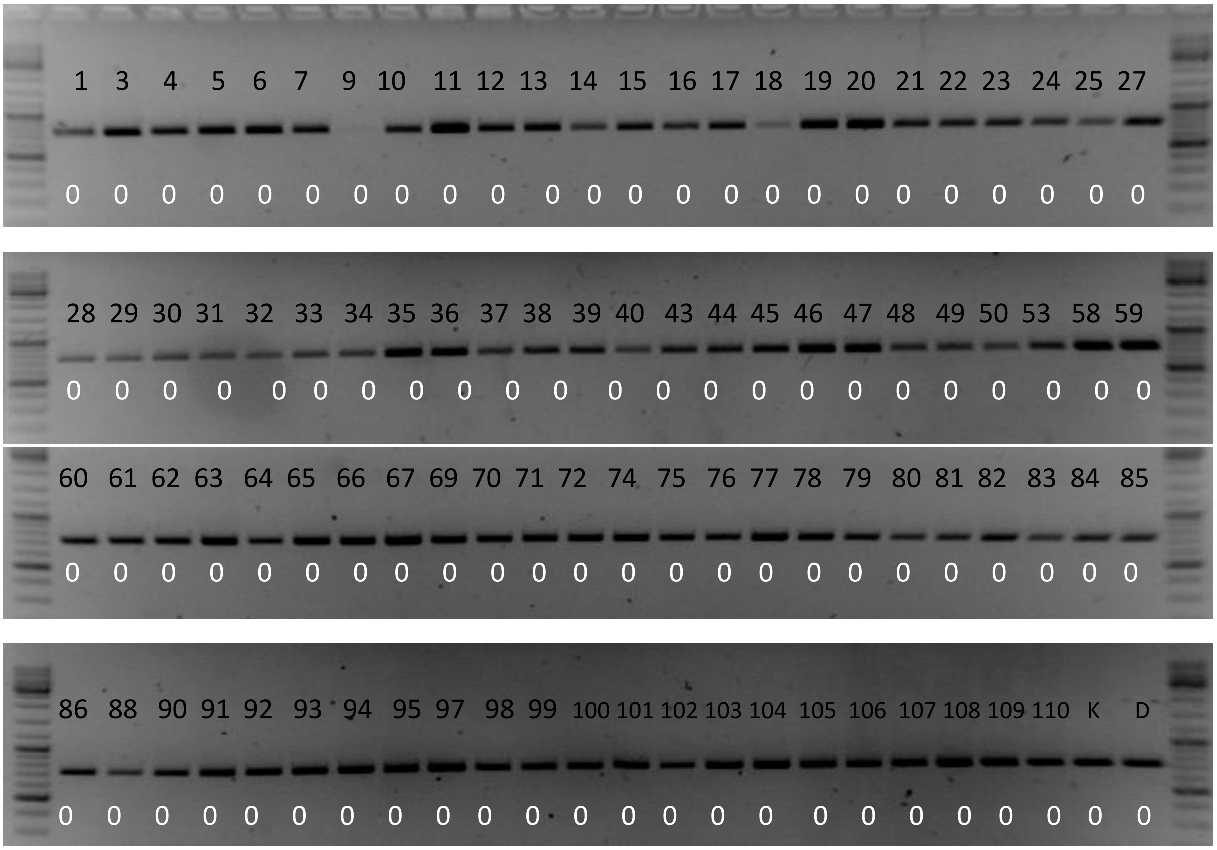

Plate 9

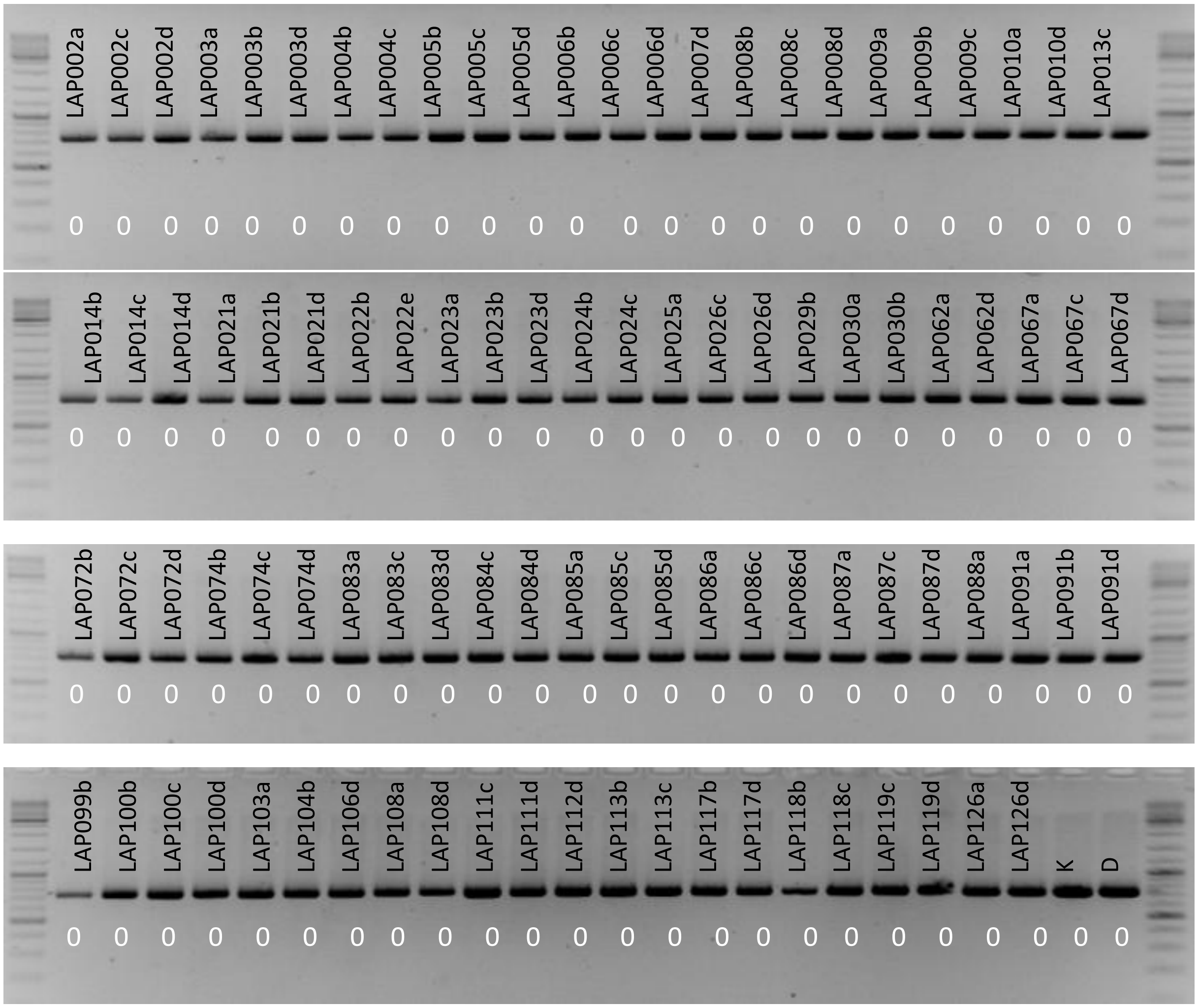
LAP002a LAP002c LAP002d LAP003a LAP003b LAP003d LAP004b LAP004c LAP005b LAP005c LAP005d LAP006b LAP006c LAP006d LAP007d LAP008b LAP008c LAP008d LAP009a LAP009b LAP009c LAP010a LAP010d LAP013c
0 0 0 0 0 0 0 0 0 0 0 0 0 0 0 0 0 0 0 0 0 0 0 0
LAP014b LAP014c LAP014d LAP021a LAP021b LAP021d LAP022b LAP022e LAP023a LAP023b LAP023d LAP024b LAP024c LAP025a LAP026c LAP026d LAP029b LAP030a LAP030b LAP062a LAP062d LAP067a LAP067c LAP067d
0 0 0 0 0 0 0 0 0 0 0 0 0 0 0 0 0 0 0 0 0 0 0 0
LAP072b LAP072c LAP072d LAP074b LAP074c LAP074d LAP083a LAP083c LAP083d LAP084c LAP084d LAP085a LAP085c LAP085d LAP086a LAP086c LAP086d LAP087a LAP087c LAP087d LAP088a LAP091a LAP091b LAP091d
0 0 0 0 0 0 0 0 0 0 0 0 0 0 0 0 0 0 0 0 0 0 0 0
LAP099b LAP100b LAP100c LAP100d LAP103a LAP104b LAP106d LAP108a LAP108d LAP111c LAP111d LAP112d LAP113b LAP113c LAP117b LAP117d LAP118b LAP118c LAP119c LAP119d LAP126a LAP126d K D
0 0 0 0 0 0 0 0 0 0 0 0 0 0 0 0 0 0 0 0 0 0 0 0

PR_51

PRFTc2_F3b CCCTGAAGTGTGATATCCTAGGAAG

PRFTc2_R3c AGAATTGATATGGTGGTTGGTTTGC

Plate 1

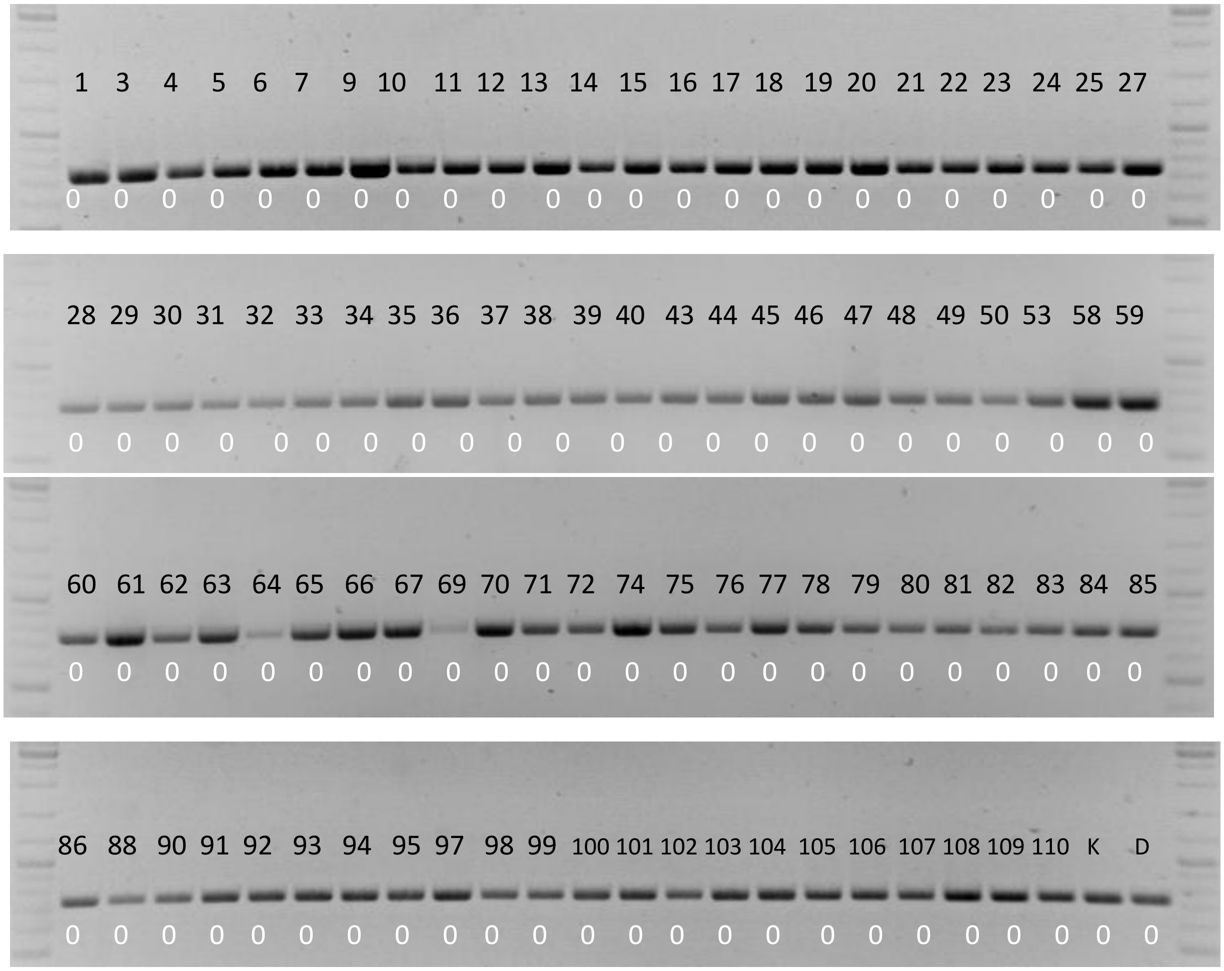

Plate 9

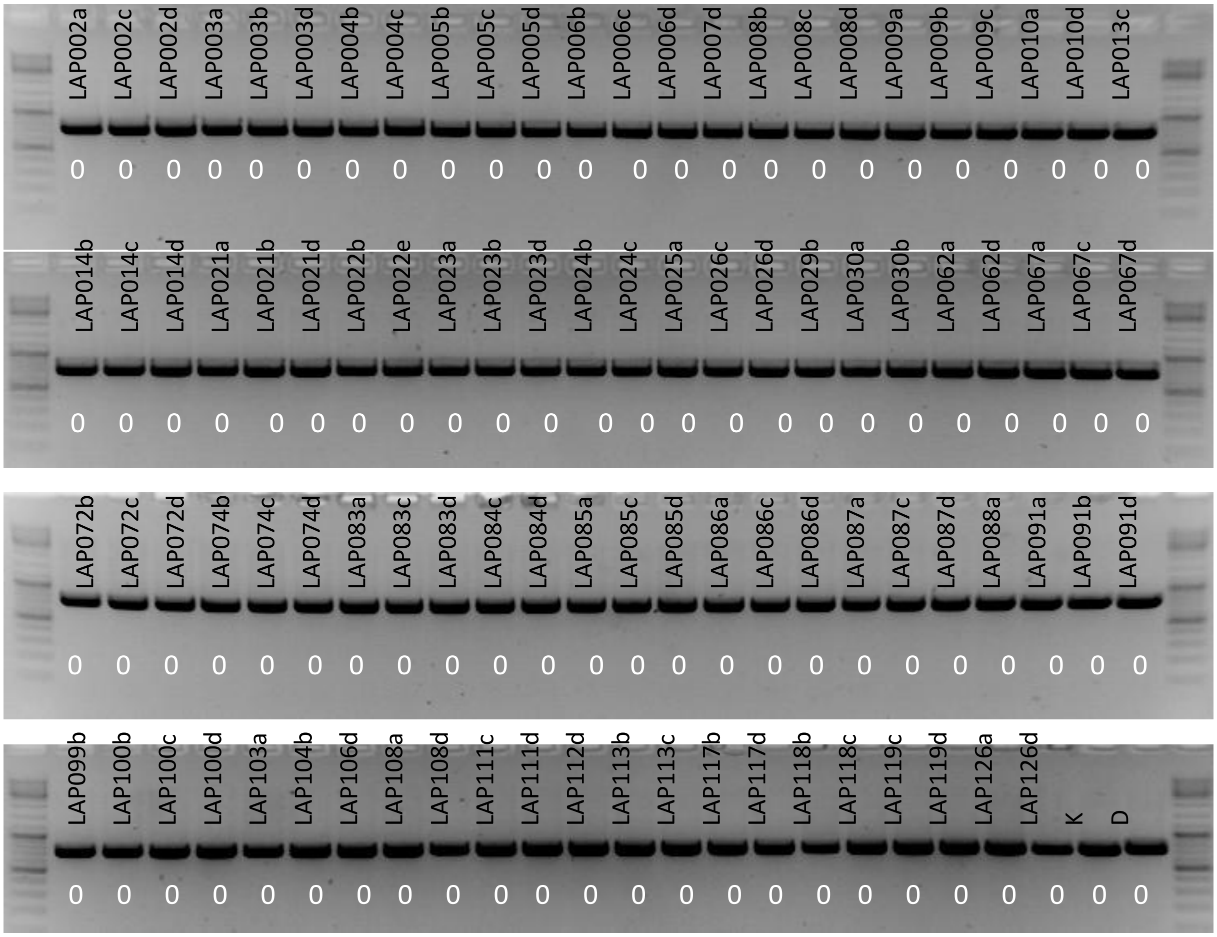

LAP002a LAP002c LAP002d LAP003a LAP003b LAP003d LAP004b LAP004c LAP005b LAP005c LAP005d LAP006b LAP006c LAP006d LAP007d LAP008b LAP008c LAP008d LAP009a LAP009b LAP009c LAP010a LAP010d LAP013c
0 0 0 0 0 0 0 0 0 0 0 0 0 0 0 0 0 0 0 0 0 0 0 0
LAP014b LAP014c LAP014d LAP021a LAP021b LAP021d LAP022b LAP022e LAP023a LAP023b LAP023d LAP024b LAP024c LAP025a LAP026c LAP026d LAP029b LAP030a LAP030b LAP062a LAP062d LAP067a LAP067c LAP067d
0 0 0 0 0 0 0 0 0 0 0 0 0 0 0 0 0 0 0 0 0 0 0 0
LAP072b LAP072c LAP072d LAP074b LAP074c LAP074d LAP083a LAP083c LAP083d LAP084c LAP084d LAP085a LAP085c LAP085d LAP086a LAP086c LAP086d LAP087a LAP087c LAP087d LAP088a LAP091a LAP091b LAP091d
0 0 0 0 0 0 0 0 0 0 0 0 0 0 0 0 0 0 0 0 0 0 0 0
LAP099b LAP100b LAP100c LAP100d LAP103a LAP104b LAP106d LAP108a LAP108d LAP111c LAP111d LAP112d LAP113b LAP113c LAP117b LAP117d LAP118b LAP118c LAP119c LAP119d LAP126a LAP126d K D
0 0 0 0 0 0 0 0 0 0 0 0 0 0 0 0 0 0 0 0 0 0 0 0

PR_52

PRFTc2_F3c GAACTTATTCCTTGTCTGCTTGCTT

PRFTC2R3 TGGTATCTTGATACAGGGTGTTCC

Plate 1

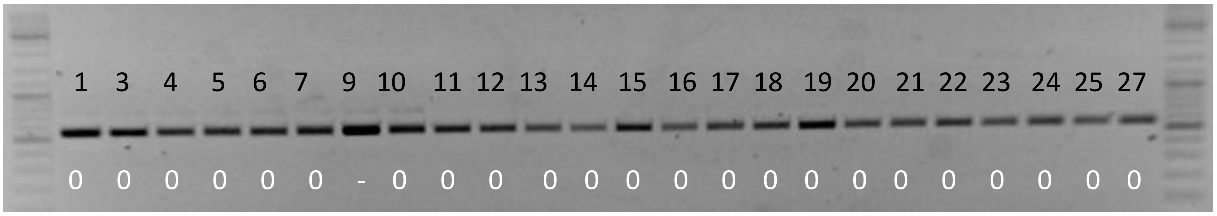

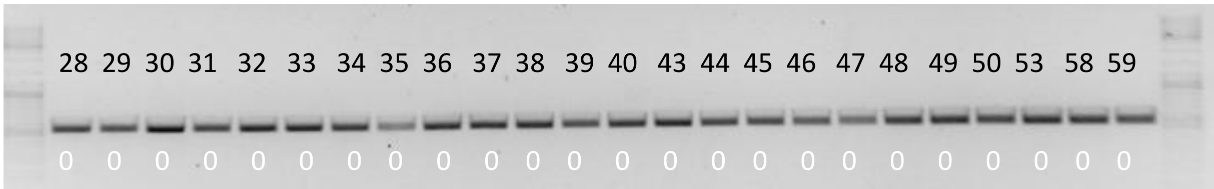

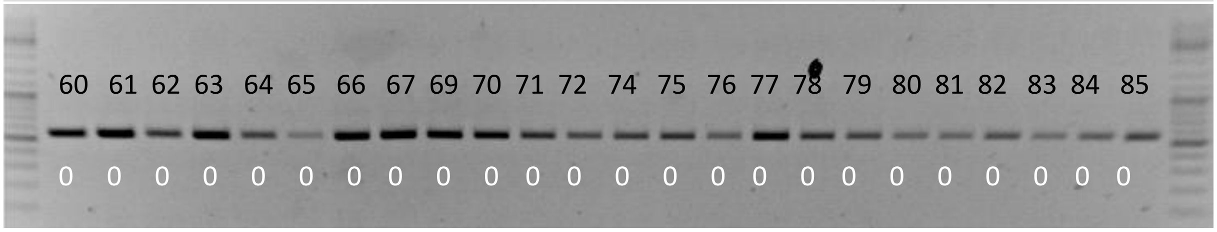

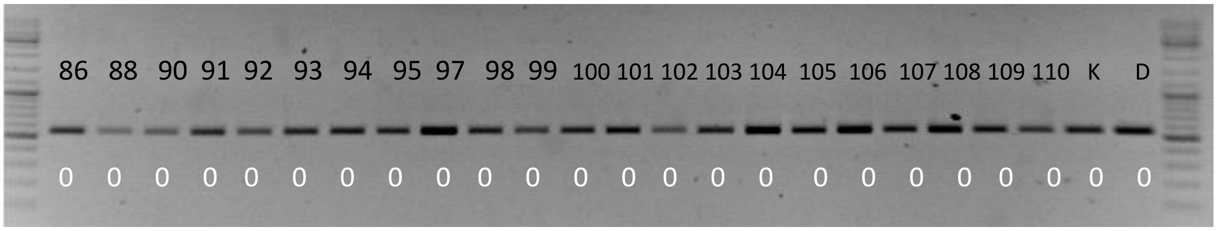

Plate 9

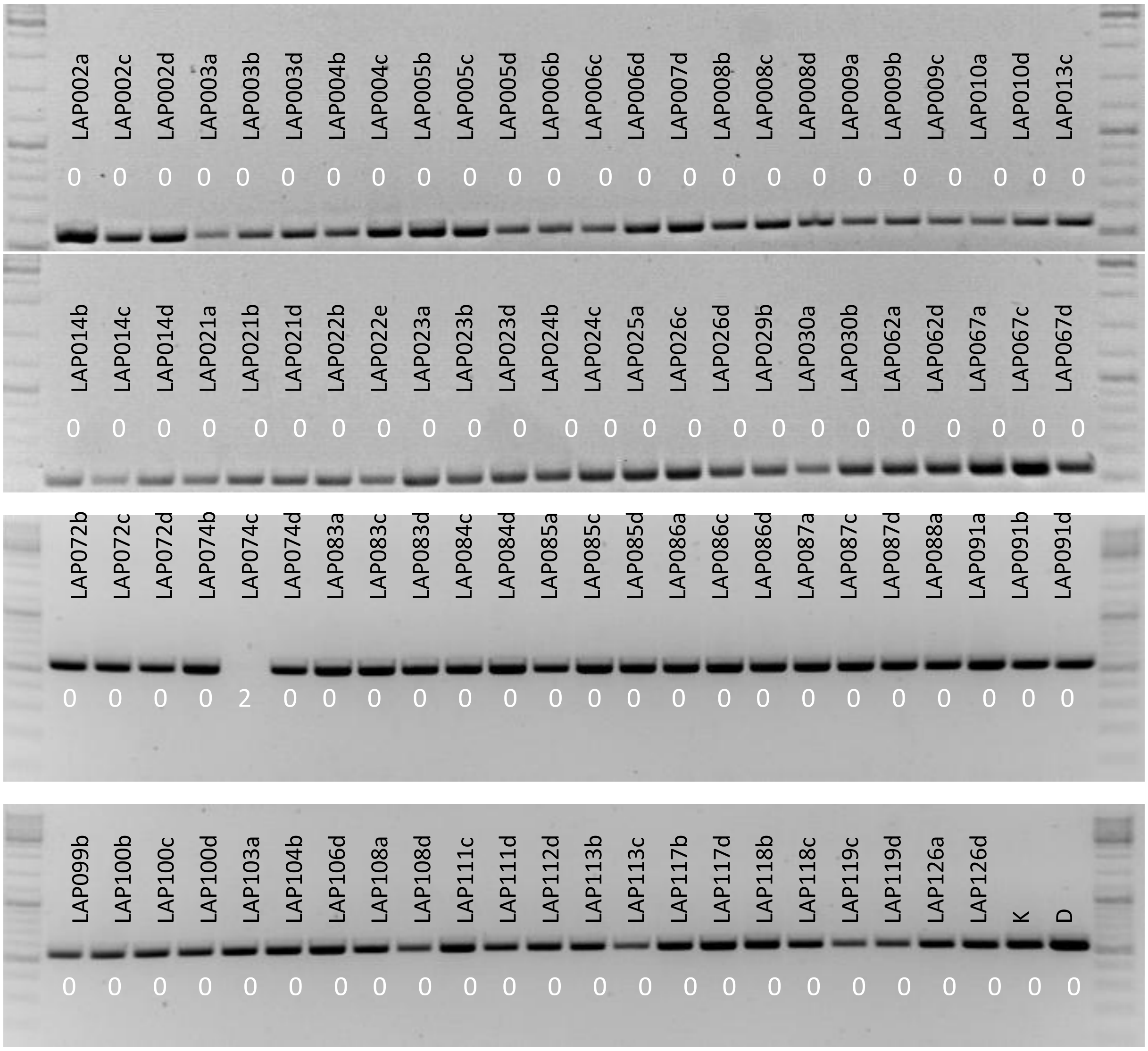

Repeat

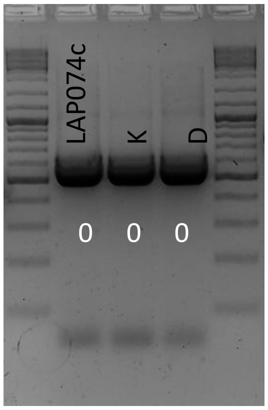

PR_53

PRFTC2F4 GCTATCATCTGCAAACTTGACCT

PRFTc2_R4b AAACTCATGGTGAATAAGTGGCAGA

Plate 1

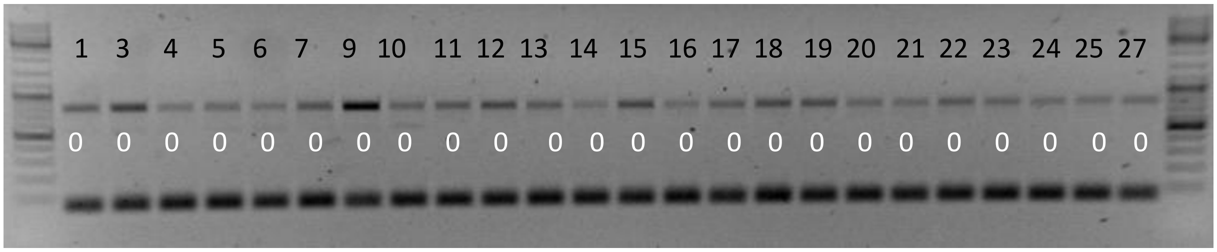

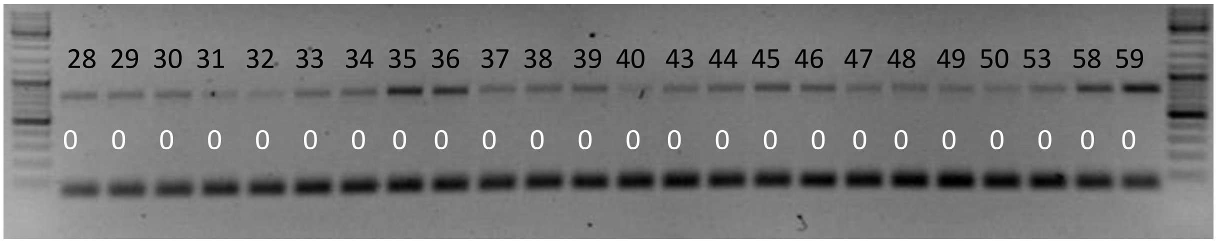

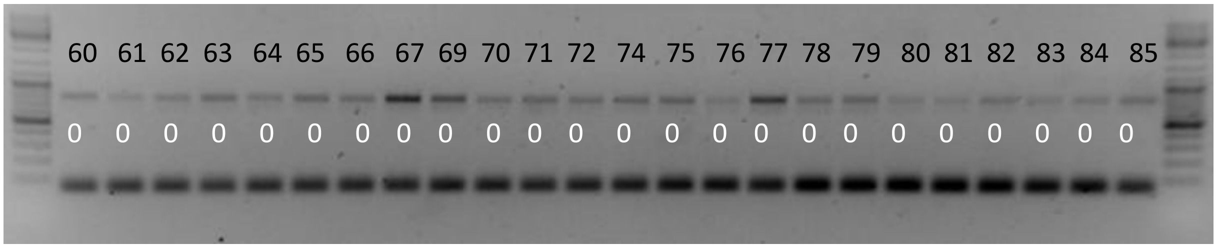

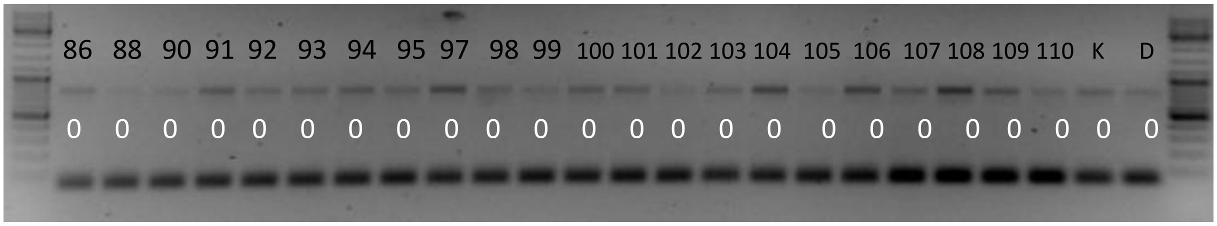

Plate 9

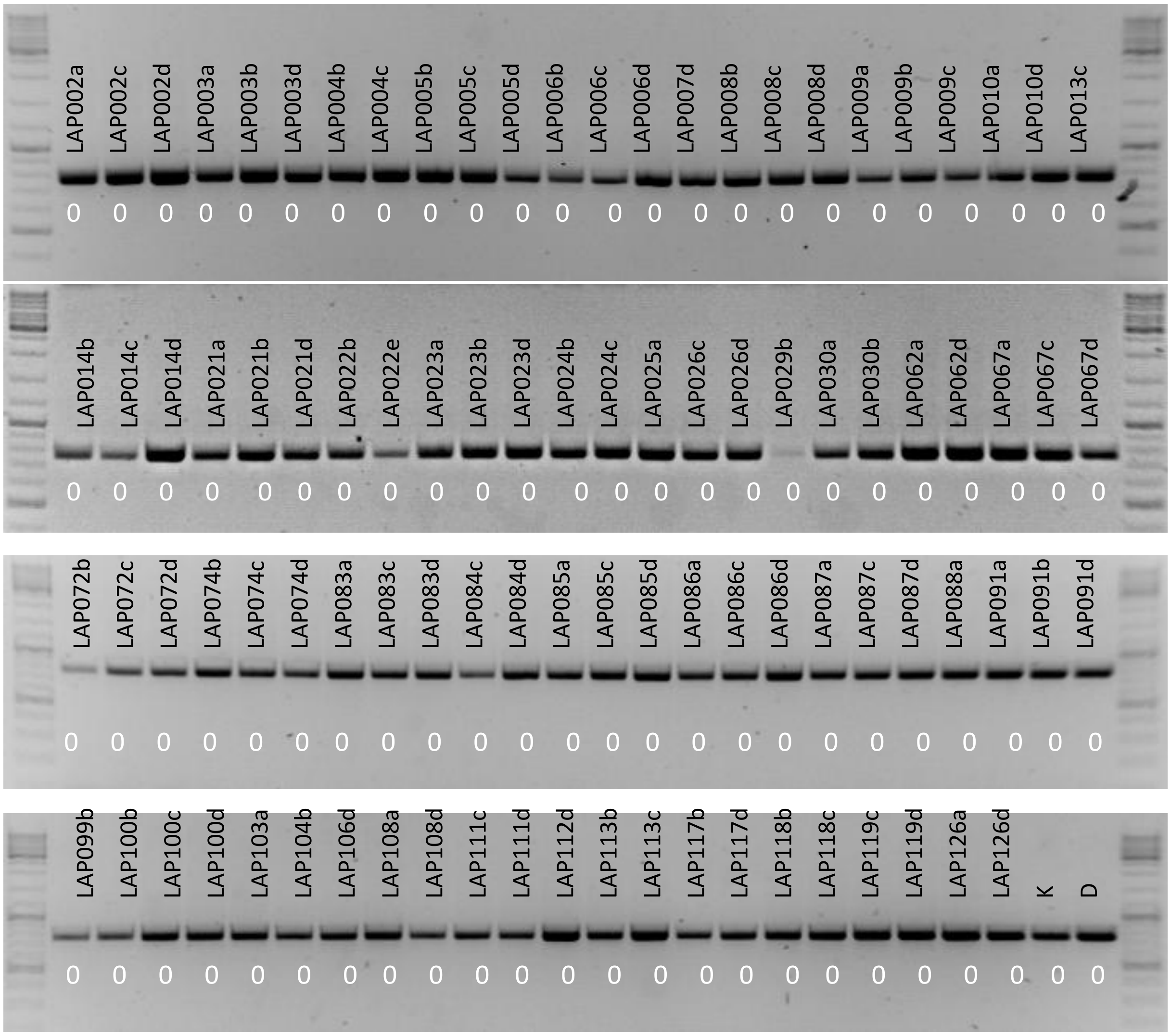
LAP002a LAP002c LAP002d LAP003a LAP003b LAP003d LAP004b LAP004c LAP005b LAP005c LAP005d LAP006b LAP006c LAP006d LAP007d LAP008b LAP008c LAP008d LAP009a LAP009b LAP009c LAP010a LAP010d LAP013c
0 0 0 0 0 0 0 0 0 0 0 0 0 0 0 0 0 0 0 0 0 0 0 0
LAP014b LAP014c LAP014d LAP021a LAP021b LAP021d LAP022b LAP022e LAP023a LAP023b LAP023d LAP024b LAP024c LAP025a LAP026c LAP026d LAP029b LAP030a LAP030b LAP062a LAP062d LAP067a LAP067c LAP067d
0 0 0 0 0 0 0 0 0 0 0 0 0 0 0 0 0 0 0 0 0 0 0 0
LAP072b LAP072c LAP072d LAP074b LAP074c LAP074d LAP083a LAP083c LAP083d LAP084c LAP084d LAP085a LAP085c LAP085d LAP086a LAP086c LAP086d LAP087a LAP087c LAP087d LAP088a LAP091a LAP091b LAP091d
0 0 0 0 0 0 0 0 0 0 0 0 0 0 0 0 0 0 0 0 0 0 0 0
LAP099b LAP100b LAP100c LAP100d LAP103a LAP104b LAP106d LAP108a LAP108d LAP111c LAP111d LAP112d LAP113b LAP113c LAP117b LAP117d LAP118b LAP118c LAP119c LAP119d LAP126a LAP126d K D
0 0 0 0 0 0 0 0 0 0 0 0 0 0 0 0 0 0 0 0 0 0 0 0

PR_54

PRFTc2_F4b TTTGACTCCTCAATTGCAACAATCA

PRFTc2_R4c AATCTCTCAACTCTCTGCAACTTTC

Plate 1

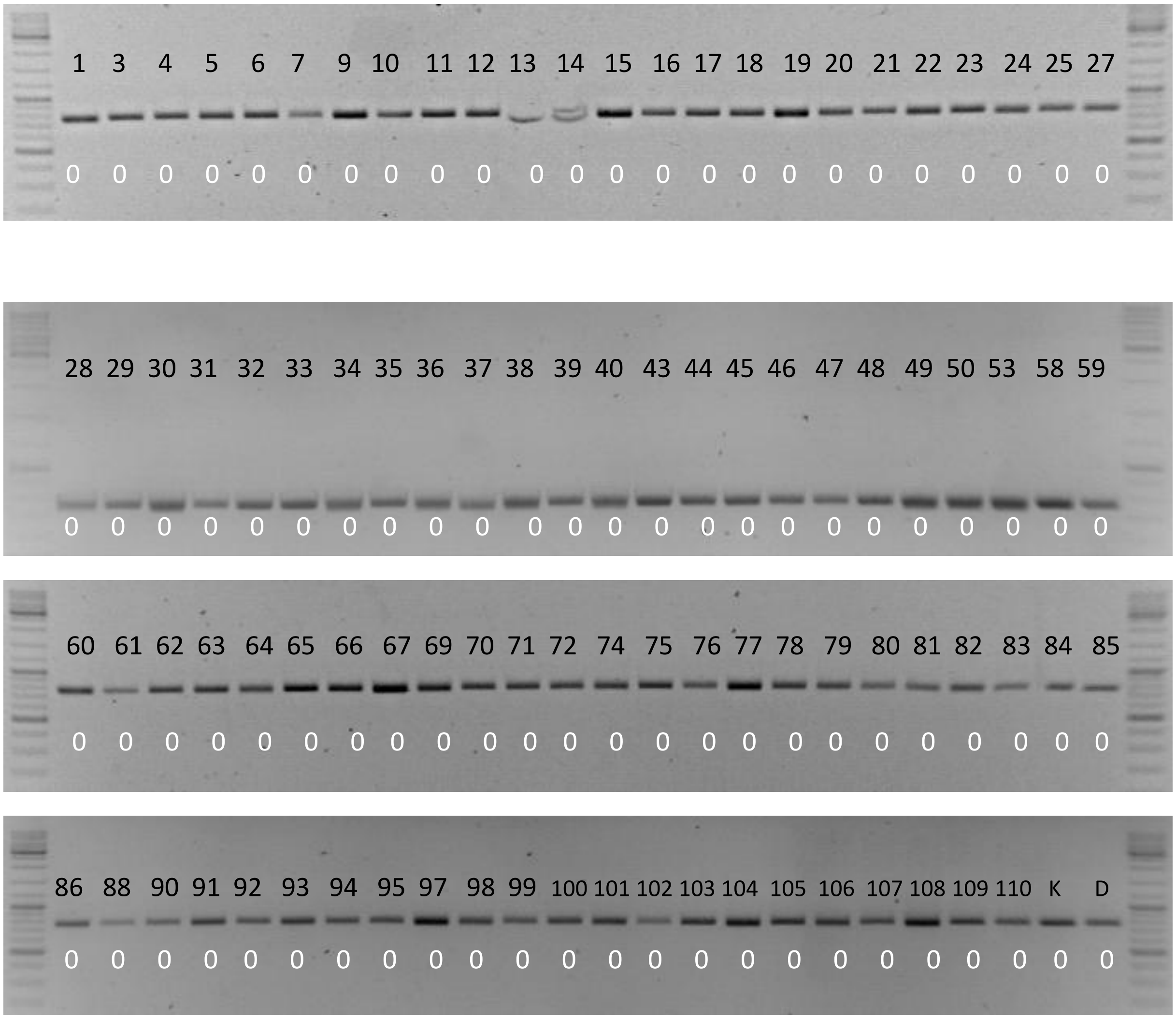

Plate 9

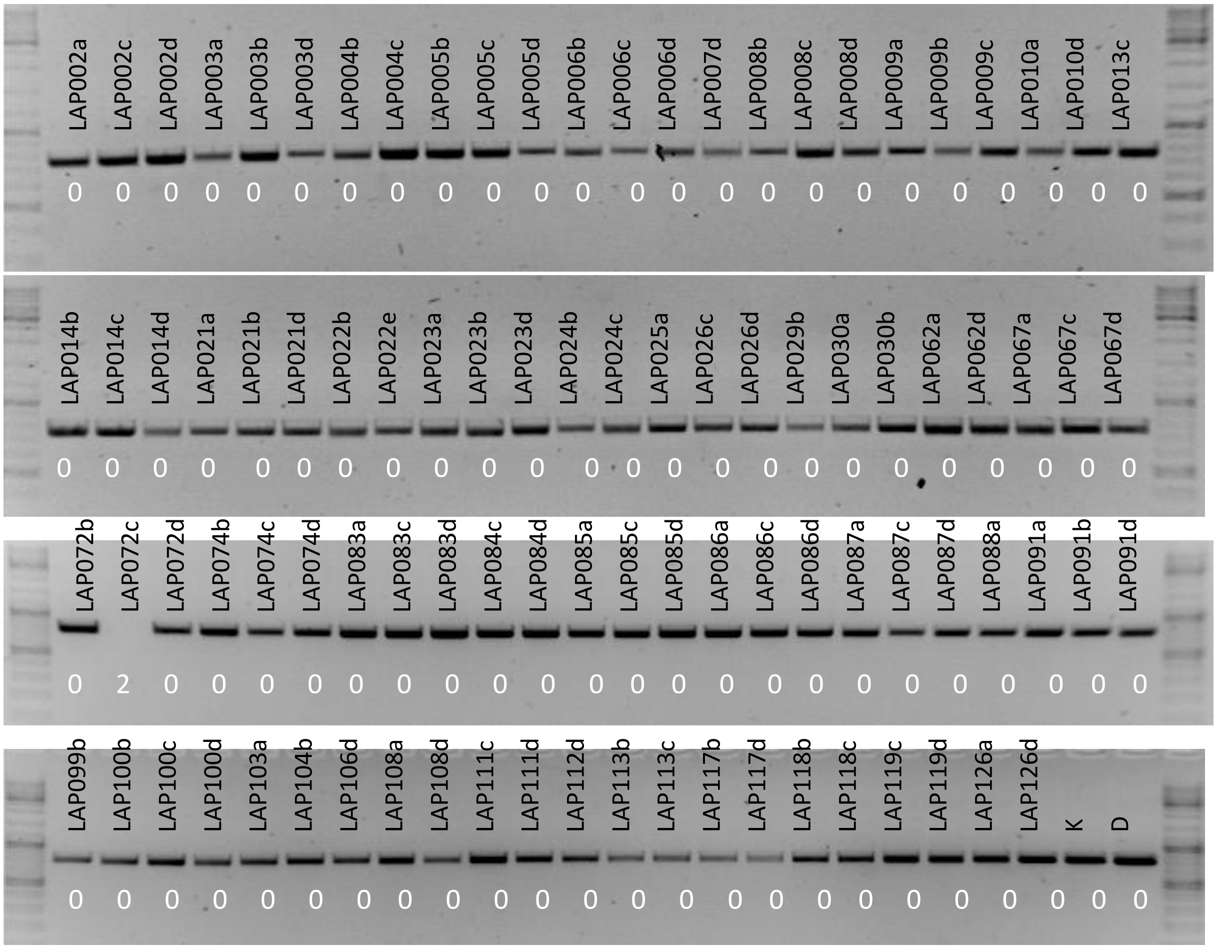

Repeat

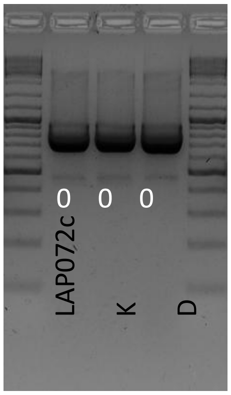

PR_55

PRFTc2_F4c GATGGCTCTTGATACCACTGTTAGA

PRFTC2R4 TGCCAGTAAAATGCATGAGAAGA

Plate 1

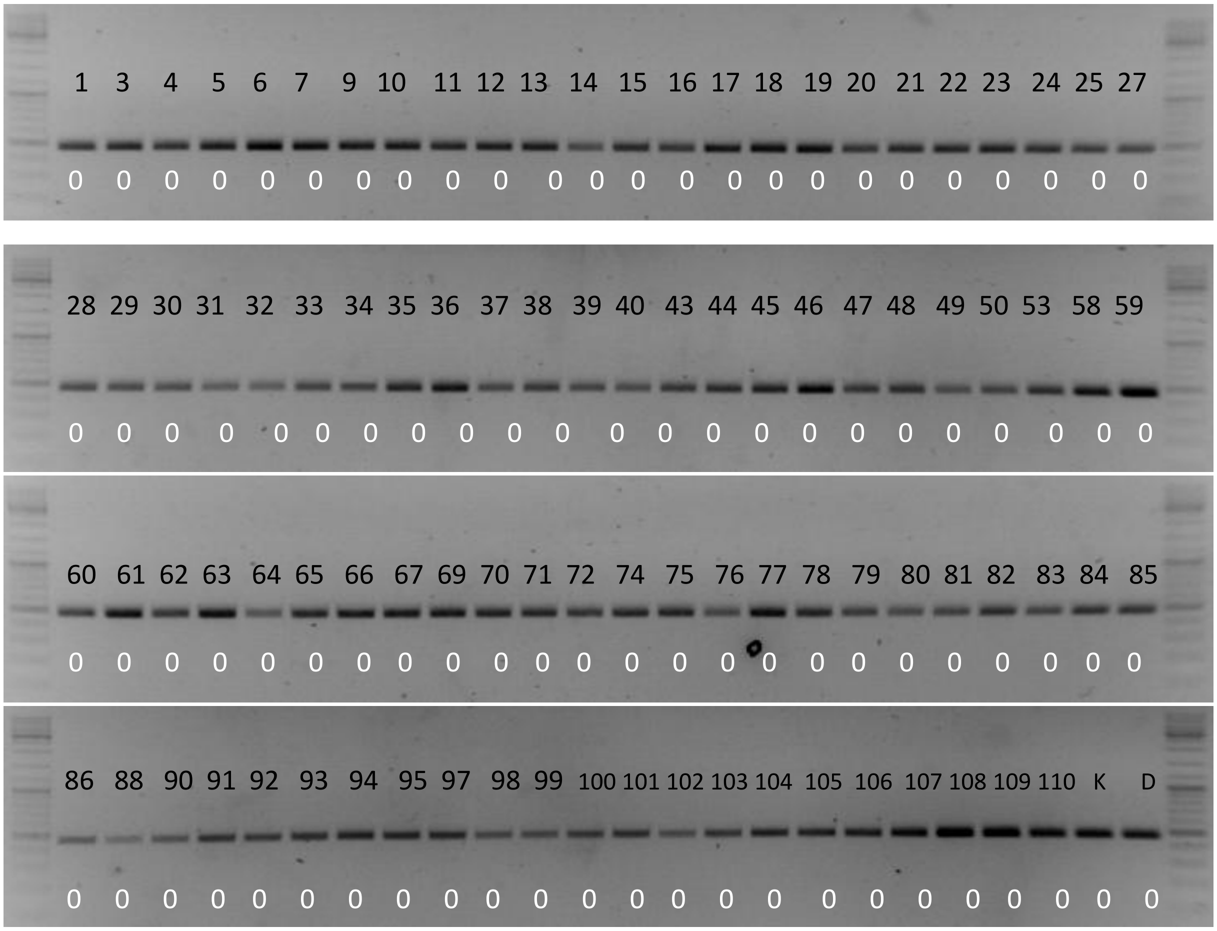

Plate 9

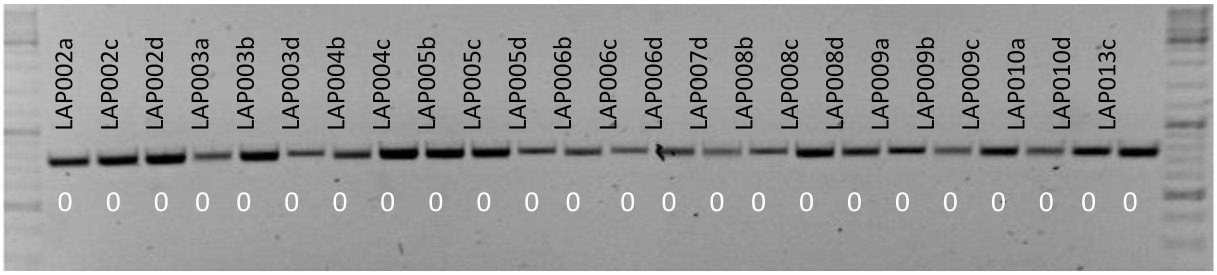

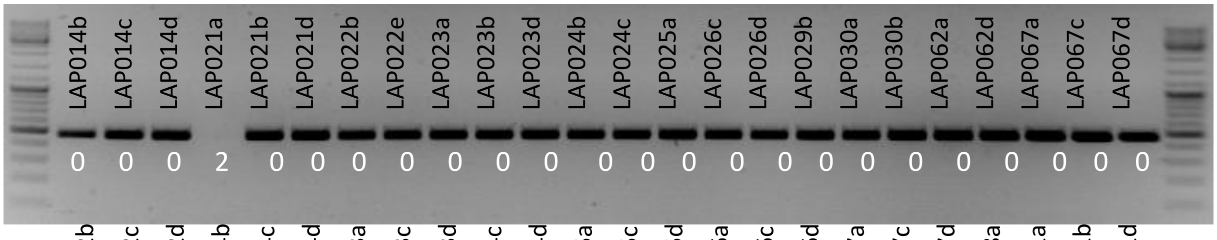

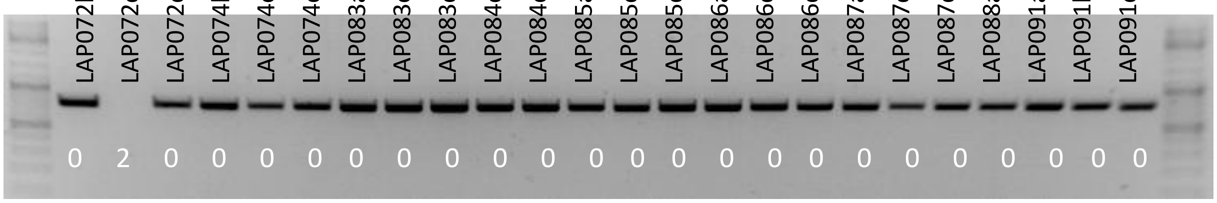

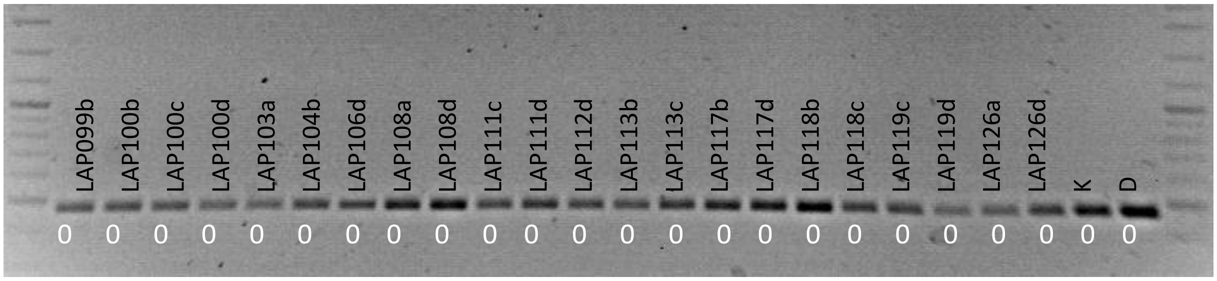

Repeat

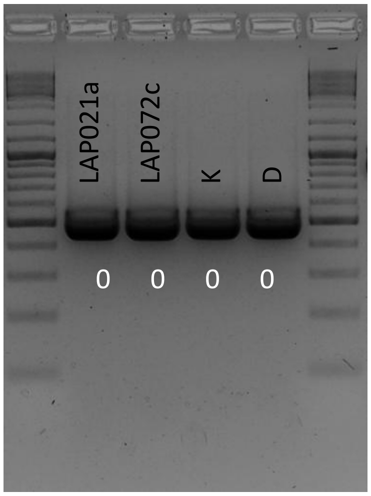

PR_56

PRFTC2F5 TCAAGATCCAGATAGCATAAGAGTT

PRFTc2_R5b CACAAACCAAACTAAATTTCTCTTACA

Plate 1

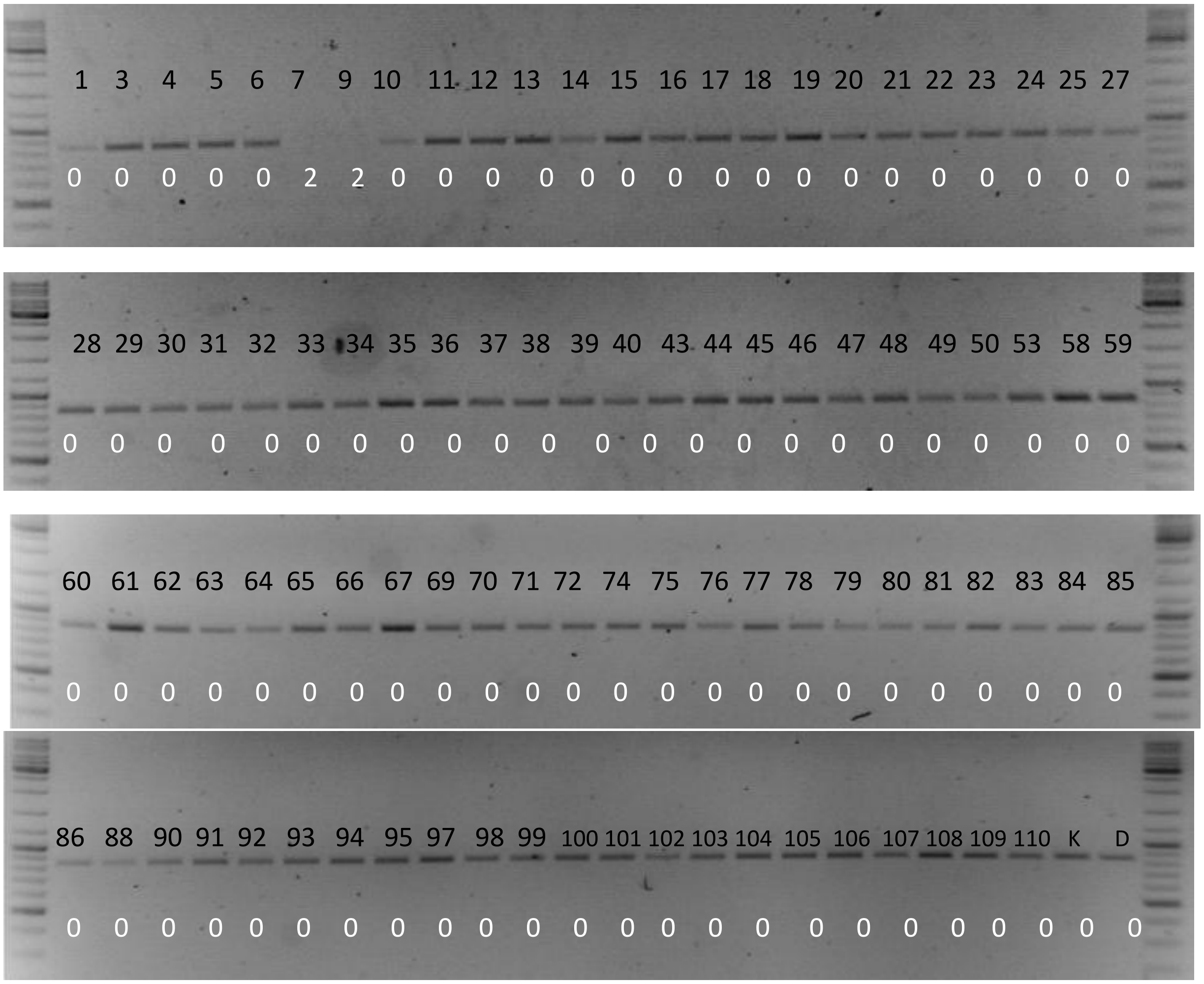

Plate 9

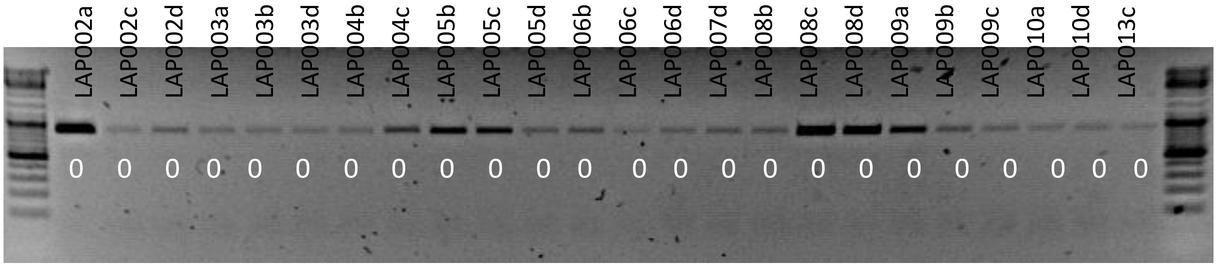

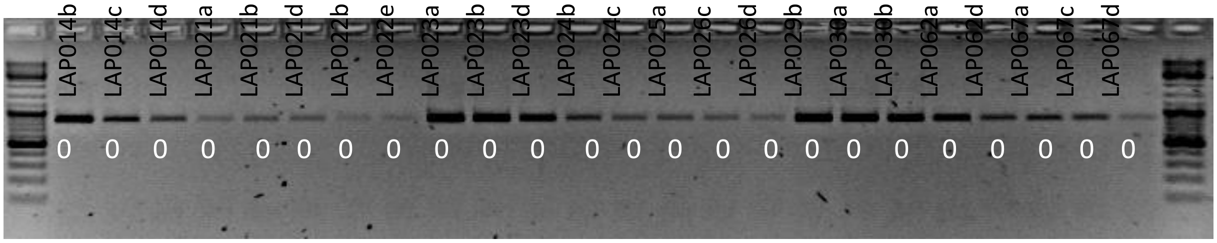

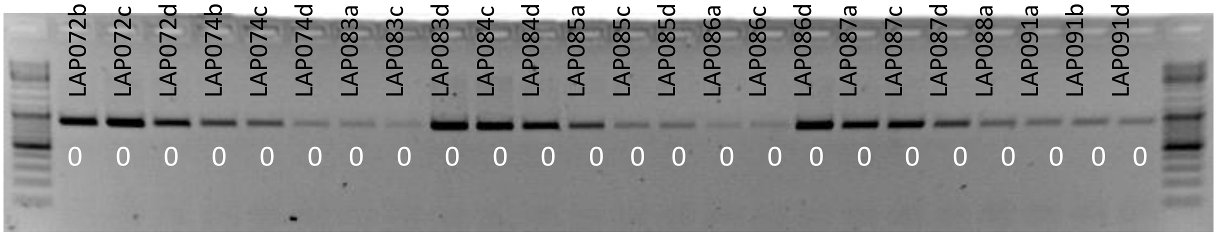

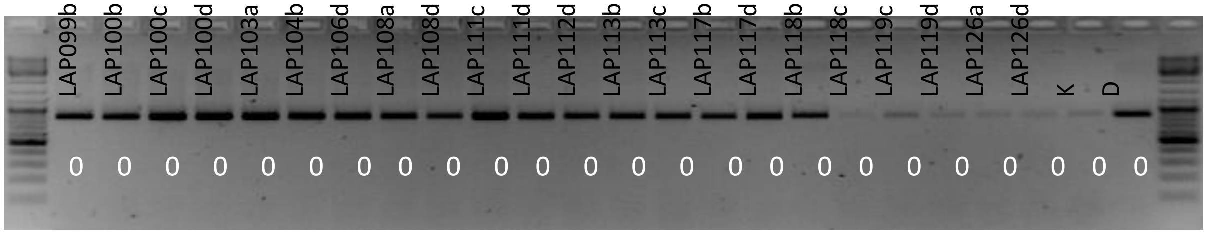

Repeat

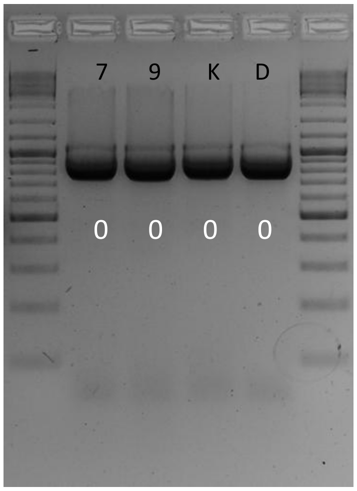

PR_57

PRFTc2_F5b GTTTGGTTTGTGAAATGGATGAAGC

PRFTC2R5 CCAACAGAAACTCTTGGTCGG

Plate 1

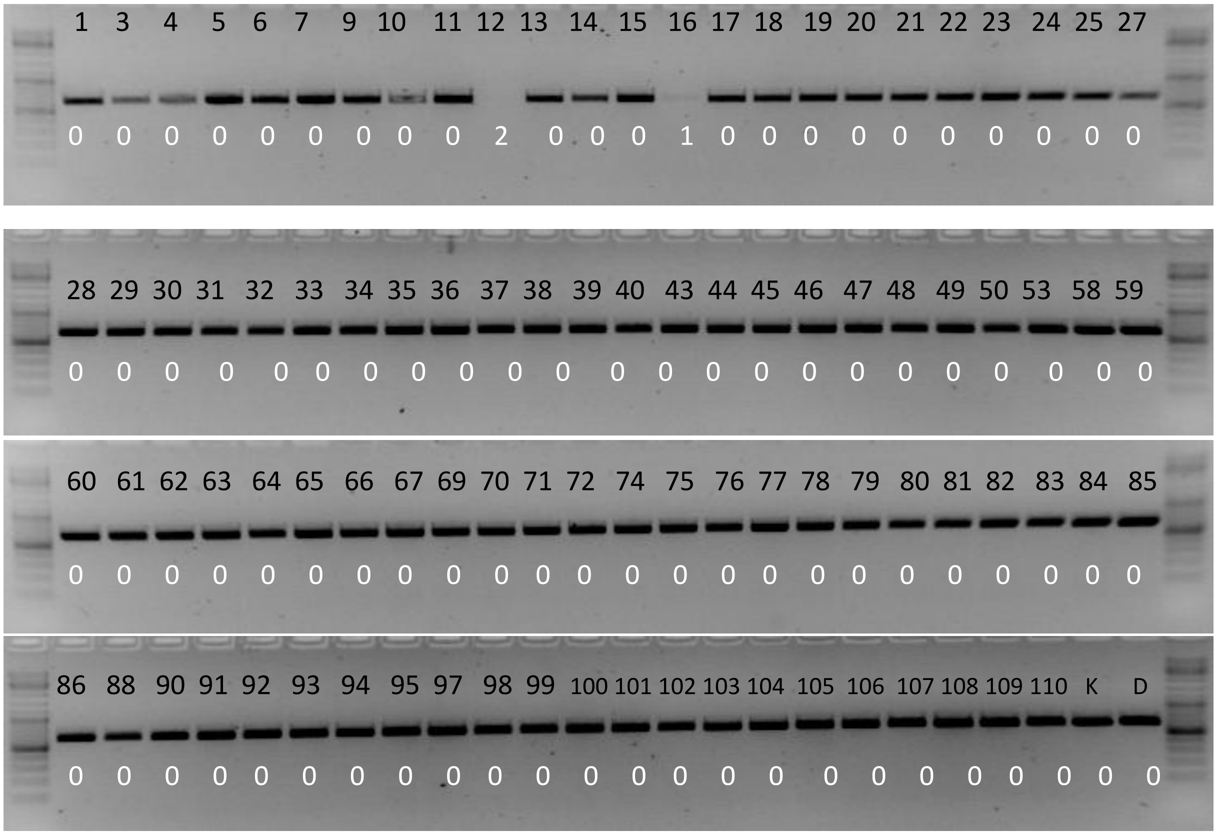

Plate 9

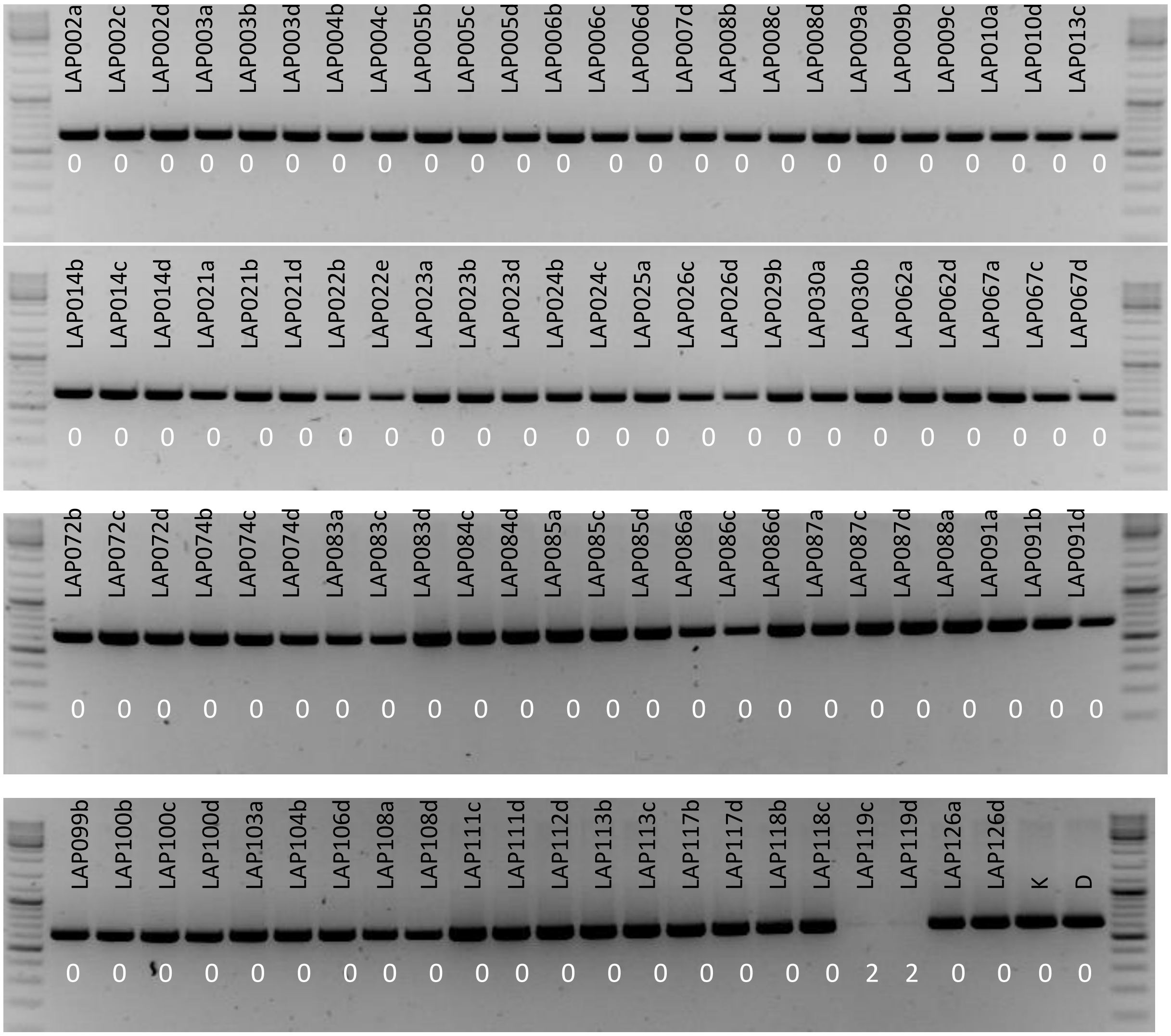

Repeat

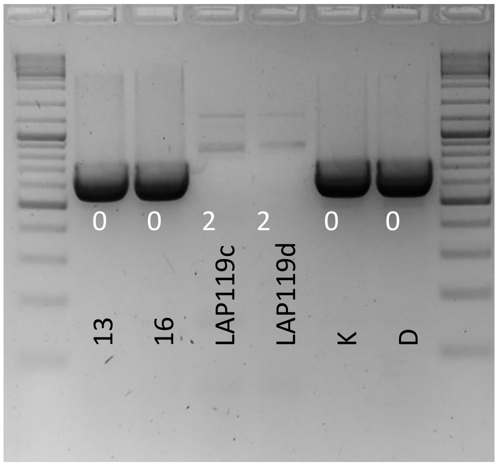

PR_64

FTc2_F5a AGTAGCTGGAATATCAGTCACCAT

PRFTC2R5 CCAACAGAAACTCTTGGTCGG

Plate 1

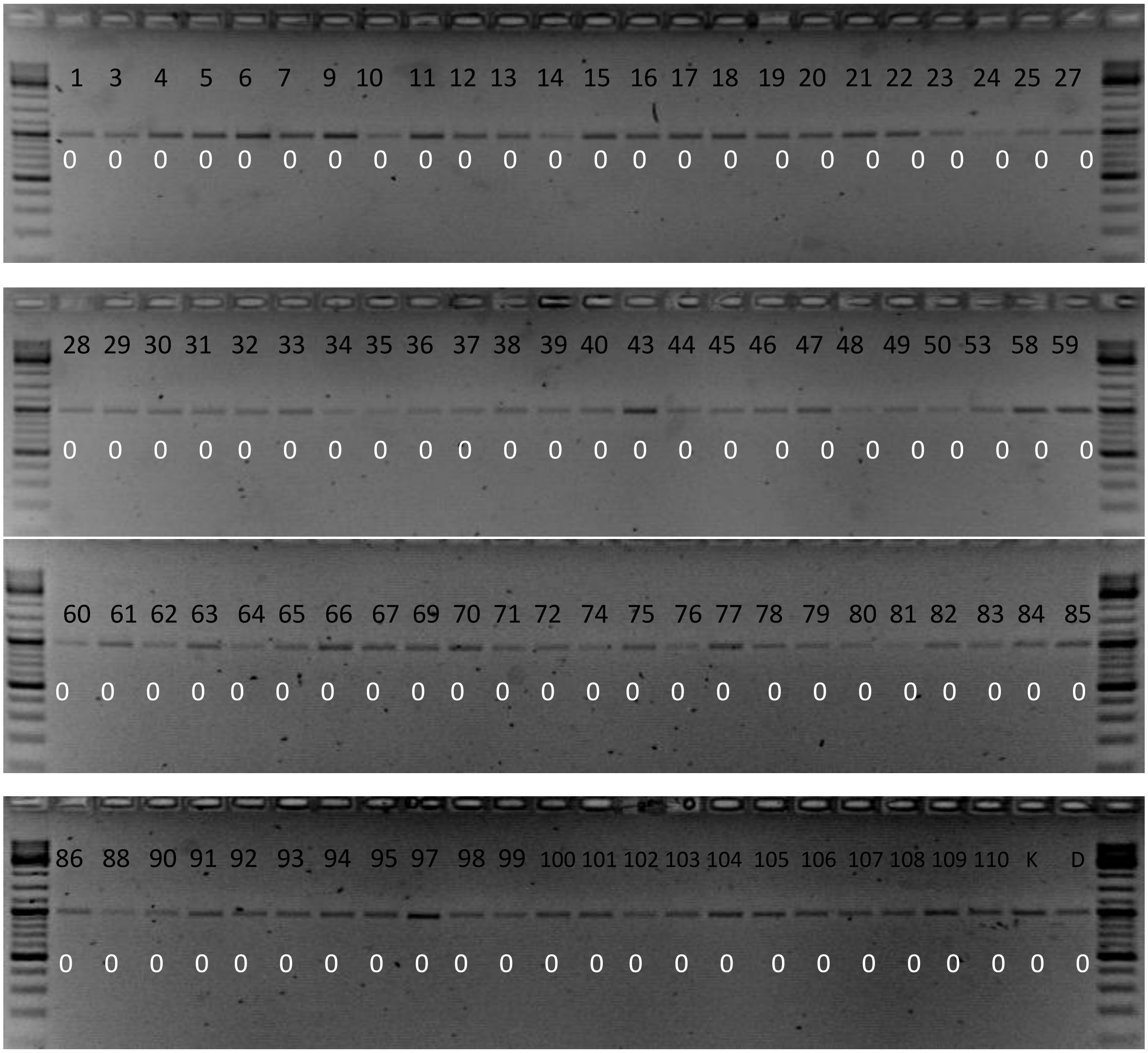

Plate 9

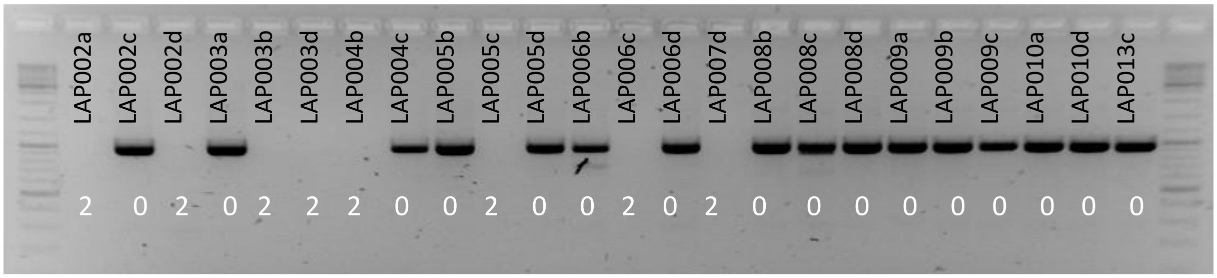
LAP002a LAP002c LAP002d LAP003a LAP003b LAP003d LAP004b LAP004c LAP005b LAP005c LAP005d LAP006b LAP006c LAP006d LAP007d LAP008b LAP008c LAP008d LAP009a LAP009b LAP009c LAP010a LAP010d LAP013c
2 0 2 0 2 2 2 0 0 2 0 0 2 0 2 0 0 0 0 0 0 0 0 0

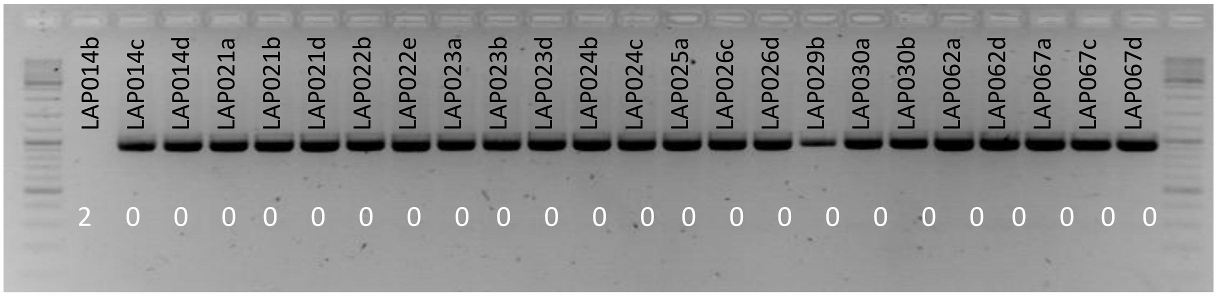
LAP014b LAP014c LAP014d LAP021a LAP021b LAP021d LAP022b LAP022e LAP023a LAP023b LAP023d LAP024b LAP024c LAP025a LAP026c LAP026d LAP029b LAP030a LAP030b LAP062a LAP062d LAP067a LAP067c LAP067d
2 0 0 0 0 0 0 0 0 0 0 0 0 0 0 0 0 0 0 0 0 0 0 0

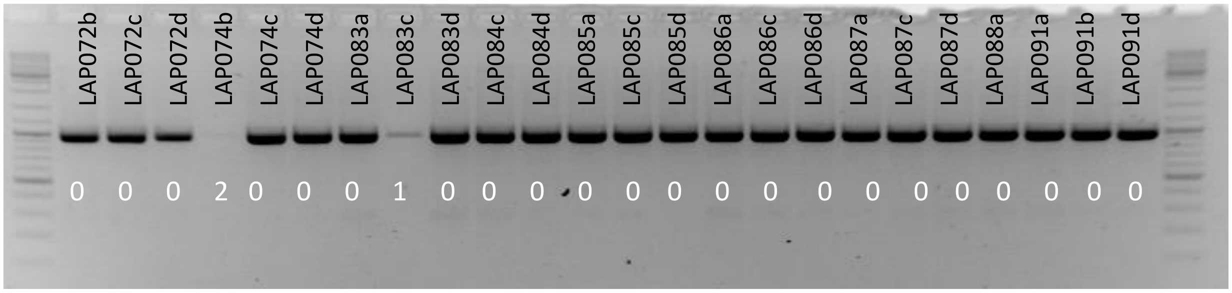
LAP072b LAP072c LAP072d LAP074b LAP074c LAP074d LAP083a LAP083c LAP083d LAP084c LAP084d LAP085a LAP085c LAP085d LAP086a LAP086c LAP086d LAP087a LAP087c LAP087d LAP088a LAP091a LAP091b LAP091d
0 0 0 2 0 0 0 1 0 0 0 0 0 0 0 0 0 0 0 0 0 0 0 0

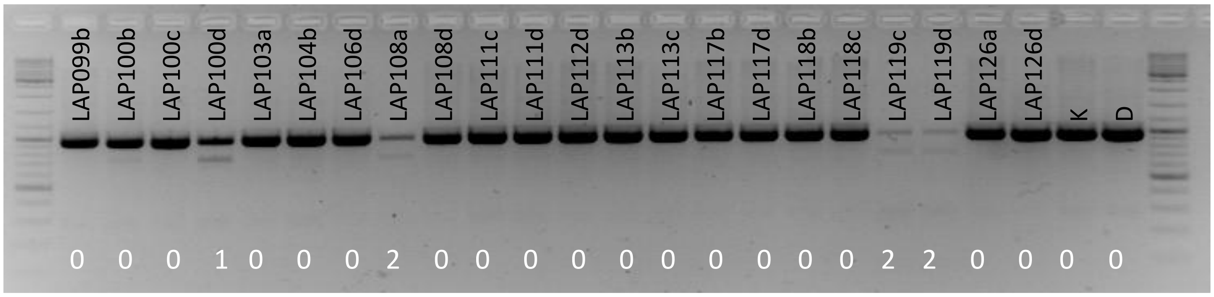
LAP099b LAP100b LAP100c LAP100d LAP103a LAP104b LAP106d LAP108a LAP108d LAP111c LAP111d LAP112d LAP113b LAP113c LAP117b LAP117d LAP118b LAP118c LAP119c LAP119d LAP126a LAP126d K D
0 0 0 1 0 0 0 2 0 0 0 0 0 0 0 0 0 0 2 2 0 0 0 0

PR_73

FTc2_F7 ACGTTGGTTTAGTAGCGTAAAATTC

FT1_F1 TCTGATCTCCTTCCACCACAAC

Plate 1

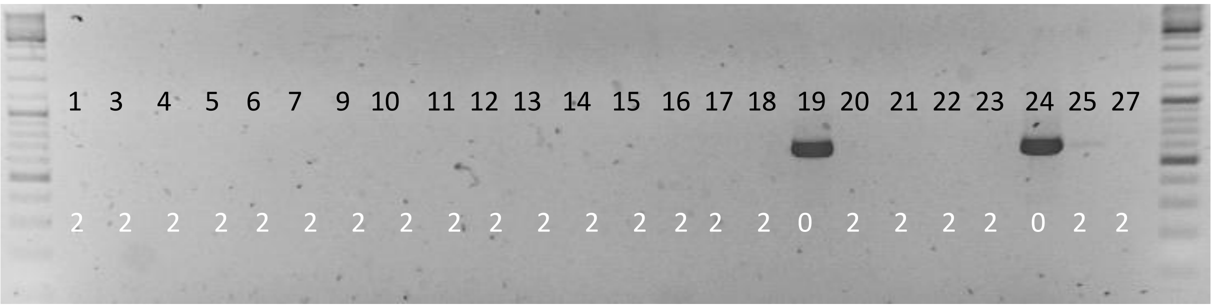

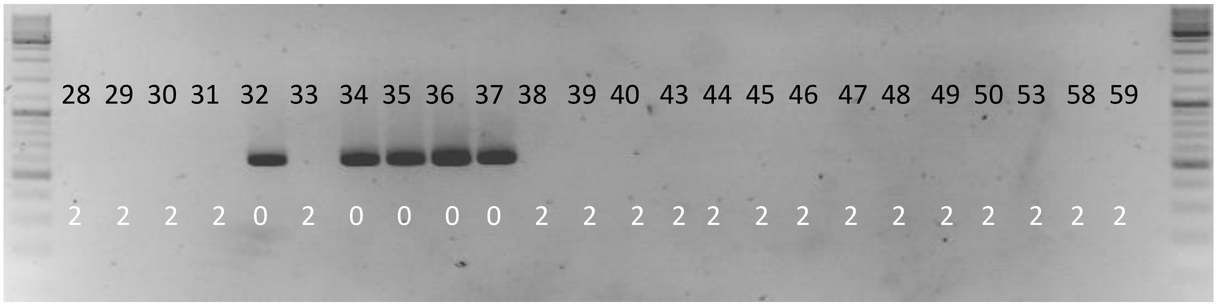

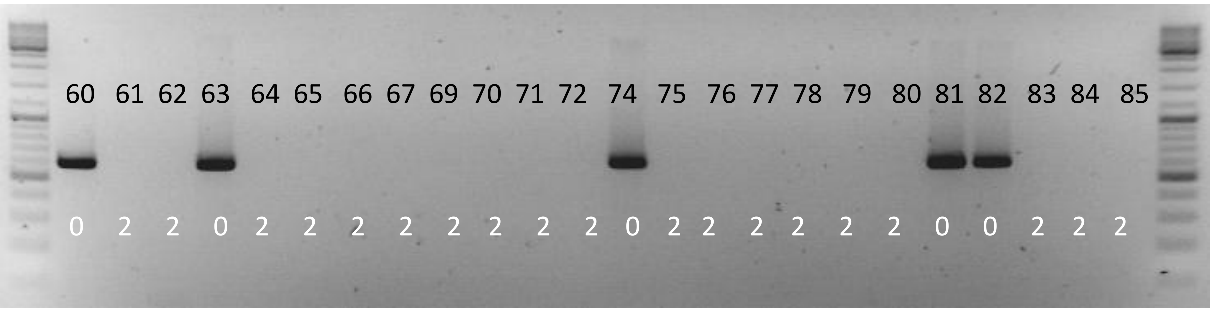

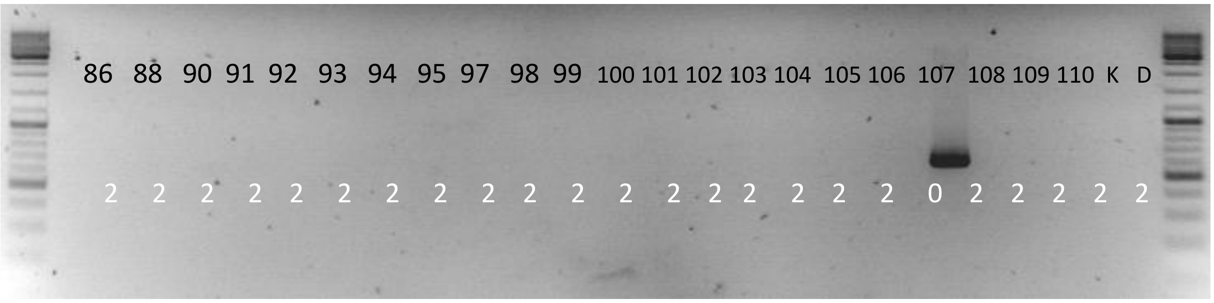

Plate 7

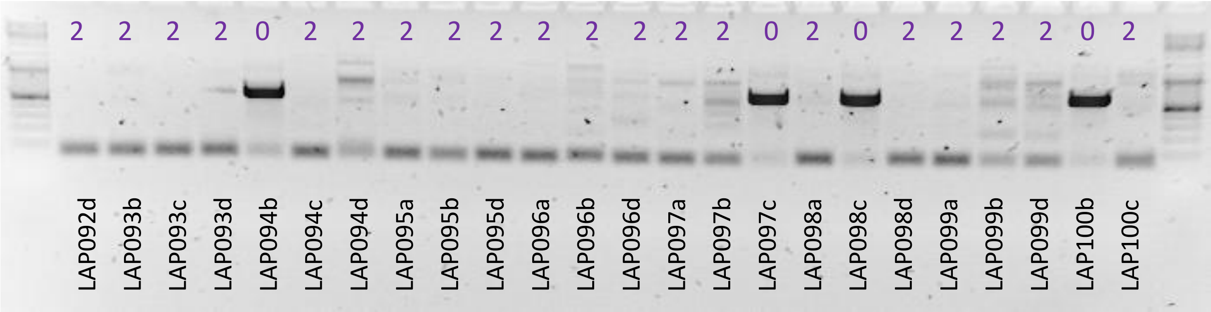
2 2 2 2 0 2 2 2 2 2 2 2 2 2 2 0 2 0 2 2 2 2 0 2
LAP092d LAP093b LAP093c LAP093d LAP094b LAP094c LAP094d LAP095a LAP095b LAP095d LAP096a LAP096b LAP096d LAP097a LAP097b LAP097c LAP098a LAP098c LAP098d LAP099a LAP099b LAP099d LAP100b LAP100c

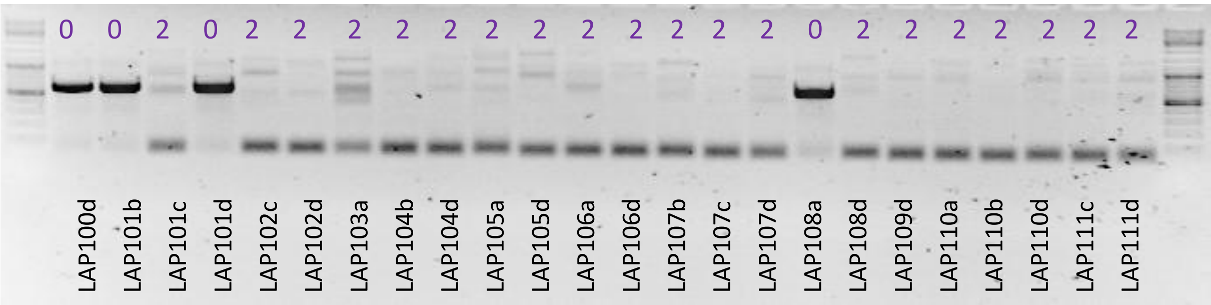
0 0 2 0 2 2 2 2 2 2 2 2 2 2 2 2 0 2 2 2 2 2 2 2
LAP100d LAP101b LAP101c LAP101d LAP102c LAP102d LAP103a LAP104b LAP104d LAP105a LAP105d LAP106a LAP106d LAP107b LAP107c LAP107d LAP108a LAP108d LAP109d LAP110a LAP110b LAP110d LAP111c LAP111d

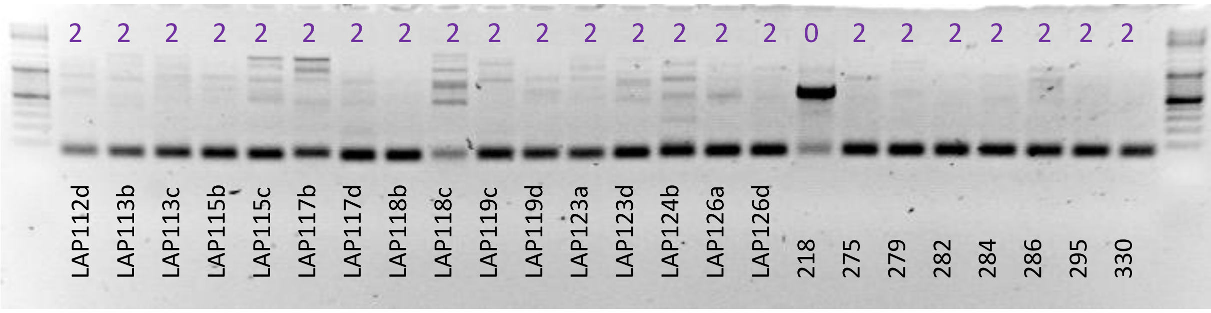
2 2 2 2 2 2 2 2 2 2 2 2 2 2 2 2 0 2 2 2 2 2 2 2
LAP112d LAP113b LAP113c LAP115b LAP115c LAP117b LAP117d LAP118b LAP118c LAP119c LAP119d LAP123a LAP123d LAP124b LAP126a LAP126d 218 275 279 282 284 286 295 330

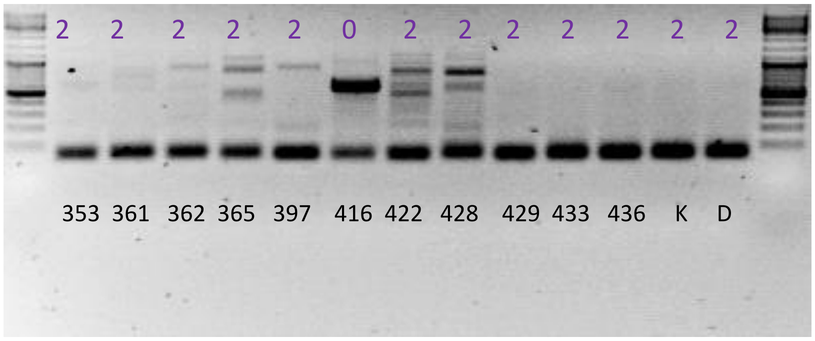
2 2 2 2 2 0 2 2 2 2 2 2 2
353 361 362 365 397 416 422 428 429 433 436 K D
