# Supplementary material for: Evaluation of the Global White Lupin Collection Reveals Significant Associations Between Homologous FLOWERING LOCUS T Indels and Flowering Time, Providing Validated Markers for Tracking Spring Ecotypes Within a Large Gene Pool
Source: Int J Mol Sci. 2025 Jul 17;26(14):6858. doi: 10.3390/ijms26146858 (PMC12295241; doi:10.3390/ijms26146858)
Supplement: Supplementary file 1 [file ijms-26-06858-s001.zip › Supplementary_Figure_S9_Correlation of PCR markers tagging LalbFTc2 gene indels with white lupin phenology.pdf]

Wojciech Bielski, Anna Surma, Michał Książkiewicz, Sandra Rychel-Bielska

Evaluation of the global white lupin collection reveals significant associations between homologous *FLOWERING LOCUS T* indels and flowering time, providing validated markers for tracking spring ecotypes within a large gene pool

International Journal of Molecular Sciences

Supplementary Figure S9. Correlation of PCR markers tagging *LalbFTc2* gene indels with white lupin phenology. Phenotyping was conducted without pre-sowing vernalization during the 2020 and 2021 growing seasons in a greenhouse located at the Institute of Plant Genetics, Polish Academy of Sciences in Poznań. Spearman′s rank correlation coefficient calculated for three phenology traits (the number of days to floral bud emergence (BE), start of flowering (SF), and end of flowering (EF)) is presented in color scale from -0.5 to 0.5, whereas the Bonferroni-corrected p-value is shown in the following scheme: ***, $p < 0.0001$; **, $0.0001 \le p < 0.001$; *, $0.001 \le p \le 0.05$; no symbol, $p > 0.05$ (not significant).

Marker	Variant	BE 2020	SF 2020	EF 2020	BE 2021	SF 2021	EF 2021
PR_73	indel 3						
PR_64	indel 1						
PR_57	PAV						

Scale	-0.50	-0.33	-0.17	0.00	0.17	0.33	0.50
